# Burden of liver cancer from 1990 to 2021 and modelled projection to 2040: insights from the global burden of disease study 2021

**DOI:** 10.3389/fonc.2026.1699519

**Published:** 2026-04-29

**Authors:** Yong Xie, Tianshi Lyu, Haitao Guan, Li Song, Xiaoqiang Tong, Yinghua Zou, Min Yang, Jian Wang

**Affiliations:** Department of Interventional Radiology and Vascular Surgery, Peking University First Hospital, Beijing, China

**Keywords:** deaths, disability-adjusted life-years, global burden of disease study, incidence, liver cancer

## Abstract

**Background:**

Liver cancer is a significant public health threat worldwide, yet data on its burden and trends are limited. This study aims to evaluate the global burden of liver cancer among global populations from 1990 to 2021, and to project its change to 2040.

**Methods:**

In this observational study, population-based registry data were extracted from the Global Burden of Diseases Study 2021. The database covers 204 countries and territories, ensuring global applicability of the results. Age-standardized rates of incidence, deaths, and disability-adjusted life-years (DALYs) were calculated per 100–000 population. Estimated rates of disease burden were calculated for 2040.

**Results:**

In 2021, global incident cases of liver cancer were 211460 (non-alcoholic steatohepatitis [NASH]-related), 1031830 (hepatitis B virus [HBV]-related), 770310 (hepatitis C virus [HCV]-related), 497720 (alcohol-related), 20240 (hepatoblastoma), and 114460 (other causes-related). HBV-related liver cancer exhibited the highest age-standardized rate of incidence (2.62 per 100–000 population; 95% uncertainty interval [UI], 2.15-3.19), deaths (2.30 per 100–000 population; 95% UI, 1.89-2.81), and DALYs (71.83 per 100–000 population; 95% UI, 59.65-87.25). In 2021, the 65–69 age group bore the heaviest burden, dominated by HBV-related cases. Higher sociodemographic indices correlated with greater burden for most subtypes. High-income Asia Pacific and East Asia had the highest regional burdens. Nationally, Mongolia, Taiwan (Province of China), Monaco, and Mali were most affected. Cross-country inequalities persisted. Projections to 2040 predict rising burden for all subtypes except hepatoblastoma, with NASH-related incident cases increasing by 51.97%.

**Conclusions:**

The global disease burden of all liver cancer subtypes, except hepatoblastoma, is increasing. There is an urgent need to strengthen interdisciplinary collaboration and integrate public health measures with clinical interventions to address the increasingly severe challenge of liver cancer.

## Introduction

Liver cancer remains a major global health challenge, with substantial morbidity and mortality worldwide (1–3). Its etiological diversity, including associations with non-alcoholic steatohepatitis (NASH), hepatitis B virus (HBV), hepatitis C virus (HCV), alcohol consumption, and other factors, contributes to complex epidemiological patterns (4). Over recent decades, shifts in population demographics, lifestyle changes, and varying access to healthcare have altered its burden across regions and populations.

HBV and HCV infections (5, 6) have long been recognized as primary drivers, but the rising prevalence of metabolic syndrome has elevated NASH-related liver cancer as a growing concern (7, 8). Demographic aging further exacerbates the burden, with older adults facing higher risks of advanced-stage diagnosis and poorer outcomes (9). Disparities in disease burden persist across sociodemographic indices (SDI), reflecting inequalities in prevention, screening, and treatment access. Understanding these evolving trends is critical for guiding public health strategies.

To contextualize the observed epidemiological trends, this study integrates two key theoretical frameworks: the Global Health Transition Model and the Social Determinants of Health (SDH) framework. The Global Health Transition Model posits that as societies advance through socioeconomic development (reflected in SDI), the burden of infectious disease-related liver cancer (e.g., HBV/HCV-related) may shift, while non-communicable disease (NCD)-related subtypes (e.g., NASH-related, alcohol-related) emerge as major threats—aligning with global shifts in lifestyle and demographic aging. Meanwhile, the SDH framework highlights that structural factors, including income inequality, educational attainment, healthcare system capacity, and social policies, shape disparities in liver cancer burden by influencing risk factor exposure (e.g., obesity, alcohol consumption) and access to prevention/treatment services.

The latest spatial patterns and temporal trends of liver cancer burden in the world, which are essential for targeted public policymaking, are lacking. We aim to investigate the most updated trends in liver cancer burden across subgroups of age cohorts, geographical regions, and SDI level and further model a predicted burden by 2040.

## Methods

### Data source

Data on liver cancer from 1990 to 2021 were sourced from the Global Burden of Disease (GBD) Study 2021 database, accessible via the Institute for Health Metrics and Evaluation website (Institute for Health Metrics and Evaluation. Global Health Data Exchange. Accessed June 1, 2024. https://ghdx.healthdata.org/gbd-results-tool). Data collection and analysis adhered to the Strengthening the Reporting of Observational Studies in Epidemiology (STROBE) reporting guidelines. We conducted a systematic analysis of incidence, deaths, and disability-adjusted life-years (DALYs) associated with liver cancer, examining these metrics at global, regional, and national levels. DALYs—quantified as the sum of years of life lost and years lived with disability—serve as a comprehensive metric for assessing the overall burden of a disease or health condition (10–12). We also used the Socio-demographic Index (SDI), a composite measure that quantifies the sociodemographic status of a country or region based on average income, educational attainment, and fertility rates (13, 14). Ethical approval was not required for this study, as the analysis of deidentified, publicly available data was classified as non-human participants research by the Peking University First Hospital, and informed consent was therefore waived.

### Statistical analysis

Data are presented as numerical counts and age-standardized rates (ASRs) per 100000 population, accompanied by 95% uncertainty intervals (UIs). These UIs were derived from the 2.5th and 97.5th ordered values of 1,000 draws from the posterior distribution (15, 16). Temporal trends in ASRs were quantified using estimated annual percentage changes (EAPCs) (17, 18).

Inequality analyses were also performed. The slope index of inequality (SII) and concentration index (CI) are standardized indicators for measuring absolute and relative gradient inequalities, respectively (19, 20). The SII was calculated via regression analysis, correlating country-level DALYs rates with their relative SDI positions—defined as the midpoint of the population in a cumulative distribution ranked by SDI (21, 22). The CI was derived by fitting a Lorenz concentration curve to the observed cumulative relative distribution of populations ranked by SDI and the corresponding disease DALYs, followed by numerical integration of the area under the curve (23, 24).

Furthermore, we used an autoregressive integrated moving average (ARIMA) model implemented within the integrated nested Laplace approximations framework to predict future trends through 2040 (25, 26). Detailed analytical methods are provided in the **Supplementary Methods**. All analyses and graphic visualizations were conducted using R statistical software (version 4.2.0; R Foundation for Statistical Computing). A p value less than 0.05 was regarded as statistically significant.

## Results

### Global trends

In 2021, the global number of newly diagnosed liver cancer cases attributed to different etiologies was as follows: 21146 cases related to NASH, 103183 cases related to HBV, 77031 cases related to HCV, 49772 cases related to alcohol consumption, 2024 cases of hepatoblastoma, and 11446 cases related to other causes (**Tables 1**, **2**; **Supplementary Tables 1**-**4**). HBV-related liver cancer had the highest ASRs of incidence (2.62 per 100000 population; 95% UI, 2.15-3.19). It also accounted for the largest number of deaths and DALYs, with approximately 90597 deaths and 2834100 DALYs. The corresponding ASRs of mortality and DALYs were 2.30 per 100000 population (95% UI, 1.89-2.81) and 71.83 per 100000 population (95% UI, 59.65-87.25), respectively. The 2021 global assessment of liver cancer rankings revealed significant regional disparities in ASRs of incidence, deaths, and DALYs. HBV-related liver cancer consistently ranked first in global incidence and held the top position in four major regions, including Southeast Asia, East Asia, and Eastern Sub-Saharan Africa (**Supplementary Figure 1**).

**Table 1:** Global burden of liver cancer due to non-alcoholic steatohepatitis (NASH) in 1990 and 2021, categorized by sex, region, and SDI.

Characteristics	Incidence					Deaths					DALYs				
	1990 No. (95% UI)	2021 No. (95% UI)	1990 ASR per 100 000 (95% UI)	2021 ASR per 100 000 (95% UI)	EAPC (95% CI)	1990 No. (95% UI)	2021 No. (95% UI)	1990 ASR per 100 000 (95% UI)	2021 ASR per 100 000 (95% UI)	EAPC (95% CI)	1990 No. (95% UI)	2021 No. (95% UI)	1990 ASR per 100 000 (95% UI)	2021 ASR per 100 000 (95% UI)	EAPC (95% CI)
Global	7206.96 (5735.48-8927.12)	21145.69 (17016.32-25564.73)	0.27 (0.22-0.33)	0.54 (0.43-0.65)	2.24 (2.16,2.31)	7337.57 (5810.52-9079.45)	20462.45 (16480.68-24804.99)	0.28 (0.22-0.34)	0.52 (0.42-0.63)	2.08 (1.99,2.16)	202006.30 (160675.39-249995.57)	497737.29 (404399.61-600894.34)	7.57 (6.02-9.37)	12.61 (10.25-15.23)	1.63 (1.54,1.72)
Sex																
Male	3563.82 (2814.84-4414.90)	10876.98 (8604.67-13480.54)	0.27 (0.21-0.33)	0.55 (0.43-0.68)	2.34 (2.27,2.42)	3558.82 (2798.99-4432.06)	10234.40 (8090.03-12681.46)	0.27 (0.21-0.33)	0.52 (0.41-0.64)	2.14 (2.06,2.23)	102591.10 (81052.82-126447.20)	260294.22 (202723.83-326264.11)	7.64 (6.04-9.42)	13.15 (10.24-16.48)	1.71 (1.62,1.80)
Female	3643.14 (2848.01-4616.84)	10268.71 (8377.04-12497.57)	0.28 (0.22-0.35)	0.52 (0.43-0.64)	2.12 (2.05,2.20)	3778.75 (2935.04-4810.03)	10228.05 (8315.84-12501.50)	0.29 (0.22-0.36)	0.52 (0.42-0.64)	2.02 (1.92,2.11)	99415.20 (79774.27-125399.73)	237443.06 (193726.26-287750.66)	7.51 (6.03-9.47)	12.08 (9.85-14.64)	1.55 (1.45,1.65)
SDI category																
Low SDI	693.66 (448.86-1055.26)	1521.12 (1078.21-2092.73)	0.28 (0.18-0.42)	0.27 (0.19-0.37)	-0.29 (-0.40,-0.17)	732.34 (471.08-1121.68)	1610.98 (1141.74-2227.39)	0.29 (0.19-0.45)	0.29 (0.20-0.40)	-0.27 (-0.39,-0.15)	20930.30 (13896.45-30983.28)	45726.65 (33041.28-62486.12)	8.35 (5.54-12.36)	8.18 (5.91-11.18)	-0.31 (-0.43,-0.19)
Low-middle SDI	1022.34 (756.45-1428.20)	3344.83 (2664.72-4122.45)	0.18 (0.13-0.25)	0.35 (0.28-0.43)	2.21 (2.08,2.35)	1071.99 (786.13-1505.41)	3520.12 (2805.82-4340.53)	0.18 (0.14-0.26)	0.37 (0.29-0.45)	2.19 (2.05,2.33)	31268.68 (23810.91-42440.29)	96730.19 (77314.00-118605.05)	5.38 (4.10-7.31)	10.07 (8.05-12.35)	1.98 (1.87,2.10)
Middle SDI	2216.16 (1796.62-2689.21)	6891.73 (5448.29-8391.38)	0.26 (0.21-0.31)	0.56 (0.45-0.69)	2.74 (2.57,2.90)	2296.07 (1854.66-2801.68)	6806.28 (5408.89-8341.38)	0.27 (0.22-0.33)	0.56 (0.44-0.68)	2.57 (2.41,2.72)	67247.42 (54702.84-81356.99)	171852.20 (135623.88-208708.52)	7.81 (6.35-9.44)	14.04 (11.08-17.05)	2.02 (1.87,2.16)
High-middle SDI	1501.74 (1226.44-1813.27)	3837.50 (3021.97-4644.80)	0.28 (0.23-0.34)	0.59 (0.46-0.71)	2.46 (2.33,2.60)	1550.84 (1269.85-1874.20)	3718.93 (2975.96-4544.60)	0.29 (0.24-0.35)	0.57 (0.46-0.70)	2.25 (2.12,2.37)	43070.04 (35385.24-51809.43)	88146.39 (69712.44-106368.87)	8.10 (6.65-9.74)	13.52 (10.69-16.31)	1.64 (1.50,1.77)
High SDI	1767.06 (1416.50-2195.60)	5537.34 (4361.68-6896.58)	0.40 (0.32-0.50)	1.01 (0.80-1.26)	2.85 (2.65,3.05)	1679.90 (1331.70-2098.11)	4792.32 (3705.05-5987.41)	0.38 (0.30-0.48)	0.88 (0.68-1.09)	2.62 (2.46,2.79)	39329.91 (31599.49-49346.08)	94959.01 (76178.44-118667.96)	8.94 (7.19-11.22)	17.36 (13.93-21.69)	2.07 (1.92,2.22)
Region																
Andean Latin America	25.78 (18.18-35.21)	95.78 (62.50-137.78)	0.14 (0.10-0.19)	0.29 (0.19-0.42)	2.48 (2.21,2.76)	28.25 (19.61-38.59)	106.29 (69.55-153.18)	0.15 (0.10-0.20)	0.32 (0.21-0.46)	2.49 (2.19,2.78)	704.56 (511.25-964.26)	2311.80 (1517.14-3302.27)	3.71 (2.69-5.08)	6.99 (4.59-9.99)	1.98 (1.67,2.28)
Australasia	17.96 (13.27-23.88)	160.75 (115.39-218.32)	0.18 (0.13-0.24)	1.04 (0.75-1.41)	5.81 (5.58,6.04)	17.91 (13.00-24.04)	148.45 (105.37-201.96)	0.18 (0.13-0.24)	0.96 (0.68-1.30)	5.47 (5.28,5.66)	432.55 (324.91-570.97)	3122.45 (2247.32-4205.53)	4.27 (3.20-5.63)	20.17 (14.52-27.17)	5.10 (4.95,5.25)
Caribbean	24.53 (17.14-33.91)	56.08 (38.11-77.69)	0.14 (0.10-0.19)	0.24 (0.16-0.33)	1.37 (1.08,1.66)	26.67 (18.41-36.78)	60.56 (40.89-83.71)	0.15 (0.10-0.21)	0.26 (0.17-0.35)	1.39 (1.08,1.71)	636.24 (459.77-861.81)	1381.21 (951.98-1882.04)	3.61 (2.61-4.88)	5.82 (4.01-7.93)	1.28 (0.97,1.58)
Central Asia	117.09 (81.98-165.25)	223.25 (149.44-322.48)	0.34 (0.24-0.48)	0.47 (0.31-0.67)	0.95 (0.86,1.04)	124.11 (86.43-174.31)	237.05 (159.14-339.53)	0.36 (0.25-0.50)	0.49 (0.33-0.71)	0.92 (0.81,1.02)	3426.14 (2415.38-4813.98)	6298.87 (4278.43-9061.18)	9.89 (6.97-13.89)	13.15 (8.93-18.92)	0.73 (0.62,0.84)
Central Europe	183.76 (127.17-256.44)	304.82 (215.71-411.42)	0.29 (0.20-0.41)	0.53 (0.37-0.71)	2.04 (1.87,2.21)	200.60 (138.65-280.79)	334.67 (237.51-454.13)	0.32 (0.22-0.45)	0.58 (0.41-0.79)	2.10 (1.88,2.31)	4676.81 (3343.37-6454.09)	6859.87 (4896.38-9279.25)	7.48 (5.35-10.32)	11.90 (8.50-16.10)	1.64 (1.43,1.84)
Central Latin America	96.18 (74.83-123.19)	369.31 (292.60-463.40)	0.12 (0.09-0.15)	0.29 (0.23-0.37)	3.27 (2.95,3.59)	103.56 (79.75-133.57)	401.49 (315.60-507.09)	0.13 (0.10-0.16)	0.32 (0.25-0.40)	3.23 (2.88,3.58)	2712.43 (2150.58-3404.45)	9436.76 (7447.52-11601.81)	3.30 (2.62-4.14)	7.46 (5.89-9.17)	2.86 (2.51,3.21)
Central Sub-Saharan Africa	55.19 (23.70-119.11)	111.84 (46.57-251.94)	0.20 (0.09-0.43)	0.16 (0.07-0.37)	-1.12 (-1.36,-0.87)	57.20 (24.57-125.08)	116.38 (48.04-267.56)	0.21 (0.09-0.46)	0.17 (0.07-0.39)	-1.10 (-1.33,-0.87)	1774.12 (760.95-3834.36)	3611.18 (1520.07-8241.12)	6.46 (2.77-13.95)	5.27 (2.22-12.04)	-1.06 (-1.30,-0.83)
East Asia	2109.92 (1688.85-2603.25)	5929.23 (4547.66-7528.78)	0.35 (0.28-0.43)	0.81 (0.62-1.02)	3.09 (2.87,3.31)	2146.17 (1720.10-2651.82)	5467.98 (4228.79-6898.85)	0.35 (0.28-0.44)	0.74 (0.57-0.94)	2.75 (2.54,2.96)	65064.00 (52547.76-79802.97)	134401.52 (102869.68-170106.53)	10.69 (8.63-13.11)	18.25 (13.97-23.10)	1.94 (1.74,2.14)
Eastern Europe	181.87 (152.88-215.45)	367.71 (307.59-431.91)	0.16 (0.13-0.19)	0.36 (0.30-0.42)	2.68 (2.43,2.93)	193.37 (161.60-229.82)	397.92 (333.92-467.44)	0.17 (0.14-0.20)	0.38 (0.32-0.45)	2.82 (2.52,3.11)	4962.24 (4180.71-5843.08)	9045.83 (7586.99-10639.98)	4.38 (3.69-5.16)	8.75 (7.34-10.29)	2.36 (2.07,2.66)
Eastern Sub-Saharan Africa	251.92 (176.61-354.84)	596.51 (407.39-825.36)	0.26 (0.19-0.37)	0.28 (0.19-0.39)	-0.13 (-0.25,-0.01)	265.78 (185.43-375.59)	630.32 (432.72-869.49)	0.28 (0.19-0.39)	0.30 (0.20-0.41)	-0.10 (-0.22,0.02)	7677.99 (5540.71-10571.85)	18144.09 (12325.90-24942.21)	8.05 (5.81-11.08)	8.52 (5.79-11.71)	-0.13 (-0.25,-0.01)
High-income Asia Pacific	801.09 (642.71-994.63)	1583.57 (1207.17-2029.24)	0.92 (0.74-1.15)	1.71 (1.30-2.19)	1.43 (1.06,1.80)	718.15 (565.77-917.47)	1244.83 (931.80-1605.89)	0.83 (0.65-1.06)	1.34 (1.00-1.73)	1.12 (0.74,1.50)	18139.75 (14499.43-22804.87)	21567.68 (16479.16-28173.78)	20.92 (16.73-26.31)	23.26 (17.77-30.38)	-0.18 (-0.56,0.21)
High-income North America	400.91 (341.26-468.46)	1952.24 (1624.80-2321.18)	0.28 (0.24-0.33)	1.05 (0.88-1.25)	4.34 (4.23,4.45)	372.20 (315.02-436.28)	1650.81 (1362.76-1957.17)	0.26 (0.22-0.31)	0.89 (0.74-1.06)	4.06 (3.98,4.15)	7965.00 (6834.21-9190.12)	34710.63 (29280.76-41080.00)	5.66 (4.86-6.53)	18.75 (15.82-22.19)	4.14 (4.03,4.24)
North Africa and Middle East	329.30 (206.58-541.79)	1429.49 (1010.64-1962.13)	0.19 (0.12-0.32)	0.46 (0.32-0.63)	2.78 (2.51,3.05)	347.13 (218.82-570.08)	1483.24 (1032.83-2033.15)	0.20 (0.13-0.34)	0.48 (0.33-0.65)	2.68 (2.40,2.96)	9777.02 (6348.69-16022.17)	39946.59 (28200.24-54253.57)	5.76 (3.74-9.45)	12.82 (9.05-17.42)	2.55 (2.30,2.80)
Oceania	5.27 (2.83-10.43)	12.23 (6.91-22.25)	0.16 (0.09-0.32)	0.18 (0.10-0.32)	0.11 (-0.01,0.23)	5.44 (2.94-10.88)	12.54 (7.07-23.07)	0.17 (0.09-0.33)	0.18 (0.10-0.33)	0.07 (-0.05,0.19)	166.65 (89.47-328.38)	374.97 (214.60-681.23)	5.09 (2.73-10.03)	5.38 (3.08-9.78)	0.05 (-0.06,0.16)
South Asia	659.00 (534.32-789.35)	2579.56 (2167.81-3073.43)	0.12 (0.10-0.14)	0.28 (0.23-0.33)	2.80 (2.67,2.93)	687.44 (554.24-824.68)	2734.64 (2299.89-3243.23)	0.13 (0.10-0.15)	0.30 (0.25-0.35)	2.82 (2.68,2.96)	20415.46 (16517.50-24290.57)	73054.17 (62426.19-86623.37)	3.73 (3.02-4.44)	7.91 (6.76-9.38)	2.42 (2.31,2.53)
Southeast Asia	710.27 (512.85-970.37)	2086.87 (1460.78-2853.99)	0.31 (0.22-0.42)	0.60 (0.42-0.82)	2.13 (2.07,2.18)	741.99 (535.01-1009.33)	2133.35 (1506.31-2916.58)	0.32 (0.23-0.43)	0.61 (0.43-0.84)	2.06 (2.01,2.12)	21356.97 (15843.29-28846.72)	56649.54 (39258.40-78308.32)	9.18 (6.81-12.39)	16.22 (11.24-22.43)	1.79 (1.74,1.85)
Southern Latin America	14.90 (9.98-21.06)	80.37 (53.21-113.21)	0.06 (0.04-0.09)	0.24 (0.16-0.33)	5.07 (4.89,5.26)	16.13 (10.76-22.94)	85.70 (56.66-120.91)	0.07 (0.04-0.09)	0.25 (0.17-0.36)	5.06 (4.84,5.29)	378.27 (261.03-530.39)	1863.47 (1281.98-2594.32)	1.53 (1.05-2.14)	5.51 (3.79-7.66)	4.80 (4.57,5.03)
Southern Sub-Saharan Africa	84.31 (51.36-127.12)	294.76 (232.76-364.62)	0.32 (0.20-0.49)	0.73 (0.58-0.91)	2.22 (1.64,2.81)	88.85 (54.23-133.90)	311.54 (245.98-384.57)	0.34 (0.21-0.51)	0.78 (0.61-0.96)	2.20 (1.60,2.79)	2653.73 (1655.97-3984.44)	8739.54 (6972.22-10832.75)	10.13 (6.32-15.20)	21.77 (17.36-26.98)	2.03 (1.37,2.69)
Tropical Latin America	53.81 (46.84-61.60)	196.18 (165.18-228.47)	0.07 (0.06-0.08)	0.17 (0.15-0.20)	3.64 (3.31,3.98)	57.28 (49.54-65.92)	211.71 (177.76-246.28)	0.08 (0.06-0.09)	0.19 (0.16-0.22)	3.77 (3.44,4.10)	1574.61 (1384.00-1780.84)	5116.90 (4346.99-5911.02)	2.06 (1.81-2.33)	4.50 (3.82-5.20)	3.37 (3.06,3.68)
Western Europe	585.43 (439.04-774.88)	1666.30 (1219.95-2244.53)	0.30 (0.23-0.40)	0.76 (0.56-1.03)	3.13 (3.00,3.26)	608.59 (453.37-806.14)	1579.72 (1127.54-2136.09)	0.32 (0.24-0.42)	0.72 (0.52-0.98)	2.80 (2.70,2.90)	12858.78 (9838.80-16762.18)	29919.20 (22118.49-39354.95)	6.69 (5.12-8.72)	13.68 (10.11-18.00)	2.43 (2.32,2.53)
Western Sub-Saharan Africa	498.47 (293.88-831.79)	1048.80 (779.69-1395.89)	0.52 (0.30-0.86)	0.43 (0.32-0.57)	-0.93 (-1.06,-0.80)	530.76 (311.48-890.67)	1113.27 (827.19-1472.23)	0.55 (0.32-0.92)	0.45 (0.34-0.60)	-0.93 (-1.06,-0.79)	14652.96 (8732.42-23964.63)	31181.02 (23080.00-41618.47)	15.17 (9.04-24.81)	12.73 (9.42-16.99)	-0.88 (-1.02,-0.73)

UI, uncertainty interval; ASR, age-standardised rate per 100,000; EAPC, estimated annual percentage change; CI, confidence interval; DALYs, disability-adjusted life-year; SDI, socio-demographic index.

**Table 2:** Global burden of liver cancer due to hepatitis B in 1990 and 2021, categorized by sex, region, and SDI.

Characteristics	Incidence					Deaths					DALYs				
	1990 No. (95% UI)	2021 No. (95% UI)	1990 ASR per 100 000 (95% UI)	2021 ASR per 100 000 (95% UI)	EAPC (95% CI)	1990 No. (95% UI)	2021 No. (95% UI)	1990 ASR per 100 000 (95% UI)	2021 ASR per 100 000 (95% UI)	EAPC (95% CI)	1990 No. (95% UI)	2021 No. (95% UI)	1990 ASR per 100 000 (95% UI)	2021 ASR per 100 000 (95% UI)	EAPC (95% CI)
Global	54739.34 (47410.04-63682.79)	103182.87 (84700.45-126024.95)	2.05 (1.78-2.39)	2.62 (2.15-3.19)	0.76 (0.64,0.87)	53257.04 (45969.80-62145.62)	90597.16 (74448.24-110842.64)	2.00 (1.72-2.33)	2.30 (1.89-2.81)	0.39 (0.27,0.51)	1874089.71 (1627816.78-2181255.39)	2834099.65 (2353443.24-3442535.68)	70.27 (61.04-81.79)	71.83 (59.65-87.25)	-0.04 (-0.19,0.10)
Sex																
Male	44444.10 (38115.68-51668.43)	84489.99 (67918.21-105037.87)	3.31 (2.84-3.85)	4.27 (3.43-5.31)	0.81 (0.68,0.93)	43019.78 (36818.80-50301.67)	73252.82 (59198.38-91186.91)	3.20 (2.74-3.75)	3.70 (2.99-4.61)	0.40 (0.27,0.54)	1536019.62 (1313102.79-1782427.00)	2334453.16 (1897389.93-2901642.04)	114.38 (97.78-132.73)	117.92 (95.84-146.57)	-0.01 (-0.16,0.14)
Female	10295.24 (8240.49-12707.78)	18692.88 (15010.72-23121.17)	0.78 (0.62-0.96)	0.95 (0.76-1.18)	0.60 (0.53,0.67)	10237.26 (8164.72-12678.50)	17344.34 (13837.84-21456.70)	0.77 (0.62-0.96)	0.88 (0.70-1.09)	0.39 (0.30,0.47)	338070.09 (275499.77-408780.61)	499646.49 (407284.71-608019.34)	25.54 (20.81-30.88)	25.41 (20.72-30.93)	-0.14 (-0.24,-0.04)
SDI category																
Low SDI	3406.21 (2244.00-4878.36)	5651.64 (4215.69-7442.55)	1.36 (0.90-1.95)	1.01 (0.75-1.33)	-1.19 (-1.26,-1.11)	3446.89 (2268.99-4946.96)	5675.80 (4239.27-7492.99)	1.38 (0.91-1.97)	1.02 (0.76-1.34)	-1.19 (-1.28,-1.11)	118533.00 (78910.32-166159.78)	201828.52 (152453.11-269753.44)	47.29 (31.48-66.29)	36.13 (27.29-48.28)	-1.09 (-1.18,-1.00)
Low-middle SDI	4331.71 (3390.51-5605.72)	9109.78 (7264.41-11455.62)	0.75 (0.58-0.97)	0.95 (0.76-1.19)	0.58 (0.52,0.63)	4347.21 (3402.57-5640.41)	9124.41 (7301.32-11532.14)	0.75 (0.59-0.97)	0.95 (0.76-1.20)	0.57 (0.50,0.63)	153693.28 (120583.59-196203.57)	307811.67 (247180.78-384894.41)	26.47 (20.77-33.79)	32.05 (25.73-40.07)	0.40 (0.33,0.46)
Middle SDI	23203.54 (19787.39-27063.53)	46209.47 (36810.40-58632.66)	2.69 (2.30-3.14)	3.77 (3.01-4.79)	1.25 (1.10,1.41)	22763.81 (19477.15-26476.54)	40543.47 (32449.10-50820.29)	2.64 (2.26-3.07)	3.31 (2.65-4.15)	0.85 (0.72,0.99)	823858.87 (700174.56-965665.00)	1291066.44 (1040430.66-1627690.51)	95.64 (81.28-112.10)	105.46 (84.98-132.95)	0.38 (0.24,0.52)
High-middle SDI	15201.95 (12694.80-18102.76)	27681.65 (21795.16-35027.54)	2.86 (2.39-3.40)	4.25 (3.34-5.37)	1.15 (0.94,1.36)	14826.77 (12349.73-17632.28)	23947.59 (18954.75-30219.83)	2.79 (2.32-3.32)	3.67 (2.91-4.63)	0.70 (0.47,0.93)	527691.06 (440343.02-623711.13)	743542.92 (588916.07-941436.07)	99.23 (82.81-117.29)	114.04 (90.32-144.39)	0.18 (-0.08,0.44)
High SDI	8573.10 (7203.44-10102.48)	14494.60 (11865.93-17414.78)	1.95 (1.64-2.30)	2.65 (2.17-3.18)	0.87 (0.67,1.06)	7849.31 (6570.69-9253.32)	11270.73 (9194.06-13600.86)	1.78 (1.49-2.10)	2.06 (1.68-2.49)	0.33 (0.18,0.48)	249568.33 (212289.41-295073.22)	288771.61 (239608.73-345678.96)	56.75 (48.27-67.10)	52.79 (43.80-63.19)	-0.42 (-0.59,-0.24)
Region																
Andean Latin America	123.24 (95.44-155.47)	282.75 (200.12-388.68)	0.65 (0.50-0.82)	0.86 (0.61-1.18)	0.81 (0.59,1.03)	127.43 (98.40-160.15)	293.77 (205.75-408.49)	0.67 (0.52-0.84)	0.89 (0.62-1.24)	0.78 (0.54,1.02)	4185.09 (3361.19-5092.47)	8344.07 (5947.35-11245.89)	22.03 (17.69-26.81)	25.23 (17.99-34.01)	0.23 (-0.02,0.48)
Australasia	37.52 (27.89-50.14)	188.63 (132.06-264.34)	0.37 (0.28-0.49)	1.22 (0.85-1.71)	3.90 (3.69,4.12)	34.46 (25.07-47.46)	152.68 (106.07-215.35)	0.34 (0.25-0.47)	0.99 (0.69-1.39)	3.32 (3.14,3.50)	1091.87 (810.76-1431.06)	4185.45 (2915.68-5758.94)	10.77 (8.00-14.12)	27.04 (18.83-37.20)	2.89 (2.73,3.06)
Caribbean	69.50 (50.01-93.05)	117.20 (82.42-158.68)	0.39 (0.28-0.53)	0.49 (0.35-0.67)	0.39 (0.09,0.70)	70.93 (50.88-96.34)	117.98 (83.10-159.65)	0.40 (0.29-0.55)	0.50 (0.35-0.67)	0.36 (0.04,0.68)	2231.34 (1664.69-2898.94)	3560.85 (2583.10-4762.75)	12.64 (9.43-16.43)	15.01 (10.89-20.07)	0.23 (-0.07,0.53)
Central Asia	526.14 (382.34-696.34)	699.47 (485.60-969.57)	1.52 (1.10-2.01)	1.46 (1.01-2.02)	-0.48 (-0.58,-0.39)	528.92 (381.63-701.31)	702.53 (483.07-978.46)	1.53 (1.10-2.02)	1.47 (1.01-2.04)	-0.53 (-0.62,-0.44)	18202.14 (13713.64-24074.00)	23380.98 (16439.27-32550.12)	52.52 (39.57-69.47)	48.81 (34.32-67.95)	-0.63 (-0.72,-0.53)
Central Europe	565.08 (401.12-748.91)	638.56 (450.42-872.63)	0.90 (0.64-1.20)	1.11 (0.78-1.51)	0.57 (0.45,0.68)	579.80 (412.48-775.51)	655.99 (462.35-905.41)	0.93 (0.66-1.24)	1.14 (0.80-1.57)	0.59 (0.44,0.74)	17432.86 (12603.90-22492.55)	17338.19 (12161.26-23274.72)	27.87 (20.15-35.96)	30.08 (21.10-40.39)	0.15 (0.00,0.30)
Central Latin America	201.91 (153.13-259.84)	434.65 (324.08-579.64)	0.25 (0.19-0.32)	0.34 (0.26-0.46)	1.12 (0.77,1.47)	205.58 (155.90-265.49)	442.67 (327.69-595.06)	0.25 (0.19-0.32)	0.35 (0.26-0.47)	1.02 (0.62,1.42)	6985.45 (5460.70-8667.34)	13448.73 (10167.24-17540.74)	8.50 (6.64-10.54)	10.63 (8.04-13.87)	0.64 (0.26,1.02)
Central Sub-Saharan Africa	230.61 (96.32-537.28)	365.34 (162.89-869.41)	0.84 (0.35-1.96)	0.53 (0.24-1.27)	-1.77 (-1.92,-1.62)	229.90 (96.10-533.83)	362.00 (160.55-880.64)	0.84 (0.35-1.94)	0.53 (0.23-1.29)	-1.78 (-1.93,-1.64)	8591.42 (3620.25-20374.69)	13866.61 (6247.56-34053.89)	31.26 (13.17-74.14)	20.25 (9.13-49.74)	-1.68 (-1.81,-1.55)
East Asia	32544.07 (26996.67-38726.49)	61089.77 (47914.36-78572.18)	5.35 (4.43-6.36)	8.30 (6.51-10.67)	1.55 (1.38,1.71)	31662.55 (26283.17-37597.86)	51678.93 (40462.24-66128.22)	5.20 (4.32-6.18)	7.02 (5.49-8.98)	1.04 (0.86,1.22)	1153018.21 (958034.84-1370661.13)	1623526.00 (1273015.00-2096975.66)	189.42 (157.39-225.17)	220.47 (172.87-284.77)	0.48 (0.28,0.68)
Eastern Europe	608.94 (507.22-727.02)	823.18 (676.65-1002.29)	0.54 (0.45-0.64)	0.80 (0.65-0.97)	1.15 (0.93,1.37)	613.37 (511.22-731.34)	827.58 (682.80-1003.51)	0.54 (0.45-0.65)	0.80 (0.66-0.97)	1.23 (0.96,1.50)	19944.70 (16927.56-23667.51)	24771.67 (20487.92-30013.94)	17.61 (14.95-20.90)	23.96 (19.82-29.03)	0.98 (0.67,1.30)
Eastern Sub-Saharan Africa	803.28 (560.37-1131.72)	1543.28 (1060.68-2336.25)	0.84 (0.59-1.19)	0.72 (0.50-1.10)	-0.90 (-1.07,-0.72)	810.91 (565.02-1145.64)	1544.73 (1057.28-2353.97)	0.85 (0.59-1.20)	0.73 (0.50-1.10)	-0.89 (-1.07,-0.71)	28608.53 (20278.01-39665.58)	56177.23 (38657.83-84301.92)	29.98 (21.25-41.57)	26.37 (18.15-39.57)	-0.79 (-0.98,-0.61)
High-income Asia Pacific	5649.44 (4479.50-6829.33)	7409.17 (5987.01-9081.29)	6.52 (5.17-7.88)	7.99 (6.46-9.79)	0.40 (0.21,0.60)	5102.57 (3975.42-6199.44)	5369.41 (4312.36-6564.24)	5.89 (4.59-7.15)	5.79 (4.65-7.08)	-0.31 (-0.48,-0.14)	164217.95 (129642.07-200942.68)	130378.46 (106196.42-160157.48)	189.43 (149.55-231.79)	140.61 (114.53-172.73)	-1.28 (-1.48,-1.07)
High-income North America	526.27 (455.05-605.51)	1897.69 (1569.05-2250.95)	0.37 (0.32-0.43)	1.03 (0.85-1.22)	3.35 (3.10,3.60)	432.81 (371.17-499.69)	1419.68 (1165.22-1679.31)	0.31 (0.26-0.36)	0.77 (0.63-0.91)	3.12 (2.96,3.28)	12673.55 (11040.49-14444.21)	37732.17 (31392.79-44889.37)	9.01 (7.85-10.27)	20.39 (16.96-24.25)	2.79 (2.57,3.01)
North Africa and Middle East	1204.30 (890.41-1663.74)	3151.87 (2374.52-4077.11)	0.71 (0.53-0.98)	1.01 (0.76-1.31)	0.90 (0.60,1.21)	1217.72 (889.71-1685.54)	3109.58 (2340.28-4039.49)	0.72 (0.52-0.99)	1.00 (0.75-1.30)	0.80 (0.48,1.11)	40773.85 (30872.19-54909.26)	99272.66 (75659.88-127093.83)	24.04 (18.20-32.38)	31.87 (24.29-40.80)	0.65 (0.35,0.94)
Oceania	40.68 (24.18-82.66)	74.68 (45.69-148.42)	1.24 (0.74-2.52)	1.07 (0.66-2.13)	-0.56 (-0.63,-0.50)	40.55 (24.12-81.81)	73.69 (44.95-147.00)	1.24 (0.74-2.50)	1.06 (0.65-2.11)	-0.60 (-0.67,-0.53)	1443.31 (850.39-2896.96)	2616.87 (1586.33-5193.11)	44.07 (25.97-88.46)	37.58 (22.78-74.57)	-0.58 (-0.66,-0.51)
South Asia	2443.15 (2041.71-2895.03)	7002.12 (5904.19-8399.86)	0.45 (0.37-0.53)	0.76 (0.64-0.91)	1.70 (1.65,1.76)	2462.17 (2059.17-2916.90)	7086.11 (5952.02-8498.05)	0.45 (0.38-0.53)	0.77 (0.64-0.92)	1.70 (1.64,1.76)	85720.05 (73476.41-101610.47)	228264.47 (193229.96-271094.95)	15.68 (13.44-18.59)	24.72 (20.93-29.36)	1.41 (1.37,1.45)
Southeast Asia	4441.27 (3484.27-5437.98)	8850.39 (6432.12-12185.60)	1.91 (1.50-2.34)	2.53 (1.84-3.49)	0.72 (0.64,0.81)	4437.70 (3487.48-5440.46)	8565.75 (6181.78-11772.77)	1.91 (1.50-2.34)	2.45 (1.77-3.37)	0.63 (0.55,0.72)	155077.30 (124195.78-186876.33)	282280.58 (209491.10-382463.62)	66.63 (53.36-80.29)	80.85 (60.00-109.54)	0.44 (0.34,0.54)
Southern Latin America	38.58 (26.66-54.13)	136.36 (94.47-194.79)	0.16 (0.11-0.22)	0.40 (0.28-0.58)	3.54 (3.37,3.71)	39.40 (27.12-55.45)	136.28 (93.87-196.06)	0.16 (0.11-0.22)	0.40 (0.28-0.58)	3.54 (3.29,3.79)	1180.77 (821.73-1646.56)	3744.91 (2663.91-5172.31)	4.77 (3.32-6.65)	11.06 (7.87-15.28)	3.26 (3.00,3.52)
Southern Sub-Saharan Africa	306.54 (172.73-499.22)	830.64 (665.31-1049.16)	1.17 (0.66-1.90)	2.07 (1.66-2.61)	1.02 (0.32,1.73)	303.32 (171.47-493.81)	820.29 (654.70-1036.27)	1.16 (0.65-1.88)	2.04 (1.63-2.58)	1.00 (0.28,1.73)	11617.90 (6654.93-19032.95)	30185.93 (24217.35-38122.52)	44.33 (25.39-72.62)	75.18 (60.31-94.95)	0.90 (0.12,1.68)
Tropical Latin America	178.85 (156.14-205.84)	457.52 (381.80-540.59)	0.23 (0.20-0.27)	0.40 (0.34-0.48)	2.04 (1.86,2.21)	179.65 (156.03-207.12)	462.47 (385.59-547.60)	0.24 (0.20-0.27)	0.41 (0.34-0.48)	2.16 (1.97,2.35)	6410.70 (5626.27-7266.66)	14303.27 (12022.74-16764.83)	8.40 (7.38-9.53)	12.57 (10.57-14.74)	1.70 (1.51,1.89)
Western Europe	1296.96 (984.67-1698.17)	2743.57 (2008.56-3695.80)	0.67 (0.51-0.88)	1.25 (0.92-1.69)	1.99 (1.87,2.11)	1235.70 (925.14-1626.29)	2318.90 (1707.05-3169.03)	0.64 (0.48-0.85)	1.06 (0.78-1.45)	1.57 (1.45,1.68)	34075.40 (25995.19-43887.64)	57077.49 (41556.31-76393.47)	17.73 (13.52-22.83)	26.10 (19.00-34.93)	1.20 (1.10,1.31)
Western Sub-Saharan Africa	2903.01 (1814.58-4288.22)	4446.03 (3400.97-5626.06)	3.01 (1.88-4.44)	1.82 (1.39-2.30)	-1.89 (-2.00,-1.79)	2941.59 (1842.07-4365.77)	4456.13 (3427.81-5672.81)	3.05 (1.91-4.52)	1.82 (1.40-2.32)	-1.91 (-2.02,-1.79)	100607.32 (62791.27-147736.72)	159643.05 (123064.11-203000.81)	104.17 (65.02-152.97)	65.18 (50.25-82.89)	-1.76 (-1.87,-1.64)

UI, uncertainty interval; ASR, age-standardised rate per 100,000; EAPC, estimated annual percentage change; CI, confidence interval; DALYs, disability-adjusted life-year; SDI, socio-demographic index.

From 1990 to 2021, the incidence rate of NASH-related liver cancer increased across all age subgroups except the 40–44 years group, with the most significant increase observed in the 85–89 years population (**Supplementary Table 5**). For NASH-related liver cancer, mortality and DALYs rates decreased in the 30–44 years group but increased in all other age groups, with the most dramatic rise in the population aged 95 years and older (**Supplementary Table 5**). In the 75 years and older age group, incidence, mortality, and DALYs rates of both HBV- and HCV-related liver cancer increased, with the most notable increments in the population aged 95 years and older (**Supplementary Tables 6**, **7**). Regarding alcohol-related liver cancer, incidence decreased in the 15–49 years group but showed an upward trend in the 50 years and older groups, with the most significant increase in the 95 years and older population. Additionally, mortality and DALYs rates increased in the 55 years and older groups, with the most substantial rise in the 95 years and older population (**Supplementary Table 8**). Hepatoblastoma was exclusively observed in the 0–5 years age group, with its incidence, mortality, and DALYs rates all decreasing; the most significant decline was noted in the 5–9 years population (**Supplementary Table 9**). For liver cancer related to other causes, incidence increased in the 50–54 years and 60 years and older groups. In the 75 years and older group, both mortality and DALYs rates rose, with the most remarkable increase in the 95 years and older population (**Supplementary Table 10**). Significant age-related differences were observed globally in 2021. The 65–69 years group had the highest number of incident cases, deaths, and DALYs, indicating high susceptibility in this cohort, with HBV-related liver cancer accounting for the largest proportion across all metrics (**Supplementary Figure 2**).

Except for the low SDI category, the ASRs of incidence, mortality, and DALYs for NASH-related liver cancer showed an upward trend with increasing SDI. High SDI countries had the highest ASRs of incidence, mortality, and DALYs, at 1.01 per 100000 population (95% uncertainty interval [UI], 0.80–1.26), 0.88 per 100000 population (95% UI, 0.68–1.09), and 17.36 per 100000 population (95% UI, 13.93–21.69), respectively. These rates were 1.7- to 2.9-fold higher than those in low-middle SDI countries (**Table 1**; **Supplementary Figure 3**). High-middle SDI countries had the highest ASRs of incidence, mortality, and DALYs for HBV-related liver cancer, with values of 4.25 per 100000 population (95% UI, 3.34–5.37), 3.67 per 100000 population (95% UI, 2.91–4.63), and 114.04 per 100000 population (95% UI, 90.32–144.39), respectively (**Table 2**; **Supplementary Figure 3**). For HCV-related liver cancer, ASRs of incidence, mortality, and DALYs increased with rising SDI. High SDI countries exhibited the highest ASRs: 5.58 per 100000 population (95% UI, 4.74–6.33) for incidence, 4.75 per 100000 population (95% UI, 4.02–5.39) for mortality, and 84.77 per 100000 population (95% UI, 72.97–96.71) for DALYs (**Supplementary Table 1**; **Supplementary Figure 3**). Alcohol-related liver cancer also showed increasing ASRs of incidence, mortality, and DALYs with higher SDI. High SDI countries had the highest rates: 3.17 per 100000 population (95% UI, 2.65–3.74) for incidence, 2.61 per 100000 population (95% UI, 2.17–3.09) for mortality, and 57.51 per 100,000 population (95% UI, 48.08–67.92) for DALYs (**Supplementary Table 2**; **Supplementary Figure 3**). ASRs of incidence, mortality, and DALYs for hepatoblastoma decreased across all SDI categories (**Supplementary Table 3**; **Supplementary Figure 3**). For liver cancer related to other causes, ASRs of incidence, mortality, and DALYs rose with increasing SDI. High SDI countries had the highest rates: 0.58 per 100000 population (95% UI, 0.47–0.70) for incidence, 0.47 per 100000 population (95% UI, 0.38–0.58) for mortality, and 11.11 per 100000 population (95% UI, 9.06–13.42) for DALYs (**Supplementary Table 4**; **Supplementary Figure 3**).

### Regional and national trends

Among the 21 regions, High-income Asia Pacific bore the greatest burden of NASH-related liver cancer, with the highest ASRs of incidence (1.71 per 100000 population; 95% UI, 1.30-2.19), mortality (1.34 per 100000 population; 95% UI, 1.00-1.73), and DALYs (23.26 per 100000 population; 95% UI, 17.77-30.38) (**Table 1**). East Asia had the highest ASRs of incidence (8.30 per 100000 population; 95% UI, 6.51-10.67), mortality (7.02 per 100000 population; 95% UI, 5.49-8.98), and DALYs (220.47 per 100000 population; 95% UI, 172.87-284.77) for HBV-related liver cancer (**Table 2**). For HCV-related liver cancer, High-income Asia Pacific exhibited the highest ASRs: incidence (17.03 per 100000 population; 95% UI, 14.46–19.05), mortality (13.69 per 100000 population; 95% UI, 11.45–15.31), and DALYs (215.50 per 100000 population; 95% UI, 186.75–239.49) (**Supplementary Table 1**). High-income Asia Pacific had the highest ASRs of incidence (3.96 per 100000 population; 95% UI, 3.08-4.97) and mortality (2.98 per 100000 population; 95% UI, 2.35-3.76) rates for alcohol-related liver cancer, whereas Australasia had the highest ASR of DALYs for this subtype (76.06 per 100000 population; 95% UI, 60.70–93.54) (**Supplementary Table 2**). Western Sub-Saharan Africa showed the highest ASRs of incidence (0.17 per 100000 population; 95% UI, 0.12-0.22), mortality (0.12 per 100000 population; 95% UI, 0.08-0.15), and DALYs (10.38 per 100000 population; 95% UI, 7.52-13.72) for hepatoblastoma (**Supplementary Table 3**). For liver cancer related to other causes, High-income Asia Pacific had the highest ASRs of incidence (0.82 per 100000 population; 95% UI, 0.62-1.02) and mortality (0.62 per 100000 population; 95% UI, 0.46-0.77), while East Asia had the highest ASRs of DALYs (16.66 per 100000 population; 95% UI, 12.72-21.73) (**Supplementary Table 4**).

At the national level in 2021, Mongolia bore the greatest burden of NASH-related liver cancer, with ASRs of incidence, mortality, and DALYs being 3.31 (95% UI, 2.06-5.13), 3.53 (95% UI, 2.19-5.49), and 93.67 (95% UI, 59.00-146.19) per 100000 population, respectively. Among these, Taiwan (Province of China) showed the most significant increases in incidence, mortality, and DALYs rates; Zambia had the most notable decrease in incidence; Lithuania exhibited the most substantial reduction in mortality; and Algeria demonstrated the most marked decline in DALYs rate (**Data Sheet 1**; **Supplementary Figure 4**). Taiwan (Province of China) had the highest burden of HBV-related liver cancer, with ASRs of incidence, mortality, and DALYs of 9.00 (95% UI, 7.12-11.21), 8.41 (95% UI, 5.51-12.08), and 96.02 (95% UI, 52.21-171.00) per 100000 population, respectively. Poland showed the most significant increases in incidence, mortality, and DALYs rates for HBV-related liver cancer, while Zambia had the most prominent decreases in these metrics (**Data Sheet 2**; **Supplementary Figure 5**). Monaco carried the heaviest burden of HCV-related liver cancer, with ASRs of incidence, mortality, and DALYs of 8.07 (95% UI, 5.23-11.51), 8.26 (95% UI, 5.45-11.89), and 93.97 (95% UI, 62.45-136.38) per 100000 population, respectively. Taiwan (Province of China) exhibited the most significant increases in incidence, mortality, and DALYs rates for HCV-related liver cancer; Zambia had the most notable decreases in incidence and mortality; and Nepal showed the most marked decline in DALYs rate (**Supplementary Table 11**; **Supplementary Figure 6**). Monaco also had the highest burden of alcohol-related liver cancer, with ASRs of incidence, mortality, and DALYs of 6.95 (95% UI, 4.45-10.76), 6.69 (95% UI, 4.26-10.29), and 99.93 (95% UI, 77.79-124.39) per 100000 population, respectively. Poland showed the most significant increases in incidence, mortality, and DALYs rates for alcohol-related liver cancer; Zambia had the most prominent decrease in incidence; Mexico exhibited the most substantial reduction in mortality; and Malta demonstrated the most marked decline in DALYs rate (**Supplementary Table 12**; **Supplementary Figure 7**). Mali bore the greatest burden of hepatoblastoma, with ASRs of incidence, mortality, and DALYs of 0.70 (95% UI, 0.41-1.05), 0.49 (95% UI, 0.29-0.73), and 9.86 (95% UI, 5.07-16.52) per 100000 population, respectively. Saudi Arabia showed the most significant decreases in incidence, mortality, and DALYs rates for hepatoblastoma (**Supplementary Table 13**; **Supplementary Figure 8**). Mongolia had the highest burden of liver cancer related to other causes, with ASRs of incidence, mortality, and DALYs of 1.45 (95% UI, 0.92-2.24), 1.50 (95% UI, 0.95-2.30), and 9.99 (95% UI, 7.03-13.58) per 100000 population, respectively. Poland exhibited the most significant increases in incidence, mortality, and DALYs rates for this subtype; Zambia had the most notable decrease in incidence; the Netherlands showed the most substantial reduction in mortality; and Guam demonstrated the most marked decline in DALYs rate (**Supplementary Table 14**; **Supplementary Figure 9**).

### Cross-country inequality analysis

Significant absolute and relative SDI-related inequalities were identified, with a disproportionately greater burden borne by countries with higher SDI. The SII showed that the gap in NASH-related liver cancer DALYs between high-SDI and low-SDI countries decreased from 1.32 (95% CI, -0.74–3.38) in 1990 to -0.94 (95% CI, -3.78–1.90) in 2021. For HBV-related liver cancer, the corresponding SII declined from 5.03 (95% CI, -7.61–17.68) to 1.54 (95% CI, -12.35–15.43) over the same period. For HCV-related liver cancer, the SII increased from -0.86 (95% CI, -7.40–5.68) to -1.78 (95% CI, -11.17–7.61). A reversal was observed in the SII for alcohol-related liver cancer, which shifted from 1.36 (95% CI, -6.07–8.79) to -2.46 (95% CI, -14.28–9.36). For hepatoblastoma, the SII increased from 0.19 (95% CI, -2.61–2.99) to 0.24 (95% CI, -0.55–1.02). Regarding liver cancer related to other causes, the SII rose from 0.47 (95% CI, -0.79–1.73) to 0.84 (95% CI, -0.59–2.26). Meanwhile, the concentration index showed a downward trend or relative stability between 1990 and 2021, indicating the persistence of inequalities (**Supplementary Figure 10**).

### Projections up to 2040

Globally, the ASRs of incidence, mortality, and DALYs for NASH-related liver cancer increased by up to 32.09%, 28.52%, and 24.49%, respectively. For HBV-related liver cancer, the ASRs of incidence, mortality, and DALYs rose by up to 19.39%, 1.79%, and 3.30%, respectively. In terms of HCV-related liver cancer, the ASRs of incidence, mortality, and DALYs increased by up to 26.79%, 21.00%, and 14.69%, respectively. For alcohol-related liver cancer, the ASRs of incidence, mortality, and DALYs increased by up to 28.00%, 25.69%, and 20.37%, respectively. Hepatoblastoma showed decreases in ASRs of incidence, mortality, and DALYs by up to 105.88%, 116.13%, and 116.82%, respectively. For liver cancer related to other causes, the ASRs of incidence, mortality, and DALYs increased by up to 21.38%, 14.94%, and 1.13%, respectively (**Supplementary Figures 11**–**16**).

## Discussion

To our knowledge, this observational study is the first to comprehensively analyze the epidemiological trends of disease burdens associated with various etiologies of liver cancer and to reveal the impacts of age, SDI, and regional disparities on liver cancer burden. Despite declining trends in the ASRs of incidence, mortality, and DALYs of hepatoblastoma, the global burden of liver cancer has continued to rise over these 30 years. The ARIMA model predicts that by 2040, the ASRs of incidence, deaths, and DALYs of liver cancer associated with all etiologies except hepatoblastoma will increase.

To contextualize our epidemiological findings within a broader theoretical framework, we integrate the Global Health Transition Model and Social Determinants of Health (SDH) framework, which help explain the observed inequalities and trends. The Global Health Transition Model posits that as societies advance through socioeconomic development (reflected in SDI), the burden of infectious disease-related illnesses (e.g., HBV/HCV-related liver cancer) may shift, but non-communicable disease (NCD)-related subtypes (e.g., NASH-related, alcohol-related liver cancer) emerge as major public health threats—consistent with our observations. High-SDI regions, which have undergone advanced health transitions, exhibit higher burdens of NASH-related and alcohol-related liver cancer, driven by lifestyle shifts (e.g., high-calorie diets, alcohol consumption) and improved healthcare access that extends life expectancy, allowing for the progression of chronic liver conditions. In contrast, low- and middle-SDI regions remain burdened by HBV/HCV-related liver cancer, reflecting persistent challenges in infectious disease control (e.g., limited vaccination coverage, inadequate antiviral access) characteristic of earlier stages of the health transition.

From a global perspective, hepatitis B-related liver cancer remains the most burdensome type currently, with its age-standardized incidence rate, mortality rate, and DALY rate significantly higher than those of other types. This is closely related to the high global prevalence of HBV and the characteristics of chronic HBV infection (1, 27). Notably, the increasing trend of NASH-related liver cancer is the most prominent, with the most significant increase in incidence observed in the 85–89 age group. This may be associated with the global rise in obesity rates, the prevalence of metabolic syndrome, and the intensification of population aging (8). Importantly, societies worldwide—particularly economically developed ones—are experiencing rapid demographic aging, a shift that is reshaping the epidemiological landscape of liver cancer. Many older age groups (e.g., 95 years and older) are now bearing a disproportionately higher burden of subtypes such as NASH-related, HBV-related, and alcohol-related liver cancer compared to previous decades. These age cohorts were either underrepresented or not a major focus in older studies, as their population size and disease prevalence were far less substantial. This aging-related shift underscores the need for updated research frameworks that prioritize these growing high-risk groups, as well as targeted interventions tailored to the unique health needs of elderly populations. In addition, the incidence of alcohol-related liver cancer has increased significantly in the population aged 50 years and older, suggesting that interventions targeting alcohol consumption behaviors in middle-aged and elderly populations need to be strengthened.

The association between SDI levels and liver cancer burden presents a complex pattern. Except in low-SDI regions, the burden of NASH-related liver cancer increases with rising SDI. This may be linked to the westernization of dietary patterns, high obesity rates, and the high prevalence of metabolic diseases in high-income regions (28). The SDH framework further elaborates on these disparities by highlighting how structural factors—including income inequality, educational attainment, healthcare system capacity, and social policies—shape liver cancer outcomes. For instance, high-SDI countries benefit from SDH advantages such as universal health coverage, health literacy programs, and regulations on alcohol marketing, which mitigate some disease risks (e.g., the reversed association between SDI and alcohol-related liver cancer burden). However, these regions also face SDH-driven challenges: socioeconomic privileges enable access to high-calorie diets and sedentary lifestyles, fueling the NASH epidemic. In low- and middle-SDI regions, SDH deficits (e.g., limited access to clean water, poor sanitation, and inadequate healthcare funding) perpetuate HBV/HCV transmission and hinder early detection, amplifying the burden of viral-related liver cancer. Cross-country inequalities in liver cancer burden, as measured by SII and CI, thus reflect underlying SDH inequities that persist despite global health progress. Notably, differences in healthcare systems across regions with varying incomes further contribute to these disparities. Wealthy countries with well-developed healthcare systems—often featuring robust public or state-owned infrastructure—typically have higher rates of early detection, standardized screening programs, and timely access to treatment (e.g., antiviral therapy for HBV/HCV, surgical resection). This enhanced healthcare capacity may lead to more accurate documentation of incident cases and better disease management, which could partially explain the higher reported ASRs of certain subtypes (e.g., NASH-related, HCV-related) in high-SDI regions. In contrast, low- and middle-income regions often face challenges such as limited healthcare funding, inadequate screening coverage, and restricted access to essential medications, resulting in underdiagnosis of asymptomatic cases and delayed treatment initiation. These systemic differences not only affect the accuracy of burden estimates but also exacerbate the actual disease impact, as undiagnosed or untreated liver cancer progresses to advanced stages with higher mortality and DALY rates. Notably, the reliability of liver cancer burden estimates varies substantially across SDI regions, with low-SDI regions exhibiting greater potential biases and wider uncertainty ranges. Quantitatively, the 95% UIs for age-standardized incidence rates of HBV-related liver cancer— the most burdensome subtype in low-SDI regions—are 1.7–2.3 times wider in low-SDI countries (e.g., 0.75–1.33 per 100,000 population) compared to high-SDI countries (e.g., 2.17–3.18 per 100,000 population). This widening UI reflects higher data uncertainty, primarily driven by incomplete cancer registry systems, underreporting of asymptomatic cases, and limited diagnostic capacity in low-resource settings. Based on GBD study methodologies and related literature (10, 29), low-SDI regions may underestimate liver cancer incidence by 15–35% and mortality by 20–40% due to these registry disparities. Such biases could obscure the true burden of viral-related liver cancer in these regions, as many cases are not captured in formal health records. In contrast, the association between SDI and alcohol-related liver cancer has shown a reversal, suggesting that alcohol control measures in high-SDI regions may be gradually taking effect. However, vigilance is needed regarding the increased risk among the population aged 50 years and older. Hepatoblastoma has exhibited a declining trend across all SDI regions, which may be related to improvements in perinatal healthcare and the control of environmental risk factors (29, 30).

Regional and national disparities further reflect the complexity of the liver cancer burden. The High-income Asia Pacific region bears the highest burden of NASH-related, HCV-related, and alcohol-related liver cancer, which is closely linked to lifestyle patterns, medical screening coverage, and viral epidemiological history in this region. East Asia exhibits a significantly higher burden of HBV-related liver cancer than the global average—an outcome shaped by the interplay of biological, lifestyle, and health-system factors. Biologically, the region has a high prevalence of chronic HBV infection, with historical vertical transmission (mother-to-child) and horizontal transmission (in early childhood) contributing to a large reservoir of at-risk individuals (27). Compounding this, lifestyle factors such as high salt intake, heavy alcohol consumption in certain subgroups, and exposure to aflatoxins (in areas with suboptimal food storage) accelerate liver carcinogenesis in HBV-infected populations (4, 29). From a health-system perspective, while East Asian countries (e.g., China, South Korea) have expanded hepatitis B vaccination coverage in recent decades, historical gaps in childhood vaccination mean a large adult population remains unprotected. Additionally, despite advances in antiviral therapy, disparities in healthcare accessibility persist—rural and low-income populations often face barriers to timely diagnosis (e.g., limited access to screening programs) and sustained treatment (e.g., high out-of-pocket costs for antiviral drugs). Even in regions with robust screening policies (e.g., national liver cancer screening programs in China), uneven implementation across urban-rural areas results in late-stage diagnosis for many patients, driving higher mortality and DALY rates. These interconnected factors—biological susceptibility from HBV, lifestyle co-factors, and health-system inequities—collectively explain the persistently high burden of HBV-related liver cancer in East Asia. At the national level, countries and regions such as Mongolia, Taiwan (Province of China), and Monaco bear the heaviest burden of different types of liver cancer, respectively. In contrast, the declining incidence in countries like Zambia may be attributed to improved medical resources and the advancement of prevention and control measures.

Inequality analysis reveals that while high-SDI countries generally have better medical resources, low-SDI countries continue to bear a disproportionate burden of liver cancer. Although the gap in some key indicators such as incidence rate and mortality has narrowed, the concentration index indicates that inequalities persist. This may be related to regional disparities in the distribution of medical resources, health literacy, and prevention and control strategies (29).

Projections based on the ARIMA model indicate that the burden of most types of liver cancer will continue to rise by 2040, with NASH-related liver cancer showing the most significant increase, while hepatoblastoma, a primary liver cancer predominantly affecting children, is the only type projected to decline. This trend underscores the urgency of targeted prevention and control measures: for NASH-related liver cancer, it is necessary to strengthen metabolic disease management and lifestyle interventions (8); for viral-related liver cancer, sustained efforts should be made to promote vaccination and antiviral therapy (27, 31); for alcohol-related liver cancer, it is necessary to strengthen the control of alcohol consumption among middle-aged and elderly populations with long-term drinking habits (29).

To address the growing liver cancer burden, interventions must be grounded in the Global Health Transition Model and SDH framework. For high-SDI regions, priority should be given to SDH-related strategies targeting NASH and alcohol-related liver cancer, such as regulating obesogenic food environments, scaling up metabolic syndrome screening, and strengthening alcohol control policies. For low- and middle-SDI regions, interventions should focus on mitigating infectious disease-related burdens through expanding HBV vaccination coverage, improving antiviral access for HCV, and addressing structural barriers to healthcare (e.g., financial constraints, geographic inaccessibility). Cross-country inequalities, as measured by SII and CI, further emphasize the need for global health initiatives that address SDH inequities—including knowledge transfer, capacity building for cancer registries, and equitable access to novel therapies. Interdisciplinary collaboration should integrate public health (e.g., policy interventions) and clinical care (e.g., personalized screening) while accounting for stage of health transition and local SDH contexts.

### Limitations

Several limitations of this study should be considered when interpreting the results. First, the etiological classification of liver cancer relies on the standardized definitions and modeling framework of the GBD study. However, variations in diagnostic criteria for etiologies, accessibility to diagnostic technologies, and the completeness of reporting across different regions may lead to classification biases. Second, liver cancer registration systems in some countries and regions are inadequate—characterized by low data coverage and delayed updates—particularly in low-SDI areas, where data are primarily estimated through mathematical models, which may not fully reflect the actual disease burden. Specifically, low-SDI regions often exhibit incomplete cancer registry coverage and lack of standardized diagnostic protocols, leading to potential underestimation of incidence and mortality for liver cancer subtypes. This underestimation may be more pronounced for non-viral subtypes (e.g., NASH-related) that require specialized diagnostic tools (e.g., liver biopsy, advanced imaging) rarely available in low-resource settings. Additionally, the GBD framework’s imputation methods, while robust, may not fully capture local variations in underreporting or diagnostic practices, introducing residual biases into cross-regional comparisons. Additionally, while the SDI serves as a comprehensive indicator to reflect regional development levels, it cannot fully encompass nuanced factors such as the distribution of medical resources and cultural differences in health behaviors, potentially masking the heterogeneity of liver cancer burden within individual countries, such as urban-rural disparities or differences across population subgroups. Third, the 2040 projections based on the ARIMA model, while providing a baseline trend estimate, are inherently limited by the model’s reliance on the continuity of historical epidemiological patterns. A key limitation is that the ARIMA model does not explicitly account for potential future shifts in critical influencing factors, such as breakthroughs in medical technology (e.g., the development of more effective antiviral therapies for HBV/HCV, novel treatments for NASH), scaled-up public health interventions (e.g., expanded vaccination coverage, enhanced metabolic syndrome screening, stricter alcohol regulation policies), or widespread lifestyle modifications. These unmodeled factors could substantially alter the trajectory of liver cancer burden—either mitigating or exacerbating the projected trends. The absence of scenario-based projections (e.g., baseline vs. optimistic/pessimistic) means the current forecasts may not capture the full range of potential future outcomes, limiting the robustness of the predictions and their utility for contingency planning. Finally, projections for 2040 based on the ARIMA model depend on the continuity of historical trends, yet the actual disease burden may be influenced by future factors such as prevention and control policies, breakthroughs in medical technology (e.g., new antiviral drugs, improved vaccination coverage), and changes in lifestyle, introducing a certain degree of uncertainty.

## Conclusions

In conclusion, the results of this study indicate that, except for hepatoblastoma, the global disease burden of other types of liver cancer is currently on the rise and projected to continue increasing. Our findings emphasize the urgent need to strengthen interdisciplinary collaboration and integrate public health prevention strategies with clinical diagnosis, treatment, and long-term management interventions to address the increasingly severe challenges posed by liver cancer. Specifically, immediate actions should include: (1) expanding HBV birth-dose vaccination to 95% coverage globally by 2030; (2) integrating metabolic risk screening into routine primary care in high-SDI regions; (3) implementing alcohol taxation and marketing restrictions in middle- and high-SDI regions; (4) ensuring universal access to HCV and HBV antivirals by 2035. These targeted interventions, supported by interdisciplinary collaboration and cross-sector partnerships, will be critical to mitigating the growing liver cancer burden.

## Data Availability

The original contributions presented in the study are included in the article/**Supplementary Material**. Further inquiries can be directed to the corresponding authors.
