# Supplementary material for: Burden of liver cancer from 1990 to 2021 and modelled projection to 2040: insights from the global burden of disease study 2021
Source: Front Oncol. 2026 Apr 29;16:1699519. doi: 10.3389/fonc.2026.1699519 (PMC13167583; doi:10.3389/fonc.2026.1699519)
Supplement: Supplementary file 1 [file DataSheet1.docx]

**Data Sheet 1. Burden of liver cancer due to non-alcoholic steatohepatitis (NASH) in 204 countries and territories in 1990, 2021 and the estimated annual percentage changes (EAPC) from 1990 to 2021**

| **Country** | **Incidence** | | | | |  | **Deaths** | | | | |  | **DALYs** | | | | |
| --- | --- | --- | --- | --- | --- | --- | --- | --- | --- | --- | --- | --- | --- | --- | --- | --- | --- |
|  | **1990 No. (95% UI)** | **2021 No. (95% UI)** | **1990 ASR per 100 000 (95% UI)** | **2021 ASR per 100 000 (95% UI)** | **EAPC (95% CI)** |  | **1990 No. (95% UI)** | **2021 No. (95% UI)** | **1990 ASR per 100 000 (95% UI)** | **2021 ASR per 100 000 (95% UI)** | **EAPC (95% CI)** |  | **1990 No. (95% UI)** | **2021 No. (95% UI)** | **1990 ASR per 100 000 (95% UI)** | **2021 ASR per 100 000 (95% UI)** | **EAPC (95% CI)** |
| Afghanistan | 16.31 (9.03-28.43) | 29.77 (18.30-47.72) | 0.33 (0.18-0.57) | 0.19 (0.12-0.31) | -1.83 (-2.24,-1.41) |  | 17.27 (9.36-30.86) | 30.80 (18.83-49.49) | 0.35 (0.19-0.62) | 0.20 (0.12-0.32) | -1.90 (-2.31,-1.48) |  | 490.65 (282.13-834.08) | 1001.46 (609.46-1600.20) | 9.87 (5.67-16.78) | 6.42 (3.90-10.25) | -1.52 (-1.97,-1.07) |
| Albania | 10.57 (6.58-15.97) | 17.82 (10.37-28.98) | 0.64 (0.40-0.97) | 1.34 (0.78-2.17) | 2.38 (1.88,2.88) |  | 11.65 (7.26-17.48) | 19.91 (11.39-32.43) | 0.70 (0.44-1.06) | 1.49 (0.85-2.43) | 2.38 (1.82,2.94) |  | 277.37 (176.39-411.44) | 403.94 (235.27-654.79) | 16.78 (10.67-24.90) | 30.28 (17.64-49.09) | 1.79 (1.26,2.32) |
| Algeria | 7.21 (4.66-10.87) | 38.33 (24.33-56.46) | 0.06 (0.04-0.09) | 0.17 (0.11-0.26) | 3.71 (3.54,3.88) |  | 7.62 (4.92-11.46) | 40.25 (25.73-59.20) | 0.06 (0.04-0.09) | 0.18 (0.12-0.27) | 3.65 (3.49,3.82) |  | 211.72 (141.21-314.48) | 1030.87 (651.07-1518.38) | 1.67 (1.12-2.49) | 4.66 (2.95-6.87) | 3.45 (3.29,3.61) |
| American Samoa | 0.06 (0.04-0.09) | 0.22 (0.14-0.32) | 0.24 (0.15-0.35) | 0.87 (0.57-1.28) | 4.16 (3.87,4.46) |  | 0.06 (0.04-0.09) | 0.22 (0.15-0.33) | 0.24 (0.16-0.36) | 0.90 (0.59-1.32) | 4.27 (3.94,4.59) |  | 1.78 (1.16-2.66) | 6.14 (4.04-8.90) | 7.35 (4.80-10.96) | 24.66 (16.24-35.77) | 3.99 (3.66,4.31) |
| Andorra | 0.22 (0.13-0.36) | 0.70 (0.41-1.13) | 0.82 (0.48-1.34) | 1.64 (0.96-2.65) | 2.43 (2.29,2.58) |  | 0.22 (0.13-0.37) | 0.69 (0.41-1.11) | 0.82 (0.48-1.35) | 1.61 (0.96-2.59) | 2.42 (2.27,2.58) |  | 5.28 (3.20-8.59) | 14.20 (8.09-22.88) | 19.43 (11.76-31.62) | 33.18 (18.90-53.47) | 1.87 (1.70,2.03) |
| Angola | 15.85 (2.44-46.77) | 36.43 (8.73-96.12) | 0.31 (0.05-0.91) | 0.22 (0.05-0.59) | -1.53 (-1.83,-1.23) |  | 16.33 (2.50-46.98) | 37.76 (9.07-101.54) | 0.32 (0.05-0.91) | 0.23 (0.06-0.62) | -1.48 (-1.77,-1.18) |  | 522.74 (81.94-1579.79) | 1196.56 (281.48-3222.09) | 10.17 (1.59-30.75) | 7.32 (1.72-19.70) | -1.46 (-1.73,-1.18) |
| Antigua and Barbuda | 0.07 (0.05-0.09) | 0.13 (0.09-0.19) | 0.22 (0.15-0.31) | 0.30 (0.20-0.42) | 0.63 (0.00,1.26) |  | 0.07 (0.05-0.10) | 0.14 (0.10-0.20) | 0.25 (0.17-0.35) | 0.32 (0.22-0.45) | 0.48 (-0.12,1.08) |  | 1.62 (1.15-2.19) | 3.31 (2.27-4.59) | 5.39 (3.83-7.28) | 7.40 (5.08-10.27) | 0.67 (0.08,1.27) |
| Argentina | 6.63 (4.48-9.35) | 29.08 (19.56-40.75) | 0.04 (0.03-0.06) | 0.13 (0.09-0.18) | 4.45 (4.13,4.77) |  | 7.19 (4.81-10.26) | 31.40 (20.95-44.38) | 0.04 (0.03-0.06) | 0.14 (0.09-0.20) | 4.46 (4.07,4.87) |  | 170.67 (117.97-238.82) | 711.82 (502.14-983.95) | 1.03 (0.71-1.44) | 3.13 (2.21-4.33) | 4.29 (3.91,4.68) |
| Armenia | 7.86 (5.18-11.51) | 13.00 (8.53-18.77) | 0.46 (0.30-0.67) | 0.87 (0.57-1.25) | 2.45 (1.85,3.06) |  | 8.59 (5.63-12.64) | 14.41 (9.43-21.01) | 0.50 (0.33-0.74) | 0.96 (0.63-1.40) | 2.23 (1.54,2.94) |  | 206.23 (137.44-300.84) | 312.06 (208.03-449.63) | 12.06 (8.04-17.59) | 20.84 (13.89-30.02) | 1.83 (1.21,2.46) |
| Australia | 14.12 (9.76-19.64) | 136.42 (92.42-190.60) | 0.17 (0.12-0.23) | 1.06 (0.72-1.48) | 6.16 (5.93,6.39) |  | 14.60 (9.92-20.49) | 130.17 (89.43-182.29) | 0.17 (0.12-0.24) | 1.01 (0.69-1.41) | 5.72 (5.53,5.91) |  | 350.41 (248.89-477.90) | 2716.04 (1874.10-3767.61) | 4.16 (2.95-5.67) | 21.06 (14.53-29.22) | 5.36 (5.22,5.50) |
| Austria | 9.06 (6.10-13.02) | 31.99 (21.50-45.90) | 0.23 (0.16-0.34) | 0.71 (0.48-1.02) | 3.94 (3.61,4.28) |  | 9.20 (6.08-13.35) | 28.82 (19.14-42.10) | 0.24 (0.16-0.34) | 0.64 (0.43-0.94) | 3.47 (3.20,3.75) |  | 196.93 (135.55-272.06) | 561.59 (385.89-792.63) | 5.07 (3.49-7.00) | 12.50 (8.59-17.65) | 3.11 (2.84,3.37) |
| Azerbaijan | 11.77 (5.98-21.19) | 35.18 (17.89-65.27) | 0.32 (0.16-0.58) | 0.67 (0.34-1.24) | 2.23 (2.06,2.41) |  | 12.61 (6.38-22.68) | 37.36 (18.98-68.28) | 0.34 (0.17-0.62) | 0.71 (0.36-1.30) | 2.28 (2.08,2.47) |  | 337.41 (181.13-597.17) | 978.14 (492.75-1834.66) | 9.21 (4.94-16.30) | 18.63 (9.39-34.95) | 2.04 (1.80,2.28) |
| Bahamas | 0.23 (0.16-0.32) | 0.62 (0.43-0.89) | 0.18 (0.13-0.25) | 0.32 (0.22-0.46) | 1.65 (1.39,1.91) |  | 0.25 (0.17-0.33) | 0.65 (0.45-0.95) | 0.19 (0.13-0.26) | 0.34 (0.23-0.49) | 1.68 (1.38,1.98) |  | 6.65 (4.83-8.83) | 16.96 (11.73-24.52) | 5.18 (3.77-6.88) | 8.74 (6.05-12.64) | 1.53 (1.24,1.82) |
| Bahrain | 0.65 (0.42-0.94) | 2.57 (1.61-3.92) | 0.26 (0.17-0.37) | 0.34 (0.21-0.51) | -0.33 (-0.96,0.31) |  | 0.68 (0.44-0.99) | 2.58 (1.61-3.90) | 0.27 (0.17-0.39) | 0.34 (0.21-0.51) | -0.56 (-1.24,0.12) |  | 18.69 (12.57-26.79) | 70.15 (43.82-109.80) | 7.38 (4.96-10.58) | 9.17 (5.73-14.36) | -0.46 (-1.10,0.19) |
| Bangladesh | 37.01 (24.09-55.94) | 131.19 (80.49-199.46) | 0.07 (0.04-0.10) | 0.16 (0.10-0.24) | 3.10 (2.88,3.32) |  | 38.89 (25.19-58.32) | 139.87 (85.36-215.39) | 0.07 (0.05-0.11) | 0.17 (0.10-0.26) | 3.21 (2.98,3.43) |  | 1160.52 (776.75-1706.34) | 3696.98 (2246.39-5712.44) | 2.13 (1.42-3.13) | 4.49 (2.73-6.94) | 2.81 (2.61,3.01) |
| Barbados | 0.33 (0.22-0.47) | 0.74 (0.47-1.09) | 0.26 (0.18-0.37) | 0.49 (0.32-0.73) | 1.75 (1.57,1.92) |  | 0.37 (0.25-0.53) | 0.82 (0.52-1.20) | 0.30 (0.19-0.42) | 0.55 (0.35-0.81) | 1.65 (1.43,1.88) |  | 7.77 (5.50-10.56) | 17.00 (11.02-24.72) | 6.13 (4.34-8.34) | 11.37 (7.37-16.53) | 1.72 (1.51,1.93) |
| Belarus | 11.54 (7.89-16.37) | 17.63 (11.22-25.62) | 0.22 (0.15-0.31) | 0.38 (0.24-0.55) | 1.41 (1.19,1.63) |  | 12.45 (8.57-17.64) | 18.83 (11.93-27.35) | 0.24 (0.16-0.34) | 0.40 (0.26-0.59) | 1.39 (1.15,1.63) |  | 300.79 (207.31-420.50) | 438.09 (281.80-638.51) | 5.76 (3.97-8.05) | 9.40 (6.04-13.70) | 1.19 (0.95,1.44) |
| Belgium | 13.72 (8.58-20.08) | 32.02 (20.28-45.28) | 0.28 (0.17-0.40) | 0.56 (0.35-0.79) | 2.28 (1.88,2.68) |  | 14.86 (9.17-21.88) | 32.75 (20.55-47.43) | 0.30 (0.18-0.44) | 0.57 (0.36-0.83) | 2.18 (1.66,2.70) |  | 290.76 (189.90-419.00) | 621.64 (398.64-879.91) | 5.83 (3.81-8.40) | 10.84 (6.95-15.34) | 2.09 (1.67,2.52) |
| Belize | 0.08 (0.06-0.11) | 0.38 (0.26-0.52) | 0.08 (0.06-0.12) | 0.17 (0.12-0.24) | 1.64 (1.30,1.97) |  | 0.09 (0.06-0.12) | 0.40 (0.27-0.54) | 0.09 (0.07-0.13) | 0.19 (0.13-0.25) | 1.57 (1.14,2.01) |  | 2.08 (1.49-2.91) | 10.70 (7.44-14.75) | 2.23 (1.59-3.11) | 4.99 (3.47-6.88) | 1.96 (1.53,2.40) |
| Benin | 18.87 (9.35-33.93) | 40.47 (25.83-61.64) | 0.78 (0.39-1.40) | 0.60 (0.38-0.91) | -1.17 (-1.31,-1.03) |  | 20.24 (9.89-36.90) | 43.12 (27.40-65.59) | 0.83 (0.41-1.52) | 0.64 (0.41-0.97) | -1.14 (-1.28,-0.99) |  | 549.83 (279.46-1006.50) | 1209.43 (754.36-1876.84) | 22.67 (11.52-41.50) | 17.92 (11.18-27.81) | -1.07 (-1.23,-0.90) |
| Bermuda | 0.09 (0.06-0.13) | 0.13 (0.08-0.19) | 0.31 (0.22-0.43) | 0.41 (0.26-0.59) | 0.90 (0.39,1.40) |  | 0.10 (0.07-0.14) | 0.14 (0.09-0.20) | 0.34 (0.23-0.46) | 0.43 (0.27-0.64) | 0.72 (0.18,1.26) |  | 2.26 (1.59-3.04) | 2.67 (1.73-3.87) | 7.62 (5.35-10.23) | 8.40 (5.45-12.17) | 0.25 (-0.29,0.80) |
| Bhutan | 0.35 (0.19-0.56) | 1.31 (0.71-2.10) | 0.11 (0.06-0.18) | 0.35 (0.19-0.55) | 3.72 (3.61,3.84) |  | 0.36 (0.19-0.58) | 1.40 (0.77-2.25) | 0.11 (0.06-0.18) | 0.37 (0.20-0.59) | 3.87 (3.74,4.00) |  | 11.21 (6.15-18.13) | 36.28 (19.80-57.33) | 3.56 (1.95-5.76) | 9.59 (5.23-15.15) | 3.22 (3.11,3.34) |
| Bolivia (Plurinational State of) | 4.85 (2.88-7.78) | 18.30 (10.24-30.21) | 0.15 (0.09-0.24) | 0.31 (0.17-0.51) | 2.43 (2.37,2.50) |  | 5.27 (3.17-8.43) | 20.19 (11.28-32.96) | 0.17 (0.10-0.26) | 0.34 (0.19-0.56) | 2.52 (2.45,2.59) |  | 133.51 (79.17-211.01) | 462.51 (264.27-754.14) | 4.18 (2.48-6.61) | 7.84 (4.48-12.79) | 2.10 (2.01,2.19) |
| Bosnia and Herzegovina | 10.94 (7.15-15.66) | 20.53 (12.60-30.92) | 0.49 (0.32-0.70) | 1.24 (0.76-1.87) | 2.90 (2.77,3.03) |  | 11.72 (7.60-16.66) | 22.68 (13.83-33.90) | 0.52 (0.34-0.74) | 1.37 (0.84-2.05) | 2.97 (2.87,3.07) |  | 298.93 (199.15-429.26) | 466.20 (289.00-701.62) | 13.29 (8.85-19.09) | 28.23 (17.50-42.49) | 2.10 (1.97,2.23) |
| Botswana | 1.55 (0.72-3.07) | 5.40 (2.71-10.95) | 0.23 (0.11-0.46) | 0.45 (0.23-0.91) | 1.56 (1.31,1.82) |  | 1.63 (0.77-3.23) | 5.68 (2.84-11.36) | 0.25 (0.12-0.49) | 0.47 (0.24-0.95) | 1.58 (1.30,1.85) |  | 47.34 (21.80-93.27) | 164.91 (79.55-358.08) | 7.18 (3.31-14.14) | 13.78 (6.65-29.92) | 1.57 (1.27,1.87) |
| Brazil | 52.39 (45.68-59.81) | 189.57 (159.85-220.05) | 0.07 (0.06-0.08) | 0.17 (0.15-0.20) | 3.63 (3.30,3.96) |  | 55.73 (48.38-64.10) | 204.58 (172.27-238.26) | 0.08 (0.07-0.09) | 0.19 (0.16-0.22) | 3.76 (3.43,4.09) |  | 1535.07 (1351.28-1734.00) | 4939.93 (4212.44-5692.99) | 2.07 (1.82-2.34) | 4.48 (3.82-5.17) | 3.35 (3.04,3.66) |
| Brunei Darussalam | 0.39 (0.23-0.63) | 1.08 (0.67-1.67) | 0.30 (0.18-0.48) | 0.48 (0.30-0.74) | 1.33 (1.14,1.52) |  | 0.40 (0.24-0.66) | 1.07 (0.66-1.63) | 0.31 (0.18-0.51) | 0.47 (0.29-0.72) | 1.15 (0.93,1.38) |  | 10.41 (6.28-16.92) | 27.29 (17.30-42.21) | 8.03 (4.85-13.06) | 12.10 (7.67-18.71) | 1.13 (0.90,1.36) |
| Bulgaria | 39.10 (25.30-57.36) | 20.58 (12.75-31.63) | 0.90 (0.58-1.32) | 0.61 (0.38-0.93) | -1.12 (-1.54,-0.70) |  | 42.41 (27.59-63.31) | 22.86 (14.15-34.98) | 0.98 (0.64-1.46) | 0.67 (0.42-1.03) | -1.00 (-1.53,-0.47) |  | 1001.69 (655.41-1477.88) | 482.89 (303.51-732.72) | 23.08 (15.10-34.05) | 14.23 (8.94-21.59) | -1.36 (-1.86,-0.85) |
| Burkina Faso | 44.53 (19.01-96.79) | 83.15 (40.11-158.82) | 0.93 (0.40-2.03) | 0.73 (0.35-1.40) | -1.05 (-1.24,-0.86) |  | 47.24 (20.14-101.76) | 88.40 (42.48-172.19) | 0.99 (0.42-2.14) | 0.78 (0.37-1.51) | -1.06 (-1.25,-0.87) |  | 1313.60 (561.41-2814.29) | 2517.84 (1207.97-4878.93) | 27.57 (11.78-59.07) | 22.12 (10.61-42.87) | -0.94 (-1.15,-0.72) |
| Burundi | 6.24 (3.53-10.26) | 8.82 (5.31-14.29) | 0.22 (0.13-0.37) | 0.13 (0.08-0.22) | -2.25 (-2.52,-1.99) |  | 6.57 (3.75-10.89) | 9.22 (5.53-14.87) | 0.24 (0.13-0.39) | 0.14 (0.08-0.22) | -2.26 (-2.53,-2.00) |  | 195.08 (113.59-322.06) | 288.61 (172.33-462.89) | 7.03 (4.09-11.60) | 4.37 (2.61-7.00) | -2.04 (-2.28,-1.79) |
| Cabo Verde | 1.38 (0.81-2.35) | 3.96 (2.36-5.98) | 0.78 (0.46-1.33) | 1.42 (0.84-2.14) | 1.82 (1.57,2.08) |  | 1.55 (0.90-2.65) | 4.39 (2.60-6.65) | 0.88 (0.51-1.50) | 1.57 (0.93-2.38) | 1.77 (1.50,2.05) |  | 34.45 (21.84-56.64) | 100.26 (60.53-148.73) | 19.47 (12.34-32.02) | 35.85 (21.65-53.19) | 1.83 (1.60,2.07) |
| Cambodia | 15.36 (7.16-30.08) | 35.93 (17.87-65.58) | 0.30 (0.14-0.59) | 0.42 (0.21-0.77) | 1.11 (0.95,1.26) |  | 16.09 (7.45-32.07) | 37.70 (18.71-68.72) | 0.31 (0.15-0.62) | 0.44 (0.22-0.81) | 1.11 (0.97,1.26) |  | 471.34 (218.11-922.41) | 1014.36 (500.50-1877.40) | 9.18 (4.25-17.96) | 11.90 (5.87-22.03) | 0.79 (0.66,0.93) |
| Cameroon | 44.82 (27.07-67.44) | 101.66 (51.56-161.77) | 0.86 (0.52-1.29) | 0.64 (0.32-1.02) | -1.37 (-1.59,-1.15) |  | 47.25 (28.36-71.29) | 106.76 (54.35-169.90) | 0.91 (0.54-1.37) | 0.67 (0.34-1.07) | -1.37 (-1.60,-1.14) |  | 1365.06 (848.59-2014.42) | 3137.56 (1622.17-5129.48) | 26.16 (16.26-38.60) | 19.75 (10.21-32.28) | -1.33 (-1.57,-1.10) |
| Canada | 42.27 (29.94-56.77) | 261.23 (183.25-360.05) | 0.31 (0.22-0.42) | 1.39 (0.98-1.92) | 5.22 (5.10,5.34) |  | 42.11 (29.74-57.05) | 251.17 (174.22-349.94) | 0.31 (0.22-0.42) | 1.34 (0.93-1.87) | 5.26 (5.13,5.40) |  | 931.67 (671.86-1241.99) | 4871.24 (3426.84-6631.40) | 6.84 (4.93-9.11) | 26.00 (18.29-35.40) | 4.81 (4.67,4.94) |
| Central African Republic | 4.41 (1.69-9.36) | 5.72 (2.31-12.64) | 0.32 (0.12-0.69) | 0.21 (0.08-0.46) | -1.95 (-2.15,-1.76) |  | 4.54 (1.74-9.70) | 5.86 (2.37-13.01) | 0.33 (0.13-0.71) | 0.21 (0.09-0.47) | -2.00 (-2.21,-1.79) |  | 143.55 (55.71-302.92) | 192.85 (78.75-419.80) | 10.51 (4.08-22.19) | 7.03 (2.87-15.31) | -1.86 (-2.06,-1.66) |
| Chad | 18.46 (7.52-39.69) | 36.04 (19.59-67.98) | 0.61 (0.25-1.32) | 0.41 (0.22-0.77) | -1.74 (-1.89,-1.59) |  | 19.85 (7.98-42.67) | 38.18 (20.60-71.88) | 0.66 (0.26-1.42) | 0.43 (0.23-0.81) | -1.77 (-1.93,-1.61) |  | 528.21 (215.48-1100.10) | 1097.69 (605.24-2065.08) | 17.53 (7.15-36.51) | 12.37 (6.82-23.27) | -1.52 (-1.69,-1.34) |
| Chile | 6.80 (4.53-9.61) | 45.30 (29.49-64.97) | 0.10 (0.07-0.14) | 0.48 (0.31-0.69) | 5.62 (5.32,5.92) |  | 7.36 (4.85-10.56) | 47.85 (31.12-68.36) | 0.11 (0.07-0.16) | 0.51 (0.33-0.73) | 5.60 (5.34,5.85) |  | 171.72 (116.27-241.94) | 1013.11 (677.37-1429.69) | 2.59 (1.75-3.64) | 10.78 (7.21-15.21) | 5.27 (5.02,5.51) |
| China | 2028.43 (1618.75-2488.80) | 5646.35 (4331.54-7156.99) | 0.34 (0.28-0.42) | 0.79 (0.61-1.01) | 3.03 (2.79,3.27) |  | 2064.10 (1646.48-2533.95) | 5204.67 (4018.17-6589.91) | 0.35 (0.28-0.43) | 0.73 (0.56-0.93) | 2.69 (2.47,2.92) |  | 62576.33 (50296.53-76634.31) | 128104.33 (97184.08-163011.63) | 10.64 (8.55-13.03) | 18.01 (13.66-22.92) | 1.89 (1.67,2.11) |
| Colombia | 21.14 (15.01-29.66) | 73.48 (48.82-104.94) | 0.13 (0.09-0.18) | 0.30 (0.20-0.43) | 2.97 (2.36,3.58) |  | 22.83 (15.95-32.17) | 81.25 (53.65-116.65) | 0.14 (0.10-0.20) | 0.33 (0.22-0.48) | 3.06 (2.46,3.66) |  | 587.42 (434.75-804.57) | 1712.93 (1151.83-2400.34) | 3.62 (2.68-4.95) | 6.98 (4.70-9.79) | 2.31 (1.73,2.90) |
| Comoros | 0.77 (0.45-1.20) | 1.83 (1.07-2.76) | 0.33 (0.20-0.52) | 0.49 (0.29-0.74) | 0.90 (0.71,1.09) |  | 0.82 (0.48-1.28) | 1.96 (1.14-3.03) | 0.35 (0.21-0.55) | 0.53 (0.31-0.81) | 0.95 (0.76,1.13) |  | 23.18 (13.81-36.33) | 52.98 (31.66-80.80) | 10.02 (5.97-15.71) | 14.24 (8.51-21.71) | 0.72 (0.46,0.97) |
| Congo | 5.19 (2.08-11.38) | 9.17 (3.92-19.79) | 0.43 (0.17-0.95) | 0.34 (0.15-0.73) | -1.30 (-1.58,-1.02) |  | 5.40 (2.12-11.81) | 9.47 (4.02-20.42) | 0.45 (0.18-0.98) | 0.35 (0.15-0.76) | -1.30 (-1.57,-1.02) |  | 163.22 (66.87-354.75) | 296.97 (126.02-640.75) | 13.59 (5.57-29.54) | 11.02 (4.67-23.77) | -1.17 (-1.45,-0.89) |
| Cook Islands | 0.07 (0.04-0.10) | 0.17 (0.11-0.26) | 0.70 (0.46-1.04) | 1.94 (1.25-2.92) | 3.36 (3.16,3.57) |  | 0.07 (0.05-0.10) | 0.17 (0.11-0.26) | 0.73 (0.49-1.09) | 1.96 (1.25-2.89) | 3.26 (3.06,3.46) |  | 1.89 (1.24-2.80) | 4.18 (2.60-6.28) | 19.96 (13.14-29.62) | 47.13 (29.34-70.76) | 2.89 (2.69,3.09) |
| Costa Rica | 3.48 (2.42-4.76) | 17.73 (11.87-25.00) | 0.23 (0.16-0.31) | 0.75 (0.50-1.05) | 3.72 (3.23,4.20) |  | 3.75 (2.58-5.19) | 19.16 (12.63-27.13) | 0.25 (0.17-0.34) | 0.81 (0.53-1.14) | 3.88 (3.39,4.38) |  | 91.75 (66.16-125.41) | 423.14 (288.77-591.43) | 6.03 (4.35-8.25) | 17.82 (12.16-24.91) | 3.47 (2.98,3.97) |
| Côte d'Ivoire | 11.70 (6.84-18.23) | 24.93 (12.59-40.54) | 0.19 (0.11-0.30) | 0.18 (0.09-0.29) | -0.56 (-0.70,-0.43) |  | 12.15 (7.16-18.96) | 26.07 (13.15-41.66) | 0.20 (0.12-0.31) | 0.19 (0.09-0.30) | -0.49 (-0.62,-0.35) |  | 373.91 (220.64-590.94) | 773.45 (413.80-1226.83) | 6.13 (3.62-9.69) | 5.55 (2.97-8.81) | -0.61 (-0.76,-0.46) |
| Croatia | 8.03 (5.53-11.40) | 16.55 (10.53-24.01) | 0.33 (0.23-0.47) | 0.79 (0.50-1.14) | 3.47 (2.92,4.03) |  | 8.59 (5.87-12.22) | 16.69 (10.52-24.41) | 0.35 (0.24-0.50) | 0.79 (0.50-1.16) | 3.37 (2.82,3.92) |  | 189.48 (131.08-268.83) | 313.11 (197.62-448.03) | 7.80 (5.39-11.06) | 14.88 (9.39-21.29) | 2.76 (2.23,3.30) |
| Cuba | 10.63 (7.46-15.01) | 17.87 (12.29-24.72) | 0.20 (0.14-0.28) | 0.32 (0.22-0.44) | 0.98 (0.57,1.38) |  | 11.62 (7.95-16.37) | 19.25 (13.21-26.32) | 0.21 (0.15-0.30) | 0.34 (0.23-0.47) | 0.90 (0.37,1.43) |  | 265.46 (192.70-365.59) | 422.49 (289.39-590.86) | 4.89 (3.55-6.74) | 7.50 (5.14-10.49) | 0.81 (0.31,1.31) |
| Cyprus | 0.76 (0.47-1.17) | 2.34 (1.39-3.76) | 0.20 (0.12-0.30) | 0.35 (0.21-0.55) | 1.95 (1.88,2.02) |  | 0.82 (0.51-1.28) | 2.36 (1.39-3.83) | 0.21 (0.13-0.33) | 0.35 (0.20-0.56) | 1.66 (1.56,1.76) |  | 17.83 (11.24-26.79) | 46.19 (27.15-72.38) | 4.58 (2.89-6.89) | 6.80 (4.00-10.66) | 1.30 (1.24,1.37) |
| Czechia | 24.11 (16.34-35.09) | 24.00 (15.34-36.20) | 0.47 (0.32-0.68) | 0.45 (0.29-0.68) | -0.24 (-0.45,-0.03) |  | 26.57 (18.10-38.67) | 25.89 (16.55-39.33) | 0.52 (0.35-0.75) | 0.49 (0.31-0.74) | -0.36 (-0.62,-0.10) |  | 586.43 (402.15-845.37) | 505.94 (332.92-763.23) | 11.39 (7.81-16.42) | 9.52 (6.26-14.36) | -0.72 (-0.98,-0.47) |
| Democratic People's Republic of Korea | 43.94 (22.73-78.29) | 72.99 (43.01-118.16) | 0.43 (0.22-0.76) | 0.55 (0.33-0.90) | 0.71 (0.65,0.77) |  | 45.11 (23.59-81.77) | 74.31 (42.98-120.43) | 0.44 (0.23-0.79) | 0.56 (0.33-0.91) | 0.71 (0.65,0.77) |  | 1367.75 (695.68-2412.38) | 2012.08 (1187.64-3296.00) | 13.28 (6.76-23.43) | 15.25 (9.00-24.98) | 0.30 (0.22,0.37) |
| Democratic Republic of the Congo | 27.32 (12.92-55.79) | 53.87 (23.38-128.58) | 0.14 (0.07-0.29) | 0.12 (0.05-0.29) | -0.99 (-1.21,-0.76) |  | 28.38 (13.46-57.97) | 56.36 (24.03-137.58) | 0.15 (0.07-0.30) | 0.13 (0.05-0.31) | -0.98 (-1.19,-0.77) |  | 872.53 (425.94-1808.42) | 1720.25 (763.90-4020.37) | 4.57 (2.23-9.48) | 3.82 (1.70-8.93) | -0.97 (-1.21,-0.72) |
| Denmark | 4.30 (2.80-6.13) | 13.28 (8.28-19.21) | 0.17 (0.11-0.24) | 0.45 (0.28-0.66) | 2.92 (2.66,3.18) |  | 3.69 (2.42-5.26) | 12.29 (7.62-17.94) | 0.14 (0.09-0.20) | 0.42 (0.26-0.61) | 3.32 (3.01,3.63) |  | 78.69 (53.88-108.89) | 237.08 (151.49-339.30) | 3.06 (2.09-4.23) | 8.10 (5.18-11.60) | 3.06 (2.74,3.39) |
| Djibouti | 0.32 (0.19-0.52) | 1.94 (1.15-3.18) | 0.15 (0.09-0.25) | 0.31 (0.18-0.51) | 2.08 (1.94,2.22) |  | 0.33 (0.20-0.53) | 2.02 (1.21-3.36) | 0.16 (0.10-0.25) | 0.32 (0.19-0.53) | 2.18 (2.02,2.35) |  | 10.41 (6.37-17.33) | 60.15 (35.46-98.47) | 5.03 (3.07-8.37) | 9.56 (5.63-15.65) | 1.99 (1.80,2.19) |
| Dominica | 0.07 (0.04-0.12) | 0.16 (0.10-0.25) | 0.20 (0.12-0.32) | 0.49 (0.30-0.73) | 2.54 (2.39,2.69) |  | 0.08 (0.05-0.13) | 0.18 (0.11-0.27) | 0.22 (0.13-0.37) | 0.55 (0.33-0.82) | 2.60 (2.37,2.82) |  | 1.72 (1.05-2.68) | 3.97 (2.45-5.91) | 4.74 (2.89-7.40) | 11.85 (7.30-17.61) | 2.68 (2.49,2.88) |
| Dominican Republic | 1.95 (1.27-2.83) | 8.87 (5.54-13.27) | 0.05 (0.04-0.08) | 0.16 (0.10-0.24) | 3.95 (3.79,4.10) |  | 2.10 (1.38-3.05) | 9.61 (5.91-14.45) | 0.06 (0.04-0.09) | 0.17 (0.11-0.26) | 3.96 (3.81,4.11) |  | 56.59 (37.59-83.04) | 240.07 (153.13-357.58) | 1.58 (1.05-2.32) | 4.36 (2.78-6.49) | 3.68 (3.50,3.86) |
| Ecuador | 11.00 (7.83-14.89) | 32.39 (22.32-46.11) | 0.22 (0.16-0.30) | 0.36 (0.25-0.51) | 1.73 (1.05,2.41) |  | 12.09 (8.48-16.47) | 36.34 (25.07-52.01) | 0.24 (0.17-0.33) | 0.40 (0.28-0.58) | 1.60 (0.88,2.33) |  | 299.01 (217.04-394.15) | 766.45 (528.87-1081.56) | 5.99 (4.35-7.90) | 8.49 (5.86-11.97) | 1.01 (0.29,1.73) |
| Egypt | 149.12 (83.29-276.38) | 694.87 (441.89-1002.39) | 0.54 (0.30-1.00) | 1.32 (0.84-1.90) | 2.88 (2.73,3.03) |  | 155.58 (87.20-290.70) | 713.55 (444.56-1025.92) | 0.56 (0.32-1.05) | 1.35 (0.84-1.94) | 2.74 (2.55,2.94) |  | 4560.80 (2647.10-8309.36) | 20338.84 (13141.16-29135.72) | 16.48 (9.57-30.03) | 38.51 (24.88-55.17) | 2.71 (2.57,2.86) |
| El Salvador | 2.43 (1.66-3.47) | 5.77 (3.75-8.33) | 0.09 (0.06-0.13) | 0.18 (0.12-0.26) | 2.57 (2.16,2.98) |  | 2.63 (1.78-3.79) | 6.27 (4.03-9.10) | 0.10 (0.07-0.14) | 0.19 (0.12-0.28) | 2.45 (2.03,2.87) |  | 68.19 (48.06-93.15) | 148.75 (97.86-211.00) | 2.57 (1.81-3.51) | 4.61 (3.03-6.54) | 2.15 (1.70,2.59) |
| Equatorial Guinea | 0.29 (0.14-0.59) | 1.60 (0.91-2.63) | 0.14 (0.07-0.28) | 0.21 (0.12-0.35) | 1.83 (1.31,2.35) |  | 0.30 (0.14-0.61) | 1.66 (0.95-2.68) | 0.14 (0.07-0.29) | 0.22 (0.13-0.35) | 1.86 (1.34,2.38) |  | 9.01 (4.50-18.34) | 51.28 (28.46-83.14) | 4.26 (2.13-8.67) | 6.78 (3.76-10.99) | 1.88 (1.36,2.40) |
| Eritrea | 2.64 (1.60-4.27) | 6.98 (4.00-11.34) | 0.16 (0.09-0.25) | 0.21 (0.12-0.34) | 0.52 (0.39,0.64) |  | 2.71 (1.64-4.36) | 7.26 (4.18-11.78) | 0.16 (0.10-0.26) | 0.22 (0.13-0.36) | 0.59 (0.45,0.74) |  | 87.68 (53.94-139.68) | 224.05 (129.31-356.96) | 5.15 (3.17-8.20) | 6.79 (3.92-10.82) | 0.42 (0.28,0.57) |
| Estonia | 2.45 (1.66-3.56) | 4.79 (3.16-6.99) | 0.31 (0.21-0.45) | 0.73 (0.48-1.07) | 2.33 (2.04,2.61) |  | 2.66 (1.80-3.84) | 5.28 (3.42-7.77) | 0.34 (0.23-0.49) | 0.81 (0.52-1.19) | 2.50 (2.20,2.81) |  | 63.20 (44.06-91.66) | 102.78 (68.80-147.18) | 8.06 (5.62-11.69) | 15.68 (10.50-22.46) | 1.73 (1.42,2.04) |
| Eswatini | 1.59 (0.69-2.83) | 7.13 (2.86-15.27) | 0.40 (0.17-0.70) | 1.23 (0.49-2.64) | 3.79 (2.69,4.91) |  | 1.68 (0.73-3.03) | 7.38 (2.99-15.79) | 0.42 (0.18-0.75) | 1.28 (0.52-2.73) | 3.76 (2.65,4.89) |  | 49.52 (21.77-86.07) | 231.50 (85.62-515.76) | 12.28 (5.40-21.35) | 40.08 (14.82-89.29) | 4.00 (2.78,5.23) |
| Ethiopia | 39.86 (29.70-55.40) | 68.04 (44.48-109.23) | 0.16 (0.12-0.22) | 0.12 (0.08-0.20) | -1.34 (-1.66,-1.02) |  | 41.49 (30.91-58.27) | 72.37 (47.47-115.36) | 0.16 (0.12-0.23) | 0.13 (0.09-0.21) | -1.24 (-1.56,-0.92) |  | 1279.86 (959.01-1757.87) | 2069.83 (1341.80-3244.12) | 5.06 (3.79-6.95) | 3.80 (2.46-5.96) | -1.54 (-1.85,-1.22) |
| Fiji | 0.68 (0.40-1.10) | 2.08 (1.28-3.24) | 0.18 (0.11-0.29) | 0.45 (0.28-0.70) | 3.32 (3.17,3.47) |  | 0.70 (0.41-1.13) | 2.14 (1.32-3.34) | 0.18 (0.11-0.30) | 0.46 (0.29-0.72) | 3.39 (3.19,3.60) |  | 21.80 (13.21-35.15) | 61.17 (37.07-95.06) | 5.75 (3.48-9.27) | 13.23 (8.02-20.57) | 3.12 (2.91,3.32) |
| Finland | 8.64 (5.50-12.56) | 26.80 (17.27-39.22) | 0.34 (0.22-0.50) | 0.97 (0.62-1.42) | 3.60 (3.52,3.68) |  | 7.92 (4.94-11.66) | 21.67 (13.92-31.73) | 0.32 (0.20-0.47) | 0.78 (0.50-1.15) | 3.15 (2.95,3.35) |  | 167.21 (111.83-237.14) | 388.79 (251.70-543.06) | 6.68 (4.46-9.47) | 14.05 (9.09-19.62) | 2.67 (2.50,2.84) |
| France | 112.25 (75.25-163.05) | 337.54 (215.32-479.25) | 0.39 (0.26-0.56) | 1.02 (0.65-1.44) | 3.00 (2.81,3.19) |  | 119.18 (78.89-172.40) | 319.75 (202.81-462.17) | 0.41 (0.27-0.60) | 0.96 (0.61-1.39) | 2.55 (2.33,2.78) |  | 2564.48 (1710.42-3675.49) | 6071.79 (4003.12-8552.11) | 8.88 (5.92-12.72) | 18.29 (12.06-25.76) | 2.13 (1.90,2.37) |
| Gabon | 2.14 (0.86-4.71) | 5.06 (2.82-8.54) | 0.43 (0.18-0.96) | 0.56 (0.31-0.94) | 0.43 (0.28,0.58) |  | 2.26 (0.91-5.00) | 5.29 (2.95-8.87) | 0.46 (0.18-1.02) | 0.58 (0.32-0.98) | 0.44 (0.28,0.59) |  | 63.07 (25.55-134.89) | 153.26 (84.47-264.17) | 12.82 (5.20-27.43) | 16.88 (9.30-29.09) | 0.57 (0.41,0.73) |
| Gambia | 4.40 (2.85-6.49) | 18.23 (10.43-28.60) | 0.90 (0.58-1.32) | 1.52 (0.87-2.39) | 1.34 (1.13,1.54) |  | 4.60 (2.98-6.88) | 19.16 (10.96-30.08) | 0.94 (0.61-1.40) | 1.60 (0.92-2.51) | 1.44 (1.22,1.65) |  | 137.54 (89.35-203.31) | 560.52 (322.15-892.11) | 28.02 (18.21-41.42) | 46.82 (26.91-74.52) | 1.28 (1.04,1.53) |
| Georgia | 11.10 (7.34-16.07) | 9.59 (6.33-13.85) | 0.40 (0.27-0.58) | 0.53 (0.35-0.77) | 0.52 (-0.55,1.61) |  | 11.86 (7.82-17.14) | 10.54 (6.94-15.26) | 0.43 (0.28-0.62) | 0.58 (0.38-0.85) | 0.58 (-0.54,1.71) |  | 301.79 (203.73-433.61) | 239.45 (159.87-341.82) | 10.93 (7.38-15.70) | 13.28 (8.86-18.95) | 0.14 (-1.03,1.32) |
| Germany | 120.71 (81.67-171.54) | 366.36 (244.73-532.77) | 0.30 (0.20-0.43) | 0.86 (0.57-1.25) | 4.03 (3.70,4.36) |  | 127.82 (85.67-182.93) | 338.01 (223.17-509.56) | 0.32 (0.21-0.46) | 0.79 (0.52-1.19) | 3.65 (3.35,3.94) |  | 2562.33 (1776.15-3580.47) | 6371.36 (4363.16-8888.87) | 6.41 (4.44-8.96) | 14.93 (10.22-20.82) | 3.41 (3.10,3.71) |
| Ghana | 32.66 (18.33-57.34) | 82.19 (45.95-128.06) | 0.44 (0.24-0.77) | 0.48 (0.27-0.75) | -0.39 (-0.75,-0.02) |  | 34.45 (19.14-60.95) | 87.24 (48.25-136.48) | 0.46 (0.26-0.81) | 0.51 (0.28-0.80) | -0.37 (-0.73,-0.02) |  | 1024.77 (580.37-1765.17) | 2468.12 (1439.69-3862.24) | 13.69 (7.75-23.58) | 14.41 (8.41-22.56) | -0.54 (-0.89,-0.18) |
| Greece | 10.65 (7.18-15.19) | 37.74 (25.41-54.59) | 0.20 (0.14-0.29) | 0.74 (0.50-1.07) | 4.25 (4.02,4.48) |  | 11.36 (7.60-16.30) | 40.65 (27.43-58.61) | 0.22 (0.15-0.31) | 0.80 (0.54-1.15) | 4.40 (4.12,4.68) |  | 221.97 (155.95-312.26) | 707.02 (492.05-997.46) | 4.27 (3.00-6.01) | 13.90 (9.67-19.61) | 4.01 (3.71,4.31) |
| Greenland | 0.10 (0.06-0.14) | 0.24 (0.15-0.37) | 0.35 (0.23-0.51) | 0.85 (0.52-1.31) | 2.96 (2.76,3.17) |  | 0.10 (0.07-0.15) | 0.25 (0.15-0.38) | 0.36 (0.24-0.53) | 0.89 (0.54-1.37) | 3.04 (2.80,3.29) |  | 2.94 (2.02-4.38) | 6.09 (3.75-9.30) | 10.60 (7.28-15.77) | 21.70 (13.38-33.14) | 2.47 (2.30,2.64) |
| Grenada | 0.07 (0.04-0.10) | 0.20 (0.14-0.29) | 0.15 (0.10-0.23) | 0.39 (0.27-0.57) | 2.90 (2.29,3.52) |  | 0.08 (0.05-0.12) | 0.22 (0.15-0.32) | 0.17 (0.11-0.27) | 0.43 (0.29-0.62) | 2.79 (2.16,3.42) |  | 1.65 (1.09-2.39) | 5.09 (3.61-7.19) | 3.79 (2.52-5.49) | 9.93 (7.03-14.02) | 3.06 (2.54,3.58) |
| Guam | 0.12 (0.08-0.17) | 0.63 (0.43-0.90) | 0.17 (0.12-0.25) | 0.79 (0.54-1.14) | 5.60 (5.40,5.81) |  | 0.12 (0.08-0.17) | 0.64 (0.44-0.91) | 0.18 (0.12-0.25) | 0.80 (0.55-1.15) | 5.59 (5.36,5.81) |  | 3.48 (2.47-4.89) | 16.97 (11.49-24.50) | 5.08 (3.61-7.15) | 21.32 (14.43-30.78) | 5.42 (5.19,5.66) |
| Guatemala | 9.39 (6.71-12.76) | 27.62 (18.76-39.24) | 0.22 (0.16-0.30) | 0.35 (0.24-0.50) | 1.57 (-0.04,3.19) |  | 10.01 (7.04-13.65) | 30.01 (20.39-42.59) | 0.24 (0.17-0.33) | 0.38 (0.26-0.54) | 1.17 (-0.70,3.08) |  | 279.11 (207.03-369.34) | 753.11 (519.26-1045.02) | 6.66 (4.94-8.81) | 9.55 (6.59-13.26) | 0.79 (-0.95,2.56) |
| Guinea | 38.52 (24.48-56.83) | 62.51 (35.80-93.83) | 1.29 (0.82-1.90) | 0.93 (0.53-1.40) | -1.07 (-1.29,-0.84) |  | 41.12 (26.45-60.40) | 66.26 (38.04-99.11) | 1.37 (0.88-2.02) | 0.99 (0.57-1.48) | -1.06 (-1.31,-0.82) |  | 1125.30 (727.15-1593.07) | 1913.91 (1113.47-2865.18) | 37.55 (24.26-53.16) | 28.50 (16.58-42.67) | -0.89 (-1.06,-0.72) |
| Guinea-Bissau | 5.38 (2.36-9.17) | 7.26 (4.69-11.44) | 1.07 (0.47-1.82) | 0.70 (0.45-1.11) | -1.74 (-1.86,-1.61) |  | 5.61 (2.43-9.62) | 7.55 (4.87-11.71) | 1.11 (0.48-1.91) | 0.73 (0.47-1.13) | -1.69 (-1.83,-1.55) |  | 169.69 (74.39-287.67) | 234.92 (151.33-369.87) | 33.70 (14.77-57.13) | 22.76 (14.66-35.84) | -1.61 (-1.75,-1.47) |
| Guyana | 0.39 (0.26-0.56) | 0.72 (0.46-1.06) | 0.10 (0.07-0.14) | 0.19 (0.12-0.28) | 1.96 (1.72,2.21) |  | 0.41 (0.27-0.59) | 0.76 (0.49-1.11) | 0.11 (0.07-0.15) | 0.20 (0.13-0.29) | 2.08 (1.78,2.38) |  | 11.15 (7.66-15.75) | 20.86 (13.37-29.98) | 2.86 (1.96-4.04) | 5.45 (3.50-7.84) | 2.13 (1.85,2.41) |
| Haiti | 2.09 (1.10-4.26) | 4.12 (2.04-8.33) | 0.07 (0.03-0.13) | 0.06 (0.03-0.13) | -0.10 (-0.18,-0.02) |  | 2.22 (1.15-4.55) | 4.39 (2.16-8.97) | 0.07 (0.04-0.14) | 0.07 (0.03-0.14) | -0.07 (-0.15,0.01) |  | 62.27 (35.45-119.27) | 121.31 (60.69-244.46) | 1.95 (1.11-3.74) | 1.89 (0.94-3.80) | -0.10 (-0.18,-0.02) |
| Honduras | 1.75 (0.94-3.37) | 11.77 (6.88-18.82) | 0.07 (0.04-0.14) | 0.23 (0.14-0.37) | 3.90 (3.73,4.07) |  | 1.88 (1.01-3.67) | 12.76 (7.30-20.25) | 0.08 (0.04-0.16) | 0.25 (0.14-0.40) | 4.02 (3.80,4.24) |  | 51.73 (29.56-93.67) | 314.29 (191.36-495.56) | 2.20 (1.26-3.98) | 6.22 (3.79-9.80) | 3.62 (3.43,3.81) |
| Hungary | 17.46 (11.94-25.39) | 16.00 (10.23-23.54) | 0.34 (0.23-0.49) | 0.33 (0.21-0.49) | -0.55 (-1.25,0.15) |  | 19.27 (13.11-28.03) | 17.53 (11.25-26.01) | 0.37 (0.25-0.54) | 0.37 (0.23-0.54) | -0.51 (-1.19,0.17) |  | 433.75 (300.29-615.80) | 360.53 (232.67-536.66) | 8.35 (5.78-11.85) | 7.51 (4.85-11.18) | -0.78 (-1.40,-0.15) |
| Iceland | 0.26 (0.17-0.39) | 1.03 (0.68-1.48) | 0.21 (0.14-0.31) | 0.59 (0.39-0.85) | 3.21 (2.88,3.54) |  | 0.27 (0.18-0.40) | 0.99 (0.65-1.43) | 0.21 (0.14-0.31) | 0.57 (0.37-0.81) | 2.95 (2.59,3.31) |  | 5.80 (3.96-8.41) | 19.16 (12.88-26.96) | 4.57 (3.12-6.63) | 10.94 (7.35-15.39) | 2.59 (2.24,2.94) |
| India | 558.34 (447.05-668.29) | 2229.24 (1870.29-2669.77) | 0.13 (0.10-0.16) | 0.32 (0.26-0.38) | 2.92 (2.77,3.06) |  | 581.56 (465.96-695.92) | 2367.43 (1984.35-2824.05) | 0.14 (0.11-0.16) | 0.33 (0.28-0.40) | 2.93 (2.77,3.10) |  | 17203.64 (13903.01-20381.96) | 62057.17 (52059.36-73837.84) | 4.03 (3.26-4.78) | 8.77 (7.36-10.44) | 2.48 (2.34,2.62) |
| Indonesia | 152.60 (94.67-224.35) | 528.39 (283.48-821.85) | 0.16 (0.10-0.24) | 0.38 (0.20-0.59) | 2.83 (2.77,2.88) |  | 156.89 (97.49-231.57) | 542.28 (287.99-849.31) | 0.17 (0.11-0.25) | 0.39 (0.21-0.61) | 2.82 (2.76,2.88) |  | 4931.13 (3074.42-7230.42) | 15699.88 (8665.05-24281.75) | 5.33 (3.32-7.82) | 11.26 (6.21-17.41) | 2.55 (2.49,2.61) |
| Iran (Islamic Republic of) | 27.54 (21.39-36.30) | 156.37 (128.70-185.42) | 0.10 (0.07-0.13) | 0.37 (0.30-0.43) | 4.13 (3.46,4.80) |  | 29.29 (22.68-38.78) | 168.75 (138.27-200.68) | 0.10 (0.08-0.14) | 0.40 (0.32-0.47) | 4.16 (3.47,4.85) |  | 794.77 (625.71-1054.48) | 3787.79 (3133.72-4474.83) | 2.78 (2.19-3.69) | 8.88 (7.34-10.49) | 3.47 (2.77,4.17) |
| Iraq | 15.62 (9.84-23.73) | 65.43 (39.95-95.66) | 0.17 (0.11-0.26) | 0.32 (0.19-0.46) | 2.40 (1.88,2.93) |  | 16.57 (10.42-25.12) | 68.11 (41.20-99.73) | 0.18 (0.11-0.27) | 0.33 (0.20-0.48) | 2.33 (1.79,2.88) |  | 456.12 (299.12-673.37) | 1810.27 (1121.80-2631.57) | 4.95 (3.25-7.31) | 8.78 (5.44-12.77) | 2.22 (1.75,2.70) |
| Ireland | 2.71 (1.77-3.93) | 11.22 (7.33-16.07) | 0.15 (0.10-0.22) | 0.45 (0.30-0.65) | 3.99 (3.84,4.14) |  | 2.90 (1.86-4.25) | 10.99 (7.06-15.90) | 0.16 (0.10-0.24) | 0.44 (0.29-0.64) | 3.56 (3.33,3.78) |  | 60.85 (41.06-87.57) | 215.98 (143.21-303.41) | 3.38 (2.28-4.86) | 8.74 (5.80-12.28) | 3.31 (3.13,3.49) |
| Israel | 4.44 (2.90-6.42) | 13.24 (8.77-18.65) | 0.18 (0.12-0.26) | 0.28 (0.18-0.39) | 1.47 (1.32,1.61) |  | 4.82 (3.12-7.06) | 13.67 (8.92-19.63) | 0.19 (0.13-0.28) | 0.28 (0.19-0.41) | 1.24 (1.08,1.40) |  | 100.20 (67.38-142.52) | 272.20 (182.08-382.80) | 4.04 (2.72-5.75) | 5.67 (3.80-7.98) | 1.12 (0.96,1.28) |
| Italy | 131.64 (108.84-156.71) | 168.55 (135.92-203.67) | 0.46 (0.38-0.55) | 0.56 (0.45-0.68) | 0.44 (0.20,0.68) |  | 132.56 (108.98-157.74) | 157.50 (123.87-193.09) | 0.47 (0.38-0.56) | 0.53 (0.41-0.65) | 0.06 (-0.27,0.39) |  | 2896.15 (2403.66-3403.61) | 2889.02 (2367.19-3470.25) | 10.20 (8.46-11.98) | 9.66 (7.92-11.60) | -0.55 (-0.89,-0.21) |
| Jamaica | 0.96 (0.62-1.40) | 2.87 (1.85-4.18) | 0.08 (0.05-0.12) | 0.20 (0.13-0.30) | 3.04 (2.94,3.14) |  | 1.07 (0.69-1.58) | 3.15 (2.03-4.58) | 0.09 (0.06-0.13) | 0.23 (0.15-0.33) | 3.03 (2.70,3.37) |  | 23.08 (15.58-32.98) | 71.40 (47.48-103.00) | 1.95 (1.32-2.79) | 5.10 (3.39-7.36) | 3.17 (2.80,3.54) |
| Japan | 505.09 (431.33-585.64) | 916.86 (693.43-1135.86) | 0.80 (0.69-0.93) | 1.44 (1.09-1.78) | 1.12 (0.54,1.70) |  | 418.71 (357.24-488.69) | 735.09 (552.71-923.99) | 0.67 (0.57-0.78) | 1.15 (0.87-1.45) | 1.20 (0.57,1.83) |  | 10314.12 (8835.47-11934.62) | 11261.09 (8898.04-13680.57) | 16.39 (14.04-18.97) | 17.64 (13.94-21.43) | -0.39 (-1.03,0.25) |
| Jordan | 1.32 (0.76-2.23) | 7.56 (4.52-11.38) | 0.07 (0.04-0.12) | 0.12 (0.07-0.18) | 1.40 (1.23,1.57) |  | 1.38 (0.80-2.36) | 7.75 (4.62-11.68) | 0.07 (0.04-0.13) | 0.13 (0.07-0.19) | 1.35 (1.17,1.54) |  | 38.79 (22.88-64.82) | 203.32 (122.88-302.24) | 2.08 (1.23-3.47) | 3.30 (1.99-4.90) | 1.09 (0.90,1.27) |
| Kazakhstan | 43.41 (29.91-61.32) | 38.40 (25.34-54.81) | 0.53 (0.36-0.75) | 0.41 (0.27-0.58) | -1.62 (-1.93,-1.31) |  | 45.64 (31.76-65.15) | 40.94 (26.83-58.46) | 0.56 (0.39-0.79) | 0.43 (0.28-0.62) | -1.62 (-1.92,-1.31) |  | 1278.27 (886.89-1814.44) | 1045.31 (702.48-1490.31) | 15.60 (10.82-22.14) | 11.03 (7.41-15.72) | -1.99 (-2.31,-1.67) |
| Kenya | 15.95 (10.92-24.33) | 81.54 (59.27-109.29) | 0.14 (0.09-0.21) | 0.33 (0.24-0.44) | 2.91 (2.84,2.98) |  | 16.77 (11.52-25.68) | 84.97 (62.08-113.78) | 0.14 (0.10-0.22) | 0.34 (0.25-0.45) | 2.86 (2.77,2.96) |  | 497.68 (344.44-753.97) | 2537.87 (1845.87-3424.56) | 4.30 (2.98-6.51) | 10.14 (7.37-13.68) | 2.89 (2.81,2.97) |
| Kiribati | 0.12 (0.08-0.18) | 0.25 (0.16-0.36) | 0.32 (0.21-0.49) | 0.41 (0.26-0.59) | 0.52 (0.26,0.79) |  | 0.12 (0.08-0.19) | 0.26 (0.16-0.37) | 0.33 (0.22-0.50) | 0.43 (0.26-0.61) | 0.51 (0.25,0.76) |  | 3.96 (2.57-5.95) | 8.15 (5.14-11.82) | 10.64 (6.90-15.99) | 13.45 (8.48-19.51) | 0.53 (0.27,0.79) |
| Kuwait | 1.33 (0.92-1.82) | 2.49 (1.71-3.42) | 0.15 (0.11-0.21) | 0.11 (0.07-0.15) | -0.78 (-1.20,-0.35) |  | 1.33 (0.92-1.81) | 2.49 (1.68-3.45) | 0.15 (0.11-0.21) | 0.11 (0.07-0.15) | -0.95 (-1.93,0.04) |  | 40.14 (28.56-54.84) | 61.93 (43.20-87.84) | 4.67 (3.32-6.38) | 2.66 (1.86-3.78) | -1.56 (-2.54,-0.56) |
| Kyrgyzstan | 6.20 (4.17-8.88) | 6.03 (3.84-9.08) | 0.28 (0.19-0.40) | 0.18 (0.11-0.26) | -0.94 (-1.89,0.02) |  | 6.58 (4.43-9.38) | 6.38 (4.07-9.56) | 0.29 (0.20-0.42) | 0.19 (0.12-0.28) | -1.33 (-2.38,-0.28) |  | 181.43 (124.36-255.33) | 168.63 (106.41-253.92) | 8.13 (5.57-11.44) | 4.91 (3.10-7.40) | -1.47 (-2.50,-0.43) |
| Lao People's Democratic Republic | 7.44 (4.51-11.93) | 12.47 (7.05-19.51) | 0.36 (0.22-0.57) | 0.34 (0.19-0.53) | -0.38 (-0.55,-0.20) |  | 7.78 (4.74-12.40) | 13.02 (7.31-20.27) | 0.37 (0.23-0.59) | 0.35 (0.20-0.55) | -0.40 (-0.56,-0.23) |  | 228.05 (137.79-368.65) | 369.49 (218.50-579.51) | 10.94 (6.61-17.68) | 10.02 (5.92-15.71) | -0.48 (-0.65,-0.31) |
| Latvia | 3.59 (2.37-5.21) | 5.33 (3.55-7.43) | 0.27 (0.18-0.39) | 0.57 (0.38-0.79) | 1.75 (1.51,1.99) |  | 3.87 (2.54-5.68) | 5.93 (3.95-8.28) | 0.29 (0.19-0.43) | 0.63 (0.42-0.89) | 2.16 (1.86,2.45) |  | 94.09 (62.97-134.19) | 119.62 (81.47-164.22) | 7.08 (4.74-10.10) | 12.79 (8.71-17.56) | 1.50 (1.20,1.79) |
| Lebanon | 2.47 (1.56-3.76) | 6.67 (4.25-9.93) | 0.17 (0.10-0.25) | 0.24 (0.15-0.36) | 1.28 (1.08,1.47) |  | 2.63 (1.65-4.00) | 7.02 (4.45-10.48) | 0.18 (0.11-0.27) | 0.25 (0.16-0.38) | 1.20 (1.00,1.41) |  | 67.80 (43.79-101.75) | 159.56 (104.05-238.07) | 4.53 (2.93-6.80) | 5.76 (3.76-8.59) | 0.75 (0.57,0.93) |
| Lesotho | 2.66 (0.96-5.64) | 10.25 (4.27-22.04) | 0.35 (0.13-0.74) | 1.09 (0.46-2.35) | 3.67 (2.97,4.38) |  | 2.87 (1.02-6.06) | 10.71 (4.48-23.26) | 0.37 (0.13-0.79) | 1.14 (0.48-2.48) | 3.62 (2.93,4.32) |  | 75.67 (27.40-163.36) | 318.81 (130.31-699.22) | 9.88 (3.58-21.32) | 34.02 (13.90-74.61) | 4.13 (3.41,4.85) |
| Liberia | 12.46 (6.51-20.97) | 22.23 (14.19-33.37) | 1.01 (0.53-1.70) | 0.81 (0.52-1.22) | -1.43 (-1.82,-1.04) |  | 13.36 (7.01-22.44) | 23.33 (14.92-35.07) | 1.09 (0.57-1.82) | 0.85 (0.55-1.28) | -1.54 (-1.95,-1.12) |  | 354.20 (182.69-594.57) | 693.26 (435.74-1056.39) | 28.79 (14.85-48.33) | 25.40 (15.96-38.70) | -1.09 (-1.54,-0.64) |
| Libya | 4.98 (2.95-7.71) | 23.85 (15.10-35.75) | 0.24 (0.14-0.37) | 0.69 (0.44-1.04) | 3.91 (3.71,4.10) |  | 5.26 (3.11-8.20) | 24.63 (15.55-36.99) | 0.25 (0.15-0.39) | 0.72 (0.45-1.08) | 3.79 (3.58,4.01) |  | 144.49 (89.24-219.13) | 683.02 (431.37-1017.31) | 6.86 (4.23-10.40) | 19.88 (12.56-29.61) | 3.85 (3.62,4.08) |
| Lithuania | 3.85 (2.61-5.49) | 8.26 (5.60-11.68) | 0.21 (0.14-0.30) | 0.61 (0.41-0.86) | 3.18 (2.81,3.54) |  | 4.11 (2.77-5.84) | 8.70 (5.83-12.21) | 0.22 (0.15-0.32) | 0.64 (0.43-0.90) | 3.42 (3.08,3.76) |  | 95.38 (66.33-132.08) | 180.64 (120.58-258.52) | 5.19 (3.61-7.19) | 13.24 (8.84-18.95) | 2.89 (2.55,3.23) |
| Luxembourg | 0.56 (0.38-0.80) | 1.56 (1.01-2.26) | 0.29 (0.20-0.42) | 0.48 (0.31-0.70) | 1.93 (1.70,2.16) |  | 0.60 (0.40-0.86) | 1.58 (1.01-2.26) | 0.31 (0.21-0.45) | 0.49 (0.31-0.70) | 1.67 (1.41,1.92) |  | 12.63 (8.72-18.00) | 30.32 (20.40-43.58) | 6.63 (4.57-9.44) | 9.41 (6.33-13.53) | 1.32 (1.07,1.56) |
| Madagascar | 10.17 (6.47-15.54) | 21.73 (12.73-35.49) | 0.17 (0.11-0.26) | 0.15 (0.09-0.25) | -0.62 (-0.90,-0.35) |  | 10.62 (6.74-16.14) | 22.46 (13.06-36.67) | 0.18 (0.11-0.27) | 0.16 (0.09-0.26) | -0.66 (-0.93,-0.39) |  | 328.88 (217.42-483.57) | 720.83 (439.72-1165.28) | 5.53 (3.65-8.13) | 5.05 (3.08-8.16) | -0.49 (-0.75,-0.22) |
| Malawi | 8.61 (5.29-12.78) | 24.22 (15.50-34.64) | 0.18 (0.11-0.26) | 0.25 (0.16-0.36) | 0.30 (-0.14,0.74) |  | 9.02 (5.51-13.54) | 25.31 (16.02-36.21) | 0.18 (0.11-0.28) | 0.26 (0.16-0.37) | 0.31 (-0.15,0.77) |  | 270.74 (168.27-400.67) | 764.59 (494.30-1070.89) | 5.52 (3.43-8.17) | 7.86 (5.08-11.01) | 0.37 (-0.08,0.81) |
| Malaysia | 17.09 (11.21-24.96) | 88.48 (58.12-129.67) | 0.19 (0.13-0.28) | 0.56 (0.37-0.82) | 3.71 (3.43,3.99) |  | 18.18 (11.87-26.57) | 91.19 (60.27-134.42) | 0.21 (0.13-0.30) | 0.57 (0.38-0.85) | 3.64 (3.34,3.94) |  | 478.82 (319.28-693.19) | 2293.38 (1510.12-3323.49) | 5.42 (3.61-7.85) | 14.42 (9.49-20.89) | 3.49 (3.20,3.79) |
| Maldives | 0.21 (0.12-0.32) | 0.68 (0.42-1.03) | 0.19 (0.11-0.29) | 0.26 (0.16-0.40) | 1.19 (1.04,1.35) |  | 0.22 (0.13-0.34) | 0.72 (0.44-1.09) | 0.20 (0.11-0.30) | 0.28 (0.17-0.42) | 1.21 (1.04,1.38) |  | 6.07 (3.58-9.50) | 16.83 (10.29-25.94) | 5.46 (3.22-8.54) | 6.51 (3.98-10.03) | 0.48 (0.39,0.57) |
| Mali | 42.81 (27.45-63.59) | 111.59 (72.00-173.56) | 0.99 (0.63-1.47) | 0.93 (0.60-1.44) | -0.20 (-0.35,-0.06) |  | 44.96 (28.83-66.78) | 117.25 (75.60-182.22) | 1.04 (0.67-1.54) | 0.97 (0.63-1.51) | -0.17 (-0.31,-0.04) |  | 1291.48 (837.05-1911.95) | 3410.58 (2238.44-5200.47) | 29.82 (19.33-44.14) | 28.30 (18.57-43.15) | -0.14 (-0.30,0.02) |
| Malta | 0.28 (0.18-0.41) | 1.03 (0.64-1.56) | 0.15 (0.10-0.22) | 0.47 (0.29-0.70) | 3.89 (3.72,4.05) |  | 0.30 (0.19-0.43) | 1.04 (0.64-1.60) | 0.16 (0.10-0.23) | 0.47 (0.29-0.72) | 3.84 (3.65,4.03) |  | 6.40 (4.23-9.22) | 20.44 (12.98-30.43) | 3.45 (2.28-4.98) | 9.24 (5.87-13.76) | 3.52 (3.32,3.72) |
| Marshall Islands | 0.02 (0.01-0.04) | 0.07 (0.04-0.11) | 0.10 (0.05-0.19) | 0.24 (0.13-0.38) | 2.87 (2.79,2.96) |  | 0.02 (0.01-0.05) | 0.07 (0.04-0.11) | 0.11 (0.06-0.20) | 0.24 (0.13-0.40) | 2.74 (2.65,2.82) |  | 0.73 (0.39-1.31) | 2.18 (1.24-3.48) | 3.21 (1.72-5.77) | 7.74 (4.40-12.36) | 2.91 (2.78,3.03) |
| Mauritania | 19.15 (5.42-42.45) | 29.20 (15.83-46.21) | 1.86 (0.53-4.13) | 1.33 (0.72-2.10) | -1.52 (-1.75,-1.29) |  | 20.47 (5.75-44.98) | 31.46 (17.31-49.49) | 1.99 (0.56-4.38) | 1.43 (0.79-2.25) | -1.51 (-1.73,-1.28) |  | 550.37 (151.20-1207.17) | 803.80 (433.85-1216.53) | 53.57 (14.72-117.51) | 36.57 (19.74-55.34) | -1.68 (-1.90,-1.46) |
| Mauritius | 1.48 (1.03-2.04) | 0.83 (0.57-1.17) | 0.27 (0.19-0.37) | 0.13 (0.09-0.18) | 2.08 (0.29,3.90) |  | 1.57 (1.09-2.18) | 0.85 (0.59-1.19) | 0.29 (0.20-0.40) | 0.13 (0.09-0.19) | 3.62 (1.65,5.62) |  | 40.24 (28.25-55.52) | 20.71 (14.24-28.64) | 7.34 (5.15-10.13) | 3.26 (2.24-4.50) | 3.46 (1.51,5.45) |
| Mexico | 32.64 (28.04-37.83) | 179.87 (149.96-212.14) | 0.08 (0.07-0.09) | 0.28 (0.23-0.33) | 4.46 (4.18,4.74) |  | 35.18 (30.09-41.10) | 194.66 (162.06-228.88) | 0.08 (0.07-0.10) | 0.30 (0.25-0.35) | 4.51 (4.22,4.79) |  | 938.14 (817.56-1062.61) | 4681.32 (3885.50-5544.94) | 2.20 (1.91-2.49) | 7.24 (6.01-8.58) | 4.20 (3.92,4.48) |
| Micronesia (Federated States of) | 0.11 (0.07-0.17) | 0.21 (0.12-0.34) | 0.21 (0.13-0.34) | 0.41 (0.24-0.66) | 2.03 (1.82,2.24) |  | 0.11 (0.07-0.18) | 0.21 (0.12-0.34) | 0.22 (0.14-0.35) | 0.42 (0.24-0.67) | 1.96 (1.75,2.18) |  | 3.44 (2.12-5.48) | 6.59 (3.71-10.52) | 6.65 (4.09-10.59) | 12.85 (7.23-20.51) | 2.06 (1.83,2.29) |
| Monaco | 0.11 (0.06-0.18) | 0.35 (0.21-0.54) | 0.71 (0.40-1.17) | 1.85 (1.09-2.83) | 3.36 (2.77,3.95) |  | 0.11 (0.06-0.19) | 0.35 (0.21-0.55) | 0.75 (0.42-1.26) | 1.86 (1.10-2.91) | 3.22 (2.62,3.82) |  | 2.19 (1.27-3.55) | 6.73 (3.97-10.20) | 14.43 (8.35-23.37) | 35.55 (20.98-53.88) | 3.18 (2.61,3.76) |
| Mongolia | 16.22 (9.93-25.44) | 55.17 (34.33-85.57) | 1.50 (0.92-2.36) | 3.31 (2.06-5.13) | 3.06 (2.73,3.39) |  | 17.12 (10.54-26.71) | 58.89 (36.47-91.54) | 1.59 (0.98-2.48) | 3.53 (2.19-5.49) | 3.07 (2.75,3.39) |  | 494.16 (307.14-780.84) | 1562.49 (984.15-2438.65) | 45.80 (28.47-72.37) | 93.67 (59.00-146.19) | 2.65 (2.36,2.93) |
| Montenegro | 1.60 (0.99-2.42) | 3.49 (2.19-5.20) | 0.51 (0.32-0.77) | 1.13 (0.71-1.68) | 2.89 (2.78,3.00) |  | 1.73 (1.08-2.66) | 3.80 (2.38-5.66) | 0.55 (0.34-0.85) | 1.23 (0.77-1.83) | 2.96 (2.85,3.07) |  | 40.45 (25.41-60.75) | 79.86 (50.04-119.57) | 12.92 (8.12-19.40) | 25.84 (16.19-38.69) | 2.51 (2.40,2.61) |
| Morocco | 2.35 (1.43-3.68) | 10.04 (6.21-15.03) | 0.02 (0.01-0.03) | 0.05 (0.03-0.08) | 3.55 (3.30,3.79) |  | 2.49 (1.53-3.87) | 10.58 (6.52-15.72) | 0.02 (0.01-0.03) | 0.06 (0.04-0.08) | 3.53 (3.29,3.77) |  | 68.30 (42.14-103.57) | 277.47 (171.32-417.39) | 0.54 (0.33-0.82) | 1.49 (0.92-2.25) | 3.35 (3.08,3.62) |
| Mozambique | 55.50 (33.41-85.42) | 140.92 (71.01-261.67) | 0.83 (0.50-1.28) | 0.91 (0.46-1.68) | 0.42 (0.32,0.51) |  | 60.09 (36.04-92.20) | 151.88 (77.12-281.76) | 0.90 (0.54-1.38) | 0.98 (0.50-1.81) | 0.40 (0.30,0.49) |  | 1505.38 (906.11-2316.28) | 3901.99 (1981.05-7151.94) | 22.54 (13.56-34.68) | 25.12 (12.75-46.03) | 0.52 (0.42,0.62) |
| Myanmar | 30.19 (11.03-55.59) | 67.01 (29.57-124.13) | 0.15 (0.05-0.27) | 0.24 (0.10-0.44) | 1.44 (1.32,1.56) |  | 31.54 (11.68-58.06) | 70.18 (30.76-130.30) | 0.16 (0.06-0.29) | 0.25 (0.11-0.46) | 1.44 (1.32,1.56) |  | 938.54 (349.67-1751.47) | 1892.57 (831.88-3469.64) | 4.64 (1.73-8.66) | 6.71 (2.95-12.30) | 1.12 (1.00,1.24) |
| Namibia | 0.78 (0.41-1.30) | 2.41 (1.55-3.50) | 0.11 (0.06-0.19) | 0.20 (0.13-0.29) | 1.51 (1.14,1.87) |  | 0.83 (0.43-1.37) | 2.56 (1.66-3.71) | 0.12 (0.06-0.20) | 0.21 (0.14-0.31) | 1.52 (1.14,1.91) |  | 22.74 (12.54-37.89) | 70.35 (44.44-104.23) | 3.24 (1.79-5.40) | 5.79 (3.66-8.57) | 1.53 (1.12,1.93) |
| Nauru | 0.01 (0.01-0.02) | 0.02 (0.01-0.03) | 0.28 (0.17-0.41) | 0.31 (0.18-0.49) | -0.24 (-0.53,0.05) |  | 0.01 (0.01-0.02) | 0.02 (0.01-0.03) | 0.28 (0.17-0.42) | 0.31 (0.18-0.48) | -0.34 (-0.65,-0.02) |  | 0.48 (0.30-0.72) | 0.60 (0.34-0.94) | 9.45 (5.92-14.18) | 10.87 (6.15-16.99) | -0.15 (-0.43,0.12) |
| Nepal | 6.61 (3.85-10.13) | 36.21 (20.54-57.83) | 0.07 (0.04-0.10) | 0.23 (0.13-0.37) | 4.60 (4.13,5.07) |  | 6.91 (4.07-10.68) | 38.46 (21.77-61.72) | 0.07 (0.04-0.11) | 0.25 (0.14-0.40) | 4.67 (4.20,5.14) |  | 208.66 (124.30-319.48) | 1032.79 (593.64-1644.83) | 2.14 (1.28-3.28) | 6.64 (3.81-10.57) | 4.25 (3.79,4.72) |
| Netherlands | 9.77 (6.48-13.29) | 39.33 (26.03-54.74) | 0.13 (0.09-0.18) | 0.46 (0.30-0.64) | 4.42 (4.18,4.67) |  | 10.91 (7.25-14.91) | 43.74 (28.81-62.76) | 0.15 (0.10-0.20) | 0.51 (0.33-0.73) | 4.36 (4.13,4.58) |  | 231.69 (161.94-314.77) | 864.08 (580.79-1204.78) | 3.11 (2.17-4.22) | 10.04 (6.75-14.00) | 4.14 (3.94,4.34) |
| New Zealand | 3.84 (3.23-4.49) | 24.33 (20.07-28.82) | 0.22 (0.19-0.26) | 0.94 (0.78-1.12) | 4.30 (4.04,4.56) |  | 3.31 (2.77-3.89) | 18.28 (15.02-21.82) | 0.19 (0.16-0.23) | 0.71 (0.58-0.84) | 4.07 (3.77,4.38) |  | 82.14 (69.83-95.22) | 406.40 (339.26-477.34) | 4.81 (4.09-5.57) | 15.72 (13.13-18.47) | 3.76 (3.49,4.03) |
| Nicaragua | 1.85 (1.22-2.77) | 7.31 (4.74-10.61) | 0.09 (0.06-0.14) | 0.22 (0.14-0.32) | 3.21 (2.81,3.62) |  | 1.97 (1.29-3.00) | 7.82 (5.05-11.37) | 0.10 (0.07-0.15) | 0.23 (0.15-0.34) | 3.23 (2.80,3.67) |  | 54.17 (37.47-78.68) | 198.48 (129.31-288.71) | 2.79 (1.93-4.05) | 5.95 (3.88-8.66) | 2.97 (2.52,3.42) |
| Niger | 22.90 (10.44-45.57) | 43.52 (24.65-77.54) | 0.57 (0.26-1.13) | 0.35 (0.20-0.62) | -1.88 (-1.99,-1.76) |  | 23.88 (10.98-47.20) | 46.14 (26.07-82.53) | 0.59 (0.27-1.18) | 0.37 (0.21-0.66) | -1.77 (-1.88,-1.65) |  | 722.81 (337.02-1451.19) | 1306.72 (738.78-2298.19) | 18.00 (8.39-36.13) | 10.44 (5.90-18.36) | -2.00 (-2.13,-1.88) |
| Nigeria | 134.18 (63.17-266.15) | 287.59 (189.73-426.63) | 0.30 (0.14-0.59) | 0.25 (0.16-0.37) | -0.99 (-1.20,-0.78) |  | 144.65 (68.58-287.35) | 307.18 (207.67-451.07) | 0.32 (0.15-0.64) | 0.27 (0.18-0.39) | -1.00 (-1.21,-0.78) |  | 3753.22 (1726.64-7487.01) | 8218.15 (5253.73-12424.74) | 8.34 (3.84-16.63) | 7.11 (4.55-10.75) | -0.90 (-1.11,-0.69) |
| Niue | 0.00 (0.00-0.01) | 0.01 (0.00-0.01) | 0.39 (0.23-0.63) | 0.78 (0.46-1.24) | 2.17 (1.74,2.60) |  | 0.00 (0.00-0.01) | 0.01 (0.00-0.01) | 0.43 (0.26-0.68) | 0.82 (0.48-1.29) | 2.07 (1.64,2.50) |  | 0.12 (0.07-0.19) | 0.17 (0.10-0.28) | 10.27 (6.23-16.51) | 20.51 (12.00-33.01) | 2.06 (1.64,2.48) |
| North Macedonia | 7.24 (4.66-10.55) | 12.96 (7.85-20.11) | 0.73 (0.47-1.06) | 1.19 (0.72-1.85) | 1.43 (1.26,1.60) |  | 7.88 (5.08-11.40) | 14.16 (8.47-21.50) | 0.79 (0.51-1.14) | 1.30 (0.78-1.98) | 1.42 (1.25,1.60) |  | 189.97 (125.22-275.97) | 307.26 (186.35-462.19) | 19.07 (12.57-27.70) | 28.24 (17.12-42.47) | 1.01 (0.83,1.19) |
| Northern Mariana Islands | 0.05 (0.04-0.08) | 0.22 (0.14-0.33) | 0.24 (0.16-0.36) | 0.92 (0.57-1.35) | 4.65 (4.18,5.13) |  | 0.05 (0.04-0.08) | 0.22 (0.14-0.32) | 0.24 (0.16-0.36) | 0.91 (0.57-1.32) | 4.74 (4.24,5.25) |  | 1.83 (1.21-2.76) | 6.16 (3.96-9.07) | 8.12 (5.34-12.25) | 25.41 (16.32-37.41) | 4.09 (3.66,4.51) |
| Norway | 3.68 (3.04-4.38) | 13.67 (11.20-16.14) | 0.17 (0.14-0.21) | 0.50 (0.41-0.60) | 3.36 (2.97,3.74) |  | 3.82 (3.15-4.56) | 12.93 (10.53-15.23) | 0.18 (0.15-0.21) | 0.48 (0.39-0.56) | 3.06 (2.59,3.53) |  | 79.14 (66.20-91.99) | 260.80 (216.86-303.40) | 3.73 (3.12-4.33) | 9.63 (8.01-11.20) | 3.04 (2.57,3.50) |
| Oman | 0.80 (0.43-1.43) | 4.67 (3.09-7.06) | 0.08 (0.04-0.14) | 0.20 (0.13-0.30) | 3.37 (3.11,3.63) |  | 0.83 (0.45-1.50) | 4.61 (3.04-6.92) | 0.08 (0.05-0.15) | 0.20 (0.13-0.29) | 3.28 (2.98,3.58) |  | 23.79 (12.94-42.64) | 135.47 (90.61-203.40) | 2.40 (1.30-4.30) | 5.76 (3.85-8.65) | 3.36 (3.03,3.69) |
| Pakistan | 56.71 (43.03-72.45) | 181.61 (134.75-244.33) | 0.10 (0.08-0.13) | 0.15 (0.11-0.21) | 1.23 (1.13,1.33) |  | 59.72 (45.43-76.82) | 187.49 (139.14-250.43) | 0.11 (0.08-0.14) | 0.16 (0.12-0.21) | 1.18 (1.08,1.28) |  | 1831.44 (1392.47-2348.96) | 6230.95 (4626.23-8314.76) | 3.30 (2.51-4.23) | 5.29 (3.93-7.06) | 1.43 (1.27,1.58) |
| Palau | 0.03 (0.02-0.04) | 0.08 (0.05-0.13) | 0.35 (0.21-0.59) | 0.92 (0.51-1.45) | 2.97 (2.71,3.22) |  | 0.03 (0.02-0.05) | 0.08 (0.05-0.13) | 0.36 (0.21-0.62) | 0.92 (0.52-1.47) | 2.87 (2.64,3.10) |  | 0.79 (0.46-1.34) | 2.52 (1.38-4.06) | 10.41 (6.10-17.70) | 27.87 (15.28-44.82) | 3.05 (2.74,3.36) |
| Palestine | 2.76 (1.71-4.26) | 7.50 (4.93-11.08) | 0.27 (0.17-0.42) | 0.29 (0.19-0.43) | 0.03 (-0.16,0.22) |  | 3.01 (1.86-4.65) | 7.89 (5.17-11.80) | 0.29 (0.18-0.45) | 0.31 (0.20-0.46) | -0.11 (-0.30,0.07) |  | 73.22 (44.70-114.21) | 201.77 (135.76-300.41) | 7.15 (4.37-11.16) | 7.86 (5.29-11.70) | 0.02 (-0.19,0.23) |
| Panama | 2.19 (1.50-3.09) | 7.17 (4.70-10.46) | 0.18 (0.13-0.26) | 0.33 (0.22-0.49) | 2.52 (1.84,3.21) |  | 2.40 (1.64-3.38) | 7.86 (5.11-11.50) | 0.20 (0.14-0.28) | 0.37 (0.24-0.54) | 2.44 (1.77,3.12) |  | 56.88 (40.85-76.93) | 173.78 (115.92-247.34) | 4.76 (3.42-6.44) | 8.10 (5.40-11.53) | 2.23 (1.56,2.90) |
| Papua New Guinea | 2.49 (1.04-6.21) | 5.32 (2.25-13.03) | 0.12 (0.05-0.30) | 0.10 (0.04-0.25) | -0.83 (-0.93,-0.74) |  | 2.56 (1.07-6.42) | 5.45 (2.29-13.60) | 0.12 (0.05-0.31) | 0.10 (0.04-0.26) | -0.91 (-1.02,-0.80) |  | 80.27 (34.02-201.36) | 171.94 (73.29-406.84) | 3.91 (1.66-9.81) | 3.29 (1.40-7.78) | -0.81 (-0.90,-0.73) |
| Paraguay | 1.42 (0.95-2.10) | 6.61 (4.15-9.94) | 0.07 (0.05-0.10) | 0.18 (0.12-0.28) | 4.09 (3.71,4.46) |  | 1.54 (1.03-2.28) | 7.13 (4.46-10.64) | 0.08 (0.05-0.11) | 0.20 (0.12-0.30) | 4.13 (3.73,4.53) |  | 39.54 (27.06-58.42) | 176.97 (111.69-265.33) | 1.96 (1.34-2.89) | 4.94 (3.12-7.40) | 3.95 (3.58,4.33) |
| Peru | 9.93 (6.33-14.99) | 45.10 (27.43-66.65) | 0.09 (0.06-0.14) | 0.25 (0.15-0.37) | 3.12 (2.96,3.29) |  | 10.89 (6.86-16.53) | 49.76 (30.01-75.80) | 0.10 (0.06-0.15) | 0.27 (0.17-0.42) | 3.23 (3.01,3.45) |  | 272.04 (182.73-399.97) | 1082.83 (675.18-1602.56) | 2.51 (1.69-3.70) | 5.97 (3.72-8.84) | 2.73 (2.51,2.95) |
| Philippines | 72.72 (49.10-102.84) | 213.56 (170.00-268.98) | 0.23 (0.16-0.33) | 0.38 (0.30-0.48) | 1.54 (1.43,1.64) |  | 75.20 (50.72-106.66) | 221.91 (176.41-279.49) | 0.24 (0.16-0.34) | 0.39 (0.31-0.49) | 1.53 (1.43,1.64) |  | 2345.97 (1594.00-3248.77) | 6227.86 (4908.44-7849.59) | 7.45 (5.06-10.31) | 11.00 (8.67-13.86) | 1.18 (1.08,1.28) |
| Poland | 12.63 (10.69-14.74) | 58.65 (48.65-69.10) | 0.07 (0.06-0.08) | 0.31 (0.25-0.36) | 5.90 (5.22,6.58) |  | 14.31 (12.05-16.73) | 66.23 (55.09-78.19) | 0.07 (0.06-0.09) | 0.35 (0.29-0.41) | 5.97 (5.26,6.68) |  | 300.61 (257.33-349.98) | 1341.93 (1113.23-1581.95) | 1.58 (1.35-1.83) | 7.02 (5.82-8.27) | 5.93 (5.22,6.65) |
| Portugal | 8.16 (5.41-11.66) | 37.02 (24.62-53.90) | 0.16 (0.11-0.23) | 0.70 (0.46-1.02) | 4.51 (4.22,4.80) |  | 8.97 (5.84-12.90) | 39.67 (26.03-57.59) | 0.18 (0.12-0.25) | 0.75 (0.49-1.09) | 4.54 (4.23,4.85) |  | 198.60 (136.78-276.46) | 794.74 (527.86-1136.65) | 3.92 (2.70-5.45) | 14.98 (9.95-21.43) | 4.23 (3.93,4.52) |
| Puerto Rico | 5.25 (3.59-7.28) | 13.33 (8.95-19.09) | 0.29 (0.20-0.40) | 0.81 (0.54-1.16) | 2.67 (2.20,3.15) |  | 5.67 (3.84-7.89) | 14.42 (9.46-20.65) | 0.31 (0.21-0.44) | 0.88 (0.57-1.25) | 2.94 (2.54,3.34) |  | 132.41 (94.81-180.93) | 292.32 (196.16-406.11) | 7.33 (5.25-10.02) | 17.75 (11.91-24.65) | 2.43 (2.02,2.84) |
| Qatar | 0.60 (0.39-0.91) | 8.51 (5.33-13.03) | 0.27 (0.17-0.41) | 0.57 (0.36-0.88) | 1.37 (0.75,2.00) |  | 0.61 (0.39-0.92) | 8.08 (5.05-12.24) | 0.28 (0.18-0.41) | 0.54 (0.34-0.82) | 0.96 (0.33,1.59) |  | 18.73 (12.12-29.00) | 235.32 (144.14-365.46) | 8.42 (5.45-13.04) | 15.81 (9.68-24.55) | 0.95 (0.37,1.53) |
| Republic of Korea | 291.62 (186.55-436.60) | 648.38 (416.95-988.97) | 1.32 (0.84-1.97) | 2.51 (1.62-3.84) | 2.07 (1.98,2.17) |  | 295.10 (185.64-441.55) | 495.16 (313.05-742.70) | 1.33 (0.84-2.00) | 1.92 (1.21-2.88) | 1.01 (0.89,1.13) |  | 7718.73 (5153.44-11336.23) | 10002.50 (6495.35-15154.15) | 34.89 (23.29-51.24) | 38.79 (25.19-58.77) | 0.07 (-0.01,0.15) |
| Republic of Moldova | 3.97 (2.76-5.45) | 6.74 (4.69-9.47) | 0.18 (0.12-0.25) | 0.38 (0.26-0.53) | 2.03 (1.35,2.72) |  | 4.21 (2.95-5.80) | 7.33 (5.08-10.37) | 0.19 (0.13-0.26) | 0.41 (0.28-0.58) | 2.03 (1.28,2.79) |  | 112.14 (80.90-152.79) | 170.30 (119.01-240.09) | 5.04 (3.64-6.87) | 9.48 (6.62-13.36) | 1.57 (0.80,2.34) |
| Romania | 13.51 (8.99-19.21) | 50.00 (33.33-74.07) | 0.12 (0.08-0.16) | 0.53 (0.35-0.78) | 5.51 (5.25,5.77) |  | 14.69 (9.80-21.26) | 54.99 (37.11-81.24) | 0.13 (0.08-0.18) | 0.58 (0.39-0.86) | 5.68 (5.32,6.04) |  | 359.16 (244.43-511.71) | 1161.88 (782.06-1718.09) | 3.07 (2.09-4.38) | 12.27 (8.26-18.14) | 5.14 (4.79,5.49) |
| Russian Federation | 107.03 (91.16-123.62) | 281.82 (238.79-328.61) | 0.14 (0.12-0.16) | 0.39 (0.33-0.45) | 3.93 (3.46,4.41) |  | 114.29 (97.08-132.71) | 306.91 (260.18-358.37) | 0.15 (0.13-0.18) | 0.42 (0.36-0.49) | 4.01 (3.48,4.54) |  | 2964.18 (2547.56-3401.42) | 6933.73 (5862.44-8061.79) | 3.93 (3.37-4.51) | 9.57 (8.09-11.13) | 3.50 (2.99,4.00) |
| Rwanda | 10.55 (6.26-15.84) | 18.28 (10.93-27.65) | 0.29 (0.17-0.44) | 0.28 (0.16-0.42) | -0.97 (-1.46,-0.49) |  | 11.01 (6.62-16.68) | 19.29 (11.63-29.44) | 0.31 (0.18-0.46) | 0.29 (0.18-0.44) | -0.80 (-1.31,-0.28) |  | 341.41 (200.11-513.19) | 557.02 (337.71-836.24) | 9.50 (5.57-14.28) | 8.40 (5.09-12.60) | -1.04 (-1.54,-0.53) |
| Saint Kitts and Nevis | 0.07 (0.05-0.10) | 0.12 (0.08-0.17) | 0.33 (0.22-0.47) | 0.40 (0.27-0.58) | -0.09 (-0.78,0.59) |  | 0.08 (0.05-0.11) | 0.12 (0.08-0.18) | 0.37 (0.25-0.53) | 0.43 (0.28-0.61) | -0.09 (-0.70,0.53) |  | 1.69 (1.20-2.33) | 3.09 (2.04-4.44) | 8.17 (5.78-11.22) | 10.55 (6.96-15.15) | 0.35 (-0.33,1.04) |
| Saint Lucia | 0.08 (0.05-0.11) | 0.17 (0.12-0.25) | 0.11 (0.08-0.16) | 0.19 (0.13-0.28) | 1.10 (0.64,1.55) |  | 0.09 (0.06-0.12) | 0.19 (0.12-0.27) | 0.13 (0.09-0.18) | 0.21 (0.14-0.30) | 1.05 (0.60,1.49) |  | 2.05 (1.44-2.82) | 4.48 (3.08-6.47) | 3.01 (2.10-4.13) | 5.05 (3.47-7.29) | 1.15 (0.69,1.62) |
| Saint Vincent and the Grenadines | 0.10 (0.07-0.14) | 0.17 (0.12-0.24) | 0.18 (0.12-0.25) | 0.30 (0.21-0.43) | 1.62 (1.22,2.03) |  | 0.11 (0.07-0.15) | 0.19 (0.13-0.27) | 0.19 (0.13-0.28) | 0.33 (0.22-0.47) | 1.54 (1.09,1.98) |  | 2.50 (1.76-3.42) | 4.53 (3.10-6.32) | 4.56 (3.21-6.24) | 7.94 (5.43-11.09) | 1.59 (1.14,2.05) |
| Samoa | 0.21 (0.13-0.32) | 0.36 (0.22-0.53) | 0.25 (0.15-0.38) | 0.34 (0.21-0.50) | 0.68 (0.47,0.89) |  | 0.22 (0.13-0.34) | 0.38 (0.23-0.56) | 0.26 (0.16-0.41) | 0.35 (0.22-0.52) | 0.64 (0.41,0.86) |  | 5.88 (3.58-9.25) | 10.03 (6.16-14.86) | 6.96 (4.24-10.95) | 9.39 (5.77-13.91) | 0.64 (0.44,0.83) |
| San Marino | 0.02 (0.01-0.04) | 0.07 (0.04-0.11) | 0.21 (0.12-0.32) | 0.41 (0.22-0.68) | 2.52 (2.40,2.64) |  | 0.03 (0.01-0.04) | 0.07 (0.04-0.11) | 0.21 (0.13-0.33) | 0.41 (0.23-0.68) | 2.45 (2.34,2.57) |  | 0.52 (0.32-0.79) | 1.27 (0.71-2.10) | 4.34 (2.66-6.63) | 7.73 (4.35-12.82) | 2.22 (2.09,2.35) |
| Sao Tome and Principe | 0.09 (0.06-0.13) | 0.16 (0.08-0.26) | 0.14 (0.09-0.21) | 0.15 (0.08-0.24) | -0.09 (-0.21,0.03) |  | 0.09 (0.06-0.14) | 0.17 (0.09-0.28) | 0.15 (0.10-0.23) | 0.16 (0.08-0.26) | -0.17 (-0.32,-0.03) |  | 2.33 (1.57-3.37) | 4.73 (2.46-7.93) | 3.85 (2.58-5.56) | 4.36 (2.27-7.32) | 0.15 (-0.02,0.31) |
| Saudi Arabia | 18.96 (10.02-31.16) | 85.81 (56.45-127.83) | 0.24 (0.13-0.39) | 0.46 (0.30-0.68) | 1.19 (0.85,1.53) |  | 19.96 (10.50-33.04) | 85.85 (56.01-125.98) | 0.25 (0.13-0.42) | 0.46 (0.30-0.67) | 1.06 (0.69,1.42) |  | 558.60 (302.17-928.68) | 2455.09 (1604.95-3656.34) | 7.05 (3.81-11.71) | 13.02 (8.51-19.39) | 1.13 (0.77,1.48) |
| Senegal | 24.22 (15.98-35.95) | 53.81 (34.25-81.17) | 0.63 (0.42-0.94) | 0.68 (0.43-1.02) | -0.04 (-0.18,0.10) |  | 25.79 (17.04-38.42) | 57.90 (36.44-87.33) | 0.68 (0.45-1.01) | 0.73 (0.46-1.10) | -0.05 (-0.21,0.11) |  | 716.13 (476.45-1061.95) | 1521.72 (975.87-2274.12) | 18.77 (12.48-27.83) | 19.19 (12.31-28.68) | -0.24 (-0.41,-0.07) |
| Serbia | 19.41 (11.89-29.85) | 33.17 (20.02-50.92) | 0.40 (0.25-0.62) | 0.74 (0.45-1.14) | 1.64 (1.43,1.85) |  | 20.88 (12.82-31.83) | 36.21 (21.83-55.82) | 0.43 (0.27-0.66) | 0.81 (0.49-1.25) | 1.73 (1.51,1.95) |  | 516.92 (324.26-780.75) | 753.11 (457.59-1147.44) | 10.74 (6.73-16.22) | 16.89 (10.26-25.73) | 1.06 (0.85,1.27) |
| Seychelles | 0.23 (0.15-0.33) | 0.27 (0.18-0.40) | 0.63 (0.42-0.89) | 0.52 (0.34-0.75) | -0.26 (-0.90,0.38) |  | 0.25 (0.17-0.36) | 0.28 (0.18-0.41) | 0.68 (0.46-0.97) | 0.54 (0.35-0.77) | -0.44 (-1.09,0.20) |  | 6.00 (4.07-8.34) | 7.20 (4.73-10.43) | 16.47 (11.17-22.90) | 13.67 (8.98-19.79) | -0.25 (-0.90,0.41) |
| Sierra Leone | 16.09 (5.94-33.41) | 20.69 (12.69-33.40) | 0.78 (0.29-1.61) | 0.47 (0.29-0.75) | -2.16 (-2.37,-1.95) |  | 17.34 (6.36-35.79) | 22.04 (13.38-35.32) | 0.84 (0.31-1.72) | 0.50 (0.30-0.80) | -2.22 (-2.45,-2.00) |  | 456.62 (170.07-939.67) | 617.17 (380.63-1005.91) | 22.00 (8.19-45.27) | 13.92 (8.59-22.69) | -1.93 (-2.13,-1.74) |
| Singapore | 3.99 (2.65-5.96) | 17.26 (11.29-26.22) | 0.26 (0.17-0.39) | 0.60 (0.39-0.92) | 2.58 (2.32,2.83) |  | 3.94 (2.60-5.93) | 13.51 (8.86-20.39) | 0.26 (0.17-0.39) | 0.47 (0.31-0.71) | 1.94 (1.65,2.24) |  | 96.50 (64.55-143.55) | 276.81 (182.87-423.44) | 6.33 (4.24-9.42) | 9.67 (6.39-14.79) | 1.32 (1.02,1.62) |
| Slovakia | 12.00 (7.61-17.57) | 15.79 (9.24-24.47) | 0.45 (0.29-0.67) | 0.58 (0.34-0.90) | 0.42 (0.28,0.56) |  | 13.10 (8.31-19.50) | 17.14 (10.11-26.87) | 0.50 (0.31-0.74) | 0.63 (0.37-0.99) | 0.36 (0.20,0.52) |  | 302.41 (195.91-432.04) | 365.10 (210.55-580.77) | 11.45 (7.42-16.36) | 13.45 (7.76-21.39) | 0.12 (-0.04,0.28) |
| Slovenia | 4.23 (2.90-5.96) | 10.86 (7.05-15.94) | 0.43 (0.29-0.60) | 1.05 (0.68-1.54) | 3.28 (3.06,3.50) |  | 4.59 (3.13-6.46) | 11.70 (7.55-17.19) | 0.47 (0.32-0.66) | 1.13 (0.73-1.66) | 3.03 (2.78,3.29) |  | 104.86 (74.18-145.40) | 218.27 (142.73-326.11) | 10.63 (7.52-14.74) | 21.09 (13.79-31.51) | 2.28 (2.04,2.52) |
| Solomon Islands | 0.31 (0.11-0.72) | 0.73 (0.40-1.24) | 0.18 (0.06-0.43) | 0.21 (0.12-0.36) | 0.40 (0.15,0.65) |  | 0.31 (0.11-0.75) | 0.74 (0.40-1.25) | 0.19 (0.06-0.44) | 0.22 (0.12-0.37) | 0.34 (0.11,0.58) |  | 9.88 (3.33-22.92) | 24.07 (13.22-40.38) | 5.83 (1.96-13.52) | 7.04 (3.87-11.82) | 0.48 (0.27,0.70) |
| Somalia | 12.73 (6.04-23.16) | 30.86 (15.67-58.41) | 0.32 (0.15-0.58) | 0.29 (0.15-0.54) | -0.80 (-0.96,-0.64) |  | 13.09 (6.24-23.77) | 32.07 (16.15-62.08) | 0.33 (0.16-0.60) | 0.30 (0.15-0.57) | -0.71 (-0.87,-0.55) |  | 417.60 (202.38-772.33) | 993.79 (506.78-1906.64) | 10.52 (5.10-19.46) | 9.20 (4.69-17.65) | -0.87 (-1.06,-0.68) |
| South Africa | 53.86 (33.04-86.31) | 209.99 (168.82-254.12) | 0.29 (0.18-0.47) | 0.74 (0.59-0.89) | 2.64 (2.07,3.23) |  | 56.47 (34.46-91.56) | 222.50 (179.53-268.78) | 0.31 (0.19-0.49) | 0.78 (0.63-0.95) | 2.59 (2.01,3.18) |  | 1762.30 (1110.09-2738.32) | 6107.95 (4925.70-7442.08) | 9.52 (6.00-14.80) | 21.49 (17.33-26.18) | 2.17 (1.52,2.83) |
| South Sudan | 8.05 (4.71-12.90) | 17.59 (10.18-27.65) | 0.27 (0.16-0.44) | 0.36 (0.21-0.57) | 0.49 (0.00,0.99) |  | 8.52 (5.03-13.62) | 18.36 (10.57-29.10) | 0.29 (0.17-0.46) | 0.38 (0.22-0.60) | 0.52 (0.03,1.01) |  | 242.60 (141.93-399.07) | 552.70 (323.24-867.86) | 8.26 (4.83-13.58) | 11.43 (6.68-17.94) | 0.75 (0.28,1.24) |
| Spain | 53.92 (35.58-77.55) | 160.82 (104.32-233.60) | 0.28 (0.18-0.40) | 0.71 (0.46-1.03) | 2.63 (2.16,3.10) |  | 57.23 (37.18-83.59) | 156.72 (100.08-233.15) | 0.30 (0.19-0.43) | 0.69 (0.44-1.02) | 2.38 (1.98,2.78) |  | 1264.90 (868.96-1783.52) | 3046.52 (2031.94-4465.61) | 6.52 (4.48-9.20) | 13.38 (8.92-19.61) | 1.98 (1.64,2.32) |
| Sri Lanka | 8.85 (6.04-12.69) | 19.27 (10.91-31.18) | 0.10 (0.07-0.15) | 0.17 (0.10-0.28) | 1.06 (0.69,1.44) |  | 9.44 (6.44-13.53) | 19.70 (11.10-32.17) | 0.11 (0.08-0.16) | 0.18 (0.10-0.29) | 0.99 (0.59,1.39) |  | 246.99 (169.66-353.62) | 469.42 (256.63-767.99) | 2.88 (1.98-4.13) | 4.22 (2.30-6.90) | 0.67 (0.27,1.06) |
| Sudan | 13.73 (6.56-27.15) | 43.45 (25.20-68.21) | 0.14 (0.07-0.27) | 0.20 (0.12-0.31) | 1.34 (0.95,1.74) |  | 14.60 (6.86-29.32) | 46.00 (26.72-72.46) | 0.15 (0.07-0.29) | 0.21 (0.12-0.33) | 1.31 (0.92,1.69) |  | 409.41 (206.63-791.04) | 1286.92 (749.74-2048.81) | 4.09 (2.06-7.90) | 5.93 (3.45-9.44) | 1.34 (0.94,1.75) |
| Suriname | 0.23 (0.15-0.33) | 0.82 (0.49-1.22) | 0.12 (0.08-0.17) | 0.28 (0.17-0.42) | 2.85 (2.60,3.11) |  | 0.25 (0.16-0.36) | 0.88 (0.53-1.31) | 0.13 (0.08-0.18) | 0.30 (0.18-0.45) | 2.67 (2.35,2.99) |  | 6.40 (4.29-9.15) | 22.23 (13.39-33.48) | 3.31 (2.22-4.73) | 7.67 (4.62-11.56) | 2.51 (2.18,2.84) |
| Sweden | 14.51 (11.86-17.12) | 24.42 (19.27-29.78) | 0.34 (0.28-0.40) | 0.47 (0.37-0.57) | 1.59 (0.74,2.44) |  | 16.27 (13.18-19.28) | 26.73 (21.17-32.64) | 0.38 (0.31-0.45) | 0.52 (0.41-0.63) | 1.78 (0.96,2.60) |  | 332.64 (275.89-389.13) | 514.57 (411.10-625.19) | 7.75 (6.43-9.06) | 9.92 (7.93-12.05) | 1.72 (0.87,2.58) |
| Switzerland | 11.92 (8.00-17.19) | 25.87 (16.75-38.00) | 0.35 (0.23-0.50) | 0.58 (0.38-0.85) | 1.79 (1.64,1.94) |  | 11.93 (7.97-17.29) | 24.58 (15.59-36.87) | 0.35 (0.23-0.50) | 0.55 (0.35-0.83) | 1.71 (1.61,1.82) |  | 254.98 (173.61-357.45) | 461.72 (302.96-669.21) | 7.43 (5.06-10.41) | 10.35 (6.79-15.00) | 1.30 (1.18,1.42) |
| Syrian Arab Republic | 14.90 (8.91-24.84) | 37.44 (22.88-56.09) | 0.23 (0.14-0.39) | 0.53 (0.33-0.80) | 2.34 (1.49,3.19) |  | 15.88 (9.38-26.45) | 39.01 (23.81-58.28) | 0.25 (0.15-0.42) | 0.56 (0.34-0.83) | 2.26 (1.38,3.15) |  | 438.64 (276.34-719.18) | 995.63 (610.52-1524.64) | 6.90 (4.35-11.31) | 14.19 (8.70-21.73) | 1.95 (1.09,2.82) |
| Taiwan (Province of China) | 37.55 (26.85-51.62) | 209.89 (143.75-293.14) | 0.37 (0.26-0.51) | 1.78 (1.22-2.48) | 6.40 (5.53,7.28) |  | 36.97 (26.53-50.57) | 189.00 (128.73-265.72) | 0.36 (0.26-0.50) | 1.60 (1.09-2.25) | 6.18 (5.24,7.12) |  | 1119.92 (817.43-1535.73) | 4285.11 (2934.21-5980.79) | 10.98 (8.02-15.06) | 36.26 (24.83-50.61) | 5.15 (4.24,6.06) |
| Tajikistan | 4.37 (2.47-7.46) | 8.13 (4.41-13.57) | 0.16 (0.09-0.28) | 0.16 (0.09-0.27) | -0.23 (-0.40,-0.05) |  | 4.69 (2.59-8.30) | 8.55 (4.67-14.26) | 0.17 (0.10-0.31) | 0.17 (0.09-0.28) | -0.26 (-0.45,-0.08) |  | 131.96 (82.98-206.70) | 247.88 (136.79-408.72) | 4.92 (3.09-7.70) | 4.88 (2.69-8.05) | -0.31 (-0.53,-0.08) |
| Thailand | 205.87 (132.32-306.03) | 607.44 (389.98-900.63) | 0.73 (0.47-1.08) | 1.82 (1.17-2.70) | 2.61 (2.35,2.88) |  | 215.19 (138.44-320.58) | 621.29 (401.61-931.43) | 0.76 (0.49-1.13) | 1.86 (1.20-2.79) | 2.56 (2.33,2.79) |  | 5996.19 (3966.95-8777.72) | 14752.92 (9267.62-22648.07) | 21.13 (13.98-30.93) | 44.25 (27.80-67.93) | 1.99 (1.70,2.27) |
| Timor-Leste | 0.40 (0.23-0.62) | 1.18 (0.65-1.95) | 0.10 (0.06-0.16) | 0.17 (0.09-0.28) | 1.88 (1.76,2.00) |  | 0.41 (0.24-0.64) | 1.25 (0.69-2.10) | 0.11 (0.06-0.16) | 0.18 (0.10-0.30) | 1.99 (1.85,2.13) |  | 12.71 (7.34-20.15) | 32.45 (18.34-52.81) | 3.25 (1.88-5.16) | 4.64 (2.62-7.56) | 1.33 (1.16,1.51) |
| Togo | 5.84 (3.80-8.66) | 19.61 (11.76-30.42) | 0.32 (0.21-0.48) | 0.47 (0.28-0.73) | 0.86 (0.74,0.97) |  | 6.14 (3.93-9.26) | 20.66 (12.26-32.41) | 0.34 (0.22-0.51) | 0.49 (0.29-0.77) | 0.89 (0.76,1.02) |  | 182.94 (120.85-268.43) | 590.84 (362.01-906.37) | 10.03 (6.63-14.72) | 14.12 (8.65-21.66) | 0.74 (0.61,0.87) |
| Tokelau | 0.00 (0.00-0.00) | 0.00 (0.00-0.01) | 0.30 (0.16-0.57) | 0.53 (0.29-0.90) | 1.63 (1.41,1.85) |  | 0.00 (0.00-0.01) | 0.00 (0.00-0.01) | 0.32 (0.17-0.63) | 0.55 (0.30-0.96) | 1.49 (1.26,1.72) |  | 0.07 (0.04-0.12) | 0.10 (0.05-0.17) | 8.25 (4.40-15.36) | 14.31 (7.81-24.18) | 1.68 (1.47,1.90) |
| Tonga | 0.53 (0.31-0.88) | 0.93 (0.56-1.39) | 1.08 (0.62-1.78) | 1.75 (1.05-2.62) | 1.03 (0.55,1.52) |  | 0.56 (0.31-0.92) | 0.97 (0.59-1.44) | 1.12 (0.64-1.86) | 1.83 (1.11-2.72) | 1.07 (0.58,1.57) |  | 15.68 (9.30-26.03) | 25.79 (15.62-38.70) | 31.72 (18.83-52.67) | 48.53 (29.39-72.81) | 0.90 (0.42,1.37) |
| Trinidad and Tobago | 0.95 (0.65-1.33) | 2.50 (1.62-3.65) | 0.16 (0.11-0.22) | 0.36 (0.23-0.52) | 2.49 (2.18,2.80) |  | 1.03 (0.70-1.45) | 2.69 (1.72-3.91) | 0.17 (0.12-0.24) | 0.39 (0.25-0.56) | 2.36 (2.00,2.72) |  | 25.25 (18.03-34.93) | 65.41 (43.24-95.01) | 4.19 (2.99-5.80) | 9.39 (6.21-13.64) | 2.37 (2.04,2.71) |
| Tunisia | 2.57 (1.62-4.00) | 11.11 (6.51-18.07) | 0.06 (0.04-0.10) | 0.19 (0.11-0.31) | 3.73 (3.53,3.93) |  | 2.73 (1.70-4.29) | 11.62 (6.64-18.90) | 0.07 (0.04-0.10) | 0.20 (0.11-0.32) | 3.67 (3.49,3.86) |  | 71.84 (46.50-111.02) | 284.14 (168.31-468.61) | 1.72 (1.11-2.66) | 4.80 (2.84-7.91) | 3.45 (3.26,3.65) |
| Turkey | 39.22 (25.36-59.95) | 150.56 (95.37-219.00) | 0.14 (0.09-0.21) | 0.36 (0.23-0.52) | 3.50 (3.03,3.97) |  | 42.26 (27.16-65.05) | 161.21 (102.32-232.96) | 0.15 (0.09-0.23) | 0.39 (0.24-0.56) | 3.49 (3.00,3.98) |  | 1079.68 (710.23-1591.57) | 3605.56 (2311.20-5309.98) | 3.76 (2.47-5.54) | 8.62 (5.53-12.70) | 2.90 (2.42,3.38) |
| Turkmenistan | 3.26 (2.30-4.66) | 9.82 (6.37-14.61) | 0.18 (0.12-0.25) | 0.38 (0.25-0.57) | 2.99 (2.78,3.21) |  | 3.43 (2.42-4.87) | 10.25 (6.63-15.34) | 0.19 (0.13-0.26) | 0.40 (0.26-0.59) | 2.87 (2.50,3.23) |  | 100.61 (73.01-138.93) | 295.19 (192.54-439.86) | 5.44 (3.95-7.51) | 11.44 (7.47-17.05) | 2.92 (2.57,3.26) |
| Tuvalu | 0.01 (0.01-0.02) | 0.03 (0.02-0.04) | 0.27 (0.16-0.48) | 0.41 (0.25-0.66) | 1.02 (0.83,1.22) |  | 0.01 (0.01-0.02) | 0.03 (0.02-0.04) | 0.29 (0.16-0.50) | 0.43 (0.25-0.70) | 1.03 (0.82,1.24) |  | 0.39 (0.23-0.69) | 0.73 (0.44-1.16) | 8.27 (4.86-14.47) | 11.83 (7.14-18.83) | 0.96 (0.78,1.13) |
| Uganda | 25.92 (15.83-38.58) | 65.48 (40.78-99.93) | 0.30 (0.18-0.45) | 0.30 (0.19-0.46) | -0.59 (-0.78,-0.39) |  | 27.43 (16.85-40.68) | 68.67 (42.36-105.27) | 0.32 (0.19-0.47) | 0.32 (0.20-0.49) | -0.58 (-0.78,-0.38) |  | 778.64 (481.01-1146.30) | 2098.61 (1288.34-3231.99) | 9.01 (5.56-13.26) | 9.69 (5.95-14.92) | -0.39 (-0.62,-0.15) |
| Ukraine | 49.45 (39.05-61.08) | 43.14 (30.15-57.32) | 0.19 (0.15-0.23) | 0.20 (0.14-0.27) | -0.74 (-1.32,-0.17) |  | 51.78 (40.95-64.35) | 44.94 (31.56-59.74) | 0.20 (0.16-0.24) | 0.21 (0.15-0.28) | -0.49 (-1.11,0.14) |  | 1332.46 (1068.36-1633.53) | 1100.67 (757.81-1451.00) | 5.06 (4.05-6.20) | 5.11 (3.52-6.74) | -0.72 (-1.36,-0.08) |
| United Arab Emirates | 2.03 (1.29-3.13) | 29.77 (17.82-47.33) | 0.22 (0.14-0.33) | 0.62 (0.37-0.98) | 2.44 (1.85,3.04) |  | 2.07 (1.30-3.20) | 29.06 (17.54-45.76) | 0.22 (0.14-0.34) | 0.60 (0.36-0.95) | 2.28 (1.70,2.86) |  | 64.95 (41.29-98.46) | 946.42 (578.05-1490.88) | 6.94 (4.41-10.53) | 19.65 (12.00-30.96) | 2.58 (2.08,3.08) |
| United Kingdom | 62.67 (51.64-74.57) | 317.88 (261.14-377.92) | 0.22 (0.18-0.26) | 0.94 (0.77-1.11) | 5.60 (5.38,5.81) |  | 62.30 (50.86-74.63) | 290.79 (236.88-349.63) | 0.22 (0.18-0.26) | 0.86 (0.70-1.03) | 5.16 (4.92,5.40) |  | 1296.04 (1081.19-1535.69) | 5475.63 (4549.17-6549.88) | 4.52 (3.77-5.36) | 16.14 (13.41-19.31) | 4.87 (4.63,5.11) |
| United Republic of Tanzania | 38.24 (25.07-58.98) | 84.82 (53.68-129.59) | 0.30 (0.19-0.46) | 0.29 (0.18-0.44) | -0.40 (-0.57,-0.24) |  | 40.27 (26.44-61.97) | 89.66 (55.89-137.12) | 0.31 (0.20-0.48) | 0.31 (0.19-0.47) | -0.39 (-0.56,-0.22) |  | 1167.04 (780.22-1746.68) | 2591.72 (1615.83-4016.82) | 9.03 (6.04-13.52) | 8.87 (5.53-13.74) | -0.41 (-0.58,-0.23) |
| United States of America | 358.53 (306.25-415.23) | 1690.74 (1427.47-1979.00) | 0.28 (0.24-0.33) | 1.02 (0.86-1.19) | 4.22 (4.10,4.34) |  | 329.98 (280.61-385.35) | 1399.36 (1170.51-1634.93) | 0.26 (0.22-0.30) | 0.84 (0.70-0.98) | 3.88 (3.79,3.97) |  | 7030.20 (6093.73-8018.72) | 29832.76 (25222.85-35024.54) | 5.53 (4.80-6.31) | 17.94 (15.17-21.06) | 4.04 (3.93,4.14) |
| United States Virgin Islands | 0.08 (0.05-0.12) | 0.28 (0.17-0.43) | 0.15 (0.09-0.23) | 0.65 (0.39-1.01) | 4.86 (4.63,5.09) |  | 0.08 (0.05-0.13) | 0.30 (0.18-0.48) | 0.16 (0.10-0.24) | 0.70 (0.43-1.11) | 4.93 (4.71,5.16) |  | 2.08 (1.27-3.14) | 6.59 (3.97-10.49) | 3.92 (2.40-5.92) | 15.34 (9.24-24.42) | 4.49 (4.26,4.72) |
| Uruguay | 1.46 (0.95-2.12) | 5.98 (3.89-8.52) | 0.09 (0.06-0.14) | 0.35 (0.23-0.50) | 5.08 (4.80,5.37) |  | 1.58 (1.03-2.30) | 6.44 (4.19-9.12) | 0.10 (0.07-0.15) | 0.38 (0.25-0.54) | 5.08 (4.75,5.42) |  | 35.86 (24.04-51.16) | 138.44 (91.50-197.05) | 2.28 (1.53-3.26) | 8.13 (5.37-11.57) | 4.87 (4.55,5.19) |
| Uzbekistan | 12.90 (8.01-20.05) | 47.94 (30.03-71.99) | 0.12 (0.08-0.19) | 0.28 (0.18-0.42) | 2.58 (2.44,2.72) |  | 13.59 (8.44-21.29) | 49.74 (31.09-75.10) | 0.13 (0.08-0.20) | 0.29 (0.18-0.44) | 2.57 (2.44,2.70) |  | 394.29 (246.87-594.54) | 1449.72 (928.20-2195.67) | 3.76 (2.36-5.67) | 8.47 (5.42-12.83) | 2.63 (2.49,2.78) |
| Vanuatu | 0.11 (0.06-0.23) | 0.35 (0.20-0.59) | 0.15 (0.08-0.30) | 0.22 (0.13-0.38) | 1.44 (1.34,1.54) |  | 0.12 (0.06-0.24) | 0.37 (0.21-0.62) | 0.15 (0.08-0.31) | 0.23 (0.13-0.40) | 1.44 (1.31,1.56) |  | 3.54 (1.85-7.14) | 10.65 (6.33-17.37) | 4.66 (2.43-9.37) | 6.80 (4.05-11.10) | 1.29 (1.17,1.41) |
| Venezuela (Bolivarian Republic of) | 21.31 (14.45-29.57) | 38.58 (24.78-58.21) | 0.23 (0.15-0.31) | 0.29 (0.19-0.44) | 1.11 (-0.11,2.34) |  | 22.91 (15.43-31.86) | 41.69 (26.25-62.21) | 0.24 (0.16-0.34) | 0.31 (0.20-0.47) | 1.00 (-0.39,2.41) |  | 585.05 (417.80-794.79) | 1030.97 (674.83-1515.59) | 6.22 (4.44-8.45) | 7.74 (5.07-11.38) | 0.91 (-0.42,2.26) |
| Viet Nam | 196.81 (122.10-309.94) | 508.44 (317.53-768.36) | 0.58 (0.36-0.91) | 1.01 (0.63-1.53) | 2.01 (1.87,2.15) |  | 208.15 (128.04-325.91) | 509.99 (322.78-763.52) | 0.61 (0.38-0.96) | 1.02 (0.64-1.52) | 1.84 (1.73,1.96) |  | 5624.02 (3426.36-8796.09) | 13773.46 (8540.24-21196.57) | 16.49 (10.04-25.78) | 27.47 (17.03-42.28) | 1.88 (1.72,2.04) |
| Yemen | 4.66 (1.71-10.22) | 11.40 (5.83-21.59) | 0.07 (0.03-0.15) | 0.07 (0.03-0.13) | -0.05 (-0.25,0.16) |  | 4.89 (1.80-10.74) | 12.02 (6.04-23.30) | 0.07 (0.03-0.16) | 0.07 (0.04-0.14) | -0.05 (-0.24,0.14) |  | 140.54 (51.21-308.91) | 338.33 (173.38-645.12) | 2.06 (0.75-4.53) | 2.01 (1.03-3.84) | -0.12 (-0.31,0.06) |
| Zambia | 16.18 (9.61-26.87) | 22.94 (9.48-54.72) | 0.41 (0.24-0.68) | 0.24 (0.10-0.56) | -3.24 (-3.72,-2.75) |  | 16.85 (9.95-28.38) | 24.26 (10.13-57.48) | 0.42 (0.25-0.72) | 0.25 (0.10-0.59) | -3.19 (-3.70,-2.69) |  | 526.30 (319.25-872.79) | 713.55 (281.00-1790.75) | 13.26 (8.05-21.99) | 7.31 (2.88-18.35) | -3.41 (-3.93,-2.89) |
| Zimbabwe | 23.87 (13.84-37.63) | 59.58 (33.88-90.65) | 0.46 (0.27-0.73) | 0.76 (0.43-1.16) | 0.96 (0.32,1.61) |  | 25.38 (14.81-40.38) | 62.71 (35.64-95.60) | 0.49 (0.29-0.78) | 0.80 (0.46-1.23) | 0.98 (0.30,1.65) |  | 696.16 (411.23-1080.63) | 1846.03 (1043.72-2816.90) | 13.46 (7.95-20.90) | 23.67 (13.38-36.12) | 1.31 (0.62,2.01) |

**Abbreviations:** UI, uncertainty interval; ASR, age-standardised rate per 100,000; EAPC, estimated annual percentage change; CI, confidence interval; DALYs, disability-adjusted life-year; SDI, socio-demographic index.
