# Supplementary material for: Burden of liver cancer from 1990 to 2021 and modelled projection to 2040: insights from the global burden of disease study 2021
Source: Front Oncol. 2026 Apr 29;16:1699519. doi: 10.3389/fonc.2026.1699519 (PMC13167583; doi:10.3389/fonc.2026.1699519)
Supplement: Supplementary file 2 [file DataSheet2.docx]

**Data Sheet 2. Burden of liver cancer due to hepatitis B in 204 countries and territories in 1990, 2021 and the estimated annual percentage changes (EAPC) from 1990 to 2021**

| **Country** | **Incidence** | | | | |  | **Deaths** | | | | |  | **DALYs** | | | | |
| --- | --- | --- | --- | --- | --- | --- | --- | --- | --- | --- | --- | --- | --- | --- | --- | --- | --- |
|  | **1990 No. (95% UI)** | **2021 No. (95% UI)** | **1990 ASR per 100 000 (95% UI)** | **2021 ASR per 100 000 (95% UI)** | **EAPC (95% CI)** |  | **1990 No. (95% UI)** | **2021 No. (95% UI)** | **1990 ASR per 100 000 (95% UI)** | **2021 ASR per 100 000 (95% UI)** | **EAPC (95% CI)** |  | **1990 No. (95% UI)** | **2021 No. (95% UI)** | **1990 ASR per 100 000 (95% UI)** | **2021 ASR per 100 000 (95% UI)** | **EAPC (95% CI)** |
| Afghanistan | 78.33 (49.28-121.24) | 127.98 (82.40-187.02) | 1.58 (0.99-2.44) | 0.82 (0.53-1.20) | -2.20 (-2.55,-1.84) |  | 79.93 (49.63-126.53) | 126.60 (81.69-183.44) | 1.61 (1.00-2.54) | 0.81 (0.52-1.18) | -2.30 (-2.66,-1.95) |  | 2629.83 (1657.84-3972.35) | 4883.17 (3192.93-7165.25) | 52.90 (33.35-79.90) | 31.28 (20.45-45.90) | -1.79 (-2.15,-1.42) |
| Albania | 38.25 (24.52-55.22) | 40.56 (22.97-68.07) | 2.31 (1.48-3.34) | 3.04 (1.72-5.10) | 0.54 (0.05,1.03) |  | 39.63 (25.37-57.46) | 42.48 (23.99-72.08) | 2.40 (1.54-3.48) | 3.18 (1.80-5.40) | 0.51 (-0.04,1.05) |  | 1235.68 (813.81-1746.74) | 1109.39 (633.67-1829.69) | 74.77 (49.24-105.70) | 83.17 (47.50-137.17) | -0.12 (-0.63,0.40) |
| Algeria | 32.51 (23.18-43.95) | 110.06 (72.42-160.97) | 0.26 (0.18-0.35) | 0.50 (0.33-0.73) | 2.15 (2.09,2.21) |  | 32.91 (23.41-44.80) | 108.52 (71.05-158.88) | 0.26 (0.19-0.35) | 0.49 (0.32-0.72) | 2.04 (1.97,2.11) |  | 1126.76 (815.12-1496.57) | 3496.06 (2288.45-4999.00) | 8.91 (6.45-11.84) | 15.82 (10.36-22.62) | 1.85 (1.80,1.90) |
| American Samoa | 0.27 (0.19-0.38) | 0.81 (0.57-1.12) | 1.13 (0.80-1.57) | 3.26 (2.29-4.48) | 3.52 (3.37,3.68) |  | 0.27 (0.19-0.38) | 0.80 (0.56-1.11) | 1.12 (0.79-1.56) | 3.21 (2.26-4.45) | 3.62 (3.44,3.80) |  | 9.70 (6.97-13.50) | 26.39 (18.78-35.67) | 39.99 (28.72-55.68) | 106.07 (75.47-143.36) | 3.42 (3.23,3.60) |
| Andorra | 0.46 (0.27-0.73) | 1.02 (0.60-1.63) | 1.70 (1.00-2.68) | 2.38 (1.40-3.81) | 0.98 (0.75,1.21) |  | 0.43 (0.26-0.68) | 0.89 (0.52-1.45) | 1.59 (0.94-2.51) | 2.09 (1.22-3.38) | 0.81 (0.56,1.07) |  | 13.08 (7.79-20.44) | 24.27 (13.70-39.31) | 48.13 (28.65-75.19) | 56.71 (32.01-91.84) | 0.43 (0.19,0.66) |
| Angola | 86.80 (10.90-247.16) | 142.94 (32.35-367.72) | 1.69 (0.21-4.81) | 0.87 (0.20-2.25) | -2.49 (-2.68,-2.29) |  | 86.11 (10.71-244.07) | 141.70 (32.10-365.78) | 1.68 (0.21-4.75) | 0.87 (0.20-2.24) | -2.45 (-2.63,-2.26) |  | 3275.54 (413.54-9432.52) | 5466.80 (1241.09-14956.71) | 63.75 (8.05-183.58) | 33.43 (7.59-91.46) | -2.35 (-2.52,-2.19) |
| Antigua and Barbuda | 0.22 (0.16-0.30) | 0.32 (0.23-0.44) | 0.73 (0.53-0.99) | 0.72 (0.50-0.99) | -0.50 (-1.09,0.10) |  | 0.23 (0.17-0.31) | 0.33 (0.23-0.45) | 0.76 (0.55-1.02) | 0.74 (0.52-1.00) | -0.70 (-1.28,-0.11) |  | 6.62 (4.84-8.59) | 9.40 (6.59-12.44) | 21.99 (16.09-28.53) | 21.02 (14.74-27.83) | -0.68 (-1.20,-0.16) |
| Argentina | 18.21 (12.56-25.49) | 59.14 (41.81-83.55) | 0.11 (0.08-0.15) | 0.26 (0.18-0.37) | 3.31 (2.99,3.63) |  | 18.57 (12.84-26.18) | 59.48 (42.18-84.66) | 0.11 (0.08-0.16) | 0.26 (0.19-0.37) | 3.33 (2.91,3.74) |  | 568.09 (393.67-780.47) | 1741.83 (1251.41-2353.84) | 3.43 (2.38-4.71) | 7.66 (5.50-10.35) | 3.16 (2.76,3.57) |
| Armenia | 26.70 (18.06-36.86) | 27.85 (18.06-40.11) | 1.56 (1.06-2.16) | 1.86 (1.21-2.68) | 0.70 (0.15,1.25) |  | 27.34 (18.33-38.02) | 28.72 (18.62-41.46) | 1.60 (1.07-2.22) | 1.92 (1.24-2.77) | 0.41 (-0.23,1.05) |  | 849.78 (585.84-1161.88) | 816.72 (540.72-1163.60) | 49.69 (34.25-67.93) | 54.53 (36.10-77.70) | 0.15 (-0.43,0.73) |
| Australia | 29.53 (20.77-41.34) | 158.76 (106.47-227.78) | 0.35 (0.25-0.49) | 1.23 (0.83-1.77) | 4.12 (3.88,4.35) |  | 28.03 (19.43-40.33) | 131.85 (87.07-192.06) | 0.33 (0.23-0.48) | 1.02 (0.68-1.49) | 3.46 (3.26,3.65) |  | 879.30 (629.25-1190.74) | 3558.50 (2377.85-5007.40) | 10.43 (7.46-14.13) | 27.59 (18.44-38.83) | 3.03 (2.85,3.20) |
| Austria | 11.65 (7.74-17.26) | 30.23 (19.72-45.07) | 0.30 (0.20-0.44) | 0.67 (0.44-1.00) | 2.66 (2.25,3.08) |  | 10.89 (7.28-16.22) | 24.75 (15.85-37.10) | 0.28 (0.19-0.42) | 0.55 (0.35-0.83) | 2.14 (1.82,2.47) |  | 308.90 (207.28-446.82) | 621.68 (401.97-905.66) | 7.95 (5.34-11.50) | 13.84 (8.95-20.17) | 1.67 (1.34,2.00) |
| Azerbaijan | 44.65 (23.98-72.66) | 97.54 (48.12-179.20) | 1.22 (0.65-1.98) | 1.86 (0.92-3.41) | 0.99 (0.84,1.13) |  | 45.07 (24.40-73.79) | 98.47 (47.85-181.22) | 1.23 (0.67-2.01) | 1.88 (0.91-3.45) | 1.01 (0.85,1.17) |  | 1533.37 (843.12-2464.75) | 3168.71 (1548.07-5742.65) | 41.86 (23.02-67.28) | 60.36 (29.49-109.38) | 0.76 (0.56,0.96) |
| Bahamas | 0.81 (0.58-1.08) | 1.65 (1.11-2.37) | 0.63 (0.45-0.84) | 0.85 (0.57-1.22) | 0.62 (0.36,0.89) |  | 0.81 (0.57-1.07) | 1.64 (1.11-2.34) | 0.63 (0.45-0.84) | 0.85 (0.57-1.21) | 0.63 (0.34,0.91) |  | 28.19 (20.87-36.66) | 54.25 (37.40-77.79) | 21.97 (16.26-28.57) | 27.96 (19.28-40.10) | 0.40 (0.13,0.67) |
| Bahrain | 2.54 (1.69-3.58) | 7.08 (4.61-10.60) | 1.00 (0.67-1.41) | 0.93 (0.60-1.39) | -1.54 (-2.20,-0.87) |  | 2.55 (1.70-3.59) | 6.70 (4.33-10.07) | 1.01 (0.67-1.42) | 0.88 (0.57-1.32) | -1.82 (-2.53,-1.11) |  | 84.03 (57.67-117.28) | 219.87 (144.99-328.20) | 33.18 (22.77-46.31) | 28.75 (18.96-42.91) | -1.66 (-2.31,-1.01) |
| Bangladesh | 197.03 (141.17-275.59) | 462.75 (293.63-705.67) | 0.36 (0.26-0.51) | 0.56 (0.36-0.86) | 1.64 (1.46,1.82) |  | 199.65 (142.75-277.43) | 471.26 (295.58-723.96) | 0.37 (0.26-0.51) | 0.57 (0.36-0.88) | 1.73 (1.53,1.92) |  | 7076.74 (5186.97-9653.13) | 15037.96 (9867.08-23351.11) | 12.97 (9.51-17.69) | 18.27 (11.99-28.37) | 1.36 (1.17,1.55) |
| Barbados | 0.72 (0.51-0.98) | 1.17 (0.75-1.75) | 0.57 (0.41-0.78) | 0.78 (0.50-1.17) | 0.51 (0.31,0.70) |  | 0.76 (0.54-1.04) | 1.21 (0.78-1.83) | 0.60 (0.43-0.82) | 0.81 (0.52-1.22) | 0.42 (0.18,0.67) |  | 21.10 (15.59-28.00) | 32.19 (20.98-47.52) | 16.65 (12.31-22.10) | 21.53 (14.03-31.79) | 0.32 (0.10,0.53) |
| Belarus | 38.71 (26.63-55.03) | 45.40 (29.29-69.25) | 0.74 (0.51-1.05) | 0.97 (0.63-1.49) | 0.36 (0.18,0.54) |  | 39.26 (26.92-55.36) | 45.51 (29.44-68.89) | 0.75 (0.52-1.06) | 0.98 (0.63-1.48) | 0.31 (0.12,0.49) |  | 1217.39 (842.46-1707.44) | 1358.48 (889.47-2036.85) | 23.31 (16.13-32.70) | 29.14 (19.08-43.69) | 0.19 (0.01,0.38) |
| Belgium | 23.68 (15.86-35.04) | 46.92 (31.70-69.01) | 0.47 (0.32-0.70) | 0.82 (0.55-1.20) | 1.60 (1.40,1.81) |  | 23.76 (15.86-34.79) | 43.58 (28.66-64.94) | 0.48 (0.32-0.70) | 0.76 (0.50-1.13) | 1.42 (1.09,1.74) |  | 616.25 (427.43-866.92) | 1077.18 (730.85-1561.69) | 12.35 (8.57-17.38) | 18.78 (12.74-27.23) | 1.25 (0.99,1.50) |
| Belize | 0.24 (0.17-0.32) | 0.93 (0.69-1.25) | 0.25 (0.18-0.34) | 0.44 (0.32-0.58) | 0.99 (0.60,1.38) |  | 0.24 (0.18-0.33) | 0.93 (0.68-1.24) | 0.26 (0.19-0.35) | 0.43 (0.32-0.58) | 0.86 (0.36,1.36) |  | 7.78 (5.82-10.35) | 32.00 (23.73-42.05) | 8.32 (6.23-11.07) | 14.92 (11.06-19.60) | 1.09 (0.61,1.59) |
| Benin | 116.06 (61.71-170.31) | 175.34 (121.10-248.68) | 4.79 (2.54-7.02) | 2.60 (1.79-3.68) | -2.46 (-2.69,-2.23) |  | 118.35 (62.93-173.96) | 175.41 (120.93-247.89) | 4.88 (2.59-7.17) | 2.60 (1.79-3.67) | -2.46 (-2.69,-2.22) |  | 4001.11 (2135.09-5896.72) | 6423.35 (4410.56-9176.66) | 164.97 (88.03-243.13) | 95.17 (65.35-135.96) | -2.21 (-2.46,-1.97) |
| Bermuda | 0.21 (0.15-0.29) | 0.19 (0.13-0.28) | 0.71 (0.51-0.98) | 0.61 (0.41-0.88) | -0.48 (-0.90,-0.06) |  | 0.22 (0.15-0.30) | 0.19 (0.12-0.27) | 0.73 (0.52-1.00) | 0.60 (0.39-0.85) | -0.76 (-1.22,-0.29) |  | 6.38 (4.63-8.51) | 4.81 (3.31-6.85) | 21.47 (15.60-28.66) | 15.14 (10.40-21.57) | -1.25 (-1.69,-0.81) |
| Bhutan | 1.53 (0.81-2.37) | 3.39 (2.05-5.45) | 0.49 (0.26-0.75) | 0.90 (0.54-1.44) | 1.67 (1.52,1.82) |  | 1.53 (0.81-2.39) | 3.45 (2.07-5.58) | 0.49 (0.26-0.76) | 0.91 (0.55-1.48) | 1.78 (1.61,1.94) |  | 56.62 (29.90-89.02) | 110.84 (67.16-178.75) | 17.97 (9.49-28.26) | 29.29 (17.75-47.23) | 1.31 (1.15,1.47) |
| Bolivia (Plurinational State of) | 22.86 (15.19-32.76) | 56.06 (35.56-86.05) | 0.72 (0.48-1.03) | 0.95 (0.60-1.46) | 0.75 (0.64,0.86) |  | 23.63 (15.74-34.04) | 58.74 (36.92-89.89) | 0.74 (0.49-1.07) | 1.00 (0.63-1.52) | 0.83 (0.74,0.92) |  | 756.65 (509.73-1068.09) | 1682.54 (1081.99-2518.53) | 23.72 (15.98-33.48) | 28.52 (18.34-42.70) | 0.39 (0.26,0.52) |
| Bosnia and Herzegovina | 41.29 (29.41-56.17) | 39.44 (24.17-61.23) | 1.84 (1.31-2.50) | 2.39 (1.46-3.71) | 0.24 (0.02,0.46) |  | 41.94 (29.65-57.23) | 41.13 (25.07-63.94) | 1.86 (1.32-2.54) | 2.49 (1.52-3.87) | 0.27 (0.08,0.47) |  | 1331.82 (962.17-1781.67) | 1059.92 (656.20-1652.27) | 59.21 (42.78-79.22) | 64.18 (39.74-100.05) | -0.46 (-0.68,-0.25) |
| Botswana | 6.59 (3.08-12.82) | 18.16 (8.52-43.07) | 1.00 (0.47-1.94) | 1.52 (0.71-3.60) | 0.05 (-0.47,0.58) |  | 6.59 (3.12-12.96) | 17.93 (8.38-43.35) | 1.00 (0.47-1.96) | 1.50 (0.70-3.62) | 0.08 (-0.48,0.64) |  | 241.85 (110.09-476.49) | 672.65 (294.20-1618.08) | 36.66 (16.69-72.24) | 56.21 (24.59-135.22) | 0.13 (-0.46,0.72) |
| Brazil | 174.88 (152.81-201.00) | 440.39 (369.56-518.15) | 0.24 (0.21-0.27) | 0.40 (0.34-0.47) | 1.98 (1.81,2.16) |  | 175.60 (153.10-202.01) | 445.18 (372.50-523.65) | 0.24 (0.21-0.27) | 0.40 (0.34-0.48) | 2.11 (1.93,2.30) |  | 6274.80 (5521.35-7092.43) | 13748.48 (11609.23-16180.57) | 8.45 (7.44-9.55) | 12.48 (10.54-14.69) | 1.64 (1.45,1.83) |
| Brunei Darussalam | 4.05 (2.86-5.76) | 8.59 (6.08-11.68) | 3.13 (2.21-4.44) | 3.81 (2.70-5.18) | 0.69 (0.43,0.95) |  | 3.99 (2.83-5.66) | 8.03 (5.66-10.93) | 3.08 (2.18-4.37) | 3.56 (2.51-4.85) | 0.50 (0.21,0.80) |  | 131.87 (93.13-183.07) | 257.57 (184.16-356.14) | 101.73 (71.84-141.23) | 114.19 (81.64-157.88) | 0.37 (0.09,0.65) |
| Bulgaria | 115.23 (77.57-164.50) | 45.59 (27.58-69.93) | 2.66 (1.79-3.79) | 1.34 (0.81-2.06) | -2.19 (-2.61,-1.78) |  | 118.72 (79.55-169.67) | 47.26 (28.99-74.28) | 2.74 (1.83-3.91) | 1.39 (0.85-2.19) | -2.11 (-2.61,-1.62) |  | 3479.63 (2408.02-4891.95) | 1313.34 (804.71-1997.67) | 80.18 (55.48-112.72) | 38.70 (23.71-58.87) | -2.29 (-2.76,-1.81) |
| Burkina Faso | 323.41 (154.17-615.59) | 429.01 (199.13-731.96) | 6.79 (3.24-12.92) | 3.77 (1.75-6.43) | -2.01 (-2.21,-1.80) |  | 327.77 (156.18-629.93) | 429.05 (199.44-735.88) | 6.88 (3.28-13.22) | 3.77 (1.75-6.47) | -2.05 (-2.26,-1.83) |  | 11059.82 (5281.53-21337.53) | 15753.44 (7290.84-27728.92) | 232.15 (110.86-447.89) | 138.42 (64.06-243.65) | -1.75 (-1.99,-1.52) |
| Burundi | 15.91 (8.88-32.68) | 20.80 (11.54-40.19) | 0.57 (0.32-1.18) | 0.31 (0.17-0.61) | -2.59 (-2.82,-2.37) |  | 15.99 (8.92-32.75) | 20.72 (11.53-40.14) | 0.58 (0.32-1.18) | 0.31 (0.17-0.61) | -2.58 (-2.81,-2.36) |  | 594.10 (334.32-1227.30) | 800.09 (458.94-1595.22) | 21.40 (12.04-44.20) | 12.10 (6.94-24.13) | -2.41 (-2.62,-2.20) |
| Cabo Verde | 5.08 (3.47-7.50) | 12.41 (8.34-17.38) | 2.87 (1.96-4.24) | 4.44 (2.98-6.21) | 1.22 (1.12,1.32) |  | 5.33 (3.61-8.00) | 12.50 (8.14-17.60) | 3.01 (2.04-4.52) | 4.47 (2.91-6.30) | 1.04 (0.90,1.19) |  | 168.34 (118.12-241.57) | 415.84 (284.39-587.48) | 95.16 (66.77-136.55) | 148.71 (101.70-210.09) | 1.31 (1.23,1.39) |
| Cambodia | 93.52 (47.98-179.19) | 153.23 (73.86-303.27) | 1.82 (0.93-3.49) | 1.80 (0.87-3.56) | -0.04 (-0.10,0.02) |  | 93.98 (48.12-180.67) | 152.82 (73.46-304.73) | 1.83 (0.94-3.52) | 1.79 (0.86-3.58) | -0.06 (-0.13,0.01) |  | 3297.47 (1744.43-6376.32) | 5107.82 (2470.87-10004.34) | 64.20 (33.96-124.14) | 59.94 (28.99-117.39) | -0.25 (-0.32,-0.17) |
| Cameroon | 227.86 (151.48-383.30) | 407.75 (227.75-747.90) | 4.37 (2.90-7.35) | 2.57 (1.43-4.71) | -2.27 (-2.47,-2.07) |  | 229.01 (150.90-388.58) | 403.37 (227.73-733.36) | 4.39 (2.89-7.45) | 2.54 (1.43-4.62) | -2.30 (-2.50,-2.09) |  | 8092.52 (5428.87-13413.00) | 15257.04 (8296.77-27171.46) | 155.08 (104.03-257.03) | 96.02 (52.21-171.00) | -2.06 (-2.27,-1.86) |
| Canada | 28.10 (19.34-39.69) | 113.22 (74.79-163.48) | 0.21 (0.14-0.29) | 0.60 (0.40-0.87) | 3.72 (3.54,3.89) |  | 25.42 (17.46-35.94) | 97.94 (64.51-143.48) | 0.19 (0.13-0.26) | 0.52 (0.34-0.77) | 3.71 (3.54,3.88) |  | 768.40 (532.14-1075.99) | 2447.09 (1590.18-3514.91) | 5.64 (3.91-7.90) | 13.06 (8.49-18.76) | 3.04 (2.86,3.22) |
| Central African Republic | 22.83 (8.82-45.16) | 25.34 (10.58-53.21) | 1.67 (0.65-3.31) | 0.92 (0.39-1.94) | -2.42 (-2.58,-2.26) |  | 22.63 (8.79-45.19) | 24.86 (10.49-51.92) | 1.66 (0.64-3.31) | 0.91 (0.38-1.89) | -2.50 (-2.67,-2.33) |  | 851.28 (322.01-1679.42) | 976.45 (401.92-2086.84) | 62.35 (23.58-123.01) | 35.61 (14.66-76.10) | -2.37 (-2.55,-2.20) |
| Chad | 130.45 (58.25-243.59) | 220.31 (125.26-378.70) | 4.33 (1.93-8.08) | 2.48 (1.41-4.27) | -2.19 (-2.44,-1.93) |  | 133.59 (59.82-250.08) | 221.48 (126.02-380.91) | 4.43 (1.99-8.30) | 2.50 (1.42-4.29) | -2.21 (-2.48,-1.95) |  | 4396.62 (1924.36-8288.27) | 7985.65 (4547.36-13879.54) | 145.91 (63.86-275.06) | 89.98 (51.24-156.39) | -1.91 (-2.19,-1.63) |
| Chile | 16.43 (11.17-23.27) | 65.67 (44.68-94.83) | 0.25 (0.17-0.35) | 0.70 (0.48-1.01) | 3.81 (3.62,4.01) |  | 16.79 (11.44-23.80) | 65.12 (44.06-95.76) | 0.25 (0.17-0.36) | 0.69 (0.47-1.02) | 3.81 (3.61,4.00) |  | 497.23 (341.87-701.68) | 1690.80 (1159.61-2428.36) | 7.49 (5.15-10.56) | 17.99 (12.34-25.84) | 3.38 (3.19,3.58) |
| China | 31559.19 (26008.94-37613.25) | 59332.31 (46139.90-76777.91) | 5.37 (4.42-6.39) | 8.34 (6.49-10.79) | 1.54 (1.37,1.72) |  | 30707.26 (25371.60-36560.92) | 50096.81 (38860.60-64569.19) | 5.22 (4.31-6.22) | 7.04 (5.46-9.08) | 1.03 (0.84,1.21) |  | 1118038.67 (921308.04-1331679.00) | 1574276.41 (1221432.47-2054507.00) | 190.07 (156.62-226.39) | 221.30 (171.70-288.81) | 0.47 (0.27,0.67) |
| Colombia | 61.05 (45.23-79.24) | 112.26 (75.51-162.63) | 0.38 (0.28-0.49) | 0.46 (0.31-0.66) | 0.60 (0.04,1.17) |  | 62.14 (45.93-81.50) | 115.74 (78.18-167.00) | 0.38 (0.28-0.50) | 0.47 (0.32-0.68) | 0.60 (0.03,1.18) |  | 2107.52 (1630.40-2629.19) | 3188.18 (2162.83-4539.36) | 12.97 (10.04-16.18) | 13.00 (8.82-18.51) | -0.13 (-0.69,0.43) |
| Comoros | 1.83 (1.02-2.86) | 3.33 (1.88-5.38) | 0.79 (0.44-1.24) | 0.89 (0.51-1.45) | -0.09 (-0.31,0.13) |  | 1.86 (1.03-2.89) | 3.37 (1.90-5.56) | 0.80 (0.44-1.25) | 0.91 (0.51-1.49) | -0.08 (-0.30,0.13) |  | 65.45 (36.43-103.17) | 116.91 (66.92-192.61) | 28.30 (15.75-44.61) | 31.41 (17.98-51.75) | -0.18 (-0.46,0.10) |
| Congo | 19.56 (9.09-37.94) | 25.31 (11.88-50.13) | 1.63 (0.76-3.16) | 0.94 (0.44-1.86) | -2.31 (-2.56,-2.05) |  | 19.48 (8.99-37.47) | 24.84 (11.75-49.21) | 1.62 (0.75-3.12) | 0.92 (0.44-1.83) | -2.32 (-2.58,-2.05) |  | 721.65 (331.12-1416.34) | 952.51 (439.71-1896.63) | 60.09 (27.57-117.94) | 35.33 (16.31-70.35) | -2.21 (-2.47,-1.94) |
| Cook Islands | 0.34 (0.25-0.47) | 0.57 (0.39-0.80) | 3.61 (2.60-5.00) | 6.37 (4.39-9.05) | 1.92 (1.84,1.99) |  | 0.34 (0.25-0.47) | 0.54 (0.37-0.77) | 3.62 (2.61-4.96) | 6.09 (4.19-8.63) | 1.79 (1.70,1.88) |  | 11.23 (8.03-15.22) | 16.18 (11.13-23.16) | 118.66 (84.84-160.90) | 182.32 (125.44-261.02) | 1.57 (1.47,1.67) |
| Costa Rica | 7.07 (5.00-9.67) | 20.69 (13.81-29.50) | 0.46 (0.33-0.64) | 0.87 (0.58-1.24) | 1.69 (1.31,2.06) |  | 7.15 (5.00-9.89) | 20.77 (13.93-30.09) | 0.47 (0.33-0.65) | 0.88 (0.59-1.27) | 1.77 (1.33,2.21) |  | 232.59 (168.79-302.80) | 597.20 (400.14-832.72) | 15.29 (11.10-19.91) | 25.16 (16.86-35.08) | 1.28 (0.86,1.70) |
| Côte d'Ivoire | 86.56 (54.26-129.87) | 116.84 (67.01-188.07) | 1.42 (0.89-2.13) | 0.84 (0.48-1.35) | -2.35 (-2.56,-2.14) |  | 86.37 (54.40-128.38) | 116.13 (66.41-188.76) | 1.42 (0.89-2.10) | 0.83 (0.48-1.35) | -2.30 (-2.52,-2.09) |  | 3144.78 (1937.51-4679.75) | 4232.76 (2451.18-6979.76) | 51.56 (31.77-76.73) | 30.38 (17.60-50.10) | -2.28 (-2.50,-2.06) |
| Croatia | 22.82 (15.69-30.73) | 33.18 (21.38-48.20) | 0.94 (0.65-1.26) | 1.58 (1.02-2.29) | 1.97 (1.53,2.42) |  | 22.67 (15.58-30.57) | 31.55 (19.97-45.91) | 0.93 (0.64-1.26) | 1.50 (0.95-2.18) | 1.93 (1.48,2.38) |  | 676.74 (474.18-909.41) | 765.42 (496.68-1105.86) | 27.84 (19.51-37.41) | 36.37 (23.60-52.55) | 1.17 (0.73,1.62) |
| Cuba | 28.08 (20.90-37.31) | 31.47 (21.60-45.36) | 0.52 (0.39-0.69) | 0.56 (0.38-0.80) | -0.31 (-0.82,0.21) |  | 28.72 (21.35-38.10) | 31.48 (21.70-45.04) | 0.53 (0.39-0.70) | 0.56 (0.39-0.80) | -0.46 (-1.06,0.13) |  | 865.50 (659.56-1109.59) | 886.78 (614.42-1268.78) | 15.96 (12.16-20.46) | 15.74 (10.90-22.52) | -0.67 (-1.22,-0.11) |
| Cyprus | 1.93 (1.21-2.92) | 4.31 (2.71-6.92) | 0.50 (0.31-0.75) | 0.64 (0.40-1.02) | 0.75 (0.68,0.83) |  | 1.95 (1.23-2.97) | 3.96 (2.45-6.38) | 0.50 (0.32-0.76) | 0.58 (0.36-0.94) | 0.40 (0.28,0.51) |  | 54.86 (35.25-79.48) | 99.97 (61.69-155.65) | 14.10 (9.06-20.43) | 14.73 (9.09-22.93) | -0.03 (-0.13,0.07) |
| Czechia | 70.60 (48.66-97.21) | 47.31 (29.95-68.74) | 1.37 (0.95-1.89) | 0.89 (0.56-1.29) | -1.58 (-1.92,-1.25) |  | 73.00 (50.53-101.23) | 48.29 (30.74-70.67) | 1.42 (0.98-1.97) | 0.91 (0.58-1.33) | -1.65 (-1.98,-1.32) |  | 2097.08 (1484.49-2853.69) | 1185.92 (769.01-1698.49) | 40.74 (28.84-55.44) | 22.31 (14.47-31.95) | -2.19 (-2.55,-1.83) |
| Democratic People's Republic of Korea | 590.82 (315.32-911.53) | 693.63 (433.33-1034.70) | 5.74 (3.06-8.85) | 5.26 (3.28-7.84) | -0.62 (-0.75,-0.49) |  | 580.56 (311.74-893.95) | 668.03 (418.13-992.99) | 5.64 (3.03-8.68) | 5.06 (3.17-7.52) | -0.67 (-0.80,-0.54) |  | 21244.16 (11403.77-32952.13) | 23073.59 (13825.18-34324.02) | 206.34 (110.76-320.06) | 174.85 (104.77-260.11) | -0.86 (-0.99,-0.74) |
| Democratic Republic of the Congo | 93.54 (44.11-213.86) | 155.64 (67.02-370.12) | 0.49 (0.23-1.12) | 0.35 (0.15-0.82) | -1.30 (-1.43,-1.17) |  | 93.75 (43.97-211.58) | 154.73 (66.43-373.30) | 0.49 (0.23-1.11) | 0.34 (0.15-0.83) | -1.35 (-1.48,-1.23) |  | 3463.91 (1665.32-7772.75) | 5882.04 (2571.62-14101.54) | 18.16 (8.73-40.75) | 13.07 (5.71-31.33) | -1.23 (-1.35,-1.11) |
| Denmark | 9.87 (6.81-13.89) | 25.73 (16.53-38.98) | 0.38 (0.26-0.54) | 0.88 (0.56-1.33) | 2.50 (2.24,2.77) |  | 7.85 (5.38-11.09) | 21.93 (13.76-33.87) | 0.31 (0.21-0.43) | 0.75 (0.47-1.16) | 2.89 (2.56,3.22) |  | 221.28 (155.08-303.54) | 513.06 (330.92-760.83) | 8.60 (6.03-11.80) | 17.54 (11.31-26.00) | 2.30 (1.92,2.68) |
| Djibouti | 1.03 (0.57-1.87) | 5.06 (2.86-8.34) | 0.50 (0.28-0.90) | 0.80 (0.45-1.33) | 1.39 (1.15,1.63) |  | 1.02 (0.57-1.85) | 5.00 (2.84-8.24) | 0.49 (0.28-0.89) | 0.79 (0.45-1.31) | 1.48 (1.20,1.77) |  | 39.54 (22.19-71.66) | 184.27 (103.85-307.29) | 19.09 (10.71-34.60) | 29.28 (16.50-48.83) | 1.34 (1.02,1.66) |
| Dominica | 0.19 (0.12-0.29) | 0.32 (0.20-0.48) | 0.53 (0.33-0.80) | 0.95 (0.59-1.44) | 1.76 (1.56,1.95) |  | 0.20 (0.13-0.31) | 0.33 (0.21-0.49) | 0.56 (0.35-0.85) | 0.99 (0.62-1.47) | 1.77 (1.54,2.01) |  | 5.59 (3.63-8.28) | 9.29 (5.89-13.83) | 15.43 (10.03-22.87) | 27.69 (17.55-41.23) | 1.81 (1.60,2.03) |
| Dominican Republic | 7.66 (5.21-10.82) | 24.96 (15.81-37.16) | 0.21 (0.15-0.30) | 0.45 (0.29-0.67) | 2.62 (2.49,2.75) |  | 7.75 (5.26-11.02) | 25.21 (15.73-37.81) | 0.22 (0.15-0.31) | 0.46 (0.29-0.69) | 2.62 (2.48,2.75) |  | 275.45 (191.35-383.73) | 831.08 (536.42-1203.23) | 7.70 (5.35-10.73) | 15.09 (9.74-21.85) | 2.35 (2.20,2.51) |
| Ecuador | 48.22 (39.28-58.24) | 83.03 (57.79-116.83) | 0.97 (0.79-1.17) | 0.92 (0.64-1.29) | -0.13 (-0.77,0.52) |  | 50.02 (40.33-60.74) | 87.29 (60.53-124.35) | 1.00 (0.81-1.22) | 0.97 (0.67-1.38) | -0.29 (-0.95,0.37) |  | 1637.13 (1386.41-1922.84) | 2424.64 (1722.30-3366.54) | 32.81 (27.79-38.54) | 26.84 (19.07-37.27) | -0.95 (-1.61,-0.28) |
| Egypt | 280.64 (172.16-485.58) | 782.62 (461.70-1188.96) | 1.01 (0.62-1.76) | 1.48 (0.87-2.25) | 0.89 (0.69,1.09) |  | 280.66 (170.77-488.10) | 771.58 (454.78-1182.42) | 1.01 (0.62-1.76) | 1.46 (0.86-2.24) | 0.80 (0.56,1.04) |  | 9896.92 (6277.83-16604.29) | 25753.59 (15836.74-37798.00) | 35.77 (22.69-60.02) | 48.76 (29.99-71.57) | 0.65 (0.45,0.86) |
| El Salvador | 5.12 (3.59-6.97) | 7.25 (4.77-10.75) | 0.19 (0.14-0.26) | 0.22 (0.15-0.33) | 0.60 (0.24,0.97) |  | 5.24 (3.68-7.21) | 7.31 (4.81-10.87) | 0.20 (0.14-0.27) | 0.23 (0.15-0.34) | 0.41 (0.01,0.82) |  | 176.89 (130.47-227.05) | 231.81 (154.01-332.38) | 6.67 (4.92-8.56) | 7.19 (4.78-10.31) | 0.26 (-0.15,0.67) |
| Equatorial Guinea | 0.90 (0.42-2.21) | 4.02 (2.18-7.38) | 0.42 (0.20-1.05) | 0.53 (0.29-0.98) | 0.85 (0.29,1.42) |  | 0.90 (0.42-2.23) | 3.93 (2.15-7.17) | 0.43 (0.20-1.06) | 0.52 (0.28-0.95) | 0.84 (0.28,1.41) |  | 33.04 (15.69-78.21) | 156.45 (83.70-286.07) | 15.63 (7.42-36.99) | 20.69 (11.07-37.83) | 1.06 (0.51,1.62) |
| Eritrea | 9.00 (5.45-15.81) | 17.32 (10.04-29.69) | 0.53 (0.32-0.93) | 0.52 (0.30-0.90) | -0.74 (-0.95,-0.53) |  | 8.87 (5.42-15.65) | 17.11 (9.96-29.37) | 0.52 (0.32-0.92) | 0.52 (0.30-0.89) | -0.67 (-0.91,-0.43) |  | 345.65 (212.60-609.45) | 660.23 (383.37-1144.30) | 20.30 (12.48-35.79) | 20.01 (11.62-34.69) | -0.70 (-0.95,-0.46) |
| Estonia | 7.80 (5.38-10.78) | 8.10 (5.30-11.32) | 1.00 (0.69-1.38) | 1.24 (0.81-1.73) | -0.23 (-0.60,0.15) |  | 7.90 (5.39-10.86) | 8.32 (5.43-11.75) | 1.01 (0.69-1.38) | 1.27 (0.83-1.79) | 0.04 (-0.34,0.41) |  | 247.74 (178.12-338.42) | 215.35 (143.00-299.27) | 31.59 (22.71-43.15) | 32.86 (21.82-45.66) | -0.66 (-1.03,-0.30) |
| Eswatini | 6.12 (2.06-11.52) | 28.62 (9.08-67.44) | 1.52 (0.51-2.86) | 4.95 (1.57-11.68) | 3.84 (2.42,5.28) |  | 6.09 (2.07-11.29) | 27.98 (8.71-65.99) | 1.51 (0.51-2.80) | 4.84 (1.51-11.42) | 3.83 (2.38,5.31) |  | 225.83 (75.12-430.13) | 1109.91 (356.14-2687.81) | 56.01 (18.63-106.68) | 192.15 (61.66-465.33) | 4.08 (2.57,5.60) |
| Ethiopia | 121.82 (88.63-174.52) | 161.24 (106.55-265.82) | 0.48 (0.35-0.69) | 0.30 (0.20-0.49) | -2.09 (-2.38,-1.81) |  | 122.25 (88.82-174.97) | 162.59 (107.50-267.70) | 0.48 (0.35-0.69) | 0.30 (0.20-0.49) | -2.05 (-2.33,-1.77) |  | 4551.88 (3263.90-6502.93) | 5973.30 (3956.89-9712.74) | 18.00 (12.91-25.72) | 10.97 (7.26-17.83) | -2.13 (-2.42,-1.85) |
| Fiji | 4.74 (3.28-6.93) | 9.47 (6.13-13.75) | 1.25 (0.87-1.83) | 2.05 (1.33-2.97) | 2.01 (1.73,2.30) |  | 4.69 (3.22-6.90) | 9.39 (5.98-13.54) | 1.24 (0.85-1.82) | 2.03 (1.29-2.93) | 2.10 (1.76,2.45) |  | 170.56 (118.76-248.20) | 314.98 (202.75-452.33) | 44.98 (31.32-65.45) | 68.14 (43.86-97.86) | 1.87 (1.52,2.22) |
| Finland | 18.12 (12.57-25.34) | 44.90 (29.03-69.36) | 0.72 (0.50-1.01) | 1.62 (1.05-2.51) | 2.82 (2.75,2.90) |  | 15.36 (10.65-21.82) | 33.69 (21.96-51.89) | 0.61 (0.43-0.87) | 1.22 (0.79-1.87) | 2.44 (2.25,2.62) |  | 437.02 (306.90-598.49) | 761.55 (495.89-1127.46) | 17.45 (12.25-23.89) | 27.51 (17.92-40.73) | 1.68 (1.52,1.84) |
| France | 263.36 (175.92-381.44) | 548.79 (362.92-809.91) | 0.91 (0.61-1.32) | 1.65 (1.09-2.44) | 1.81 (1.57,2.04) |  | 258.97 (173.46-377.15) | 460.42 (303.33-685.93) | 0.90 (0.60-1.31) | 1.39 (0.91-2.07) | 1.17 (0.90,1.43) |  | 7214.56 (4943.88-10078.59) | 11364.75 (7463.16-16600.79) | 24.98 (17.12-34.89) | 34.24 (22.48-50.01) | 0.82 (0.55,1.10) |
| Gabon | 6.98 (2.99-15.05) | 12.09 (6.27-20.70) | 1.42 (0.61-3.06) | 1.33 (0.69-2.28) | -0.39 (-0.47,-0.31) |  | 7.03 (3.00-14.95) | 11.93 (6.20-20.40) | 1.43 (0.61-3.04) | 1.31 (0.68-2.25) | -0.39 (-0.49,-0.29) |  | 246.00 (104.89-541.82) | 432.37 (220.40-742.82) | 50.02 (21.33-110.18) | 47.62 (24.27-81.80) | -0.28 (-0.41,-0.15) |
| Gambia | 34.73 (24.79-46.84) | 97.08 (60.38-146.64) | 7.08 (5.05-9.54) | 8.11 (5.04-12.25) | -0.06 (-0.24,0.13) |  | 34.60 (24.66-47.01) | 96.23 (60.24-144.61) | 7.05 (5.02-9.58) | 8.04 (5.03-12.08) | 0.01 (-0.20,0.23) |  | 1262.10 (889.16-1706.72) | 3543.39 (2181.94-5331.62) | 257.15 (181.16-347.74) | 296.00 (182.27-445.39) | -0.00 (-0.25,0.24) |
| Georgia | 39.23 (27.95-54.66) | 25.35 (17.04-36.02) | 1.42 (1.01-1.98) | 1.41 (0.94-2.00) | -0.59 (-1.72,0.55) |  | 39.62 (28.29-55.48) | 25.95 (17.36-37.26) | 1.43 (1.02-2.01) | 1.44 (0.96-2.07) | -0.55 (-1.73,0.64) |  | 1265.78 (921.99-1742.95) | 767.33 (515.65-1087.68) | 45.84 (33.39-63.12) | 42.54 (28.59-60.30) | -0.75 (-1.90,0.42) |
| Germany | 202.89 (142.51-293.98) | 444.08 (283.94-653.54) | 0.51 (0.36-0.74) | 1.04 (0.67-1.53) | 2.29 (1.97,2.61) |  | 196.09 (136.37-285.09) | 369.22 (237.90-545.03) | 0.49 (0.34-0.71) | 0.86 (0.56-1.28) | 1.92 (1.69,2.15) |  | 5197.02 (3737.58-7195.73) | 8798.71 (5673.11-12730.94) | 13.00 (9.35-18.00) | 20.61 (13.29-29.82) | 1.47 (1.22,1.72) |
| Ghana | 216.72 (138.72-332.76) | 355.58 (229.86-548.86) | 2.90 (1.85-4.45) | 2.08 (1.34-3.21) | -1.59 (-1.98,-1.20) |  | 216.61 (138.72-332.28) | 354.00 (228.63-549.03) | 2.89 (1.85-4.44) | 2.07 (1.34-3.21) | -1.62 (-2.01,-1.24) |  | 8309.66 (5207.83-12736.53) | 13460.29 (8829.08-20302.05) | 111.01 (69.57-170.14) | 78.61 (51.56-118.57) | -1.64 (-2.02,-1.26) |
| Greece | 58.43 (45.78-71.95) | 176.33 (133.01-223.32) | 1.12 (0.88-1.39) | 3.47 (2.61-4.39) | 3.41 (3.07,3.76) |  | 57.49 (44.13-71.92) | 171.03 (128.84-217.04) | 1.11 (0.85-1.38) | 3.36 (2.53-4.27) | 3.56 (3.20,3.92) |  | 1511.13 (1216.41-1818.54) | 4012.48 (3073.04-5026.56) | 29.09 (23.42-35.01) | 78.87 (60.40-98.80) | 3.17 (2.82,3.53) |
| Greenland | 0.15 (0.10-0.23) | 0.24 (0.14-0.38) | 0.55 (0.36-0.81) | 0.86 (0.49-1.37) | 1.68 (1.58,1.78) |  | 0.15 (0.10-0.22) | 0.24 (0.14-0.38) | 0.54 (0.35-0.80) | 0.85 (0.49-1.35) | 1.74 (1.63,1.86) |  | 5.70 (3.68-8.29) | 7.16 (4.22-11.47) | 20.51 (13.26-29.83) | 25.53 (15.05-40.87) | 1.01 (0.88,1.14) |
| Grenada | 0.19 (0.13-0.27) | 0.46 (0.31-0.65) | 0.44 (0.31-0.62) | 0.89 (0.60-1.27) | 1.90 (1.39,2.40) |  | 0.20 (0.14-0.29) | 0.47 (0.31-0.67) | 0.46 (0.32-0.66) | 0.92 (0.61-1.30) | 1.78 (1.25,2.32) |  | 5.94 (4.19-8.33) | 13.84 (9.49-19.46) | 13.65 (9.62-19.15) | 26.97 (18.49-37.93) | 1.83 (1.40,2.26) |
| Guam | 0.73 (0.58-0.90) | 3.17 (2.40-4.01) | 1.07 (0.85-1.31) | 3.98 (3.02-5.04) | 5.12 (4.81,5.42) |  | 0.72 (0.57-0.89) | 3.07 (2.32-3.91) | 1.05 (0.83-1.29) | 3.85 (2.92-4.91) | 5.10 (4.81,5.39) |  | 24.93 (20.05-30.69) | 99.14 (76.22-122.06) | 36.45 (29.31-44.87) | 124.56 (95.75-153.34) | 4.90 (4.57,5.24) |
| Guatemala | 24.10 (17.45-31.90) | 42.90 (29.30-60.52) | 0.57 (0.42-0.76) | 0.54 (0.37-0.77) | -0.27 (-1.58,1.06) |  | 24.49 (17.72-32.97) | 43.74 (29.90-61.78) | 0.58 (0.42-0.79) | 0.55 (0.38-0.78) | -0.68 (-2.26,0.92) |  | 867.71 (645.10-1120.52) | 1445.62 (1020.36-1955.48) | 20.70 (15.39-26.73) | 18.34 (12.94-24.80) | -0.95 (-2.34,0.47) |
| Guinea | 219.95 (163.13-293.17) | 301.91 (187.92-483.17) | 7.34 (5.44-9.78) | 4.50 (2.80-7.20) | -1.35 (-1.66,-1.04) |  | 224.16 (165.41-297.28) | 303.39 (189.29-484.55) | 7.48 (5.52-9.92) | 4.52 (2.82-7.22) | -1.36 (-1.68,-1.04) |  | 7418.08 (5488.85-9755.50) | 10730.13 (6749.07-17209.65) | 247.52 (183.14-325.51) | 159.80 (100.51-256.29) | -1.14 (-1.42,-0.86) |
| Guinea-Bissau | 43.46 (17.48-66.14) | 43.65 (26.79-63.21) | 8.63 (3.47-13.13) | 4.23 (2.60-6.13) | -2.59 (-2.68,-2.49) |  | 43.54 (17.49-66.47) | 43.05 (26.54-62.56) | 8.65 (3.47-13.20) | 4.17 (2.57-6.06) | -2.56 (-2.68,-2.45) |  | 1564.03 (627.00-2402.15) | 1685.92 (1024.16-2473.78) | 310.60 (124.52-477.04) | 163.37 (99.24-239.72) | -2.30 (-2.42,-2.18) |
| Guyana | 1.40 (1.02-1.90) | 1.91 (1.27-2.80) | 0.36 (0.26-0.49) | 0.50 (0.33-0.73) | 0.94 (0.73,1.16) |  | 1.42 (1.03-1.92) | 1.91 (1.27-2.79) | 0.36 (0.26-0.49) | 0.50 (0.33-0.73) | 1.08 (0.83,1.32) |  | 48.96 (36.15-65.06) | 65.69 (44.36-93.99) | 12.56 (9.27-16.69) | 17.18 (11.60-24.58) | 1.02 (0.76,1.28) |
| Haiti | 7.72 (4.18-14.29) | 14.37 (6.81-29.46) | 0.24 (0.13-0.45) | 0.22 (0.11-0.46) | -0.24 (-0.36,-0.12) |  | 7.84 (4.20-14.92) | 14.59 (6.88-31.09) | 0.25 (0.13-0.47) | 0.23 (0.11-0.48) | -0.22 (-0.33,-0.10) |  | 271.07 (152.01-485.34) | 500.47 (243.44-993.89) | 8.50 (4.76-15.21) | 7.78 (3.78-15.45) | -0.22 (-0.34,-0.09) |
| Honduras | 3.94 (2.33-7.04) | 15.60 (9.22-24.53) | 0.17 (0.10-0.30) | 0.31 (0.18-0.49) | 1.88 (1.68,2.08) |  | 4.01 (2.37-7.23) | 16.06 (9.51-25.10) | 0.17 (0.10-0.31) | 0.32 (0.19-0.50) | 2.01 (1.79,2.23) |  | 141.37 (88.78-232.27) | 487.30 (288.93-764.89) | 6.00 (3.77-9.86) | 9.64 (5.72-15.13) | 1.43 (1.20,1.66) |
| Hungary | 47.48 (33.40-65.56) | 36.62 (23.67-55.09) | 0.91 (0.64-1.26) | 0.76 (0.49-1.15) | -1.08 (-1.62,-0.54) |  | 48.89 (33.78-67.16) | 37.63 (24.59-56.31) | 0.94 (0.65-1.29) | 0.78 (0.51-1.17) | -1.00 (-1.56,-0.44) |  | 1460.51 (1044.17-2003.10) | 984.16 (642.03-1470.73) | 28.10 (20.09-38.54) | 20.51 (13.38-30.65) | -1.43 (-1.91,-0.96) |
| Iceland | 0.59 (0.41-0.81) | 1.91 (1.25-2.81) | 0.46 (0.32-0.64) | 1.09 (0.71-1.60) | 2.60 (2.30,2.90) |  | 0.55 (0.38-0.78) | 1.67 (1.09-2.45) | 0.44 (0.30-0.62) | 0.95 (0.62-1.40) | 2.29 (1.94,2.64) |  | 15.70 (10.97-21.58) | 41.21 (26.96-60.16) | 12.37 (8.64-17.00) | 23.52 (15.39-34.34) | 1.86 (1.56,2.17) |
| India | 2086.09 (1731.30-2461.66) | 6048.03 (5081.74-7287.45) | 0.49 (0.41-0.58) | 0.86 (0.72-1.03) | 1.79 (1.72,1.86) |  | 2100.70 (1735.32-2484.07) | 6124.64 (5146.94-7368.72) | 0.49 (0.41-0.58) | 0.87 (0.73-1.04) | 1.78 (1.70,1.86) |  | 72707.83 (61484.07-85200.88) | 194798.66 (164047.45-233018.79) | 17.05 (14.41-19.98) | 27.54 (23.20-32.95) | 1.46 (1.41,1.52) |
| Indonesia | 523.81 (355.67-794.53) | 1436.74 (869.44-2320.91) | 0.57 (0.38-0.86) | 1.03 (0.62-1.66) | 1.97 (1.95,2.00) |  | 518.29 (349.61-788.31) | 1409.27 (848.05-2293.93) | 0.56 (0.38-0.85) | 1.01 (0.61-1.64) | 1.94 (1.90,1.97) |  | 19481.66 (13117.24-29559.41) | 49314.30 (30419.23-79676.08) | 21.06 (14.18-31.96) | 35.36 (21.81-57.13) | 1.70 (1.67,1.73) |
| Iran (Islamic Republic of) | 140.41 (113.75-179.68) | 495.55 (433.25-566.90) | 0.49 (0.40-0.63) | 1.16 (1.02-1.33) | 2.47 (1.70,3.24) |  | 142.72 (115.40-182.84) | 494.84 (430.01-565.13) | 0.50 (0.40-0.64) | 1.16 (1.01-1.32) | 2.36 (1.57,3.16) |  | 4738.98 (3857.95-6008.20) | 14668.18 (12879.51-16564.32) | 16.60 (13.51-21.04) | 34.37 (30.18-38.81) | 2.00 (1.23,2.78) |
| Iraq | 65.08 (44.45-92.84) | 201.55 (128.25-286.67) | 0.71 (0.48-1.01) | 0.98 (0.62-1.39) | 1.23 (0.89,1.56) |  | 65.73 (45.54-93.72) | 198.76 (125.71-283.80) | 0.71 (0.49-1.02) | 0.96 (0.61-1.38) | 1.14 (0.78,1.50) |  | 2227.93 (1564.35-3127.23) | 6409.13 (4194.04-9255.35) | 24.19 (16.99-33.96) | 31.09 (20.35-44.90) | 0.98 (0.68,1.28) |
| Ireland | 5.24 (3.53-7.47) | 16.65 (10.51-24.61) | 0.29 (0.20-0.41) | 0.67 (0.43-1.00) | 3.20 (3.02,3.38) |  | 5.22 (3.54-7.48) | 14.85 (9.50-21.98) | 0.29 (0.20-0.42) | 0.60 (0.38-0.89) | 2.74 (2.55,2.93) |  | 142.09 (99.11-196.00) | 371.61 (239.50-538.49) | 7.89 (5.50-10.88) | 15.04 (9.69-21.80) | 2.39 (2.20,2.58) |
| Israel | 8.72 (5.88-12.36) | 21.52 (14.36-31.08) | 0.35 (0.24-0.50) | 0.45 (0.30-0.65) | 0.72 (0.56,0.87) |  | 8.92 (5.96-12.86) | 20.24 (13.31-29.54) | 0.36 (0.24-0.52) | 0.42 (0.28-0.62) | 0.39 (0.26,0.53) |  | 234.68 (159.73-321.15) | 526.38 (348.80-742.76) | 9.46 (6.44-12.95) | 10.97 (7.27-15.48) | 0.39 (0.21,0.57) |
| Italy | 360.52 (294.80-437.01) | 362.42 (292.23-437.32) | 1.27 (1.04-1.54) | 1.21 (0.98-1.46) | -0.49 (-0.78,-0.20) |  | 332.85 (270.59-407.59) | 292.29 (235.19-355.93) | 1.17 (0.95-1.44) | 0.98 (0.79-1.19) | -1.06 (-1.48,-0.64) |  | 9204.75 (7520.76-11090.93) | 7053.56 (5702.35-8520.96) | 32.41 (26.48-39.05) | 23.59 (19.07-28.49) | -1.50 (-1.92,-1.08) |
| Jamaica | 2.46 (1.67-3.45) | 5.65 (3.63-8.45) | 0.21 (0.14-0.29) | 0.40 (0.26-0.60) | 2.00 (1.77,2.23) |  | 2.58 (1.76-3.63) | 5.77 (3.71-8.59) | 0.22 (0.15-0.31) | 0.41 (0.26-0.61) | 1.98 (1.49,2.48) |  | 72.69 (51.74-99.07) | 171.39 (112.59-258.18) | 6.15 (4.37-8.38) | 12.24 (8.04-18.44) | 2.07 (1.53,2.62) |
| Japan | 1852.92 (1566.60-2144.58) | 2032.27 (1611.54-2484.57) | 2.95 (2.49-3.41) | 3.18 (2.52-3.89) | -0.61 (-1.09,-0.14) |  | 1466.47 (1254.89-1698.91) | 1521.03 (1196.29-1859.10) | 2.33 (1.99-2.70) | 2.38 (1.87-2.91) | -0.65 (-1.17,-0.12) |  | 45263.87 (38987.44-52503.15) | 29704.67 (24100.88-35699.57) | 71.95 (61.97-83.45) | 46.52 (37.75-55.91) | -2.21 (-2.73,-1.68) |
| Jordan | 5.84 (3.56-9.32) | 19.59 (13.03-28.75) | 0.31 (0.19-0.50) | 0.32 (0.21-0.47) | -0.67 (-0.93,-0.41) |  | 5.84 (3.57-9.40) | 18.91 (12.44-27.89) | 0.31 (0.19-0.50) | 0.31 (0.20-0.45) | -0.77 (-1.05,-0.49) |  | 198.62 (124.24-314.51) | 613.04 (414.88-895.93) | 10.63 (6.65-16.84) | 9.95 (6.73-14.54) | -0.93 (-1.21,-0.65) |
| Kazakhstan | 195.57 (138.84-264.72) | 97.47 (63.98-137.17) | 2.39 (1.69-3.23) | 1.03 (0.68-1.45) | -3.83 (-4.26,-3.39) |  | 195.49 (138.07-265.04) | 97.92 (64.27-138.31) | 2.39 (1.68-3.23) | 1.03 (0.68-1.46) | -3.87 (-4.32,-3.42) |  | 6728.23 (4826.54-8846.22) | 3161.04 (2119.53-4358.50) | 82.09 (58.89-107.93) | 33.35 (22.36-45.99) | -4.07 (-4.53,-3.61) |
| Kenya | 25.27 (17.56-39.03) | 110.16 (79.20-157.12) | 0.22 (0.15-0.34) | 0.44 (0.32-0.63) | 2.12 (1.88,2.37) |  | 25.31 (17.56-39.01) | 108.96 (78.41-155.83) | 0.22 (0.15-0.34) | 0.44 (0.31-0.62) | 2.06 (1.82,2.30) |  | 950.60 (661.74-1450.54) | 4076.38 (2909.71-5903.55) | 8.21 (5.72-12.53) | 16.29 (11.62-23.58) | 2.06 (1.79,2.34) |
| Kiribati | 0.73 (0.52-1.01) | 1.23 (0.83-1.82) | 1.96 (1.40-2.71) | 2.03 (1.37-3.00) | -0.22 (-0.46,0.03) |  | 0.73 (0.52-0.99) | 1.21 (0.82-1.81) | 1.95 (1.39-2.67) | 2.00 (1.35-2.99) | -0.21 (-0.44,0.02) |  | 27.00 (19.26-37.60) | 45.13 (30.38-67.58) | 72.57 (51.77-101.08) | 74.49 (50.15-111.56) | -0.19 (-0.44,0.07) |
| Kuwait | 6.19 (4.79-7.77) | 5.63 (3.93-7.69) | 0.72 (0.56-0.90) | 0.24 (0.17-0.33) | -3.27 (-3.68,-2.85) |  | 5.90 (4.54-7.45) | 5.13 (3.55-7.12) | 0.69 (0.53-0.87) | 0.22 (0.15-0.31) | -3.52 (-4.54,-2.49) |  | 216.62 (168.82-268.78) | 166.60 (116.30-232.05) | 25.21 (19.65-31.28) | 7.17 (5.00-9.98) | -3.80 (-4.84,-2.75) |
| Kyrgyzstan | 27.90 (19.86-38.55) | 20.43 (13.20-29.67) | 1.25 (0.89-1.73) | 0.60 (0.38-0.86) | -2.12 (-2.95,-1.28) |  | 27.89 (19.77-38.46) | 20.57 (13.18-30.06) | 1.25 (0.89-1.72) | 0.60 (0.38-0.88) | -2.48 (-3.42,-1.54) |  | 982.85 (704.80-1349.39) | 660.53 (440.85-964.22) | 44.04 (31.58-60.46) | 19.25 (12.85-28.10) | -2.69 (-3.57,-1.80) |
| Lao People's Democratic Republic | 52.02 (31.84-76.07) | 68.85 (43.97-103.57) | 2.50 (1.53-3.65) | 1.87 (1.19-2.81) | -1.18 (-1.27,-1.10) |  | 52.24 (32.28-77.30) | 68.48 (43.79-104.12) | 2.51 (1.55-3.71) | 1.86 (1.19-2.82) | -1.22 (-1.31,-1.13) |  | 1834.53 (1111.78-2661.64) | 2397.56 (1507.44-3589.24) | 88.00 (53.33-127.67) | 65.00 (40.87-97.30) | -1.22 (-1.29,-1.14) |
| Latvia | 11.51 (8.16-15.60) | 9.77 (6.44-13.92) | 0.87 (0.61-1.17) | 1.04 (0.69-1.49) | -0.28 (-0.60,0.05) |  | 11.60 (8.14-15.90) | 9.99 (6.55-14.30) | 0.87 (0.61-1.20) | 1.07 (0.70-1.53) | 0.10 (-0.29,0.48) |  | 365.71 (267.45-497.75) | 269.77 (183.51-389.78) | 27.52 (20.12-37.45) | 28.85 (19.62-41.68) | -0.40 (-0.77,-0.03) |
| Lebanon | 17.61 (12.33-24.45) | 30.01 (21.21-41.11) | 1.18 (0.82-1.63) | 1.08 (0.77-1.48) | -0.20 (-0.37,-0.03) |  | 17.86 (12.60-24.70) | 29.43 (20.93-40.59) | 1.19 (0.84-1.65) | 1.06 (0.76-1.47) | -0.35 (-0.54,-0.17) |  | 550.83 (388.79-763.73) | 845.53 (606.95-1131.41) | 36.82 (25.99-51.05) | 30.52 (21.91-40.84) | -0.59 (-0.76,-0.42) |
| Lesotho | 10.33 (3.23-23.45) | 39.65 (13.89-100.47) | 1.35 (0.42-3.06) | 4.23 (1.48-10.72) | 3.36 (2.40,4.32) |  | 10.47 (3.27-23.62) | 39.17 (13.56-99.66) | 1.37 (0.43-3.08) | 4.18 (1.45-10.63) | 3.35 (2.40,4.31) |  | 355.25 (108.81-821.29) | 1482.09 (496.09-3869.37) | 46.36 (14.20-107.18) | 158.14 (52.93-412.87) | 3.78 (2.81,4.77) |
| Liberia | 73.72 (36.18-114.84) | 109.85 (64.87-166.14) | 5.99 (2.94-9.33) | 4.02 (2.38-6.09) | -1.87 (-2.20,-1.53) |  | 75.48 (37.38-118.62) | 108.53 (64.57-162.89) | 6.14 (3.04-9.64) | 3.98 (2.37-5.97) | -2.07 (-2.45,-1.68) |  | 2453.38 (1196.32-3836.50) | 4105.16 (2425.22-6172.69) | 199.41 (97.24-311.83) | 150.39 (88.84-226.13) | -1.45 (-1.84,-1.05) |
| Libya | 19.06 (12.43-28.66) | 70.32 (44.63-107.30) | 0.90 (0.59-1.36) | 2.05 (1.30-3.12) | 2.77 (2.57,2.97) |  | 19.08 (12.51-28.32) | 68.12 (43.12-102.62) | 0.91 (0.59-1.34) | 1.98 (1.26-2.99) | 2.60 (2.38,2.82) |  | 655.96 (435.22-958.05) | 2364.65 (1516.06-3566.59) | 31.12 (20.65-45.46) | 68.83 (44.13-103.82) | 2.73 (2.52,2.94) |
| Lithuania | 12.06 (8.58-16.35) | 17.92 (11.78-25.55) | 0.66 (0.47-0.89) | 1.31 (0.86-1.87) | 1.58 (1.26,1.89) |  | 11.71 (8.33-15.87) | 16.97 (11.19-24.05) | 0.64 (0.45-0.86) | 1.24 (0.82-1.76) | 1.75 (1.45,2.06) |  | 365.09 (268.21-487.95) | 470.99 (310.75-661.09) | 19.87 (14.60-26.56) | 34.53 (22.78-48.46) | 1.33 (1.02,1.64) |
| Luxembourg | 1.18 (0.80-1.70) | 2.66 (1.80-3.81) | 0.62 (0.42-0.89) | 0.83 (0.56-1.18) | 0.88 (0.73,1.03) |  | 1.18 (0.80-1.72) | 2.43 (1.62-3.50) | 0.62 (0.42-0.90) | 0.75 (0.50-1.09) | 0.55 (0.38,0.73) |  | 32.70 (22.21-46.36) | 61.52 (40.67-87.43) | 17.16 (11.66-24.33) | 19.10 (12.62-27.14) | 0.21 (0.04,0.38) |
| Madagascar | 30.24 (18.40-49.43) | 54.46 (30.77-88.95) | 0.51 (0.31-0.83) | 0.38 (0.22-0.62) | -1.14 (-1.27,-1.01) |  | 30.24 (18.30-49.25) | 53.92 (30.41-88.60) | 0.51 (0.31-0.83) | 0.38 (0.21-0.62) | -1.21 (-1.35,-1.06) |  | 1152.52 (729.12-1817.16) | 2099.10 (1217.67-3460.88) | 19.37 (12.25-30.54) | 14.70 (8.53-24.24) | -1.09 (-1.22,-0.96) |
| Malawi | 26.35 (16.71-41.34) | 66.85 (42.22-99.03) | 0.54 (0.34-0.84) | 0.69 (0.43-1.02) | -0.30 (-0.94,0.34) |  | 26.45 (16.81-41.13) | 66.23 (41.96-98.05) | 0.54 (0.34-0.84) | 0.68 (0.43-1.01) | -0.30 (-0.95,0.35) |  | 976.93 (627.31-1514.75) | 2528.87 (1625.65-3802.33) | 19.93 (12.79-30.90) | 26.01 (16.72-39.10) | -0.15 (-0.79,0.50) |
| Malaysia | 116.23 (85.85-151.15) | 442.47 (317.36-593.27) | 1.32 (0.97-1.71) | 2.78 (2.00-3.73) | 2.61 (2.43,2.79) |  | 118.23 (87.52-153.86) | 435.19 (312.70-587.39) | 1.34 (0.99-1.74) | 2.74 (1.97-3.69) | 2.54 (2.34,2.74) |  | 3814.64 (2865.01-4954.17) | 13425.27 (9687.65-17841.81) | 43.18 (32.43-56.08) | 84.40 (60.91-112.17) | 2.37 (2.16,2.59) |
| Maldives | 1.55 (1.05-2.17) | 3.18 (2.18-4.46) | 1.39 (0.95-1.95) | 1.23 (0.84-1.72) | -0.85 (-1.01,-0.68) |  | 1.57 (1.08-2.20) | 3.10 (2.11-4.42) | 1.41 (0.97-1.98) | 1.20 (0.81-1.71) | -0.95 (-1.09,-0.81) |  | 51.15 (34.32-71.15) | 97.49 (67.68-138.58) | 46.02 (30.88-64.03) | 37.70 (26.17-53.59) | -1.20 (-1.47,-0.94) |
| Mali | 301.31 (224.38-392.21) | 658.60 (443.04-903.63) | 6.96 (5.18-9.06) | 5.46 (3.68-7.50) | -0.74 (-0.86,-0.63) |  | 304.34 (225.93-395.92) | 661.05 (438.36-918.69) | 7.03 (5.22-9.14) | 5.48 (3.64-7.62) | -0.74 (-0.85,-0.62) |  | 10313.78 (7718.74-13394.70) | 23255.46 (15716.09-32333.37) | 238.12 (178.20-309.25) | 192.96 (130.40-268.28) | -0.61 (-0.73,-0.49) |
| Malta | 0.66 (0.44-0.96) | 1.82 (1.15-2.78) | 0.36 (0.24-0.52) | 0.82 (0.52-1.26) | 2.84 (2.73,2.94) |  | 0.66 (0.45-0.97) | 1.71 (1.09-2.63) | 0.36 (0.24-0.52) | 0.77 (0.49-1.19) | 2.75 (2.63,2.86) |  | 18.08 (12.15-25.75) | 41.33 (26.59-61.63) | 9.76 (6.56-13.90) | 18.69 (12.03-27.87) | 2.33 (2.19,2.47) |
| Marshall Islands | 0.16 (0.09-0.29) | 0.40 (0.21-0.75) | 0.70 (0.40-1.28) | 1.42 (0.76-2.65) | 2.33 (2.21,2.44) |  | 0.16 (0.09-0.29) | 0.39 (0.21-0.74) | 0.69 (0.40-1.28) | 1.40 (0.74-2.62) | 2.28 (2.17,2.38) |  | 5.73 (3.41-10.35) | 14.45 (7.84-26.96) | 25.22 (15.01-45.57) | 51.36 (27.87-95.80) | 2.25 (2.08,2.41) |
| Mauritania | 116.32 (25.11-231.77) | 110.13 (51.73-176.06) | 11.32 (2.44-22.56) | 5.01 (2.35-8.01) | -3.04 (-3.22,-2.85) |  | 118.42 (25.53-236.24) | 112.06 (52.63-178.58) | 11.53 (2.49-23.00) | 5.10 (2.39-8.12) | -3.05 (-3.23,-2.87) |  | 3961.60 (840.48-8036.87) | 3657.80 (1708.31-5843.45) | 385.63 (81.81-782.32) | 166.41 (77.72-265.84) | -3.13 (-3.31,-2.96) |
| Mauritius | 6.53 (5.05-8.10) | 2.46 (1.77-3.21) | 1.19 (0.92-1.48) | 0.39 (0.28-0.50) | 0.83 (-0.98,2.69) |  | 6.59 (5.04-8.23) | 2.42 (1.74-3.16) | 1.20 (0.92-1.50) | 0.38 (0.27-0.50) | 2.35 (0.42,4.32) |  | 209.74 (165.87-254.97) | 71.44 (52.95-90.99) | 38.27 (30.27-46.53) | 11.23 (8.33-14.31) | 2.14 (0.20,4.11) |
| Mexico | 48.86 (41.03-57.71) | 159.53 (127.15-200.76) | 0.11 (0.10-0.14) | 0.25 (0.20-0.31) | 2.53 (2.21,2.85) |  | 49.70 (41.89-58.71) | 161.94 (128.84-203.19) | 0.12 (0.10-0.14) | 0.25 (0.20-0.31) | 2.55 (2.24,2.86) |  | 1742.54 (1491.44-2038.71) | 5027.17 (4003.83-6230.96) | 4.08 (3.49-4.77) | 7.78 (6.19-9.64) | 2.19 (1.90,2.48) |
| Micronesia (Federated States of) | 0.70 (0.45-1.11) | 1.08 (0.61-1.86) | 1.36 (0.87-2.15) | 2.11 (1.20-3.63) | 1.37 (1.25,1.48) |  | 0.70 (0.45-1.11) | 1.06 (0.60-1.83) | 1.36 (0.87-2.15) | 2.07 (1.17-3.57) | 1.35 (1.24,1.46) |  | 25.24 (16.08-39.78) | 37.95 (21.53-65.24) | 48.77 (31.07-76.88) | 73.99 (41.98-127.21) | 1.33 (1.19,1.48) |
| Monaco | 0.19 (0.11-0.30) | 0.48 (0.29-0.75) | 1.22 (0.72-1.96) | 2.54 (1.51-3.97) | 2.47 (1.96,2.99) |  | 0.18 (0.10-0.30) | 0.44 (0.26-0.70) | 1.19 (0.69-1.94) | 2.33 (1.39-3.69) | 2.27 (1.75,2.80) |  | 4.57 (2.77-7.21) | 10.86 (6.41-16.91) | 30.07 (18.23-47.40) | 57.38 (33.88-89.34) | 2.21 (1.68,2.73) |
| Mongolia | 102.12 (66.90-153.69) | 224.83 (145.44-337.43) | 9.47 (6.20-14.24) | 13.48 (8.72-20.23) | 1.14 (0.92,1.36) |  | 103.34 (67.74-154.85) | 225.54 (145.98-340.43) | 9.58 (6.28-14.35) | 13.52 (8.75-20.41) | 1.10 (0.90,1.29) |  | 3573.29 (2324.64-5389.91) | 7632.09 (4983.46-11418.56) | 331.19 (215.46-499.57) | 457.52 (298.74-684.51) | 0.97 (0.74,1.21) |
| Montenegro | 4.57 (3.02-6.64) | 6.77 (4.19-10.37) | 1.46 (0.97-2.12) | 2.19 (1.35-3.36) | 1.58 (1.34,1.82) |  | 4.62 (3.05-6.70) | 6.91 (4.25-10.69) | 1.48 (0.97-2.14) | 2.24 (1.37-3.46) | 1.64 (1.39,1.89) |  | 142.52 (98.08-206.17) | 186.36 (118.18-286.48) | 45.52 (31.32-65.85) | 60.30 (38.24-92.70) | 1.15 (0.92,1.39) |
| Morocco | 12.12 (8.10-17.04) | 32.88 (20.29-49.53) | 0.10 (0.06-0.13) | 0.18 (0.11-0.27) | 1.86 (1.70,2.01) |  | 12.29 (8.20-17.38) | 33.02 (20.21-49.73) | 0.10 (0.06-0.14) | 0.18 (0.11-0.27) | 1.80 (1.64,1.97) |  | 406.83 (278.54-570.70) | 1037.74 (653.59-1502.55) | 3.21 (2.20-4.50) | 5.58 (3.52-8.08) | 1.63 (1.47,1.79) |
| Mozambique | 221.84 (126.22-341.71) | 502.24 (244.00-1127.40) | 3.32 (1.89-5.12) | 3.23 (1.57-7.26) | -0.06 (-0.38,0.26) |  | 228.03 (128.32-349.18) | 508.24 (244.10-1122.58) | 3.41 (1.92-5.23) | 3.27 (1.57-7.23) | -0.10 (-0.41,0.22) |  | 7098.08 (4092.88-10934.73) | 16931.86 (8153.81-38011.92) | 106.26 (61.27-163.69) | 108.98 (52.48-244.66) | 0.18 (-0.14,0.51) |
| Myanmar | 193.00 (89.03-382.79) | 321.90 (161.38-663.88) | 0.95 (0.44-1.89) | 1.14 (0.57-2.35) | 0.27 (0.19,0.36) |  | 193.97 (89.03-381.60) | 321.30 (160.50-662.45) | 0.96 (0.44-1.89) | 1.14 (0.57-2.35) | 0.24 (0.16,0.33) |  | 6794.61 (3111.80-13686.21) | 10603.50 (5334.73-21898.58) | 33.60 (15.39-67.69) | 37.59 (18.91-77.63) | 0.07 (-0.01,0.15) |
| Namibia | 2.64 (1.29-5.08) | 8.28 (5.56-12.55) | 0.38 (0.18-0.72) | 0.68 (0.46-1.03) | 1.54 (0.90,2.19) |  | 2.67 (1.31-5.16) | 8.22 (5.54-12.50) | 0.38 (0.19-0.74) | 0.68 (0.46-1.03) | 1.53 (0.89,2.17) |  | 92.25 (45.14-178.80) | 294.79 (196.98-451.02) | 13.14 (6.43-25.47) | 24.25 (16.20-37.10) | 1.63 (0.94,2.32) |
| Nauru | 0.11 (0.08-0.16) | 0.11 (0.06-0.18) | 2.25 (1.55-3.09) | 2.04 (1.17-3.25) | -0.70 (-0.85,-0.54) |  | 0.11 (0.08-0.15) | 0.11 (0.06-0.17) | 2.19 (1.52-3.03) | 1.96 (1.12-3.15) | -0.73 (-0.91,-0.56) |  | 4.36 (2.96-5.96) | 4.31 (2.48-7.02) | 85.40 (58.10-116.81) | 78.14 (44.93-127.44) | -0.64 (-0.82,-0.47) |
| Nepal | 27.69 (17.68-42.24) | 101.40 (60.15-154.21) | 0.28 (0.18-0.43) | 0.65 (0.39-0.99) | 3.17 (2.82,3.52) |  | 27.93 (17.87-42.76) | 103.11 (61.02-158.65) | 0.29 (0.18-0.44) | 0.66 (0.39-1.02) | 3.23 (2.87,3.59) |  | 998.11 (637.52-1510.85) | 3328.26 (2016.21-5077.84) | 10.25 (6.55-15.52) | 21.38 (12.95-32.62) | 2.86 (2.52,3.20) |
| Netherlands | 18.33 (12.69-25.64) | 57.98 (39.41-82.62) | 0.25 (0.17-0.34) | 0.67 (0.46-0.96) | 3.65 (3.45,3.85) |  | 19.02 (13.05-27.04) | 60.30 (40.50-87.20) | 0.25 (0.17-0.36) | 0.70 (0.47-1.01) | 3.61 (3.45,3.78) |  | 540.71 (384.57-743.59) | 1480.03 (988.15-2089.50) | 7.25 (5.15-9.97) | 17.20 (11.48-24.28) | 3.12 (2.97,3.27) |
| New Zealand | 7.99 (6.60-9.59) | 29.87 (24.29-36.70) | 0.47 (0.39-0.56) | 1.16 (0.94-1.42) | 2.98 (2.79,3.18) |  | 6.44 (5.25-7.72) | 20.82 (16.92-25.43) | 0.38 (0.31-0.45) | 0.81 (0.65-0.98) | 2.58 (2.39,2.77) |  | 212.57 (174.74-253.82) | 626.95 (509.90-772.66) | 12.44 (10.23-14.86) | 24.26 (19.73-29.89) | 2.24 (2.05,2.43) |
| Nicaragua | 4.46 (3.06-6.52) | 10.67 (6.86-16.30) | 0.23 (0.16-0.34) | 0.32 (0.21-0.49) | 1.28 (0.97,1.59) |  | 4.52 (3.11-6.64) | 10.69 (6.91-16.35) | 0.23 (0.16-0.34) | 0.32 (0.21-0.49) | 1.29 (0.94,1.64) |  | 160.07 (114.44-221.60) | 352.16 (229.62-530.52) | 8.24 (5.89-11.40) | 10.56 (6.89-15.91) | 1.02 (0.67,1.36) |
| Niger | 159.87 (73.63-277.63) | 183.30 (111.06-312.02) | 3.98 (1.83-6.91) | 1.46 (0.89-2.49) | -3.36 (-3.46,-3.26) |  | 159.86 (73.68-277.90) | 184.39 (112.69-310.99) | 3.98 (1.83-6.92) | 1.47 (0.90-2.48) | -3.27 (-3.38,-3.17) |  | 5786.19 (2644.18-10157.13) | 6612.99 (4039.49-11036.93) | 144.07 (65.84-252.91) | 52.82 (32.27-88.16) | -3.32 (-3.44,-3.20) |
| Nigeria | 554.05 (237.01-1043.68) | 858.70 (558.90-1292.14) | 1.23 (0.53-2.32) | 0.74 (0.48-1.12) | -2.03 (-2.17,-1.89) |  | 565.17 (242.25-1067.04) | 867.07 (573.45-1292.82) | 1.26 (0.54-2.37) | 0.75 (0.50-1.12) | -2.03 (-2.17,-1.89) |  | 18633.96 (7756.87-34893.43) | 29599.50 (18973.30-45411.28) | 41.39 (17.23-77.50) | 25.61 (16.42-39.29) | -1.93 (-2.09,-1.77) |
| Niue | 0.02 (0.01-0.03) | 0.03 (0.02-0.04) | 1.97 (1.26-3.00) | 3.04 (1.84-4.99) | 1.15 (0.89,1.41) |  | 0.02 (0.01-0.04) | 0.03 (0.02-0.04) | 2.02 (1.29-3.06) | 3.02 (1.84-4.90) | 1.09 (0.82,1.36) |  | 0.72 (0.45-1.09) | 0.78 (0.47-1.27) | 62.67 (39.43-94.66) | 93.71 (56.48-152.50) | 1.04 (0.79,1.29) |
| North Macedonia | 22.74 (15.62-33.05) | 28.96 (18.08-43.36) | 2.28 (1.57-3.32) | 2.66 (1.66-3.98) | 0.22 (0.09,0.35) |  | 23.41 (16.20-33.83) | 29.92 (18.80-44.48) | 2.35 (1.63-3.40) | 2.75 (1.73-4.09) | 0.19 (0.06,0.31) |  | 711.36 (504.06-1003.61) | 812.78 (514.45-1209.70) | 71.41 (50.60-100.75) | 74.69 (47.27-111.16) | -0.21 (-0.33,-0.08) |
| Northern Mariana Islands | 0.31 (0.22-0.46) | 0.96 (0.71-1.27) | 1.39 (0.96-2.02) | 3.94 (2.91-5.22) | 4.09 (3.63,4.57) |  | 0.30 (0.21-0.44) | 0.92 (0.69-1.22) | 1.33 (0.93-1.93) | 3.80 (2.86-5.01) | 4.22 (3.73,4.72) |  | 11.81 (7.89-17.08) | 29.13 (21.81-38.34) | 52.33 (34.96-75.70) | 120.13 (89.96-158.14) | 3.44 (3.03,3.86) |
| Norway | 8.22 (6.87-9.77) | 24.68 (20.48-28.88) | 0.39 (0.32-0.46) | 0.91 (0.76-1.07) | 2.66 (2.40,2.92) |  | 7.80 (6.54-9.31) | 20.56 (16.95-24.34) | 0.37 (0.31-0.44) | 0.76 (0.63-0.90) | 2.27 (1.90,2.64) |  | 217.67 (184.93-255.92) | 551.84 (461.81-645.47) | 10.25 (8.71-12.05) | 20.37 (17.05-23.83) | 2.18 (1.85,2.52) |
| Oman | 4.94 (2.97-8.20) | 16.81 (11.08-23.44) | 0.50 (0.30-0.83) | 0.71 (0.47-1.00) | 1.35 (1.16,1.55) |  | 4.89 (2.97-8.07) | 15.55 (10.30-21.74) | 0.49 (0.30-0.81) | 0.66 (0.44-0.92) | 1.23 (0.99,1.47) |  | 172.14 (104.53-285.05) | 566.47 (377.63-770.66) | 17.35 (10.54-28.73) | 24.09 (16.06-32.77) | 1.35 (1.09,1.60) |
| Pakistan | 130.80 (95.13-178.99) | 386.54 (278.05-529.71) | 0.24 (0.17-0.32) | 0.33 (0.24-0.45) | 0.74 (0.56,0.93) |  | 132.35 (96.50-181.80) | 383.65 (278.03-528.36) | 0.24 (0.17-0.33) | 0.33 (0.24-0.45) | 0.71 (0.52,0.90) |  | 4880.75 (3600.74-6625.46) | 14988.75 (11020.85-20470.18) | 8.78 (6.48-11.92) | 12.73 (9.36-17.38) | 0.90 (0.67,1.14) |
| Palau | 0.24 (0.15-0.36) | 0.56 (0.37-0.79) | 3.12 (1.99-4.77) | 6.14 (4.10-8.77) | 2.03 (1.83,2.24) |  | 0.24 (0.15-0.36) | 0.54 (0.36-0.78) | 3.11 (2.00-4.75) | 5.93 (3.98-8.57) | 1.98 (1.81,2.15) |  | 8.27 (5.19-13.02) | 18.93 (12.78-26.95) | 108.93 (68.33-171.37) | 209.17 (141.28-297.80) | 1.99 (1.73,2.24) |
| Palestine | 10.52 (6.86-15.52) | 23.83 (16.73-33.24) | 1.03 (0.67-1.52) | 0.93 (0.65-1.29) | -0.69 (-0.93,-0.45) |  | 10.89 (7.07-16.10) | 23.64 (16.64-33.13) | 1.06 (0.69-1.57) | 0.92 (0.65-1.29) | -0.87 (-1.12,-0.62) |  | 328.20 (220.33-475.31) | 748.87 (538.54-1015.95) | 32.07 (21.53-46.44) | 29.16 (20.97-39.56) | -0.68 (-0.93,-0.43) |
| Panama | 4.52 (3.22-6.23) | 9.03 (5.91-13.38) | 0.38 (0.27-0.52) | 0.42 (0.28-0.62) | 0.77 (0.21,1.34) |  | 4.68 (3.35-6.46) | 9.20 (6.04-13.65) | 0.39 (0.28-0.54) | 0.43 (0.28-0.64) | 0.69 (0.13,1.25) |  | 142.98 (103.31-190.79) | 268.62 (176.27-389.18) | 11.97 (8.65-15.97) | 12.52 (8.21-18.14) | 0.53 (0.02,1.06) |
| Papua New Guinea | 21.03 (9.77-52.61) | 38.81 (17.30-103.76) | 1.03 (0.48-2.56) | 0.74 (0.33-1.98) | -1.24 (-1.32,-1.15) |  | 20.96 (9.76-52.56) | 38.39 (17.13-102.79) | 1.02 (0.48-2.56) | 0.73 (0.33-1.97) | -1.31 (-1.40,-1.21) |  | 753.38 (348.40-1862.36) | 1401.47 (631.44-3770.76) | 36.72 (16.98-90.78) | 26.79 (12.07-72.09) | -1.23 (-1.31,-1.14) |
| Paraguay | 3.98 (2.57-5.59) | 17.14 (10.89-25.89) | 0.20 (0.13-0.28) | 0.48 (0.30-0.72) | 3.78 (3.40,4.16) |  | 4.06 (2.64-5.72) | 17.29 (10.95-25.90) | 0.20 (0.13-0.28) | 0.48 (0.31-0.72) | 3.78 (3.39,4.18) |  | 135.90 (90.03-187.10) | 554.80 (359.43-811.84) | 6.72 (4.45-9.26) | 15.48 (10.03-22.65) | 3.56 (3.19,3.93) |
| Peru | 52.16 (37.30-70.52) | 143.67 (95.12-209.27) | 0.48 (0.34-0.65) | 0.79 (0.52-1.15) | 1.49 (1.31,1.67) |  | 53.78 (38.27-72.55) | 147.75 (97.37-215.99) | 0.50 (0.35-0.67) | 0.81 (0.54-1.19) | 1.55 (1.33,1.78) |  | 1791.31 (1332.17-2372.81) | 4236.88 (2783.59-6019.47) | 16.56 (12.32-21.94) | 23.36 (15.35-33.19) | 1.02 (0.80,1.24) |
| Philippines | 609.77 (424.09-781.42) | 1142.68 (915.74-1432.41) | 1.94 (1.35-2.48) | 2.02 (1.62-2.53) | -0.05 (-0.14,0.04) |  | 598.90 (415.30-771.47) | 1125.45 (901.68-1403.82) | 1.90 (1.32-2.45) | 1.99 (1.59-2.48) | -0.07 (-0.17,0.03) |  | 23474.75 (16456.89-29501.83) | 39587.54 (31788.02-49083.92) | 74.51 (52.24-93.64) | 69.91 (56.14-86.68) | -0.44 (-0.55,-0.33) |
| Poland | 29.81 (24.76-35.10) | 116.54 (95.62-141.15) | 0.16 (0.13-0.18) | 0.61 (0.50-0.74) | 5.31 (4.75,5.87) |  | 31.24 (25.94-36.91) | 121.71 (99.51-148.08) | 0.16 (0.14-0.19) | 0.64 (0.52-0.77) | 5.36 (4.75,5.97) |  | 910.40 (763.17-1076.23) | 3284.65 (2715.62-3955.09) | 4.77 (4.00-5.64) | 17.18 (14.20-20.69) | 5.14 (4.53,5.76) |
| Portugal | 19.88 (13.40-27.99) | 68.65 (44.91-101.09) | 0.39 (0.26-0.55) | 1.29 (0.85-1.91) | 3.55 (3.28,3.82) |  | 20.31 (13.62-28.65) | 67.39 (44.34-99.73) | 0.40 (0.27-0.57) | 1.27 (0.84-1.88) | 3.52 (3.23,3.81) |  | 606.06 (422.87-832.82) | 1774.69 (1147.90-2644.92) | 11.96 (8.34-16.43) | 33.46 (21.64-49.87) | 3.15 (2.88,3.43) |
| Puerto Rico | 12.96 (9.22-17.76) | 21.28 (14.45-30.37) | 0.72 (0.51-0.98) | 1.29 (0.88-1.84) | 1.17 (0.66,1.68) |  | 13.17 (9.30-18.23) | 21.26 (14.28-30.38) | 0.73 (0.51-1.01) | 1.29 (0.87-1.84) | 1.30 (0.86,1.75) |  | 399.91 (299.58-534.84) | 563.76 (389.90-800.03) | 22.14 (16.59-29.61) | 34.23 (23.67-48.57) | 0.76 (0.32,1.21) |
| Qatar | 2.65 (1.79-3.93) | 23.99 (15.34-35.03) | 1.19 (0.80-1.77) | 1.61 (1.03-2.35) | -0.23 (-0.73,0.28) |  | 2.58 (1.74-3.83) | 21.31 (13.46-31.54) | 1.16 (0.78-1.72) | 1.43 (0.90-2.12) | -0.56 (-1.10,-0.02) |  | 94.79 (64.24-141.19) | 759.32 (491.18-1099.22) | 42.62 (28.88-63.48) | 51.01 (33.00-73.85) | -0.48 (-0.96,0.01) |
| Republic of Korea | 3724.38 (2674.27-4785.70) | 5179.46 (4081.29-6541.95) | 16.84 (12.09-21.63) | 20.09 (15.83-25.37) | 0.68 (0.54,0.82) |  | 3568.12 (2555.41-4556.84) | 3699.20 (2902.80-4726.86) | 16.13 (11.55-20.60) | 14.35 (11.26-18.33) | -0.43 (-0.52,-0.34) |  | 116899.78 (83336.43-153459.10) | 96964.99 (76098.98-124089.94) | 528.42 (376.70-693.67) | 376.05 (295.13-481.24) | -1.21 (-1.34,-1.09) |
| Republic of Moldova | 15.50 (10.95-21.35) | 16.48 (11.20-23.40) | 0.70 (0.49-0.96) | 0.92 (0.62-1.30) | 0.21 (-0.68,1.10) |  | 15.72 (11.04-21.61) | 16.81 (11.38-23.87) | 0.71 (0.50-0.97) | 0.94 (0.63-1.33) | 0.15 (-0.79,1.10) |  | 519.48 (380.69-702.09) | 508.38 (352.04-704.64) | 23.36 (17.12-31.57) | 28.29 (19.59-39.22) | -0.08 (-0.97,0.81) |
| Romania | 48.27 (33.08-66.17) | 107.93 (70.20-159.25) | 0.41 (0.28-0.57) | 1.14 (0.74-1.68) | 3.40 (3.11,3.69) |  | 49.57 (33.92-67.97) | 111.15 (71.92-164.41) | 0.42 (0.29-0.58) | 1.17 (0.76-1.74) | 3.52 (3.18,3.87) |  | 1545.28 (1095.44-2089.19) | 3023.75 (1993.85-4483.23) | 13.22 (9.37-17.87) | 31.93 (21.06-47.35) | 3.05 (2.72,3.39) |
| Russian Federation | 365.19 (311.37-428.47) | 601.28 (498.29-725.63) | 0.48 (0.41-0.57) | 0.83 (0.69-1.00) | 2.22 (1.87,2.57) |  | 369.11 (314.28-432.36) | 607.82 (502.99-735.53) | 0.49 (0.42-0.57) | 0.84 (0.69-1.02) | 2.24 (1.81,2.66) |  | 12154.05 (10481.82-14142.51) | 18144.83 (15054.17-21748.78) | 16.10 (13.88-18.73) | 25.05 (20.79-30.03) | 1.93 (1.48,2.39) |
| Rwanda | 29.16 (17.85-45.51) | 37.21 (22.83-58.80) | 0.81 (0.50-1.27) | 0.56 (0.34-0.89) | -2.26 (-2.65,-1.87) |  | 29.15 (17.79-45.69) | 37.20 (22.46-60.37) | 0.81 (0.49-1.27) | 0.56 (0.34-0.91) | -2.05 (-2.51,-1.58) |  | 1106.94 (685.72-1681.05) | 1369.09 (835.55-2166.42) | 30.79 (19.08-46.76) | 20.63 (12.59-32.65) | -2.18 (-2.65,-1.70) |
| Saint Kitts and Nevis | 0.18 (0.12-0.25) | 0.30 (0.20-0.42) | 0.85 (0.58-1.19) | 1.01 (0.67-1.43) | -0.22 (-0.97,0.54) |  | 0.19 (0.13-0.26) | 0.30 (0.20-0.42) | 0.89 (0.62-1.26) | 1.02 (0.68-1.44) | -0.42 (-1.14,0.31) |  | 5.29 (3.80-7.01) | 8.92 (5.86-12.55) | 25.50 (18.32-33.81) | 30.43 (19.99-42.79) | -0.17 (-0.86,0.53) |
| Saint Lucia | 0.24 (0.17-0.33) | 0.42 (0.28-0.58) | 0.36 (0.25-0.48) | 0.47 (0.32-0.66) | 0.44 (0.05,0.84) |  | 0.25 (0.18-0.34) | 0.42 (0.28-0.59) | 0.37 (0.26-0.50) | 0.47 (0.32-0.66) | 0.33 (-0.05,0.71) |  | 7.95 (5.75-10.59) | 13.21 (9.15-17.99) | 11.64 (8.43-15.51) | 14.88 (10.31-20.27) | 0.36 (0.01,0.72) |
| Saint Vincent and the Grenadines | 0.33 (0.24-0.45) | 0.46 (0.32-0.63) | 0.61 (0.44-0.81) | 0.80 (0.56-1.10) | 0.64 (0.35,0.94) |  | 0.34 (0.25-0.46) | 0.47 (0.33-0.64) | 0.63 (0.45-0.84) | 0.82 (0.57-1.12) | 0.55 (0.20,0.89) |  | 10.69 (7.99-13.82) | 14.46 (10.10-19.50) | 19.52 (14.58-25.25) | 25.35 (17.71-34.19) | 0.55 (0.26,0.84) |
| Samoa | 1.23 (0.88-1.67) | 1.81 (1.22-2.55) | 1.46 (1.04-1.97) | 1.69 (1.14-2.39) | 0.31 (0.21,0.41) |  | 1.25 (0.89-1.70) | 1.81 (1.22-2.54) | 1.48 (1.06-2.01) | 1.69 (1.14-2.38) | 0.24 (0.12,0.36) |  | 40.01 (28.74-54.84) | 58.44 (39.05-83.08) | 47.37 (34.03-64.92) | 54.71 (36.56-77.78) | 0.32 (0.20,0.44) |
| San Marino | 0.05 (0.03-0.08) | 0.10 (0.05-0.19) | 0.43 (0.27-0.66) | 0.63 (0.33-1.16) | 1.40 (1.28,1.51) |  | 0.05 (0.03-0.08) | 0.09 (0.05-0.17) | 0.41 (0.25-0.64) | 0.58 (0.31-1.06) | 1.26 (1.11,1.40) |  | 1.28 (0.81-1.90) | 2.33 (1.23-4.14) | 10.77 (6.78-16.02) | 14.24 (7.49-25.30) | 1.05 (0.90,1.20) |
| Sao Tome and Principe | 0.47 (0.35-0.61) | 0.69 (0.39-1.26) | 0.78 (0.58-1.01) | 0.63 (0.36-1.16) | -0.85 (-1.04,-0.66) |  | 0.49 (0.37-0.64) | 0.68 (0.39-1.23) | 0.80 (0.60-1.05) | 0.63 (0.36-1.14) | -0.98 (-1.19,-0.76) |  | 15.79 (11.79-20.53) | 25.08 (14.29-46.33) | 26.04 (19.43-33.86) | 23.15 (13.19-42.78) | -0.58 (-0.82,-0.34) |
| Saudi Arabia | 96.55 (59.61-150.67) | 216.05 (140.06-309.46) | 1.22 (0.75-1.90) | 1.15 (0.74-1.64) | -1.39 (-1.85,-0.92) |  | 96.94 (59.93-151.31) | 206.82 (134.64-299.30) | 1.22 (0.76-1.91) | 1.10 (0.71-1.59) | -1.49 (-1.95,-1.02) |  | 3269.22 (2022.18-5109.73) | 6877.10 (4496.24-9876.81) | 41.24 (25.51-64.45) | 36.48 (23.85-52.39) | -1.55 (-2.04,-1.05) |
| Senegal | 149.94 (80.12-207.73) | 194.56 (134.11-278.35) | 3.93 (2.10-5.44) | 2.45 (1.69-3.51) | -1.72 (-1.81,-1.64) |  | 152.79 (82.38-209.64) | 197.79 (136.39-282.49) | 4.00 (2.16-5.49) | 2.49 (1.72-3.56) | -1.76 (-1.88,-1.63) |  | 5121.19 (2721.36-7238.36) | 6685.85 (4615.09-9533.78) | 134.20 (71.31-189.68) | 84.31 (58.20-120.23) | -1.70 (-1.83,-1.58) |
| Serbia | 63.68 (40.40-93.75) | 68.81 (40.61-109.73) | 1.32 (0.84-1.95) | 1.54 (0.91-2.46) | 0.02 (-0.28,0.32) |  | 64.78 (41.37-95.53) | 70.42 (41.54-113.12) | 1.35 (0.86-1.98) | 1.58 (0.93-2.54) | 0.04 (-0.28,0.35) |  | 1986.86 (1271.28-2937.77) | 1850.54 (1083.71-2872.05) | 41.27 (26.40-61.02) | 41.49 (24.30-64.40) | -0.47 (-0.76,-0.18) |
| Seychelles | 1.01 (0.76-1.36) | 1.02 (0.71-1.38) | 2.78 (2.09-3.74) | 1.93 (1.35-2.62) | -0.94 (-1.58,-0.30) |  | 1.04 (0.77-1.40) | 1.00 (0.70-1.36) | 2.85 (2.12-3.83) | 1.89 (1.32-2.58) | -1.11 (-1.76,-0.45) |  | 32.25 (24.56-42.14) | 31.84 (22.47-42.57) | 88.52 (67.41-115.67) | 60.40 (42.63-80.76) | -0.99 (-1.60,-0.37) |
| Sierra Leone | 109.35 (40.28-194.55) | 93.60 (61.80-141.07) | 5.27 (1.94-9.37) | 2.11 (1.39-3.18) | -3.27 (-3.50,-3.05) |  | 111.98 (41.31-199.15) | 93.83 (62.29-142.69) | 5.39 (1.99-9.59) | 2.12 (1.40-3.22) | -3.34 (-3.58,-3.10) |  | 3674.58 (1318.92-6633.32) | 3411.50 (2217.02-5200.37) | 177.02 (63.54-319.55) | 76.95 (50.00-117.29) | -2.93 (-3.15,-2.71) |
| Singapore | 68.08 (57.84-79.24) | 188.85 (151.00-228.54) | 4.47 (3.80-5.20) | 6.59 (5.27-7.98) | 1.06 (0.86,1.26) |  | 63.99 (53.98-74.87) | 141.15 (112.45-171.32) | 4.20 (3.54-4.91) | 4.93 (3.93-5.98) | 0.46 (0.23,0.69) |  | 1922.44 (1659.22-2191.03) | 3451.23 (2801.72-4088.68) | 126.18 (108.90-143.81) | 120.52 (97.84-142.78) | -0.25 (-0.51,0.01) |
| Slovakia | 38.09 (25.32-54.93) | 36.22 (20.69-58.75) | 1.44 (0.96-2.08) | 1.33 (0.76-2.16) | -0.70 (-0.89,-0.52) |  | 38.74 (25.59-56.33) | 36.54 (20.82-59.57) | 1.47 (0.97-2.13) | 1.35 (0.77-2.19) | -0.80 (-1.01,-0.59) |  | 1168.55 (780.68-1664.73) | 1000.46 (572.95-1608.45) | 44.24 (29.55-63.02) | 36.85 (21.11-59.25) | -1.07 (-1.26,-0.87) |
| Slovenia | 13.22 (9.38-17.85) | 21.32 (13.74-29.60) | 1.34 (0.95-1.81) | 2.06 (1.33-2.86) | 1.28 (1.21,1.35) |  | 13.31 (9.52-17.83) | 21.43 (13.78-29.90) | 1.35 (0.96-1.81) | 2.07 (1.33-2.89) | 1.11 (0.91,1.32) |  | 407.65 (290.69-532.23) | 509.15 (323.09-714.71) | 41.31 (29.46-53.94) | 49.20 (31.22-69.07) | 0.23 (0.03,0.43) |
| Solomon Islands | 3.01 (0.93-6.49) | 5.53 (3.37-9.08) | 1.78 (0.55-3.83) | 1.62 (0.99-2.66) | -0.39 (-0.55,-0.23) |  | 3.00 (0.94-6.65) | 5.39 (3.28-8.96) | 1.77 (0.55-3.92) | 1.58 (0.96-2.62) | -0.47 (-0.62,-0.32) |  | 108.14 (31.88-233.93) | 205.64 (124.20-334.83) | 63.79 (18.81-137.99) | 60.17 (36.34-97.97) | -0.25 (-0.41,-0.09) |
| Somalia | 48.32 (19.02-93.85) | 106.80 (45.04-213.24) | 1.22 (0.48-2.36) | 0.99 (0.42-1.97) | -1.24 (-1.43,-1.04) |  | 47.67 (18.93-93.76) | 105.57 (44.76-207.11) | 1.20 (0.48-2.36) | 0.98 (0.41-1.92) | -1.17 (-1.37,-0.97) |  | 1821.15 (715.64-3565.83) | 4080.13 (1700.99-8183.45) | 45.88 (18.03-89.83) | 37.77 (15.75-75.75) | -1.17 (-1.38,-0.95) |
| South Africa | 182.83 (104.98-306.44) | 549.71 (446.35-666.09) | 0.99 (0.57-1.66) | 1.93 (1.57-2.34) | 1.53 (0.84,2.24) |  | 178.68 (101.96-300.77) | 542.49 (440.87-655.36) | 0.97 (0.55-1.63) | 1.91 (1.55-2.31) | 1.49 (0.77,2.21) |  | 7282.63 (4254.13-11974.27) | 19598.68 (16002.53-23663.08) | 39.35 (22.99-64.70) | 68.94 (56.29-83.24) | 1.07 (0.29,1.85) |
| South Sudan | 22.69 (12.30-37.28) | 47.21 (26.64-77.07) | 0.77 (0.42-1.27) | 0.98 (0.55-1.59) | 0.38 (-0.06,0.83) |  | 22.88 (12.56-37.67) | 47.08 (26.67-77.39) | 0.78 (0.43-1.28) | 0.97 (0.55-1.60) | 0.45 (0.00,0.90) |  | 809.49 (445.35-1327.96) | 1726.87 (1001.77-2839.50) | 27.55 (15.15-45.19) | 35.71 (20.71-58.71) | 0.63 (0.22,1.04) |
| Spain | 112.35 (75.72-159.37) | 249.17 (159.11-372.90) | 0.58 (0.39-0.82) | 1.09 (0.70-1.64) | 1.65 (1.30,2.00) |  | 109.55 (72.83-157.54) | 209.27 (132.58-314.82) | 0.56 (0.38-0.81) | 0.92 (0.58-1.38) | 1.14 (0.86,1.42) |  | 3187.86 (2231.78-4368.87) | 5445.64 (3451.91-8144.93) | 16.44 (11.51-22.53) | 23.91 (15.16-35.76) | 0.87 (0.63,1.11) |
| Sri Lanka | 48.54 (36.33-62.55) | 73.25 (42.79-117.72) | 0.57 (0.42-0.73) | 0.66 (0.38-1.06) | -0.07 (-0.46,0.33) |  | 49.28 (36.78-63.43) | 71.08 (41.36-114.10) | 0.58 (0.43-0.74) | 0.64 (0.37-1.02) | -0.09 (-0.49,0.30) |  | 1604.09 (1227.79-2019.82) | 2101.17 (1248.36-3328.72) | 18.73 (14.33-23.58) | 18.87 (11.21-29.89) | -0.45 (-0.88,-0.02) |
| Sudan | 53.81 (24.60-97.87) | 118.47 (69.44-181.68) | 0.54 (0.25-0.98) | 0.55 (0.32-0.84) | 0.12 (-0.17,0.41) |  | 54.73 (24.80-99.69) | 118.82 (69.81-180.18) | 0.55 (0.25-1.00) | 0.55 (0.32-0.83) | 0.05 (-0.24,0.34) |  | 1871.81 (894.46-3384.48) | 4103.18 (2388.08-6400.58) | 18.70 (8.94-33.81) | 18.90 (11.00-29.48) | 0.11 (-0.17,0.39) |
| Suriname | 0.80 (0.55-1.12) | 2.16 (1.33-3.23) | 0.41 (0.28-0.58) | 0.75 (0.46-1.12) | 1.73 (1.60,1.87) |  | 0.81 (0.56-1.13) | 2.19 (1.36-3.28) | 0.42 (0.29-0.59) | 0.76 (0.47-1.13) | 1.50 (1.27,1.73) |  | 26.97 (18.91-37.29) | 69.30 (43.48-103.08) | 13.95 (9.78-19.28) | 23.93 (15.01-35.59) | 1.33 (1.10,1.55) |
| Sweden | 13.59 (10.00-18.30) | 20.60 (14.95-28.83) | 0.32 (0.23-0.43) | 0.40 (0.29-0.56) | 1.42 (0.57,2.28) |  | 14.21 (10.47-19.20) | 20.80 (15.05-29.03) | 0.33 (0.24-0.45) | 0.40 (0.29-0.56) | 1.61 (0.76,2.48) |  | 382.86 (282.67-508.34) | 516.70 (375.23-712.61) | 8.92 (6.58-11.84) | 9.96 (7.23-13.74) | 1.43 (0.57,2.30) |
| Switzerland | 30.87 (20.67-43.17) | 53.46 (34.92-77.56) | 0.90 (0.60-1.26) | 1.20 (0.78-1.74) | 1.07 (0.85,1.30) |  | 28.07 (18.71-39.95) | 45.31 (28.94-66.05) | 0.82 (0.54-1.16) | 1.02 (0.65-1.48) | 0.90 (0.75,1.06) |  | 790.43 (540.46-1091.36) | 1078.59 (707.15-1562.65) | 23.02 (15.74-31.79) | 24.18 (15.85-35.03) | 0.32 (0.13,0.51) |
| Syrian Arab Republic | 67.55 (44.34-99.87) | 111.14 (67.33-171.51) | 1.06 (0.70-1.57) | 1.58 (0.96-2.44) | 0.76 (-0.09,1.63) |  | 68.29 (44.52-100.41) | 109.88 (66.59-169.17) | 1.07 (0.70-1.58) | 1.57 (0.95-2.41) | 0.66 (-0.24,1.56) |  | 2331.16 (1543.27-3360.23) | 3368.07 (2080.81-5213.01) | 36.66 (24.27-52.84) | 48.01 (29.66-74.31) | 0.33 (-0.53,1.19) |
| Taiwan (Province of China) | 394.06 (337.67-455.17) | 1063.82 (840.95-1325.34) | 3.87 (3.31-4.46) | 9.00 (7.12-11.21) | 3.83 (3.18,4.47) |  | 374.73 (319.03-435.58) | 914.09 (718.26-1146.07) | 3.68 (3.13-4.27) | 7.73 (6.08-9.70) | 3.55 (2.79,4.32) |  | 13735.37 (12015.87-15516.40) | 26176.00 (21057.17-32156.32) | 134.72 (117.86-152.19) | 221.49 (178.17-272.09) | 2.65 (1.90,3.41) |
| Tajikistan | 15.55 (9.31-25.35) | 24.78 (13.36-42.17) | 0.58 (0.35-0.94) | 0.49 (0.26-0.83) | -0.94 (-1.09,-0.79) |  | 15.68 (9.44-25.52) | 24.80 (13.34-42.22) | 0.58 (0.35-0.95) | 0.49 (0.26-0.83) | -0.91 (-1.08,-0.75) |  | 574.98 (343.26-908.33) | 890.66 (494.97-1459.39) | 21.42 (12.79-33.84) | 17.53 (9.74-28.73) | -1.14 (-1.34,-0.93) |
| Thailand | 1302.35 (960.82-1767.81) | 2279.65 (1508.60-3272.27) | 4.59 (3.39-6.23) | 6.84 (4.52-9.81) | 0.63 (0.35,0.92) |  | 1298.29 (959.70-1766.49) | 2180.46 (1428.85-3167.83) | 4.57 (3.38-6.22) | 6.54 (4.29-9.50) | 0.54 (0.29,0.80) |  | 44502.33 (33493.72-60598.08) | 67251.94 (44888.36-95897.84) | 156.80 (118.01-213.51) | 201.71 (134.63-287.62) | 0.17 (-0.14,0.47) |
| Timor-Leste | 2.49 (1.53-4.01) | 4.99 (2.85-9.01) | 0.64 (0.39-1.03) | 0.71 (0.41-1.29) | 0.57 (0.44,0.71) |  | 2.47 (1.54-3.99) | 5.06 (2.89-9.10) | 0.63 (0.40-1.02) | 0.72 (0.41-1.30) | 0.67 (0.51,0.82) |  | 91.52 (56.37-148.62) | 161.58 (93.13-280.14) | 23.43 (14.43-38.05) | 23.12 (13.33-40.08) | 0.07 (-0.12,0.26) |
| Togo | 33.58 (23.53-48.79) | 76.66 (46.00-129.60) | 1.84 (1.29-2.68) | 1.83 (1.10-3.10) | -0.41 (-0.61,-0.21) |  | 33.64 (23.56-48.77) | 76.06 (45.76-130.95) | 1.85 (1.29-2.68) | 1.82 (1.09-3.13) | -0.40 (-0.61,-0.19) |  | 1226.48 (862.78-1788.96) | 2800.04 (1702.60-4745.49) | 67.27 (47.32-98.11) | 66.90 (40.68-113.38) | -0.37 (-0.61,-0.14) |
| Tokelau | 0.01 (0.01-0.02) | 0.02 (0.01-0.03) | 1.53 (0.88-2.74) | 2.57 (1.53-4.13) | 1.65 (1.43,1.87) |  | 0.01 (0.01-0.02) | 0.02 (0.01-0.03) | 1.58 (0.91-2.83) | 2.57 (1.51-4.15) | 1.55 (1.32,1.79) |  | 0.38 (0.22-0.69) | 0.55 (0.33-0.88) | 48.04 (27.73-86.16) | 79.70 (48.12-128.03) | 1.70 (1.49,1.91) |
| Tonga | 3.44 (2.32-4.97) | 4.51 (2.96-6.63) | 6.96 (4.70-10.05) | 8.49 (5.57-12.47) | 0.26 (0.02,0.50) |  | 3.46 (2.34-4.97) | 4.47 (2.93-6.42) | 6.99 (4.74-10.05) | 8.41 (5.51-12.08) | 0.27 (0.01,0.52) |  | 114.34 (78.24-165.39) | 146.82 (96.48-217.58) | 231.33 (158.30-334.63) | 276.24 (181.52-409.36) | 0.31 (0.06,0.56) |
| Trinidad and Tobago | 2.52 (1.84-3.32) | 4.72 (3.11-7.01) | 0.42 (0.31-0.55) | 0.68 (0.45-1.01) | 1.22 (1.05,1.39) |  | 2.58 (1.89-3.42) | 4.78 (3.12-7.11) | 0.43 (0.31-0.57) | 0.69 (0.45-1.02) | 1.07 (0.80,1.34) |  | 82.67 (61.85-106.13) | 145.77 (97.21-214.81) | 13.72 (10.27-17.62) | 20.93 (13.96-30.84) | 0.92 (0.68,1.17) |
| Tunisia | 12.62 (8.62-17.86) | 35.90 (21.70-54.77) | 0.30 (0.21-0.43) | 0.61 (0.37-0.93) | 1.94 (1.70,2.17) |  | 12.78 (8.74-18.05) | 35.18 (21.11-55.01) | 0.31 (0.21-0.43) | 0.59 (0.36-0.93) | 1.81 (1.58,2.04) |  | 414.17 (288.49-568.05) | 1076.99 (662.49-1622.40) | 9.92 (6.91-13.61) | 18.19 (11.19-27.40) | 1.66 (1.44,1.87) |
| Turkey | 257.39 (189.92-335.73) | 572.96 (409.05-789.65) | 0.90 (0.66-1.17) | 1.37 (0.98-1.89) | 1.43 (1.14,1.73) |  | 263.26 (193.25-343.77) | 572.79 (410.60-789.82) | 0.92 (0.67-1.20) | 1.37 (0.98-1.89) | 1.33 (1.01,1.65) |  | 8210.49 (6168.08-10618.83) | 16119.08 (11456.14-21850.15) | 28.57 (21.46-36.95) | 38.56 (27.40-52.27) | 0.92 (0.61,1.23) |
| Turkmenistan | 16.50 (11.72-22.06) | 28.37 (17.77-43.71) | 0.89 (0.63-1.19) | 1.10 (0.69-1.69) | 1.25 (1.01,1.49) |  | 16.58 (11.74-22.42) | 28.26 (17.68-43.85) | 0.90 (0.63-1.21) | 1.10 (0.69-1.70) | 1.05 (0.71,1.39) |  | 590.64 (437.34-781.29) | 981.13 (636.51-1490.09) | 31.93 (23.64-42.24) | 38.04 (24.68-57.77) | 0.99 (0.70,1.28) |
| Tuvalu | 0.08 (0.05-0.13) | 0.13 (0.08-0.20) | 1.77 (1.15-2.77) | 2.08 (1.30-3.17) | 0.34 (0.19,0.50) |  | 0.09 (0.06-0.13) | 0.13 (0.08-0.20) | 1.79 (1.16-2.80) | 2.07 (1.29-3.17) | 0.34 (0.18,0.51) |  | 2.86 (1.88-4.36) | 4.29 (2.68-6.57) | 60.12 (39.47-91.73) | 69.35 (43.40-106.28) | 0.38 (0.22,0.55) |
| Uganda | 90.03 (58.59-132.64) | 174.10 (107.54-260.37) | 1.04 (0.68-1.53) | 0.80 (0.50-1.20) | -1.52 (-1.77,-1.27) |  | 90.72 (58.51-133.18) | 172.55 (106.17-260.32) | 1.05 (0.68-1.54) | 0.80 (0.49-1.20) | -1.54 (-1.79,-1.29) |  | 3230.03 (2146.10-4797.45) | 6805.18 (4344.01-10106.55) | 37.36 (24.82-55.49) | 31.42 (20.06-46.67) | -1.22 (-1.50,-0.93) |
| Ukraine | 158.17 (127.61-198.30) | 124.23 (86.61-172.48) | 0.60 (0.48-0.75) | 0.58 (0.40-0.80) | -1.47 (-2.18,-0.76) |  | 158.07 (127.11-198.66) | 122.16 (85.83-169.82) | 0.60 (0.48-0.75) | 0.57 (0.40-0.79) | -1.23 (-1.98,-0.48) |  | 5075.25 (4097.41-6244.93) | 3803.87 (2668.49-5262.83) | 19.26 (15.55-23.69) | 17.66 (12.39-24.43) | -1.35 (-2.11,-0.59) |
| United Arab Emirates | 9.13 (6.05-12.83) | 97.24 (65.84-144.05) | 0.98 (0.65-1.37) | 2.02 (1.37-2.99) | 1.29 (0.74,1.85) |  | 8.92 (5.94-12.44) | 91.67 (61.78-134.91) | 0.95 (0.64-1.33) | 1.90 (1.28-2.80) | 1.15 (0.60,1.70) |  | 337.99 (228.43-471.22) | 3389.45 (2297.76-4975.91) | 36.13 (24.42-50.37) | 70.39 (47.72-103.33) | 1.25 (0.80,1.70) |
| United Kingdom | 125.12 (102.51-150.66) | 536.74 (439.97-645.87) | 0.44 (0.36-0.53) | 1.58 (1.30-1.90) | 5.18 (4.92,5.45) |  | 113.33 (91.92-138.11) | 430.03 (348.06-524.98) | 0.40 (0.32-0.48) | 1.27 (1.03-1.55) | 4.63 (4.36,4.90) |  | 3093.84 (2541.87-3704.43) | 10797.29 (8894.00-12966.89) | 10.80 (8.87-12.93) | 31.83 (26.22-38.22) | 4.36 (4.10,4.63) |
| United Republic of Tanzania | 108.61 (69.59-159.83) | 180.96 (111.69-293.90) | 0.84 (0.54-1.24) | 0.62 (0.38-1.01) | -1.57 (-1.73,-1.40) |  | 109.32 (69.70-163.53) | 180.78 (111.54-291.22) | 0.85 (0.54-1.27) | 0.62 (0.38-1.00) | -1.59 (-1.76,-1.43) |  | 3941.19 (2541.98-5788.59) | 6705.67 (4174.12-10777.63) | 30.51 (19.68-44.81) | 22.95 (14.28-36.88) | -1.49 (-1.65,-1.32) |
| United States of America | 498.01 (434.31-567.46) | 1784.20 (1486.14-2089.35) | 0.39 (0.34-0.45) | 1.07 (0.89-1.26) | 3.33 (3.08,3.59) |  | 407.23 (351.80-466.97) | 1321.48 (1092.80-1553.31) | 0.32 (0.28-0.37) | 0.79 (0.66-0.93) | 3.08 (2.92,3.25) |  | 11899.16 (10453.10-13507.48) | 35277.33 (29539.22-41609.22) | 9.37 (8.23-10.63) | 21.21 (17.76-25.02) | 2.78 (2.56,3.00) |
| United States Virgin Islands | 0.21 (0.13-0.32) | 0.50 (0.27-0.82) | 0.40 (0.25-0.60) | 1.16 (0.64-1.92) | 3.57 (3.28,3.86) |  | 0.22 (0.14-0.32) | 0.51 (0.28-0.84) | 0.41 (0.26-0.60) | 1.19 (0.65-1.95) | 3.67 (3.39,3.95) |  | 7.07 (4.59-10.53) | 13.76 (7.73-22.35) | 13.32 (8.66-19.87) | 32.03 (18.00-52.03) | 2.93 (2.69,3.18) |
| Uruguay | 3.94 (2.56-5.65) | 11.55 (7.57-16.74) | 0.25 (0.16-0.36) | 0.68 (0.44-0.98) | 3.64 (3.41,3.87) |  | 4.03 (2.60-5.81) | 11.67 (7.66-16.74) | 0.26 (0.17-0.37) | 0.69 (0.45-0.98) | 3.64 (3.36,3.93) |  | 115.39 (76.33-158.60) | 312.07 (209.33-448.81) | 7.35 (4.86-10.10) | 18.33 (12.29-26.36) | 3.41 (3.13,3.70) |
| Uzbekistan | 57.91 (37.75-85.30) | 152.84 (95.87-227.93) | 0.55 (0.36-0.81) | 0.89 (0.56-1.33) | 1.35 (1.22,1.49) |  | 57.91 (37.78-85.48) | 152.30 (95.01-229.07) | 0.55 (0.36-0.82) | 0.89 (0.56-1.34) | 1.41 (1.26,1.55) |  | 2103.24 (1412.47-3050.53) | 5302.77 (3369.51-7904.33) | 20.07 (13.48-29.11) | 30.98 (19.69-46.18) | 1.32 (1.16,1.47) |
| Vanuatu | 0.92 (0.55-1.72) | 2.14 (1.32-3.50) | 1.20 (0.72-2.26) | 1.37 (0.84-2.24) | 0.51 (0.37,0.66) |  | 0.91 (0.55-1.74) | 2.13 (1.31-3.49) | 1.20 (0.72-2.29) | 1.36 (0.84-2.23) | 0.51 (0.34,0.68) |  | 32.58 (19.16-60.65) | 74.81 (45.95-123.20) | 42.81 (25.17-79.68) | 47.81 (29.37-78.73) | 0.43 (0.27,0.59) |
| Venezuela (Bolivarian Republic of) | 42.78 (30.08-59.48) | 56.73 (35.93-83.19) | 0.45 (0.32-0.63) | 0.43 (0.27-0.62) | -0.06 (-1.07,0.96) |  | 43.63 (30.73-60.83) | 57.21 (36.28-84.52) | 0.46 (0.33-0.65) | 0.43 (0.27-0.63) | -0.21 (-1.42,1.01) |  | 1413.77 (1009.78-1922.64) | 1850.67 (1185.68-2682.71) | 15.03 (10.74-20.45) | 13.90 (8.91-20.15) | -0.18 (-1.27,0.92) |
| Viet Nam | 1484.03 (968.28-2075.70) | 2907.64 (1910.35-4440.25) | 4.35 (2.84-6.08) | 5.80 (3.81-8.86) | 1.08 (0.98,1.17) |  | 1496.43 (972.77-2073.41) | 2778.17 (1803.68-4247.06) | 4.39 (2.85-6.08) | 5.54 (3.60-8.47) | 0.90 (0.81,0.98) |  | 49664.24 (32998.88-67863.80) | 91735.40 (59904.40-141380.16) | 145.59 (96.73-198.94) | 182.98 (119.49-282.00) | 0.92 (0.83,1.02) |
| Yemen | 28.14 (10.29-56.23) | 49.28 (25.76-90.00) | 0.41 (0.15-0.82) | 0.29 (0.15-0.54) | -1.36 (-1.54,-1.18) |  | 28.29 (10.34-56.79) | 49.41 (25.91-90.63) | 0.41 (0.15-0.83) | 0.29 (0.15-0.54) | -1.39 (-1.56,-1.22) |  | 988.25 (363.29-1992.17) | 1713.99 (907.27-3201.77) | 14.50 (5.33-29.22) | 10.19 (5.39-19.03) | -1.42 (-1.59,-1.25) |
| Zambia | 50.61 (33.22-73.23) | 54.20 (20.39-137.64) | 1.28 (0.84-1.85) | 0.56 (0.21-1.41) | -4.37 (-5.07,-3.67) |  | 50.58 (33.05-73.56) | 54.06 (20.61-136.14) | 1.27 (0.83-1.85) | 0.55 (0.21-1.40) | -4.39 (-5.11,-3.66) |  | 1904.52 (1268.70-2737.90) | 2070.38 (744.32-5427.60) | 47.99 (31.97-69.00) | 21.22 (7.63-55.62) | -4.31 (-5.05,-3.57) |
| Zimbabwe | 98.04 (54.53-155.44) | 186.21 (111.51-287.87) | 1.90 (1.05-3.01) | 2.39 (1.43-3.69) | -0.43 (-1.16,0.31) |  | 98.82 (55.19-157.43) | 184.48 (109.87-287.18) | 1.91 (1.07-3.04) | 2.37 (1.41-3.68) | -0.41 (-1.18,0.36) |  | 3420.09 (1886.51-5454.58) | 7027.82 (4135.81-10788.80) | 66.13 (36.48-105.48) | 90.13 (53.04-138.36) | -0.02 (-0.84,0.80) |

**Abbreviations:** UI, uncertainty interval; ASR, age-standardised rate per 100,000; EAPC, estimated annual percentage change; CI, confidence interval; DALYs, disability-adjusted life-year; SDI, socio-demographic index.
