# Supplementary material for: Burden of liver cancer from 1990 to 2021 and modelled projection to 2040: insights from the global burden of disease study 2021
Source: Front Oncol. 2026 Apr 29;16:1699519. doi: 10.3389/fonc.2026.1699519 (PMC13167583; doi:10.3389/fonc.2026.1699519)
Supplement: Supplementary file 3 [file DataSheet3.docx]

**Supplementary Material**

Burden of liver cancer from 1990 to 2021 and modelled projection to 2040: insights from the Global Burden of Disease Study 2021

Yong Xie, Tianshi Lyu, Haitao Guan, Li Song, Xiaoqiang Tong, Yinghua Zou, Min Yang* and Jian Wang*

**Affiliation:** Department of Interventional Radiology and Vascular Surgery, Peking University First Hospital, Beijing 100034, P.R. China.

***Correspondence:**

Min Yang, M.D., Department of Interventional Radiology and Vascular Surgery, Peking University First Hospital, No. 8 Xishiku Street, Xicheng District, Beijing 100034, P.R. China. E-mail addresses: [dryangmin@gmail.com](mailto:dryangmin@gmail.com).

Jian Wang, M.D, Department of Interventional Radiology and Vascular Surgery, Peking University First Hospital, No. 8 Xishiku Street, Xicheng District, Beijing 100034, P.R. China. E-mail addresses: [jianwang0987@sina.com](mailto:jianwang0987@sina.com).

**Methods**

**Table 1.** Global burden of liver cancer due to hepatitis C in 1990 and 2021, categorized by sex, region, and SDI

**Table 2.** Global burden of liver cancer due to alcohol use in 1990 and 2021, categorized by sex, region, and SDI

**Table 3.** Global burden of hepatoblastoma in 1990 and 2021, categorized by sex, region, and SDI

**Table 4.** Global burden of liver cancer due to other causes in 1990 and 2021, categorized by sex, region, and SDI

**Table 5.** Global burden of liver cancer due to non-alcoholic steatohepatitis (NASH) by age group in 1990, 2021 and estimated annual percentage change (EAPC) from 1990 to 2021.

**Table 6.** Global burden of liver cancer due to hepatitis B by age group in 1990, 2021 and estimated annual percentage change (EAPC) from 1990 to 2021.

**Table 7.** Global burden of liver cancer due to hepatitis C by age group in 1990, 2021 and estimated annual percentage change (EAPC) from 1990 to 2021.

**Table 8.** Global burden of liver cancer due to alcohol use by age group in 1990, 2021 and estimated annual percentage change (EAPC) from 1990 to 2021.

**Table 9.** Global burden of hepatoblastoma by age group in 1990, 2021 and estimated annual percentage changes (EAPC) from 1990 to 2021

**Table 10.** Global burden of liver cancer due to other causes by age group in 1990, 2021 and estimated annual percentage change (EAPC) from 1990 to 2021.

**Table 11.** Burden of liver cancer due to hepatitis C in 204 countries and territories in 1990, 2021 and the estimated annual percentage changes (EAPC) from 1990 to 2021

**Table 12.** Burden of liver cancer due to alcohol use in 204 countries and territories in 1990, 2021 and the estimated annual percentage changes (EAPC) from 1990 to 2021

**Table 13.** Burden of hepatoblastoma in 204 countries and territories in 1990, 2021 and the estimated annual percentage changes (EAPC) from 1990 to 2021

**Table 14.** Burden of liver cancer due to other causes in 204 countries and territories in 1990, 2021 and the estimated annual percentage changes (EAPC) from 1990 to 2021

**Figure 1.** Etiology-specific percentages and ranking heatmap of liver cancer incidence, mortality, and disability-adjusted life years (DALYs) in 2021

**Figure 2.** Age-specific number and percentages of liver cancer incidence, mortality, and disability-adjusted life years (DALYs) across six etiology in 2021

**Figure 3.** Epidemiologic trends in liver cancer incidence, mortality, and disability-adjusted life years (DALYs) rates across six etiology from 1990 to 2021

**Figure 4.** Global maps of rates and estimated annual percentage change (EAPC) of incidence, deaths, and disability-adjusted life-years (DALYs) attributable to liver cancer due to non-alcoholic steatohepatitis (NASH) in 2021

**Figure 5.** Global maps of rates and estimated annual percentage change (EAPC) of incidence, deaths, and disability-adjusted life-years (DALYs) attributable to liver cancer due to hepatitis B in 2021

**Figure 6.** Global maps of rates and estimated annual percentage change (EAPC) of incidence, deaths, and disability-adjusted life-years (DALYs) attributable to liver cancer due to hepatitis C in 2021

**Figure 7.** Global maps of rates and estimated annual percentage change (EAPC) of incidence, deaths, and disability-adjusted life-years (DALYs) attributable to liver cancer due to alcohol use in 2021

**Figure 8.** Global maps of rates and estimated annual percentage change (EAPC) of incidence, deaths, and disability-adjusted life-years (DALYs) attributable to hepatoblastoma in 2021

**Figure 9.** Global maps of rates and estimated annual percentage change (EAPC) of incidence, deaths, and disability-adjusted life-years (DALYs) attributable to liver cancer due to other causes in 2021

**Figure 10.** Inequality analysis of DALYs attributable to liver cancer, 1990-2021

**Figure 11.** Projected burden of liver cancer due to non-alcoholic steatohepatitis (NASH) by 2040

**Figure 12.** Projected burden of liver cancer due to hepatitis B by 2040

**Figure 13.** Projected burden of liver cancer due to hepatitis C by 2040

**Figure 14.** Projected burden of liver cancer due to alcohol use by 2040

**Figure 15.** Projected burden of hepatoblastoma by 2040

**Figure 16.** Projected burden of liver cancer due to other causes by 2040

**Methods**

**1.Temporal trends analysis**

Temporal trends in age-standardized rates (ASR) were quantified using estimated annual percentage changes (EAPC), which were derived from a log-linear regression model: Y = αX + β. Here, Y denotes log₁₀-transformed ASR values and X corresponds to the calendar year. The EAPC was calculated using the formula: 100 × (10^α−1). A declining trend is indicated when the EAPC value is less than 0 and the upper bound of its 95% confidence intervals (CIs) also falls below 0. In contrast, an increasing trend is signified when the EAPC value exceeds 0 and the lower bound of its 95% CIs lies above 0 [1,2].

**2. Cross-country inequality analysis**

In this study, the slope index of inequality (SII) and concentration index (CI), as defined by the World Health Organization (WHO), were employed to measure the absolute and relative inequalities in the global burden of skin cancer across countries [3,4]. The slope index of inequality is calculated by regressing the disability-adjusted life-years (DALYs) rate on the sociodemographic index (SDI), using the midpoint of the cumulative population distribution sorted by SDI [5,6]. To analyze temporal changes in health inequality, we compared data from 204 countries and territories spanning the period from 1990 to 2021. To enhance bias control and account for heterogeneity, a robust weighted regression model (rlm) was utilized in the health inequality analysis instead of the ordinary linear regression model (lm). This robust regression approach reduces sensitivity to outliers, thereby minimizing bias arising from data heterogeneity or extreme values and enabling a more accurate characterization of health inequality patterns. Additionally, the concentration index is computed through numerical integration of the area under the Lorenz curve, which graphs the cumulative proportion of DALYs against the cumulative proportion of the population [7,8]. The index ranges from -1 to 1, with interpretations as follows: a value of 0 indicates perfect equality, meaning DALYs are evenly distributed across the population; positive values signify that DALYs are disproportionately concentrated among higher-SDI groups; and negative values indicate a concentration of DALYs among lower-SDI groups.

**3. The autoregressive integrated moving average (ARIMA) model projection**

To forecast the future burden of liver cancer from 2022 to 2040, time-series modeling techniques were applied using historical ASR and numerical data. Specifically, ARIMA models were utilized to capture short-term fluctuations, while exponential smoothing models were employed for long-term trend estimation. Model selection was guided by standard diagnostic criteria, including the Akaike Information Criterion, Bayesian Information Criterion, residual autocorrelation checks, and visual assessment of fitted versus actual value plots. Goodness-of-fit was evaluated using the mean absolute percentage error and root mean square error. These models were implemented in adherence to established guidelines for time-series forecasting in global epidemiological research. In other words, this study used data from 1990 to 2021 and applied the ARIMA model to predict the trend of liver cancer burden over the next 19 years. Incidence was modeled using ARIMA(0,2,0), while mortality and DALYs were modeled using ARIMA(2,1,0). The models fit well, with small errors and residuals conforming to white noise, validating that the predictions are robust and reliable. Model selection was automated via auto.arima() (forecast v8.21.1). Residual analysis includes the residual mean being close to 0, the smaller the residual standard deviation the better, the maximum autocorrelation function (ACF) value <0.2, and the maximum partial autocorrelation function (PACF) value <0.2. Prediction quality includes a moderately wide average confidence interval and prediction stability (coefficient of variation) <10%. Model selection criteria include the smaller the akaike information criterion (AIC) the better, the smaller the bayesian information criterion (BIC) the better, and the larger the log-likelihood value the better. All projections are reported with 95% uncertainty intervals (UIs), which were derived from bootstrapped simulations to account for estimation variability [9,10]. The ARIMA model projection has been widely validated in previous studies [9-12].

**References**

1. Yi M, Li A, Zhou L, Chu Q, Song Y, Wu K. The global burden and attributable risk factor analysis of acute myeloid leukemia in 195 countries and territories from 1990 to 2017: estimates based on the global burden of disease study 2017. J Hematol Oncol. 2020;13(1):72. doi: 10.1186/s13045-020-00908-z.
2. Bai X, Yi M, Dong B, Zheng X, Wu K. The global, regional, and national burden of kidney cancer and attributable risk factor analysis from 1990 to 2017. Exp Hematol Oncol. 2020;9:27. doi: 10.1186/s40164-020-00181-3.
3. Wagstaff A, Paci P, van Doorslaer E. On the measurement of inequalities in health. Soc Sci Med. 1991;33(5):545-57. doi:10.1016/0277-9536(91)90212-u
4. Ordunez P, Martinez R, Soliz P, Giraldo G, Mujica OJ, Nordet P. Rheumatic heart disease burden, trends, and inequalities in the Americas, 1990-2017: a population-based study. Lancet Glob Health. 2019;7(10):e1388-e1397. doi:10.1016/s2214-109x(19)30360-2.
5. Cao F, He YS, Wang Y, Zha CK, Lu JM, Tao LM, et al. Global burden and cross-country inequalities in autoimmune diseases from 1990 to 2019. Autoimmun Rev. 2023;22(6):103326. doi:10.1016/j.autrev.2023.103326.
6. Chen J, Cui Y, Deng Y, Xiang Y, Chen J, Wang Y, et al. Global, regional, and national burden of cancers attributable to particulate matter pollution from 1990 to 2019 and projection to 2050: Worsening or improving? J Hazard Mater. 2024;477:135319. doi:10.1016/j.jhazmat.2024.135319.
7. Erreygers G, Clarke P, Van Ourti T. "Mirror, mirror, on the wall, who in this land is fairest of all?"--Distributional sensitivity in the measurement of socioeconomic inequality of health. J Health Econ. 2012;31(1):257-70. doi:10.1016/j.jhealeco.2011.10.009.
8. Chen Z, Roy K. Calculating concentration index with repetitive values of indicators of economic welfare. J Health Econ. 2009;28(1):169-75. doi:10.1016/j.jhealeco.2008.09.004.
9. Ye J, Chen J, Ding H, Xia Z, Wang J, Jia Y. The global burden of low back pain attributable high body mass index over the period 1990-2021 and projections up to 2035. Front Nutr. 2025;12:1568015. doi: 10.3389/fnut.2025.1568015.
10. Guo J, Wang P, Gong J, Sun W, Han X, Xu C, et al. The disease burden, risk factors and future predictions of Alzheimer's disease and other types of dementia in Asia from 1990 to 2021. J Prev Alzheimers Dis. 2025;12(5):100122. doi: 10.1016/j.tjpad.2025.100122.
11. Li Y, Ning Y, Shen B, Shi Y, Song N, Fang Y, et al. Temporal trends in prevalence and mortality for chronic kidney disease in China from 1990 to 2019: an analysis of the Global Burden of Disease Study 2019. Clin Kidney J. 2022;16(2):312-321. doi: 10.1093/ckj/sfac218.
12. Duo T, Wen Y, Bian Y, Wang Y, Zhang X, Ju J, et al. Rising burden of MASLD and CKM syndrome in Asia: A decade of trends and future projections. Metabolism. 2026;178:156549. doi: 10.1016/j.metabol.2026.156549.

**Table 1. Global burden of liver cancer due to hepatitis C in 1990 and 2021, categorized by sex, region, and SDI**

| **Characteristics** | **Incidence** | | | | |  | **Deaths** | | | | |  | **DALYs** | | | | |
| --- | --- | --- | --- | --- | --- | --- | --- | --- | --- | --- | --- | --- | --- | --- | --- | --- | --- |
|  | **1990 No. (95% UI)** | **2021 No. (95% UI)** | **1990 ASR per 100 000 (95% UI)** | **2021 ASR per 100 000 (95% UI)** | **EAPC (95% CI)** |  | **1990 No. (95% UI)** | **2021 No. (95% UI)** | **1990 ASR per 100 000 (95% UI)** | **2021 ASR per 100 000 (95% UI)** | **EAPC (95% CI)** |  | **1990 No. (95% UI)** | **2021 No. (95% UI)** | **1990 ASR per 100 000 (95% UI)** | **2021 ASR per 100 000 (95% UI)** | **EAPC (95% CI)** |
| **Global** | 32186.46 (27835.20-37695.90) | 77030.78 (65958.04-88627.55) | 1.21 (1.04-1.41) | 1.95 (1.67-2.25) | 1.34 (1.21,1.47) |  | 32064.99 (27648.33-37762.30) | 73261.01 (62968.07-84259.47) | 1.20 (1.04-1.42) | 1.86 (1.60-2.14) | 1.26 (1.15,1.38) |  | 786102.80 (673665.90-935257.38) | 1549435.20 (1331149.20-1804540.78) | 29.48 (25.26-35.07) | 39.27 (33.74-45.73) | 0.77 (0.66,0.88) |
| **Sex** |  |  |  |  |  |  |  |  |  |  |  |  |  |  |  |  |  |
| Male | 17154.81 (14867.06-20005.27) | 40854.74 (34564.01-48021.92) | 1.28 (1.11-1.49) | 2.06 (1.75-2.43) | 1.22 (1.06,1.39) |  | 16466.85 (14232.36-19277.55) | 37448.12 (31517.19-44173.28) | 1.23 (1.06-1.44) | 1.89 (1.59-2.23) | 1.15 (0.98,1.31) |  | 427923.22 (364772.08-507796.58) | 826898.71 (685222.78-989794.65) | 31.87 (27.16-37.81) | 41.77 (34.61-50.00) | 0.62 (0.47,0.77) |
| Female | 15031.65 (12814.15-17945.39) | 36176.04 (30639.89-41419.76) | 1.14 (0.97-1.36) | 1.84 (1.56-2.11) | 1.47 (1.38,1.56) |  | 15598.14 (13185.99-18726.81) | 35812.89 (30214.37-41042.51) | 1.18 (1.00-1.41) | 1.82 (1.54-2.09) | 1.39 (1.32,1.46) |  | 358179.58 (301616.71-433878.74) | 722536.50 (624631.81-830654.74) | 27.05 (22.78-32.77) | 36.75 (31.77-42.25) | 0.94 (0.88,1.01) |
| **SDI category** |  |  |  |  |  |  |  |  |  |  |  |  |  |  |  |  |  |
| Low SDI | 1769.57 (1210.65-2651.50) | 3350.97 (2466.16-4613.87) | 0.71 (0.48-1.06) | 0.60 (0.44-0.83) | -0.76 (-0.87,-0.66) |  | 1891.31 (1301.71-2850.33) | 3602.07 (2674.56-4970.47) | 0.75 (0.52-1.14) | 0.64 (0.48-0.89) | -0.74 (-0.84,-0.63) |  | 49135.83 (33838.11-74138.62) | 91799.43 (66640.33-128940.58) | 19.60 (13.50-29.58) | 16.43 (11.93-23.08) | -0.81 (-0.92,-0.70) |
| Low-middle SDI | 3983.16 (3055.33-5474.95) | 10245.85 (8532.45-12115.59) | 0.69 (0.53-0.94) | 1.07 (0.89-1.26) | 1.64 (1.55,1.73) |  | 4233.02 (3240.67-5804.26) | 10862.76 (9040.97-12769.69) | 0.73 (0.56-1.00) | 1.13 (0.94-1.33) | 1.59 (1.51,1.67) |  | 111582.78 (85577.71-152131.87) | 278149.11 (230765.72-331758.19) | 19.22 (14.74-26.20) | 28.96 (24.02-34.54) | 1.51 (1.43,1.59) |
| Middle SDI | 6613.68 (5510.44-7852.17) | 17945.13 (14766.93-21591.25) | 0.77 (0.64-0.91) | 1.47 (1.21-1.76) | 2.28 (2.06,2.50) |  | 7065.76 (5895.94-8369.49) | 18112.62 (14954.31-21661.14) | 0.82 (0.68-0.97) | 1.48 (1.22-1.77) | 2.08 (1.88,2.29) |  | 177067.90 (147692.40-213703.05) | 405865.68 (331183.07-493264.14) | 20.55 (17.14-24.81) | 33.15 (27.05-40.29) | 1.70 (1.51,1.89) |
| High-middle SDI | 6974.01 (5969.03-8127.23) | 14927.13 (12579.37-17551.92) | 1.31 (1.12-1.53) | 2.29 (1.93-2.69) | 1.74 (1.65,1.83) |  | 7335.51 (6282.37-8532.77) | 14685.89 (12380.37-17188.05) | 1.38 (1.18-1.60) | 2.25 (1.90-2.64) | 1.49 (1.40,1.58) |  | 176372.40 (149822.03-206241.87) | 309048.03 (260241.45-366691.93) | 33.17 (28.17-38.78) | 47.40 (39.91-56.24) | 1.02 (0.92,1.11) |
| High SDI | 12826.66 (11614.13-14183.30) | 30523.70 (25939.29-34621.11) | 2.92 (2.64-3.23) | 5.58 (4.74-6.33) | 1.51 (1.15,1.88) |  | 11518.45 (10381.36-12800.09) | 25957.77 (21986.14-29476.18) | 2.62 (2.36-2.91) | 4.75 (4.02-5.39) | 1.49 (1.13,1.86) |  | 271463.29 (244210.83-301889.47) | 463711.44 (399138.87-529013.77) | 61.73 (55.53-68.65) | 84.77 (72.97-96.71) | 0.55 (0.19,0.91) |
| **Region** |  |  |  |  |  |  |  |  |  |  |  |  |  |  |  |  |  |
| Andean Latin America | 30.65 (21.26-43.42) | 97.58 (61.60-143.81) | 0.16 (0.11-0.23) | 0.30 (0.19-0.43) | 2.13 (1.83,2.43) |  | 34.22 (23.75-48.45) | 110.06 (70.14-162.07) | 0.18 (0.13-0.26) | 0.33 (0.21-0.49) | 2.13 (1.82,2.44) |  | 754.01 (521.57-1071.64) | 2199.67 (1386.62-3312.86) | 3.97 (2.75-5.64) | 6.65 (4.19-10.02) | 1.76 (1.46,2.06) |
| Australasia | 62.23 (48.22-80.20) | 462.94 (359.03-600.06) | 0.61 (0.48-0.79) | 2.99 (2.32-3.88) | 5.11 (4.91,5.32) |  | 62.88 (48.50-81.12) | 434.19 (333.05-563.47) | 0.62 (0.48-0.80) | 2.80 (2.15-3.64) | 4.80 (4.62,4.98) |  | 1434.34 (1092.91-1849.36) | 8739.53 (6742.69-11230.30) | 14.15 (10.78-18.24) | 56.45 (43.56-72.54) | 4.48 (4.34,4.61) |
| Caribbean | 70.89 (53.13-94.15) | 128.32 (94.89-169.96) | 0.40 (0.30-0.53) | 0.54 (0.40-0.72) | 0.60 (0.32,0.88) |  | 78.46 (58.73-102.91) | 141.12 (104.18-185.94) | 0.44 (0.33-0.58) | 0.59 (0.44-0.78) | 0.61 (0.30,0.91) |  | 1700.99 (1258.72-2287.29) | 3002.07 (2202.30-4096.68) | 9.64 (7.13-12.96) | 12.65 (9.28-17.26) | 0.58 (0.28,0.88) |
| Central Asia | 637.29 (475.96-813.23) | 1095.10 (823.50-1410.03) | 1.84 (1.37-2.35) | 2.29 (1.72-2.94) | 0.63 (0.54,0.71) |  | 680.93 (517.54-870.01) | 1168.48 (878.52-1490.42) | 1.96 (1.49-2.51) | 2.44 (1.83-3.11) | 0.58 (0.48,0.68) |  | 17483.38 (12977.87-22801.89) | 29612.36 (21893.30-38173.28) | 50.45 (37.45-65.80) | 61.82 (45.70-79.69) | 0.48 (0.39,0.57) |
| Central Europe | 525.81 (390.89-694.73) | 760.30 (582.11-983.48) | 0.84 (0.62-1.11) | 1.32 (1.01-1.71) | 1.46 (1.36,1.56) |  | 578.09 (428.11-763.62) | 841.22 (648.45-1089.53) | 0.92 (0.68-1.22) | 1.46 (1.13-1.89) | 1.52 (1.38,1.66) |  | 12909.42 (9430.75-17000.61) | 16774.53 (12685.91-21861.91) | 20.64 (15.08-27.18) | 29.11 (22.01-37.93) | 1.11 (0.97,1.25) |
| Central Latin America | 499.09 (430.34-583.19) | 1589.38 (1367.72-1827.58) | 0.61 (0.52-0.71) | 1.26 (1.08-1.44) | 2.67 (2.42,2.93) |  | 546.24 (471.61-633.77) | 1750.09 (1511.44-2009.49) | 0.66 (0.57-0.77) | 1.38 (1.19-1.59) | 2.62 (2.34,2.90) |  | 12773.98 (10935.13-14967.13) | 38158.00 (32858.82-44274.57) | 15.54 (13.30-18.21) | 30.16 (25.98-35.00) | 2.39 (2.10,2.67) |
| Central Sub-Saharan Africa | 414.60 (200.16-812.20) | 692.49 (326.91-1476.80) | 1.51 (0.73-2.96) | 1.01 (0.48-2.16) | -1.75 (-1.96,-1.53) |  | 434.03 (210.49-841.71) | 729.01 (340.70-1591.91) | 1.58 (0.77-3.06) | 1.06 (0.50-2.33) | -1.72 (-1.93,-1.52) |  | 12241.48 (5738.09-24065.49) | 20355.14 (9411.20-46017.04) | 44.54 (20.88-87.57) | 29.73 (13.75-67.21) | -1.73 (-1.94,-1.51) |
| East Asia | 7511.24 (6096.42-9116.65) | 19251.83 (15149.08-23685.02) | 1.23 (1.00-1.50) | 2.61 (2.06-3.22) | 2.62 (2.49,2.76) |  | 7945.33 (6444.40-9606.84) | 18451.78 (14567.66-22567.45) | 1.31 (1.06-1.58) | 2.51 (1.98-3.06) | 2.27 (2.13,2.42) |  | 201394.27 (161984.65-244963.08) | 396635.44 (310653.42-491125.43) | 33.08 (26.61-40.24) | 53.86 (42.19-66.69) | 1.68 (1.55,1.81) |
| Eastern Europe | 654.70 (562.91-753.52) | 1143.21 (971.21-1305.01) | 0.58 (0.50-0.67) | 1.11 (0.94-1.26) | 2.24 (2.03,2.46) |  | 705.34 (607.13-808.18) | 1254.14 (1067.17-1423.67) | 0.62 (0.54-0.71) | 1.21 (1.03-1.38) | 2.37 (2.11,2.64) |  | 16705.88 (14363.87-19336.27) | 26679.81 (22515.81-30878.31) | 14.75 (12.68-17.07) | 25.81 (21.78-29.87) | 1.97 (1.71,2.24) |
| Eastern Sub-Saharan Africa | 637.93 (473.17-874.03) | 1257.40 (916.21-1699.60) | 0.67 (0.50-0.92) | 0.59 (0.43-0.80) | -0.69 (-0.79,-0.58) |  | 683.70 (504.99-930.57) | 1357.53 (988.42-1821.58) | 0.72 (0.53-0.98) | 0.64 (0.46-0.86) | -0.65 (-0.75,-0.54) |  | 17538.80 (12937.06-24556.00) | 33997.67 (24402.41-46739.85) | 18.38 (13.56-25.74) | 15.96 (11.45-21.94) | -0.74 (-0.85,-0.63) |
| High-income Asia Pacific | 8788.93 (8129.72-9506.91) | 15789.95 (13403.86-17666.51) | 10.14 (9.38-10.97) | 17.03 (14.46-19.05) | 0.81 (0.30,1.33) |  | 7459.47 (6881.76-8086.71) | 12696.87 (10618.32-14194.24) | 8.60 (7.94-9.33) | 13.69 (11.45-15.31) | 0.82 (0.26,1.38) |  | 185190.94 (170020.11-201130.63) | 199822.22 (173163.56-222059.10) | 213.62 (196.12-232.01) | 215.50 (186.75-239.49) | -0.74 (-1.31,-0.17) |
| High-income North America | 1389.64 (1242.86-1525.28) | 6412.24 (5603.23-7214.30) | 0.99 (0.88-1.08) | 3.46 (3.03-3.90) | 4.13 (3.98,4.29) |  | 1267.31 (1134.57-1395.47) | 5296.84 (4619.37-5968.17) | 0.90 (0.81-0.99) | 2.86 (2.50-3.22) | 3.82 (3.73,3.91) |  | 27838.86 (25044.62-30633.81) | 114069.38 (100196.68-128828.04) | 19.79 (17.80-21.77) | 61.63 (54.13-69.60) | 3.92 (3.77,4.07) |
| North Africa and Middle East | 1879.26 (1326.10-2891.83) | 5061.13 (3780.11-6411.81) | 1.11 (0.78-1.71) | 1.62 (1.21-2.06) | 1.57 (1.44,1.71) |  | 1997.27 (1415.72-3073.35) | 5302.44 (3984.86-6663.77) | 1.18 (0.83-1.81) | 1.70 (1.28-2.14) | 1.48 (1.36,1.60) |  | 52177.27 (36864.13-80394.96) | 135639.37 (100855.01-173920.23) | 30.77 (21.74-47.40) | 43.54 (32.38-55.83) | 1.45 (1.30,1.60) |
| Oceania | 12.37 (6.47-26.06) | 25.05 (14.43-46.53) | 0.38 (0.20-0.80) | 0.36 (0.21-0.67) | -0.37 (-0.44,-0.30) |  | 13.03 (6.86-27.14) | 26.31 (15.30-48.22) | 0.40 (0.21-0.83) | 0.38 (0.22-0.69) | -0.40 (-0.48,-0.32) |  | 350.74 (176.94-745.15) | 695.62 (387.99-1269.14) | 10.71 (5.40-22.75) | 9.99 (5.57-18.22) | -0.39 (-0.45,-0.34) |
| South Asia | 1510.50 (1253.44-1793.50) | 5205.11 (4420.10-6091.63) | 0.28 (0.23-0.33) | 0.56 (0.48-0.66) | 2.42 (2.32,2.52) |  | 1598.84 (1321.59-1889.12) | 5557.88 (4747.57-6483.50) | 0.29 (0.24-0.35) | 0.60 (0.51-0.70) | 2.43 (2.32,2.54) |  | 43042.79 (35731.57-51230.67) | 141189.08 (119408.10-165999.94) | 7.87 (6.54-9.37) | 15.29 (12.93-17.98) | 2.21 (2.13,2.29) |
| Southeast Asia | 1930.04 (1437.55-2510.91) | 4777.98 (3195.20-6644.95) | 0.83 (0.62-1.08) | 1.37 (0.92-1.90) | 1.59 (1.55,1.63) |  | 2057.26 (1543.72-2678.75) | 5018.23 (3376.55-7003.52) | 0.88 (0.66-1.15) | 1.44 (0.97-2.01) | 1.55 (1.51,1.59) |  | 53194.68 (39224.82-70430.54) | 122425.88 (80902.93-171618.03) | 22.85 (16.85-30.26) | 35.06 (23.17-49.15) | 1.36 (1.31,1.41) |
| Southern Latin America | 62.12 (46.92-81.96) | 281.87 (214.20-362.52) | 0.25 (0.19-0.33) | 0.83 (0.63-1.07) | 4.50 (4.30,4.69) |  | 67.61 (51.06-88.57) | 302.99 (230.37-387.56) | 0.27 (0.21-0.36) | 0.90 (0.68-1.15) | 4.50 (4.27,4.72) |  | 1536.09 (1142.58-2022.27) | 6397.60 (4851.59-8284.99) | 6.20 (4.61-8.16) | 18.90 (14.33-24.48) | 4.25 (4.02,4.48) |
| Southern Sub-Saharan Africa | 186.77 (118.95-283.94) | 549.73 (441.24-678.75) | 0.71 (0.45-1.08) | 1.37 (1.10-1.69) | 1.80 (1.26,2.33) |  | 202.85 (130.39-307.22) | 596.45 (481.37-735.13) | 0.77 (0.50-1.17) | 1.49 (1.20-1.83) | 1.75 (1.20,2.30) |  | 5066.35 (3229.50-7703.09) | 14640.35 (11567.88-18372.15) | 19.33 (12.32-29.39) | 36.46 (28.81-45.76) | 1.75 (1.18,2.32) |
| Tropical Latin America | 248.14 (218.20-277.18) | 810.12 (701.67-913.25) | 0.33 (0.29-0.36) | 0.71 (0.62-0.80) | 3.24 (2.94,3.53) |  | 269.73 (237.48-299.79) | 889.85 (769.28-998.60) | 0.35 (0.31-0.39) | 0.78 (0.68-0.88) | 3.34 (3.04,3.64) |  | 6503.62 (5700.37-7300.74) | 19692.05 (17066.65-22533.79) | 8.53 (7.47-9.57) | 17.31 (15.00-19.81) | 3.10 (2.80,3.40) |
| Western Europe | 4182.45 (3556.59-4879.83) | 10044.28 (8263.66-11951.15) | 2.18 (1.85-2.54) | 4.59 (3.78-5.46) | 2.41 (2.28,2.53) |  | 4351.77 (3678.81-5050.60) | 9606.72 (7830.13-11482.21) | 2.26 (1.91-2.63) | 4.39 (3.58-5.25) | 2.08 (1.96,2.21) |  | 90743.88 (77413.91-106577.61) | 176073.88 (144925.64-209849.91) | 47.21 (40.28-55.45) | 80.51 (66.27-95.96) | 1.65 (1.52,1.78) |
| Western Sub-Saharan Africa | 951.83 (562.57-1619.00) | 1594.77 (1172.96-2124.30) | 0.99 (0.58-1.68) | 0.65 (0.48-0.87) | -1.63 (-1.76,-1.51) |  | 1030.66 (611.59-1748.24) | 1728.83 (1290.94-2285.86) | 1.07 (0.63-1.81) | 0.71 (0.53-0.93) | -1.61 (-1.74,-1.49) |  | 25521.03 (15273.41-43721.74) | 42635.56 (30741.86-57714.99) | 26.42 (15.81-45.27) | 17.41 (12.55-23.57) | -1.63 (-1.77,-1.50) |

**Abbreviations:** UI, uncertainty interval; ASR, age-standardised rate per 100,000; EAPC, estimated annual percentage change; CI, confidence interval; DALYs, disability-adjusted life-year; SDI, socio-demographic index.

**Table 2. Global burden of liver cancer due to alcohol use in 1990 and 2021, categorized by sex, region, and SDI**

| **Characteristics** | **Incidence** | | | | |  | **Deaths** | | | | |  | **DALYs** | | | | |
| --- | --- | --- | --- | --- | --- | --- | --- | --- | --- | --- | --- | --- | --- | --- | --- | --- | --- |
|  | **1990 No. (95% UI)** | **2021 No. (95% UI)** | **1990 ASR per 100 000 (95% UI)** | **2021 ASR per 100 000 (95% UI)** | **EAPC (95% CI)** |  | **1990 No. (95% UI)** | **2021 No. (95% UI)** | **1990 ASR per 100 000 (95% UI)** | **2021 ASR per 100 000 (95% UI)** | **EAPC (95% CI)** |  | **1990 No. (95% UI)** | **2021 No. (95% UI)** | **1990 ASR per 100 000 (95% UI)** | **2021 ASR per 100 000 (95% UI)** | **EAPC (95% CI)** |
| **Global** | 19222.62 (15770.01-23199.47) | 49771.83 (40478.70-60200.93) | 0.72 (0.59-0.87) | 1.26 (1.03-1.53) | 1.79 (1.73,1.84) |  | 19085.81 (15584.94-23099.86) | 46113.89 (37526.56-56080.13) | 0.72 (0.58-0.87) | 1.17 (0.95-1.42) | 1.57 (1.51,1.62) |  | 521057.95 (426435.73-640271.85) | 1158013.52 (943506.47-1422894.49) | 19.54 (15.99-24.01) | 29.35 (23.91-36.06) | 1.29 (1.23,1.34) |
| **Sex** |  |  |  |  |  |  |  |  |  |  |  |  |  |  |  |  |  |
| Male | 14760.13 (12178.02-17963.04) | 38966.66 (32057.97-47081.42) | 1.10 (0.91-1.34) | 1.97 (1.62-2.38) | 1.85 (1.79,1.90) |  | 14510.20 (11958.80-17722.53) | 35633.84 (29097.47-43306.27) | 1.08 (0.89-1.32) | 1.80 (1.47-2.19) | 1.61 (1.55,1.67) |  | 402542.82 (330970.93-495523.00) | 908183.01 (738733.69-1123302.99) | 29.98 (24.65-36.90) | 45.87 (37.32-56.74) | 1.33 (1.28,1.39) |
| Female | 4462.49 (3526.20-5577.56) | 10805.17 (8563.81-13261.19) | 0.34 (0.27-0.42) | 0.55 (0.44-0.67) | 1.61 (1.54,1.68) |  | 4575.62 (3613.82-5754.22) | 10480.06 (8321.50-12883.93) | 0.35 (0.27-0.43) | 0.53 (0.42-0.66) | 1.46 (1.38,1.53) |  | 118515.13 (93957.45-147687.30) | 249830.51 (199337.23-306871.31) | 8.95 (7.10-11.16) | 12.71 (10.14-15.61) | 1.16 (1.08,1.24) |
| **SDI category** |  |  |  |  |  |  |  |  |  |  |  |  |  |  |  |  |  |
| Low SDI | 1277.76 (824.86-1871.65) | 2454.74 (1764.15-3351.77) | 0.51 (0.33-0.75) | 0.44 (0.32-0.60) | -0.76 (-0.88,-0.64) |  | 1331.85 (864.71-1942.88) | 2558.57 (1851.36-3510.22) | 0.53 (0.34-0.78) | 0.46 (0.33-0.63) | -0.75 (-0.88,-0.62) |  | 38018.85 (24462.87-54955.72) | 73035.74 (52259.18-101619.01) | 15.17 (9.76-21.93) | 13.07 (9.35-18.19) | -0.75 (-0.88,-0.62) |
| Low-middle SDI | 1906.16 (1467.83-2498.69) | 5564.40 (4445.38-7197.14) | 0.33 (0.25-0.43) | 0.58 (0.46-0.75) | 1.86 (1.72,2.00) |  | 1979.88 (1526.98-2580.79) | 5789.49 (4638.46-7485.84) | 0.34 (0.26-0.44) | 0.60 (0.48-0.78) | 1.82 (1.67,1.97) |  | 56919.08 (43410.03-75612.54) | 160378.07 (127172.84-205651.27) | 9.80 (7.48-13.02) | 16.70 (13.24-21.41) | 1.70 (1.58,1.83) |
| Middle SDI | 4725.56 (3803.68-5904.15) | 14562.60 (11442.47-18431.30) | 0.55 (0.44-0.69) | 1.19 (0.93-1.51) | 2.65 (2.52,2.78) |  | 4854.06 (3937.05-6072.88) | 14166.89 (11197.37-17815.77) | 0.56 (0.46-0.70) | 1.16 (0.91-1.46) | 2.45 (2.34,2.56) |  | 141397.79 (112966.05-178418.75) | 378602.58 (298317.65-482238.04) | 16.41 (13.11-20.71) | 30.92 (24.37-39.39) | 2.15 (2.05,2.25) |
| High-middle SDI | 4822.30 (3998.04-5738.67) | 9800.84 (7904.82-11840.52) | 0.91 (0.75-1.08) | 1.50 (1.21-1.82) | 1.60 (1.50,1.71) |  | 4901.19 (4065.29-5832.46) | 9271.89 (7554.04-11160.09) | 0.92 (0.76-1.10) | 1.42 (1.16-1.71) | 1.35 (1.25,1.45) |  | 133372.92 (110766.76-161195.57) | 230369.55 (184570.73-280415.76) | 25.08 (20.83-30.31) | 35.33 (28.31-43.01) | 1.04 (0.94,1.15) |
| High SDI | 6467.87 (5451.02-7549.53) | 17346.58 (14483.94-20478.92) | 1.47 (1.24-1.72) | 3.17 (2.65-3.74) | 2.39 (2.26,2.52) |  | 5994.78 (5035.01-7029.93) | 14283.86 (11882.60-16929.49) | 1.36 (1.14-1.60) | 2.61 (2.17-3.09) | 2.06 (1.97,2.16) |  | 150735.19 (126275.69-176463.36) | 314583.24 (262989.22-371513.59) | 34.28 (28.71-40.13) | 57.51 (48.08-67.92) | 1.63 (1.52,1.73) |
| **Region** |  |  |  |  |  |  |  |  |  |  |  |  |  |  |  |  |  |
| Andean Latin America | 62.56 (44.54-83.97) | 211.87 (148.10-299.67) | 0.33 (0.23-0.44) | 0.64 (0.45-0.91) | 2.33 (2.04,2.61) |  | 67.16 (47.88-89.96) | 228.45 (158.29-324.31) | 0.35 (0.25-0.47) | 0.69 (0.48-0.98) | 2.32 (2.03,2.62) |  | 1713.76 (1220.16-2300.42) | 5306.78 (3674.58-7459.92) | 9.02 (6.42-12.11) | 16.05 (11.11-22.56) | 1.95 (1.65,2.24) |
| Australasia | 95.72 (76.67-113.93) | 592.35 (470.60-730.91) | 0.94 (0.76-1.12) | 3.83 (3.04-4.72) | 4.80 (4.62,4.98) |  | 92.61 (74.15-111.59) | 505.47 (399.27-630.26) | 0.91 (0.73-1.10) | 3.27 (2.58-4.07) | 4.22 (4.12,4.33) |  | 2374.46 (1914.53-2833.33) | 11775.17 (9397.08-14481.21) | 23.42 (18.88-27.95) | 76.06 (60.70-93.54) | 3.99 (3.89,4.09) |
| Caribbean | 74.59 (55.02-97.85) | 153.07 (113.86-195.52) | 0.42 (0.31-0.55) | 0.65 (0.48-0.82) | 1.08 (0.78,1.39) |  | 79.42 (58.86-104.00) | 160.34 (119.18-205.56) | 0.45 (0.33-0.59) | 0.68 (0.50-0.87) | 1.03 (0.72,1.34) |  | 1978.99 (1462.37-2617.35) | 3964.04 (2938.29-5116.24) | 11.21 (8.29-14.83) | 16.71 (12.38-21.56) | 1.03 (0.73,1.32) |
| Central Asia | 430.44 (305.63-576.48) | 710.05 (493.56-971.36) | 1.24 (0.88-1.66) | 1.48 (1.03-2.03) | 0.24 (0.07,0.41) |  | 446.76 (318.01-595.84) | 737.51 (513.10-1008.15) | 1.29 (0.92-1.72) | 1.54 (1.07-2.10) | 0.20 (0.03,0.37) |  | 12703.02 (9023.47-16860.71) | 20543.86 (14309.28-28514.44) | 36.65 (26.04-48.65) | 42.89 (29.87-59.52) | 0.12 (-0.08,0.33) |
| Central Europe | 853.73 (668.09-1037.19) | 1344.37 (1089.01-1577.81) | 1.36 (1.07-1.66) | 2.33 (1.89-2.74) | 1.80 (1.64,1.97) |  | 906.19 (710.82-1101.18) | 1421.33 (1151.19-1670.28) | 1.45 (1.14-1.76) | 2.47 (2.00-2.90) | 1.81 (1.61,2.01) |  | 22766.33 (17880.16-28085.95) | 32734.98 (26765.47-38727.75) | 36.40 (28.59-44.90) | 56.80 (46.44-67.20) | 1.52 (1.32,1.72) |
| Central Latin America | 292.08 (233.47-355.82) | 926.00 (740.86-1129.15) | 0.36 (0.28-0.43) | 0.73 (0.59-0.89) | 2.50 (2.04,2.95) |  | 310.33 (247.09-377.38) | 982.42 (785.34-1203.98) | 0.38 (0.30-0.46) | 0.78 (0.62-0.95) | 2.38 (1.88,2.89) |  | 8065.88 (6462.90-9860.83) | 24120.13 (19266.16-29701.17) | 9.81 (7.86-12.00) | 19.07 (15.23-23.48) | 2.18 (1.68,2.68) |
| Central Sub-Saharan Africa | 114.18 (48.35-251.80) | 207.82 (83.14-459.51) | 0.42 (0.18-0.92) | 0.30 (0.12-0.67) | -1.30 (-1.53,-1.06) |  | 117.55 (49.71-262.10) | 213.24 (84.77-483.82) | 0.43 (0.18-0.95) | 0.31 (0.12-0.71) | -1.32 (-1.54,-1.09) |  | 3527.79 (1485.13-7366.59) | 6549.99 (2601.70-14197.54) | 12.84 (5.40-26.81) | 9.57 (3.80-20.74) | -1.20 (-1.42,-0.98) |
| East Asia | 3857.51 (2969.63-4914.71) | 10474.83 (7816.52-13895.83) | 0.63 (0.49-0.81) | 1.42 (1.06-1.89) | 2.94 (2.71,3.17) |  | 3894.88 (3011.66-4973.78) | 9384.72 (7009.52-12438.42) | 0.64 (0.49-0.82) | 1.27 (0.95-1.69) | 2.51 (2.32,2.71) |  | 117052.07 (89679.78-150988.55) | 244914.42 (181032.82-325156.58) | 19.23 (14.73-24.80) | 33.26 (24.58-44.16) | 1.99 (1.81,2.17) |
| Eastern Europe | 829.46 (727.14-940.75) | 1486.69 (1282.29-1691.20) | 0.73 (0.64-0.83) | 1.44 (1.24-1.64) | 2.15 (1.86,2.44) |  | 862.45 (758.16-977.90) | 1549.73 (1344.93-1767.63) | 0.76 (0.67-0.86) | 1.50 (1.30-1.71) | 2.24 (1.91,2.56) |  | 23309.96 (20328.82-26695.98) | 39093.03 (33582.75-44953.44) | 20.58 (17.95-23.57) | 37.82 (32.48-43.48) | 2.03 (1.67,2.39) |
| Eastern Sub-Saharan Africa | 442.82 (296.33-664.64) | 917.67 (619.09-1325.83) | 0.46 (0.31-0.70) | 0.43 (0.29-0.62) | -0.71 (-0.86,-0.56) |  | 461.67 (311.38-688.12) | 953.03 (641.88-1379.82) | 0.48 (0.33-0.72) | 0.45 (0.30-0.65) | -0.70 (-0.85,-0.55) |  | 13145.66 (8798.57-19379.31) | 27640.80 (18328.99-40366.49) | 13.78 (9.22-20.31) | 12.97 (8.60-18.95) | -0.64 (-0.79,-0.48) |
| High-income Asia Pacific | 2339.03 (1906.96-2900.68) | 3676.19 (2859.62-4609.44) | 2.70 (2.20-3.35) | 3.96 (3.08-4.97) | 0.58 (0.26,0.91) |  | 2025.52 (1645.02-2522.49) | 2767.26 (2180.85-3483.90) | 2.34 (1.90-2.91) | 2.98 (2.35-3.76) | 0.22 (-0.13,0.58) |  | 55899.85 (44445.10-70045.73) | 56319.02 (43466.91-72332.95) | 64.48 (51.27-80.80) | 60.74 (46.88-78.01) | -0.82 (-1.17,-0.47) |
| High-income North America | 1256.03 (1130.96-1409.79) | 5734.04 (4999.12-6505.58) | 0.89 (0.80-1.00) | 3.10 (2.70-3.51) | 4.15 (4.10,4.20) |  | 1094.79 (983.55-1230.41) | 4517.35 (3920.73-5106.84) | 0.78 (0.70-0.87) | 2.44 (2.12-2.76) | 3.86 (3.77,3.96) |  | 26682.72 (23990.38-29745.04) | 107158.30 (93483.39-121717.71) | 18.96 (17.05-21.14) | 57.90 (50.51-65.76) | 3.85 (3.78,3.92) |
| North Africa and Middle East | 331.07 (209.63-517.31) | 1105.45 (729.99-1616.67) | 0.20 (0.12-0.31) | 0.35 (0.23-0.52) | 1.92 (1.66,2.19) |  | 347.46 (218.67-547.14) | 1138.46 (755.95-1647.87) | 0.20 (0.13-0.32) | 0.37 (0.24-0.53) | 1.82 (1.55,2.10) |  | 9409.76 (5929.66-14751.31) | 30222.19 (20140.48-44664.66) | 5.55 (3.50-8.70) | 9.70 (6.47-14.34) | 1.76 (1.51,2.02) |
| Oceania | 7.67 (4.12-17.94) | 15.78 (8.51-32.22) | 0.23 (0.13-0.55) | 0.23 (0.12-0.46) | -0.14 (-0.19,-0.09) |  | 7.88 (4.22-18.17) | 16.12 (8.63-32.77) | 0.24 (0.13-0.55) | 0.23 (0.12-0.47) | -0.17 (-0.23,-0.11) |  | 233.37 (123.57-535.57) | 475.59 (254.13-978.42) | 7.13 (3.77-16.35) | 6.83 (3.65-14.05) | -0.12 (-0.19,-0.06) |
| South Asia | 1359.54 (1116.31-1642.26) | 5203.66 (4277.27-6300.21) | 0.25 (0.20-0.30) | 0.56 (0.46-0.68) | 2.75 (2.60,2.90) |  | 1405.28 (1162.67-1695.90) | 5420.82 (4468.99-6604.90) | 0.26 (0.21-0.31) | 0.59 (0.48-0.72) | 2.73 (2.57,2.89) |  | 41060.72 (33936.32-49713.40) | 148080.79 (121494.12-181466.72) | 7.51 (6.21-9.09) | 16.04 (13.16-19.65) | 2.50 (2.35,2.64) |
| Southeast Asia | 1862.18 (1392.90-2491.35) | 5570.38 (4020.19-7829.06) | 0.80 (0.60-1.07) | 1.60 (1.15-2.24) | 2.15 (2.12,2.19) |  | 1919.54 (1432.59-2598.53) | 5582.03 (4040.78-7861.97) | 0.82 (0.62-1.12) | 1.60 (1.16-2.25) | 2.07 (2.03,2.11) |  | 56083.41 (41879.48-75256.87) | 156326.77 (111362.83-224998.33) | 24.10 (17.99-32.33) | 44.77 (31.90-64.44) | 1.94 (1.89,1.99) |
| Southern Latin America | 58.86 (43.01-77.07) | 228.16 (167.49-295.43) | 0.24 (0.17-0.31) | 0.67 (0.49-0.87) | 4.08 (3.82,4.35) |  | 62.10 (45.74-81.12) | 235.65 (172.38-308.34) | 0.25 (0.18-0.33) | 0.70 (0.51-0.91) | 4.06 (3.76,4.35) |  | 1574.86 (1167.52-2089.02) | 5564.96 (4117.49-7193.16) | 6.36 (4.71-8.43) | 16.44 (12.16-21.25) | 3.83 (3.53,4.13) |
| Southern Sub-Saharan Africa | 156.85 (85.02-267.26) | 456.29 (362.30-576.21) | 0.60 (0.32-1.02) | 1.14 (0.90-1.44) | 1.08 (0.50,1.66) |  | 162.66 (88.99-278.63) | 472.77 (375.67-593.49) | 0.62 (0.34-1.06) | 1.18 (0.94-1.48) | 1.05 (0.43,1.68) |  | 4819.67 (2618.13-8090.26) | 13597.85 (10676.24-17385.51) | 18.39 (9.99-30.87) | 33.87 (26.59-43.30) | 1.00 (0.35,1.65) |
| Tropical Latin America | 220.16 (193.22-249.60) | 719.99 (621.21-825.64) | 0.29 (0.25-0.33) | 0.63 (0.55-0.73) | 3.22 (2.96,3.48) |  | 230.89 (202.51-261.38) | 756.35 (653.86-867.40) | 0.30 (0.27-0.34) | 0.66 (0.57-0.76) | 3.31 (3.05,3.58) |  | 6339.61 (5550.99-7248.42) | 19296.55 (16530.04-22168.71) | 8.31 (7.28-9.50) | 16.96 (14.53-19.49) | 3.10 (2.82,3.38) |
| Western Europe | 3699.48 (3139.45-4330.19) | 8504.53 (6860.16-10119.58) | 1.92 (1.63-2.25) | 3.89 (3.14-4.63) | 2.37 (2.21,2.53) |  | 3671.66 (3101.60-4308.49) | 7472.43 (6000.25-8986.61) | 1.91 (1.61-2.24) | 3.42 (2.74-4.11) | 1.94 (1.83,2.06) |  | 86276.47 (72559.22-100880.11) | 158499.81 (129422.45-190177.98) | 44.89 (37.75-52.49) | 72.48 (59.18-86.96) | 1.60 (1.48,1.72) |
| Western Sub-Saharan Africa | 878.69 (491.92-1359.17) | 1532.64 (1105.33-2070.59) | 0.91 (0.51-1.41) | 0.63 (0.45-0.85) | -1.55 (-1.68,-1.42) |  | 919.01 (519.53-1415.35) | 1598.41 (1163.98-2149.98) | 0.95 (0.54-1.47) | 0.65 (0.48-0.88) | -1.54 (-1.67,-1.41) |  | 26039.58 (14469.99-40201.31) | 45828.47 (32250.93-62210.43) | 26.96 (14.98-41.63) | 18.71 (13.17-25.40) | -1.50 (-1.64,-1.36) |

**Abbreviations:** UI, uncertainty interval; ASR, age-standardised rate per 100,000; EAPC, estimated annual percentage change; CI, confidence interval; DALYs, disability-adjusted life-year; SDI, socio-demographic index.

**Table 3. Global burden of hepatoblastoma in 1990 and 2021, categorized by sex, region, and SDI**

| **Characteristics** | **Incidence** | | | | |  | **Deaths** | | | | |  | **DALYs** | | | | |
| --- | --- | --- | --- | --- | --- | --- | --- | --- | --- | --- | --- | --- | --- | --- | --- | --- | --- |
|  | **1990 No. (95% UI)** | **2021 No. (95% UI)** | **1990 ASR per 100 000 (95% UI)** | **2021 ASR per 100 000 (95% UI)** | **EAPC (95% CI)** |  | **1990 No. (95% UI)** | **2021 No. (95% UI)** | **1990 ASR per 100 000 (95% UI)** | **2021 ASR per 100 000 (95% UI)** | **EAPC (95% CI)** |  | **1990 No. (95% UI)** | **2021 No. (95% UI)** | **1990 ASR per 100 000 (95% UI)** | **2021 ASR per 100 000 (95% UI)** | **EAPC (95% CI)** |
| **Global** | 3531.85 (2899.42-4140.36) | 2024.21 (1626.23-2500.23) | 0.13 (0.11-0.16) | 0.05 (0.04-0.06) | -2.93 (-3.09,-2.77) |  | 2414.14 (1969.30-2835.27) | 1208.08 (961.23-1509.52) | 0.09 (0.07-0.11) | 0.03 (0.02-0.04) | -3.43 (-3.58,-3.28) |  | 213182.33 (174427.85-250077.94) | 106738.95 (85044.76-133625.13) | 7.99 (6.54-9.38) | 2.71 (2.16-3.39) | -3.42 (-3.57,-3.27) |
| **Sex** |  |  |  |  |  |  |  |  |  |  |  |  |  |  |  |  |  |
| Male | 1867.66 (1513.91-2171.88) | 1044.09 (849.29-1278.93) | 0.14 (0.11-0.16) | 0.05 (0.04-0.06) | -3.04 (-3.20,-2.88) |  | 1274.05 (1025.19-1485.94) | 609.28 (489.82-759.76) | 0.09 (0.08-0.11) | 0.03 (0.02-0.04) | -3.62 (-3.78,-3.46) |  | 112367.51 (90531.95-131570.86) | 53807.51 (43214.50-67188.20) | 8.37 (6.74-9.80) | 2.72 (2.18-3.39) | -3.60 (-3.76,-3.45) |
| Female | 1664.19 (1328.52-2000.67) | 980.13 (770.58-1249.78) | 0.13 (0.10-0.15) | 0.05 (0.04-0.06) | -2.80 (-2.98,-2.63) |  | 1140.10 (905.48-1380.72) | 598.80 (464.73-768.74) | 0.09 (0.07-0.10) | 0.03 (0.02-0.04) | -3.22 (-3.39,-3.06) |  | 100814.83 (80022.07-122169.64) | 52931.45 (41128.80-67786.86) | 7.61 (6.04-9.23) | 2.69 (2.09-3.45) | -3.22 (-3.38,-3.05) |
| **SDI category** |  |  |  |  |  |  |  |  |  |  |  |  |  |  |  |  |  |
| Low SDI | 715.76 (494.14-925.41) | 708.29 (498.08-958.45) | 0.29 (0.20-0.37) | 0.13 (0.09-0.17) | -2.50 (-2.62,-2.38) |  | 506.20 (350.01-654.27) | 493.69 (348.05-669.53) | 0.20 (0.14-0.26) | 0.09 (0.06-0.12) | -2.55 (-2.67,-2.42) |  | 44849.39 (31157.25-57976.82) | 43752.66 (30821.44-59422.92) | 17.89 (12.43-23.13) | 7.83 (5.52-10.64) | -2.54 (-2.67,-2.42) |
| Low-middle SDI | 826.58 (557.21-1042.85) | 550.99 (443.17-672.66) | 0.14 (0.10-0.18) | 0.06 (0.05-0.07) | -2.58 (-2.69,-2.46) |  | 583.48 (393.36-734.77) | 376.15 (301.77-459.78) | 0.10 (0.07-0.13) | 0.04 (0.03-0.05) | -2.69 (-2.80,-2.57) |  | 51486.60 (34738.86-64774.95) | 33196.44 (26623.53-40496.03) | 8.87 (5.98-11.15) | 3.46 (2.77-4.22) | -2.68 (-2.80,-2.57) |
| Middle SDI | 1324.51 (1151.30-1527.96) | 411.75 (327.92-521.99) | 0.15 (0.13-0.18) | 0.03 (0.03-0.04) | -4.91 (-5.26,-4.56) |  | 921.10 (802.46-1064.38) | 229.43 (184.66-284.54) | 0.11 (0.09-0.12) | 0.02 (0.02-0.02) | -5.67 (-5.97,-5.37) |  | 81272.45 (70783.49-93958.10) | 20149.49 (16195.66-25082.70) | 9.43 (8.22-10.91) | 1.65 (1.32-2.05) | -5.67 (-5.97,-5.38) |
| High-middle SDI | 498.43 (429.62-583.46) | 179.82 (144.99-224.03) | 0.09 (0.08-0.11) | 0.03 (0.02-0.03) | -3.96 (-4.39,-3.53) |  | 330.16 (283.93-387.21) | 65.65 (54.38-79.32) | 0.06 (0.05-0.07) | 0.01 (0.01-0.01) | -6.23 (-6.62,-5.85) |  | 29110.99 (24990.00-34221.46) | 5789.99 (4798.42-6976.87) | 5.47 (4.70-6.44) | 0.89 (0.74-1.07) | -6.21 (-6.58,-5.83) |
| High SDI | 165.07 (156.10-174.71) | 172.48 (157.36-187.01) | 0.04 (0.04-0.04) | 0.03 (0.03-0.03) | -0.33 (-0.57,-0.09) |  | 72.19 (66.55-78.11) | 42.61 (39.06-45.82) | 0.02 (0.02-0.02) | 0.01 (0.01-0.01) | -2.22 (-2.30,-2.15) |  | 6373.19 (5882.64-6891.12) | 3801.84 (3489.33-4094.71) | 1.45 (1.34-1.57) | 0.70 (0.64-0.75) | -2.18 (-2.25,-2.11) |
| **Region** |  |  |  |  |  |  |  |  |  |  |  |  |  |  |  |  |  |
| Andean Latin America | 19.74 (14.18-25.93) | 7.77 (5.22-11.20) | 0.10 (0.07-0.14) | 0.02 (0.02-0.03) | -4.83 (-5.15,-4.52) |  | 13.86 (9.98-18.24) | 4.96 (3.43-7.22) | 0.07 (0.05-0.10) | 0.01 (0.01-0.02) | -5.15 (-5.43,-4.87) |  | 1220.02 (878.55-1604.86) | 433.12 (297.78-629.12) | 6.42 (4.62-8.45) | 1.31 (0.90-1.90) | -5.17 (-5.45,-4.88) |
| Australasia | 3.11 (2.76-3.47) | 6.64 (5.20-8.31) | 0.03 (0.03-0.03) | 0.04 (0.03-0.05) | 1.33 (1.06,1.59) |  | 1.03 (0.91-1.15) | 1.54 (1.23-1.90) | 0.01 (0.01-0.01) | 0.01 (0.01-0.01) | 0.30 (0.07,0.53) |  | 91.14 (80.74-101.39) | 137.25 (109.24-169.33) | 0.90 (0.80-1.00) | 0.89 (0.71-1.09) | 0.32 (0.10,0.55) |
| Caribbean | 5.77 (3.68-8.35) | 3.55 (2.34-5.52) | 0.03 (0.02-0.05) | 0.01 (0.01-0.02) | -2.40 (-2.67,-2.13) |  | 4.02 (2.55-5.84) | 2.39 (1.56-3.75) | 0.02 (0.01-0.03) | 0.01 (0.01-0.02) | -2.50 (-2.78,-2.22) |  | 354.37 (224.59-514.32) | 210.62 (137.26-329.54) | 2.01 (1.27-2.91) | 0.89 (0.58-1.39) | -2.50 (-2.78,-2.22) |
| Central Asia | 57.34 (46.93-71.83) | 21.21 (15.52-28.38) | 0.17 (0.14-0.21) | 0.04 (0.03-0.06) | -4.01 (-4.45,-3.56) |  | 39.56 (32.42-49.68) | 14.02 (10.26-18.63) | 0.11 (0.09-0.14) | 0.03 (0.02-0.04) | -4.20 (-4.61,-3.79) |  | 3484.63 (2851.87-4377.13) | 1226.28 (895.28-1628.59) | 10.05 (8.23-12.63) | 2.56 (1.87-3.40) | -4.21 (-4.62,-3.79) |
| Central Europe | 21.57 (18.60-25.07) | 4.46 (3.60-5.52) | 0.03 (0.03-0.04) | 0.01 (0.01-0.01) | -4.60 (-5.22,-3.97) |  | 14.04 (12.11-16.37) | 1.76 (1.42-2.16) | 0.02 (0.02-0.03) | 0.00 (0.00-0.00) | -6.38 (-6.93,-5.83) |  | 1233.69 (1065.02-1439.81) | 154.46 (125.49-189.93) | 1.97 (1.70-2.30) | 0.27 (0.22-0.33) | -6.37 (-6.92,-5.81) |
| Central Latin America | 87.50 (81.09-95.54) | 43.12 (33.20-56.43) | 0.11 (0.10-0.12) | 0.03 (0.03-0.04) | -3.24 (-3.37,-3.12) |  | 61.03 (56.59-66.51) | 27.49 (21.26-35.56) | 0.07 (0.07-0.08) | 0.02 (0.02-0.03) | -3.53 (-3.65,-3.40) |  | 5370.02 (4975.09-5852.60) | 2407.24 (1859.44-3119.14) | 6.53 (6.05-7.12) | 1.90 (1.47-2.47) | -3.54 (-3.67,-3.42) |
| Central Sub-Saharan Africa | 74.84 (40.26-118.23) | 48.27 (21.53-88.53) | 0.27 (0.15-0.43) | 0.07 (0.03-0.13) | -4.18 (-4.44,-3.92) |  | 53.25 (28.80-84.59) | 33.97 (15.27-64.07) | 0.19 (0.10-0.31) | 0.05 (0.02-0.09) | -4.22 (-4.48,-3.96) |  | 4698.11 (2535.45-7432.43) | 2985.53 (1341.49-5655.79) | 17.10 (9.23-27.05) | 4.36 (1.96-8.26) | -4.23 (-4.49,-3.97) |
| East Asia | 1157.60 (961.82-1417.13) | 282.89 (200.78-400.95) | 0.19 (0.16-0.23) | 0.04 (0.03-0.05) | -5.40 (-5.94,-4.85) |  | 800.39 (667.25-977.50) | 110.81 (80.17-154.72) | 0.13 (0.11-0.16) | 0.02 (0.01-0.02) | -7.36 (-7.83,-6.88) |  | 70711.30 (58927.63-86429.24) | 9746.69 (7028.83-13664.61) | 11.62 (9.68-14.20) | 1.32 (0.95-1.86) | -7.35 (-7.81,-6.88) |
| Eastern Europe | 77.58 (72.49-82.84) | 28.85 (26.06-31.55) | 0.07 (0.06-0.07) | 0.03 (0.03-0.03) | -2.35 (-3.13,-1.56) |  | 49.54 (46.50-52.81) | 12.97 (11.82-14.13) | 0.04 (0.04-0.05) | 0.01 (0.01-0.01) | -3.74 (-4.33,-3.13) |  | 4346.62 (4081.84-4623.92) | 1137.96 (1032.51-1241.19) | 3.84 (3.60-4.08) | 1.10 (1.00-1.20) | -3.70 (-4.29,-3.10) |
| Eastern Sub-Saharan Africa | 285.29 (194.79-375.41) | 254.89 (148.46-443.85) | 0.30 (0.20-0.39) | 0.12 (0.07-0.21) | -2.78 (-2.92,-2.64) |  | 201.58 (137.66-265.32) | 178.05 (104.45-311.34) | 0.21 (0.14-0.28) | 0.08 (0.05-0.15) | -2.81 (-2.96,-2.67) |  | 17826.16 (12167.59-23400.56) | 15692.87 (9206.16-27487.44) | 18.68 (12.75-24.53) | 7.37 (4.32-12.90) | -2.82 (-2.97,-2.68) |
| High-income Asia Pacific | 50.03 (41.78-59.44) | 18.81 (16.54-22.24) | 0.06 (0.05-0.07) | 0.02 (0.02-0.02) | -3.46 (-4.18,-2.73) |  | 23.53 (18.14-29.57) | 4.27 (3.81-4.96) | 0.03 (0.02-0.03) | 0.00 (0.00-0.01) | -5.87 (-6.27,-5.46) |  | 2066.33 (1603.68-2595.23) | 381.59 (340.80-442.17) | 2.38 (1.85-2.99) | 0.41 (0.37-0.48) | -5.81 (-6.22,-5.39) |
| High-income North America | 54.98 (53.54-56.49) | 89.58 (79.76-100.06) | 0.04 (0.04-0.04) | 0.05 (0.04-0.05) | 1.06 (0.78,1.33) |  | 18.80 (18.36-19.24) | 22.36 (20.09-24.83) | 0.01 (0.01-0.01) | 0.01 (0.01-0.01) | 0.05 (-0.10,0.20) |  | 1669.55 (1629.81-1708.52) | 1995.85 (1791.89-2215.00) | 1.19 (1.16-1.21) | 1.08 (0.97-1.20) | 0.07 (-0.08,0.23) |
| North Africa and Middle East | 247.25 (181.60-313.38) | 164.60 (129.13-209.12) | 0.15 (0.11-0.18) | 0.05 (0.04-0.07) | -2.54 (-2.77,-2.30) |  | 172.56 (126.98-219.91) | 103.91 (81.57-132.31) | 0.10 (0.07-0.13) | 0.03 (0.03-0.04) | -2.88 (-3.09,-2.66) |  | 15224.29 (11192.09-19395.73) | 9155.78 (7184.75-11655.27) | 8.98 (6.60-11.44) | 2.94 (2.31-3.74) | -2.87 (-3.09,-2.65) |
| Oceania | 1.47 (0.91-2.44) | 2.02 (1.16-3.53) | 0.04 (0.03-0.07) | 0.03 (0.02-0.05) | -1.60 (-1.89,-1.31) |  | 1.03 (0.64-1.78) | 1.39 (0.79-2.43) | 0.03 (0.02-0.05) | 0.02 (0.01-0.03) | -1.64 (-1.93,-1.35) |  | 91.41 (56.77-157.95) | 123.23 (70.27-214.88) | 2.79 (1.73-4.82) | 1.77 (1.01-3.09) | -1.64 (-1.94,-1.35) |
| South Asia | 588.83 (353.35-793.85) | 407.77 (316.34-522.27) | 0.11 (0.06-0.15) | 0.04 (0.03-0.06) | -2.62 (-2.72,-2.53) |  | 416.96 (248.23-561.09) | 279.74 (218.07-358.31) | 0.08 (0.05-0.10) | 0.03 (0.02-0.04) | -2.73 (-2.83,-2.64) |  | 36750.68 (21967.55-49489.88) | 24696.14 (19325.24-31649.98) | 6.72 (4.02-9.05) | 2.67 (2.09-3.43) | -2.72 (-2.82,-2.63) |
| Southeast Asia | 324.72 (217.45-416.36) | 126.21 (92.86-173.28) | 0.14 (0.09-0.18) | 0.04 (0.03-0.05) | -4.13 (-4.29,-3.97) |  | 226.31 (151.70-290.68) | 81.91 (60.91-113.07) | 0.10 (0.07-0.12) | 0.02 (0.02-0.03) | -4.37 (-4.51,-4.23) |  | 19908.91 (13365.88-25638.45) | 7189.73 (5334.48-9902.34) | 8.55 (5.74-11.02) | 2.06 (1.53-2.84) | -4.37 (-4.52,-4.23) |
| Southern Latin America | 1.74 (1.50-2.01) | 1.93 (1.58-2.31) | 0.01 (0.01-0.01) | 0.01 (0.00-0.01) | 0.18 (-0.13,0.50) |  | 1.17 (1.01-1.36) | 1.07 (0.88-1.29) | 0.00 (0.00-0.01) | 0.00 (0.00-0.00) | -0.57 (-0.86,-0.27) |  | 103.19 (88.87-119.16) | 93.29 (76.45-112.47) | 0.42 (0.36-0.48) | 0.28 (0.23-0.33) | -0.58 (-0.88,-0.28) |
| Southern Sub-Saharan Africa | 16.48 (10.66-22.48) | 19.83 (13.88-26.68) | 0.06 (0.04-0.09) | 0.05 (0.03-0.07) | -0.79 (-1.02,-0.55) |  | 11.54 (7.44-15.98) | 13.89 (9.86-18.63) | 0.04 (0.03-0.06) | 0.03 (0.02-0.05) | -0.79 (-1.03,-0.55) |  | 1016.42 (654.57-1408.63) | 1216.16 (864.96-1635.26) | 3.88 (2.50-5.37) | 3.03 (2.15-4.07) | -0.81 (-1.06,-0.57) |
| Tropical Latin America | 39.05 (33.77-46.00) | 19.96 (15.45-24.45) | 0.05 (0.04-0.06) | 0.02 (0.01-0.02) | -2.98 (-3.25,-2.71) |  | 27.17 (23.52-32.09) | 12.83 (9.91-15.69) | 0.04 (0.03-0.04) | 0.01 (0.01-0.01) | -3.21 (-3.50,-2.92) |  | 2391.95 (2068.55-2826.53) | 1127.97 (871.84-1380.47) | 3.14 (2.71-3.71) | 0.99 (0.77-1.21) | -3.22 (-3.50,-2.93) |
| Western Europe | 52.85 (50.41-55.38) | 60.04 (52.33-68.24) | 0.03 (0.03-0.03) | 0.03 (0.02-0.03) | 0.30 (0.11,0.50) |  | 20.46 (19.50-21.46) | 13.47 (11.92-15.14) | 0.01 (0.01-0.01) | 0.01 (0.01-0.01) | -1.31 (-1.55,-1.07) |  | 1814.88 (1730.19-1902.57) | 1206.18 (1071.81-1355.32) | 0.94 (0.90-0.99) | 0.55 (0.49-0.62) | -1.28 (-1.51,-1.04) |
| Western Sub-Saharan Africa | 364.10 (264.11-479.33) | 411.82 (295.08-546.45) | 0.38 (0.27-0.50) | 0.17 (0.12-0.22) | -2.48 (-2.63,-2.32) |  | 256.29 (186.37-336.42) | 285.28 (206.30-377.15) | 0.27 (0.19-0.35) | 0.12 (0.08-0.15) | -2.53 (-2.69,-2.37) |  | 22808.64 (16619.87-29931.13) | 25421.03 (18406.10-33592.11) | 23.62 (17.21-30.99) | 10.38 (7.52-13.72) | -2.52 (-2.68,-2.36) |

**Abbreviations:** UI, uncertainty interval; ASR, age-standardised rate per 100,000; EAPC, estimated annual percentage change; CI, confidence interval; DALYs, disability-adjusted life-year; SDI, socio-demographic index.

**Table 4. Global burden of liver cancer due to other causes in 1990 and 2021, categorized by sex, region, and SDI**

| **Characteristics** | **Incidence** | | | | |  | **Deaths** | | | | |  | **DALYs** | | | | |
| --- | --- | --- | --- | --- | --- | --- | --- | --- | --- | --- | --- | --- | --- | --- | --- | --- | --- |
|  | **1990 No. (95% UI)** | **2021 No. (95% UI)** | **1990 ASR per 100 000 (95% UI)** | **2021 ASR per 100 000 (95% UI)** | **EAPC (95% CI)** |  | **1990 No. (95% UI)** | **2021 No. (95% UI)** | **1990 ASR per 100 000 (95% UI)** | **2021 ASR per 100 000 (95% UI)** | **EAPC (95% CI)** |  | **1990 No. (95% UI)** | **2021 No. (95% UI)** | **1990 ASR per 100 000 (95% UI)** | **2021 ASR per 100 000 (95% UI)** | **EAPC (95% CI)** |
| **Global** | 5457.45 (4463.40-6792.12) | 11445.85 (9187.44-13900.70) | 0.20 (0.17-0.25) | 0.29 (0.23-0.35) | 1.10 (1.03,1.17) |  | 5324.97 (4350.28-6662.97) | 10294.96 (8185.59-12523.81) | 0.20 (0.16-0.25) | 0.26 (0.21-0.32) | 0.84 (0.75,0.93) |  | 180394.30 (149631.61-222072.88) | 297801.60 (242398.28-366156.71) | 6.76 (5.61-8.33) | 7.55 (6.14-9.28) | 0.27 (0.16,0.37) |
| **Sex** |  |  |  |  |  |  |  |  |  |  |  |  |  |  |  |  |  |
| Male | 2706.88 (2204.80-3352.97) | 5944.80 (4605.02-7447.36) | 0.20 (0.16-0.25) | 0.30 (0.23-0.38) | 1.22 (1.14,1.31) |  | 2591.58 (2106.08-3208.39) | 5169.74 (3992.47-6491.93) | 0.19 (0.16-0.24) | 0.26 (0.20-0.33) | 0.90 (0.81,0.99) |  | 89881.97 (73977.75-112322.93) | 154451.67 (120598.63-197068.46) | 6.69 (5.51-8.36) | 7.80 (6.09-9.95) | 0.37 (0.26,0.49) |
| Female | 2750.57 (2182.69-3485.11) | 5501.05 (4397.37-6734.29) | 0.21 (0.16-0.26) | 0.28 (0.22-0.34) | 0.97 (0.90,1.04) |  | 2733.39 (2157.33-3467.62) | 5125.22 (4104.60-6291.14) | 0.21 (0.16-0.26) | 0.26 (0.21-0.32) | 0.78 (0.70,0.87) |  | 90512.34 (72993.48-113925.18) | 143349.93 (116090.60-175747.07) | 6.84 (5.51-8.61) | 7.29 (5.90-8.94) | 0.16 (0.06,0.26) |
| **SDI category** |  |  |  |  |  |  |  |  |  |  |  |  |  |  |  |  |  |
| Low SDI | 362.74 (240.01-566.31) | 706.77 (502.85-988.29) | 0.14 (0.10-0.23) | 0.13 (0.09-0.18) | -0.68 (-0.77,-0.59) |  | 372.39 (246.04-580.71) | 723.17 (513.84-1011.37) | 0.15 (0.10-0.23) | 0.13 (0.09-0.18) | -0.68 (-0.78,-0.59) |  | 13199.78 (8979.82-20289.27) | 26127.86 (18756.64-36675.58) | 5.27 (3.58-8.09) | 4.68 (3.36-6.56) | -0.63 (-0.72,-0.53) |
| Low-middle SDI | 520.36 (380.79-748.59) | 1274.86 (981.10-1641.06) | 0.09 (0.07-0.13) | 0.13 (0.10-0.17) | 1.30 (1.24,1.36) |  | 531.89 (389.27-765.70) | 1295.86 (997.94-1650.61) | 0.09 (0.07-0.13) | 0.13 (0.10-0.17) | 1.25 (1.17,1.33) |  | 19119.67 (14364.99-26675.64) | 43425.46 (34361.85-55299.57) | 3.29 (2.47-4.59) | 4.52 (3.58-5.76) | 1.02 (0.98,1.06) |
| Middle SDI | 1923.51 (1570.85-2381.87) | 3850.95 (3012.09-4861.18) | 0.22 (0.18-0.28) | 0.31 (0.25-0.40) | 1.32 (1.12,1.51) |  | 1912.69 (1562.29-2356.88) | 3506.27 (2737.69-4372.88) | 0.22 (0.18-0.27) | 0.29 (0.22-0.36) | 1.02 (0.85,1.19) |  | 69145.02 (57027.48-84849.46) | 105477.57 (83268.40-133272.70) | 8.03 (6.62-9.85) | 8.62 (6.80-10.89) | 0.31 (0.15,0.48) |
| High-middle SDI | 1339.41 (1079.12-1657.95) | 2410.13 (1874.45-3017.55) | 0.25 (0.20-0.31) | 0.37 (0.29-0.46) | 1.19 (1.04,1.34) |  | 1324.93 (1067.29-1643.03) | 2167.53 (1687.63-2692.73) | 0.25 (0.20-0.31) | 0.33 (0.26-0.41) | 0.85 (0.70,1.01) |  | 45837.93 (37105.77-56703.76) | 61883.17 (48433.27-78582.39) | 8.62 (6.98-10.66) | 9.49 (7.43-12.05) | 0.10 (-0.10,0.30) |
| High SDI | 1308.62 (1083.49-1576.43) | 3198.21 (2580.49-3852.99) | 0.30 (0.25-0.36) | 0.58 (0.47-0.70) | 1.92 (1.72,2.11) |  | 1180.15 (966.52-1433.29) | 2597.18 (2072.37-3161.41) | 0.27 (0.22-0.33) | 0.47 (0.38-0.58) | 1.64 (1.50,1.79) |  | 33003.14 (27589.71-39917.61) | 60747.93 (49558.49-73401.54) | 7.50 (6.27-9.08) | 11.11 (9.06-13.42) | 1.08 (0.93,1.24) |
| **Region** |  |  |  |  |  |  |  |  |  |  |  |  |  |  |  |  |  |
| Andean Latin America | 18.27 (13.11-24.56) | 45.90 (30.66-64.23) | 0.10 (0.07-0.13) | 0.14 (0.09-0.19) | 1.21 (0.94,1.47) |  | 19.22 (13.84-25.70) | 48.65 (32.53-68.35) | 0.10 (0.07-0.14) | 0.15 (0.10-0.21) | 1.20 (0.92,1.48) |  | 632.30 (463.50-845.70) | 1321.78 (880.33-1848.30) | 3.33 (2.44-4.45) | 4.00 (2.66-5.59) | 0.49 (0.20,0.77) |
| Australasia | 9.82 (7.37-12.58) | 62.17 (45.40-82.45) | 0.10 (0.07-0.12) | 0.40 (0.29-0.53) | 4.73 (4.56,4.90) |  | 9.29 (6.87-11.98) | 54.11 (39.83-72.31) | 0.09 (0.07-0.12) | 0.35 (0.26-0.47) | 4.36 (4.23,4.50) |  | 278.03 (211.68-353.61) | 1374.33 (1014.15-1819.33) | 2.74 (2.09-3.49) | 8.88 (6.55-11.75) | 3.89 (3.78,4.00) |
| Caribbean | 15.22 (10.71-20.58) | 25.23 (17.60-34.24) | 0.09 (0.06-0.12) | 0.11 (0.07-0.14) | 0.38 (0.12,0.63) |  | 15.87 (11.10-21.46) | 26.09 (18.23-35.62) | 0.09 (0.06-0.12) | 0.11 (0.08-0.15) | 0.39 (0.10,0.67) |  | 485.14 (359.86-649.93) | 745.61 (524.64-1025.45) | 2.75 (2.04-3.68) | 3.14 (2.21-4.32) | 0.19 (-0.08,0.46) |
| Central Asia | 59.83 (42.28-84.90) | 89.15 (60.98-126.12) | 0.17 (0.12-0.24) | 0.19 (0.13-0.26) | 0.09 (0.01,0.17) |  | 61.47 (43.33-87.01) | 91.64 (63.17-129.74) | 0.18 (0.13-0.25) | 0.19 (0.13-0.27) | 0.04 (-0.04,0.13) |  | 2096.99 (1526.25-2878.59) | 2924.79 (2007.16-4078.60) | 6.05 (4.40-8.31) | 6.11 (4.19-8.51) | -0.20 (-0.29,-0.11) |
| Central Europe | 71.08 (50.44-96.23) | 85.79 (61.77-114.71) | 0.11 (0.08-0.15) | 0.15 (0.11-0.20) | 0.96 (0.81,1.12) |  | 75.01 (53.20-101.35) | 91.07 (65.18-122.83) | 0.12 (0.09-0.16) | 0.16 (0.11-0.21) | 1.01 (0.82,1.21) |  | 2105.08 (1543.38-2854.06) | 2169.78 (1587.91-2915.78) | 3.37 (2.47-4.56) | 3.76 (2.76-5.06) | 0.44 (0.25,0.64) |
| Central Latin America | 68.63 (54.89-86.73) | 170.71 (132.59-211.38) | 0.08 (0.07-0.11) | 0.13 (0.10-0.17) | 1.80 (1.47,2.12) |  | 71.58 (56.96-90.25) | 178.28 (138.07-221.90) | 0.09 (0.07-0.11) | 0.14 (0.11-0.18) | 1.73 (1.37,2.09) |  | 2467.94 (2045.56-3015.79) | 5252.01 (4178.13-6536.32) | 3.00 (2.49-3.67) | 4.15 (3.30-5.17) | 1.22 (0.88,1.55) |
| Central Sub-Saharan Africa | 32.19 (14.16-73.60) | 59.99 (24.88-135.17) | 0.12 (0.05-0.27) | 0.09 (0.04-0.20) | -1.25 (-1.43,-1.08) |  | 32.63 (14.37-73.67) | 60.72 (25.15-138.36) | 0.12 (0.05-0.27) | 0.09 (0.04-0.20) | -1.25 (-1.42,-1.08) |  | 1251.74 (558.90-2811.38) | 2336.24 (979.51-5284.16) | 4.55 (2.03-10.23) | 3.41 (1.43-7.72) | -1.20 (-1.36,-1.04) |
| East Asia | 2604.12 (2065.71-3248.98) | 4769.93 (3650.87-6130.63) | 0.43 (0.34-0.53) | 0.65 (0.50-0.83) | 1.51 (1.36,1.66) |  | 2559.22 (2032.27-3179.99) | 4153.20 (3168.40-5283.49) | 0.42 (0.33-0.52) | 0.56 (0.43-0.72) | 1.10 (0.95,1.24) |  | 92925.24 (74612.73-115282.98) | 122675.89 (93644.92-160042.30) | 15.27 (12.26-18.94) | 16.66 (12.72-21.73) | 0.28 (0.10,0.45) |
| Eastern Europe | 80.79 (68.17-97.58) | 118.41 (97.94-140.69) | 0.07 (0.06-0.09) | 0.11 (0.09-0.14) | 1.58 (1.36,1.81) |  | 83.67 (70.60-100.51) | 123.48 (101.84-146.30) | 0.07 (0.06-0.09) | 0.12 (0.10-0.14) | 1.67 (1.40,1.95) |  | 2622.48 (2240.92-3122.16) | 3364.17 (2827.24-4016.69) | 2.32 (1.98-2.76) | 3.25 (2.73-3.89) | 1.15 (0.87,1.43) |
| Eastern Sub-Saharan Africa | 139.67 (100.61-196.79) | 293.83 (203.65-412.27) | 0.15 (0.11-0.21) | 0.14 (0.10-0.19) | -0.53 (-0.64,-0.42) |  | 143.71 (102.93-202.79) | 301.39 (209.40-420.88) | 0.15 (0.11-0.21) | 0.14 (0.10-0.20) | -0.52 (-0.63,-0.41) |  | 5261.55 (3905.20-7175.36) | 11061.46 (7593.02-15716.11) | 5.51 (4.09-7.52) | 5.19 (3.56-7.38) | -0.51 (-0.62,-0.40) |
| High-income Asia Pacific | 522.34 (421.87-651.56) | 756.80 (570.70-948.48) | 0.60 (0.49-0.75) | 0.82 (0.62-1.02) | 0.22 (-0.17,0.62) |  | 449.13 (358.91-564.70) | 572.16 (428.80-717.26) | 0.52 (0.41-0.65) | 0.62 (0.46-0.77) | -0.08 (-0.48,0.32) |  | 13031.42 (10327.28-16426.71) | 11188.23 (8547.83-14157.39) | 15.03 (11.91-18.95) | 12.07 (9.22-15.27) | -1.39 (-1.79,-0.99) |
| High-income North America | 373.16 (322.99-423.65) | 1400.89 (1188.11-1652.35) | 0.27 (0.23-0.30) | 0.76 (0.64-0.89) | 3.35 (3.24,3.46) |  | 318.75 (275.59-365.16) | 1093.15 (927.28-1288.65) | 0.23 (0.20-0.26) | 0.59 (0.50-0.70) | 3.09 (3.04,3.14) |  | 8762.90 (7681.02-9978.40) | 27534.92 (23477.55-32486.91) | 6.23 (5.46-7.09) | 14.88 (12.68-17.55) | 2.91 (2.81,3.01) |
| North Africa and Middle East | 247.49 (162.67-390.85) | 697.99 (490.80-979.75) | 0.15 (0.10-0.23) | 0.22 (0.16-0.31) | 1.43 (1.23,1.63) |  | 254.29 (166.42-403.52) | 702.10 (499.78-978.96) | 0.15 (0.10-0.24) | 0.23 (0.16-0.31) | 1.32 (1.11,1.53) |  | 8820.09 (6078.70-13139.65) | 22407.05 (16059.35-31027.61) | 5.20 (3.58-7.75) | 7.19 (5.16-9.96) | 1.06 (0.89,1.24) |
| Oceania | 2.97 (1.57-6.44) | 5.74 (3.20-10.62) | 0.09 (0.05-0.20) | 0.08 (0.05-0.15) | -0.34 (-0.41,-0.27) |  | 2.98 (1.57-6.41) | 5.69 (3.16-10.55) | 0.09 (0.05-0.20) | 0.08 (0.05-0.15) | -0.40 (-0.47,-0.33) |  | 111.64 (58.10-240.62) | 209.74 (119.74-388.98) | 3.41 (1.77-7.35) | 3.01 (1.72-5.59) | -0.43 (-0.52,-0.35) |
| South Asia | 208.14 (174.83-249.47) | 648.99 (537.50-776.78) | 0.04 (0.03-0.05) | 0.07 (0.06-0.08) | 2.04 (2.01,2.08) |  | 213.60 (179.71-254.54) | 668.19 (554.16-796.51) | 0.04 (0.03-0.05) | 0.07 (0.06-0.09) | 2.03 (1.98,2.08) |  | 7803.82 (6592.25-9224.90) | 22014.92 (18438.24-26226.66) | 1.43 (1.21-1.69) | 2.38 (2.00-2.84) | 1.65 (1.59,1.71) |
| Southeast Asia | 282.97 (210.70-381.51) | 600.63 (405.96-853.68) | 0.12 (0.09-0.16) | 0.17 (0.12-0.24) | 1.08 (1.02,1.13) |  | 286.14 (213.65-385.65) | 590.21 (398.00-835.24) | 0.12 (0.09-0.17) | 0.17 (0.11-0.24) | 0.99 (0.94,1.04) |  | 10103.58 (7789.24-13336.76) | 18766.16 (12869.93-26417.09) | 4.34 (3.35-5.73) | 5.37 (3.69-7.57) | 0.64 (0.58,0.70) |
| Southern Latin America | 9.84 (6.79-14.03) | 39.83 (28.43-53.74) | 0.04 (0.03-0.06) | 0.12 (0.08-0.16) | 4.10 (3.92,4.28) |  | 10.26 (7.08-14.57) | 40.83 (28.67-56.45) | 0.04 (0.03-0.06) | 0.12 (0.08-0.17) | 4.09 (3.87,4.32) |  | 289.98 (203.34-408.69) | 1051.39 (751.69-1416.65) | 1.17 (0.82-1.65) | 3.11 (2.22-4.19) | 3.78 (3.55,4.01) |
| Southern Sub-Saharan Africa | 40.38 (26.30-60.10) | 106.05 (80.99-136.75) | 0.15 (0.10-0.23) | 0.26 (0.20-0.34) | 1.41 (0.76,2.07) |  | 40.74 (26.53-61.18) | 107.54 (82.58-137.70) | 0.16 (0.10-0.23) | 0.27 (0.21-0.34) | 1.38 (0.73,2.03) |  | 1559.00 (1035.58-2298.52) | 3776.24 (2850.47-4912.81) | 5.95 (3.95-8.77) | 9.40 (7.10-12.24) | 1.12 (0.39,1.85) |
| Tropical Latin America | 42.15 (36.90-48.48) | 107.43 (91.96-125.15) | 0.06 (0.05-0.06) | 0.09 (0.08-0.11) | 2.36 (2.10,2.62) |  | 43.36 (37.98-49.78) | 111.36 (95.06-130.02) | 0.06 (0.05-0.07) | 0.10 (0.08-0.11) | 2.47 (2.22,2.73) |  | 1517.98 (1333.53-1721.23) | 3266.30 (2847.79-3786.09) | 1.99 (1.75-2.26) | 2.87 (2.50-3.33) | 1.90 (1.65,2.16) |
| Western Europe | 379.66 (286.87-488.30) | 907.80 (666.28-1179.24) | 0.20 (0.15-0.25) | 0.42 (0.30-0.54) | 2.53 (2.41,2.66) |  | 378.58 (283.09-493.90) | 813.56 (587.23-1069.81) | 0.20 (0.15-0.26) | 0.37 (0.27-0.49) | 2.15 (2.06,2.24) |  | 9536.85 (7475.42-12123.06) | 18160.25 (13639.22-23467.40) | 4.96 (3.89-6.31) | 8.30 (6.24-10.73) | 1.77 (1.67,1.86) |
| Western Sub-Saharan Africa | 248.74 (147.22-413.77) | 452.60 (329.70-616.37) | 0.26 (0.15-0.43) | 0.18 (0.13-0.25) | -1.35 (-1.46,-1.23) |  | 255.47 (150.98-422.41) | 461.56 (333.78-623.08) | 0.26 (0.16-0.44) | 0.19 (0.14-0.25) | -1.36 (-1.47,-1.24) |  | 8730.54 (5257.23-14643.18) | 16200.35 (11859.92-22122.21) | 9.04 (5.44-15.16) | 6.61 (4.84-9.03) | -1.27 (-1.39,-1.14) |

**Abbreviations:** UI, uncertainty interval; ASR, age-standardised rate per 100,000; EAPC, estimated annual percentage change; CI, confidence interval; DALYs, disability-adjusted life-year; SDI, socio-demographic index.

**Table 5. Global burden of liver cancer due to NASH by age group in 1990, 2021 and estimated annual percentage change (EAPC) from 1990 to 2021.**

| **Age group, y** | **Incidence** | | | | |  | **Deaths** | | | | |  | **DALYs** | | | | |
| --- | --- | --- | --- | --- | --- | --- | --- | --- | --- | --- | --- | --- | --- | --- | --- | --- | --- |
|  | **1990 No. (95% UI)** | **2021 No. (95% UI)** | **1990 ASR per 100 000 (95% UI)** | **2021 ASR per 100 000 (95% UI)** | **EAPC (95% CI)** |  | **1990 No. (95% UI)** | **2021 No. (95% UI)** | **1990 ASR per 100 000 (95% UI)** | **2021 ASR per 100 000 (95% UI)** | **EAPC (95% CI)** |  | **1990 No. (95% UI)** | **2021 No. (95% UI)** | **1990 ASR per 100 000 (95% UI)** | **2021 ASR per 100 000 (95% UI)** | **EAPC (95% CI)** |
| 0-4 | / | / | / | / | / |  | / | / | / | / | / |  | / | / | / | / | / |
| 5-9 | / | / | / | / | / |  | / | / | / | / | / |  | / | / | / | / | / |
| 10-14 | / | / | / | / | / |  | / | / | / | / | / |  | / | / | / | / | / |
| 15-19 | 51.02 (36.33-69.01) | 79.29 (55.69-106.51) | 0.02 (0.01-0.03) | 0.03 (0.02-0.03) | 0.66 (0.61,0.72) |  | 49.34 (35.10-67.13) | 73.79 (51.82-100.58) | 0.02 (0.01-0.03) | 0.02 (0.02-0.03) | 0.53 (0.47,0.60) |  | 3592.43 (2555.08-4885.75) | 5372.38 (3772.50-7325.23) | 1.38 (0.98-1.88) | 1.72 (1.21-2.35) | 0.53 (0.47,0.60) |
| 20-24 | 54.84 (40.06-72.60) | 85.01 (61.99-114.02) | 0.02 (0.02-0.03) | 0.03 (0.02-0.04) | 0.37 (0.25,0.49) |  | 55.09 (40.21-73.09) | 81.04 (58.73-110.59) | 0.02 (0.02-0.03) | 0.03 (0.02-0.04) | 0.24 (0.11,0.37) |  | 3741.59 (2730.44-4962.45) | 5504.01 (3990.38-7508.55) | 1.52 (1.11-2.02) | 1.84 (1.34-2.51) | 0.24 (0.11,0.37) |
| 25-29 | 72.97 (47.59-106.51) | 120.82 (80.29-182.75) | 0.03 (0.02-0.05) | 0.04 (0.03-0.06) | 0.25 (0.06,0.44) |  | 66.99 (43.67-98.04) | 103.68 (68.14-157.80) | 0.03 (0.02-0.04) | 0.04 (0.02-0.05) | 0.04 (-0.18,0.25) |  | 4225.60 (2754.91-6186.82) | 6533.81 (4294.56-9944.61) | 1.91 (1.24-2.80) | 2.22 (1.46-3.38) | 0.04 (-0.18,0.25) |
| 30-34 | 105.54 (73.86-148.22) | 196.14 (138.25-273.49) | 0.05 (0.04-0.08) | 0.06 (0.05-0.09) | 0.03 (-0.21,0.28) |  | 95.33 (66.68-133.96) | 162.52 (113.98-227.95) | 0.05 (0.03-0.07) | 0.05 (0.04-0.08) | -0.24 (-0.51,0.03) |  | 5526.39 (3863.45-7771.46) | 9431.70 (6613.55-13233.86) | 2.87 (2.00-4.03) | 3.12 (2.19-4.38) | -0.24 (-0.51,0.03) |
| 35-39 | 178.72 (123.15-248.89) | 314.99 (212.46-439.06) | 0.10 (0.07-0.14) | 0.11 (0.08-0.16) | 0.05 (-0.09,0.19) |  | 152.84 (105.14-213.77) | 247.17 (164.99-347.55) | 0.09 (0.06-0.12) | 0.09 (0.06-0.12) | -0.20 (-0.36,-0.05) |  | 8112.79 (5578.06-11341.21) | 13131.36 (8750.19-18467.69) | 4.61 (3.17-6.44) | 4.68 (3.12-6.59) | -0.20 (-0.36,-0.05) |
| 40-44 | 263.51 (183.84-362.04) | 488.96 (337.39-678.33) | 0.18 (0.13-0.25) | 0.20 (0.13-0.27) | -0.02 (-0.12,0.07) |  | 234.10 (162.92-323.50) | 396.36 (271.48-558.48) | 0.16 (0.11-0.23) | 0.16 (0.11-0.22) | -0.35 (-0.47,-0.23) |  | 11272.50 (7840.93-15596.31) | 19087.40 (13070.47-26895.50) | 7.87 (5.47-10.89) | 7.63 (5.23-10.75) | -0.36 (-0.48,-0.24) |
| 45-49 | 374.97 (253.96-522.16) | 862.96 (603.78-1183.71) | 0.32 (0.22-0.45) | 0.36 (0.26-0.50) | 0.38 (0.21,0.55) |  | 341.12 (230.81-475.87) | 707.98 (490.12-980.06) | 0.29 (0.20-0.41) | 0.30 (0.21-0.41) | 0.00 (-0.19,0.19) |  | 14735.29 (9959.00-20560.14) | 30609.78 (21191.59-42365.19) | 12.69 (8.58-17.71) | 12.93 (8.95-17.89) | 0.00 (-0.19,0.19) |
| 50-54 | 595.16 (410.25-810.30) | 1484.94 (1032.28-2038.62) | 0.56 (0.39-0.76) | 0.67 (0.46-0.92) | 0.70 (0.60,0.80) |  | 548.05 (375.93-748.88) | 1232.67 (853.06-1698.27) | 0.52 (0.35-0.70) | 0.55 (0.38-0.76) | 0.32 (0.22,0.42) |  | 21058.50 (14440.32-28732.94) | 47429.22 (32796.69-65322.12) | 19.81 (13.59-27.03) | 21.32 (14.74-29.36) | 0.32 (0.22,0.43) |
| 55-59 | 834.81 (578.88-1213.19) | 2113.84 (1463.94-3050.81) | 0.90 (0.63-1.31) | 1.07 (0.74-1.54) | 0.58 (0.55,0.61) |  | 791.42 (546.01-1154.49) | 1856.25 (1283.87-2692.98) | 0.85 (0.59-1.25) | 0.94 (0.65-1.36) | 0.33 (0.29,0.36) |  | 26706.18 (18431.75-38941.04) | 62739.22 (43332.67-91100.57) | 28.84 (19.90-42.05) | 31.71 (21.90-46.04) | 0.33 (0.29,0.37) |
| 60-64 | 1048.73 (733.20-1492.77) | 2647.75 (1861.92-3740.93) | 1.31 (0.91-1.86) | 1.65 (1.16-2.34) | 0.73 (0.62,0.85) |  | 1018.48 (708.45-1457.52) | 2384.51 (1675.72-3369.59) | 1.27 (0.88-1.81) | 1.49 (1.05-2.11) | 0.46 (0.34,0.58) |  | 29646.97 (20621.72-42436.20) | 69472.69 (48827.20-98161.16) | 36.92 (25.68-52.84) | 43.41 (30.51-61.34) | 0.47 (0.35,0.59) |
| 65-69 | 1091.97 (738.32-1466.46) | 3197.67 (2232.01-4310.27) | 1.77 (1.19-2.37) | 2.32 (1.62-3.13) | 0.79 (0.68,0.91) |  | 1104.98 (746.67-1490.65) | 3024.26 (2115.52-4076.33) | 1.79 (1.21-2.41) | 2.19 (1.53-2.96) | 0.57 (0.44,0.71) |  | 27109.95 (18326.36-36571.52) | 74256.48 (51960.74-99963.56) | 43.86 (29.65-59.17) | 53.84 (37.67-72.48) | 0.58 (0.44,0.71) |
| 70-74 | 958.85 (651.39-1310.32) | 3006.65 (2111.67-3966.34) | 2.27 (1.54-3.10) | 2.92 (2.05-3.85) | 0.74 (0.64,0.85) |  | 1018.71 (687.56-1396.04) | 2958.52 (2077.51-3915.42) | 2.41 (1.62-3.30) | 2.87 (2.02-3.80) | 0.54 (0.44,0.63) |  | 20579.32 (13864.18-28274.56) | 59860.56 (41992.17-79153.05) | 48.62 (32.75-66.79) | 58.16 (40.80-76.91) | 0.54 (0.44,0.63) |
| 75-79 | 807.47 (561.73-1114.99) | 2574.98 (1852.16-3483.63) | 2.62 (1.83-3.62) | 3.90 (2.81-5.28) | 1.20 (1.07,1.33) |  | 910.47 (631.32-1261.32) | 2697.72 (1936.93-3643.88) | 2.96 (2.05-4.10) | 4.09 (2.94-5.53) | 0.99 (0.87,1.10) |  | 14722.08 (10206.87-20380.75) | 43615.38 (31350.92-59077.16) | 47.83 (33.16-66.22) | 66.14 (47.54-89.59) | 0.98 (0.86,1.10) |
| 80-84 | 481.61 (331.46-686.02) | 2087.74 (1457.06-2862.40) | 2.72 (1.87-3.88) | 4.77 (3.33-6.54) | 2.09 (1.91,2.26) |  | 581.53 (400.54-828.77) | 2341.86 (1637.94-3196.28) | 3.29 (2.26-4.69) | 5.35 (3.74-7.30) | 1.87 (1.71,2.02) |  | 7381.03 (5079.23-10507.80) | 29669.09 (20742.60-40553.66) | 41.73 (28.72-59.41) | 67.75 (47.37-92.61) | 1.86 (1.70,2.02) |
| 85-89 | 222.14 (153.02-314.39) | 1335.83 (913.92-1817.21) | 2.94 (2.03-4.16) | 5.84 (4.00-7.95) | 2.36 (2.25,2.46) |  | 274.28 (189.54-390.14) | 1415.34 (984.62-1924.73) | 3.63 (2.51-5.16) | 6.19 (4.31-8.42) | 1.93 (1.83,2.03) |  | 2772.25 (1912.80-3940.40) | 14300.30 (9929.13-19417.56) | 36.69 (25.32-52.15) | 62.55 (43.43-84.94) | 1.92 (1.83,2.02) |
| 90-94 | 54.84 (35.45-80.09) | 438.24 (281.10-627.24) | 2.56 (1.65-3.74) | 4.90 (3.14-7.01) | 2.17 (2.07,2.27) |  | 79.24 (50.95-116.10) | 608.26 (391.62-869.97) | 3.70 (2.38-5.42) | 6.80 (4.38-9.73) | 2.04 (1.94,2.15) |  | 694.64 (446.16-1016.55) | 5333.29 (3440.72-7626.00) | 32.42 (20.82-47.45) | 59.63 (38.47-85.26) | 2.04 (1.94,2.15) |
| ≥95 | 9.81 (5.39-15.56) | 109.87 (60.99-176.94) | 1.93 (1.06-3.06) | 4.03 (2.24-6.49) | 2.35 (2.20,2.50) |  | 15.60 (8.61-24.80) | 170.53 (94.58-276.08) | 3.07 (1.69-4.87) | 6.26 (3.47-10.13) | 2.25 (2.09,2.41) |  | 128.79 (71.14-204.20) | 1390.61 (773.16-2256.73) | 25.30 (13.97-40.11) | 51.03 (28.37-82.81) | 2.19 (2.03,2.35) |

**Abbreviations:** DALYs, disability-adjusted life-year; NASH, non-alcoholic steatohepatitis (NASH); UI, uncertainty interval.

**Table 6. Global burden of liver cancer due to hepatitis B by age group in 1990, 2021 and estimated annual percentage change (EAPC) from 1990 to 2021.**

| **Age group, y** | **Incidence** | | | | |  | **Deaths** | | | | |  | **DALYs** | | | | |
| --- | --- | --- | --- | --- | --- | --- | --- | --- | --- | --- | --- | --- | --- | --- | --- | --- | --- |
|  | **1990 No. (95% UI)** | **2021 No. (95% UI)** | **1990 ASR per 100 000 (95% UI)** | **2021 ASR per 100 000 (95% UI)** | **EAPC (95% CI)** |  | **1990 No. (95% UI)** | **2021 No. (95% UI)** | **1990 ASR per 100 000 (95% UI)** | **2021 ASR per 100 000 (95% UI)** | **EAPC (95% CI)** |  | **1990 No. (95% UI)** | **2021 No. (95% UI)** | **1990 ASR per 100 000 (95% UI)** | **2021 ASR per 100 000 (95% UI)** | **EAPC (95% CI)** |
| 0-4 | / | / | / | / | / |  | / | / | / | / | / |  | / | / | / | / | / |
| 5-9 | / | / | / | / | / |  | / | / | / | / | / |  | / | / | / | / | / |
| 10-14 | 166.40 (139.77-198.88) | 102.86 (81.22-131.22) | 0.06 (0.05-0.07) | 0.03 (0.02-0.04) | -2.82 (-3.19,-2.46) |  | 185.75 (158.80-218.19) | 116.03 (94.70-144.69) | 0.07 (0.06-0.08) | 0.03 (0.03-0.04) | -2.76 (-3.12,-2.39) |  | 14441.03 (12346.75-16966.02) | 9015.97 (7361.73-11242.39) | 5.39 (4.61-6.33) | 2.70 (2.21-3.37) | -2.76 (-3.12,-2.40) |
| 15-19 | 279.81 (236.44-336.01) | 217.92 (180.32-265.80) | 0.11 (0.09-0.13) | 0.07 (0.06-0.09) | -1.60 (-1.78,-1.42) |  | 271.87 (229.62-326.57) | 198.76 (163.03-243.07) | 0.10 (0.09-0.13) | 0.06 (0.05-0.08) | -1.81 (-2.00,-1.61) |  | 19787.44 (16718.95-23779.62) | 14468.60 (11866.73-17674.93) | 7.62 (6.44-9.16) | 4.64 (3.80-5.67) | -1.81 (-2.00,-1.61) |
| 20-24 | 524.62 (446.50-622.97) | 411.90 (355.90-482.90) | 0.21 (0.18-0.25) | 0.14 (0.12-0.16) | -2.00 (-2.19,-1.81) |  | 529.73 (449.63-630.33) | 382.95 (328.70-448.75) | 0.22 (0.18-0.26) | 0.13 (0.11-0.15) | -2.21 (-2.41,-2.01) |  | 35987.07 (30541.38-42807.34) | 26003.09 (22309.24-30467.29) | 14.63 (12.41-17.40) | 8.71 (7.47-10.20) | -2.21 (-2.41,-2.02) |
| 25-29 | 1087.50 (911.50-1313.36) | 1119.91 (934.16-1328.59) | 0.49 (0.41-0.59) | 0.38 (0.32-0.45) | -1.54 (-1.99,-1.09) |  | 1002.94 (842.44-1212.40) | 927.32 (771.46-1100.88) | 0.45 (0.38-0.55) | 0.32 (0.26-0.37) | -1.87 (-2.35,-1.40) |  | 63294.42 (53153.68-76536.33) | 58419.55 (48622.44-69401.29) | 28.60 (24.02-34.58) | 19.86 (16.53-23.59) | -1.87 (-2.35,-1.40) |
| 30-34 | 2047.97 (1748.92-2445.30) | 2704.75 (2274.47-3289.81) | 1.06 (0.91-1.27) | 0.89 (0.75-1.09) | -1.50 (-1.93,-1.06) |  | 1860.77 (1585.60-2219.32) | 2149.25 (1813.27-2612.75) | 0.97 (0.82-1.15) | 0.71 (0.60-0.86) | -1.92 (-2.41,-1.43) |  | 107732.20 (91745.34-128454.04) | 124642.71 (105174.85-151634.25) | 55.90 (47.61-66.66) | 41.24 (34.80-50.17) | -1.91 (-2.40,-1.42) |
| 35-39 | 3662.03 (3089.08-4339.37) | 4365.04 (3589.08-5382.36) | 2.08 (1.75-2.46) | 1.56 (1.28-1.92) | -1.41 (-1.70,-1.13) |  | 3151.90 (2663.48-3752.77) | 3282.28 (2696.45-4045.49) | 1.79 (1.51-2.13) | 1.17 (0.96-1.44) | -1.81 (-2.12,-1.49) |  | 167265.10 (141344.77-199228.33) | 174455.08 (143365.29-215091.29) | 94.97 (80.25-113.12) | 62.21 (51.12-76.70) | -1.80 (-2.12,-1.49) |
| 40-44 | 5062.60 (4222.60-5965.42) | 6351.91 (5054.09-7712.09) | 3.53 (2.95-4.16) | 2.54 (2.02-3.08) | -1.22 (-1.38,-1.06) |  | 4532.00 (3762.72-5338.89) | 4889.30 (3897.43-5960.31) | 3.16 (2.63-3.73) | 1.95 (1.56-2.38) | -1.76 (-1.97,-1.55) |  | 218165.59 (181097.03-256888.19) | 235398.94 (187707.08-286553.82) | 152.31 (126.43-179.34) | 94.11 (75.05-114.56) | -1.77 (-1.97,-1.56) |
| 45-49 | 5830.75 (4887.30-6921.68) | 10269.11 (8139.43-12890.76) | 5.02 (4.21-5.96) | 4.34 (3.44-5.44) | -0.33 (-0.63,-0.02) |  | 5340.00 (4465.80-6370.26) | 7980.18 (6357.04-9993.24) | 4.60 (3.85-5.49) | 3.37 (2.69-4.22) | -0.94 (-1.26,-0.63) |  | 230635.63 (193059.67-275202.94) | 344695.08 (274665.26-431844.36) | 198.66 (166.29-237.04) | 145.59 (116.01-182.40) | -0.94 (-1.26,-0.63) |
| 50-54 | 7150.38 (5920.68-8523.04) | 13935.07 (10775.85-17916.60) | 6.73 (5.57-8.02) | 6.26 (4.84-8.05) | -0.03 (-0.22,0.16) |  | 6637.17 (5494.74-7920.86) | 11018.11 (8495.11-14137.89) | 6.24 (5.17-7.45) | 4.95 (3.82-6.36) | -0.64 (-0.86,-0.42) |  | 254872.30 (211094.19-303873.55) | 423839.02 (326261.40-543706.26) | 239.80 (198.61-285.90) | 190.52 (146.66-244.40) | -0.64 (-0.86,-0.41) |
| 55-59 | 7634.93 (6211.84-9251.12) | 14262.48 (10959.27-18727.60) | 8.25 (6.71-9.99) | 7.21 (5.54-9.46) | -0.57 (-0.71,-0.43) |  | 7321.91 (5939.19-8881.97) | 12105.11 (9280.19-15875.80) | 7.91 (6.41-9.59) | 6.12 (4.69-8.02) | -0.97 (-1.14,-0.79) |  | 247139.69 (200471.88-299585.09) | 409378.44 (313863.22-537273.25) | 266.89 (216.49-323.53) | 206.90 (158.63-271.54) | -0.96 (-1.13,-0.78) |
| 60-64 | 7199.75 (5562.69-8926.85) | 12896.39 (9085.00-17174.09) | 8.97 (6.93-11.12) | 8.06 (5.68-10.73) | -0.24 (-0.41,-0.08) |  | 7088.36 (5472.63-8800.69) | 11295.78 (7973.95-15193.10) | 8.83 (6.81-10.96) | 7.06 (4.98-9.49) | -0.67 (-0.82,-0.52) |  | 206351.70 (159424.35-256250.93) | 328859.73 (232018.76-442310.79) | 256.96 (198.52-319.10) | 205.51 (144.99-276.40) | -0.67 (-0.82,-0.51) |
| 65-69 | 5945.11 (4610.89-7335.48) | 13093.38 (9697.99-16512.58) | 9.62 (7.46-11.87) | 9.49 (7.03-11.97) | -0.08 (-0.25,0.09) |  | 6105.60 (4726.98-7545.19) | 12090.46 (8952.32-15275.82) | 9.88 (7.65-12.21) | 8.77 (6.49-11.08) | -0.46 (-0.65,-0.26) |  | 149700.77 (115898.98-184771.73) | 296674.84 (219541.76-374542.16) | 242.22 (187.52-298.96) | 215.10 (159.18-271.56) | -0.45 (-0.65,-0.25) |
| 70-74 | 4141.88 (3096.25-5336.80) | 9575.14 (6893.36-12360.00) | 9.78 (7.31-12.61) | 9.30 (6.70-12.01) | -0.31 (-0.47,-0.14) |  | 4471.75 (3342.68-5786.94) | 9229.77 (6608.96-11871.03) | 10.56 (7.90-13.67) | 8.97 (6.42-11.53) | -0.67 (-0.85,-0.49) |  | 90299.00 (67561.75-116835.88) | 186627.78 (133528.12-240527.32) | 213.32 (159.61-276.01) | 181.33 (129.74-233.70) | -0.67 (-0.85,-0.49) |
| 75-79 | 2449.27 (1883.38-3067.92) | 6407.60 (4875.16-8090.46) | 7.96 (6.12-9.97) | 9.72 (7.39-12.27) | 0.48 (0.27,0.69) |  | 2806.23 (2150.58-3522.08) | 6593.37 (5025.18-8321.52) | 9.12 (6.99-11.44) | 10.00 (7.62-12.62) | 0.13 (-0.09,0.36) |  | 45463.20 (34824.13-57063.51) | 106592.06 (81002.43-134744.66) | 147.71 (113.15-185.41) | 161.64 (122.84-204.34) | 0.12 (-0.10,0.35) |
| 80-84 | 1063.96 (772.73-1410.28) | 4338.79 (3103.57-5594.20) | 6.02 (4.37-7.97) | 9.91 (7.09-12.77) | 2.15 (1.87,2.44) |  | 1306.30 (957.39-1734.50) | 4790.77 (3410.76-6206.31) | 7.39 (5.41-9.81) | 10.94 (7.79-14.17) | 1.78 (1.50,2.06) |  | 16605.99 (12190.45-22035.81) | 60674.16 (43185.58-78787.44) | 93.88 (68.92-124.58) | 138.55 (98.62-179.91) | 1.77 (1.49,2.05) |
| 85-89 | 407.76 (303.81-542.25) | 2398.79 (1792.06-3163.00) | 5.40 (4.02-7.18) | 10.49 (7.84-13.84) | 2.37 (2.24,2.49) |  | 520.33 (388.94-693.08) | 2513.38 (1860.25-3328.66) | 6.89 (5.15-9.17) | 10.99 (8.14-14.56) | 1.75 (1.60,1.90) |  | 5264.37 (3929.03-7017.99) | 25387.22 (18786.29-33709.72) | 69.68 (52.00-92.89) | 111.05 (82.18-147.46) | 1.75 (1.60,1.89) |
| 90-94 | 75.95 (51.43-113.28) | 623.87 (427.54-878.00) | 3.54 (2.40-5.29) | 6.97 (4.78-9.82) | 2.32 (2.14,2.51) |  | 110.53 (74.88-164.80) | 866.57 (595.68-1224.22) | 5.16 (3.49-7.69) | 9.69 (6.66-13.69) | 2.14 (1.96,2.32) |  | 969.40 (657.18-1442.01) | 7600.19 (5223.10-10744.69) | 45.24 (30.67-67.30) | 84.97 (58.39-120.12) | 2.14 (1.96,2.32) |
| ≥95 | 8.68 (4.52-15.18) | 107.94 (58.20-176.01) | 1.71 (0.89-2.98) | 3.96 (2.14-6.46) | 2.94 (2.73,3.15) |  | 13.88 (7.25-24.50) | 167.78 (91.62-274.39) | 2.73 (1.42-4.81) | 6.16 (3.36-10.07) | 2.78 (2.56,3.00) |  | 114.80 (59.93-202.17) | 1367.18 (747.62-2238.07) | 22.55 (11.77-39.72) | 50.17 (27.43-82.13) | 2.72 (2.49,2.95) |

**Abbreviations:** DALYs, disability-adjusted life-year; UI, uncertainty interval.

**Table 7. Global burden of liver cancer due to hepatitis C by age group in 1990, 2021 and estimated annual percentage change (EAPC) from 1990 to 2021.**

| **Age group, y** | **Incidence** | | | | |  | **Deaths** | | | | |  | **DALYs** | | | | |
| --- | --- | --- | --- | --- | --- | --- | --- | --- | --- | --- | --- | --- | --- | --- | --- | --- | --- |
|  | **1990 No. (95% UI)** | **2021 No. (95% UI)** | **1990 ASR per 100 000 (95% UI)** | **2021 ASR per 100 000 (95% UI)** | **EAPC (95% CI)** |  | **1990 No. (95% UI)** | **2021 No. (95% UI)** | **1990 ASR per 100 000 (95% UI)** | **2021 ASR per 100 000 (95% UI)** | **EAPC (95% CI)** |  | **1990 No. (95% UI)** | **2021 No. (95% UI)** | **1990 ASR per 100 000 (95% UI)** | **2021 ASR per 100 000 (95% UI)** | **EAPC (95% CI)** |
| 0-4 | / | / | / | / | / |  | / | / | / | / | / |  | / | / | / | / | / |
| 5-9 | / | / | / | / | / |  | / | / | / | / | / |  | / | / | / | / | / |
| 10-14 | 4.32 (2.47-6.96) | 4.17 (2.30-6.65) | 0.00 (0.00-0.00) | 0.00 (0.00-0.00) | -0.86 (-0.97,-0.74) |  | 5.27 (2.97-8.44) | 5.37 (2.99-8.45) | 0.00 (0.00-0.00) | 0.00 (0.00-0.00) | -0.60 (-0.70,-0.50) |  | 409.72 (231.09-656.61) | 417.56 (232.16-656.71) | 0.15 (0.09-0.25) | 0.13 (0.07-0.20) | -0.60 (-0.70,-0.50) |
| 15-19 | 7.21 (3.99-11.66) | 8.93 (4.72-15.07) | 0.00 (0.00-0.00) | 0.00 (0.00-0.00) | -0.00 (-0.05,0.05) |  | 6.88 (3.77-11.19) | 8.24 (4.28-14.08) | 0.00 (0.00-0.00) | 0.00 (0.00-0.00) | -0.09 (-0.15,-0.04) |  | 500.65 (274.30-814.98) | 600.30 (311.29-1024.60) | 0.19 (0.11-0.31) | 0.19 (0.10-0.33) | -0.09 (-0.15,-0.04) |
| 20-24 | 19.04 (12.69-28.36) | 23.85 (15.22-36.27) | 0.01 (0.01-0.01) | 0.01 (0.01-0.01) | -0.24 (-0.35,-0.13) |  | 18.90 (12.56-28.37) | 22.51 (14.26-34.61) | 0.01 (0.01-0.01) | 0.01 (0.00-0.01) | -0.36 (-0.48,-0.24) |  | 1283.80 (852.89-1926.91) | 1528.80 (967.85-2351.16) | 0.52 (0.35-0.78) | 0.51 (0.32-0.79) | -0.36 (-0.48,-0.24) |
| 25-29 | 47.74 (27.04-79.13) | 61.27 (32.81-103.78) | 0.02 (0.01-0.04) | 0.02 (0.01-0.04) | -0.49 (-0.64,-0.35) |  | 43.30 (24.50-72.35) | 51.70 (27.13-88.57) | 0.02 (0.01-0.03) | 0.02 (0.01-0.03) | -0.72 (-0.89,-0.54) |  | 2732.02 (1545.20-4561.03) | 3258.70 (1709.53-5577.25) | 1.23 (0.70-2.06) | 1.11 (0.58-1.90) | -0.72 (-0.89,-0.54) |
| 30-34 | 118.99 (81.83-163.25) | 174.20 (118.91-244.37) | 0.06 (0.04-0.08) | 0.06 (0.04-0.08) | -0.64 (-0.83,-0.44) |  | 105.93 (72.42-146.27) | 141.69 (95.59-201.16) | 0.05 (0.04-0.08) | 0.05 (0.03-0.07) | -0.93 (-1.15,-0.70) |  | 6141.38 (4197.42-8481.91) | 8223.93 (5551.16-11676.28) | 3.19 (2.18-4.40) | 2.72 (1.84-3.86) | -0.92 (-1.15,-0.70) |
| 35-39 | 313.71 (213.15-439.07) | 434.46 (289.97-635.95) | 0.18 (0.12-0.25) | 0.15 (0.10-0.23) | -0.62 (-0.76,-0.47) |  | 262.35 (176.89-370.31) | 333.18 (218.66-487.87) | 0.15 (0.10-0.21) | 0.12 (0.08-0.17) | -0.88 (-1.03,-0.73) |  | 13923.23 (9391.32-19654.35) | 17701.71 (11623.26-25957.45) | 7.91 (5.33-11.16) | 6.31 (4.14-9.26) | -0.88 (-1.03,-0.73) |
| 40-44 | 637.39 (464.23-853.45) | 906.36 (631.17-1237.90) | 0.44 (0.32-0.60) | 0.36 (0.25-0.49) | -0.86 (-0.94,-0.77) |  | 545.73 (393.48-734.83) | 713.86 (497.24-985.09) | 0.38 (0.27-0.51) | 0.29 (0.20-0.39) | -1.16 (-1.26,-1.05) |  | 26288.37 (18957.74-35362.56) | 34383.47 (23984.42-47442.79) | 18.35 (13.23-24.69) | 13.75 (9.59-18.97) | -1.16 (-1.27,-1.05) |
| 45-49 | 1148.99 (827.11-1567.91) | 1953.95 (1354.23-2658.39) | 0.99 (0.71-1.35) | 0.83 (0.57-1.12) | -0.72 (-0.88,-0.56) |  | 1001.65 (718.36-1386.02) | 1554.43 (1075.72-2135.79) | 0.86 (0.62-1.19) | 0.66 (0.45-0.90) | -1.01 (-1.18,-0.85) |  | 43259.71 (31014.77-59864.89) | 67222.04 (46517.45-92424.40) | 37.26 (26.71-51.56) | 28.39 (19.65-39.04) | -1.01 (-1.18,-0.85) |
| 50-54 | 2248.94 (1699.30-2940.03) | 4004.87 (2907.32-5207.83) | 2.12 (1.60-2.77) | 1.80 (1.31-2.34) | -0.58 (-0.74,-0.42) |  | 1973.62 (1471.30-2594.79) | 3217.76 (2324.29-4221.65) | 1.86 (1.38-2.44) | 1.45 (1.04-1.90) | -0.81 (-0.96,-0.65) |  | 75831.76 (56513.55-99662.93) | 123831.40 (89334.58-162679.83) | 71.35 (53.17-93.77) | 55.66 (40.16-73.13) | -0.81 (-0.96,-0.65) |
| 55-59 | 3913.22 (3041.27-4885.98) | 6485.15 (4673.05-8440.21) | 4.23 (3.28-5.28) | 3.28 (2.36-4.27) | -0.88 (-0.98,-0.77) |  | 3494.30 (2681.62-4442.12) | 5517.56 (3972.73-7232.36) | 3.77 (2.90-4.80) | 2.79 (2.01-3.66) | -0.97 (-1.05,-0.89) |  | 117901.62 (90569.10-149986.92) | 186499.80 (134247.07-244785.66) | 127.32 (97.81-161.97) | 94.26 (67.85-123.71) | -0.96 (-1.05,-0.88) |
| 60-64 | 5252.09 (4143.96-6533.08) | 8825.38 (6508.81-11433.71) | 6.54 (5.16-8.14) | 5.52 (4.07-7.15) | -0.89 (-1.04,-0.74) |  | 4827.16 (3777.41-6090.13) | 7673.65 (5592.91-9964.73) | 6.01 (4.70-7.58) | 4.80 (3.50-6.23) | -1.00 (-1.14,-0.86) |  | 140532.24 (109963.30-177522.98) | 223600.86 (163193.62-290631.93) | 175.00 (136.93-221.06) | 139.73 (101.98-181.62) | -1.00 (-1.14,-0.86) |
| 65-69 | 5338.24 (4381.22-6424.17) | 11226.04 (9049.77-13561.51) | 8.64 (7.09-10.39) | 8.14 (6.56-9.83) | -0.69 (-0.93,-0.46) |  | 5191.87 (4255.15-6295.33) | 10220.25 (8260.04-12448.78) | 8.40 (6.88-10.19) | 7.41 (5.99-9.03) | -0.82 (-1.06,-0.58) |  | 127435.09 (104441.84-154583.62) | 250910.34 (202700.17-305542.87) | 206.19 (168.99-250.12) | 181.92 (146.97-221.53) | -0.82 (-1.06,-0.58) |
| 70-74 | 4673.42 (3854.10-5628.40) | 11383.50 (9333.96-13558.35) | 11.04 (9.10-13.30) | 11.06 (9.07-13.17) | -0.40 (-0.72,-0.08) |  | 4801.65 (3916.23-5829.45) | 10655.47 (8750.58-12775.30) | 11.34 (9.25-13.77) | 10.35 (8.50-12.41) | -0.62 (-0.90,-0.33) |  | 96926.16 (79163.98-117880.25) | 215484.45 (177281.15-258745.73) | 228.97 (187.01-278.48) | 209.37 (172.25-251.41) | -0.62 (-0.90,-0.33) |
| 75-79 | 4057.86 (3352.22-4774.55) | 10387.89 (8365.16-12376.27) | 13.18 (10.89-15.51) | 15.75 (12.69-18.77) | 0.28 (-0.03,0.60) |  | 4464.27 (3676.24-5291.19) | 10328.89 (8319.78-12471.17) | 14.50 (11.94-17.19) | 15.66 (12.62-18.91) | 0.02 (-0.26,0.30) |  | 72119.21 (59446.57-85444.77) | 166730.85 (134310.25-201292.93) | 234.32 (193.15-277.62) | 252.84 (203.68-305.26) | 0.02 (-0.26,0.30) |
| 80-84 | 2602.61 (2159.45-3071.85) | 9618.29 (7871.14-11295.28) | 14.71 (12.21-17.37) | 21.96 (17.97-25.79) | 1.39 (1.11,1.68) |  | 3059.19 (2532.67-3623.57) | 10216.27 (8358.65-11994.40) | 17.30 (14.32-20.49) | 23.33 (19.09-27.39) | 1.14 (0.89,1.38) |  | 38805.93 (32094.89-46105.80) | 129306.25 (105811.82-151892.41) | 219.39 (181.45-260.66) | 295.28 (241.63-346.85) | 1.13 (0.88,1.37) |
| 85-89 | 1391.72 (1161.43-1624.62) | 7960.32 (6264.99-9315.51) | 18.42 (15.37-21.50) | 34.82 (27.40-40.75) | 1.97 (1.79,2.16) |  | 1663.04 (1392.33-1957.64) | 7632.11 (6070.14-8978.49) | 22.01 (18.43-25.91) | 33.39 (26.55-39.27) | 1.40 (1.25,1.55) |  | 16803.61 (14028.96-19746.17) | 77065.42 (61425.85-90308.45) | 222.40 (185.68-261.35) | 337.11 (268.69-395.03) | 1.40 (1.25,1.55) |
| 90-94 | 351.52 (274.14-421.18) | 2795.05 (2067.24-3348.48) | 16.41 (12.79-19.66) | 31.25 (23.11-37.44) | 1.99 (1.90,2.09) |  | 505.31 (397.28-605.69) | 3777.51 (2799.94-4532.03) | 23.58 (18.54-28.27) | 42.23 (31.30-50.67) | 1.85 (1.77,1.94) |  | 4427.35 (3469.03-5313.47) | 33076.49 (24555.93-39665.91) | 206.64 (161.91-247.99) | 369.79 (274.53-443.46) | 1.85 (1.77,1.93) |
| ≥95 | 59.45 (42.59-73.51) | 777.09 (528.52-950.95) | 11.68 (8.37-14.44) | 28.52 (19.39-34.90) | 2.85 (2.73,2.97) |  | 94.58 (68.59-116.98) | 1190.54 (808.11-1454.97) | 18.58 (13.47-22.98) | 43.69 (29.65-53.39) | 2.75 (2.63,2.86) |  | 780.93 (566.30-965.91) | 9592.86 (6533.03-11716.89) | 153.41 (111.25-189.75) | 352.01 (239.73-429.95) | 2.67 (2.55,2.78) |

**Abbreviations:** DALYs, disability-adjusted life-year; UI, uncertainty interval.

**Table 8. Global burden of liver cancer due to alcohol use by age group in 1990, 2021 and estimated annual percentage change (EAPC) from 1990 to 2021.**

| **Age group, y** | **Incidence** | | | | |  | **Deaths** | | | | |  | **DALYs** | | | | |
| --- | --- | --- | --- | --- | --- | --- | --- | --- | --- | --- | --- | --- | --- | --- | --- | --- | --- |
|  | **1990 No. (95% UI)** | **2021 No. (95% UI)** | **1990 ASR per 100 000 (95% UI)** | **2021 ASR per 100 000 (95% UI)** | **EAPC (95% CI)** |  | **1990 No. (95% UI)** | **2021 No. (95% UI)** | **1990 ASR per 100 000 (95% UI)** | **2021 ASR per 100 000 (95% UI)** | **EAPC (95% CI)** |  | **1990 No. (95% UI)** | **2021 No. (95% UI)** | **1990 ASR per 100 000 (95% UI)** | **2021 ASR per 100 000 (95% UI)** | **EAPC (95% CI)** |
| 0-4 | / | / | / | / | / |  | / | / | / | / | / |  | / | / | / | / | / |
| 5-9 | / | / | / | / | / |  | / | / | / | / | / |  | / | / | / | / | / |
| 10-14 | / | / | / | / | / |  | / | / | / | / | / |  | / | / | / | / | / |
| 15-19 | 4.73 (1.99-8.90) | 5.83 (2.55-11.11) | 0.00 (0.00-0.00) | 0.00 (0.00-0.00) | -0.12 (-0.18,-0.06) |  | 4.49 (1.87-8.50) | 5.26 (2.27-10.20) | 0.00 (0.00-0.00) | 0.00 (0.00-0.00) | -0.28 (-0.35,-0.22) |  | 326.97 (136.15-619.07) | 383.23 (164.99-742.29) | 0.13 (0.05-0.24) | 0.12 (0.05-0.24) | -0.28 (-0.35,-0.22) |
| 20-24 | 19.82 (12.95-30.01) | 26.38 (17.01-40.42) | 0.01 (0.01-0.01) | 0.01 (0.01-0.01) | -0.10 (-0.22,0.01) |  | 19.49 (12.73-29.75) | 24.18 (15.47-37.57) | 0.01 (0.01-0.01) | 0.01 (0.01-0.01) | -0.32 (-0.44,-0.19) |  | 1323.77 (863.61-2019.73) | 1641.81 (1050.11-2552.39) | 0.54 (0.35-0.82) | 0.55 (0.35-0.85) | -0.32 (-0.44,-0.19) |
| 25-29 | 57.98 (34.13-90.85) | 86.64 (51.24-139.32) | 0.03 (0.02-0.04) | 0.03 (0.02-0.05) | -0.06 (-0.21,0.09) |  | 52.05 (30.41-82.01) | 71.29 (41.55-116.87) | 0.02 (0.01-0.04) | 0.02 (0.01-0.04) | -0.34 (-0.52,-0.17) |  | 3283.06 (1919.62-5172.77) | 4493.72 (2618.95-7379.73) | 1.48 (0.87-2.34) | 1.53 (0.89-2.51) | -0.34 (-0.52,-0.17) |
| 30-34 | 146.18 (100.46-213.28) | 244.32 (170.15-344.57) | 0.08 (0.05-0.11) | 0.08 (0.06-0.11) | -0.28 (-0.46,-0.09) |  | 129.39 (88.18-189.93) | 196.33 (135.84-278.29) | 0.07 (0.05-0.10) | 0.06 (0.04-0.09) | -0.59 (-0.80,-0.38) |  | 7503.06 (5114.35-11006.96) | 11395.74 (7885.68-16155.58) | 3.89 (2.65-5.71) | 3.77 (2.61-5.35) | -0.59 (-0.80,-0.38) |
| 35-39 | 371.21 (235.40-543.23) | 590.30 (379.75-871.93) | 0.21 (0.13-0.31) | 0.21 (0.14-0.31) | -0.33 (-0.45,-0.20) |  | 311.10 (195.98-458.10) | 451.22 (288.74-674.01) | 0.18 (0.11-0.26) | 0.16 (0.10-0.24) | -0.59 (-0.72,-0.46) |  | 16513.30 (10412.19-24328.52) | 23972.80 (15362.17-35791.73) | 9.38 (5.91-13.81) | 8.55 (5.48-12.76) | -0.59 (-0.72,-0.46) |
| 40-44 | 669.19 (440.87-920.51) | 1107.65 (732.40-1562.59) | 0.47 (0.31-0.64) | 0.44 (0.29-0.62) | -0.45 (-0.56,-0.34) |  | 580.74 (380.38-803.72) | 876.82 (575.83-1245.92) | 0.41 (0.27-0.56) | 0.35 (0.23-0.50) | -0.75 (-0.87,-0.63) |  | 27964.99 (18317.58-38712.67) | 42228.20 (27753.85-59956.24) | 19.52 (12.79-27.03) | 16.88 (11.10-23.97) | -0.75 (-0.87,-0.63) |
| 45-49 | 1003.95 (643.82-1543.30) | 2081.73 (1340.66-3095.21) | 0.86 (0.55-1.33) | 0.88 (0.57-1.31) | -0.03 (-0.17,0.12) |  | 893.05 (567.02-1366.60) | 1669.80 (1061.66-2509.74) | 0.77 (0.49-1.18) | 0.71 (0.45-1.06) | -0.37 (-0.50,-0.24) |  | 38571.53 (24492.45-59039.06) | 72208.36 (45866.04-108595.02) | 33.22 (21.10-50.85) | 30.50 (19.37-45.87) | -0.37 (-0.50,-0.24) |
| 50-54 | 1685.07 (1120.78-2440.35) | 3747.72 (2501.83-5430.87) | 1.59 (1.05-2.30) | 1.68 (1.12-2.44) | 0.34 (0.21,0.48) |  | 1521.34 (1004.69-2210.32) | 3027.15 (2010.69-4419.41) | 1.43 (0.95-2.08) | 1.36 (0.90-1.99) | -0.03 (-0.14,0.08) |  | 58466.86 (38583.90-84966.29) | 116511.09 (77408.63-170130.59) | 55.01 (36.30-79.94) | 52.37 (34.80-76.48) | -0.02 (-0.13,0.08) |
| 55-59 | 2462.87 (1621.00-3663.71) | 5626.40 (3729.33-8407.35) | 2.66 (1.75-3.96) | 2.84 (1.88-4.25) | 0.33 (0.27,0.40) |  | 2295.21 (1500.51-3434.85) | 4817.02 (3172.31-7194.07) | 2.48 (1.62-3.71) | 2.43 (1.60-3.64) | 0.07 (0.01,0.12) |  | 77432.21 (50672.06-115819.80) | 162792.63 (107262.70-243015.06) | 83.62 (54.72-125.08) | 82.28 (54.21-122.82) | 0.07 (0.02,0.13) |
| 60-64 | 3280.43 (2304.08-4553.33) | 7501.49 (5266.44-10454.65) | 4.08 (2.87-5.67) | 4.69 (3.29-6.53) | 0.40 (0.30,0.50) |  | 3134.73 (2186.94-4359.78) | 6565.75 (4564.60-9210.75) | 3.90 (2.72-5.43) | 4.10 (2.85-5.76) | 0.09 (-0.01,0.20) |  | 91262.13 (63690.41-126861.59) | 191368.87 (133211.42-268698.36) | 113.64 (79.31-157.98) | 119.59 (83.24-167.91) | 0.10 (-0.01,0.21) |
| 65-69 | 3563.10 (2705.95-4584.71) | 9230.00 (7091.62-11925.73) | 5.77 (4.38-7.42) | 6.69 (5.14-8.65) | 0.34 (0.24,0.43) |  | 3557.72 (2691.82-4599.09) | 8512.85 (6521.65-11003.13) | 5.76 (4.36-7.44) | 6.17 (4.73-7.98) | 0.08 (-0.03,0.18) |  | 87330.61 (66056.85-112878.79) | 209147.85 (160183.78-270329.89) | 141.30 (106.88-182.64) | 151.64 (116.14-196.00) | 0.08 (-0.03,0.19) |
| 70-74 | 2733.41 (2057.51-3556.66) | 8085.47 (6219.09-10362.05) | 6.46 (4.86-8.40) | 7.86 (6.04-10.07) | 0.44 (0.30,0.58) |  | 2862.95 (2147.25-3738.72) | 7727.09 (5919.12-9947.78) | 6.76 (5.07-8.83) | 7.51 (5.75-9.67) | 0.17 (0.04,0.29) |  | 57864.97 (43335.54-75547.53) | 156298.62 (119819.29-201384.72) | 136.70 (102.37-178.47) | 151.86 (116.42-195.67) | 0.16 (0.04,0.29) |
| 75-79 | 1914.06 (1485.71-2403.08) | 5453.71 (4282.15-6805.93) | 6.22 (4.83-7.81) | 8.27 (6.49-10.32) | 0.97 (0.82,1.12) |  | 2131.74 (1644.75-2680.10) | 5561.16 (4372.64-6931.15) | 6.93 (5.34-8.71) | 8.43 (6.63-10.51) | 0.70 (0.58,0.83) |  | 34441.17 (26571.34-43306.83) | 89920.37 (70876.72-111959.63) | 111.90 (86.33-140.71) | 136.36 (107.48-169.78) | 0.70 (0.57,0.83) |
| 80-84 | 911.40 (682.87-1191.87) | 3557.36 (2623.13-4560.87) | 5.15 (3.86-6.74) | 8.12 (5.99-10.41) | 1.70 (1.55,1.84) |  | 1089.42 (816.46-1428.52) | 3895.25 (2871.77-5024.99) | 6.16 (4.62-8.08) | 8.89 (6.56-11.47) | 1.45 (1.32,1.58) |  | 13836.93 (10381.53-18143.14) | 49365.91 (36473.42-63675.69) | 78.23 (58.69-102.57) | 112.73 (83.29-145.41) | 1.44 (1.31,1.58) |
| 85-89 | 328.10 (243.66-440.56) | 1864.87 (1387.03-2429.33) | 4.34 (3.22-5.83) | 8.16 (6.07-10.63) | 2.13 (2.01,2.24) |  | 398.62 (294.45-538.16) | 1920.07 (1430.82-2514.34) | 5.28 (3.90-7.12) | 8.40 (6.26-11.00) | 1.66 (1.54,1.78) |  | 4033.43 (2995.74-5460.48) | 19415.49 (14418.03-25478.53) | 53.38 (39.65-72.27) | 84.93 (63.07-111.45) | 1.65 (1.54,1.77) |
| 90-94 | 62.61 (43.16-90.28) | 474.27 (327.93-667.00) | 2.92 (2.01-4.21) | 5.30 (3.67-7.46) | 1.89 (1.79,2.00) |  | 90.24 (62.46-130.86) | 656.35 (455.64-926.59) | 4.21 (2.92-6.11) | 7.34 (5.09-10.36) | 1.77 (1.64,1.90) |  | 791.09 (548.05-1151.34) | 5755.79 (4006.93-8112.40) | 36.92 (25.58-53.74) | 64.35 (44.80-90.70) | 1.77 (1.64,1.90) |
| ≥95 | 8.50 (4.96-13.59) | 87.69 (52.29-138.11) | 1.67 (0.97-2.67) | 3.22 (1.92-5.07) | 2.09 (1.97,2.20) |  | 13.52 (7.89-21.70) | 136.29 (80.64-214.96) | 2.66 (1.55-4.26) | 5.00 (2.96-7.89) | 2.00 (1.87,2.13) |  | 111.88 (65.43-179.61) | 1113.02 (656.66-1757.64) | 21.98 (12.85-35.28) | 40.84 (24.10-64.50) | 1.93 (1.80,2.07) |

**Abbreviations:** DALYs, disability-adjusted life-year; UI, uncertainty interval.

**Table 9. Global burden of hepatoblastoma by age group in 1990, 2021 and estimated annual percentage changes (EAPC) from 1990 to 2021**

| **Age group, y** | **Incidence** | | | | |  | **Deaths** | | | | |  | **DALYs** | | | | |
| --- | --- | --- | --- | --- | --- | --- | --- | --- | --- | --- | --- | --- | --- | --- | --- | --- | --- |
|  | **1990 No. (95% UI)** | **2021 No. (95% UI)** | **1990 ASR per 100 000 (95% UI)** | **2021 ASR per 100 000 (95% UI)** | **EAPC (95% CI)** |  | **1990 No. (95% UI)** | **2021 No. (95% UI)** | **1990 ASR per 100 000 (95% UI)** | **2021 ASR per 100 000 (95% UI)** | **EAPC (95% CI)** |  | **1990 No. (95% UI)** | **2021 No. (95% UI)** | **1990 ASR per 100 000 (95% UI)** | **2021 ASR per 100 000 (95% UI)** | **EAPC (95% CI)** |
| 0-4 | 3047.30 (2448.38-3592.12) | 1739.64 (1376.77-2168.89) | 0.98 (0.79-1.16) | 0.53 (0.42-0.66) | -1.98 (-2.08,-1.87) |  | 2039.32 (1622.96-2414.20) | 1002.16 (780.98-1268.73) | 0.66 (0.52-0.78) | 0.30 (0.24-0.39) | -2.52 (-2.62,-2.42) |  | 181908.71 (144876.42-215024.53) | 89553.73 (69875.70-113663.38) | 58.69 (46.74-69.37) | 27.21 (21.23-34.54) | -2.51 (-2.61,-2.41) |
| 5-9 | 484.55 (429.03-541.57) | 284.57 (241.17-342.18) | 0.17 (0.15-0.19) | 0.08 (0.07-0.10) | -2.65 (-2.91,-2.38) |  | 374.82 (331.39-419.09) | 205.92 (173.64-249.52) | 0.13 (0.11-0.14) | 0.06 (0.05-0.07) | -2.87 (-3.13,-2.60) |  | 31273.62 (27651.28-34948.16) | 17185.22 (14477.11-20903.42) | 10.72 (9.48-11.98) | 5.00 (4.21-6.08) | -2.86 (-3.13,-2.60) |
| 10-14 | / | / | / | / | / |  | / | / | / | / | / |  | / | / | / | / | / |
| 15-19 | / | / | / | / | / |  | / | / | / | / | / |  | / | / | / | / | / |
| 20-24 | / | / | / | / | / |  | / | / | / | / | / |  | / | / | / | / | / |
| 25-29 | / | / | / | / | / |  | / | / | / | / | / |  | / | / | / | / | / |
| 30-34 | / | / | / | / | / |  | / | / | / | / | / |  | / | / | / | / | / |
| 35-39 | / | / | / | / | / |  | / | / | / | / | / |  | / | / | / | / | / |
| 40-44 | / | / | / | / | / |  | / | / | / | / | / |  | / | / | / | / | / |
| 45-49 | / | / | / | / | / |  | / | / | / | / | / |  | / | / | / | / | / |
| 50-54 | / | / | / | / | / |  | / | / | / | / | / |  | / | / | / | / | / |
| 55-59 | / | / | / | / | / |  | / | / | / | / | / |  | / | / | / | / | / |
| 60-64 | / | / | / | / | / |  | / | / | / | / | / |  | / | / | / | / | / |
| 65-69 | / | / | / | / | / |  | / | / | / | / | / |  | / | / | / | / | / |
| 70-74 | / | / | / | / | / |  | / | / | / | / | / |  | / | / | / | / | / |
| 75-79 | / | / | / | / | / |  | / | / | / | / | / |  | / | / | / | / | / |
| 80-84 | / | / | / | / | / |  | / | / | / | / | / |  | / | / | / | / | / |
| 85-89 | / | / | / | / | / |  | / | / | / | / | / |  | / | / | / | / | / |
| 90-94 | / | / | / | / | / |  | / | / | / | / | / |  | / | / | / | / | / |
| ≥95 | / | / | / | / | / |  | / | / | / | / | / |  | / | / | / | / | / |

**Abbreviations:** DALYs, disability-adjusted life-year; UI, uncertainty interval.

**Table 10. Global burden of liver cancer due to other causes by age group in 1990, 2021 and estimated annual percentage change (EAPC) from 1990 to 2021**

| **Age group, y** | **Incidence** | | | | |  | **Deaths** | | | | |  | **DALYs** | | | | |
| --- | --- | --- | --- | --- | --- | --- | --- | --- | --- | --- | --- | --- | --- | --- | --- | --- | --- |
|  | **1990 No. (95% UI)** | **2021 No. (95% UI)** | **1990 ASR per 100 000 (95% UI)** | **2021 ASR per 100 000 (95% UI)** | **EAPC (95% CI)** |  | **1990 No. (95% UI)** | **2021 No. (95% UI)** | **1990 ASR per 100 000 (95% UI)** | **2021 ASR per 100 000 (95% UI)** | **EAPC (95% CI)** |  | **1990 No. (95% UI)** | **2021 No. (95% UI)** | **1990 ASR per 100 000 (95% UI)** | **2021 ASR per 100 000 (95% UI)** | **EAPC (95% CI)** |
| 0-4 | / | / | / | / | / |  | / | / | / | / | / |  | / | / | / | / | / |
| 5-9 | / | / | / | / | / |  | / | / | / | / | / |  | / | / | / | / | / |
| 10-14 | 61.57 (47.90-76.66) | 53.62 (40.13-70.66) | 0.02 (0.02-0.03) | 0.02 (0.01-0.02) | -1.43 (-1.64,-1.22) |  | 72.62 (56.52-89.40) | 64.42 (49.06-82.78) | 0.03 (0.02-0.03) | 0.02 (0.01-0.02) | -1.30 (-1.51,-1.10) |  | 5645.80 (4393.38-6951.02) | 5006.22 (3811.49-6431.76) | 2.11 (1.64-2.60) | 1.50 (1.14-1.93) | -1.31 (-1.51,-1.10) |
| 15-19 | 79.68 (59.98-104.78) | 89.34 (63.55-119.85) | 0.03 (0.02-0.04) | 0.03 (0.02-0.04) | -0.38 (-0.44,-0.32) |  | 76.09 (56.89-100.60) | 80.24 (56.18-109.17) | 0.03 (0.02-0.04) | 0.03 (0.02-0.03) | -0.58 (-0.66,-0.50) |  | 5539.10 (4141.18-7320.71) | 5842.34 (4090.09-7941.11) | 2.13 (1.59-2.82) | 1.87 (1.31-2.55) | -0.58 (-0.66,-0.50) |
| 20-24 | 95.27 (70.78-123.55) | 95.41 (70.65-125.47) | 0.04 (0.03-0.05) | 0.03 (0.02-0.04) | -1.10 (-1.25,-0.95) |  | 94.81 (70.13-123.38) | 86.95 (63.23-115.02) | 0.04 (0.03-0.05) | 0.03 (0.02-0.04) | -1.35 (-1.52,-1.18) |  | 6439.34 (4765.05-8379.19) | 5906.02 (4293.57-7809.64) | 2.62 (1.94-3.41) | 1.98 (1.44-2.62) | -1.35 (-1.51,-1.18) |
| 25-29 | 124.61 (85.41-168.91) | 138.49 (91.13-193.10) | 0.06 (0.04-0.08) | 0.05 (0.03-0.07) | -1.16 (-1.51,-0.82) |  | 113.41 (77.42-154.12) | 112.92 (73.11-159.26) | 0.05 (0.03-0.07) | 0.04 (0.02-0.05) | -1.53 (-1.91,-1.15) |  | 7156.39 (4886.03-9727.60) | 7116.57 (4604.98-10038.27) | 3.23 (2.21-4.40) | 2.42 (1.57-3.41) | -1.53 (-1.91,-1.15) |
| 30-34 | 175.82 (124.93-238.90) | 231.90 (159.56-320.28) | 0.09 (0.06-0.12) | 0.08 (0.05-0.11) | -1.31 (-1.70,-0.93) |  | 157.40 (111.71-213.66) | 182.17 (124.24-252.81) | 0.08 (0.06-0.11) | 0.06 (0.04-0.08) | -1.74 (-2.17,-1.30) |  | 9117.18 (6471.43-12378.79) | 10569.66 (7208.34-14682.51) | 4.73 (3.36-6.42) | 3.50 (2.38-4.86) | -1.74 (-2.17,-1.30) |
| 35-39 | 284.83 (192.49-375.08) | 346.93 (237.54-468.15) | 0.16 (0.11-0.21) | 0.12 (0.08-0.17) | -1.22 (-1.43,-1.00) |  | 241.64 (162.78-319.61) | 258.46 (175.65-353.75) | 0.14 (0.09-0.18) | 0.09 (0.06-0.13) | -1.60 (-1.85,-1.36) |  | 12824.69 (8637.30-16955.60) | 13737.35 (9339.70-18805.19) | 7.28 (4.90-9.63) | 4.90 (3.33-6.71) | -1.60 (-1.84,-1.35) |
| 40-44 | 376.71 (265.59-500.39) | 488.08 (339.28-667.53) | 0.26 (0.19-0.35) | 0.20 (0.14-0.27) | -1.18 (-1.31,-1.04) |  | 332.10 (232.42-443.72) | 374.23 (258.47-516.39) | 0.23 (0.16-0.31) | 0.15 (0.10-0.21) | -1.66 (-1.83,-1.48) |  | 15992.89 (11189.63-21374.54) | 18024.02 (12445.69-24872.14) | 11.17 (7.81-14.92) | 7.21 (4.98-9.94) | -1.66 (-1.84,-1.49) |
| 45-49 | 450.32 (328.63-615.47) | 789.20 (552.79-1113.10) | 0.39 (0.28-0.53) | 0.33 (0.23-0.47) | -0.49 (-0.75,-0.24) |  | 406.26 (295.29-556.05) | 614.48 (430.92-872.24) | 0.35 (0.25-0.48) | 0.26 (0.18-0.37) | -1.01 (-1.28,-0.73) |  | 17548.02 (12741.29-24009.13) | 26553.55 (18611.75-37731.26) | 15.11 (10.97-20.68) | 11.22 (7.86-15.94) | -1.01 (-1.28,-0.73) |
| 50-54 | 585.55 (418.64-813.05) | 1185.73 (819.66-1664.10) | 0.55 (0.39-0.76) | 0.53 (0.37-0.75) | 0.07 (-0.11,0.24) |  | 534.42 (380.91-744.61) | 935.70 (639.73-1320.43) | 0.50 (0.36-0.70) | 0.42 (0.29-0.59) | -0.46 (-0.66,-0.25) |  | 20525.25 (14612.85-28616.67) | 35999.84 (24632.94-50770.24) | 19.31 (13.75-26.92) | 16.18 (11.07-22.82) | -0.45 (-0.65,-0.25) |
| 55-59 | 691.84 (504.11-981.79) | 1398.69 (954.53-2005.85) | 0.75 (0.54-1.06) | 0.71 (0.48-1.01) | -0.14 (-0.28,-0.00) |  | 650.94 (471.33-925.89) | 1176.36 (799.29-1686.40) | 0.70 (0.51-1.00) | 0.59 (0.40-0.85) | -0.51 (-0.65,-0.36) |  | 21966.98 (15898.38-31277.25) | 39775.58 (27037.63-57049.65) | 23.72 (17.17-33.78) | 20.10 (13.66-28.83) | -0.50 (-0.65,-0.35) |
| 60-64 | 730.87 (512.59-1046.89) | 1472.49 (1020.26-2116.39) | 0.91 (0.64-1.30) | 0.92 (0.64-1.32) | 0.13 (0.01,0.25) |  | 704.16 (489.97-1011.02) | 1270.89 (878.43-1843.53) | 0.88 (0.61-1.26) | 0.79 (0.55-1.15) | -0.23 (-0.35,-0.12) |  | 20493.85 (14247.52-29402.03) | 37019.29 (25628.73-53551.73) | 25.52 (17.74-36.61) | 23.13 (16.02-33.46) | -0.23 (-0.34,-0.12) |
| 65-69 | 645.73 (458.86-876.13) | 1553.96 (1098.19-2059.40) | 1.04 (0.74-1.42) | 1.13 (0.80-1.49) | 0.19 (0.06,0.32) |  | 648.53 (460.10-884.97) | 1421.93 (1003.62-1886.43) | 1.05 (0.74-1.43) | 1.03 (0.73-1.37) | -0.10 (-0.25,0.05) |  | 15902.39 (11298.52-21654.99) | 34902.67 (24590.89-46307.97) | 25.73 (18.28-35.04) | 25.31 (17.83-33.58) | -0.09 (-0.24,0.06) |
| 70-74 | 489.11 (346.03-657.19) | 1269.45 (908.18-1682.18) | 1.16 (0.82-1.55) | 1.23 (0.88-1.63) | 0.04 (-0.07,0.15) |  | 515.55 (362.66-695.51) | 1209.97 (865.65-1603.53) | 1.22 (0.86-1.64) | 1.18 (0.84-1.56) | -0.22 (-0.34,-0.11) |  | 10409.87 (7324.26-14033.60) | 24469.62 (17531.18-32438.58) | 24.59 (17.30-33.15) | 23.78 (17.03-31.52) | -0.22 (-0.34,-0.11) |
| 75-79 | 359.18 (250.51-476.31) | 956.97 (692.46-1287.42) | 1.17 (0.81-1.55) | 1.45 (1.05-1.95) | 0.51 (0.35,0.67) |  | 401.90 (279.30-535.06) | 976.11 (702.77-1324.98) | 1.31 (0.91-1.74) | 1.48 (1.07-2.01) | 0.26 (0.11,0.41) |  | 6497.58 (4521.80-8646.41) | 15772.50 (11366.60-21400.07) | 21.11 (14.69-28.09) | 23.92 (17.24-32.45) | 0.25 (0.10,0.40) |
| 80-84 | 192.67 (130.28-272.79) | 723.60 (482.63-1000.18) | 1.09 (0.74-1.54) | 1.65 (1.10-2.28) | 1.65 (1.44,1.86) |  | 230.55 (155.63-329.17) | 792.45 (535.06-1100.68) | 1.30 (0.88-1.86) | 1.81 (1.22-2.51) | 1.39 (1.20,1.59) |  | 2924.82 (1974.14-4169.93) | 10033.59 (6786.52-13951.71) | 16.54 (11.16-23.57) | 22.91 (15.50-31.86) | 1.39 (1.19,1.58) |
| 85-89 | 89.09 (61.74-122.76) | 468.39 (311.01-639.24) | 1.18 (0.82-1.62) | 2.05 (1.36-2.80) | 1.86 (1.74,1.97) |  | 108.61 (74.85-151.32) | 478.77 (317.88-656.17) | 1.44 (0.99-2.00) | 2.09 (1.39-2.87) | 1.39 (1.28,1.50) |  | 1097.69 (756.57-1529.60) | 4837.88 (3208.33-6618.76) | 14.53 (10.01-20.24) | 21.16 (14.03-28.95) | 1.39 (1.28,1.50) |
| 90-94 | 20.97 (13.43-30.85) | 147.10 (91.92-210.06) | 0.98 (0.63-1.44) | 1.64 (1.03-2.35) | 1.73 (1.64,1.82) |  | 30.21 (19.49-44.60) | 202.48 (125.38-288.80) | 1.41 (0.91-2.08) | 2.26 (1.40-3.23) | 1.60 (1.50,1.69) |  | 264.83 (171.37-391.37) | 1775.34 (1100.10-2531.36) | 12.36 (8.00-18.27) | 19.85 (12.30-28.30) | 1.60 (1.50,1.69) |
| ≥95 | 3.63 (2.05-6.05) | 36.50 (20.17-59.08) | 0.71 (0.40-1.19) | 1.34 (0.74-2.17) | 1.99 (1.82,2.15) |  | 5.77 (3.24-9.65) | 56.43 (30.96-91.55) | 1.13 (0.64-1.90) | 2.07 (1.14-3.36) | 1.87 (1.69,2.05) |  | 47.64 (26.75-79.56) | 459.57 (252.37-746.85) | 9.36 (5.26-15.63) | 16.86 (9.26-27.41) | 1.82 (1.63,2.00) |

**Abbreviations:** DALYs, disability-adjusted life-year; UI, uncertainty interval.

**Table 11. Burden of liver cancer due to hepatitis C in 204 countries and territories in 1990, 2021 and the estimated annual percentage changes (EAPC) from 1990 to 2021**

| **Country** | **Incidence** | | | | |  | **Deaths** | | | | |  | **DALYs** | | | | |
| --- | --- | --- | --- | --- | --- | --- | --- | --- | --- | --- | --- | --- | --- | --- | --- | --- | --- |
|  | **1990 No. (95% UI)** | **2021 No. (95% UI)** | **1990 ASR per 100 000 (95% UI)** | **2021 ASR per 100 000 (95% UI)** | **EAPC (95% CI)** |  | **1990 No. (95% UI)** | **2021 No. (95% UI)** | **1990 ASR per 100 000 (95% UI)** | **2021 ASR per 100 000 (95% UI)** | **EAPC (95% CI)** |  | **1990 No. (95% UI)** | **2021 No. (95% UI)** | **1990 ASR per 100 000 (95% UI)** | **2021 ASR per 100 000 (95% UI)** | **EAPC (95% CI)** |
| Afghanistan | 79.87 (46.82-132.96) | 115.31 (72.24-181.86) | 1.61 (0.94-2.67) | 0.74 (0.46-1.16) | -2.63 (-2.95,-2.31) |  | 85.47 (50.01-143.02) | 121.73 (76.03-192.46) | 1.72 (1.01-2.88) | 0.78 (0.49-1.23) | -2.64 (-2.95,-2.33) |  | 2212.19 (1325.20-3565.92) | 3398.63 (2118.66-5290.77) | 44.50 (26.65-71.72) | 21.77 (13.57-33.89) | -2.47 (-2.83,-2.11) |
| Albania | 29.59 (18.61-42.44) | 41.58 (23.80-66.60) | 1.79 (1.13-2.57) | 3.12 (1.78-4.99) | 1.71 (1.22,2.20) |  | 33.03 (20.75-47.17) | 46.82 (26.33-74.85) | 2.00 (1.26-2.85) | 3.51 (1.97-5.61) | 1.70 (1.15,2.26) |  | 718.80 (453.32-1044.76) | 909.04 (515.19-1444.26) | 43.49 (27.43-63.22) | 68.15 (38.62-108.27) | 1.29 (0.76,1.81) |
| Algeria | 27.69 (19.46-38.45) | 110.98 (73.69-155.51) | 0.22 (0.15-0.30) | 0.50 (0.33-0.70) | 2.68 (2.55,2.82) |  | 29.76 (20.72-40.94) | 119.05 (79.43-166.31) | 0.24 (0.16-0.32) | 0.54 (0.36-0.75) | 2.64 (2.51,2.76) |  | 733.37 (515.46-1018.75) | 2768.89 (1809.60-3914.81) | 5.80 (4.08-8.06) | 12.53 (8.19-17.71) | 2.52 (2.39,2.65) |
| American Samoa | 0.09 (0.05-0.13) | 0.30 (0.20-0.44) | 0.36 (0.23-0.56) | 1.20 (0.78-1.78) | 4.04 (3.85,4.23) |  | 0.09 (0.06-0.14) | 0.32 (0.21-0.46) | 0.38 (0.24-0.58) | 1.27 (0.83-1.85) | 4.16 (3.93,4.39) |  | 2.40 (1.48-3.77) | 7.69 (5.01-11.49) | 9.90 (6.12-15.55) | 30.89 (20.12-46.17) | 3.93 (3.71,4.15) |
| Andorra | 1.10 (0.67-1.68) | 3.44 (2.20-4.86) | 4.04 (2.48-6.20) | 8.03 (5.14-11.36) | 2.37 (2.22,2.52) |  | 1.11 (0.69-1.68) | 3.43 (2.23-4.79) | 4.08 (2.54-6.17) | 8.03 (5.21-11.18) | 2.40 (2.24,2.56) |  | 25.28 (15.72-38.50) | 67.24 (42.17-96.43) | 93.00 (57.85-141.66) | 157.11 (98.53-225.33) | 1.80 (1.63,1.96) |
| Angola | 131.59 (20.61-344.76) | 229.63 (57.75-568.87) | 2.56 (0.40-6.71) | 1.40 (0.35-3.48) | -2.44 (-2.74,-2.14) |  | 137.05 (21.50-367.31) | 240.86 (59.97-604.17) | 2.67 (0.42-7.15) | 1.47 (0.37-3.69) | -2.39 (-2.68,-2.10) |  | 3972.64 (622.02-10496.02) | 6828.76 (1730.26-16768.04) | 77.32 (12.11-204.28) | 41.76 (10.58-102.54) | -2.43 (-2.70,-2.15) |
| Antigua and Barbuda | 0.22 (0.16-0.29) | 0.33 (0.24-0.44) | 0.72 (0.53-0.96) | 0.75 (0.54-0.99) | -0.21 (-0.85,0.44) |  | 0.25 (0.18-0.32) | 0.37 (0.27-0.48) | 0.82 (0.61-1.08) | 0.82 (0.60-1.08) | -0.35 (-0.96,0.26) |  | 4.91 (3.65-6.63) | 7.91 (5.78-10.57) | 16.30 (12.10-22.01) | 17.70 (12.92-23.65) | -0.10 (-0.72,0.53) |
| Argentina | 28.58 (21.45-37.64) | 110.03 (83.57-139.86) | 0.17 (0.13-0.23) | 0.48 (0.37-0.61) | 4.05 (3.76,4.34) |  | 31.16 (23.60-40.68) | 119.96 (91.73-150.82) | 0.19 (0.14-0.25) | 0.53 (0.40-0.66) | 4.08 (3.71,4.45) |  | 712.20 (530.18-944.43) | 2595.82 (1951.54-3334.12) | 4.30 (3.20-5.70) | 11.41 (8.58-14.66) | 3.88 (3.52,4.25) |
| Armenia | 42.13 (30.56-57.05) | 55.95 (41.76-72.57) | 2.46 (1.79-3.34) | 3.74 (2.79-4.85) | 1.58 (0.97,2.20) |  | 46.26 (33.63-62.73) | 62.10 (46.23-79.68) | 2.70 (1.97-3.67) | 4.15 (3.09-5.32) | 1.35 (0.65,2.06) |  | 1062.07 (765.85-1478.87) | 1314.94 (959.85-1736.47) | 62.10 (44.78-86.47) | 87.80 (64.09-115.95) | 1.02 (0.38,1.66) |
| Australia | 49.80 (36.60-66.52) | 396.81 (299.76-526.02) | 0.59 (0.43-0.79) | 3.08 (2.32-4.08) | 5.42 (5.21,5.62) |  | 51.90 (38.00-68.99) | 383.15 (288.67-507.90) | 0.62 (0.45-0.82) | 2.97 (2.24-3.94) | 5.02 (4.85,5.19) |  | 1181.11 (863.58-1554.24) | 7667.27 (5725.05-10019.91) | 14.01 (10.24-18.44) | 59.45 (44.39-77.70) | 4.70 (4.57,4.82) |
| Austria | 46.84 (35.55-60.20) | 149.35 (110.33-194.02) | 1.21 (0.92-1.55) | 3.33 (2.46-4.32) | 3.61 (3.25,3.97) |  | 48.12 (36.11-61.44) | 136.88 (100.59-178.14) | 1.24 (0.93-1.58) | 3.05 (2.24-3.97) | 3.15 (2.85,3.45) |  | 980.76 (748.30-1283.40) | 2550.76 (1867.18-3385.00) | 25.25 (19.27-33.04) | 56.80 (41.57-75.37) | 2.82 (2.53,3.12) |
| Azerbaijan | 59.82 (33.29-97.87) | 147.22 (78.88-256.42) | 1.63 (0.91-2.67) | 2.80 (1.50-4.88) | 1.36 (1.26,1.45) |  | 64.56 (36.02-104.87) | 157.02 (84.60-273.31) | 1.76 (0.98-2.86) | 2.99 (1.61-5.21) | 1.40 (1.31,1.49) |  | 1612.66 (894.63-2665.15) | 3941.37 (2101.51-6782.81) | 44.02 (24.42-72.75) | 75.07 (40.03-129.19) | 1.28 (1.14,1.42) |
| Bahamas | 0.60 (0.44-0.81) | 1.35 (0.93-1.89) | 0.47 (0.35-0.63) | 0.69 (0.48-0.98) | 1.01 (0.69,1.32) |  | 0.65 (0.48-0.87) | 1.46 (1.01-2.04) | 0.51 (0.37-0.68) | 0.75 (0.52-1.05) | 1.02 (0.68,1.37) |  | 15.66 (11.41-21.27) | 34.38 (23.15-49.70) | 12.20 (8.89-16.57) | 17.72 (11.93-25.62) | 0.98 (0.64,1.32) |
| Bahrain | 2.05 (1.40-2.84) | 6.15 (4.15-8.91) | 0.81 (0.55-1.12) | 0.80 (0.54-1.16) | -1.41 (-2.05,-0.77) |  | 2.18 (1.49-2.99) | 6.27 (4.26-9.01) | 0.86 (0.59-1.18) | 0.82 (0.56-1.18) | -1.63 (-2.31,-0.94) |  | 54.89 (37.15-75.92) | 159.05 (105.67-232.13) | 21.68 (14.67-29.98) | 20.80 (13.82-30.35) | -1.52 (-2.19,-0.85) |
| Bangladesh | 173.34 (118.98-234.32) | 555.90 (370.43-770.50) | 0.32 (0.22-0.43) | 0.68 (0.45-0.94) | 2.79 (2.56,3.02) |  | 184.33 (126.85-248.48) | 599.04 (396.22-824.14) | 0.34 (0.23-0.46) | 0.73 (0.48-1.00) | 2.88 (2.64,3.11) |  | 4921.32 (3364.30-6733.81) | 14583.03 (9854.53-20761.92) | 9.02 (6.17-12.34) | 17.72 (11.97-25.22) | 2.62 (2.41,2.83) |
| Barbados | 0.89 (0.65-1.20) | 1.64 (1.13-2.27) | 0.70 (0.51-0.95) | 1.10 (0.75-1.52) | 1.24 (1.05,1.43) |  | 1.01 (0.74-1.37) | 1.84 (1.28-2.52) | 0.80 (0.58-1.08) | 1.23 (0.86-1.69) | 1.14 (0.91,1.37) |  | 19.23 (14.18-26.22) | 35.89 (24.41-49.58) | 15.18 (11.19-20.70) | 24.00 (16.33-33.16) | 1.31 (1.09,1.53) |
| Belarus | 44.41 (31.73-60.18) | 56.93 (39.11-79.95) | 0.85 (0.61-1.15) | 1.22 (0.84-1.71) | 0.91 (0.73,1.08) |  | 48.34 (34.78-66.13) | 61.39 (42.53-85.59) | 0.93 (0.67-1.27) | 1.32 (0.91-1.84) | 0.88 (0.68,1.08) |  | 1110.20 (784.46-1524.12) | 1353.61 (909.49-1929.55) | 21.26 (15.02-29.19) | 29.03 (19.51-41.39) | 0.68 (0.47,0.88) |
| Belgium | 90.84 (70.01-113.07) | 203.29 (156.18-252.46) | 1.82 (1.40-2.27) | 3.54 (2.72-4.40) | 2.01 (1.59,2.43) |  | 98.97 (76.40-123.22) | 210.49 (161.59-259.54) | 1.98 (1.53-2.47) | 3.67 (2.82-4.53) | 1.93 (1.39,2.48) |  | 1884.10 (1460.96-2370.68) | 3857.34 (2929.79-4841.09) | 37.76 (29.28-47.51) | 67.26 (51.09-84.42) | 1.83 (1.39,2.27) |
| Belize | 0.25 (0.18-0.33) | 0.85 (0.63-1.12) | 0.27 (0.20-0.36) | 0.40 (0.29-0.52) | 0.57 (0.23,0.91) |  | 0.28 (0.21-0.37) | 0.93 (0.70-1.21) | 0.30 (0.22-0.40) | 0.43 (0.33-0.56) | 0.51 (0.07,0.96) |  | 5.97 (4.33-8.26) | 22.16 (16.29-29.74) | 6.38 (4.64-8.84) | 10.33 (7.59-13.86) | 0.91 (0.47,1.35) |
| Benin | 34.95 (17.33-64.68) | 56.74 (35.49-84.22) | 1.44 (0.71-2.67) | 0.84 (0.53-1.25) | -1.98 (-2.12,-1.85) |  | 38.23 (18.95-70.53) | 61.95 (38.32-91.22) | 1.58 (0.78-2.91) | 0.92 (0.57-1.35) | -1.93 (-2.07,-1.79) |  | 919.53 (471.05-1686.16) | 1502.26 (940.83-2257.63) | 37.91 (19.42-69.52) | 22.26 (13.94-33.45) | -1.94 (-2.10,-1.79) |
| Bermuda | 0.22 (0.16-0.30) | 0.25 (0.17-0.34) | 0.74 (0.54-0.99) | 0.78 (0.53-1.07) | 0.15 (-0.34,0.63) |  | 0.24 (0.18-0.33) | 0.27 (0.19-0.36) | 0.82 (0.60-1.10) | 0.84 (0.58-1.15) | -0.02 (-0.54,0.51) |  | 5.04 (3.64-6.85) | 4.89 (3.40-6.74) | 16.97 (12.26-23.07) | 15.40 (10.71-21.22) | -0.44 (-0.97,0.09) |
| Bhutan | 1.17 (0.67-1.86) | 3.84 (2.21-5.66) | 0.37 (0.21-0.59) | 1.01 (0.58-1.50) | 3.27 (3.14,3.39) |  | 1.23 (0.70-1.94) | 4.15 (2.39-6.06) | 0.39 (0.22-0.62) | 1.10 (0.63-1.60) | 3.41 (3.27,3.54) |  | 34.39 (19.03-55.51) | 99.68 (58.21-148.15) | 10.92 (6.04-17.62) | 26.34 (15.38-39.15) | 2.86 (2.75,2.97) |
| Bolivia (Plurinational State of) | 6.34 (3.57-10.11) | 19.77 (10.69-32.23) | 0.20 (0.11-0.32) | 0.34 (0.18-0.55) | 1.96 (1.84,2.07) |  | 7.00 (3.93-11.21) | 22.09 (11.76-35.71) | 0.22 (0.12-0.35) | 0.37 (0.20-0.61) | 2.03 (1.90,2.16) |  | 159.85 (89.19-262.70) | 470.69 (252.87-755.13) | 5.01 (2.80-8.23) | 7.98 (4.29-12.80) | 1.72 (1.59,1.86) |
| Bosnia and Herzegovina | 29.82 (20.09-40.62) | 45.31 (29.48-66.23) | 1.33 (0.89-1.81) | 2.74 (1.79-4.01) | 2.06 (1.89,2.22) |  | 32.23 (21.93-44.04) | 50.35 (33.19-73.92) | 1.43 (0.98-1.96) | 3.05 (2.01-4.48) | 2.12 (1.99,2.26) |  | 776.47 (521.16-1062.71) | 1006.26 (652.53-1483.07) | 34.52 (23.17-47.25) | 60.93 (39.51-89.81) | 1.36 (1.19,1.53) |
| Botswana | 3.89 (1.86-7.72) | 10.30 (5.75-18.71) | 0.59 (0.28-1.17) | 0.86 (0.48-1.56) | 0.81 (0.60,1.03) |  | 4.19 (2.03-8.20) | 11.10 (6.20-19.89) | 0.64 (0.31-1.24) | 0.93 (0.52-1.66) | 0.81 (0.58,1.05) |  | 106.01 (50.04-219.70) | 281.88 (147.22-545.13) | 16.07 (7.59-33.31) | 23.56 (12.30-45.56) | 0.82 (0.56,1.07) |
| Brazil | 241.58 (212.95-268.74) | 782.17 (673.59-877.52) | 0.33 (0.29-0.36) | 0.71 (0.61-0.80) | 3.22 (2.93,3.51) |  | 262.44 (231.42-290.65) | 859.10 (738.82-962.23) | 0.35 (0.31-0.39) | 0.78 (0.67-0.87) | 3.32 (3.03,3.62) |  | 6340.33 (5573.63-7128.79) | 19005.06 (16526.05-21606.11) | 8.54 (7.51-9.60) | 17.25 (15.00-19.61) | 3.08 (2.78,3.38) |
| Brunei Darussalam | 2.13 (1.26-3.21) | 5.55 (3.64-7.87) | 1.64 (0.97-2.47) | 2.46 (1.61-3.49) | 1.09 (0.88,1.31) |  | 2.23 (1.31-3.34) | 5.53 (3.61-7.84) | 1.72 (1.01-2.58) | 2.45 (1.60-3.47) | 0.91 (0.66,1.17) |  | 54.81 (32.68-83.34) | 137.16 (88.94-196.00) | 42.28 (25.21-64.29) | 60.81 (39.43-86.89) | 0.96 (0.69,1.22) |
| Bulgaria | 94.31 (63.39-134.50) | 47.21 (30.83-71.33) | 2.17 (1.46-3.10) | 1.39 (0.91-2.10) | -1.23 (-1.69,-0.76) |  | 102.95 (68.79-146.35) | 52.87 (34.97-78.87) | 2.37 (1.58-3.37) | 1.56 (1.03-2.32) | -1.10 (-1.66,-0.54) |  | 2337.34 (1566.68-3331.75) | 1071.12 (693.05-1637.04) | 53.86 (36.10-76.77) | 31.56 (20.42-48.24) | -1.46 (-2.01,-0.91) |
| Burkina Faso | 84.37 (36.64-195.80) | 128.03 (61.04-246.18) | 1.77 (0.77-4.11) | 1.12 (0.54-2.16) | -1.75 (-1.95,-1.55) |  | 90.95 (40.33-212.41) | 139.32 (65.89-268.58) | 1.91 (0.85-4.46) | 1.22 (0.58-2.36) | -1.74 (-1.94,-1.54) |  | 2282.62 (1007.84-5176.51) | 3440.40 (1599.51-6809.75) | 47.91 (21.16-108.66) | 30.23 (14.05-59.84) | -1.75 (-1.98,-1.52) |
| Burundi | 20.10 (11.81-35.10) | 22.61 (13.95-37.53) | 0.72 (0.43-1.26) | 0.34 (0.21-0.57) | -2.79 (-3.07,-2.52) |  | 21.56 (12.97-36.96) | 24.12 (14.86-39.77) | 0.78 (0.47-1.33) | 0.36 (0.22-0.60) | -2.80 (-3.08,-2.52) |  | 554.96 (316.30-986.70) | 638.83 (389.39-1086.22) | 19.99 (11.39-35.54) | 9.66 (5.89-16.43) | -2.69 (-2.98,-2.41) |
| Cabo Verde | 2.46 (1.44-4.31) | 5.13 (2.91-7.55) | 1.39 (0.82-2.43) | 1.83 (1.04-2.70) | 0.77 (0.53,1.02) |  | 2.82 (1.67-5.01) | 5.86 (3.31-8.60) | 1.60 (0.94-2.83) | 2.09 (1.18-3.08) | 0.76 (0.50,1.02) |  | 55.21 (33.44-93.50) | 115.94 (65.97-165.75) | 31.21 (18.90-52.86) | 41.46 (23.59-59.27) | 0.76 (0.51,1.01) |
| Cambodia | 50.57 (24.20-98.11) | 104.87 (55.14-182.93) | 0.98 (0.47-1.91) | 1.23 (0.65-2.15) | 0.64 (0.48,0.79) |  | 54.01 (25.91-105.27) | 112.69 (59.26-196.41) | 1.05 (0.50-2.05) | 1.32 (0.70-2.30) | 0.66 (0.52,0.80) |  | 1399.94 (671.28-2666.03) | 2715.94 (1398.58-4994.11) | 27.26 (13.07-51.90) | 31.87 (16.41-58.60) | 0.39 (0.25,0.54) |
| Cameroon | 73.86 (44.42-109.24) | 117.10 (53.46-195.54) | 1.42 (0.85-2.09) | 0.74 (0.34-1.23) | -2.40 (-2.62,-2.17) |  | 79.37 (48.06-117.35) | 126.32 (58.71-214.99) | 1.52 (0.92-2.25) | 0.80 (0.37-1.35) | -2.36 (-2.59,-2.13) |  | 2028.90 (1225.06-3012.25) | 3189.63 (1461.34-5370.58) | 38.88 (23.48-57.72) | 20.07 (9.20-33.80) | -2.43 (-2.67,-2.20) |
| Canada | 72.39 (53.67-97.81) | 390.24 (278.43-530.14) | 0.53 (0.39-0.72) | 2.08 (1.49-2.83) | 4.78 (4.67,4.90) |  | 71.85 (53.09-96.62) | 373.98 (267.22-503.32) | 0.53 (0.39-0.71) | 2.00 (1.43-2.69) | 4.83 (4.68,4.99) |  | 1593.18 (1180.24-2122.34) | 7348.07 (5266.95-9941.98) | 11.69 (8.66-15.58) | 39.22 (28.11-53.07) | 4.43 (4.27,4.59) |
| Central African Republic | 33.89 (13.69-64.63) | 39.36 (17.35-78.62) | 2.48 (1.00-4.73) | 1.44 (0.63-2.87) | -2.33 (-2.56,-2.10) |  | 35.16 (14.22-67.70) | 40.72 (17.78-82.61) | 2.57 (1.04-4.96) | 1.48 (0.65-3.01) | -2.36 (-2.60,-2.11) |  | 1019.48 (414.37-1947.50) | 1216.41 (537.65-2459.79) | 74.67 (30.35-142.64) | 44.36 (19.61-89.71) | -2.25 (-2.48,-2.01) |
| Chad | 35.55 (15.12-77.20) | 64.01 (33.09-123.16) | 1.18 (0.50-2.56) | 0.72 (0.37-1.39) | -1.95 (-2.07,-1.83) |  | 38.85 (16.24-84.77) | 69.25 (36.07-133.00) | 1.29 (0.54-2.81) | 0.78 (0.41-1.50) | -1.96 (-2.09,-1.83) |  | 932.08 (397.55-1988.91) | 1741.08 (907.52-3467.09) | 30.93 (13.19-66.01) | 19.62 (10.23-39.07) | -1.80 (-1.94,-1.67) |
| Chile | 27.39 (20.33-36.66) | 150.03 (111.54-193.65) | 0.41 (0.31-0.55) | 1.60 (1.19-2.06) | 4.93 (4.57,5.30) |  | 29.75 (22.23-39.87) | 159.35 (118.72-204.06) | 0.45 (0.33-0.60) | 1.70 (1.26-2.17) | 4.91 (4.60,5.23) |  | 675.19 (495.15-912.80) | 3308.21 (2478.03-4284.35) | 10.16 (7.45-13.74) | 35.20 (26.37-45.58) | 4.62 (4.31,4.92) |
| China | 7211.12 (5844.11-8769.70) | 18213.60 (14201.81-22419.76) | 1.23 (0.99-1.49) | 2.56 (2.00-3.15) | 2.53 (2.36,2.71) |  | 7634.22 (6204.22-9250.21) | 17449.54 (13706.31-21482.37) | 1.30 (1.05-1.57) | 2.45 (1.93-3.02) | 2.18 (2.01,2.36) |  | 193240.31 (154614.55-235926.91) | 375510.14 (292740.95-466847.72) | 32.85 (26.28-40.11) | 52.79 (41.15-65.63) | 1.60 (1.44,1.76) |
| Colombia | 65.58 (49.52-84.83) | 175.05 (128.48-227.87) | 0.40 (0.30-0.52) | 0.71 (0.52-0.93) | 2.15 (1.55,2.75) |  | 72.02 (54.83-92.46) | 196.73 (144.29-254.05) | 0.44 (0.34-0.57) | 0.80 (0.59-1.04) | 2.25 (1.67,2.83) |  | 1656.20 (1241.65-2166.09) | 3833.57 (2814.49-5075.00) | 10.19 (7.64-13.33) | 15.63 (11.47-20.69) | 1.64 (1.08,2.22) |
| Comoros | 1.78 (1.04-2.65) | 3.72 (2.40-5.55) | 0.77 (0.45-1.15) | 1.00 (0.65-1.49) | 0.59 (0.45,0.72) |  | 1.92 (1.13-2.84) | 4.07 (2.63-6.06) | 0.83 (0.49-1.23) | 1.09 (0.71-1.63) | 0.64 (0.51,0.77) |  | 47.83 (27.37-71.94) | 97.06 (61.21-149.61) | 20.68 (11.83-31.11) | 26.08 (16.45-40.20) | 0.47 (0.30,0.63) |
| Congo | 35.20 (16.22-69.40) | 47.83 (22.72-94.93) | 2.93 (1.35-5.78) | 1.77 (0.84-3.52) | -2.20 (-2.52,-1.87) |  | 36.95 (17.15-73.66) | 50.01 (23.55-99.71) | 3.08 (1.43-6.13) | 1.86 (0.87-3.70) | -2.18 (-2.50,-1.87) |  | 1022.96 (479.34-2052.90) | 1416.38 (672.05-2888.30) | 85.18 (39.92-170.95) | 52.54 (24.93-107.14) | -2.11 (-2.45,-1.78) |
| Cook Islands | 0.13 (0.08-0.19) | 0.26 (0.17-0.37) | 1.34 (0.86-1.96) | 2.88 (1.89-4.15) | 2.59 (2.44,2.74) |  | 0.14 (0.09-0.20) | 0.27 (0.18-0.38) | 1.44 (0.93-2.08) | 2.99 (2.00-4.33) | 2.50 (2.35,2.65) |  | 3.25 (2.06-4.86) | 5.75 (3.72-8.43) | 34.33 (21.80-51.35) | 64.74 (41.92-94.96) | 2.19 (2.04,2.34) |
| Costa Rica | 15.46 (12.09-19.52) | 61.27 (47.08-79.01) | 1.02 (0.80-1.28) | 2.58 (1.98-3.33) | 2.90 (2.38,3.41) |  | 17.03 (13.21-21.48) | 67.26 (51.81-85.87) | 1.12 (0.87-1.41) | 2.83 (2.18-3.62) | 3.05 (2.53,3.57) |  | 370.97 (292.63-463.99) | 1381.20 (1037.24-1793.33) | 24.39 (19.24-30.51) | 58.18 (43.69-75.54) | 2.81 (2.28,3.33) |
| Côte d'Ivoire | 17.92 (11.10-28.24) | 32.23 (15.58-53.44) | 0.29 (0.18-0.46) | 0.23 (0.11-0.38) | -1.02 (-1.14,-0.91) |  | 18.94 (11.87-29.61) | 34.50 (16.73-57.84) | 0.31 (0.19-0.49) | 0.25 (0.12-0.42) | -0.92 (-1.04,-0.81) |  | 513.26 (313.67-830.39) | 888.26 (425.13-1485.39) | 8.42 (5.14-13.61) | 6.38 (3.05-10.66) | -1.12 (-1.24,-1.00) |
| Croatia | 9.51 (6.26-13.80) | 16.66 (10.66-24.44) | 0.39 (0.26-0.57) | 0.79 (0.51-1.16) | 2.81 (2.27,3.35) |  | 10.29 (6.78-15.02) | 16.91 (10.81-24.88) | 0.42 (0.28-0.62) | 0.80 (0.51-1.18) | 2.68 (2.15,3.21) |  | 213.59 (139.89-320.74) | 307.21 (196.04-448.93) | 8.79 (5.76-13.20) | 14.60 (9.32-21.33) | 2.19 (1.68,2.71) |
| Cuba | 29.97 (22.39-38.72) | 39.08 (28.22-51.14) | 0.55 (0.41-0.71) | 0.69 (0.50-0.91) | 0.10 (-0.30,0.50) |  | 33.42 (24.84-43.12) | 42.89 (30.93-55.74) | 0.62 (0.46-0.80) | 0.76 (0.55-0.99) | 0.02 (-0.50,0.55) |  | 688.60 (514.96-891.35) | 878.93 (630.41-1160.10) | 12.70 (9.49-16.43) | 15.60 (11.19-20.59) | 0.04 (-0.46,0.54) |
| Cyprus | 4.50 (3.11-6.29) | 13.07 (8.87-18.70) | 1.16 (0.80-1.62) | 1.93 (1.31-2.75) | 1.81 (1.70,1.92) |  | 4.87 (3.42-6.79) | 13.28 (9.05-19.12) | 1.25 (0.88-1.75) | 1.96 (1.33-2.82) | 1.54 (1.41,1.68) |  | 102.93 (71.12-141.76) | 250.84 (170.95-359.05) | 26.46 (18.28-36.44) | 36.95 (25.18-52.89) | 1.16 (1.07,1.26) |
| Czechia | 61.79 (42.77-84.69) | 53.41 (35.20-76.20) | 1.20 (0.83-1.65) | 1.00 (0.66-1.43) | -0.68 (-0.88,-0.48) |  | 68.63 (47.10-93.71) | 57.96 (38.29-83.03) | 1.33 (0.92-1.82) | 1.09 (0.72-1.56) | -0.81 (-1.07,-0.56) |  | 1448.83 (1010.75-1993.10) | 1100.45 (727.89-1578.15) | 28.15 (19.64-38.72) | 20.70 (13.69-29.69) | -1.10 (-1.35,-0.85) |
| Democratic People's Republic of Korea | 147.70 (79.37-259.78) | 246.75 (155.51-383.67) | 1.43 (0.77-2.52) | 1.87 (1.18-2.91) | 0.78 (0.72,0.84) |  | 156.39 (84.70-277.26) | 260.03 (166.25-404.65) | 1.52 (0.82-2.69) | 1.97 (1.26-3.07) | 0.79 (0.74,0.84) |  | 4098.28 (2141.19-7264.44) | 6097.02 (3736.47-9465.27) | 39.81 (20.80-70.56) | 46.20 (28.31-71.73) | 0.36 (0.30,0.43) |
| Democratic Republic of the Congo | 196.04 (105.36-410.23) | 344.76 (167.51-738.14) | 1.03 (0.55-2.15) | 0.77 (0.37-1.64) | -1.35 (-1.52,-1.18) |  | 205.86 (108.91-433.03) | 364.77 (175.21-793.69) | 1.08 (0.57-2.27) | 0.81 (0.39-1.76) | -1.34 (-1.50,-1.18) |  | 5727.75 (3075.91-12211.47) | 10019.59 (4716.94-21431.68) | 30.03 (16.13-64.02) | 22.26 (10.48-47.62) | -1.34 (-1.51,-1.16) |
| Denmark | 24.94 (18.79-31.68) | 69.99 (52.00-90.62) | 0.97 (0.73-1.23) | 2.39 (1.78-3.10) | 2.57 (2.31,2.83) |  | 21.68 (16.41-27.38) | 65.48 (48.60-85.10) | 0.84 (0.64-1.06) | 2.24 (1.66-2.91) | 2.96 (2.66,3.27) |  | 445.99 (341.70-575.19) | 1232.65 (906.22-1630.17) | 17.34 (13.29-22.36) | 42.13 (30.97-55.72) | 2.76 (2.44,3.08) |
| Djibouti | 0.76 (0.47-1.22) | 4.00 (2.47-6.47) | 0.37 (0.23-0.59) | 0.64 (0.39-1.03) | 1.65 (1.51,1.80) |  | 0.80 (0.49-1.28) | 4.24 (2.62-6.86) | 0.38 (0.24-0.62) | 0.67 (0.42-1.09) | 1.72 (1.56,1.88) |  | 22.25 (13.28-36.20) | 113.37 (68.29-186.28) | 10.74 (6.41-17.48) | 18.01 (10.85-29.60) | 1.57 (1.39,1.74) |
| Dominica | 0.21 (0.13-0.34) | 0.36 (0.23-0.52) | 0.58 (0.36-0.95) | 1.07 (0.70-1.54) | 1.48 (1.33,1.63) |  | 0.24 (0.15-0.39) | 0.41 (0.27-0.58) | 0.66 (0.41-1.08) | 1.21 (0.79-1.73) | 1.54 (1.32,1.75) |  | 4.80 (3.00-7.67) | 8.15 (5.30-11.86) | 13.24 (8.28-21.19) | 24.29 (15.80-35.35) | 1.66 (1.45,1.87) |
| Dominican Republic | 6.21 (4.29-8.79) | 23.13 (14.84-33.06) | 0.17 (0.12-0.25) | 0.42 (0.27-0.60) | 3.20 (3.08,3.32) |  | 6.84 (4.71-9.58) | 25.62 (16.48-36.84) | 0.19 (0.13-0.27) | 0.47 (0.30-0.67) | 3.19 (3.07,3.31) |  | 159.73 (111.67-231.81) | 571.92 (363.51-843.62) | 4.47 (3.12-6.48) | 10.39 (6.60-15.32) | 3.02 (2.89,3.16) |
| Ecuador | 11.95 (8.17-17.25) | 30.19 (19.38-45.30) | 0.24 (0.16-0.35) | 0.33 (0.21-0.50) | 1.42 (0.72,2.13) |  | 13.41 (9.21-19.22) | 34.43 (22.25-51.41) | 0.27 (0.18-0.39) | 0.38 (0.25-0.57) | 1.28 (0.54,2.03) |  | 290.00 (202.45-418.22) | 665.82 (421.86-1003.56) | 5.81 (4.06-8.38) | 7.37 (4.67-11.11) | 0.89 (0.17,1.62) |
| Egypt | 1274.67 (827.26-2087.29) | 3360.30 (2457.84-4356.46) | 4.61 (2.99-7.54) | 6.36 (4.65-8.25) | 1.48 (1.20,1.76) |  | 1344.21 (872.89-2183.90) | 3489.54 (2550.93-4477.65) | 4.86 (3.16-7.89) | 6.61 (4.83-8.48) | 1.37 (1.11,1.63) |  | 36087.93 (23765.12-58317.98) | 92649.63 (67080.22-121804.80) | 130.44 (85.90-210.79) | 175.43 (127.01-230.63) | 1.39 (1.08,1.71) |
| El Salvador | 11.73 (9.05-15.21) | 21.03 (15.50-27.78) | 0.44 (0.34-0.57) | 0.65 (0.48-0.86) | 1.62 (1.13,2.11) |  | 12.88 (9.96-16.63) | 23.27 (17.21-30.71) | 0.49 (0.38-0.63) | 0.72 (0.53-0.95) | 1.50 (1.00,2.01) |  | 299.33 (231.40-391.40) | 501.42 (361.61-683.57) | 11.28 (8.72-14.75) | 15.55 (11.21-21.20) | 1.24 (0.70,1.78) |
| Equatorial Guinea | 2.12 (1.15-4.66) | 6.36 (3.70-10.40) | 1.00 (0.54-2.20) | 0.84 (0.49-1.38) | -0.47 (-0.99,0.04) |  | 2.23 (1.22-4.98) | 6.72 (3.94-10.99) | 1.06 (0.58-2.35) | 0.89 (0.52-1.45) | -0.41 (-0.92,0.10) |  | 61.85 (32.93-136.36) | 181.16 (101.13-301.42) | 29.25 (15.58-64.50) | 23.96 (13.37-39.86) | -0.57 (-1.10,-0.03) |
| Eritrea | 6.96 (4.25-11.96) | 16.76 (10.39-28.77) | 0.41 (0.25-0.70) | 0.51 (0.31-0.87) | 0.34 (0.24,0.44) |  | 7.23 (4.44-12.35) | 17.75 (10.95-30.28) | 0.42 (0.26-0.73) | 0.54 (0.33-0.92) | 0.43 (0.32,0.55) |  | 209.24 (132.11-364.79) | 481.44 (291.42-821.55) | 12.29 (7.76-21.42) | 14.59 (8.83-24.90) | 0.20 (0.08,0.32) |
| Estonia | 8.83 (6.53-11.78) | 13.95 (9.88-18.86) | 1.13 (0.83-1.50) | 2.13 (1.51-2.88) | 1.56 (1.28,1.84) |  | 9.66 (7.17-12.81) | 15.50 (11.00-20.50) | 1.23 (0.91-1.63) | 2.36 (1.68-3.13) | 1.71 (1.41,2.01) |  | 216.92 (157.58-291.64) | 288.63 (204.48-392.43) | 27.66 (20.09-37.19) | 44.04 (31.20-59.88) | 1.01 (0.69,1.32) |
| Eswatini | 3.26 (1.66-5.68) | 10.95 (4.55-22.69) | 0.81 (0.41-1.41) | 1.90 (0.79-3.93) | 2.98 (2.05,3.91) |  | 3.51 (1.79-6.09) | 11.64 (4.88-24.49) | 0.87 (0.44-1.51) | 2.01 (0.84-4.24) | 2.96 (2.03,3.91) |  | 89.74 (44.72-162.34) | 314.23 (129.17-673.13) | 22.26 (11.09-40.26) | 54.40 (22.36-116.53) | 3.19 (2.14,4.24) |
| Ethiopia | 135.62 (101.22-193.17) | 199.65 (131.67-320.33) | 0.54 (0.40-0.76) | 0.37 (0.24-0.59) | -1.66 (-1.93,-1.39) |  | 143.60 (107.50-204.56) | 217.66 (144.47-345.99) | 0.57 (0.43-0.81) | 0.40 (0.27-0.64) | -1.54 (-1.81,-1.26) |  | 3857.77 (2864.72-5406.58) | 5246.87 (3408.34-8460.92) | 15.26 (11.33-21.38) | 9.63 (6.26-15.53) | -1.94 (-2.22,-1.66) |
| Fiji | 1.44 (0.84-2.49) | 3.76 (2.38-5.66) | 0.38 (0.22-0.66) | 0.81 (0.52-1.22) | 2.83 (2.61,3.04) |  | 1.52 (0.89-2.61) | 3.96 (2.54-5.93) | 0.40 (0.23-0.69) | 0.86 (0.55-1.28) | 2.89 (2.63,3.15) |  | 41.27 (23.74-71.29) | 101.08 (62.37-154.38) | 10.88 (6.26-18.80) | 21.87 (13.49-33.40) | 2.72 (2.47,2.98) |
| Finland | 46.69 (35.59-59.52) | 139.78 (103.53-178.20) | 1.86 (1.42-2.38) | 5.05 (3.74-6.44) | 3.44 (3.34,3.54) |  | 43.19 (32.77-55.45) | 114.64 (85.09-144.76) | 1.72 (1.31-2.21) | 4.14 (3.07-5.23) | 3.00 (2.78,3.23) |  | 880.55 (673.66-1135.06) | 1977.41 (1467.30-2528.79) | 35.15 (26.89-45.31) | 71.44 (53.01-91.36) | 2.51 (2.33,2.69) |
| France | 738.44 (556.27-960.26) | 2064.44 (1591.98-2594.75) | 2.56 (1.93-3.32) | 6.22 (4.80-7.82) | 2.61 (2.42,2.81) |  | 784.19 (591.50-1005.16) | 1962.86 (1526.27-2444.10) | 2.71 (2.05-3.48) | 5.91 (4.60-7.36) | 2.18 (1.95,2.41) |  | 16700.56 (12364.54-22012.21) | 36234.14 (27922.92-45736.81) | 57.82 (42.81-76.20) | 109.16 (84.12-137.78) | 1.72 (1.47,1.97) |
| Gabon | 15.76 (7.00-32.28) | 24.55 (14.75-38.67) | 3.20 (1.42-6.56) | 2.70 (1.62-4.26) | -0.91 (-1.08,-0.74) |  | 16.78 (7.43-34.04) | 25.92 (15.59-40.34) | 3.41 (1.51-6.92) | 2.85 (1.72-4.44) | -0.89 (-1.07,-0.72) |  | 436.80 (192.72-891.55) | 692.84 (408.16-1104.37) | 88.82 (39.19-181.29) | 76.30 (44.95-121.62) | -0.79 (-0.96,-0.62) |
| Gambia | 6.27 (3.95-9.43) | 22.16 (11.72-34.73) | 1.28 (0.81-1.92) | 1.85 (0.98-2.90) | 0.93 (0.77,1.09) |  | 6.70 (4.25-10.01) | 23.91 (12.69-37.70) | 1.37 (0.87-2.04) | 2.00 (1.06-3.15) | 1.04 (0.87,1.21) |  | 175.30 (109.20-275.28) | 600.40 (320.24-925.13) | 35.72 (22.25-56.09) | 50.16 (26.75-77.28) | 0.81 (0.61,1.01) |
| Georgia | 53.62 (39.36-69.75) | 41.58 (30.75-53.57) | 1.94 (1.43-2.53) | 2.31 (1.70-2.97) | 0.00 (-1.02,1.04) |  | 57.55 (42.16-74.68) | 45.74 (34.33-58.57) | 2.08 (1.53-2.70) | 2.54 (1.90-3.25) | 0.06 (-1.01,1.14) |  | 1410.20 (1028.22-1862.09) | 1021.73 (747.86-1336.16) | 51.07 (37.23-67.43) | 56.65 (41.46-74.08) | -0.32 (-1.46,0.83) |
| Germany | 560.52 (436.59-695.58) | 1519.54 (1138.24-1975.63) | 1.40 (1.09-1.74) | 3.56 (2.67-4.63) | 3.44 (3.23,3.64) |  | 600.34 (469.91-743.93) | 1429.10 (1064.98-1846.10) | 1.50 (1.18-1.86) | 3.35 (2.49-4.32) | 3.08 (2.89,3.27) |  | 11584.36 (9005.78-14483.20) | 25791.53 (19567.27-32928.45) | 28.98 (22.53-36.23) | 60.42 (45.84-77.14) | 2.86 (2.66,3.07) |
| Ghana | 61.99 (35.20-109.96) | 103.33 (54.37-165.38) | 0.83 (0.47-1.47) | 0.60 (0.32-0.97) | -1.82 (-2.22,-1.41) |  | 66.96 (38.08-118.13) | 112.68 (59.43-180.09) | 0.89 (0.51-1.58) | 0.66 (0.35-1.05) | -1.79 (-2.18,-1.40) |  | 1701.29 (970.42-2994.80) | 2704.20 (1458.78-4345.60) | 22.73 (12.96-40.01) | 15.79 (8.52-25.38) | -2.00 (-2.40,-1.59) |
| Greece | 31.29 (22.90-41.47) | 109.52 (78.67-144.20) | 0.60 (0.44-0.80) | 2.15 (1.55-2.83) | 4.28 (4.03,4.52) |  | 33.81 (24.54-44.53) | 120.86 (87.03-159.45) | 0.65 (0.47-0.86) | 2.38 (1.71-3.13) | 4.43 (4.14,4.73) |  | 627.90 (459.23-833.53) | 1965.46 (1453.79-2603.18) | 12.09 (8.84-16.05) | 38.63 (28.58-51.17) | 4.04 (3.73,4.36) |
| Greenland | 0.26 (0.18-0.35) | 0.57 (0.38-0.85) | 0.93 (0.65-1.28) | 2.04 (1.37-3.05) | 2.50 (2.27,2.73) |  | 0.27 (0.19-0.37) | 0.60 (0.40-0.86) | 0.96 (0.68-1.32) | 2.12 (1.43-3.07) | 2.56 (2.29,2.83) |  | 7.49 (5.17-10.61) | 14.66 (9.73-21.79) | 26.96 (18.61-38.20) | 52.27 (34.68-77.68) | 2.15 (1.96,2.33) |
| Grenada | 0.23 (0.16-0.33) | 0.50 (0.36-0.67) | 0.53 (0.37-0.75) | 0.98 (0.71-1.30) | 1.87 (1.17,2.57) |  | 0.27 (0.18-0.37) | 0.56 (0.40-0.74) | 0.61 (0.42-0.86) | 1.09 (0.78-1.44) | 1.75 (1.05,2.47) |  | 5.21 (3.61-7.47) | 11.99 (8.65-16.22) | 11.97 (8.31-17.17) | 23.37 (16.86-31.60) | 2.13 (1.50,2.75) |
| Guam | 0.22 (0.14-0.30) | 0.97 (0.64-1.37) | 0.32 (0.21-0.45) | 1.22 (0.80-1.72) | 4.77 (4.60,4.95) |  | 0.23 (0.15-0.32) | 1.01 (0.67-1.40) | 0.33 (0.22-0.46) | 1.27 (0.84-1.76) | 4.77 (4.55,4.98) |  | 5.86 (3.81-8.43) | 24.06 (15.61-34.56) | 8.57 (5.57-12.33) | 30.22 (19.61-43.42) | 4.66 (4.44,4.88) |
| Guatemala | 52.89 (42.90-63.34) | 120.16 (91.78-150.37) | 1.26 (1.02-1.51) | 1.52 (1.16-1.91) | 0.77 (-0.91,2.48) |  | 57.30 (46.45-68.26) | 132.78 (101.96-165.10) | 1.37 (1.11-1.63) | 1.68 (1.29-2.09) | 0.36 (-1.58,2.34) |  | 1411.44 (1131.66-1704.89) | 2977.39 (2292.59-3813.81) | 33.67 (26.99-40.67) | 37.77 (29.08-48.38) | 0.02 (-1.89,1.97) |
| Guinea | 70.85 (47.95-98.00) | 96.60 (52.52-150.29) | 2.36 (1.60-3.27) | 1.44 (0.78-2.24) | -1.63 (-1.88,-1.37) |  | 76.95 (52.31-106.70) | 104.98 (57.74-165.35) | 2.57 (1.75-3.56) | 1.56 (0.86-2.46) | -1.59 (-1.86,-1.32) |  | 1888.97 (1299.46-2639.65) | 2613.56 (1399.34-4040.41) | 63.03 (43.36-88.08) | 38.92 (20.84-60.17) | -1.58 (-1.78,-1.37) |
| Guinea-Bissau | 8.44 (3.80-15.10) | 9.70 (6.28-14.09) | 1.68 (0.75-3.00) | 0.94 (0.61-1.37) | -2.19 (-2.34,-2.05) |  | 8.96 (4.06-16.00) | 10.33 (6.65-15.01) | 1.78 (0.81-3.18) | 1.00 (0.64-1.45) | -2.14 (-2.29,-1.98) |  | 240.99 (109.96-413.82) | 276.32 (175.83-415.45) | 47.86 (21.84-82.18) | 26.78 (17.04-40.26) | -2.17 (-2.32,-2.01) |
| Guyana | 1.27 (0.91-1.72) | 1.80 (1.21-2.57) | 0.33 (0.23-0.44) | 0.47 (0.32-0.67) | 1.18 (0.89,1.48) |  | 1.38 (1.00-1.87) | 1.94 (1.30-2.74) | 0.35 (0.26-0.48) | 0.51 (0.34-0.72) | 1.29 (0.95,1.64) |  | 33.43 (24.34-45.62) | 48.49 (31.90-69.69) | 8.57 (6.24-11.70) | 12.68 (8.34-18.23) | 1.40 (1.07,1.72) |
| Haiti | 8.11 (4.35-14.81) | 14.63 (7.23-28.99) | 0.25 (0.14-0.46) | 0.23 (0.11-0.45) | -0.43 (-0.51,-0.35) |  | 8.74 (4.72-15.87) | 15.85 (7.79-31.35) | 0.27 (0.15-0.50) | 0.25 (0.12-0.49) | -0.40 (-0.47,-0.33) |  | 219.99 (118.33-403.42) | 393.50 (197.64-789.32) | 6.90 (3.71-12.64) | 6.12 (3.07-12.27) | -0.44 (-0.52,-0.35) |
| Honduras | 9.44 (5.53-17.00) | 52.61 (36.84-75.56) | 0.40 (0.23-0.72) | 1.04 (0.73-1.49) | 3.24 (3.10,3.38) |  | 10.29 (5.98-18.81) | 57.66 (40.39-84.10) | 0.44 (0.25-0.80) | 1.14 (0.80-1.66) | 3.34 (3.14,3.54) |  | 249.00 (151.09-442.93) | 1322.78 (924.36-1936.76) | 10.57 (6.42-18.81) | 26.17 (18.29-38.31) | 3.19 (3.01,3.37) |
| Hungary | 44.15 (30.48-61.25) | 34.16 (22.96-48.29) | 0.85 (0.59-1.18) | 0.71 (0.48-1.01) | -1.05 (-1.78,-0.32) |  | 49.24 (34.16-67.73) | 37.69 (25.53-53.23) | 0.95 (0.66-1.30) | 0.79 (0.53-1.11) | -1.03 (-1.73,-0.32) |  | 1045.06 (734.62-1469.77) | 751.09 (496.58-1070.71) | 20.11 (14.13-28.28) | 15.65 (10.35-22.31) | -1.20 (-1.86,-0.55) |
| Iceland | 1.28 (0.98-1.61) | 4.64 (3.48-6.00) | 1.00 (0.77-1.26) | 2.65 (1.99-3.43) | 2.87 (2.54,3.20) |  | 1.32 (1.01-1.65) | 4.54 (3.42-5.87) | 1.04 (0.80-1.30) | 2.59 (1.95-3.35) | 2.63 (2.27,3.00) |  | 27.45 (21.15-34.49) | 84.18 (62.15-110.56) | 21.62 (16.66-27.17) | 48.05 (35.47-63.11) | 2.27 (1.92,2.63) |
| India | 793.38 (642.12-959.43) | 3214.20 (2665.06-3815.27) | 0.19 (0.15-0.22) | 0.45 (0.38-0.54) | 3.04 (2.90,3.18) |  | 835.92 (678.38-1008.56) | 3455.77 (2866.22-4079.90) | 0.20 (0.16-0.24) | 0.49 (0.41-0.58) | 3.05 (2.90,3.21) |  | 22656.75 (18492.34-27501.89) | 84335.34 (69193.59-100751.60) | 5.31 (4.34-6.45) | 11.92 (9.78-14.25) | 2.66 (2.52,2.80) |
| Indonesia | 640.20 (398.16-941.72) | 1793.70 (974.08-2866.95) | 0.69 (0.43-1.02) | 1.29 (0.70-2.06) | 2.00 (1.92,2.09) |  | 673.50 (419.71-990.26) | 1885.84 (1010.57-3028.30) | 0.73 (0.45-1.07) | 1.35 (0.72-2.17) | 2.00 (1.92,2.09) |  | 18510.84 (11623.72-27356.86) | 48798.40 (27014.61-78558.88) | 20.01 (12.57-29.58) | 34.99 (19.37-56.33) | 1.82 (1.72,1.92) |
| Iran (Islamic Republic of) | 46.70 (35.62-60.91) | 204.69 (164.99-246.15) | 0.16 (0.12-0.21) | 0.48 (0.39-0.58) | 3.30 (2.56,4.03) |  | 50.81 (39.00-66.25) | 226.84 (183.00-272.15) | 0.18 (0.14-0.23) | 0.53 (0.43-0.64) | 3.35 (2.59,4.11) |  | 1207.75 (926.08-1590.84) | 4578.38 (3716.64-5592.73) | 4.23 (3.24-5.57) | 10.73 (8.71-13.10) | 2.72 (1.92,3.52) |
| Iraq | 53.11 (36.08-74.53) | 184.18 (120.81-259.05) | 0.58 (0.39-0.81) | 0.89 (0.59-1.26) | 1.73 (1.22,2.23) |  | 57.34 (38.76-79.91) | 195.26 (129.39-274.15) | 0.62 (0.42-0.87) | 0.95 (0.63-1.33) | 1.66 (1.15,2.18) |  | 1409.96 (949.17-2008.33) | 4736.66 (2985.46-6776.97) | 15.31 (10.31-21.81) | 22.98 (14.48-32.88) | 1.62 (1.15,2.10) |
| Ireland | 14.24 (10.81-18.42) | 52.46 (39.46-66.99) | 0.79 (0.60-1.02) | 2.12 (1.60-2.71) | 3.60 (3.44,3.77) |  | 15.33 (11.71-19.80) | 52.00 (39.57-65.84) | 0.85 (0.65-1.10) | 2.10 (1.60-2.66) | 3.19 (2.95,3.43) |  | 310.29 (238.84-393.62) | 987.11 (741.75-1265.92) | 17.23 (13.26-21.85) | 39.95 (30.02-51.24) | 2.96 (2.76,3.17) |
| Israel | 23.25 (18.50-29.26) | 64.96 (49.52-80.88) | 0.94 (0.75-1.18) | 1.35 (1.03-1.69) | 1.26 (1.13,1.38) |  | 25.36 (20.25-31.97) | 68.09 (52.38-84.54) | 1.02 (0.82-1.29) | 1.42 (1.09-1.76) | 1.06 (0.91,1.20) |  | 513.28 (406.82-643.61) | 1295.23 (1002.73-1622.33) | 20.69 (16.40-25.95) | 27.00 (20.90-33.82) | 0.89 (0.73,1.04) |
| Italy | 1519.48 (1377.64-1664.88) | 2049.33 (1773.13-2284.31) | 5.35 (4.85-5.86) | 6.85 (5.93-7.64) | 0.50 (0.27,0.73) |  | 1538.87 (1390.89-1677.94) | 1924.90 (1643.18-2133.51) | 5.42 (4.90-5.91) | 6.44 (5.49-7.13) | 0.12 (-0.22,0.46) |  | 33179.65 (30023.03-36276.66) | 35088.52 (30700.96-38924.80) | 116.83 (105.72-127.74) | 117.33 (102.66-130.16) | -0.47 (-0.81,-0.12) |
| Jamaica | 2.87 (2.04-3.95) | 6.62 (4.47-9.34) | 0.24 (0.17-0.33) | 0.47 (0.32-0.67) | 2.15 (2.05,2.26) |  | 3.26 (2.30-4.45) | 7.45 (5.04-10.36) | 0.28 (0.19-0.38) | 0.53 (0.36-0.74) | 2.16 (1.83,2.49) |  | 64.83 (46.58-89.35) | 153.54 (100.75-221.80) | 5.48 (3.94-7.55) | 10.97 (7.20-15.84) | 2.29 (1.94,2.64) |
| Japan | 7995.14 (7476.90-8485.49) | 14067.98 (11995.25-15497.70) | 12.71 (11.88-13.49) | 22.03 (18.79-24.27) | 0.87 (0.31,1.42) |  | 6645.85 (6232.89-7015.91) | 11355.73 (9514.83-12457.10) | 10.56 (9.91-11.15) | 17.79 (14.90-19.51) | 0.96 (0.36,1.57) |  | 165194.39 (155273.05-175032.68) | 174238.88 (151010.09-189655.88) | 262.57 (246.80-278.21) | 272.89 (236.51-297.04) | -0.68 (-1.30,-0.06) |
| Jordan | 4.34 (2.60-7.17) | 17.61 (11.37-25.25) | 0.23 (0.14-0.38) | 0.29 (0.18-0.41) | 0.09 (-0.16,0.33) |  | 4.62 (2.75-7.57) | 18.41 (11.98-26.78) | 0.25 (0.15-0.41) | 0.30 (0.19-0.43) | 0.06 (-0.19,0.31) |  | 117.20 (69.15-194.45) | 444.50 (279.38-658.19) | 6.27 (3.70-10.41) | 7.21 (4.53-10.68) | -0.17 (-0.44,0.09) |
| Kazakhstan | 203.46 (145.01-274.11) | 150.66 (110.73-201.12) | 2.48 (1.77-3.34) | 1.59 (1.17-2.12) | -2.26 (-2.60,-1.92) |  | 215.40 (156.69-289.37) | 161.54 (120.12-214.55) | 2.63 (1.91-3.53) | 1.70 (1.27-2.26) | -2.26 (-2.60,-1.93) |  | 5676.33 (3936.76-7733.68) | 3930.33 (2872.66-5226.05) | 69.25 (48.03-94.35) | 41.47 (30.31-55.14) | -2.60 (-2.95,-2.26) |
| Kenya | 36.53 (25.18-54.16) | 154.03 (116.50-210.19) | 0.32 (0.22-0.47) | 0.62 (0.47-0.84) | 2.31 (2.25,2.37) |  | 39.29 (27.16-58.11) | 164.44 (124.60-225.01) | 0.34 (0.23-0.50) | 0.66 (0.50-0.90) | 2.27 (2.19,2.35) |  | 992.57 (688.22-1468.95) | 4268.68 (3177.83-5863.87) | 8.58 (5.95-12.69) | 17.05 (12.70-23.43) | 2.36 (2.27,2.45) |
| Kiribati | 0.27 (0.18-0.39) | 0.53 (0.31-0.80) | 0.72 (0.49-1.04) | 0.87 (0.52-1.32) | 0.43 (0.31,0.56) |  | 0.28 (0.19-0.41) | 0.55 (0.33-0.83) | 0.76 (0.52-1.09) | 0.91 (0.55-1.37) | 0.42 (0.30,0.55) |  | 7.71 (5.12-11.05) | 15.04 (8.82-23.12) | 20.72 (13.76-29.71) | 24.82 (14.56-38.16) | 0.49 (0.37,0.60) |
| Kuwait | 3.48 (2.51-4.64) | 5.13 (3.74-6.67) | 0.40 (0.29-0.54) | 0.22 (0.16-0.29) | -1.80 (-2.19,-1.40) |  | 3.56 (2.61-4.74) | 5.30 (3.93-6.91) | 0.41 (0.30-0.55) | 0.23 (0.17-0.30) | -1.94 (-2.88,-0.98) |  | 95.91 (68.23-129.36) | 118.18 (84.02-155.92) | 11.16 (7.94-15.05) | 5.08 (3.61-6.71) | -2.53 (-3.48,-1.57) |
| Kyrgyzstan | 34.62 (25.25-45.70) | 31.43 (22.62-42.75) | 1.55 (1.13-2.05) | 0.92 (0.66-1.25) | -1.21 (-2.15,-0.25) |  | 36.99 (27.16-48.50) | 33.36 (24.22-45.09) | 1.66 (1.22-2.17) | 0.97 (0.71-1.31) | -1.60 (-2.64,-0.56) |  | 951.47 (686.53-1283.12) | 848.45 (608.96-1172.48) | 42.63 (30.76-57.49) | 24.73 (17.75-34.17) | -1.66 (-2.72,-0.60) |
| Lao People's Democratic Republic | 22.12 (13.24-34.01) | 32.07 (20.13-49.41) | 1.06 (0.63-1.63) | 0.87 (0.55-1.34) | -0.90 (-1.08,-0.71) |  | 23.52 (14.13-35.95) | 34.31 (21.82-51.78) | 1.13 (0.68-1.72) | 0.93 (0.59-1.40) | -0.89 (-1.06,-0.71) |  | 620.81 (372.45-959.06) | 861.61 (523.67-1367.89) | 29.78 (17.87-46.00) | 23.36 (14.20-37.08) | -1.03 (-1.22,-0.85) |
| Latvia | 12.39 (9.01-16.37) | 15.83 (11.44-21.13) | 0.93 (0.68-1.23) | 1.69 (1.22-2.26) | 1.24 (1.01,1.47) |  | 13.52 (9.90-17.89) | 17.81 (12.99-23.77) | 1.02 (0.75-1.35) | 1.90 (1.39-2.54) | 1.63 (1.33,1.93) |  | 310.10 (224.41-416.19) | 343.10 (248.23-463.45) | 23.33 (16.88-31.32) | 36.69 (26.54-49.56) | 1.02 (0.71,1.32) |
| Lebanon | 8.82 (5.66-13.45) | 18.92 (12.11-27.70) | 0.59 (0.38-0.90) | 0.68 (0.44-1.00) | 0.50 (0.31,0.70) |  | 9.49 (6.20-14.29) | 20.28 (13.09-29.78) | 0.63 (0.41-0.96) | 0.73 (0.47-1.07) | 0.46 (0.26,0.66) |  | 227.72 (142.99-350.80) | 428.90 (269.18-630.01) | 15.22 (9.56-23.45) | 15.48 (9.72-22.74) | 0.00 (-0.18,0.18) |
| Lesotho | 6.94 (2.96-14.55) | 19.97 (9.52-40.61) | 0.91 (0.39-1.90) | 2.13 (1.02-4.33) | 2.80 (2.15,3.45) |  | 7.61 (3.19-16.23) | 21.31 (10.22-42.41) | 0.99 (0.42-2.12) | 2.27 (1.09-4.53) | 2.76 (2.12,3.39) |  | 180.17 (74.81-370.93) | 559.76 (257.20-1134.16) | 23.51 (9.76-48.41) | 59.73 (27.44-121.02) | 3.19 (2.54,3.84) |
| Liberia | 19.14 (10.23-32.81) | 26.28 (16.50-40.49) | 1.56 (0.83-2.67) | 0.96 (0.60-1.48) | -2.56 (-2.88,-2.24) |  | 20.88 (11.01-36.13) | 28.34 (18.13-43.69) | 1.70 (0.89-2.94) | 1.04 (0.66-1.60) | -2.62 (-2.95,-2.29) |  | 500.60 (267.81-861.40) | 722.77 (438.25-1122.26) | 40.69 (21.77-70.02) | 26.48 (16.05-41.11) | -2.39 (-2.76,-2.02) |
| Libya | 19.57 (12.87-29.16) | 73.68 (47.57-103.63) | 0.93 (0.61-1.38) | 2.14 (1.38-3.02) | 2.84 (2.55,3.12) |  | 21.07 (13.94-31.34) | 77.61 (50.62-108.94) | 1.00 (0.66-1.49) | 2.26 (1.47-3.17) | 2.74 (2.44,3.04) |  | 516.27 (337.73-789.47) | 1959.70 (1248.27-2836.57) | 24.50 (16.03-37.46) | 57.05 (36.34-82.57) | 2.82 (2.49,3.15) |
| Lithuania | 14.48 (10.92-18.76) | 26.56 (19.43-34.92) | 0.79 (0.59-1.02) | 1.95 (1.42-2.56) | 2.72 (2.37,3.06) |  | 15.68 (11.78-20.29) | 28.29 (20.97-37.47) | 0.85 (0.64-1.10) | 2.07 (1.54-2.75) | 2.94 (2.61,3.27) |  | 341.74 (254.32-445.79) | 560.85 (405.82-753.05) | 18.60 (13.84-24.26) | 41.11 (29.75-55.20) | 2.48 (2.15,2.80) |
| Luxembourg | 3.07 (2.32-3.96) | 8.48 (6.42-10.82) | 1.61 (1.22-2.08) | 2.63 (1.99-3.36) | 1.83 (1.61,2.05) |  | 3.33 (2.51-4.28) | 8.71 (6.68-10.90) | 1.74 (1.32-2.25) | 2.70 (2.07-3.38) | 1.59 (1.34,1.84) |  | 67.85 (51.92-88.11) | 160.15 (119.95-203.78) | 35.60 (27.24-46.23) | 49.71 (37.24-63.26) | 1.22 (0.98,1.46) |
| Madagascar | 23.18 (14.79-35.68) | 45.48 (28.62-71.68) | 0.39 (0.25-0.60) | 0.32 (0.20-0.50) | -0.93 (-1.18,-0.67) |  | 24.74 (15.70-38.10) | 47.97 (29.95-75.40) | 0.42 (0.26-0.64) | 0.34 (0.21-0.53) | -0.95 (-1.20,-0.69) |  | 652.61 (413.78-1013.81) | 1320.96 (810.63-2079.80) | 10.97 (6.95-17.04) | 9.25 (5.68-14.57) | -0.77 (-1.02,-0.51) |
| Malawi | 20.88 (13.78-29.99) | 48.99 (32.02-72.26) | 0.43 (0.28-0.61) | 0.50 (0.33-0.74) | -0.28 (-0.75,0.18) |  | 22.21 (14.85-31.77) | 52.27 (35.16-76.89) | 0.45 (0.30-0.65) | 0.54 (0.36-0.79) | -0.26 (-0.74,0.23) |  | 583.37 (381.38-841.95) | 1364.91 (872.55-2029.02) | 11.90 (7.78-17.17) | 14.04 (8.97-20.87) | -0.27 (-0.75,0.21) |
| Malaysia | 33.24 (22.09-48.02) | 139.01 (91.29-196.80) | 0.38 (0.25-0.54) | 0.87 (0.57-1.24) | 2.85 (2.55,3.15) |  | 36.14 (24.04-52.00) | 146.60 (96.41-205.99) | 0.41 (0.27-0.59) | 0.92 (0.61-1.30) | 2.78 (2.46,3.11) |  | 855.00 (568.72-1241.73) | 3362.65 (2177.96-4865.27) | 9.68 (6.44-14.06) | 21.14 (13.69-30.59) | 2.72 (2.40,3.03) |
| Maldives | 0.55 (0.34-0.83) | 1.53 (0.97-2.22) | 0.49 (0.31-0.75) | 0.59 (0.37-0.86) | 0.62 (0.42,0.81) |  | 0.59 (0.37-0.90) | 1.66 (1.05-2.41) | 0.53 (0.33-0.81) | 0.64 (0.41-0.93) | 0.68 (0.47,0.89) |  | 14.97 (9.29-23.07) | 34.25 (21.31-51.44) | 13.47 (8.36-20.76) | 13.24 (8.24-19.89) | -0.18 (-0.29,-0.06) |
| Mali | 126.19 (80.44-186.94) | 261.73 (168.75-384.95) | 2.91 (1.86-4.32) | 2.17 (1.40-3.19) | -1.00 (-1.15,-0.86) |  | 134.20 (87.06-198.11) | 280.35 (180.55-406.39) | 3.10 (2.01-4.57) | 2.33 (1.50-3.37) | -0.96 (-1.09,-0.82) |  | 3527.57 (2272.43-5290.09) | 7216.42 (4642.25-10771.22) | 81.44 (52.46-122.13) | 59.88 (38.52-89.37) | -1.03 (-1.19,-0.88) |
| Malta | 1.61 (1.22-2.05) | 5.43 (3.90-7.12) | 0.87 (0.66-1.10) | 2.46 (1.76-3.22) | 3.57 (3.45,3.69) |  | 1.72 (1.31-2.18) | 5.56 (4.01-7.25) | 0.93 (0.71-1.18) | 2.51 (1.81-3.28) | 3.54 (3.38,3.70) |  | 36.32 (27.64-46.34) | 105.27 (75.78-137.48) | 19.60 (14.92-25.01) | 47.61 (34.27-62.18) | 3.21 (3.04,3.37) |
| Marshall Islands | 0.06 (0.03-0.10) | 0.14 (0.08-0.25) | 0.25 (0.13-0.45) | 0.49 (0.27-0.87) | 2.32 (2.21,2.43) |  | 0.06 (0.03-0.11) | 0.14 (0.08-0.25) | 0.26 (0.14-0.48) | 0.50 (0.28-0.90) | 2.15 (2.03,2.27) |  | 1.54 (0.81-2.73) | 4.00 (2.17-7.38) | 6.77 (3.57-12.02) | 14.21 (7.70-26.23) | 2.55 (2.46,2.64) |
| Mauritania | 28.47 (8.90-66.35) | 33.12 (18.08-51.98) | 2.77 (0.87-6.46) | 1.51 (0.82-2.36) | -2.36 (-2.58,-2.14) |  | 30.94 (9.64-72.06) | 36.45 (19.60-57.19) | 3.01 (0.94-7.01) | 1.66 (0.89-2.60) | -2.33 (-2.54,-2.11) |  | 748.00 (231.88-1768.26) | 830.66 (453.38-1327.21) | 72.81 (22.57-172.13) | 37.79 (20.63-60.38) | -2.52 (-2.74,-2.30) |
| Mauritius | 3.59 (2.60-4.78) | 1.59 (1.14-2.16) | 0.65 (0.48-0.87) | 0.25 (0.18-0.34) | 1.23 (-0.54,3.03) |  | 3.89 (2.80-5.15) | 1.68 (1.22-2.26) | 0.71 (0.51-0.94) | 0.26 (0.19-0.35) | 2.78 (0.83,4.77) |  | 90.27 (65.26-121.39) | 37.81 (26.80-52.33) | 16.47 (11.91-22.15) | 5.95 (4.21-8.23) | 2.70 (0.77,4.68) |
| Mexico | 226.38 (209.26-242.79) | 967.07 (834.18-1102.82) | 0.53 (0.49-0.57) | 1.50 (1.29-1.71) | 3.64 (3.41,3.87) |  | 248.29 (229.62-265.63) | 1061.08 (913.78-1204.57) | 0.58 (0.54-0.62) | 1.64 (1.41-1.86) | 3.67 (3.43,3.92) |  | 5798.37 (5339.34-6244.34) | 23473.53 (20277.45-26940.99) | 13.58 (12.51-14.63) | 36.32 (31.37-41.68) | 3.52 (3.27,3.77) |
| Micronesia (Federated States of) | 0.24 (0.15-0.38) | 0.38 (0.22-0.65) | 0.46 (0.29-0.73) | 0.74 (0.42-1.26) | 1.50 (1.43,1.57) |  | 0.25 (0.16-0.40) | 0.39 (0.23-0.67) | 0.48 (0.31-0.78) | 0.77 (0.44-1.30) | 1.42 (1.34,1.50) |  | 6.53 (4.07-10.50) | 10.73 (5.88-18.81) | 12.62 (7.87-20.29) | 20.93 (11.46-36.68) | 1.66 (1.58,1.73) |
| Monaco | 0.50 (0.32-0.74) | 1.53 (0.99-2.18) | 3.29 (2.12-4.84) | 8.07 (5.23-11.51) | 3.14 (2.56,3.72) |  | 0.53 (0.34-0.79) | 1.56 (1.03-2.25) | 3.51 (2.26-5.20) | 8.26 (5.45-11.89) | 3.01 (2.42,3.61) |  | 10.01 (6.49-14.46) | 28.65 (18.61-40.79) | 65.84 (42.69-95.09) | 151.33 (98.29-215.47) | 2.95 (2.38,3.53) |
| Mongolia | 122.14 (78.24-185.55) | 347.94 (244.51-480.05) | 11.32 (7.25-17.20) | 20.86 (14.66-28.78) | 2.63 (2.20,3.06) |  | 130.10 (82.87-196.77) | 372.63 (263.26-514.82) | 12.06 (7.68-18.24) | 22.34 (15.78-30.86) | 2.61 (2.20,3.02) |  | 3408.52 (2122.64-5246.63) | 9468.53 (6546.40-13258.16) | 315.92 (196.74-486.29) | 567.61 (392.44-794.78) | 2.42 (2.03,2.80) |
| Montenegro | 3.70 (2.43-5.56) | 7.27 (4.82-10.84) | 1.18 (0.78-1.78) | 2.35 (1.56-3.51) | 2.39 (2.28,2.51) |  | 4.05 (2.67-6.01) | 7.97 (5.30-11.90) | 1.29 (0.85-1.92) | 2.58 (1.72-3.85) | 2.45 (2.33,2.58) |  | 89.08 (58.58-137.60) | 161.45 (106.45-243.73) | 28.45 (18.71-43.95) | 52.25 (34.45-78.87) | 2.09 (1.94,2.24) |
| Morocco | 9.92 (6.42-14.59) | 32.25 (20.24-44.67) | 0.08 (0.05-0.12) | 0.17 (0.11-0.24) | 2.61 (2.31,2.92) |  | 10.65 (6.92-15.71) | 34.44 (21.84-47.36) | 0.08 (0.05-0.12) | 0.19 (0.12-0.25) | 2.61 (2.31,2.90) |  | 265.25 (169.90-385.48) | 843.67 (517.50-1203.47) | 2.09 (1.34-3.04) | 4.54 (2.78-6.47) | 2.50 (2.16,2.84) |
| Mozambique | 116.91 (73.65-181.01) | 259.64 (129.92-487.35) | 1.75 (1.10-2.71) | 1.67 (0.84-3.14) | 0.01 (-0.10,0.13) |  | 128.66 (81.55-198.17) | 285.26 (143.39-535.80) | 1.93 (1.22-2.97) | 1.84 (0.92-3.45) | -0.00 (-0.12,0.12) |  | 2961.36 (1862.80-4627.35) | 6622.27 (3291.82-12373.23) | 44.33 (27.89-69.27) | 42.62 (21.19-79.64) | 0.09 (-0.04,0.21) |
| Myanmar | 129.38 (49.59-234.62) | 256.91 (109.44-457.61) | 0.64 (0.25-1.16) | 0.91 (0.39-1.62) | 0.93 (0.83,1.04) |  | 138.03 (53.66-253.76) | 275.24 (119.55-482.05) | 0.68 (0.27-1.26) | 0.98 (0.42-1.71) | 0.95 (0.84,1.05) |  | 3602.21 (1296.83-6798.96) | 6653.55 (2868.62-12076.19) | 17.82 (6.41-33.63) | 23.59 (10.17-42.81) | 0.70 (0.58,0.81) |
| Namibia | 2.04 (1.23-3.24) | 5.34 (3.35-7.99) | 0.29 (0.18-0.46) | 0.44 (0.28-0.66) | 0.99 (0.67,1.31) |  | 2.20 (1.34-3.52) | 5.78 (3.74-8.68) | 0.31 (0.19-0.50) | 0.48 (0.31-0.71) | 1.03 (0.70,1.37) |  | 53.91 (33.42-85.62) | 140.93 (85.26-220.81) | 7.68 (4.76-12.20) | 11.59 (7.01-18.16) | 1.02 (0.67,1.36) |
| Nauru | 0.03 (0.02-0.04) | 0.03 (0.02-0.05) | 0.50 (0.31-0.76) | 0.51 (0.29-0.84) | -0.50 (-0.70,-0.30) |  | 0.03 (0.02-0.04) | 0.03 (0.02-0.05) | 0.51 (0.33-0.77) | 0.52 (0.29-0.86) | -0.61 (-0.84,-0.39) |  | 0.77 (0.47-1.18) | 0.88 (0.48-1.49) | 15.11 (9.31-23.21) | 15.98 (8.80-27.03) | -0.35 (-0.54,-0.17) |
| Nepal | 23.48 (15.31-35.67) | 108.67 (61.79-162.30) | 0.24 (0.16-0.37) | 0.70 (0.40-1.04) | 3.98 (3.56,4.39) |  | 24.79 (16.39-37.68) | 116.51 (66.23-171.14) | 0.25 (0.17-0.39) | 0.75 (0.43-1.10) | 4.05 (3.64,4.46) |  | 676.92 (427.63-1023.70) | 2895.31 (1660.67-4357.91) | 6.95 (4.39-10.52) | 18.60 (10.67-28.00) | 3.73 (3.30,4.16) |
| Netherlands | 33.46 (25.38-43.62) | 124.34 (93.27-159.79) | 0.45 (0.34-0.58) | 1.44 (1.08-1.86) | 3.97 (3.73,4.21) |  | 37.87 (28.87-48.64) | 140.02 (105.44-180.23) | 0.51 (0.39-0.65) | 1.63 (1.23-2.09) | 3.91 (3.68,4.13) |  | 763.40 (568.88-995.13) | 2667.38 (1985.18-3459.24) | 10.23 (7.62-13.34) | 31.00 (23.07-40.20) | 3.75 (3.56,3.95) |
| New Zealand | 12.43 (10.61-14.45) | 66.13 (56.16-76.27) | 0.73 (0.62-0.85) | 2.56 (2.17-2.95) | 3.70 (3.46,3.95) |  | 10.98 (9.41-12.72) | 51.04 (43.18-58.86) | 0.64 (0.55-0.74) | 1.97 (1.67-2.28) | 3.50 (3.20,3.80) |  | 253.23 (215.83-295.24) | 1072.25 (913.83-1229.50) | 14.82 (12.63-17.28) | 41.48 (35.35-47.57) | 3.23 (2.96,3.50) |
| Nicaragua | 10.40 (7.70-14.19) | 29.63 (20.98-40.41) | 0.54 (0.40-0.73) | 0.89 (0.63-1.21) | 2.16 (1.66,2.67) |  | 11.32 (8.40-15.38) | 32.21 (22.78-43.46) | 0.58 (0.43-0.79) | 0.97 (0.68-1.30) | 2.17 (1.64,2.70) |  | 272.85 (201.73-375.00) | 744.87 (516.75-1041.10) | 14.04 (10.38-19.29) | 22.34 (15.50-31.22) | 2.06 (1.50,2.63) |
| Niger | 42.07 (18.36-87.95) | 76.52 (42.55-140.73) | 1.05 (0.46-2.19) | 0.61 (0.34-1.12) | -1.94 (-2.03,-1.84) |  | 44.69 (19.79-92.49) | 82.73 (46.52-151.67) | 1.11 (0.49-2.30) | 0.66 (0.37-1.21) | -1.82 (-1.91,-1.73) |  | 1195.82 (519.91-2416.21) | 2063.15 (1127.87-3788.89) | 29.78 (12.95-60.16) | 16.48 (9.01-30.26) | -2.06 (-2.16,-1.96) |
| Nigeria | 280.40 (132.22-565.87) | 460.48 (297.42-709.98) | 0.62 (0.29-1.26) | 0.40 (0.26-0.61) | -1.82 (-1.99,-1.65) |  | 307.05 (145.75-620.56) | 501.08 (328.88-766.64) | 0.68 (0.32-1.38) | 0.43 (0.28-0.66) | -1.82 (-1.98,-1.65) |  | 7248.49 (3362.51-14743.26) | 12054.46 (7711.14-18974.94) | 16.10 (7.47-32.75) | 10.43 (6.67-16.42) | -1.76 (-1.94,-1.59) |
| Niue | 0.01 (0.01-0.02) | 0.01 (0.01-0.02) | 0.88 (0.53-1.39) | 1.31 (0.78-2.06) | 1.30 (0.94,1.65) |  | 0.01 (0.01-0.02) | 0.01 (0.01-0.02) | 0.99 (0.60-1.54) | 1.42 (0.86-2.21) | 1.20 (0.85,1.55) |  | 0.24 (0.14-0.39) | 0.27 (0.16-0.43) | 20.80 (12.46-33.49) | 31.73 (18.86-51.15) | 1.24 (0.88,1.60) |
| North Macedonia | 19.09 (13.22-27.11) | 29.29 (19.17-42.43) | 1.92 (1.33-2.72) | 2.69 (1.76-3.90) | 0.86 (0.70,1.03) |  | 20.99 (14.62-29.75) | 32.22 (21.31-46.57) | 2.11 (1.47-2.99) | 2.96 (1.96-4.28) | 0.85 (0.68,1.02) |  | 476.51 (328.18-673.30) | 676.37 (449.61-1005.86) | 47.84 (32.94-67.59) | 62.15 (41.32-92.43) | 0.53 (0.35,0.70) |
| Northern Mariana Islands | 0.08 (0.05-0.12) | 0.32 (0.20-0.46) | 0.34 (0.22-0.52) | 1.31 (0.81-1.90) | 4.96 (4.31,5.61) |  | 0.08 (0.05-0.12) | 0.32 (0.20-0.46) | 0.34 (0.22-0.52) | 1.32 (0.82-1.92) | 4.98 (4.31,5.65) |  | 2.25 (1.44-3.53) | 8.34 (5.13-12.27) | 9.99 (6.38-15.64) | 34.38 (21.17-50.63) | 4.68 (4.06,5.31) |
| Norway | 21.88 (19.38-24.31) | 76.70 (66.40-86.09) | 1.03 (0.91-1.15) | 2.83 (2.45-3.18) | 3.10 (2.73,3.48) |  | 23.02 (20.36-25.36) | 74.15 (63.65-83.28) | 1.08 (0.96-1.19) | 2.74 (2.35-3.07) | 2.84 (2.38,3.30) |  | 454.67 (401.41-503.20) | 1406.28 (1228.92-1569.46) | 21.42 (18.91-23.70) | 51.91 (45.36-57.93) | 2.78 (2.33,3.24) |
| Oman | 3.28 (1.90-5.58) | 10.97 (7.05-15.78) | 0.33 (0.19-0.56) | 0.47 (0.30-0.67) | 1.39 (1.20,1.58) |  | 3.45 (2.01-5.93) | 11.07 (7.03-15.88) | 0.35 (0.20-0.60) | 0.47 (0.30-0.68) | 1.33 (1.11,1.56) |  | 90.92 (51.82-157.67) | 294.10 (187.82-429.54) | 9.16 (5.22-15.89) | 12.50 (7.99-18.26) | 1.34 (1.10,1.58) |
| Pakistan | 519.12 (401.33-677.26) | 1322.50 (971.02-1768.96) | 0.93 (0.72-1.22) | 1.12 (0.82-1.50) | 0.55 (0.45,0.65) |  | 552.57 (428.13-717.03) | 1382.41 (1021.47-1844.77) | 0.99 (0.77-1.29) | 1.17 (0.87-1.57) | 0.50 (0.41,0.60) |  | 14753.41 (11216.80-19356.36) | 39275.71 (28313.45-52854.71) | 26.55 (20.19-34.84) | 33.35 (24.04-44.88) | 0.70 (0.57,0.83) |
| Palau | 0.05 (0.03-0.08) | 0.12 (0.07-0.18) | 0.63 (0.34-1.06) | 1.29 (0.73-2.04) | 2.26 (2.16,2.36) |  | 0.05 (0.03-0.09) | 0.12 (0.07-0.19) | 0.67 (0.37-1.12) | 1.32 (0.75-2.05) | 2.15 (2.06,2.24) |  | 1.31 (0.73-2.28) | 3.33 (1.88-5.33) | 17.31 (9.58-30.05) | 36.78 (20.81-58.91) | 2.44 (2.30,2.58) |
| Palestine | 11.66 (7.61-16.78) | 24.99 (17.21-34.39) | 1.14 (0.74-1.64) | 0.97 (0.67-1.34) | -0.64 (-0.78,-0.50) |  | 12.91 (8.49-18.57) | 26.79 (18.71-36.87) | 1.26 (0.83-1.81) | 1.04 (0.73-1.44) | -0.77 (-0.91,-0.63) |  | 285.93 (184.43-418.61) | 626.32 (428.36-873.47) | 27.94 (18.02-40.90) | 24.39 (16.68-34.02) | -0.65 (-0.81,-0.49) |
| Panama | 10.72 (8.28-13.32) | 26.47 (19.18-34.24) | 0.90 (0.69-1.11) | 1.23 (0.89-1.60) | 1.63 (0.96,2.31) |  | 11.92 (9.26-14.79) | 29.52 (21.72-38.02) | 1.00 (0.78-1.24) | 1.38 (1.01-1.77) | 1.55 (0.89,2.22) |  | 256.37 (199.62-323.77) | 599.11 (431.34-788.64) | 21.46 (16.71-27.11) | 27.92 (20.10-36.75) | 1.39 (0.71,2.07) |
| Papua New Guinea | 6.53 (2.71-16.54) | 12.65 (5.34-30.58) | 0.32 (0.13-0.81) | 0.24 (0.10-0.58) | -1.26 (-1.37,-1.15) |  | 6.85 (2.85-17.26) | 13.25 (5.61-32.07) | 0.33 (0.14-0.84) | 0.25 (0.11-0.61) | -1.32 (-1.44,-1.20) |  | 187.56 (75.47-472.87) | 362.58 (153.20-856.93) | 9.14 (3.68-23.05) | 6.93 (2.93-16.38) | -1.23 (-1.33,-1.14) |
| Paraguay | 6.56 (4.67-8.98) | 27.94 (18.02-39.72) | 0.32 (0.23-0.44) | 0.78 (0.50-1.11) | 3.75 (3.38,4.11) |  | 7.28 (5.19-9.91) | 30.75 (20.16-43.87) | 0.36 (0.26-0.49) | 0.86 (0.56-1.22) | 3.78 (3.39,4.17) |  | 163.29 (116.24-225.25) | 687.00 (428.63-987.12) | 8.08 (5.75-11.14) | 19.17 (11.96-27.54) | 3.74 (3.35,4.12) |
| Peru | 12.36 (8.05-18.60) | 47.63 (29.09-73.06) | 0.11 (0.07-0.17) | 0.26 (0.16-0.40) | 2.71 (2.52,2.90) |  | 13.80 (9.10-20.89) | 53.54 (33.08-81.13) | 0.13 (0.08-0.19) | 0.30 (0.18-0.45) | 2.82 (2.60,3.04) |  | 304.16 (198.93-449.09) | 1063.16 (654.26-1659.42) | 2.81 (1.84-4.15) | 5.86 (3.61-9.15) | 2.40 (2.18,2.63) |
| Philippines | 167.57 (114.14-246.22) | 474.33 (366.72-589.82) | 0.53 (0.36-0.78) | 0.84 (0.65-1.04) | 1.43 (1.35,1.52) |  | 178.54 (120.81-264.32) | 506.82 (395.03-626.57) | 0.57 (0.38-0.84) | 0.90 (0.70-1.11) | 1.42 (1.34,1.51) |  | 4720.09 (3208.52-6858.86) | 12518.50 (9578.01-15906.43) | 14.98 (10.18-21.77) | 22.11 (16.91-28.09) | 1.21 (1.14,1.29) |
| Poland | 34.55 (29.81-39.40) | 133.31 (112.76-154.54) | 0.18 (0.16-0.21) | 0.70 (0.59-0.81) | 5.29 (4.61,5.97) |  | 39.56 (34.19-44.96) | 152.11 (129.46-176.20) | 0.21 (0.18-0.24) | 0.80 (0.68-0.92) | 5.35 (4.64,6.06) |  | 782.13 (675.17-901.13) | 2943.73 (2474.08-3456.67) | 4.10 (3.54-4.72) | 15.40 (12.94-18.08) | 5.38 (4.67,6.10) |
| Portugal | 46.16 (35.52-59.15) | 193.91 (144.18-249.32) | 0.91 (0.70-1.17) | 3.66 (2.72-4.70) | 4.26 (3.99,4.53) |  | 51.18 (39.68-65.24) | 210.12 (156.31-266.61) | 1.01 (0.78-1.29) | 3.96 (2.95-5.03) | 4.29 (4.01,4.58) |  | 1080.72 (829.24-1397.05) | 4069.75 (3037.16-5246.55) | 21.32 (16.36-27.56) | 76.73 (57.26-98.92) | 4.04 (3.77,4.32) |
| Puerto Rico | 13.23 (9.77-17.61) | 24.76 (17.25-33.44) | 0.73 (0.54-0.97) | 1.50 (1.05-2.03) | 1.62 (1.13,2.12) |  | 14.54 (10.73-19.29) | 27.30 (18.91-36.55) | 0.81 (0.59-1.07) | 1.66 (1.15-2.22) | 1.88 (1.45,2.30) |  | 312.41 (229.19-414.89) | 514.91 (359.01-708.86) | 17.30 (12.69-22.97) | 31.26 (21.80-43.03) | 1.45 (1.03,1.88) |
| Qatar | 1.67 (1.11-2.48) | 16.09 (10.19-23.70) | 0.75 (0.50-1.12) | 1.08 (0.68-1.59) | -0.03 (-0.75,0.69) |  | 1.73 (1.17-2.56) | 15.64 (9.96-22.85) | 0.78 (0.52-1.15) | 1.05 (0.67-1.53) | -0.50 (-1.21,0.22) |  | 47.46 (30.83-72.24) | 414.03 (260.92-616.03) | 21.34 (13.86-32.48) | 27.81 (17.53-41.39) | -0.52 (-1.21,0.17) |
| Republic of Korea | 772.50 (502.43-1135.94) | 1639.18 (1102.22-2358.28) | 3.49 (2.27-5.13) | 6.36 (4.27-9.15) | 1.98 (1.89,2.07) |  | 792.31 (519.18-1172.53) | 1274.30 (850.21-1814.42) | 3.58 (2.35-5.30) | 4.94 (3.30-7.04) | 0.93 (0.81,1.05) |  | 19493.71 (12560.56-28986.85) | 24229.63 (16102.12-35165.51) | 88.12 (56.78-131.03) | 93.97 (62.45-136.38) | 0.02 (-0.06,0.09) |
| Republic of Moldova | 16.20 (11.92-21.28) | 22.41 (16.55-29.77) | 0.73 (0.54-0.96) | 1.25 (0.92-1.66) | 1.27 (0.51,2.04) |  | 17.40 (12.88-22.81) | 24.59 (18.04-32.55) | 0.78 (0.58-1.03) | 1.37 (1.00-1.81) | 1.27 (0.46,2.09) |  | 429.84 (312.63-575.48) | 541.22 (398.25-727.73) | 19.33 (14.06-25.88) | 30.12 (22.17-40.50) | 0.83 (-0.03,1.70) |
| Romania | 38.14 (26.70-53.92) | 114.30 (77.57-158.82) | 0.33 (0.23-0.46) | 1.21 (0.82-1.68) | 4.72 (4.49,4.96) |  | 41.88 (29.42-59.41) | 126.52 (86.89-176.41) | 0.36 (0.25-0.51) | 1.34 (0.92-1.86) | 4.88 (4.55,5.21) |  | 956.31 (657.50-1361.13) | 2591.06 (1729.08-3654.47) | 8.18 (5.62-11.64) | 27.36 (18.26-38.59) | 4.48 (4.15,4.81) |
| Russian Federation | 383.20 (337.29-434.99) | 862.36 (739.92-986.60) | 0.51 (0.45-0.58) | 1.19 (1.02-1.36) | 3.49 (3.03,3.94) |  | 415.11 (366.53-469.44) | 953.30 (818.87-1085.60) | 0.55 (0.49-0.62) | 1.32 (1.13-1.50) | 3.56 (3.05,4.07) |  | 9878.89 (8654.46-11341.80) | 20088.20 (17120.28-23159.70) | 13.09 (11.46-15.02) | 27.74 (23.64-31.98) | 3.12 (2.62,3.62) |
| Rwanda | 27.05 (16.51-40.36) | 41.07 (26.49-59.28) | 0.75 (0.46-1.12) | 0.62 (0.40-0.89) | -1.37 (-1.85,-0.89) |  | 28.71 (17.65-42.68) | 44.20 (28.41-63.91) | 0.80 (0.49-1.19) | 0.67 (0.43-0.96) | -1.22 (-1.73,-0.70) |  | 768.51 (456.96-1147.79) | 1113.66 (710.06-1627.25) | 21.38 (12.71-31.93) | 16.78 (10.70-24.53) | -1.43 (-1.94,-0.92) |
| Saint Kitts and Nevis | 0.21 (0.16-0.28) | 0.27 (0.19-0.37) | 1.01 (0.76-1.36) | 0.92 (0.64-1.25) | -1.02 (-1.69,-0.35) |  | 0.24 (0.17-0.32) | 0.29 (0.20-0.39) | 1.14 (0.84-1.53) | 0.99 (0.68-1.34) | -0.98 (-1.58,-0.38) |  | 4.83 (3.62-6.58) | 6.82 (4.69-9.46) | 23.31 (17.47-31.74) | 23.26 (16.00-32.25) | -0.50 (-1.19,0.20) |
| Saint Lucia | 0.25 (0.18-0.34) | 0.40 (0.28-0.55) | 0.36 (0.27-0.50) | 0.45 (0.32-0.62) | 0.14 (-0.38,0.65) |  | 0.28 (0.20-0.38) | 0.45 (0.32-0.60) | 0.41 (0.30-0.55) | 0.50 (0.36-0.68) | 0.09 (-0.41,0.59) |  | 5.97 (4.37-8.15) | 9.76 (6.83-13.66) | 8.74 (6.40-11.94) | 10.99 (7.70-15.39) | 0.21 (-0.32,0.75) |
| Saint Vincent and the Grenadines | 0.32 (0.23-0.42) | 0.41 (0.30-0.56) | 0.58 (0.42-0.77) | 0.72 (0.52-0.98) | 0.56 (0.13,0.99) |  | 0.35 (0.26-0.47) | 0.46 (0.34-0.62) | 0.65 (0.47-0.86) | 0.80 (0.59-1.08) | 0.46 (-0.01,0.94) |  | 7.49 (5.56-10.19) | 10.07 (7.08-13.81) | 13.69 (10.15-18.61) | 17.65 (12.41-24.21) | 0.58 (0.08,1.08) |
| Samoa | 0.40 (0.25-0.60) | 0.66 (0.40-0.99) | 0.48 (0.30-0.71) | 0.62 (0.38-0.92) | 0.65 (0.52,0.79) |  | 0.44 (0.28-0.65) | 0.71 (0.43-1.05) | 0.52 (0.33-0.77) | 0.66 (0.40-0.98) | 0.63 (0.48,0.79) |  | 10.36 (6.46-15.82) | 16.75 (10.04-25.96) | 12.26 (7.65-18.73) | 15.68 (9.40-24.30) | 0.59 (0.47,0.71) |
| San Marino | 0.12 (0.08-0.17) | 0.32 (0.19-0.50) | 1.03 (0.69-1.45) | 1.98 (1.19-3.08) | 2.32 (2.23,2.40) |  | 0.13 (0.09-0.18) | 0.33 (0.20-0.52) | 1.07 (0.72-1.51) | 2.02 (1.23-3.16) | 2.28 (2.18,2.37) |  | 2.52 (1.71-3.60) | 5.96 (3.53-9.43) | 21.19 (14.40-30.34) | 36.43 (21.58-57.60) | 2.00 (1.88,2.11) |
| Sao Tome and Principe | 0.14 (0.09-0.20) | 0.19 (0.09-0.33) | 0.23 (0.15-0.33) | 0.18 (0.09-0.30) | -1.09 (-1.23,-0.94) |  | 0.15 (0.10-0.22) | 0.21 (0.11-0.36) | 0.25 (0.16-0.37) | 0.20 (0.10-0.33) | -1.13 (-1.29,-0.97) |  | 3.43 (2.26-4.94) | 5.02 (2.40-8.56) | 5.65 (3.72-8.14) | 4.63 (2.22-7.90) | -0.95 (-1.11,-0.79) |
| Saudi Arabia | 64.60 (38.08-105.59) | 171.52 (108.31-251.30) | 0.81 (0.48-1.33) | 0.91 (0.57-1.33) | -0.23 (-0.45,-0.01) |  | 68.99 (41.36-113.27) | 174.54 (110.92-253.27) | 0.87 (0.52-1.43) | 0.93 (0.59-1.34) | -0.36 (-0.59,-0.13) |  | 1764.76 (1025.55-2957.49) | 4616.83 (2852.41-6976.20) | 22.26 (12.94-37.30) | 24.49 (15.13-37.00) | -0.31 (-0.57,-0.05) |
| Senegal | 21.20 (12.46-32.16) | 42.42 (23.27-69.66) | 0.56 (0.33-0.84) | 0.53 (0.29-0.88) | -0.40 (-0.52,-0.29) |  | 22.97 (13.56-35.13) | 46.61 (25.55-78.09) | 0.60 (0.36-0.92) | 0.59 (0.32-0.98) | -0.39 (-0.53,-0.26) |  | 566.64 (329.19-859.60) | 1090.64 (584.00-1765.06) | 14.85 (8.63-22.53) | 13.75 (7.36-22.26) | -0.58 (-0.73,-0.43) |
| Serbia | 109.01 (74.66-150.12) | 165.02 (113.63-236.07) | 2.26 (1.55-3.12) | 3.70 (2.55-5.29) | 1.18 (0.92,1.44) |  | 117.82 (81.74-162.83) | 180.30 (124.41-257.08) | 2.45 (1.70-3.38) | 4.04 (2.79-5.76) | 1.25 (0.98,1.53) |  | 2808.41 (1906.51-3918.09) | 3694.30 (2533.23-5324.61) | 58.33 (39.60-81.38) | 82.84 (56.80-119.39) | 0.67 (0.40,0.93) |
| Seychelles | 0.52 (0.36-0.71) | 0.49 (0.31-0.71) | 1.43 (0.97-1.95) | 0.94 (0.59-1.34) | -0.94 (-1.60,-0.28) |  | 0.58 (0.40-0.79) | 0.53 (0.34-0.74) | 1.59 (1.09-2.18) | 1.00 (0.64-1.41) | -1.12 (-1.78,-0.45) |  | 12.59 (8.66-17.39) | 12.21 (7.62-17.80) | 34.55 (23.77-47.73) | 23.17 (14.45-33.76) | -0.88 (-1.57,-0.19) |
| Sierra Leone | 27.58 (10.78-58.58) | 30.54 (19.23-46.83) | 1.33 (0.52-2.82) | 0.69 (0.43-1.06) | -2.62 (-2.86,-2.38) |  | 30.29 (12.04-65.06) | 33.32 (20.75-51.20) | 1.46 (0.58-3.13) | 0.75 (0.47-1.15) | -2.67 (-2.92,-2.42) |  | 714.62 (270.20-1555.58) | 807.58 (503.87-1254.47) | 34.43 (13.02-74.94) | 18.21 (11.36-28.29) | -2.53 (-2.76,-2.30) |
| Singapore | 19.17 (13.14-26.32) | 77.24 (52.55-107.23) | 1.26 (0.86-1.73) | 2.70 (1.83-3.74) | 2.40 (2.18,2.62) |  | 19.08 (13.26-26.03) | 61.31 (41.85-84.10) | 1.25 (0.87-1.71) | 2.14 (1.46-2.94) | 1.78 (1.50,2.06) |  | 448.03 (306.71-617.07) | 1216.54 (816.56-1729.59) | 29.41 (20.13-40.50) | 42.48 (28.51-60.40) | 1.20 (0.91,1.49) |
| Slovakia | 32.63 (22.13-45.78) | 37.40 (23.11-57.81) | 1.24 (0.84-1.73) | 1.38 (0.85-2.13) | 0.03 (-0.10,0.17) |  | 35.97 (24.29-50.05) | 40.92 (25.48-63.18) | 1.36 (0.92-1.89) | 1.51 (0.94-2.33) | -0.03 (-0.19,0.12) |  | 786.91 (523.64-1114.56) | 839.23 (513.10-1340.01) | 29.79 (19.82-42.19) | 30.91 (18.90-49.36) | -0.23 (-0.38,-0.07) |
| Slovenia | 11.11 (7.70-15.20) | 24.30 (16.64-33.91) | 1.13 (0.78-1.54) | 2.35 (1.61-3.28) | 2.68 (2.45,2.91) |  | 12.20 (8.45-16.62) | 26.33 (18.26-36.49) | 1.24 (0.86-1.68) | 2.54 (1.76-3.53) | 2.42 (2.15,2.69) |  | 263.53 (184.21-359.18) | 479.05 (327.05-662.11) | 26.71 (18.67-36.40) | 46.29 (31.60-63.98) | 1.78 (1.53,2.03) |
| Solomon Islands | 0.69 (0.25-1.63) | 1.44 (0.81-2.56) | 0.41 (0.15-0.96) | 0.42 (0.24-0.75) | -0.03 (-0.21,0.15) |  | 0.72 (0.25-1.71) | 1.49 (0.83-2.71) | 0.42 (0.15-1.01) | 0.44 (0.24-0.79) | -0.07 (-0.24,0.11) |  | 20.16 (6.76-47.53) | 42.63 (23.37-77.67) | 11.89 (3.99-28.04) | 12.47 (6.84-22.73) | -0.00 (-0.15,0.14) |
| Somalia | 32.39 (16.00-55.54) | 75.92 (38.40-139.23) | 0.82 (0.40-1.40) | 0.70 (0.36-1.29) | -0.80 (-0.95,-0.65) |  | 33.85 (16.88-58.20) | 80.26 (41.22-144.82) | 0.85 (0.43-1.47) | 0.74 (0.38-1.34) | -0.71 (-0.86,-0.56) |  | 964.99 (476.55-1685.27) | 2185.17 (1100.85-4101.64) | 24.31 (12.01-42.46) | 20.23 (10.19-37.97) | -0.92 (-1.11,-0.74) |
| South Africa | 106.70 (65.61-164.79) | 342.38 (282.15-405.72) | 0.58 (0.35-0.89) | 1.20 (0.99-1.43) | 2.06 (1.53,2.60) |  | 116.16 (71.37-179.69) | 373.90 (310.08-440.84) | 0.63 (0.39-0.97) | 1.32 (1.09-1.55) | 1.98 (1.43,2.53) |  | 2953.25 (1843.36-4525.48) | 8948.18 (7273.86-10730.86) | 15.96 (9.96-24.45) | 31.48 (25.59-37.75) | 1.83 (1.25,2.42) |
| South Sudan | 18.77 (11.23-27.81) | 33.96 (19.86-52.97) | 0.64 (0.38-0.95) | 0.70 (0.41-1.10) | -0.05 (-0.53,0.43) |  | 20.22 (12.25-29.74) | 36.10 (21.30-55.25) | 0.69 (0.42-1.01) | 0.75 (0.44-1.14) | -0.04 (-0.51,0.44) |  | 507.22 (288.36-772.59) | 958.20 (553.29-1532.61) | 17.26 (9.81-26.29) | 19.81 (11.44-31.69) | 0.19 (-0.30,0.68) |
| Spain | 507.12 (408.76-616.55) | 1405.73 (1086.12-1742.60) | 2.62 (2.11-3.18) | 6.17 (4.77-7.65) | 2.40 (2.04,2.75) |  | 539.83 (432.68-656.60) | 1375.54 (1062.38-1702.95) | 2.78 (2.23-3.39) | 6.04 (4.66-7.48) | 2.16 (1.86,2.45) |  | 11585.81 (9268.52-14174.04) | 26237.27 (20515.96-32499.51) | 59.75 (47.80-73.10) | 115.20 (90.08-142.70) | 1.80 (1.55,2.04) |
| Sri Lanka | 21.86 (14.47-30.95) | 42.17 (24.18-64.42) | 0.26 (0.17-0.36) | 0.38 (0.22-0.58) | 0.72 (0.36,1.07) |  | 23.92 (15.90-33.17) | 44.08 (25.46-67.66) | 0.28 (0.19-0.39) | 0.40 (0.23-0.61) | 0.63 (0.25,1.01) |  | 552.93 (360.10-791.14) | 961.36 (543.07-1483.88) | 6.46 (4.20-9.24) | 8.63 (4.88-13.33) | 0.43 (0.05,0.81) |
| Sudan | 44.28 (21.25-84.69) | 102.05 (62.18-153.24) | 0.44 (0.21-0.85) | 0.47 (0.29-0.71) | 0.21 (-0.10,0.53) |  | 47.99 (22.85-91.89) | 110.49 (67.11-164.13) | 0.48 (0.23-0.92) | 0.51 (0.31-0.76) | 0.19 (-0.11,0.50) |  | 1187.68 (580.56-2212.63) | 2695.55 (1665.90-4054.52) | 11.86 (5.80-22.10) | 12.42 (7.67-18.68) | 0.18 (-0.17,0.53) |
| Suriname | 0.68 (0.47-0.96) | 1.99 (1.26-2.97) | 0.35 (0.24-0.50) | 0.69 (0.43-1.03) | 2.10 (1.84,2.35) |  | 0.75 (0.53-1.05) | 2.18 (1.39-3.21) | 0.39 (0.27-0.54) | 0.75 (0.48-1.11) | 1.91 (1.59,2.23) |  | 17.54 (12.14-25.21) | 50.54 (30.85-75.23) | 9.07 (6.28-13.04) | 17.45 (10.65-25.98) | 1.83 (1.50,2.16) |
| Sweden | 67.05 (57.42-75.94) | 102.20 (84.08-119.96) | 1.56 (1.34-1.77) | 1.97 (1.62-2.31) | 1.11 (0.29,1.94) |  | 76.25 (65.54-86.75) | 114.29 (93.89-133.76) | 1.78 (1.53-2.02) | 2.20 (1.81-2.58) | 1.32 (0.52,2.12) |  | 1466.57 (1261.18-1668.35) | 2052.19 (1686.22-2423.23) | 34.15 (29.37-38.85) | 39.57 (32.51-46.72) | 1.24 (0.42,2.08) |
| Switzerland | 66.75 (49.17-88.38) | 142.02 (104.52-181.17) | 1.94 (1.43-2.57) | 3.18 (2.34-4.06) | 1.68 (1.51,1.85) |  | 67.53 (49.44-89.08) | 136.75 (102.24-174.41) | 1.97 (1.44-2.59) | 3.07 (2.29-3.91) | 1.61 (1.50,1.72) |  | 1383.52 (1034.23-1829.71) | 2477.54 (1821.83-3204.07) | 40.30 (30.12-53.29) | 55.53 (40.84-71.82) | 1.23 (1.10,1.37) |
| Syrian Arab Republic | 62.24 (39.01-96.51) | 125.55 (79.13-179.98) | 0.98 (0.61-1.52) | 1.79 (1.13-2.57) | 1.81 (0.95,2.69) |  | 67.91 (42.68-106.36) | 132.70 (84.74-190.63) | 1.07 (0.67-1.67) | 1.89 (1.21-2.72) | 1.73 (0.84,2.63) |  | 1642.85 (1032.01-2523.57) | 3160.36 (1941.42-4612.24) | 25.84 (16.23-39.69) | 45.05 (27.67-65.75) | 1.61 (0.72,2.51) |
| Taiwan (Province of China) | 152.42 (115.23-196.40) | 791.47 (597.13-1008.65) | 1.49 (1.13-1.93) | 6.70 (5.05-8.53) | 5.98 (4.93,7.04) |  | 154.71 (117.15-197.15) | 742.21 (566.41-933.41) | 1.52 (1.15-1.93) | 6.28 (4.79-7.90) | 5.78 (4.70,6.87) |  | 4055.68 (3035.77-5307.67) | 15028.28 (11324.91-19227.59) | 39.78 (29.78-52.06) | 127.16 (95.82-162.69) | 4.86 (3.82,5.91) |
| Tajikistan | 32.83 (19.91-53.72) | 57.41 (34.39-89.26) | 1.22 (0.74-2.00) | 1.13 (0.68-1.76) | -0.37 (-0.49,-0.24) |  | 35.69 (21.40-58.69) | 61.03 (36.58-94.38) | 1.33 (0.80-2.19) | 1.20 (0.72-1.86) | -0.41 (-0.56,-0.27) |  | 882.56 (554.96-1390.99) | 1606.83 (960.64-2499.64) | 32.88 (20.67-51.82) | 31.63 (18.91-49.21) | -0.30 (-0.49,-0.12) |
| Thailand | 380.80 (251.83-542.96) | 890.74 (570.08-1315.83) | 1.34 (0.89-1.91) | 2.67 (1.71-3.95) | 1.94 (1.75,2.14) |  | 407.81 (267.38-577.85) | 940.35 (595.85-1377.94) | 1.44 (0.94-2.04) | 2.82 (1.79-4.13) | 1.90 (1.74,2.07) |  | 10123.56 (6733.21-14579.00) | 19837.63 (12314.92-29703.06) | 35.67 (23.72-51.37) | 59.50 (36.94-89.09) | 1.32 (1.11,1.53) |
| Timor-Leste | 1.05 (0.61-1.59) | 3.08 (1.75-4.87) | 0.27 (0.16-0.41) | 0.44 (0.25-0.70) | 1.84 (1.75,1.94) |  | 1.12 (0.66-1.68) | 3.34 (1.87-5.35) | 0.29 (0.17-0.43) | 0.48 (0.27-0.77) | 1.93 (1.82,2.05) |  | 30.04 (17.08-46.13) | 78.10 (44.39-124.03) | 7.69 (4.37-11.81) | 11.17 (6.35-17.75) | 1.37 (1.26,1.49) |
| Togo | 9.96 (6.49-14.19) | 28.45 (15.42-47.35) | 0.55 (0.36-0.78) | 0.68 (0.37-1.13) | 0.40 (0.31,0.50) |  | 10.69 (7.00-15.31) | 30.63 (16.73-51.04) | 0.59 (0.38-0.84) | 0.73 (0.40-1.22) | 0.44 (0.34,0.54) |  | 276.88 (180.15-409.39) | 772.30 (424.04-1296.66) | 15.19 (9.88-22.45) | 18.45 (10.13-30.98) | 0.33 (0.23,0.43) |
| Tokelau | 0.01 (0.00-0.01) | 0.01 (0.00-0.01) | 0.76 (0.41-1.44) | 1.02 (0.57-1.74) | 0.67 (0.45,0.89) |  | 0.01 (0.00-0.01) | 0.01 (0.00-0.01) | 0.84 (0.46-1.60) | 1.09 (0.61-1.88) | 0.55 (0.32,0.78) |  | 0.15 (0.08-0.29) | 0.17 (0.10-0.30) | 18.95 (10.27-36.51) | 25.31 (14.31-43.07) | 0.76 (0.56,0.97) |
| Tonga | 1.07 (0.61-1.75) | 1.59 (0.99-2.41) | 2.16 (1.24-3.54) | 3.00 (1.86-4.54) | 0.71 (0.35,1.06) |  | 1.14 (0.66-1.88) | 1.72 (1.07-2.58) | 2.31 (1.33-3.80) | 3.23 (2.01-4.85) | 0.77 (0.40,1.14) |  | 28.81 (16.38-48.85) | 40.04 (24.25-62.42) | 58.29 (33.14-98.83) | 75.33 (45.63-117.45) | 0.51 (0.17,0.85) |
| Trinidad and Tobago | 2.56 (1.92-3.33) | 5.08 (3.36-7.15) | 0.42 (0.32-0.55) | 0.73 (0.48-1.03) | 1.47 (1.12,1.82) |  | 2.83 (2.12-3.69) | 5.54 (3.72-7.69) | 0.47 (0.35-0.61) | 0.80 (0.53-1.10) | 1.32 (0.93,1.72) |  | 62.82 (46.14-82.04) | 125.09 (82.40-178.02) | 10.43 (7.66-13.62) | 17.96 (11.83-25.56) | 1.43 (1.05,1.81) |
| Tunisia | 17.58 (12.20-25.00) | 56.39 (35.29-81.07) | 0.42 (0.29-0.60) | 0.95 (0.60-1.37) | 2.75 (2.64,2.86) |  | 18.91 (13.13-26.81) | 59.82 (37.52-86.24) | 0.45 (0.31-0.64) | 1.01 (0.63-1.46) | 2.69 (2.59,2.80) |  | 454.28 (309.58-646.98) | 1369.02 (835.71-1986.74) | 10.88 (7.42-15.50) | 23.12 (14.11-33.55) | 2.55 (2.42,2.68) |
| Turkey | 105.85 (70.14-157.73) | 294.54 (195.05-423.46) | 0.37 (0.24-0.55) | 0.70 (0.47-1.01) | 2.53 (2.09,2.98) |  | 116.09 (76.49-178.05) | 322.25 (215.21-464.16) | 0.40 (0.27-0.62) | 0.77 (0.51-1.11) | 2.53 (2.06,3.00) |  | 2707.35 (1790.24-3998.70) | 6680.25 (4343.46-9614.11) | 9.42 (6.23-13.92) | 15.98 (10.39-23.00) | 2.00 (1.55,2.44) |
| Turkmenistan | 18.32 (13.68-23.90) | 40.71 (27.43-58.17) | 0.99 (0.74-1.29) | 1.58 (1.06-2.26) | 1.81 (1.62,2.00) |  | 19.47 (14.64-25.24) | 42.77 (28.87-60.95) | 1.05 (0.79-1.36) | 1.66 (1.12-2.36) | 1.66 (1.30,2.02) |  | 512.74 (377.32-689.18) | 1156.42 (762.98-1688.63) | 27.72 (20.40-37.26) | 44.84 (29.58-65.47) | 1.87 (1.50,2.24) |
| Tuvalu | 0.03 (0.02-0.06) | 0.05 (0.03-0.08) | 0.67 (0.39-1.16) | 0.82 (0.50-1.32) | 0.38 (0.22,0.54) |  | 0.03 (0.02-0.06) | 0.05 (0.03-0.09) | 0.71 (0.42-1.23) | 0.87 (0.54-1.40) | 0.40 (0.22,0.58) |  | 0.87 (0.51-1.53) | 1.32 (0.79-2.15) | 18.39 (10.79-32.17) | 21.29 (12.85-34.70) | 0.29 (0.16,0.43) |
| Uganda | 70.76 (47.51-105.79) | 135.76 (86.27-197.27) | 0.82 (0.55-1.22) | 0.63 (0.40-0.91) | -1.44 (-1.64,-1.24) |  | 76.07 (51.03-113.77) | 145.87 (92.18-209.27) | 0.88 (0.59-1.32) | 0.67 (0.43-0.97) | -1.41 (-1.62,-1.20) |  | 1919.72 (1269.14-2910.63) | 3742.05 (2390.03-5550.52) | 22.20 (14.68-33.67) | 17.28 (11.04-25.63) | -1.41 (-1.64,-1.18) |
| Ukraine | 175.19 (139.44-217.10) | 145.16 (107.18-191.23) | 0.66 (0.53-0.82) | 0.67 (0.50-0.89) | -0.91 (-1.52,-0.29) |  | 185.63 (147.14-229.99) | 153.26 (112.57-201.04) | 0.70 (0.56-0.87) | 0.71 (0.52-0.93) | -0.66 (-1.31,0.00) |  | 4418.21 (3548.83-5483.81) | 3504.21 (2534.16-4622.01) | 16.76 (13.46-20.81) | 16.27 (11.77-21.46) | -0.85 (-1.51,-0.18) |
| United Arab Emirates | 5.72 (3.61-8.77) | 57.07 (36.49-86.08) | 0.61 (0.39-0.94) | 1.19 (0.76-1.79) | 0.83 (0.10,1.56) |  | 5.95 (3.80-9.11) | 56.46 (36.27-84.31) | 0.64 (0.41-0.97) | 1.17 (0.75-1.75) | 0.68 (-0.02,1.38) |  | 166.17 (103.06-259.49) | 1720.67 (1057.39-2622.36) | 17.76 (11.02-27.74) | 35.73 (21.96-54.46) | 1.04 (0.36,1.72) |
| United Kingdom | 327.88 (287.15-369.40) | 1530.94 (1308.92-1734.53) | 1.14 (1.00-1.29) | 4.51 (3.86-5.11) | 5.42 (5.16,5.68) |  | 329.66 (289.13-370.38) | 1424.66 (1221.60-1609.09) | 1.15 (1.01-1.29) | 4.20 (3.60-4.74) | 5.01 (4.74,5.29) |  | 6554.79 (5737.87-7438.86) | 25326.01 (22007.87-28690.74) | 22.88 (20.03-25.96) | 74.65 (64.87-84.57) | 4.68 (4.40,4.96) |
| United Republic of Tanzania | 89.14 (62.66-124.50) | 169.94 (110.67-246.22) | 0.69 (0.49-0.96) | 0.58 (0.38-0.84) | -0.85 (-0.98,-0.73) |  | 95.48 (67.32-134.21) | 183.64 (121.68-267.12) | 0.74 (0.52-1.04) | 0.63 (0.42-0.91) | -0.82 (-0.95,-0.69) |  | 2441.65 (1686.01-3534.48) | 4594.66 (2937.59-6726.03) | 18.90 (13.05-27.36) | 15.72 (10.05-23.02) | -0.91 (-1.05,-0.77) |
| United States of America | 1316.95 (1181.79-1442.47) | 6021.33 (5291.14-6705.95) | 1.04 (0.93-1.14) | 3.62 (3.18-4.03) | 4.10 (3.94,4.26) |  | 1195.16 (1070.88-1309.20) | 4922.18 (4300.09-5496.28) | 0.94 (0.84-1.03) | 2.96 (2.59-3.30) | 3.76 (3.66,3.85) |  | 26237.56 (23726.51-28714.45) | 106704.86 (94763.21-119357.13) | 20.65 (18.68-22.60) | 64.16 (56.98-71.76) | 3.89 (3.74,4.05) |
| United States Virgin Islands | 0.20 (0.13-0.31) | 0.51 (0.30-0.77) | 0.37 (0.25-0.58) | 1.18 (0.70-1.79) | 3.73 (3.52,3.94) |  | 0.22 (0.15-0.34) | 0.56 (0.34-0.83) | 0.41 (0.28-0.64) | 1.30 (0.78-1.94) | 3.77 (3.56,3.97) |  | 4.95 (3.32-7.83) | 11.56 (6.79-17.80) | 9.33 (6.25-14.77) | 26.91 (15.81-41.45) | 3.49 (3.30,3.69) |
| Uruguay | 6.14 (4.56-8.14) | 21.80 (15.93-28.48) | 0.39 (0.29-0.52) | 1.28 (0.94-1.67) | 4.56 (4.30,4.83) |  | 6.70 (5.01-8.82) | 23.65 (17.28-30.63) | 0.43 (0.32-0.56) | 1.39 (1.01-1.80) | 4.57 (4.25,4.89) |  | 148.63 (109.50-201.98) | 493.21 (360.23-641.25) | 9.47 (6.98-12.87) | 28.96 (21.15-37.66) | 4.34 (4.02,4.65) |
| Uzbekistan | 70.35 (46.94-97.39) | 222.19 (149.66-310.03) | 0.67 (0.45-0.93) | 1.30 (0.87-1.81) | 1.94 (1.82,2.07) |  | 74.92 (49.90-103.02) | 232.29 (156.13-325.15) | 0.71 (0.48-0.98) | 1.36 (0.91-1.90) | 1.93 (1.80,2.05) |  | 1966.82 (1319.26-2814.17) | 6323.77 (4288.32-8805.78) | 18.77 (12.59-26.85) | 36.95 (25.05-51.45) | 2.08 (1.95,2.20) |
| Vanuatu | 0.26 (0.14-0.50) | 0.73 (0.42-1.22) | 0.34 (0.18-0.66) | 0.46 (0.27-0.78) | 1.08 (0.96,1.19) |  | 0.28 (0.15-0.54) | 0.78 (0.45-1.31) | 0.37 (0.20-0.71) | 0.50 (0.29-0.83) | 1.08 (0.94,1.22) |  | 7.31 (3.90-14.59) | 19.75 (11.28-33.56) | 9.61 (5.13-19.17) | 12.62 (7.21-21.45) | 0.97 (0.84,1.11) |
| Venezuela (Bolivarian Republic of) | 96.49 (75.38-120.94) | 136.08 (95.40-186.03) | 1.03 (0.80-1.29) | 1.02 (0.72-1.40) | 0.24 (-0.98,1.49) |  | 105.18 (81.91-131.28) | 149.58 (105.69-203.04) | 1.12 (0.87-1.40) | 1.12 (0.79-1.52) | 0.15 (-1.24,1.57) |  | 2459.46 (1927.16-3095.81) | 3324.12 (2281.36-4669.09) | 26.15 (20.49-32.92) | 24.97 (17.13-35.07) | -0.02 (-1.42,1.41) |
| Viet Nam | 475.80 (295.48-752.42) | 1030.82 (604.06-1636.73) | 1.39 (0.87-2.21) | 2.06 (1.20-3.26) | 1.48 (1.31,1.66) |  | 512.64 (327.49-807.97) | 1058.09 (618.38-1655.84) | 1.50 (0.96-2.37) | 2.11 (1.23-3.30) | 1.33 (1.19,1.48) |  | 12584.47 (7613.95-20392.81) | 26383.11 (15195.49-42448.10) | 36.89 (22.32-59.78) | 52.63 (30.31-84.67) | 1.42 (1.22,1.63) |
| Yemen | 31.15 (12.77-64.40) | 68.01 (38.04-122.64) | 0.46 (0.19-0.94) | 0.40 (0.23-0.73) | -0.58 (-0.79,-0.38) |  | 33.09 (13.60-69.36) | 72.98 (40.52-132.38) | 0.49 (0.20-1.02) | 0.43 (0.24-0.79) | -0.57 (-0.76,-0.38) |  | 872.88 (352.19-1783.11) | 1849.52 (1011.66-3338.95) | 12.80 (5.17-26.16) | 10.99 (6.01-19.85) | -0.71 (-0.92,-0.50) |
| Zambia | 36.64 (22.40-62.48) | 44.77 (19.34-100.40) | 0.92 (0.56-1.57) | 0.46 (0.20-1.03) | -3.65 (-4.15,-3.15) |  | 38.86 (23.86-65.96) | 48.50 (21.27-108.51) | 0.98 (0.60-1.66) | 0.50 (0.22-1.11) | -3.59 (-4.10,-3.07) |  | 1042.22 (637.27-1759.28) | 1219.94 (498.60-2859.02) | 26.26 (16.06-44.33) | 12.50 (5.11-29.30) | -3.86 (-4.41,-3.32) |
| Zimbabwe | 63.94 (40.96-95.00) | 160.78 (93.80-243.11) | 1.24 (0.79-1.84) | 2.06 (1.20-3.12) | 1.32 (0.73,1.92) |  | 69.17 (44.49-103.23) | 172.71 (100.78-256.75) | 1.34 (0.86-2.00) | 2.21 (1.29-3.29) | 1.32 (0.70,1.95) |  | 1683.27 (1051.07-2539.49) | 4395.38 (2535.58-6703.18) | 32.55 (20.32-49.11) | 56.37 (32.52-85.96) | 1.55 (0.94,2.16) |

**Abbreviations:** UI, uncertainty interval; ASR, age-standardised rate per 100,000; EAPC, estimated annual percentage change; CI, confidence interval; DALYs, disability-adjusted life-year; SDI, socio-demographic index.

**Table 12. Burden of liver cancer due to alcohol use in 204 countries and territories in 1990, 2021 and the estimated annual percentage changes (EAPC) from 1990 to 2021**

| **Country** | **Incidence** | | | | |  | **Deaths** | | | | |  | **DALYs** | | | | |
| --- | --- | --- | --- | --- | --- | --- | --- | --- | --- | --- | --- | --- | --- | --- | --- | --- | --- |
|  | **1990 No. (95% UI)** | **2021 No. (95% UI)** | **1990 ASR per 100 000 (95% UI)** | **2021 ASR per 100 000 (95% UI)** | **EAPC (95% CI)** |  | **1990 No. (95% UI)** | **2021 No. (95% UI)** | **1990 ASR per 100 000 (95% UI)** | **2021 ASR per 100 000 (95% UI)** | **EAPC (95% CI)** |  | **1990 No. (95% UI)** | **2021 No. (95% UI)** | **1990 ASR per 100 000 (95% UI)** | **2021 ASR per 100 000 (95% UI)** | **EAPC (95% CI)** |
| Afghanistan | 15.21 (7.98-24.86) | 20.27 (11.47-32.06) | 0.31 (0.16-0.50) | 0.13 (0.07-0.21) | -2.94 (-3.28,-2.59) |  | 16.12 (8.36-26.43) | 21.01 (12.06-33.26) | 0.32 (0.17-0.53) | 0.13 (0.08-0.21) | -2.98 (-3.31,-2.65) |  | 429.21 (224.43-710.42) | 624.45 (352.61-996.58) | 8.63 (4.51-14.29) | 4.00 (2.26-6.38) | -2.69 (-3.08,-2.30) |
| Albania | 33.26 (20.80-48.69) | 59.43 (35.66-89.63) | 2.01 (1.26-2.95) | 4.46 (2.67-6.72) | 2.64 (2.40,2.87) |  | 35.72 (22.42-51.55) | 64.14 (38.90-97.68) | 2.16 (1.36-3.12) | 4.81 (2.92-7.32) | 2.60 (2.31,2.90) |  | 883.34 (560.90-1321.34) | 1432.02 (865.85-2241.97) | 53.45 (33.94-79.96) | 107.35 (64.91-168.07) | 2.21 (1.93,2.49) |
| Algeria | 6.83 (4.21-10.50) | 29.07 (16.90-45.73) | 0.05 (0.03-0.08) | 0.13 (0.08-0.21) | 2.92 (2.71,3.14) |  | 7.25 (4.42-11.36) | 30.26 (17.51-47.58) | 0.06 (0.03-0.09) | 0.14 (0.08-0.22) | 2.82 (2.61,3.04) |  | 188.54 (119.49-291.07) | 774.13 (457.23-1219.49) | 1.49 (0.95-2.30) | 3.50 (2.07-5.52) | 2.79 (2.58,3.00) |
| American Samoa | 0.05 (0.03-0.08) | 0.16 (0.10-0.24) | 0.20 (0.12-0.32) | 0.62 (0.39-0.97) | 3.69 (3.57,3.81) |  | 0.05 (0.03-0.08) | 0.16 (0.10-0.24) | 0.21 (0.12-0.32) | 0.64 (0.40-0.98) | 3.80 (3.65,3.95) |  | 1.47 (0.87-2.31) | 4.46 (2.78-6.86) | 6.06 (3.59-9.52) | 17.93 (11.16-27.56) | 3.69 (3.54,3.84) |
| Andorra | 1.17 (0.77-1.78) | 2.69 (1.70-4.02) | 4.29 (2.84-6.53) | 6.28 (3.96-9.38) | 1.03 (0.80,1.27) |  | 1.15 (0.76-1.75) | 2.49 (1.57-3.80) | 4.23 (2.79-6.43) | 5.83 (3.67-8.89) | 0.87 (0.62,1.13) |  | 28.39 (18.94-43.20) | 57.46 (35.49-86.28) | 104.46 (69.69-158.92) | 134.27 (82.92-201.61) | 0.63 (0.38,0.88) |
| Angola | 39.97 (4.67-112.45) | 87.70 (18.56-235.51) | 0.78 (0.09-2.19) | 0.54 (0.11-1.44) | -1.25 (-1.38,-1.11) |  | 40.90 (4.74-115.40) | 89.88 (18.87-241.32) | 0.80 (0.09-2.25) | 0.55 (0.12-1.48) | -1.22 (-1.36,-1.08) |  | 1260.90 (146.88-3476.01) | 2774.28 (596.27-7308.20) | 24.54 (2.86-67.65) | 16.96 (3.65-44.69) | -1.18 (-1.31,-1.04) |
| Antigua and Barbuda | 0.22 (0.16-0.29) | 0.49 (0.37-0.62) | 0.74 (0.54-0.97) | 1.10 (0.84-1.39) | 0.98 (0.27,1.68) |  | 0.24 (0.18-0.32) | 0.52 (0.40-0.65) | 0.80 (0.58-1.06) | 1.16 (0.89-1.46) | 0.76 (0.10,1.43) |  | 5.64 (4.09-7.47) | 12.62 (9.54-15.97) | 18.74 (13.57-24.80) | 28.23 (21.35-35.74) | 0.93 (0.30,1.56) |
| Argentina | 30.71 (22.90-39.74) | 88.07 (64.90-116.16) | 0.19 (0.14-0.24) | 0.39 (0.29-0.51) | 3.25 (2.92,3.58) |  | 32.40 (24.04-41.72) | 92.02 (67.39-120.76) | 0.20 (0.15-0.25) | 0.40 (0.30-0.53) | 3.25 (2.84,3.66) |  | 828.74 (622.39-1096.64) | 2251.23 (1646.68-2937.33) | 5.01 (3.76-6.62) | 9.90 (7.24-12.91) | 3.11 (2.69,3.53) |
| Armenia | 21.63 (14.76-30.06) | 33.20 (23.13-45.92) | 1.26 (0.86-1.76) | 2.22 (1.54-3.07) | 1.99 (1.38,2.61) |  | 22.96 (15.45-32.01) | 35.37 (24.68-48.90) | 1.34 (0.90-1.87) | 2.36 (1.65-3.27) | 1.72 (1.00,2.44) |  | 593.64 (401.38-835.99) | 861.64 (599.66-1183.51) | 34.71 (23.47-48.88) | 57.53 (40.04-79.02) | 1.49 (0.88,2.12) |
| Australia | 76.31 (58.50-93.21) | 510.59 (396.57-643.03) | 0.91 (0.69-1.11) | 3.96 (3.08-4.99) | 5.10 (4.92,5.29) |  | 75.95 (58.01-93.74) | 445.08 (344.95-563.56) | 0.90 (0.69-1.11) | 3.45 (2.67-4.37) | 4.45 (4.33,4.56) |  | 1932.97 (1518.38-2360.72) | 10280.52 (7970.07-12755.75) | 22.93 (18.01-28.01) | 79.72 (61.80-98.91) | 4.22 (4.12,4.32) |
| Austria | 92.42 (76.79-107.35) | 286.02 (232.34-342.74) | 2.38 (1.98-2.76) | 6.37 (5.17-7.63) | 3.45 (3.13,3.76) |  | 90.66 (74.71-106.05) | 247.54 (199.71-295.54) | 2.33 (1.92-2.73) | 5.51 (4.45-6.58) | 2.94 (2.71,3.18) |  | 2146.61 (1798.34-2480.50) | 5292.66 (4385.03-6270.64) | 55.27 (46.30-63.86) | 117.85 (97.64-139.62) | 2.55 (2.30,2.81) |
| Azerbaijan | 37.90 (19.90-64.22) | 117.67 (62.33-200.40) | 1.03 (0.54-1.75) | 2.24 (1.19-3.82) | 2.33 (2.03,2.64) |  | 39.56 (20.89-66.65) | 122.47 (65.21-205.66) | 1.08 (0.57-1.82) | 2.33 (1.24-3.92) | 2.39 (2.09,2.69) |  | 1109.71 (560.01-1895.97) | 3354.15 (1764.54-5733.81) | 30.29 (15.29-51.76) | 63.89 (33.61-109.21) | 2.25 (1.87,2.62) |
| Bahamas | 0.98 (0.75-1.28) | 1.95 (1.43-2.61) | 0.76 (0.58-1.00) | 1.01 (0.74-1.35) | 0.75 (0.29,1.21) |  | 1.02 (0.78-1.34) | 2.02 (1.49-2.70) | 0.80 (0.61-1.04) | 1.04 (0.77-1.39) | 0.73 (0.27,1.19) |  | 28.20 (21.57-37.73) | 54.72 (39.87-74.19) | 21.97 (16.81-29.39) | 28.21 (20.55-38.25) | 0.63 (0.21,1.06) |
| Bahrain | 0.74 (0.47-1.11) | 1.92 (1.09-3.02) | 0.29 (0.19-0.44) | 0.25 (0.14-0.40) | -1.82 (-2.53,-1.10) |  | 0.77 (0.49-1.17) | 1.91 (1.08-3.00) | 0.30 (0.19-0.46) | 0.25 (0.14-0.39) | -2.07 (-2.82,-1.32) |  | 20.44 (13.23-31.03) | 52.23 (29.40-82.69) | 8.07 (5.22-12.25) | 6.83 (3.84-10.81) | -1.85 (-2.57,-1.11) |
| Bangladesh | 98.04 (63.60-145.40) | 287.02 (172.65-430.09) | 0.18 (0.12-0.27) | 0.35 (0.21-0.52) | 2.41 (2.25,2.58) |  | 101.87 (66.29-150.22) | 301.33 (181.90-455.95) | 0.19 (0.12-0.28) | 0.37 (0.22-0.55) | 2.51 (2.33,2.70) |  | 2947.19 (1911.22-4421.55) | 8043.93 (4756.46-12453.03) | 5.40 (3.50-8.10) | 9.77 (5.78-15.13) | 2.25 (2.09,2.40) |
| Barbados | 0.94 (0.70-1.25) | 2.24 (1.58-3.04) | 0.74 (0.55-0.99) | 1.50 (1.06-2.03) | 2.02 (1.72,2.33) |  | 1.03 (0.77-1.37) | 2.40 (1.72-3.31) | 0.81 (0.61-1.08) | 1.61 (1.15-2.21) | 1.90 (1.56,2.24) |  | 23.04 (17.23-30.48) | 54.50 (37.94-74.17) | 18.18 (13.60-24.06) | 36.45 (25.38-49.61) | 2.01 (1.71,2.30) |
| Belarus | 55.03 (40.31-72.61) | 82.19 (57.80-111.00) | 1.05 (0.77-1.39) | 1.76 (1.24-2.38) | 1.34 (1.11,1.58) |  | 57.41 (41.82-75.72) | 84.54 (58.83-112.85) | 1.10 (0.80-1.45) | 1.81 (1.26-2.42) | 1.29 (1.06,1.53) |  | 1530.33 (1113.32-2009.40) | 2178.31 (1515.73-2982.37) | 29.30 (21.32-38.48) | 46.72 (32.51-63.97) | 1.19 (0.95,1.43) |
| Belgium | 81.97 (63.40-103.56) | 173.69 (132.44-222.64) | 1.64 (1.27-2.08) | 3.03 (2.31-3.88) | 2.05 (1.79,2.31) |  | 85.42 (65.19-107.97) | 168.50 (128.10-217.69) | 1.71 (1.31-2.16) | 2.94 (2.23-3.80) | 1.89 (1.52,2.26) |  | 1891.06 (1453.88-2382.80) | 3611.18 (2777.63-4532.05) | 37.90 (29.14-47.76) | 62.97 (48.44-79.03) | 1.78 (1.46,2.09) |
| Belize | 0.24 (0.17-0.32) | 0.99 (0.71-1.31) | 0.25 (0.18-0.34) | 0.46 (0.33-0.61) | 1.40 (1.09,1.71) |  | 0.25 (0.18-0.35) | 1.03 (0.75-1.35) | 0.27 (0.19-0.37) | 0.48 (0.35-0.63) | 1.27 (0.84,1.70) |  | 6.34 (4.51-8.69) | 28.19 (20.29-37.20) | 6.78 (4.82-9.30) | 13.14 (9.46-17.34) | 1.62 (1.21,2.03) |
| Benin | 32.66 (16.04-53.48) | 53.56 (33.88-79.80) | 1.35 (0.66-2.20) | 0.79 (0.50-1.18) | -2.16 (-2.33,-1.98) |  | 34.48 (16.86-56.37) | 55.94 (35.44-83.93) | 1.42 (0.70-2.32) | 0.83 (0.53-1.24) | -2.13 (-2.31,-1.94) |  | 950.88 (465.86-1512.94) | 1617.75 (1031.68-2510.68) | 39.21 (19.21-62.38) | 23.97 (15.29-37.20) | -1.99 (-2.19,-1.80) |
| Bermuda | 0.38 (0.30-0.48) | 0.40 (0.29-0.52) | 1.28 (1.00-1.60) | 1.26 (0.93-1.63) | 0.02 (-0.56,0.61) |  | 0.40 (0.32-0.51) | 0.41 (0.30-0.53) | 1.36 (1.06-1.70) | 1.28 (0.95-1.67) | -0.26 (-0.87,0.36) |  | 9.79 (7.72-12.12) | 8.94 (6.60-11.51) | 32.98 (26.02-40.82) | 28.14 (20.77-36.21) | -0.60 (-1.20,0.00) |
| Bhutan | 1.05 (0.54-1.74) | 2.61 (1.52-4.06) | 0.33 (0.17-0.55) | 0.69 (0.40-1.07) | 2.01 (1.88,2.15) |  | 1.08 (0.56-1.78) | 2.74 (1.59-4.29) | 0.34 (0.18-0.56) | 0.72 (0.42-1.13) | 2.12 (1.99,2.26) |  | 32.81 (16.47-55.79) | 73.19 (42.20-114.23) | 10.42 (5.23-17.71) | 19.34 (11.15-30.18) | 1.68 (1.55,1.81) |
| Bolivia (Plurinational State of) | 12.40 (7.61-19.16) | 41.96 (25.39-65.90) | 0.39 (0.24-0.60) | 0.71 (0.43-1.12) | 2.12 (1.96,2.27) |  | 13.25 (8.16-20.25) | 45.40 (27.39-71.13) | 0.42 (0.26-0.63) | 0.77 (0.46-1.21) | 2.19 (2.03,2.36) |  | 341.78 (212.48-521.52) | 1087.28 (648.68-1726.16) | 10.71 (6.66-16.35) | 18.43 (11.00-29.26) | 1.88 (1.70,2.07) |
| Bosnia and Herzegovina | 39.85 (28.57-55.71) | 69.28 (47.54-96.70) | 1.77 (1.27-2.48) | 4.19 (2.88-5.86) | 2.13 (1.85,2.41) |  | 41.74 (29.95-58.45) | 74.12 (50.63-103.98) | 1.86 (1.33-2.60) | 4.49 (3.07-6.30) | 2.17 (1.91,2.42) |  | 1121.38 (792.51-1545.52) | 1692.34 (1153.92-2346.29) | 49.86 (35.24-68.72) | 102.48 (69.87-142.08) | 1.53 (1.23,1.83) |
| Botswana | 2.65 (1.14-5.11) | 8.54 (3.82-19.37) | 0.40 (0.17-0.78) | 0.71 (0.32-1.62) | 0.59 (0.08,1.10) |  | 2.77 (1.21-5.30) | 8.83 (3.92-19.64) | 0.42 (0.18-0.80) | 0.74 (0.33-1.64) | 0.62 (0.07,1.17) |  | 79.38 (34.11-160.21) | 261.37 (112.56-627.24) | 12.03 (5.17-24.29) | 21.84 (9.41-52.42) | 0.69 (0.11,1.28) |
| Brazil | 212.02 (185.83-240.00) | 689.69 (597.97-786.18) | 0.29 (0.25-0.32) | 0.63 (0.54-0.71) | 3.22 (2.96,3.49) |  | 222.22 (194.79-250.98) | 724.49 (629.00-823.93) | 0.30 (0.26-0.34) | 0.66 (0.57-0.75) | 3.32 (3.05,3.58) |  | 6115.66 (5371.89-6969.61) | 18472.89 (15886.51-21179.81) | 8.24 (7.23-9.39) | 16.77 (14.42-19.22) | 3.10 (2.82,3.38) |
| Brunei Darussalam | 0.61 (0.37-0.98) | 1.41 (0.90-2.18) | 0.47 (0.28-0.75) | 0.63 (0.40-0.97) | 0.87 (0.59,1.15) |  | 0.62 (0.37-0.99) | 1.37 (0.88-2.15) | 0.48 (0.29-0.77) | 0.61 (0.39-0.95) | 0.68 (0.35,1.01) |  | 16.88 (10.01-27.44) | 37.56 (23.80-58.78) | 13.02 (7.72-21.17) | 16.65 (10.55-26.06) | 0.70 (0.36,1.03) |
| Bulgaria | 181.23 (129.55-230.33) | 105.44 (73.47-145.95) | 4.18 (2.99-5.31) | 3.11 (2.17-4.30) | -0.83 (-1.25,-0.41) |  | 192.39 (138.25-246.13) | 113.10 (78.73-156.14) | 4.43 (3.19-5.67) | 3.33 (2.32-4.60) | -0.74 (-1.25,-0.22) |  | 4836.20 (3462.54-6236.95) | 2683.72 (1888.18-3704.27) | 111.43 (79.78-143.71) | 79.08 (55.64-109.16) | -0.91 (-1.40,-0.41) |
| Burkina Faso | 118.30 (54.02-235.28) | 182.73 (79.29-312.83) | 2.48 (1.13-4.94) | 1.61 (0.70-2.75) | -1.67 (-1.85,-1.49) |  | 123.60 (56.01-243.86) | 190.45 (82.14-327.89) | 2.59 (1.18-5.12) | 1.67 (0.72-2.88) | -1.67 (-1.86,-1.48) |  | 3498.03 (1592.25-6975.95) | 5576.34 (2356.84-9849.46) | 73.43 (33.42-146.43) | 49.00 (20.71-86.55) | -1.53 (-1.74,-1.32) |
| Burundi | 13.34 (6.95-27.53) | 13.23 (6.99-28.55) | 0.48 (0.25-0.99) | 0.20 (0.11-0.43) | -3.78 (-4.18,-3.39) |  | 13.90 (7.18-28.30) | 13.69 (7.13-29.47) | 0.50 (0.26-1.02) | 0.21 (0.11-0.45) | -3.78 (-4.18,-3.38) |  | 400.40 (212.02-824.22) | 409.80 (212.36-895.67) | 14.42 (7.64-29.68) | 6.20 (3.21-13.55) | -3.65 (-4.04,-3.25) |
| Cabo Verde | 1.69 (1.03-2.89) | 5.11 (3.09-7.72) | 0.95 (0.58-1.63) | 1.83 (1.11-2.76) | 1.74 (1.44,2.04) |  | 1.85 (1.12-3.23) | 5.42 (3.25-8.23) | 1.05 (0.63-1.82) | 1.94 (1.16-2.94) | 1.57 (1.23,1.91) |  | 44.12 (26.87-73.44) | 142.25 (86.58-219.15) | 24.94 (15.19-41.52) | 50.87 (30.96-78.37) | 1.97 (1.70,2.25) |
| Cambodia | 28.39 (12.48-60.43) | 99.94 (46.34-201.64) | 0.55 (0.24-1.18) | 1.17 (0.54-2.37) | 2.36 (2.01,2.70) |  | 29.49 (13.02-62.94) | 103.12 (47.03-210.90) | 0.57 (0.25-1.23) | 1.21 (0.55-2.47) | 2.34 (2.01,2.68) |  | 855.02 (387.97-1814.98) | 2894.55 (1356.67-6002.23) | 16.65 (7.55-35.34) | 33.97 (15.92-70.43) | 2.23 (1.90,2.56) |
| Cameroon | 73.22 (44.48-122.64) | 155.94 (79.56-287.83) | 1.40 (0.85-2.35) | 0.98 (0.50-1.81) | -1.62 (-1.81,-1.43) |  | 76.04 (46.49-126.79) | 160.69 (83.01-295.67) | 1.46 (0.89-2.43) | 1.01 (0.52-1.86) | -1.63 (-1.82,-1.43) |  | 2221.29 (1319.63-3683.16) | 4830.96 (2461.15-9038.01) | 42.57 (25.29-70.58) | 30.40 (15.49-56.88) | -1.53 (-1.74,-1.33) |
| Canada | 168.30 (139.79-199.68) | 903.83 (737.60-1078.05) | 1.24 (1.03-1.47) | 4.82 (3.94-5.75) | 4.76 (4.68,4.84) |  | 160.83 (133.31-192.29) | 813.48 (653.36-972.79) | 1.18 (0.98-1.41) | 4.34 (3.49-5.19) | 4.68 (4.58,4.78) |  | 4048.47 (3376.64-4824.00) | 18114.82 (14848.28-21561.99) | 29.71 (24.78-35.40) | 96.69 (79.26-115.09) | 4.26 (4.15,4.36) |
| Central African Republic | 11.49 (4.42-22.20) | 11.92 (4.79-24.74) | 0.84 (0.32-1.63) | 0.43 (0.17-0.90) | -2.73 (-2.94,-2.52) |  | 11.73 (4.50-22.63) | 12.05 (4.83-25.38) | 0.86 (0.33-1.66) | 0.44 (0.18-0.93) | -2.80 (-3.02,-2.57) |  | 362.32 (137.15-695.77) | 391.17 (158.68-806.88) | 26.54 (10.05-50.96) | 14.27 (5.79-29.43) | -2.62 (-2.83,-2.41) |
| Chad | 37.39 (14.49-76.75) | 67.72 (37.07-125.08) | 1.24 (0.48-2.55) | 0.76 (0.42-1.41) | -1.95 (-2.15,-1.76) |  | 39.59 (15.33-80.93) | 70.67 (38.98-129.92) | 1.31 (0.51-2.69) | 0.80 (0.44-1.46) | -1.97 (-2.17,-1.76) |  | 1074.61 (427.70-2111.34) | 2035.96 (1107.57-3683.53) | 35.66 (14.19-70.07) | 22.94 (12.48-41.51) | -1.77 (-2.00,-1.55) |
| Chile | 22.78 (16.33-30.44) | 121.19 (87.59-158.65) | 0.34 (0.25-0.46) | 1.29 (0.93-1.69) | 4.94 (4.52,5.36) |  | 24.05 (17.33-32.19) | 123.93 (89.09-162.22) | 0.36 (0.26-0.48) | 1.32 (0.95-1.73) | 4.89 (4.51,5.26) |  | 604.83 (434.71-810.84) | 2852.93 (2064.02-3686.92) | 9.11 (6.54-12.21) | 30.35 (21.96-39.23) | 4.60 (4.24,4.97) |
| China | 3749.99 (2886.01-4781.70) | 10231.85 (7619.56-13647.86) | 0.64 (0.49-0.81) | 1.44 (1.07-1.92) | 2.96 (2.71,3.20) |  | 3787.25 (2929.01-4838.58) | 9158.54 (6826.49-12125.82) | 0.64 (0.50-0.82) | 1.29 (0.96-1.70) | 2.52 (2.32,2.72) |  | 113754.64 (87266.81-146517.14) | 238923.46 (176259.02-318877.37) | 19.34 (14.84-24.91) | 33.59 (24.78-44.83) | 2.00 (1.81,2.19) |
| Colombia | 63.79 (48.25-82.90) | 166.72 (115.67-220.44) | 0.39 (0.30-0.51) | 0.68 (0.47-0.90) | 1.78 (1.04,2.51) |  | 67.88 (51.37-87.52) | 179.27 (123.49-239.57) | 0.42 (0.32-0.54) | 0.73 (0.50-0.98) | 1.81 (1.08,2.54) |  | 1759.29 (1318.64-2267.35) | 4029.45 (2814.66-5428.12) | 10.83 (8.12-13.96) | 16.43 (11.47-22.13) | 1.28 (0.57,2.00) |
| Comoros | 0.84 (0.43-1.37) | 1.64 (0.89-2.74) | 0.36 (0.19-0.59) | 0.44 (0.24-0.74) | 0.25 (0.05,0.46) |  | 0.88 (0.45-1.43) | 1.73 (0.93-2.95) | 0.38 (0.20-0.62) | 0.46 (0.25-0.79) | 0.28 (0.09,0.48) |  | 24.38 (12.76-40.61) | 47.69 (25.62-81.79) | 10.54 (5.52-17.56) | 12.81 (6.88-21.98) | 0.24 (0.00,0.47) |
| Congo | 10.15 (4.58-18.79) | 15.99 (7.72-32.14) | 0.84 (0.38-1.56) | 0.59 (0.29-1.19) | -1.72 (-2.20,-1.24) |  | 10.45 (4.69-19.40) | 16.28 (7.92-32.03) | 0.87 (0.39-1.62) | 0.60 (0.29-1.19) | -1.74 (-2.20,-1.27) |  | 311.95 (138.92-575.09) | 505.02 (237.33-1016.52) | 25.98 (11.57-47.89) | 18.73 (8.80-37.71) | -1.61 (-2.09,-1.13) |
| Cook Islands | 0.07 (0.04-0.11) | 0.33 (0.21-0.50) | 0.74 (0.45-1.16) | 3.68 (2.39-5.62) | 5.81 (5.64,5.99) |  | 0.07 (0.04-0.11) | 0.32 (0.21-0.49) | 0.77 (0.47-1.19) | 3.65 (2.35-5.58) | 5.72 (5.52,5.92) |  | 2.01 (1.23-3.17) | 8.40 (5.37-12.92) | 21.19 (13.01-33.54) | 94.66 (60.55-145.61) | 5.54 (5.33,5.75) |
| Costa Rica | 11.54 (8.71-14.84) | 44.43 (32.28-59.34) | 0.76 (0.57-0.98) | 1.87 (1.36-2.50) | 2.63 (2.00,3.27) |  | 12.29 (9.23-15.83) | 46.64 (33.84-62.43) | 0.81 (0.61-1.04) | 1.96 (1.43-2.63) | 2.75 (2.13,3.38) |  | 303.55 (229.66-390.99) | 1100.33 (803.08-1463.30) | 19.96 (15.10-25.71) | 46.35 (33.83-61.64) | 2.50 (1.89,3.12) |
| Côte d'Ivoire | 27.68 (15.15-43.74) | 46.68 (25.72-77.79) | 0.45 (0.25-0.72) | 0.34 (0.18-0.56) | -1.68 (-1.92,-1.45) |  | 28.42 (15.88-44.80) | 47.98 (26.56-79.00) | 0.47 (0.26-0.73) | 0.34 (0.19-0.57) | -1.62 (-1.86,-1.39) |  | 861.09 (463.56-1356.79) | 1431.46 (806.54-2370.98) | 14.12 (7.60-22.25) | 10.28 (5.79-17.02) | -1.67 (-1.92,-1.43) |
| Croatia | 31.72 (24.35-40.72) | 65.86 (50.02-84.88) | 1.31 (1.00-1.68) | 3.13 (2.38-4.03) | 3.39 (2.85,3.94) |  | 32.76 (25.13-42.02) | 64.16 (48.97-82.97) | 1.35 (1.03-1.73) | 3.05 (2.33-3.94) | 3.30 (2.77,3.84) |  | 815.19 (608.47-1046.03) | 1395.39 (1057.48-1791.47) | 33.54 (25.03-43.03) | 66.31 (50.25-85.13) | 2.80 (2.28,3.32) |
| Cuba | 30.36 (22.60-39.48) | 45.49 (33.38-59.38) | 0.56 (0.42-0.73) | 0.81 (0.59-1.05) | 0.72 (0.30,1.15) |  | 32.47 (24.22-42.57) | 47.39 (34.91-62.18) | 0.60 (0.45-0.78) | 0.84 (0.62-1.10) | 0.54 (0.01,1.06) |  | 778.50 (585.37-1017.97) | 1139.81 (832.37-1511.73) | 14.35 (10.79-18.77) | 20.23 (14.77-26.83) | 0.57 (0.09,1.06) |
| Cyprus | 4.29 (2.81-6.13) | 12.30 (8.23-18.35) | 1.10 (0.72-1.58) | 1.81 (1.21-2.70) | 1.86 (1.70,2.02) |  | 4.52 (2.95-6.47) | 11.88 (7.92-17.53) | 1.16 (0.76-1.66) | 1.75 (1.17-2.58) | 1.53 (1.37,1.69) |  | 105.61 (69.74-149.58) | 255.53 (172.88-377.03) | 27.15 (17.93-38.45) | 37.64 (25.47-55.54) | 1.26 (1.09,1.44) |
| Czechia | 145.10 (115.04-177.56) | 137.84 (104.07-176.40) | 2.82 (2.23-3.45) | 2.59 (1.96-3.32) | -0.44 (-0.61,-0.26) |  | 154.94 (122.55-189.12) | 144.06 (108.87-184.86) | 3.01 (2.38-3.67) | 2.71 (2.05-3.48) | -0.57 (-0.85,-0.29) |  | 3768.65 (3013.66-4591.20) | 3109.10 (2368.17-4015.37) | 73.22 (58.55-89.20) | 58.48 (44.55-75.53) | -0.94 (-1.20,-0.67) |
| Democratic People's Republic of Korea | 58.76 (30.09-99.84) | 87.65 (51.21-142.27) | 0.57 (0.29-0.97) | 0.66 (0.39-1.08) | 0.26 (0.18,0.35) |  | 59.52 (30.65-102.26) | 87.66 (51.60-145.49) | 0.58 (0.30-0.99) | 0.66 (0.39-1.10) | 0.24 (0.15,0.33) |  | 1850.40 (921.24-3213.57) | 2533.84 (1477.91-4199.35) | 17.97 (8.95-31.21) | 19.20 (11.20-31.82) | -0.04 (-0.13,0.06) |
| Democratic Republic of the Congo | 46.61 (19.57-101.77) | 80.06 (32.05-187.60) | 0.24 (0.10-0.53) | 0.18 (0.07-0.42) | -1.42 (-1.75,-1.08) |  | 48.27 (20.18-104.98) | 82.61 (32.86-191.59) | 0.25 (0.11-0.55) | 0.18 (0.07-0.43) | -1.45 (-1.78,-1.13) |  | 1415.63 (583.63-3126.08) | 2509.27 (993.96-5959.95) | 7.42 (3.06-16.39) | 5.58 (2.21-13.24) | -1.29 (-1.62,-0.95) |
| Denmark | 26.61 (20.60-33.65) | 76.86 (56.79-99.22) | 1.03 (0.80-1.31) | 2.63 (1.94-3.39) | 2.53 (2.26,2.80) |  | 22.36 (17.29-28.60) | 68.01 (49.71-88.54) | 0.87 (0.67-1.11) | 2.32 (1.70-3.03) | 2.96 (2.63,3.28) |  | 516.30 (404.42-646.35) | 1420.93 (1059.41-1816.11) | 20.07 (15.72-25.13) | 48.56 (36.21-62.07) | 2.70 (2.34,3.06) |
| Djibouti | 0.48 (0.25-0.89) | 2.51 (1.44-4.35) | 0.23 (0.12-0.43) | 0.40 (0.23-0.69) | 1.43 (1.19,1.67) |  | 0.49 (0.25-0.90) | 2.57 (1.47-4.42) | 0.24 (0.12-0.44) | 0.41 (0.23-0.70) | 1.49 (1.23,1.74) |  | 15.31 (7.82-27.76) | 77.17 (42.96-131.98) | 7.39 (3.78-13.40) | 12.26 (6.83-20.97) | 1.37 (1.10,1.65) |
| Dominica | 0.26 (0.17-0.37) | 0.49 (0.33-0.69) | 0.72 (0.48-1.01) | 1.46 (0.97-2.07) | 1.94 (1.68,2.21) |  | 0.29 (0.19-0.40) | 0.53 (0.36-0.76) | 0.79 (0.52-1.10) | 1.57 (1.06-2.26) | 1.99 (1.71,2.27) |  | 6.53 (4.45-9.22) | 12.48 (8.11-17.93) | 18.03 (12.29-25.45) | 37.22 (24.18-53.46) | 2.15 (1.87,2.44) |
| Dominican Republic | 7.21 (4.75-10.07) | 28.79 (19.75-41.40) | 0.20 (0.13-0.28) | 0.52 (0.36-0.75) | 3.33 (3.20,3.45) |  | 7.62 (4.99-10.57) | 30.38 (20.74-43.96) | 0.21 (0.14-0.30) | 0.55 (0.38-0.80) | 3.30 (3.16,3.44) |  | 206.23 (137.67-288.31) | 798.98 (539.69-1149.12) | 5.77 (3.85-8.06) | 14.51 (9.80-20.87) | 3.20 (3.06,3.34) |
| Ecuador | 21.16 (15.38-28.98) | 53.60 (36.67-73.59) | 0.42 (0.31-0.58) | 0.59 (0.41-0.81) | 1.55 (0.91,2.20) |  | 22.84 (16.60-31.28) | 58.45 (40.29-80.45) | 0.46 (0.33-0.63) | 0.65 (0.45-0.89) | 1.38 (0.72,2.04) |  | 572.09 (415.92-773.55) | 1318.66 (914.68-1847.94) | 11.47 (8.34-15.50) | 14.60 (10.13-20.46) | 0.97 (0.32,1.62) |
| Egypt | 130.25 (73.64-228.85) | 525.58 (315.75-859.88) | 0.47 (0.27-0.83) | 1.00 (0.60-1.63) | 2.43 (2.32,2.55) |  | 135.21 (75.78-242.02) | 537.46 (320.04-889.64) | 0.49 (0.27-0.87) | 1.02 (0.61-1.68) | 2.34 (2.17,2.50) |  | 3831.33 (2152.17-6597.51) | 15023.76 (9034.37-25184.80) | 13.85 (7.78-23.85) | 28.45 (17.11-47.69) | 2.33 (2.21,2.46) |
| El Salvador | 5.92 (4.10-8.01) | 11.83 (8.13-16.24) | 0.22 (0.15-0.30) | 0.37 (0.25-0.50) | 1.65 (1.21,2.09) |  | 6.31 (4.36-8.56) | 12.51 (8.53-17.39) | 0.24 (0.16-0.32) | 0.39 (0.26-0.54) | 1.50 (1.03,1.97) |  | 163.75 (113.44-222.13) | 313.76 (218.93-433.80) | 6.17 (4.28-8.37) | 9.73 (6.79-13.45) | 1.41 (0.92,1.90) |
| Equatorial Guinea | 0.43 (0.18-1.20) | 2.35 (1.31-4.28) | 0.20 (0.08-0.57) | 0.31 (0.17-0.57) | 1.66 (1.05,2.28) |  | 0.45 (0.19-1.24) | 2.40 (1.34-4.36) | 0.21 (0.09-0.59) | 0.32 (0.18-0.58) | 1.67 (1.06,2.28) |  | 13.33 (5.45-36.90) | 73.04 (39.49-132.52) | 6.30 (2.58-17.46) | 9.66 (5.22-17.53) | 1.70 (1.07,2.35) |
| Eritrea | 4.05 (2.26-6.96) | 8.42 (4.58-16.24) | 0.24 (0.13-0.41) | 0.26 (0.14-0.49) | -0.58 (-0.81,-0.36) |  | 4.11 (2.30-7.00) | 8.59 (4.73-16.40) | 0.24 (0.14-0.41) | 0.26 (0.14-0.50) | -0.49 (-0.73,-0.25) |  | 132.32 (73.78-225.45) | 267.53 (141.68-521.79) | 7.77 (4.33-13.24) | 8.11 (4.29-15.82) | -0.62 (-0.86,-0.37) |
| Estonia | 10.47 (7.74-13.79) | 19.58 (14.79-25.18) | 1.34 (0.99-1.76) | 2.99 (2.26-3.84) | 2.39 (2.24,2.54) |  | 10.93 (8.13-14.39) | 20.59 (15.54-26.80) | 1.39 (1.04-1.83) | 3.14 (2.37-4.09) | 2.63 (2.39,2.86) |  | 290.81 (214.94-383.54) | 460.66 (350.37-598.85) | 37.08 (27.41-48.91) | 70.28 (53.46-91.37) | 1.99 (1.75,2.23) |
| Eswatini | 2.39 (0.73-4.52) | 12.38 (4.10-28.99) | 0.59 (0.18-1.12) | 2.14 (0.71-5.02) | 4.25 (2.97,5.54) |  | 2.47 (0.77-4.71) | 12.65 (4.17-29.81) | 0.61 (0.19-1.17) | 2.19 (0.72-5.16) | 4.25 (2.95,5.58) |  | 72.69 (21.90-139.60) | 398.90 (126.63-975.33) | 18.03 (5.43-34.62) | 69.06 (21.92-168.85) | 4.50 (3.12,5.89) |
| Ethiopia | 58.15 (41.71-90.06) | 89.88 (60.04-148.00) | 0.23 (0.16-0.36) | 0.17 (0.11-0.27) | -1.78 (-2.13,-1.42) |  | 60.22 (43.15-93.58) | 94.19 (62.78-155.01) | 0.24 (0.17-0.37) | 0.17 (0.12-0.28) | -1.72 (-2.07,-1.36) |  | 1770.17 (1288.94-2755.06) | 2631.14 (1760.00-4262.85) | 7.00 (5.10-10.90) | 4.83 (3.23-7.83) | -1.92 (-2.28,-1.55) |
| Fiji | 0.82 (0.48-1.34) | 2.22 (1.29-3.60) | 0.22 (0.13-0.35) | 0.48 (0.28-0.78) | 3.01 (2.77,3.24) |  | 0.84 (0.49-1.39) | 2.28 (1.32-3.71) | 0.22 (0.13-0.37) | 0.49 (0.29-0.80) | 3.10 (2.81,3.38) |  | 25.32 (14.73-41.60) | 65.13 (36.81-108.44) | 6.68 (3.89-10.97) | 14.09 (7.96-23.46) | 2.96 (2.66,3.25) |
| Finland | 39.96 (29.78-51.65) | 125.83 (90.66-163.57) | 1.60 (1.19-2.06) | 4.55 (3.28-5.91) | 3.75 (3.61,3.89) |  | 35.79 (26.60-46.34) | 97.71 (69.78-126.70) | 1.43 (1.06-1.85) | 3.53 (2.52-4.58) | 3.31 (3.12,3.50) |  | 835.39 (629.23-1063.38) | 1967.66 (1426.94-2545.16) | 33.35 (25.12-42.45) | 71.09 (51.55-91.95) | 2.84 (2.64,3.03) |
| France | 730.32 (552.51-923.15) | 1455.56 (1032.13-1942.94) | 2.53 (1.91-3.20) | 4.38 (3.11-5.85) | 1.56 (1.37,1.74) |  | 744.41 (561.97-934.41) | 1264.42 (887.01-1685.65) | 2.58 (1.95-3.23) | 3.81 (2.67-5.08) | 0.93 (0.69,1.18) |  | 17776.45 (13358.24-22913.76) | 27181.54 (19472.13-35751.31) | 61.54 (46.25-79.33) | 81.88 (58.66-107.70) | 0.62 (0.39,0.87) |
| Gabon | 5.53 (2.22-11.23) | 9.81 (5.23-17.06) | 1.13 (0.45-2.28) | 1.08 (0.58-1.88) | -0.51 (-0.72,-0.29) |  | 5.75 (2.32-11.77) | 10.04 (5.41-17.28) | 1.17 (0.47-2.39) | 1.11 (0.60-1.90) | -0.51 (-0.71,-0.30) |  | 163.65 (64.32-330.30) | 297.21 (158.00-515.61) | 33.28 (13.08-67.17) | 32.73 (17.40-56.78) | -0.35 (-0.54,-0.16) |
| Gambia | 8.71 (5.48-12.90) | 29.78 (16.72-48.76) | 1.77 (1.12-2.63) | 2.49 (1.40-4.07) | 0.71 (0.47,0.95) |  | 8.94 (5.70-13.26) | 30.72 (17.37-51.88) | 1.82 (1.16-2.70) | 2.57 (1.45-4.33) | 0.79 (0.54,1.04) |  | 272.94 (170.19-417.07) | 919.64 (519.87-1551.76) | 55.61 (34.68-84.98) | 76.82 (43.43-129.63) | 0.67 (0.41,0.93) |
| Georgia | 34.22 (23.49-46.83) | 34.05 (24.28-45.99) | 1.24 (0.85-1.70) | 1.89 (1.35-2.55) | 0.85 (-0.41,2.13) |  | 35.65 (24.41-48.96) | 35.94 (25.86-48.60) | 1.29 (0.88-1.77) | 1.99 (1.43-2.69) | 0.94 (-0.35,2.25) |  | 971.78 (661.90-1342.76) | 924.95 (658.35-1265.57) | 35.19 (23.97-48.62) | 51.28 (36.50-70.17) | 0.76 (-0.60,2.13) |
| Germany | 802.31 (662.47-958.84) | 2108.57 (1648.42-2596.99) | 2.01 (1.66-2.40) | 4.94 (3.86-6.08) | 3.58 (3.17,4.00) |  | 796.42 (654.62-948.94) | 1801.70 (1390.89-2234.12) | 1.99 (1.64-2.37) | 4.22 (3.26-5.23) | 3.20 (2.84,3.56) |  | 18266.56 (15204.91-21552.57) | 37472.96 (29587.92-45573.28) | 45.70 (38.04-53.92) | 87.79 (69.32-106.76) | 2.79 (2.42,3.16) |
| Ghana | 60.24 (35.89-98.91) | 120.11 (70.54-192.98) | 0.80 (0.48-1.32) | 0.70 (0.41-1.13) | -1.02 (-1.38,-0.66) |  | 62.66 (37.58-103.05) | 125.65 (74.63-207.16) | 0.84 (0.50-1.38) | 0.73 (0.44-1.21) | -1.03 (-1.38,-0.68) |  | 1865.10 (1079.40-3024.94) | 3602.43 (2112.36-5909.89) | 24.92 (14.42-40.41) | 21.04 (12.34-34.51) | -1.17 (-1.53,-0.81) |
| Greece | 44.96 (33.74-57.03) | 141.86 (103.65-186.72) | 0.87 (0.65-1.10) | 2.79 (2.04-3.67) | 3.65 (3.40,3.90) |  | 46.32 (34.56-58.81) | 144.10 (104.37-190.69) | 0.89 (0.67-1.13) | 2.83 (2.05-3.75) | 3.81 (3.51,4.11) |  | 1014.31 (770.16-1298.26) | 2898.52 (2135.15-3843.13) | 19.53 (14.83-24.99) | 56.97 (41.97-75.54) | 3.49 (3.18,3.80) |
| Greenland | 0.38 (0.26-0.52) | 0.86 (0.61-1.17) | 1.38 (0.95-1.88) | 3.06 (2.17-4.18) | 2.83 (2.71,2.96) |  | 0.38 (0.27-0.52) | 0.87 (0.62-1.18) | 1.38 (0.96-1.89) | 3.08 (2.20-4.20) | 2.88 (2.74,3.03) |  | 12.12 (8.29-16.79) | 23.39 (16.63-31.91) | 43.61 (29.83-60.43) | 83.38 (59.28-113.76) | 2.40 (2.28,2.52) |
| Grenada | 0.24 (0.17-0.33) | 0.80 (0.59-1.03) | 0.54 (0.38-0.75) | 1.55 (1.15-2.01) | 2.87 (2.24,3.51) |  | 0.26 (0.18-0.36) | 0.85 (0.63-1.10) | 0.59 (0.41-0.82) | 1.66 (1.23-2.14) | 2.74 (2.07,3.41) |  | 6.05 (4.26-8.28) | 20.74 (15.07-27.02) | 13.91 (9.78-19.04) | 40.43 (29.36-52.65) | 2.96 (2.39,3.53) |
| Guam | 0.15 (0.10-0.22) | 0.80 (0.52-1.20) | 0.22 (0.14-0.32) | 1.00 (0.66-1.50) | 5.72 (5.48,5.96) |  | 0.15 (0.10-0.22) | 0.80 (0.52-1.20) | 0.23 (0.15-0.33) | 1.00 (0.65-1.51) | 5.70 (5.47,5.93) |  | 4.47 (2.95-6.48) | 22.32 (14.44-34.14) | 6.54 (4.31-9.47) | 28.04 (18.14-42.89) | 5.68 (5.41,5.95) |
| Guatemala | 25.91 (18.63-34.30) | 62.62 (44.65-83.90) | 0.62 (0.44-0.82) | 0.79 (0.57-1.06) | 0.89 (-0.80,2.61) |  | 27.46 (19.75-36.36) | 66.93 (47.56-89.87) | 0.66 (0.47-0.87) | 0.85 (0.60-1.14) | 0.42 (-1.55,2.44) |  | 734.50 (535.60-981.67) | 1690.13 (1214.94-2260.71) | 17.52 (12.78-23.42) | 21.44 (15.41-28.68) | 0.19 (-1.70,2.12) |
| Guinea | 57.14 (37.70-87.47) | 81.75 (48.87-134.78) | 1.91 (1.26-2.92) | 1.22 (0.73-2.01) | -1.38 (-1.64,-1.13) |  | 60.06 (39.74-92.90) | 85.26 (50.78-137.15) | 2.00 (1.33-3.10) | 1.27 (0.76-2.04) | -1.36 (-1.63,-1.10) |  | 1661.84 (1089.23-2489.56) | 2443.92 (1430.06-3932.57) | 55.45 (36.34-83.07) | 36.40 (21.30-58.57) | -1.22 (-1.45,-0.99) |
| Guinea-Bissau | 12.11 (4.30-20.51) | 12.59 (7.03-19.53) | 2.41 (0.85-4.07) | 1.22 (0.68-1.89) | -2.56 (-2.69,-2.44) |  | 12.51 (4.42-21.17) | 12.91 (7.24-19.90) | 2.48 (0.88-4.20) | 1.25 (0.70-1.93) | -2.52 (-2.67,-2.37) |  | 373.55 (134.86-633.00) | 403.37 (223.37-637.22) | 74.18 (26.78-125.71) | 39.09 (21.65-61.75) | -2.38 (-2.53,-2.24) |
| Guyana | 1.70 (1.27-2.30) | 2.25 (1.48-3.19) | 0.44 (0.33-0.59) | 0.59 (0.39-0.83) | 0.90 (0.56,1.23) |  | 1.79 (1.33-2.40) | 2.34 (1.52-3.30) | 0.46 (0.34-0.62) | 0.61 (0.40-0.86) | 0.99 (0.64,1.35) |  | 49.30 (36.25-66.63) | 65.98 (43.36-94.21) | 12.65 (9.30-17.09) | 17.26 (11.34-24.64) | 1.07 (0.73,1.40) |
| Haiti | 7.07 (3.67-13.06) | 13.18 (6.25-26.43) | 0.22 (0.11-0.41) | 0.20 (0.10-0.41) | -0.30 (-0.42,-0.18) |  | 7.44 (3.87-13.67) | 13.90 (6.61-28.06) | 0.23 (0.12-0.43) | 0.22 (0.10-0.44) | -0.27 (-0.38,-0.16) |  | 204.90 (106.40-378.18) | 380.05 (182.00-761.05) | 6.42 (3.33-11.85) | 5.91 (2.83-11.83) | -0.28 (-0.41,-0.15) |
| Honduras | 3.75 (2.10-7.37) | 23.94 (15.35-38.02) | 0.16 (0.09-0.31) | 0.47 (0.30-0.75) | 3.75 (3.63,3.87) |  | 3.99 (2.20-7.86) | 25.65 (16.39-41.10) | 0.17 (0.09-0.33) | 0.51 (0.32-0.81) | 3.85 (3.69,4.01) |  | 105.55 (60.78-199.54) | 634.85 (402.66-1022.30) | 4.48 (2.58-8.47) | 12.56 (7.97-20.22) | 3.58 (3.44,3.72) |
| Hungary | 83.72 (64.03-106.03) | 69.77 (51.81-91.83) | 1.61 (1.23-2.04) | 1.45 (1.08-1.91) | -0.83 (-1.52,-0.13) |  | 89.56 (68.46-113.23) | 73.69 (54.51-97.12) | 1.72 (1.32-2.18) | 1.54 (1.14-2.02) | -0.83 (-1.54,-0.12) |  | 2207.55 (1698.98-2820.72) | 1691.93 (1260.90-2247.71) | 42.47 (32.69-54.27) | 35.26 (26.28-46.84) | -1.03 (-1.66,-0.40) |
| Iceland | 1.00 (0.74-1.32) | 4.78 (3.53-6.29) | 0.79 (0.58-1.04) | 2.73 (2.02-3.59) | 3.85 (3.45,4.24) |  | 1.00 (0.73-1.32) | 4.39 (3.22-5.74) | 0.79 (0.58-1.04) | 2.50 (1.84-3.28) | 3.53 (3.10,3.96) |  | 23.43 (17.50-31.08) | 94.14 (70.12-123.21) | 18.45 (13.79-24.48) | 53.74 (40.02-70.33) | 3.25 (2.84,3.67) |
| India | 1111.93 (912.73-1345.83) | 4462.33 (3660.02-5441.24) | 0.26 (0.21-0.32) | 0.63 (0.52-0.77) | 2.97 (2.79,3.14) |  | 1147.91 (940.61-1387.57) | 4652.62 (3823.97-5715.16) | 0.27 (0.22-0.33) | 0.66 (0.54-0.81) | 2.94 (2.75,3.13) |  | 33578.05 (27458.66-40239.47) | 126015.51 (103694.69-154787.33) | 7.87 (6.44-9.43) | 17.82 (14.66-21.89) | 2.67 (2.50,2.85) |
| Indonesia | 281.69 (197.32-440.89) | 829.44 (516.42-1383.95) | 0.30 (0.21-0.48) | 0.59 (0.37-0.99) | 2.19 (2.15,2.23) |  | 287.60 (202.34-451.38) | 843.28 (518.48-1419.81) | 0.31 (0.22-0.49) | 0.60 (0.37-1.02) | 2.16 (2.11,2.20) |  | 8896.02 (6237.32-14077.25) | 24830.25 (15571.56-40984.08) | 9.62 (6.74-15.22) | 17.80 (11.17-29.39) | 2.02 (1.97,2.07) |
| Iran (Islamic Republic of) | 19.47 (14.95-26.78) | 81.75 (64.55-99.10) | 0.07 (0.05-0.09) | 0.19 (0.15-0.23) | 3.15 (2.40,3.90) |  | 20.54 (15.73-28.23) | 86.33 (68.21-103.87) | 0.07 (0.06-0.10) | 0.20 (0.16-0.24) | 3.10 (2.34,3.87) |  | 534.07 (410.33-736.54) | 2017.89 (1601.19-2459.42) | 1.87 (1.44-2.58) | 4.73 (3.75-5.76) | 2.74 (1.95,3.54) |
| Iraq | 14.33 (8.99-22.15) | 51.30 (29.04-78.69) | 0.16 (0.10-0.24) | 0.25 (0.14-0.38) | 1.79 (1.29,2.28) |  | 15.12 (9.42-23.44) | 53.12 (29.98-82.13) | 0.16 (0.10-0.25) | 0.26 (0.15-0.40) | 1.72 (1.21,2.24) |  | 400.13 (248.50-624.53) | 1380.34 (765.11-2159.05) | 4.35 (2.70-6.78) | 6.70 (3.71-10.47) | 1.67 (1.21,2.13) |
| Ireland | 13.23 (9.91-16.94) | 51.67 (38.85-66.87) | 0.73 (0.55-0.94) | 2.09 (1.57-2.71) | 3.24 (3.11,3.38) |  | 13.76 (10.26-17.70) | 48.44 (35.38-63.57) | 0.76 (0.57-0.98) | 1.96 (1.43-2.57) | 2.83 (2.69,2.96) |  | 312.57 (234.31-394.33) | 1042.84 (787.79-1329.10) | 17.35 (13.01-21.89) | 42.21 (31.89-53.79) | 2.65 (2.53,2.77) |
| Israel | 12.11 (8.63-16.49) | 39.02 (27.36-52.71) | 0.49 (0.35-0.66) | 0.81 (0.57-1.10) | 1.72 (1.62,1.83) |  | 12.83 (9.12-17.50) | 38.48 (26.90-52.43) | 0.52 (0.37-0.71) | 0.80 (0.56-1.09) | 1.42 (1.30,1.55) |  | 286.80 (205.09-388.43) | 851.16 (606.83-1141.67) | 11.56 (8.27-15.66) | 17.74 (12.65-23.80) | 1.44 (1.29,1.59) |
| Italy | 868.91 (763.83-989.23) | 901.37 (780.04-1051.72) | 3.06 (2.69-3.48) | 3.01 (2.61-3.52) | -0.22 (-0.49,0.06) |  | 840.42 (740.83-956.64) | 783.09 (673.69-912.06) | 2.96 (2.61-3.37) | 2.62 (2.25-3.05) | -0.70 (-1.06,-0.34) |  | 20230.30 (17790.69-22954.90) | 16399.45 (14149.98-19114.20) | 71.24 (62.64-80.83) | 54.84 (47.32-63.91) | -1.17 (-1.53,-0.82) |
| Jamaica | 2.63 (1.86-3.62) | 6.93 (4.57-9.83) | 0.22 (0.16-0.31) | 0.49 (0.33-0.70) | 2.60 (2.48,2.72) |  | 2.85 (2.00-3.90) | 7.37 (4.85-10.46) | 0.24 (0.17-0.33) | 0.53 (0.35-0.75) | 2.61 (2.18,3.04) |  | 65.85 (46.72-90.21) | 180.76 (118.38-256.20) | 5.57 (3.95-7.63) | 12.91 (8.46-18.30) | 2.78 (2.32,3.25) |
| Japan | 1637.41 (1413.34-1940.90) | 2078.52 (1741.59-2511.51) | 2.60 (2.25-3.09) | 3.26 (2.73-3.93) | -0.10 (-0.60,0.40) |  | 1333.35 (1150.57-1579.52) | 1581.05 (1329.85-1896.98) | 2.12 (1.83-2.51) | 2.48 (2.08-2.97) | -0.19 (-0.74,0.37) |  | 36128.26 (31032.74-42883.71) | 28758.38 (24342.12-34578.88) | 57.42 (49.33-68.16) | 45.04 (38.12-54.16) | -1.51 (-2.06,-0.96) |
| Jordan | 1.15 (0.62-2.00) | 4.86 (2.86-7.70) | 0.06 (0.03-0.11) | 0.08 (0.05-0.13) | 0.32 (0.15,0.49) |  | 1.19 (0.65-2.08) | 4.94 (2.90-7.93) | 0.06 (0.03-0.11) | 0.08 (0.05-0.13) | 0.29 (0.10,0.47) |  | 32.70 (17.40-55.49) | 130.20 (74.26-204.13) | 1.75 (0.93-2.97) | 2.11 (1.21-3.31) | 0.09 (-0.11,0.28) |
| Kazakhstan | 200.15 (141.10-264.66) | 118.07 (80.23-161.42) | 2.44 (1.72-3.23) | 1.25 (0.85-1.70) | -3.30 (-3.70,-2.90) |  | 206.36 (145.71-273.59) | 122.78 (83.59-167.16) | 2.52 (1.78-3.34) | 1.30 (0.88-1.76) | -3.36 (-3.77,-2.95) |  | 5984.70 (4248.12-7973.56) | 3316.94 (2235.76-4571.78) | 73.02 (51.83-97.28) | 35.00 (23.59-48.24) | -3.61 (-4.02,-3.20) |
| Kenya | 24.30 (16.42-38.79) | 107.83 (81.23-149.63) | 0.21 (0.14-0.34) | 0.43 (0.32-0.60) | 2.25 (2.15,2.35) |  | 25.26 (17.04-40.34) | 110.80 (83.29-154.23) | 0.22 (0.15-0.35) | 0.44 (0.33-0.62) | 2.19 (2.09,2.29) |  | 728.41 (494.43-1172.15) | 3302.51 (2474.89-4589.01) | 6.29 (4.27-10.13) | 13.19 (9.89-18.33) | 2.31 (2.17,2.45) |
| Kiribati | 0.11 (0.07-0.18) | 0.20 (0.12-0.34) | 0.31 (0.19-0.48) | 0.34 (0.20-0.56) | -0.03 (-0.25,0.19) |  | 0.12 (0.07-0.18) | 0.21 (0.12-0.34) | 0.32 (0.19-0.49) | 0.34 (0.21-0.55) | -0.02 (-0.23,0.20) |  | 3.59 (2.24-5.72) | 6.43 (3.76-10.67) | 9.64 (6.03-15.38) | 10.61 (6.20-17.61) | 0.06 (-0.17,0.29) |
| Kuwait | 1.09 (0.72-1.60) | 1.34 (0.87-2.00) | 0.13 (0.08-0.19) | 0.06 (0.04-0.09) | -2.53 (-2.94,-2.11) |  | 1.09 (0.73-1.61) | 1.31 (0.85-1.96) | 0.13 (0.08-0.19) | 0.06 (0.04-0.08) | -2.69 (-3.63,-1.73) |  | 32.11 (21.21-46.87) | 33.93 (21.80-51.66) | 3.74 (2.47-5.45) | 1.46 (0.94-2.22) | -3.01 (-3.98,-2.03) |
| Kyrgyzstan | 19.99 (13.42-28.15) | 20.32 (13.60-28.55) | 0.90 (0.60-1.26) | 0.59 (0.40-0.83) | -1.10 (-1.95,-0.23) |  | 20.68 (13.97-28.95) | 21.05 (14.17-29.41) | 0.93 (0.63-1.30) | 0.61 (0.41-0.86) | -1.46 (-2.40,-0.52) |  | 599.95 (401.96-851.58) | 581.27 (384.66-812.96) | 26.88 (18.01-38.15) | 16.94 (11.21-23.69) | -1.58 (-2.53,-0.62) |
| Lao People's Democratic Republic | 22.74 (13.34-35.74) | 41.39 (24.58-61.60) | 1.09 (0.64-1.71) | 1.12 (0.67-1.67) | -0.12 (-0.43,0.19) |  | 23.57 (14.05-37.72) | 42.75 (25.18-64.05) | 1.13 (0.67-1.81) | 1.16 (0.68-1.74) | -0.14 (-0.45,0.17) |  | 687.52 (402.39-1092.29) | 1233.50 (705.98-1884.49) | 32.98 (19.30-52.39) | 33.44 (19.14-51.09) | -0.16 (-0.46,0.15) |
| Latvia | 15.15 (11.04-19.57) | 25.45 (19.49-31.89) | 1.14 (0.83-1.47) | 2.72 (2.08-3.41) | 2.13 (1.82,2.44) |  | 15.80 (11.59-20.41) | 26.88 (20.43-33.82) | 1.19 (0.87-1.54) | 2.87 (2.18-3.62) | 2.48 (2.11,2.84) |  | 421.50 (306.16-545.31) | 621.32 (476.38-791.59) | 31.71 (23.04-41.03) | 66.44 (50.94-84.65) | 1.99 (1.62,2.36) |
| Lebanon | 3.01 (1.82-4.84) | 5.74 (3.53-8.62) | 0.20 (0.12-0.32) | 0.21 (0.13-0.31) | 0.25 (0.02,0.49) |  | 3.15 (1.93-4.99) | 5.88 (3.68-8.93) | 0.21 (0.13-0.33) | 0.21 (0.13-0.32) | 0.14 (-0.10,0.38) |  | 82.97 (48.98-131.80) | 141.94 (87.16-214.14) | 5.55 (3.27-8.81) | 5.12 (3.15-7.73) | -0.18 (-0.39,0.04) |
| Lesotho | 4.38 (1.34-9.58) | 17.23 (5.84-43.75) | 0.57 (0.17-1.25) | 1.84 (0.62-4.67) | 3.35 (2.52,4.20) |  | 4.60 (1.43-9.99) | 17.76 (5.93-44.90) | 0.60 (0.19-1.30) | 1.90 (0.63-4.79) | 3.35 (2.51,4.19) |  | 125.66 (37.48-266.35) | 537.94 (174.78-1408.30) | 16.40 (4.89-34.76) | 57.40 (18.65-150.27) | 3.77 (2.90,4.64) |
| Liberia | 27.33 (13.12-45.28) | 35.93 (21.16-57.57) | 2.22 (1.07-3.68) | 1.32 (0.78-2.11) | -2.57 (-3.00,-2.14) |  | 28.89 (13.71-47.44) | 37.01 (21.84-58.67) | 2.35 (1.11-3.86) | 1.36 (0.80-2.15) | -2.70 (-3.15,-2.25) |  | 781.19 (373.42-1307.28) | 1123.84 (654.02-1868.97) | 63.50 (30.35-106.26) | 41.17 (23.96-68.47) | -2.24 (-2.72,-1.75) |
| Libya | 3.65 (2.06-5.93) | 14.93 (9.33-24.08) | 0.17 (0.10-0.28) | 0.43 (0.27-0.70) | 3.21 (3.03,3.40) |  | 3.83 (2.13-6.25) | 15.26 (9.61-24.54) | 0.18 (0.10-0.30) | 0.44 (0.28-0.71) | 3.08 (2.87,3.29) |  | 103.02 (57.22-168.52) | 422.75 (262.08-677.73) | 4.89 (2.71-8.00) | 12.31 (7.63-19.73) | 3.21 (2.99,3.43) |
| Lithuania | 15.79 (11.63-20.23) | 38.42 (29.69-48.35) | 0.86 (0.63-1.10) | 2.82 (2.18-3.54) | 3.69 (3.47,3.91) |  | 15.94 (11.83-20.41) | 37.48 (28.60-47.33) | 0.87 (0.64-1.11) | 2.75 (2.10-3.47) | 3.83 (3.61,4.06) |  | 413.57 (304.54-536.69) | 898.02 (685.04-1143.50) | 22.51 (16.58-29.21) | 65.83 (50.22-83.82) | 3.48 (3.24,3.73) |
| Luxembourg | 3.77 (3.00-4.55) | 8.78 (6.66-10.98) | 1.98 (1.57-2.39) | 2.73 (2.07-3.41) | 1.27 (1.03,1.52) |  | 3.91 (3.12-4.73) | 8.40 (6.36-10.64) | 2.05 (1.64-2.48) | 2.61 (1.98-3.30) | 0.96 (0.67,1.25) |  | 91.52 (73.93-109.40) | 183.28 (138.95-229.07) | 48.02 (38.79-57.40) | 56.90 (43.13-71.11) | 0.68 (0.40,0.95) |
| Madagascar | 15.56 (8.12-27.03) | 27.03 (14.00-48.84) | 0.26 (0.14-0.45) | 0.19 (0.10-0.34) | -1.33 (-1.59,-1.06) |  | 16.15 (8.43-28.49) | 27.74 (14.44-50.58) | 0.27 (0.14-0.48) | 0.19 (0.10-0.35) | -1.36 (-1.63,-1.09) |  | 472.89 (248.96-830.05) | 850.52 (430.02-1537.02) | 7.95 (4.18-13.95) | 5.96 (3.01-10.76) | -1.15 (-1.40,-0.90) |
| Malawi | 13.57 (7.70-22.12) | 38.50 (25.08-56.87) | 0.28 (0.16-0.45) | 0.40 (0.26-0.58) | 0.04 (-0.57,0.64) |  | 14.08 (8.03-22.93) | 39.60 (25.62-59.36) | 0.29 (0.16-0.47) | 0.41 (0.26-0.61) | 0.05 (-0.57,0.68) |  | 409.82 (233.16-672.05) | 1189.21 (768.04-1764.87) | 8.36 (4.76-13.71) | 12.23 (7.90-18.15) | 0.17 (-0.45,0.79) |
| Malaysia | 26.22 (17.08-39.03) | 112.85 (70.75-176.52) | 0.30 (0.19-0.44) | 0.71 (0.44-1.11) | 3.00 (2.83,3.18) |  | 27.57 (17.89-41.26) | 114.98 (72.73-181.27) | 0.31 (0.20-0.47) | 0.72 (0.46-1.14) | 2.95 (2.75,3.14) |  | 742.17 (481.57-1090.95) | 3030.32 (1879.53-4755.36) | 8.40 (5.45-12.35) | 19.05 (11.82-29.90) | 2.88 (2.67,3.09) |
| Maldives | 0.58 (0.35-0.86) | 1.49 (0.92-2.13) | 0.52 (0.32-0.78) | 0.58 (0.35-0.82) | 0.14 (-0.08,0.35) |  | 0.61 (0.37-0.91) | 1.53 (0.94-2.22) | 0.54 (0.34-0.82) | 0.59 (0.36-0.86) | 0.10 (-0.12,0.33) |  | 16.77 (10.15-25.42) | 38.68 (23.38-57.47) | 15.09 (9.13-22.88) | 14.96 (9.04-22.22) | -0.37 (-0.49,-0.26) |
| Mali | 79.05 (49.27-117.50) | 178.42 (114.45-271.33) | 1.83 (1.14-2.71) | 1.48 (0.95-2.25) | -0.59 (-0.75,-0.42) |  | 82.06 (51.88-120.60) | 185.40 (118.63-288.43) | 1.89 (1.20-2.78) | 1.54 (0.98-2.39) | -0.58 (-0.74,-0.41) |  | 2364.93 (1502.09-3519.90) | 5369.80 (3380.50-8216.33) | 54.60 (34.68-81.26) | 44.55 (28.05-68.17) | -0.55 (-0.73,-0.37) |
| Malta | 1.29 (0.94-1.70) | 5.31 (3.85-7.08) | 0.70 (0.51-0.92) | 2.40 (1.74-3.20) | 4.42 (4.20,4.64) |  | 1.34 (0.97-1.79) | 5.19 (3.72-6.97) | 0.72 (0.52-0.96) | 2.35 (1.68-3.15) | 4.24 (4.03,4.45) |  | 31.21 (23.05-40.95) | 110.21 (80.25-146.20) | 16.85 (12.44-22.10) | 49.84 (36.29-66.12) | 3.98 (3.77,4.19) |
| Marshall Islands | 0.03 (0.02-0.06) | 0.08 (0.04-0.16) | 0.13 (0.07-0.25) | 0.30 (0.15-0.59) | 3.03 (2.89,3.17) |  | 0.03 (0.02-0.06) | 0.09 (0.04-0.17) | 0.13 (0.07-0.26) | 0.31 (0.16-0.59) | 2.93 (2.77,3.09) |  | 0.89 (0.46-1.70) | 2.65 (1.31-5.23) | 3.91 (2.01-7.49) | 9.42 (4.64-18.59) | 3.07 (2.93,3.21) |
| Mauritania | 26.88 (5.50-56.27) | 28.94 (13.42-48.42) | 2.62 (0.54-5.48) | 1.32 (0.61-2.20) | -2.67 (-2.93,-2.40) |  | 28.24 (5.75-58.86) | 30.63 (14.48-51.51) | 2.75 (0.56-5.73) | 1.39 (0.66-2.34) | -2.66 (-2.91,-2.40) |  | 784.65 (160.76-1603.93) | 805.58 (364.26-1357.22) | 76.38 (15.65-156.13) | 36.65 (16.57-61.74) | -2.83 (-3.09,-2.58) |
| Mauritius | 3.04 (2.13-4.14) | 1.47 (1.03-2.02) | 0.55 (0.39-0.76) | 0.23 (0.16-0.32) | 1.67 (-0.15,3.53) |  | 3.16 (2.23-4.29) | 1.49 (1.04-2.04) | 0.58 (0.41-0.78) | 0.23 (0.16-0.32) | 3.21 (1.28,5.17) |  | 85.81 (60.19-117.54) | 38.82 (27.23-53.45) | 15.66 (10.98-21.45) | 6.10 (4.28-8.40) | 3.13 (1.19,5.10) |
| Mexico | 88.66 (77.43-101.76) | 475.78 (386.70-569.74) | 0.21 (0.18-0.24) | 0.74 (0.60-0.88) | 4.20 (4.03,4.38) |  | 94.27 (82.16-108.42) | 502.72 (409.50-603.11) | 0.22 (0.19-0.25) | 0.78 (0.63-0.93) | 4.18 (3.99,4.37) |  | 2466.70 (2150.67-2831.43) | 12614.40 (10294.16-15257.44) | 5.78 (5.04-6.63) | 19.52 (15.93-23.60) | 4.05 (3.86,4.23) |
| Micronesia (Federated States of) | 0.15 (0.08-0.25) | 0.23 (0.13-0.45) | 0.29 (0.16-0.49) | 0.46 (0.25-0.88) | 1.39 (1.32,1.46) |  | 0.15 (0.08-0.27) | 0.24 (0.13-0.46) | 0.30 (0.16-0.51) | 0.46 (0.25-0.90) | 1.33 (1.26,1.40) |  | 4.52 (2.50-7.66) | 7.23 (3.83-13.95) | 8.74 (4.83-14.80) | 14.10 (7.47-27.20) | 1.53 (1.44,1.63) |
| Monaco | 0.41 (0.25-0.58) | 1.32 (0.84-2.04) | 2.67 (1.67-3.85) | 6.95 (4.45-10.76) | 3.35 (2.82,3.88) |  | 0.41 (0.26-0.59) | 1.27 (0.81-1.95) | 2.72 (1.70-3.90) | 6.69 (4.26-10.29) | 3.15 (2.62,3.69) |  | 8.97 (5.70-12.92) | 26.88 (17.05-40.93) | 58.97 (37.45-84.94) | 142.00 (90.04-216.18) | 3.12 (2.60,3.65) |
| Mongolia | 55.54 (33.48-90.28) | 197.55 (132.92-297.62) | 5.15 (3.10-8.37) | 11.84 (7.97-17.84) | 3.22 (2.92,3.52) |  | 58.09 (34.82-93.38) | 204.96 (138.95-308.05) | 5.38 (3.23-8.66) | 12.29 (8.33-18.47) | 3.17 (2.88,3.45) |  | 1625.85 (991.71-2655.53) | 5870.81 (3892.05-9197.81) | 150.69 (91.92-246.13) | 351.94 (233.32-551.38) | 3.21 (2.94,3.48) |
| Montenegro | 5.99 (4.06-8.60) | 11.99 (7.81-16.96) | 1.91 (1.30-2.75) | 3.88 (2.53-5.49) | 2.45 (2.31,2.59) |  | 6.28 (4.21-8.98) | 12.62 (8.30-17.96) | 2.00 (1.35-2.87) | 4.08 (2.69-5.81) | 2.52 (2.35,2.68) |  | 159.80 (109.23-231.19) | 294.42 (189.28-416.37) | 51.04 (34.89-73.84) | 95.27 (61.25-134.73) | 2.15 (2.00,2.30) |
| Morocco | 2.77 (1.66-4.48) | 8.86 (5.27-14.53) | 0.02 (0.01-0.04) | 0.05 (0.03-0.08) | 2.47 (2.23,2.72) |  | 2.92 (1.76-4.71) | 9.25 (5.43-15.32) | 0.02 (0.01-0.04) | 0.05 (0.03-0.08) | 2.44 (2.20,2.68) |  | 77.28 (45.60-122.44) | 241.85 (144.73-391.54) | 0.61 (0.36-0.97) | 1.30 (0.78-2.11) | 2.37 (2.10,2.65) |
| Mozambique | 93.51 (50.55-160.37) | 236.23 (113.49-524.82) | 1.40 (0.76-2.40) | 1.52 (0.73-3.38) | 0.32 (0.09,0.55) |  | 99.21 (53.52-168.52) | 248.31 (117.91-550.25) | 1.49 (0.80-2.52) | 1.60 (0.76-3.54) | 0.30 (0.07,0.52) |  | 2611.23 (1383.16-4428.32) | 6813.22 (3221.17-14765.55) | 39.09 (20.71-66.29) | 43.85 (20.73-95.04) | 0.50 (0.28,0.73) |
| Myanmar | 53.68 (24.69-116.53) | 130.88 (63.01-286.84) | 0.27 (0.12-0.58) | 0.46 (0.22-1.02) | 1.73 (1.51,1.95) |  | 55.51 (25.49-118.88) | 135.32 (64.63-290.99) | 0.27 (0.13-0.59) | 0.48 (0.23-1.03) | 1.72 (1.50,1.94) |  | 1620.56 (749.89-3461.24) | 3769.41 (1795.62-8660.75) | 8.01 (3.71-17.12) | 13.36 (6.37-30.70) | 1.60 (1.38,1.83) |
| Namibia | 1.15 (0.52-2.30) | 4.89 (3.16-7.19) | 0.16 (0.07-0.33) | 0.40 (0.26-0.59) | 2.54 (2.04,3.05) |  | 1.20 (0.54-2.42) | 5.06 (3.27-7.36) | 0.17 (0.08-0.35) | 0.42 (0.27-0.61) | 2.54 (2.02,3.05) |  | 33.07 (14.86-65.06) | 145.82 (93.97-217.03) | 4.71 (2.12-9.27) | 12.00 (7.73-17.85) | 2.69 (2.14,3.25) |
| Nauru | 0.02 (0.01-0.03) | 0.02 (0.01-0.04) | 0.41 (0.25-0.63) | 0.45 (0.25-0.76) | -0.35 (-0.57,-0.12) |  | 0.02 (0.01-0.03) | 0.02 (0.01-0.04) | 0.41 (0.25-0.63) | 0.44 (0.25-0.76) | -0.40 (-0.65,-0.14) |  | 0.69 (0.41-1.11) | 0.83 (0.45-1.47) | 13.61 (8.09-21.69) | 15.10 (8.22-26.61) | -0.25 (-0.48,-0.01) |
| Nepal | 17.31 (10.69-26.65) | 93.05 (53.84-146.59) | 0.18 (0.11-0.27) | 0.60 (0.35-0.94) | 4.55 (4.14,4.96) |  | 17.90 (10.95-27.97) | 97.54 (55.82-154.76) | 0.18 (0.11-0.29) | 0.63 (0.36-0.99) | 4.63 (4.20,5.05) |  | 528.34 (323.99-818.73) | 2633.84 (1511.29-4092.05) | 5.43 (3.33-8.41) | 16.92 (9.71-26.29) | 4.32 (3.90,4.74) |
| Netherlands | 47.90 (37.42-57.83) | 179.28 (141.33-224.07) | 0.64 (0.50-0.78) | 2.08 (1.64-2.60) | 4.01 (3.77,4.25) |  | 51.51 (40.19-62.07) | 192.91 (150.65-241.47) | 0.69 (0.54-0.83) | 2.24 (1.75-2.81) | 3.96 (3.74,4.17) |  | 1223.10 (963.21-1479.71) | 4106.09 (3252.78-5192.20) | 16.39 (12.91-19.83) | 47.72 (37.80-60.34) | 3.64 (3.45,3.82) |
| New Zealand | 19.41 (16.70-22.09) | 81.76 (69.88-94.64) | 1.14 (0.98-1.29) | 3.16 (2.70-3.66) | 3.34 (3.15,3.52) |  | 16.67 (14.44-18.97) | 60.39 (51.65-69.61) | 0.98 (0.85-1.11) | 2.34 (2.00-2.69) | 2.94 (2.74,3.13) |  | 441.49 (381.01-500.53) | 1494.65 (1277.75-1723.03) | 25.84 (22.30-29.29) | 57.83 (49.43-66.66) | 2.73 (2.55,2.92) |
| Nicaragua | 4.95 (3.35-7.23) | 18.67 (12.28-27.13) | 0.25 (0.17-0.37) | 0.56 (0.37-0.81) | 3.01 (2.61,3.42) |  | 5.24 (3.56-7.64) | 19.57 (12.87-28.50) | 0.27 (0.18-0.39) | 0.59 (0.39-0.85) | 3.03 (2.59,3.47) |  | 139.45 (95.51-203.89) | 511.20 (337.86-743.31) | 7.17 (4.91-10.49) | 15.33 (10.13-22.29) | 2.98 (2.52,3.44) |
| Niger | 42.61 (18.41-77.35) | 57.05 (34.12-101.84) | 1.06 (0.46-1.93) | 0.46 (0.27-0.81) | -2.96 (-3.06,-2.85) |  | 43.94 (19.04-79.94) | 59.68 (35.94-105.88) | 1.09 (0.47-1.99) | 0.48 (0.29-0.85) | -2.85 (-2.97,-2.74) |  | 1316.06 (562.74-2374.51) | 1694.48 (998.23-2991.63) | 32.77 (14.01-59.12) | 13.54 (7.97-23.90) | -3.01 (-3.12,-2.90) |
| Nigeria | 193.28 (84.82-361.59) | 370.13 (246.33-529.52) | 0.43 (0.19-0.80) | 0.32 (0.21-0.46) | -1.44 (-1.61,-1.27) |  | 203.11 (89.26-381.27) | 388.81 (263.19-554.16) | 0.45 (0.20-0.85) | 0.34 (0.23-0.48) | -1.40 (-1.58,-1.23) |  | 5618.21 (2368.87-10349.47) | 10687.13 (6998.36-15708.71) | 12.48 (5.26-22.99) | 9.25 (6.05-13.59) | -1.44 (-1.62,-1.26) |
| Niue | 0.01 (0.00-0.01) | 0.01 (0.00-0.01) | 0.49 (0.29-0.79) | 0.97 (0.56-1.72) | 1.87 (1.49,2.25) |  | 0.01 (0.00-0.01) | 0.01 (0.00-0.01) | 0.52 (0.31-0.84) | 1.00 (0.57-1.77) | 1.80 (1.42,2.19) |  | 0.16 (0.09-0.25) | 0.22 (0.13-0.41) | 13.57 (7.82-22.07) | 26.79 (15.44-48.51) | 1.81 (1.45,2.17) |
| North Macedonia | 33.18 (23.37-44.45) | 50.75 (33.95-68.75) | 3.33 (2.35-4.46) | 4.66 (3.12-6.32) | 0.68 (0.42,0.95) |  | 35.37 (24.88-46.87) | 53.99 (36.39-73.43) | 3.55 (2.50-4.71) | 4.96 (3.34-6.75) | 0.65 (0.38,0.91) |  | 892.73 (628.15-1205.19) | 1273.97 (853.28-1773.30) | 89.62 (63.06-120.98) | 117.07 (78.41-162.95) | 0.38 (0.10,0.66) |
| Northern Mariana Islands | 0.05 (0.03-0.08) | 0.23 (0.14-0.38) | 0.23 (0.14-0.35) | 0.96 (0.60-1.55) | 5.38 (4.78,5.98) |  | 0.05 (0.03-0.08) | 0.23 (0.14-0.38) | 0.23 (0.14-0.34) | 0.95 (0.59-1.56) | 5.47 (4.85,6.09) |  | 1.70 (1.00-2.60) | 6.53 (4.03-10.66) | 7.52 (4.45-11.54) | 26.94 (16.64-43.97) | 4.98 (4.43,5.53) |
| Norway | 15.80 (14.05-18.07) | 63.14 (55.70-71.56) | 0.74 (0.66-0.85) | 2.33 (2.06-2.64) | 3.66 (3.31,4.02) |  | 15.56 (13.82-17.92) | 55.46 (49.05-63.05) | 0.73 (0.65-0.84) | 2.05 (1.81-2.33) | 3.31 (2.84,3.78) |  | 357.48 (318.51-407.91) | 1230.60 (1085.22-1391.77) | 16.84 (15.00-19.21) | 45.43 (40.06-51.37) | 3.31 (2.88,3.75) |
| Oman | 0.97 (0.55-1.76) | 3.63 (2.27-5.46) | 0.10 (0.06-0.18) | 0.15 (0.10-0.23) | 1.70 (1.45,1.94) |  | 1.00 (0.56-1.80) | 3.56 (2.28-5.31) | 0.10 (0.06-0.18) | 0.15 (0.10-0.23) | 1.63 (1.33,1.92) |  | 28.45 (15.93-51.37) | 103.30 (64.83-158.22) | 2.87 (1.61-5.18) | 4.39 (2.76-6.73) | 1.67 (1.37,1.98) |
| Pakistan | 131.21 (93.40-177.79) | 358.66 (255.26-489.80) | 0.24 (0.17-0.32) | 0.30 (0.22-0.42) | 0.65 (0.54,0.76) |  | 136.52 (96.98-183.27) | 366.58 (259.76-498.18) | 0.25 (0.17-0.33) | 0.31 (0.22-0.42) | 0.61 (0.50,0.72) |  | 3974.32 (2831.01-5404.05) | 11314.33 (8091.30-15629.82) | 7.15 (5.10-9.73) | 9.61 (6.87-13.27) | 0.79 (0.64,0.94) |
| Palau | 0.05 (0.03-0.08) | 0.14 (0.08-0.22) | 0.66 (0.39-1.10) | 1.53 (0.89-2.38) | 2.69 (2.57,2.81) |  | 0.05 (0.03-0.09) | 0.14 (0.08-0.22) | 0.68 (0.40-1.14) | 1.53 (0.89-2.41) | 2.60 (2.50,2.71) |  | 1.50 (0.86-2.54) | 4.22 (2.50-6.67) | 19.70 (11.33-33.48) | 46.62 (27.61-73.73) | 2.80 (2.61,2.98) |
| Palestine | 2.75 (1.62-4.46) | 6.92 (4.32-10.82) | 0.27 (0.16-0.44) | 0.27 (0.17-0.42) | -0.26 (-0.37,-0.15) |  | 2.97 (1.74-4.78) | 7.17 (4.52-11.19) | 0.29 (0.17-0.47) | 0.28 (0.18-0.44) | -0.45 (-0.59,-0.31) |  | 71.29 (42.36-115.26) | 184.83 (114.35-285.06) | 6.97 (4.14-11.26) | 7.20 (4.45-11.10) | -0.23 (-0.38,-0.08) |
| Panama | 7.44 (5.56-9.59) | 20.68 (14.21-27.82) | 0.62 (0.47-0.80) | 0.96 (0.66-1.30) | 1.88 (1.05,2.72) |  | 8.01 (5.97-10.40) | 22.08 (15.10-29.92) | 0.67 (0.50-0.87) | 1.03 (0.70-1.39) | 1.81 (1.00,2.63) |  | 192.72 (146.53-244.71) | 514.31 (356.16-699.04) | 16.13 (12.27-20.49) | 23.97 (16.60-32.57) | 1.72 (0.92,2.53) |
| Papua New Guinea | 4.02 (1.77-11.59) | 7.73 (3.06-22.05) | 0.20 (0.09-0.56) | 0.15 (0.06-0.42) | -0.97 (-1.10,-0.84) |  | 4.13 (1.82-11.75) | 7.93 (3.07-22.88) | 0.20 (0.09-0.57) | 0.15 (0.06-0.44) | -1.02 (-1.15,-0.89) |  | 123.18 (53.85-347.07) | 238.64 (92.93-692.94) | 6.00 (2.62-16.92) | 4.56 (1.78-13.25) | -0.93 (-1.07,-0.80) |
| Paraguay | 8.14 (5.98-10.92) | 30.30 (19.94-43.84) | 0.40 (0.30-0.54) | 0.85 (0.56-1.22) | 3.06 (2.63,3.48) |  | 8.67 (6.34-11.61) | 31.87 (21.13-46.34) | 0.43 (0.31-0.57) | 0.89 (0.59-1.29) | 3.06 (2.61,3.51) |  | 223.96 (163.30-297.99) | 823.66 (543.36-1202.56) | 11.08 (8.08-14.74) | 22.98 (15.16-33.55) | 3.02 (2.59,3.46) |
| Peru | 29.01 (19.20-39.87) | 116.31 (75.49-172.29) | 0.27 (0.18-0.37) | 0.64 (0.42-0.95) | 2.85 (2.63,3.08) |  | 31.08 (20.28-43.00) | 124.60 (79.58-187.44) | 0.29 (0.19-0.40) | 0.69 (0.44-1.03) | 2.94 (2.69,3.19) |  | 799.89 (536.67-1121.53) | 2900.84 (1871.97-4264.31) | 7.39 (4.96-10.37) | 16.00 (10.32-23.51) | 2.54 (2.29,2.80) |
| Philippines | 227.67 (151.35-313.06) | 647.23 (490.72-826.43) | 0.72 (0.48-0.99) | 1.14 (0.87-1.46) | 1.47 (1.38,1.56) |  | 232.80 (153.94-319.84) | 661.91 (504.39-841.19) | 0.74 (0.49-1.02) | 1.17 (0.89-1.49) | 1.45 (1.35,1.55) |  | 7246.09 (4844.00-9846.82) | 19190.76 (14500.13-24692.79) | 23.00 (15.38-31.26) | 33.89 (25.61-43.61) | 1.19 (1.10,1.28) |
| Poland | 41.39 (36.08-47.00) | 267.31 (233.34-299.60) | 0.22 (0.19-0.25) | 1.40 (1.22-1.57) | 7.40 (6.64,8.16) |  | 44.94 (39.35-50.87) | 288.02 (251.71-323.45) | 0.24 (0.21-0.27) | 1.51 (1.32-1.69) | 7.43 (6.66,8.21) |  | 1078.25 (931.47-1231.92) | 6698.59 (5841.85-7555.51) | 5.65 (4.88-6.45) | 35.04 (30.55-39.52) | 7.38 (6.60,8.18) |
| Portugal | 54.12 (42.28-66.90) | 227.39 (177.58-283.15) | 1.07 (0.83-1.32) | 4.29 (3.35-5.34) | 4.10 (3.87,4.33) |  | 57.53 (44.58-71.63) | 233.47 (181.61-288.76) | 1.14 (0.88-1.41) | 4.40 (3.42-5.44) | 4.10 (3.84,4.36) |  | 1400.70 (1102.47-1721.40) | 5299.98 (4126.16-6597.62) | 27.64 (21.75-33.96) | 99.93 (77.79-124.39) | 3.91 (3.67,4.15) |
| Puerto Rico | 15.10 (11.05-20.07) | 32.27 (22.80-42.74) | 0.84 (0.61-1.11) | 1.96 (1.38-2.59) | 2.10 (1.58,2.62) |  | 16.01 (11.69-21.35) | 33.53 (23.59-44.71) | 0.89 (0.65-1.18) | 2.04 (1.43-2.71) | 2.24 (1.80,2.68) |  | 392.63 (288.66-516.54) | 758.29 (530.85-1018.11) | 21.74 (15.98-28.60) | 46.04 (32.23-61.81) | 1.95 (1.53,2.37) |
| Qatar | 0.50 (0.30-0.81) | 5.61 (3.24-8.89) | 0.23 (0.14-0.36) | 0.38 (0.22-0.60) | 0.33 (-0.40,1.07) |  | 0.51 (0.31-0.82) | 5.33 (3.06-8.50) | 0.23 (0.14-0.37) | 0.36 (0.21-0.57) | -0.16 (-0.90,0.58) |  | 15.22 (9.20-24.35) | 152.35 (88.13-250.37) | 6.84 (4.13-10.95) | 10.23 (5.92-16.82) | -0.11 (-0.80,0.59) |
| Republic of Korea | 694.82 (430.65-1000.09) | 1573.14 (1030.58-2329.46) | 3.14 (1.95-4.52) | 6.10 (4.00-9.03) | 2.13 (2.03,2.24) |  | 685.57 (431.25-990.01) | 1167.09 (769.23-1702.77) | 3.10 (1.95-4.48) | 4.53 (2.98-6.60) | 1.05 (0.92,1.18) |  | 19596.40 (12126.04-29261.07) | 27121.36 (17674.87-40595.31) | 88.58 (54.81-132.27) | 105.18 (68.55-157.44) | 0.30 (0.18,0.42) |
| Republic of Moldova | 25.68 (20.01-31.53) | 32.17 (25.27-40.02) | 1.15 (0.90-1.42) | 1.79 (1.41-2.23) | 0.45 (-0.36,1.27) |  | 26.81 (21.01-32.90) | 33.82 (26.50-42.14) | 1.21 (0.94-1.48) | 1.88 (1.48-2.35) | 0.39 (-0.56,1.35) |  | 727.86 (569.79-892.30) | 858.84 (673.05-1065.54) | 32.73 (25.62-40.13) | 47.80 (37.46-59.30) | 0.19 (-0.76,1.16) |
| Romania | 70.41 (51.22-88.50) | 232.11 (175.80-288.67) | 0.60 (0.44-0.76) | 2.45 (1.86-3.05) | 5.05 (4.83,5.26) |  | 74.86 (54.97-94.22) | 245.80 (186.40-304.92) | 0.64 (0.47-0.81) | 2.60 (1.97-3.22) | 5.13 (4.85,5.41) |  | 1916.05 (1387.10-2418.11) | 5852.84 (4523.38-7348.93) | 16.39 (11.87-20.69) | 61.81 (47.77-77.61) | 4.87 (4.58,5.15) |
| Russian Federation | 512.54 (452.57-574.68) | 1117.37 (983.77-1278.52) | 0.68 (0.60-0.76) | 1.54 (1.36-1.77) | 2.98 (2.67,3.29) |  | 535.21 (473.23-598.32) | 1172.25 (1037.30-1340.06) | 0.71 (0.63-0.79) | 1.62 (1.43-1.85) | 3.02 (2.64,3.39) |  | 14551.38 (12871.47-16374.91) | 29495.77 (25949.99-33846.23) | 19.28 (17.05-21.69) | 40.72 (35.83-46.73) | 2.80 (2.39,3.21) |
| Rwanda | 23.40 (13.17-37.27) | 30.49 (17.29-48.19) | 0.65 (0.37-1.04) | 0.46 (0.26-0.73) | -2.27 (-2.77,-1.77) |  | 24.21 (13.76-39.12) | 31.66 (17.72-50.09) | 0.67 (0.38-1.09) | 0.48 (0.27-0.75) | -2.10 (-2.64,-1.56) |  | 715.68 (393.12-1142.00) | 918.47 (520.82-1458.22) | 19.91 (10.94-31.77) | 13.84 (7.85-21.98) | -2.19 (-2.73,-1.65) |
| Saint Kitts and Nevis | 0.20 (0.14-0.27) | 0.37 (0.26-0.49) | 0.96 (0.69-1.28) | 1.27 (0.87-1.68) | 0.06 (-0.83,0.96) |  | 0.22 (0.15-0.29) | 0.39 (0.26-0.51) | 1.04 (0.75-1.40) | 1.32 (0.90-1.75) | -0.18 (-1.02,0.67) |  | 5.01 (3.64-6.72) | 10.08 (6.87-13.48) | 24.16 (17.53-32.41) | 34.38 (23.43-45.96) | 0.32 (-0.53,1.19) |
| Saint Lucia | 0.36 (0.27-0.47) | 0.70 (0.50-0.93) | 0.53 (0.40-0.69) | 0.79 (0.56-1.04) | 0.73 (0.26,1.21) |  | 0.39 (0.29-0.50) | 0.74 (0.52-0.97) | 0.57 (0.42-0.74) | 0.83 (0.59-1.09) | 0.61 (0.15,1.07) |  | 9.69 (7.14-12.72) | 18.91 (13.40-25.33) | 14.19 (10.46-18.63) | 21.31 (15.09-28.54) | 0.80 (0.35,1.25) |
| Saint Vincent and the Grenadines | 0.36 (0.27-0.48) | 0.72 (0.54-0.92) | 0.66 (0.49-0.87) | 1.26 (0.94-1.61) | 1.84 (1.23,2.44) |  | 0.39 (0.29-0.51) | 0.76 (0.57-0.97) | 0.71 (0.53-0.94) | 1.34 (1.00-1.70) | 1.71 (1.10,2.33) |  | 9.53 (7.07-12.58) | 19.40 (14.26-25.39) | 17.40 (12.91-22.97) | 34.01 (25.00-44.51) | 1.87 (1.29,2.46) |
| Samoa | 0.29 (0.17-0.45) | 0.44 (0.26-0.69) | 0.34 (0.21-0.54) | 0.41 (0.25-0.64) | 0.18 (-0.06,0.42) |  | 0.30 (0.18-0.47) | 0.45 (0.27-0.71) | 0.36 (0.22-0.56) | 0.42 (0.25-0.67) | 0.13 (-0.13,0.39) |  | 8.14 (4.86-12.76) | 12.40 (7.16-19.88) | 9.64 (5.75-15.10) | 11.61 (6.70-18.61) | 0.23 (0.01,0.46) |
| San Marino | 0.14 (0.10-0.19) | 0.35 (0.20-0.53) | 1.18 (0.84-1.63) | 2.13 (1.21-3.25) | 2.19 (2.07,2.31) |  | 0.14 (0.10-0.19) | 0.34 (0.19-0.52) | 1.18 (0.84-1.64) | 2.05 (1.19-3.15) | 2.08 (1.94,2.22) |  | 3.14 (2.22-4.32) | 7.02 (4.06-10.73) | 26.47 (18.72-36.39) | 42.90 (24.81-65.57) | 1.86 (1.71,2.00) |
| Sao Tome and Principe | 0.15 (0.10-0.23) | 0.26 (0.14-0.47) | 0.25 (0.16-0.37) | 0.24 (0.13-0.43) | -0.62 (-0.82,-0.42) |  | 0.17 (0.11-0.24) | 0.27 (0.15-0.48) | 0.27 (0.18-0.40) | 0.25 (0.14-0.45) | -0.67 (-0.90,-0.44) |  | 4.29 (2.75-6.11) | 7.71 (4.05-13.98) | 7.07 (4.54-10.07) | 7.12 (3.74-12.91) | -0.34 (-0.56,-0.12) |
| Saudi Arabia | 9.91 (5.22-17.39) | 26.72 (16.02-42.60) | 0.12 (0.07-0.22) | 0.14 (0.08-0.23) | -0.52 (-0.84,-0.20) |  | 10.37 (5.51-18.49) | 26.86 (16.17-43.29) | 0.13 (0.07-0.23) | 0.14 (0.09-0.23) | -0.62 (-0.95,-0.28) |  | 282.73 (150.15-496.37) | 733.22 (434.26-1203.66) | 3.57 (1.89-6.26) | 3.89 (2.30-6.38) | -0.66 (-1.02,-0.31) |
| Senegal | 32.24 (18.17-49.64) | 50.60 (31.95-75.49) | 0.84 (0.48-1.30) | 0.64 (0.40-0.95) | -1.14 (-1.33,-0.96) |  | 33.77 (18.95-51.79) | 53.36 (34.12-79.54) | 0.89 (0.50-1.36) | 0.67 (0.43-1.00) | -1.15 (-1.35,-0.95) |  | 951.16 (527.17-1441.51) | 1455.63 (903.58-2207.38) | 24.92 (13.81-37.77) | 18.36 (11.39-27.84) | -1.24 (-1.43,-1.04) |
| Serbia | 84.04 (55.31-118.53) | 132.89 (89.32-188.79) | 1.75 (1.15-2.46) | 2.98 (2.00-4.23) | 1.24 (1.02,1.46) |  | 88.11 (57.68-124.60) | 140.20 (94.02-200.91) | 1.83 (1.20-2.59) | 3.14 (2.11-4.50) | 1.26 (1.01,1.50) |  | 2315.87 (1520.80-3288.78) | 3220.59 (2133.93-4572.68) | 48.10 (31.59-68.31) | 72.21 (47.85-102.53) | 0.79 (0.57,1.02) |
| Seychelles | 0.47 (0.32-0.68) | 0.60 (0.40-0.85) | 1.29 (0.87-1.86) | 1.14 (0.76-1.61) | -0.15 (-0.67,0.37) |  | 0.50 (0.34-0.71) | 0.61 (0.41-0.86) | 1.37 (0.93-1.96) | 1.16 (0.77-1.63) | -0.31 (-0.83,0.22) |  | 13.07 (8.72-19.12) | 16.79 (11.03-24.34) | 35.88 (23.95-52.48) | 31.85 (20.92-46.18) | -0.12 (-0.62,0.39) |
| Sierra Leone | 38.58 (13.04-70.26) | 31.38 (19.07-48.81) | 1.86 (0.63-3.38) | 0.71 (0.43-1.10) | -3.77 (-4.03,-3.50) |  | 40.90 (13.96-75.06) | 32.85 (19.71-50.93) | 1.97 (0.67-3.62) | 0.74 (0.44-1.15) | -3.82 (-4.10,-3.54) |  | 1106.88 (378.88-1966.89) | 939.95 (559.38-1487.09) | 53.32 (18.25-94.75) | 21.20 (12.62-33.54) | -3.57 (-3.83,-3.31) |
| Singapore | 6.20 (4.01-9.21) | 23.12 (14.77-35.54) | 0.41 (0.26-0.60) | 0.81 (0.52-1.24) | 1.89 (1.68,2.10) |  | 5.98 (3.88-8.99) | 17.75 (11.18-27.07) | 0.39 (0.25-0.59) | 0.62 (0.39-0.95) | 1.31 (1.06,1.55) |  | 158.32 (103.10-234.67) | 401.73 (253.74-607.74) | 10.39 (6.77-15.40) | 14.03 (8.86-21.22) | 0.78 (0.52,1.03) |
| Slovakia | 69.54 (51.03-93.98) | 81.13 (55.52-114.65) | 2.63 (1.93-3.56) | 2.99 (2.05-4.22) | -0.01 (-0.24,0.22) |  | 73.37 (53.93-98.81) | 84.50 (58.03-119.70) | 2.78 (2.04-3.74) | 3.11 (2.14-4.41) | -0.10 (-0.35,0.14) |  | 1856.74 (1357.63-2503.10) | 2005.44 (1334.59-2842.13) | 70.29 (51.39-94.76) | 73.87 (49.16-104.69) | -0.26 (-0.50,-0.02) |
| Slovenia | 20.65 (16.00-25.62) | 41.00 (30.26-53.31) | 2.09 (1.62-2.60) | 3.96 (2.92-5.15) | 2.29 (2.08,2.51) |  | 21.66 (16.79-27.00) | 42.25 (30.86-54.77) | 2.20 (1.70-2.74) | 4.08 (2.98-5.29) | 2.06 (1.77,2.34) |  | 550.51 (418.26-686.23) | 908.16 (665.15-1196.74) | 55.79 (42.39-69.54) | 87.76 (64.28-115.65) | 1.45 (1.17,1.72) |
| Solomon Islands | 0.51 (0.16-1.22) | 1.01 (0.58-1.77) | 0.30 (0.09-0.72) | 0.30 (0.17-0.52) | -0.12 (-0.36,0.12) |  | 0.52 (0.16-1.28) | 1.02 (0.59-1.78) | 0.31 (0.09-0.76) | 0.30 (0.17-0.52) | -0.19 (-0.41,0.04) |  | 15.91 (4.94-37.27) | 32.70 (18.07-55.85) | 9.38 (2.92-21.98) | 9.57 (5.29-16.34) | 0.01 (-0.20,0.22) |
| Somalia | 19.01 (6.39-38.67) | 38.57 (15.92-77.81) | 0.48 (0.16-0.97) | 0.36 (0.15-0.72) | -1.43 (-1.62,-1.24) |  | 19.35 (6.50-38.70) | 39.51 (16.22-79.55) | 0.49 (0.16-0.97) | 0.37 (0.15-0.74) | -1.35 (-1.54,-1.16) |  | 610.38 (200.60-1241.63) | 1218.96 (489.18-2425.19) | 15.38 (5.05-31.28) | 11.28 (4.53-22.45) | -1.49 (-1.70,-1.28) |
| South Africa | 105.29 (57.23-182.89) | 338.87 (270.29-418.21) | 0.57 (0.31-0.99) | 1.19 (0.95-1.47) | 1.44 (0.84,2.05) |  | 108.72 (59.40-191.61) | 351.36 (281.04-430.48) | 0.59 (0.32-1.04) | 1.24 (0.99-1.51) | 1.40 (0.75,2.05) |  | 3329.28 (1826.33-5662.82) | 9990.60 (7872.33-12348.64) | 17.99 (9.87-30.60) | 35.14 (27.69-43.44) | 1.17 (0.50,1.84) |
| South Sudan | 11.71 (5.76-19.59) | 20.96 (11.89-34.80) | 0.40 (0.20-0.67) | 0.43 (0.25-0.72) | -0.20 (-0.65,0.25) |  | 12.23 (6.02-20.08) | 21.61 (12.49-35.42) | 0.42 (0.20-0.68) | 0.45 (0.26-0.73) | -0.17 (-0.62,0.29) |  | 345.50 (168.96-570.27) | 637.54 (366.17-1058.30) | 11.76 (5.75-19.41) | 13.18 (7.57-21.88) | 0.03 (-0.42,0.48) |
| Spain | 355.66 (265.50-460.18) | 834.80 (591.05-1125.82) | 1.83 (1.37-2.37) | 3.67 (2.60-4.94) | 1.59 (1.18,2.00) |  | 362.60 (269.46-466.82) | 754.41 (525.67-1014.45) | 1.87 (1.39-2.41) | 3.31 (2.31-4.45) | 1.21 (0.86,1.56) |  | 8730.71 (6581.10-11295.45) | 16727.63 (11807.00-22269.23) | 45.02 (33.94-58.25) | 73.45 (51.84-97.78) | 1.00 (0.70,1.30) |
| Sri Lanka | 19.49 (13.26-28.33) | 47.01 (25.85-73.08) | 0.23 (0.15-0.33) | 0.42 (0.23-0.66) | 1.68 (1.36,2.00) |  | 20.56 (13.99-29.91) | 47.43 (25.87-73.48) | 0.24 (0.16-0.35) | 0.43 (0.23-0.66) | 1.63 (1.29,1.97) |  | 545.86 (373.17-785.76) | 1199.15 (644.94-1898.34) | 6.37 (4.36-9.17) | 10.77 (5.79-17.05) | 1.45 (1.09,1.81) |
| Sudan | 25.08 (11.23-48.77) | 38.66 (21.27-61.52) | 0.25 (0.11-0.49) | 0.18 (0.10-0.28) | -1.22 (-1.72,-0.72) |  | 26.65 (11.87-51.57) | 40.78 (22.00-65.44) | 0.27 (0.12-0.52) | 0.19 (0.10-0.30) | -1.27 (-1.77,-0.78) |  | 698.54 (313.41-1343.62) | 1074.45 (590.67-1740.14) | 6.98 (3.13-13.42) | 4.95 (2.72-8.02) | -1.23 (-1.74,-0.72) |
| Suriname | 0.85 (0.59-1.15) | 2.60 (1.62-3.90) | 0.44 (0.30-0.60) | 0.90 (0.56-1.35) | 2.19 (1.95,2.42) |  | 0.90 (0.62-1.23) | 2.73 (1.71-4.05) | 0.47 (0.32-0.63) | 0.94 (0.59-1.40) | 1.96 (1.65,2.28) |  | 24.06 (16.43-32.62) | 72.47 (45.68-109.87) | 12.44 (8.50-16.87) | 25.02 (15.77-37.94) | 1.93 (1.59,2.26) |
| Sweden | 70.53 (61.69-80.45) | 121.91 (100.66-145.57) | 1.64 (1.44-1.87) | 2.35 (1.94-2.81) | 1.85 (0.87,2.84) |  | 76.96 (67.12-87.87) | 129.45 (107.88-153.13) | 1.79 (1.56-2.05) | 2.50 (2.08-2.95) | 2.11 (1.13,3.10) |  | 1703.15 (1495.73-1944.05) | 2730.27 (2265.64-3257.54) | 39.66 (34.83-45.27) | 52.64 (43.68-62.80) | 2.07 (1.08,3.06) |
| Switzerland | 97.07 (76.15-120.06) | 153.78 (114.24-196.82) | 2.83 (2.22-3.50) | 3.45 (2.56-4.41) | 0.88 (0.72,1.04) |  | 92.93 (72.05-115.23) | 135.60 (99.37-179.26) | 2.71 (2.10-3.36) | 3.04 (2.23-4.02) | 0.68 (0.54,0.82) |  | 2137.95 (1701.08-2651.10) | 2816.93 (2108.07-3610.57) | 62.27 (49.55-77.22) | 63.14 (47.25-80.93) | 0.33 (0.16,0.49) |
| Syrian Arab Republic | 14.12 (8.00-23.45) | 28.21 (16.87-45.93) | 0.22 (0.13-0.37) | 0.40 (0.24-0.65) | 1.44 (0.61,2.27) |  | 14.91 (8.45-24.58) | 29.08 (17.35-47.11) | 0.23 (0.13-0.39) | 0.41 (0.25-0.67) | 1.36 (0.50,2.22) |  | 394.80 (228.10-654.11) | 745.42 (451.62-1222.83) | 6.21 (3.59-10.29) | 10.63 (6.44-17.43) | 1.24 (0.38,2.10) |
| Taiwan (Province of China) | 48.76 (33.35-70.99) | 155.32 (102.22-229.01) | 0.48 (0.33-0.70) | 1.31 (0.86-1.94) | 4.14 (3.42,4.85) |  | 48.11 (32.94-70.12) | 138.52 (92.33-203.55) | 0.47 (0.32-0.69) | 1.17 (0.78-1.72) | 3.87 (3.08,4.66) |  | 1447.03 (987.19-2077.44) | 3457.12 (2283.91-5120.24) | 14.19 (9.68-20.38) | 29.25 (19.33-43.32) | 3.25 (2.47,4.04) |
| Tajikistan | 13.67 (8.15-22.55) | 25.31 (13.66-41.14) | 0.51 (0.30-0.84) | 0.50 (0.27-0.81) | -0.25 (-0.48,-0.02) |  | 14.39 (8.51-24.00) | 26.33 (14.13-42.16) | 0.54 (0.32-0.89) | 0.52 (0.28-0.83) | -0.24 (-0.47,-0.00) |  | 399.94 (243.43-641.11) | 748.11 (407.02-1201.15) | 14.90 (9.07-23.88) | 14.73 (8.01-23.64) | -0.27 (-0.58,0.03) |
| Thailand | 691.48 (455.74-993.85) | 1815.60 (1193.43-2607.26) | 2.44 (1.61-3.50) | 5.45 (3.58-7.82) | 2.24 (2.00,2.48) |  | 711.38 (471.84-1009.82) | 1809.06 (1185.93-2631.01) | 2.51 (1.66-3.56) | 5.43 (3.56-7.89) | 2.16 (1.94,2.38) |  | 20622.33 (13534.68-29952.68) | 47596.99 (30979.71-69956.58) | 72.66 (47.69-105.53) | 142.76 (92.92-209.82) | 1.82 (1.56,2.08) |
| Timor-Leste | 0.75 (0.43-1.23) | 2.30 (1.29-4.40) | 0.19 (0.11-0.31) | 0.33 (0.18-0.63) | 1.96 (1.85,2.07) |  | 0.76 (0.44-1.25) | 2.42 (1.35-4.57) | 0.20 (0.11-0.32) | 0.35 (0.19-0.65) | 2.06 (1.94,2.19) |  | 23.55 (13.18-39.86) | 64.07 (34.99-121.65) | 6.03 (3.37-10.20) | 9.17 (5.01-17.41) | 1.50 (1.34,1.65) |
| Togo | 9.39 (5.89-14.89) | 23.95 (13.46-42.10) | 0.51 (0.32-0.82) | 0.57 (0.32-1.01) | -0.16 (-0.31,-0.02) |  | 9.74 (6.05-15.64) | 24.68 (13.89-42.86) | 0.53 (0.33-0.86) | 0.59 (0.33-1.02) | -0.14 (-0.30,0.03) |  | 287.91 (183.26-462.12) | 739.75 (410.75-1354.68) | 15.79 (10.05-25.34) | 17.67 (9.81-32.37) | -0.10 (-0.27,0.07) |
| Tokelau | 0.00 (0.00-0.01) | 0.00 (0.00-0.01) | 0.35 (0.17-0.69) | 0.65 (0.35-1.13) | 1.87 (1.61,2.13) |  | 0.00 (0.00-0.01) | 0.00 (0.00-0.01) | 0.37 (0.19-0.73) | 0.67 (0.36-1.15) | 1.77 (1.50,2.05) |  | 0.08 (0.04-0.15) | 0.12 (0.07-0.21) | 9.49 (4.77-18.94) | 17.66 (9.50-31.15) | 2.00 (1.73,2.28) |
| Tonga | 0.67 (0.39-1.11) | 0.99 (0.58-1.60) | 1.35 (0.78-2.24) | 1.87 (1.09-3.02) | 0.57 (0.09,1.06) |  | 0.69 (0.40-1.15) | 1.02 (0.60-1.63) | 1.40 (0.81-2.33) | 1.92 (1.13-3.08) | 0.59 (0.10,1.09) |  | 19.54 (11.11-32.32) | 28.22 (16.27-46.26) | 39.54 (22.49-65.40) | 53.09 (30.62-87.03) | 0.58 (0.11,1.05) |
| Trinidad and Tobago | 2.74 (2.03-3.57) | 6.39 (4.21-8.96) | 0.45 (0.34-0.59) | 0.92 (0.60-1.29) | 2.23 (1.97,2.50) |  | 2.92 (2.14-3.82) | 6.72 (4.42-9.46) | 0.48 (0.36-0.63) | 0.96 (0.63-1.36) | 2.03 (1.70,2.36) |  | 74.33 (55.65-98.04) | 171.68 (112.10-243.70) | 12.34 (9.24-16.27) | 24.65 (16.09-34.99) | 2.07 (1.79,2.36) |
| Tunisia | 4.97 (3.14-8.01) | 18.31 (10.30-28.96) | 0.12 (0.08-0.19) | 0.31 (0.17-0.49) | 2.99 (2.81,3.17) |  | 5.26 (3.29-8.52) | 18.85 (10.62-29.68) | 0.13 (0.08-0.20) | 0.32 (0.18-0.50) | 2.88 (2.71,3.05) |  | 134.57 (84.81-213.80) | 473.98 (264.09-759.30) | 3.22 (2.03-5.12) | 8.00 (4.46-12.82) | 2.84 (2.65,3.02) |
| Turkey | 66.67 (42.07-104.51) | 197.58 (129.50-296.02) | 0.23 (0.15-0.36) | 0.47 (0.31-0.71) | 2.52 (2.14,2.90) |  | 70.70 (44.10-110.03) | 205.78 (135.66-307.43) | 0.25 (0.15-0.38) | 0.49 (0.32-0.74) | 2.43 (2.03,2.84) |  | 1827.50 (1160.53-2851.59) | 4885.09 (3222.11-7428.26) | 6.36 (4.04-9.92) | 11.69 (7.71-17.77) | 2.07 (1.66,2.48) |
| Turkmenistan | 10.91 (7.30-15.63) | 27.84 (18.31-40.17) | 0.59 (0.39-0.84) | 1.08 (0.71-1.56) | 2.63 (2.36,2.89) |  | 11.32 (7.60-16.09) | 28.60 (18.84-41.09) | 0.61 (0.41-0.87) | 1.11 (0.73-1.59) | 2.40 (1.95,2.85) |  | 324.64 (216.22-460.27) | 834.47 (543.24-1210.42) | 17.55 (11.69-24.88) | 32.35 (21.06-46.93) | 2.58 (2.13,3.03) |
| Tuvalu | 0.02 (0.01-0.03) | 0.03 (0.02-0.05) | 0.36 (0.20-0.63) | 0.49 (0.27-0.83) | 0.76 (0.63,0.89) |  | 0.02 (0.01-0.03) | 0.03 (0.02-0.05) | 0.37 (0.21-0.65) | 0.50 (0.28-0.87) | 0.78 (0.64,0.91) |  | 0.50 (0.29-0.86) | 0.87 (0.49-1.48) | 10.55 (6.06-18.13) | 14.09 (7.92-23.91) | 0.81 (0.69,0.93) |
| Uganda | 64.72 (41.58-94.48) | 142.47 (90.23-208.95) | 0.75 (0.48-1.09) | 0.66 (0.42-0.96) | -1.04 (-1.23,-0.85) |  | 67.43 (43.54-97.76) | 146.77 (92.87-216.25) | 0.78 (0.50-1.13) | 0.68 (0.43-1.00) | -1.05 (-1.24,-0.86) |  | 1916.80 (1250.93-2805.52) | 4442.31 (2724.74-6618.27) | 22.17 (14.47-32.45) | 20.51 (12.58-30.56) | -0.86 (-1.06,-0.66) |
| Ukraine | 194.79 (156.18-236.73) | 171.51 (117.67-238.16) | 0.74 (0.59-0.90) | 0.80 (0.55-1.11) | -0.53 (-1.37,0.33) |  | 200.35 (160.73-244.21) | 174.16 (118.21-242.82) | 0.76 (0.61-0.93) | 0.81 (0.55-1.13) | -0.30 (-1.20,0.60) |  | 5374.52 (4282.30-6528.50) | 4580.11 (3082.61-6563.27) | 20.39 (16.25-24.77) | 21.26 (14.31-30.47) | -0.40 (-1.29,0.50) |
| United Arab Emirates | 1.92 (1.12-2.98) | 23.12 (13.65-36.97) | 0.20 (0.12-0.32) | 0.48 (0.28-0.77) | 1.36 (0.62,2.10) |  | 1.95 (1.14-3.05) | 22.67 (13.30-35.91) | 0.21 (0.12-0.33) | 0.47 (0.28-0.75) | 1.23 (0.51,1.96) |  | 59.04 (34.48-91.41) | 715.18 (426.63-1150.86) | 6.31 (3.69-9.77) | 14.85 (8.86-23.90) | 1.48 (0.82,2.14) |
| United Kingdom | 330.45 (293.92-370.96) | 1520.76 (1355.81-1727.93) | 1.15 (1.03-1.29) | 4.48 (4.00-5.09) | 5.30 (5.06,5.54) |  | 310.69 (276.07-349.47) | 1268.60 (1127.46-1454.35) | 1.08 (0.96-1.22) | 3.74 (3.32-4.29) | 4.77 (4.54,5.00) |  | 7083.86 (6280.91-7907.01) | 26575.33 (23751.81-30157.14) | 24.73 (21.92-27.60) | 78.34 (70.01-88.90) | 4.50 (4.27,4.74) |
| United Republic of Tanzania | 70.56 (44.50-105.78) | 124.69 (75.12-194.57) | 0.55 (0.34-0.82) | 0.43 (0.26-0.67) | -1.38 (-1.56,-1.20) |  | 73.49 (46.34-108.69) | 129.63 (78.56-208.35) | 0.57 (0.36-0.84) | 0.44 (0.27-0.71) | -1.40 (-1.58,-1.21) |  | 2091.29 (1305.19-3168.17) | 3758.29 (2237.82-6006.57) | 16.19 (10.10-24.52) | 12.86 (7.66-20.55) | -1.33 (-1.52,-1.15) |
| United States of America | 1087.32 (982.42-1217.12) | 4829.26 (4248.61-5487.95) | 0.86 (0.77-0.96) | 2.90 (2.55-3.30) | 4.04 (3.98,4.10) |  | 933.55 (844.19-1046.56) | 3702.94 (3248.23-4209.30) | 0.73 (0.66-0.82) | 2.23 (1.95-2.53) | 3.70 (3.59,3.80) |  | 22621.52 (20467.60-25229.52) | 89018.41 (77962.18-101228.17) | 17.81 (16.11-19.86) | 53.52 (46.87-60.86) | 3.77 (3.70,3.84) |
| United States Virgin Islands | 0.23 (0.15-0.34) | 0.85 (0.53-1.33) | 0.43 (0.28-0.64) | 1.98 (1.23-3.09) | 5.32 (4.98,5.66) |  | 0.24 (0.16-0.35) | 0.90 (0.55-1.41) | 0.45 (0.29-0.67) | 2.11 (1.29-3.27) | 5.38 (5.05,5.71) |  | 6.37 (4.13-9.65) | 21.28 (12.89-33.89) | 12.01 (7.79-18.20) | 49.53 (30.00-78.90) | 4.97 (4.65,5.28) |
| Uruguay | 5.37 (3.80-7.05) | 18.88 (13.19-26.04) | 0.34 (0.24-0.45) | 1.11 (0.77-1.53) | 4.39 (4.15,4.64) |  | 5.65 (3.99-7.40) | 19.68 (13.75-27.33) | 0.36 (0.25-0.47) | 1.16 (0.81-1.61) | 4.45 (4.15,4.76) |  | 141.21 (100.72-187.32) | 460.48 (321.39-638.23) | 9.00 (6.42-11.93) | 27.04 (18.87-37.48) | 4.21 (3.90,4.51) |
| Uzbekistan | 36.42 (24.14-53.63) | 136.04 (85.84-208.32) | 0.35 (0.23-0.51) | 0.79 (0.50-1.22) | 2.41 (2.26,2.57) |  | 37.75 (25.24-56.12) | 140.02 (87.21-212.81) | 0.36 (0.24-0.54) | 0.82 (0.51-1.24) | 2.44 (2.29,2.60) |  | 1092.81 (704.79-1610.43) | 4051.52 (2536.59-6215.05) | 10.43 (6.73-15.37) | 23.67 (14.82-36.31) | 2.49 (2.32,2.65) |
| Vanuatu | 0.16 (0.09-0.33) | 0.43 (0.23-0.73) | 0.21 (0.11-0.43) | 0.28 (0.15-0.47) | 1.08 (0.89,1.27) |  | 0.16 (0.09-0.34) | 0.45 (0.24-0.76) | 0.22 (0.12-0.45) | 0.29 (0.15-0.49) | 1.09 (0.87,1.30) |  | 4.84 (2.62-9.76) | 12.88 (6.73-21.50) | 6.36 (3.45-12.82) | 8.23 (4.30-13.74) | 1.01 (0.82,1.20) |
| Venezuela (Bolivarian Republic of) | 80.11 (60.53-99.58) | 101.33 (68.53-142.36) | 0.85 (0.64-1.06) | 0.76 (0.51-1.07) | -0.24 (-1.38,0.91) |  | 84.88 (64.07-105.62) | 107.05 (72.41-150.71) | 0.90 (0.68-1.12) | 0.80 (0.54-1.13) | -0.37 (-1.73,1.02) |  | 2200.38 (1678.51-2747.81) | 2711.70 (1815.80-3880.13) | 23.40 (17.85-29.22) | 20.37 (13.64-29.14) | -0.41 (-1.73,0.93) |
| Viet Nam | 503.29 (312.66-786.51) | 1832.41 (1111.50-2903.29) | 1.48 (0.92-2.31) | 3.66 (2.22-5.79) | 3.25 (3.02,3.48) |  | 523.24 (330.24-805.05) | 1810.34 (1107.48-2871.95) | 1.53 (0.97-2.36) | 3.61 (2.21-5.73) | 3.07 (2.86,3.27) |  | 14647.50 (9083.34-22919.25) | 52205.44 (30921.20-84461.44) | 42.94 (26.63-67.19) | 104.13 (61.68-168.47) | 3.26 (3.00,3.51) |
| Yemen | 5.50 (1.86-11.26) | 10.03 (5.08-18.74) | 0.08 (0.03-0.17) | 0.06 (0.03-0.11) | -1.14 (-1.42,-0.86) |  | 5.75 (1.93-11.81) | 10.56 (5.37-19.91) | 0.08 (0.03-0.17) | 0.06 (0.03-0.12) | -1.13 (-1.40,-0.87) |  | 160.72 (55.79-333.95) | 282.72 (144.78-526.38) | 2.36 (0.82-4.90) | 1.68 (0.86-3.13) | -1.28 (-1.56,-1.00) |
| Zambia | 29.29 (19.13-43.47) | 34.42 (13.72-89.79) | 0.74 (0.48-1.10) | 0.35 (0.14-0.92) | -4.19 (-4.84,-3.53) |  | 30.33 (19.89-44.71) | 35.80 (14.49-92.02) | 0.76 (0.50-1.13) | 0.37 (0.15-0.94) | -4.19 (-4.88,-3.50) |  | 891.69 (581.10-1323.47) | 1052.37 (400.21-2911.89) | 22.47 (14.64-33.35) | 10.79 (4.10-29.84) | -4.23 (-4.93,-3.52) |
| Zimbabwe | 40.99 (20.75-68.53) | 74.39 (43.53-118.21) | 0.79 (0.40-1.33) | 0.95 (0.56-1.52) | -0.63 (-1.16,-0.09) |  | 42.90 (22.01-71.51) | 77.11 (45.12-121.96) | 0.83 (0.43-1.38) | 0.99 (0.58-1.56) | -0.61 (-1.18,-0.03) |  | 1179.58 (604.68-1985.18) | 2263.23 (1295.29-3561.15) | 22.81 (11.69-38.39) | 29.02 (16.61-45.67) | -0.32 (-0.91,0.27) |

**Abbreviations:** UI, uncertainty interval; ASR, age-standardised rate per 100,000; EAPC, estimated annual percentage change; CI, confidence interval; DALYs, disability-adjusted life-year; SDI, socio-demographic index.

**Table 13. Burden of hepatoblastoma in 204 countries and territories in 1990, 2021 and the estimated annual percentage changes (EAPC) from 1990 to 2021**

| **Country** | **Incidence** | | | | |  | **Deaths** | | | | |  | **DALYs** | | | | |
| --- | --- | --- | --- | --- | --- | --- | --- | --- | --- | --- | --- | --- | --- | --- | --- | --- | --- |
|  | **1990 No. (95% UI)** | **2021 No. (95% UI)** | **1990 ASR per 100 000 (95% UI)** | **2021 ASR per 100 000 (95% UI)** | **EAPC (95% CI)** |  | **1990 No. (95% UI)** | **2021 No. (95% UI)** | **1990 ASR per 100 000 (95% UI)** | **2021 ASR per 100 000 (95% UI)** | **EAPC (95% CI)** |  | **1990 No. (95% UI)** | **2021 No. (95% UI)** | **1990 ASR per 100 000 (95% UI)** | **2021 ASR per 100 000 (95% UI)** | **EAPC (95% CI)** |
| Afghanistan | 17.17 (5.74-30.34) | 25.72 (16.64-38.69) | 0.35 (0.12-0.61) | 0.16 (0.11-0.25) | -2.16 (-2.37,-1.94) |  | 12.25 (4.09-21.75) | 18.07 (11.79-27.27) | 0.25 (0.08-0.44) | 0.12 (0.08-0.17) | -2.22 (-2.43,-2.01) |  | 1086.36 (361.54-1928.72) | 1597.62 (1046.62-2412.12) | 21.85 (7.27-38.79) | 10.23 (6.70-15.45) | -2.22 (-2.43,-2.01) |
| Albania | 6.14 (4.06-8.81) | 0.93 (0.55-1.54) | 0.37 (0.25-0.53) | 0.07 (0.04-0.12) | -5.86 (-6.46,-5.26) |  | 4.14 (2.75-5.96) | 0.39 (0.23-0.66) | 0.25 (0.17-0.36) | 0.03 (0.02-0.05) | -7.47 (-7.94,-7.01) |  | 364.03 (242.45-522.64) | 34.81 (20.83-57.78) | 22.03 (14.67-31.63) | 2.61 (1.56-4.33) | -7.45 (-7.94,-6.96) |
| Algeria | 10.19 (7.27-14.47) | 7.66 (4.72-12.05) | 0.08 (0.06-0.11) | 0.03 (0.02-0.05) | -1.90 (-2.29,-1.51) |  | 6.99 (5.02-10.11) | 4.42 (2.71-6.98) | 0.06 (0.04-0.08) | 0.02 (0.01-0.03) | -2.49 (-2.84,-2.13) |  | 618.65 (444.28-894.35) | 390.76 (240.04-617.12) | 4.89 (3.51-7.07) | 1.77 (1.09-2.79) | -2.47 (-2.83,-2.11) |
| American Samoa | 0.00 (0.00-0.01) | 0.00 (0.00-0.00) | 0.01 (0.01-0.02) | 0.01 (0.00-0.02) | -0.63 (-1.36,0.11) |  | 0.00 (0.00-0.00) | 0.00 (0.00-0.00) | 0.01 (0.01-0.02) | 0.01 (0.00-0.01) | -0.69 (-1.46,0.08) |  | 0.20 (0.11-0.36) | 0.15 (0.07-0.29) | 0.81 (0.45-1.48) | 0.59 (0.30-1.16) | -0.71 (-1.46,0.05) |
| Andorra | 0.05 (0.02-0.08) | 0.02 (0.01-0.04) | 0.18 (0.07-0.31) | 0.06 (0.03-0.09) | -3.32 (-3.73,-2.91) |  | 0.01 (0.01-0.02) | 0.01 (0.00-0.01) | 0.05 (0.02-0.09) | 0.01 (0.01-0.02) | -3.90 (-4.22,-3.58) |  | 1.20 (0.51-2.11) | 0.46 (0.28-0.73) | 4.43 (1.88-7.76) | 1.09 (0.64-1.71) | -3.91 (-4.23,-3.59) |
| Angola | 32.00 (11.25-61.64) | 23.57 (7.65-47.83) | 0.62 (0.22-1.20) | 0.14 (0.05-0.29) | -4.69 (-4.97,-4.41) |  | 22.77 (8.04-43.43) | 16.49 (5.38-33.96) | 0.44 (0.16-0.85) | 0.10 (0.03-0.21) | -4.74 (-5.03,-4.45) |  | 2004.76 (706.50-3832.81) | 1449.64 (470.79-2983.57) | 39.02 (13.75-74.60) | 8.86 (2.88-18.24) | -4.74 (-5.03,-4.45) |
| Antigua and Barbuda | 0.00 (0.00-0.00) | 0.00 (0.00-0.00) | 0.01 (0.01-0.02) | 0.01 (0.01-0.01) | -2.04 (-2.35,-1.72) |  | 0.00 (0.00-0.00) | 0.00 (0.00-0.00) | 0.01 (0.01-0.01) | 0.00 (0.00-0.01) | -2.41 (-2.74,-2.08) |  | 0.24 (0.19-0.30) | 0.18 (0.15-0.21) | 0.79 (0.62-0.99) | 0.39 (0.32-0.47) | -2.41 (-2.73,-2.08) |
| Argentina | 1.19 (1.00-1.39) | 1.36 (1.08-1.66) | 0.01 (0.01-0.01) | 0.01 (0.00-0.01) | 0.15 (-0.17,0.47) |  | 0.80 (0.68-0.94) | 0.83 (0.66-1.01) | 0.00 (0.00-0.01) | 0.00 (0.00-0.00) | -0.26 (-0.56,0.05) |  | 70.51 (59.66-82.49) | 71.87 (57.77-88.05) | 0.43 (0.36-0.50) | 0.32 (0.25-0.39) | -0.27 (-0.58,0.04) |
| Armenia | 1.34 (1.08-1.69) | 0.36 (0.27-0.49) | 0.08 (0.06-0.10) | 0.02 (0.02-0.03) | -3.13 (-3.61,-2.65) |  | 0.89 (0.71-1.13) | 0.21 (0.16-0.28) | 0.05 (0.04-0.07) | 0.01 (0.01-0.02) | -3.70 (-4.15,-3.25) |  | 78.28 (62.93-99.22) | 18.21 (13.85-24.49) | 4.58 (3.68-5.80) | 1.22 (0.92-1.64) | -3.72 (-4.17,-3.26) |
| Australia | 2.57 (2.26-2.89) | 5.79 (4.43-7.36) | 0.03 (0.03-0.03) | 0.04 (0.03-0.06) | 1.41 (1.12,1.70) |  | 0.78 (0.69-0.88) | 1.28 (1.00-1.60) | 0.01 (0.01-0.01) | 0.01 (0.01-0.01) | 0.49 (0.22,0.76) |  | 69.59 (61.35-78.56) | 114.24 (89.79-143.07) | 0.83 (0.73-0.93) | 0.89 (0.70-1.11) | 0.51 (0.24,0.77) |
| Austria | 1.00 (0.87-1.17) | 1.10 (0.84-1.44) | 0.03 (0.02-0.03) | 0.02 (0.02-0.03) | -0.39 (-0.61,-0.17) |  | 0.38 (0.32-0.44) | 0.24 (0.18-0.32) | 0.01 (0.01-0.01) | 0.01 (0.00-0.01) | -1.91 (-2.25,-1.57) |  | 33.43 (28.74-38.75) | 21.63 (16.62-28.07) | 0.86 (0.74-1.00) | 0.48 (0.37-0.63) | -1.88 (-2.21,-1.54) |
| Azerbaijan | 4.10 (2.06-7.28) | 2.40 (1.29-4.35) | 0.11 (0.06-0.20) | 0.05 (0.02-0.08) | -2.98 (-3.29,-2.66) |  | 2.83 (1.44-4.96) | 1.58 (0.88-2.77) | 0.08 (0.04-0.14) | 0.03 (0.02-0.05) | -3.24 (-3.54,-2.94) |  | 248.88 (125.85-437.60) | 137.28 (75.99-241.91) | 6.79 (3.44-11.95) | 2.61 (1.45-4.61) | -3.25 (-3.54,-2.95) |
| Bahamas | 0.03 (0.02-0.03) | 0.01 (0.01-0.02) | 0.02 (0.02-0.03) | 0.01 (0.01-0.01) | -3.89 (-4.17,-3.62) |  | 0.02 (0.02-0.02) | 0.01 (0.01-0.01) | 0.02 (0.01-0.02) | 0.00 (0.00-0.01) | -4.10 (-4.40,-3.79) |  | 1.72 (1.39-2.09) | 0.83 (0.61-1.14) | 1.34 (1.08-1.63) | 0.43 (0.31-0.59) | -4.09 (-4.38,-3.80) |
| Bahrain | 0.11 (0.08-0.15) | 0.11 (0.07-0.15) | 0.04 (0.03-0.06) | 0.01 (0.01-0.02) | -3.32 (-4.24,-2.40) |  | 0.07 (0.05-0.10) | 0.05 (0.03-0.07) | 0.03 (0.02-0.04) | 0.01 (0.00-0.01) | -4.78 (-5.54,-4.02) |  | 6.44 (4.72-8.76) | 4.26 (2.80-5.89) | 2.54 (1.86-3.46) | 0.56 (0.37-0.77) | -4.75 (-5.51,-3.98) |
| Bangladesh | 88.97 (33.44-147.14) | 27.75 (18.20-39.32) | 0.16 (0.06-0.27) | 0.03 (0.02-0.05) | -4.68 (-4.84,-4.52) |  | 63.10 (23.87-105.59) | 18.75 (12.42-26.90) | 0.12 (0.04-0.19) | 0.02 (0.02-0.03) | -4.84 (-5.01,-4.68) |  | 5560.81 (2108.25-9297.44) | 1644.09 (1088.53-2344.27) | 10.19 (3.86-17.04) | 2.00 (1.32-2.85) | -4.85 (-5.02,-4.69) |
| Barbados | 0.01 (0.01-0.02) | 0.01 (0.00-0.01) | 0.01 (0.01-0.01) | 0.00 (0.00-0.01) | -2.61 (-3.07,-2.15) |  | 0.01 (0.01-0.01) | 0.00 (0.00-0.01) | 0.01 (0.01-0.01) | 0.00 (0.00-0.00) | -2.90 (-3.36,-2.43) |  | 0.81 (0.65-0.98) | 0.36 (0.26-0.50) | 0.64 (0.51-0.77) | 0.24 (0.17-0.34) | -2.91 (-3.37,-2.45) |
| Belarus | 0.85 (0.66-1.12) | 1.92 (1.17-2.95) | 0.02 (0.01-0.02) | 0.04 (0.03-0.06) | 5.03 (4.05,6.01) |  | 0.53 (0.41-0.69) | 0.72 (0.47-1.07) | 0.01 (0.01-0.01) | 0.02 (0.01-0.02) | 3.02 (2.13,3.91) |  | 46.59 (36.26-60.76) | 63.77 (40.83-94.35) | 0.89 (0.69-1.16) | 1.37 (0.88-2.02) | 3.03 (2.15,3.92) |
| Belgium | 1.11 (0.94-1.29) | 1.18 (0.89-1.55) | 0.02 (0.02-0.03) | 0.02 (0.02-0.03) | 0.02 (-0.38,0.41) |  | 0.40 (0.34-0.47) | 0.26 (0.20-0.35) | 0.01 (0.01-0.01) | 0.00 (0.00-0.01) | -1.47 (-1.88,-1.06) |  | 35.69 (30.31-41.70) | 23.49 (17.79-30.83) | 0.72 (0.61-0.84) | 0.41 (0.31-0.54) | -1.44 (-1.85,-1.02) |
| Belize | 0.06 (0.05-0.07) | 0.02 (0.02-0.03) | 0.06 (0.05-0.07) | 0.01 (0.01-0.01) | -5.28 (-5.44,-5.12) |  | 0.04 (0.03-0.05) | 0.02 (0.01-0.02) | 0.04 (0.03-0.05) | 0.01 (0.01-0.01) | -5.40 (-5.57,-5.23) |  | 3.36 (2.79-4.15) | 1.47 (1.19-1.82) | 3.59 (2.98-4.44) | 0.68 (0.55-0.85) | -5.41 (-5.58,-5.24) |
| Benin | 14.50 (9.11-22.47) | 16.92 (9.46-27.46) | 0.60 (0.38-0.93) | 0.25 (0.14-0.41) | -2.70 (-3.00,-2.41) |  | 10.21 (6.43-15.96) | 11.69 (6.58-18.91) | 0.42 (0.26-0.66) | 0.17 (0.10-0.28) | -2.76 (-3.05,-2.46) |  | 909.26 (572.61-1422.60) | 1043.01 (585.81-1681.09) | 37.49 (23.61-58.66) | 15.45 (8.68-24.91) | -2.75 (-3.05,-2.45) |
| Bermuda | 0.00 (0.00-0.00) | 0.00 (0.00-0.00) | 0.01 (0.01-0.01) | 0.00 (0.00-0.01) | -1.64 (-2.05,-1.22) |  | 0.00 (0.00-0.00) | 0.00 (0.00-0.00) | 0.01 (0.00-0.01) | 0.00 (0.00-0.00) | -4.45 (-4.81,-4.08) |  | 0.17 (0.13-0.22) | 0.04 (0.03-0.05) | 0.57 (0.43-0.73) | 0.12 (0.09-0.16) | -4.43 (-4.80,-4.06) |
| Bhutan | 0.51 (0.21-0.94) | 0.26 (0.12-0.46) | 0.16 (0.07-0.30) | 0.07 (0.03-0.12) | -3.19 (-3.60,-2.78) |  | 0.36 (0.15-0.67) | 0.17 (0.08-0.31) | 0.12 (0.05-0.21) | 0.05 (0.02-0.08) | -3.36 (-3.78,-2.95) |  | 32.13 (13.53-58.98) | 15.44 (7.35-26.92) | 10.20 (4.29-18.72) | 4.08 (1.94-7.11) | -3.37 (-3.78,-2.95) |
| Bolivia (Plurinational State of) | 5.74 (2.86-9.14) | 2.60 (1.63-4.27) | 0.18 (0.09-0.29) | 0.04 (0.03-0.07) | -4.60 (-4.81,-4.39) |  | 4.07 (2.04-6.49) | 1.79 (1.13-2.90) | 0.13 (0.06-0.20) | 0.03 (0.02-0.05) | -4.72 (-4.92,-4.52) |  | 359.56 (179.69-575.68) | 156.68 (99.28-255.19) | 11.27 (5.63-18.04) | 2.66 (1.68-4.33) | -4.73 (-4.93,-4.53) |
| Bosnia and Herzegovina | 0.73 (0.52-0.99) | 0.13 (0.09-0.18) | 0.03 (0.02-0.04) | 0.01 (0.01-0.01) | -5.45 (-6.34,-4.55) |  | 0.50 (0.36-0.67) | 0.06 (0.04-0.09) | 0.02 (0.02-0.03) | 0.00 (0.00-0.01) | -6.81 (-7.72,-5.88) |  | 42.96 (30.71-58.25) | 5.44 (3.83-7.44) | 1.91 (1.37-2.59) | 0.33 (0.23-0.45) | -6.78 (-7.70,-5.86) |
| Botswana | 0.22 (0.11-0.38) | 0.43 (0.23-0.79) | 0.03 (0.02-0.06) | 0.04 (0.02-0.07) | 0.44 (0.22,0.66) |  | 0.16 (0.08-0.28) | 0.30 (0.16-0.56) | 0.02 (0.01-0.04) | 0.02 (0.01-0.05) | 0.33 (0.12,0.54) |  | 13.66 (7.03-24.59) | 26.04 (14.17-49.26) | 2.07 (1.07-3.73) | 2.18 (1.18-4.12) | 0.33 (0.12,0.54) |
| Brazil | 37.42 (32.16-44.18) | 18.54 (14.36-22.95) | 0.05 (0.04-0.06) | 0.02 (0.01-0.02) | -3.08 (-3.35,-2.80) |  | 26.04 (22.44-30.80) | 11.91 (9.18-14.71) | 0.04 (0.03-0.04) | 0.01 (0.01-0.01) | -3.31 (-3.60,-3.02) |  | 2293.09 (1978.35-2712.26) | 1047.75 (807.92-1293.75) | 3.09 (2.66-3.65) | 0.95 (0.73-1.17) | -3.31 (-3.60,-3.03) |
| Brunei Darussalam | 0.06 (0.04-0.09) | 0.05 (0.03-0.07) | 0.05 (0.03-0.07) | 0.02 (0.01-0.03) | -2.80 (-2.93,-2.66) |  | 0.04 (0.03-0.06) | 0.03 (0.02-0.04) | 0.03 (0.02-0.05) | 0.01 (0.01-0.02) | -3.18 (-3.33,-3.03) |  | 3.76 (2.52-5.53) | 2.42 (1.58-3.74) | 2.90 (1.94-4.27) | 1.07 (0.70-1.66) | -3.16 (-3.31,-3.01) |
| Bulgaria | 1.89 (1.43-2.44) | 0.24 (0.17-0.33) | 0.04 (0.03-0.06) | 0.01 (0.01-0.01) | -5.23 (-5.84,-4.62) |  | 1.21 (0.92-1.56) | 0.13 (0.09-0.18) | 0.03 (0.02-0.04) | 0.00 (0.00-0.01) | -5.81 (-6.38,-5.25) |  | 105.93 (80.44-136.70) | 11.50 (8.25-15.74) | 2.44 (1.85-3.15) | 0.34 (0.24-0.46) | -5.82 (-6.38,-5.26) |
| Burkina Faso | 30.34 (18.20-50.36) | 42.72 (20.96-71.92) | 0.64 (0.38-1.06) | 0.38 (0.18-0.63) | -1.51 (-1.72,-1.30) |  | 21.32 (12.79-35.73) | 29.65 (14.58-50.91) | 0.45 (0.27-0.75) | 0.26 (0.13-0.45) | -1.55 (-1.76,-1.34) |  | 1899.18 (1138.56-3189.46) | 2648.99 (1304.39-4547.40) | 39.86 (23.90-66.95) | 23.28 (11.46-39.96) | -1.54 (-1.75,-1.33) |
| Burundi | 6.48 (3.51-11.93) | 4.04 (1.61-9.33) | 0.23 (0.13-0.43) | 0.06 (0.02-0.14) | -3.42 (-3.83,-3.00) |  | 4.62 (2.52-8.44) | 2.87 (1.16-6.46) | 0.17 (0.09-0.30) | 0.04 (0.02-0.10) | -3.43 (-3.84,-3.02) |  | 407.25 (223.69-742.13) | 252.04 (101.08-567.85) | 14.67 (8.06-26.73) | 3.81 (1.53-8.59) | -3.44 (-3.85,-3.03) |
| Cabo Verde | 0.28 (0.13-0.43) | 0.07 (0.05-0.12) | 0.16 (0.07-0.25) | 0.03 (0.02-0.04) | -6.28 (-6.65,-5.91) |  | 0.19 (0.09-0.30) | 0.05 (0.03-0.07) | 0.11 (0.05-0.17) | 0.02 (0.01-0.03) | -6.54 (-6.92,-6.16) |  | 17.12 (7.94-26.53) | 4.24 (2.64-6.72) | 9.68 (4.49-14.99) | 1.52 (0.94-2.40) | -6.55 (-6.93,-6.17) |
| Cambodia | 12.75 (6.53-22.39) | 3.62 (1.93-6.94) | 0.25 (0.13-0.44) | 0.04 (0.02-0.08) | -6.14 (-6.42,-5.85) |  | 9.09 (4.79-15.83) | 2.51 (1.30-4.74) | 0.18 (0.09-0.31) | 0.03 (0.02-0.06) | -6.24 (-6.52,-5.96) |  | 801.05 (421.27-1394.63) | 219.90 (114.88-416.02) | 15.60 (8.20-27.15) | 2.58 (1.35-4.88) | -6.25 (-6.53,-5.97) |
| Cameroon | 17.55 (9.04-28.11) | 22.28 (8.56-43.68) | 0.34 (0.17-0.54) | 0.14 (0.05-0.27) | -2.43 (-2.63,-2.23) |  | 12.31 (6.34-19.56) | 15.35 (5.87-30.29) | 0.24 (0.12-0.37) | 0.10 (0.04-0.19) | -2.49 (-2.69,-2.29) |  | 1095.67 (566.71-1740.39) | 1369.03 (525.03-2696.79) | 21.00 (10.86-33.35) | 8.62 (3.30-16.97) | -2.48 (-2.69,-2.28) |
| Canada | 4.44 (3.94-5.01) | 8.17 (6.58-10.06) | 0.03 (0.03-0.04) | 0.04 (0.04-0.05) | 1.74 (1.35,2.13) |  | 1.19 (1.06-1.33) | 1.80 (1.47-2.19) | 0.01 (0.01-0.01) | 0.01 (0.01-0.01) | 1.12 (0.72,1.52) |  | 106.04 (94.60-118.60) | 161.04 (131.75-195.50) | 0.78 (0.69-0.87) | 0.86 (0.70-1.04) | 1.14 (0.74,1.54) |
| Central African Republic | 4.79 (2.55-7.83) | 3.51 (1.75-6.45) | 0.35 (0.19-0.57) | 0.13 (0.06-0.24) | -3.57 (-3.74,-3.40) |  | 3.42 (1.85-5.54) | 2.51 (1.23-4.50) | 0.25 (0.14-0.41) | 0.09 (0.04-0.16) | -3.58 (-3.75,-3.40) |  | 302.03 (163.58-489.71) | 220.77 (109.08-398.87) | 22.12 (11.98-35.87) | 8.05 (3.98-14.55) | -3.58 (-3.76,-3.41) |
| Chad | 11.50 (6.90-18.16) | 23.92 (14.05-37.60) | 0.38 (0.23-0.60) | 0.27 (0.16-0.42) | -1.10 (-1.32,-0.87) |  | 8.11 (4.96-12.91) | 16.74 (9.88-26.16) | 0.27 (0.16-0.43) | 0.19 (0.11-0.29) | -1.12 (-1.35,-0.89) |  | 721.19 (439.85-1147.72) | 1489.78 (879.29-2320.93) | 23.93 (14.60-38.09) | 16.79 (9.91-26.15) | -1.12 (-1.35,-0.89) |
| Chile | 0.48 (0.38-0.63) | 0.51 (0.39-0.67) | 0.01 (0.01-0.01) | 0.01 (0.00-0.01) | 0.30 (-0.13,0.74) |  | 0.32 (0.25-0.42) | 0.21 (0.16-0.27) | 0.00 (0.00-0.01) | 0.00 (0.00-0.00) | -1.49 (-1.84,-1.13) |  | 28.48 (22.37-37.10) | 18.66 (14.29-23.85) | 0.43 (0.34-0.56) | 0.20 (0.15-0.25) | -1.48 (-1.85,-1.12) |
| China | 1134.56 (940.32-1392.10) | 276.58 (195.43-391.68) | 0.19 (0.16-0.24) | 0.04 (0.03-0.06) | -5.42 (-5.98,-4.86) |  | 785.06 (652.64-962.19) | 107.89 (77.49-150.88) | 0.13 (0.11-0.16) | 0.02 (0.01-0.02) | -7.40 (-7.88,-6.91) |  | 69358.17 (57685.08-84901.10) | 9489.07 (6806.95-13320.31) | 11.79 (9.81-14.43) | 1.33 (0.96-1.87) | -7.38 (-7.86,-6.90) |
| Colombia | 11.02 (9.27-13.11) | 5.15 (3.61-7.21) | 0.07 (0.06-0.08) | 0.02 (0.01-0.03) | -3.26 (-3.68,-2.84) |  | 7.64 (6.45-9.08) | 2.99 (2.14-4.11) | 0.05 (0.04-0.06) | 0.01 (0.01-0.02) | -3.83 (-4.23,-3.42) |  | 670.62 (565.95-796.91) | 260.93 (185.98-358.77) | 4.13 (3.48-4.91) | 1.06 (0.76-1.46) | -3.85 (-4.26,-3.44) |
| Comoros | 0.49 (0.26-0.77) | 0.30 (0.17-0.51) | 0.21 (0.11-0.33) | 0.08 (0.05-0.14) | -3.19 (-3.43,-2.95) |  | 0.34 (0.18-0.54) | 0.21 (0.12-0.36) | 0.15 (0.08-0.24) | 0.06 (0.03-0.10) | -3.23 (-3.46,-2.99) |  | 30.44 (16.25-48.21) | 18.15 (10.44-31.20) | 13.16 (7.02-20.85) | 4.88 (2.81-8.38) | -3.25 (-3.48,-3.01) |
| Congo | 3.32 (1.68-5.86) | 1.51 (0.59-3.33) | 0.28 (0.14-0.49) | 0.06 (0.02-0.12) | -5.26 (-5.57,-4.95) |  | 2.35 (1.20-4.10) | 1.06 (0.41-2.31) | 0.20 (0.10-0.34) | 0.04 (0.02-0.09) | -5.31 (-5.62,-4.99) |  | 207.35 (106.19-360.15) | 92.92 (35.76-202.54) | 17.27 (8.84-29.99) | 3.45 (1.33-7.51) | -5.33 (-5.65,-5.01) |
| Cook Islands | 0.00 (0.00-0.00) | 0.00 (0.00-0.00) | 0.03 (0.02-0.05) | 0.01 (0.00-0.01) | -6.09 (-6.97,-5.21) |  | 0.00 (0.00-0.00) | 0.00 (0.00-0.00) | 0.02 (0.01-0.03) | 0.00 (0.00-0.01) | -6.87 (-7.73,-5.99) |  | 0.16 (0.09-0.27) | 0.02 (0.01-0.05) | 1.70 (0.97-2.90) | 0.26 (0.13-0.57) | -6.88 (-7.75,-6.00) |
| Costa Rica | 1.30 (1.14-1.50) | 0.66 (0.50-0.85) | 0.09 (0.07-0.10) | 0.03 (0.02-0.04) | -3.40 (-3.96,-2.84) |  | 0.83 (0.73-0.95) | 0.35 (0.27-0.44) | 0.05 (0.05-0.06) | 0.01 (0.01-0.02) | -4.05 (-4.58,-3.53) |  | 73.50 (64.22-84.54) | 30.27 (23.87-38.38) | 4.83 (4.22-5.56) | 1.27 (1.01-1.62) | -4.09 (-4.61,-3.55) |
| Côte d'Ivoire | 25.42 (14.22-41.96) | 19.66 (11.74-32.38) | 0.42 (0.23-0.69) | 0.14 (0.08-0.23) | -3.33 (-3.56,-3.10) |  | 17.87 (9.98-29.66) | 13.58 (8.14-22.92) | 0.29 (0.16-0.49) | 0.10 (0.06-0.16) | -3.38 (-3.62,-3.15) |  | 1589.78 (885.22-2642.18) | 1205.56 (720.28-2019.07) | 26.07 (14.51-43.32) | 8.65 (5.17-14.49) | -3.39 (-3.62,-3.16) |
| Croatia | 0.41 (0.30-0.54) | 0.11 (0.07-0.16) | 0.02 (0.01-0.02) | 0.01 (0.00-0.01) | -2.98 (-3.64,-2.32) |  | 0.22 (0.16-0.29) | 0.03 (0.02-0.04) | 0.01 (0.01-0.01) | 0.00 (0.00-0.00) | -5.69 (-6.32,-5.07) |  | 19.44 (14.27-25.71) | 2.49 (1.72-3.71) | 0.80 (0.59-1.06) | 0.12 (0.08-0.18) | -5.64 (-6.27,-5.01) |
| Cuba | 0.64 (0.53-0.78) | 0.26 (0.19-0.33) | 0.01 (0.01-0.01) | 0.00 (0.00-0.01) | -3.37 (-3.95,-2.79) |  | 0.41 (0.35-0.50) | 0.13 (0.09-0.16) | 0.01 (0.01-0.01) | 0.00 (0.00-0.00) | -4.15 (-4.71,-3.59) |  | 35.88 (30.28-43.65) | 11.13 (8.30-14.39) | 0.66 (0.56-0.80) | 0.20 (0.15-0.26) | -4.14 (-4.70,-3.58) |
| Cyprus | 0.09 (0.05-0.13) | 0.08 (0.05-0.13) | 0.02 (0.01-0.03) | 0.01 (0.01-0.02) | -1.36 (-1.63,-1.09) |  | 0.05 (0.03-0.07) | 0.02 (0.01-0.03) | 0.01 (0.01-0.02) | 0.00 (0.00-0.00) | -4.36 (-4.62,-4.10) |  | 4.01 (2.51-6.31) | 1.69 (1.05-2.65) | 1.03 (0.65-1.62) | 0.25 (0.15-0.39) | -4.34 (-4.59,-4.09) |
| Czechia | 1.07 (0.89-1.30) | 0.27 (0.19-0.38) | 0.02 (0.02-0.03) | 0.01 (0.00-0.01) | -4.29 (-4.82,-3.75) |  | 0.63 (0.52-0.76) | 0.08 (0.05-0.10) | 0.01 (0.01-0.01) | 0.00 (0.00-0.00) | -6.64 (-7.24,-6.04) |  | 55.04 (45.92-66.50) | 6.67 (4.74-9.08) | 1.07 (0.89-1.29) | 0.13 (0.09-0.17) | -6.63 (-7.22,-6.03) |
| Democratic People's Republic of Korea | 17.55 (8.31-34.69) | 3.12 (1.57-6.87) | 0.17 (0.08-0.34) | 0.02 (0.01-0.05) | -6.35 (-6.67,-6.04) |  | 12.00 (5.69-24.19) | 2.01 (0.99-4.47) | 0.12 (0.06-0.23) | 0.02 (0.01-0.03) | -6.54 (-6.88,-6.21) |  | 1061.72 (503.41-2150.01) | 177.04 (87.28-393.55) | 10.31 (4.89-20.88) | 1.34 (0.66-2.98) | -6.56 (-6.89,-6.23) |
| Democratic Republic of the Congo | 33.69 (14.11-66.08) | 18.69 (9.44-39.42) | 0.18 (0.07-0.35) | 0.04 (0.02-0.09) | -4.22 (-4.51,-3.93) |  | 23.97 (10.04-46.89) | 13.24 (6.65-28.26) | 0.13 (0.05-0.25) | 0.03 (0.01-0.06) | -4.24 (-4.53,-3.95) |  | 2118.76 (885.12-4148.15) | 1162.57 (582.05-2493.13) | 11.11 (4.64-21.75) | 2.58 (1.29-5.54) | -4.26 (-4.55,-3.97) |
| Denmark | 0.31 (0.27-0.35) | 0.22 (0.17-0.28) | 0.01 (0.01-0.01) | 0.01 (0.01-0.01) | -1.37 (-1.80,-0.93) |  | 0.13 (0.11-0.15) | 0.05 (0.04-0.06) | 0.01 (0.00-0.01) | 0.00 (0.00-0.00) | -3.53 (-3.75,-3.31) |  | 11.49 (10.13-13.10) | 4.45 (3.42-5.53) | 0.45 (0.39-0.51) | 0.15 (0.12-0.19) | -3.49 (-3.72,-3.27) |
| Djibouti | 0.32 (0.19-0.58) | 0.44 (0.20-0.88) | 0.16 (0.09-0.28) | 0.07 (0.03-0.14) | -2.15 (-2.58,-1.72) |  | 0.23 (0.13-0.43) | 0.30 (0.14-0.62) | 0.11 (0.06-0.21) | 0.05 (0.02-0.10) | -2.20 (-2.63,-1.77) |  | 20.06 (11.48-37.81) | 26.76 (12.39-54.51) | 9.68 (5.54-18.25) | 4.25 (1.97-8.66) | -2.20 (-2.64,-1.77) |
| Dominica | 0.01 (0.00-0.01) | 0.00 (0.00-0.01) | 0.01 (0.01-0.02) | 0.01 (0.01-0.02) | -0.01 (-0.51,0.49) |  | 0.00 (0.00-0.01) | 0.00 (0.00-0.00) | 0.01 (0.01-0.02) | 0.01 (0.01-0.01) | -0.04 (-0.55,0.48) |  | 0.32 (0.21-0.50) | 0.28 (0.18-0.43) | 0.90 (0.57-1.38) | 0.83 (0.54-1.27) | -0.06 (-0.57,0.45) |
| Dominican Republic | 1.82 (1.00-2.70) | 0.94 (0.60-1.34) | 0.05 (0.03-0.08) | 0.02 (0.01-0.02) | -3.48 (-3.82,-3.14) |  | 1.27 (0.70-1.88) | 0.63 (0.40-0.91) | 0.04 (0.02-0.05) | 0.01 (0.01-0.02) | -3.59 (-3.94,-3.23) |  | 112.18 (61.61-166.37) | 55.50 (35.80-79.69) | 3.14 (1.72-4.65) | 1.01 (0.65-1.45) | -3.59 (-3.94,-3.23) |
| Ecuador | 4.91 (4.05-5.89) | 1.74 (1.25-2.40) | 0.10 (0.08-0.12) | 0.02 (0.01-0.03) | -5.75 (-6.57,-4.92) |  | 3.45 (2.85-4.18) | 1.14 (0.83-1.54) | 0.07 (0.06-0.08) | 0.01 (0.01-0.02) | -5.97 (-6.77,-5.16) |  | 302.81 (250.08-366.44) | 98.90 (72.19-134.25) | 6.07 (5.01-7.34) | 1.09 (0.80-1.49) | -5.99 (-6.80,-5.18) |
| Egypt | 78.48 (48.23-114.32) | 56.96 (41.88-80.59) | 0.28 (0.17-0.41) | 0.11 (0.08-0.15) | -1.92 (-2.32,-1.52) |  | 55.20 (33.90-80.02) | 36.54 (27.21-50.65) | 0.20 (0.12-0.29) | 0.07 (0.05-0.10) | -2.20 (-2.60,-1.80) |  | 4868.06 (2993.01-7061.87) | 3226.13 (2397.68-4478.98) | 17.60 (10.82-25.52) | 6.11 (4.54-8.48) | -2.19 (-2.59,-1.79) |
| El Salvador | 1.82 (1.38-2.27) | 0.42 (0.29-0.65) | 0.07 (0.05-0.09) | 0.01 (0.01-0.02) | -5.26 (-5.65,-4.88) |  | 1.27 (0.98-1.59) | 0.27 (0.18-0.41) | 0.05 (0.04-0.06) | 0.01 (0.01-0.01) | -5.56 (-5.95,-5.18) |  | 112.24 (86.46-139.91) | 23.46 (16.06-36.03) | 4.23 (3.26-5.27) | 0.73 (0.50-1.12) | -5.59 (-5.98,-5.20) |
| Equatorial Guinea | 0.24 (0.09-0.51) | 0.40 (0.20-0.75) | 0.11 (0.04-0.24) | 0.05 (0.03-0.10) | -2.67 (-2.86,-2.48) |  | 0.17 (0.07-0.36) | 0.27 (0.14-0.52) | 0.08 (0.03-0.17) | 0.04 (0.02-0.07) | -2.83 (-3.03,-2.64) |  | 15.18 (5.77-31.63) | 24.08 (12.08-45.31) | 7.18 (2.73-14.96) | 3.18 (1.60-5.99) | -2.84 (-3.04,-2.64) |
| Eritrea | 2.62 (1.37-4.59) | 2.73 (1.17-6.19) | 0.15 (0.08-0.27) | 0.08 (0.04-0.19) | -2.28 (-2.46,-2.10) |  | 1.86 (0.97-3.29) | 1.92 (0.82-4.38) | 0.11 (0.06-0.19) | 0.06 (0.02-0.13) | -2.31 (-2.49,-2.13) |  | 163.99 (85.80-290.58) | 168.88 (72.09-385.91) | 9.63 (5.04-17.06) | 5.12 (2.19-11.70) | -2.31 (-2.49,-2.13) |
| Estonia | 0.20 (0.16-0.25) | 0.29 (0.22-0.39) | 0.03 (0.02-0.03) | 0.04 (0.03-0.06) | 1.54 (0.66,2.43) |  | 0.12 (0.10-0.15) | 0.09 (0.07-0.12) | 0.02 (0.01-0.02) | 0.01 (0.01-0.02) | -1.27 (-2.17,-0.37) |  | 10.93 (8.79-13.30) | 7.97 (5.92-10.38) | 1.39 (1.12-1.70) | 1.22 (0.90-1.58) | -1.25 (-2.13,-0.36) |
| Eswatini | 0.30 (0.15-0.48) | 0.45 (0.18-1.07) | 0.07 (0.04-0.12) | 0.08 (0.03-0.19) | 0.27 (-0.39,0.93) |  | 0.21 (0.11-0.34) | 0.32 (0.13-0.79) | 0.05 (0.03-0.08) | 0.06 (0.02-0.14) | 0.29 (-0.38,0.97) |  | 18.56 (9.73-29.77) | 28.08 (10.87-68.63) | 4.60 (2.41-7.38) | 4.86 (1.88-11.88) | 0.27 (-0.41,0.95) |
| Ethiopia | 53.87 (26.96-90.52) | 36.58 (18.79-75.27) | 0.21 (0.11-0.36) | 0.07 (0.03-0.14) | -4.08 (-4.36,-3.80) |  | 38.42 (19.42-63.52) | 25.54 (13.10-52.60) | 0.15 (0.08-0.25) | 0.05 (0.02-0.10) | -4.14 (-4.42,-3.87) |  | 3392.93 (1718.36-5624.00) | 2246.43 (1149.86-4637.27) | 13.42 (6.80-22.24) | 4.12 (2.11-8.51) | -4.15 (-4.43,-3.88) |
| Fiji | 0.06 (0.03-0.11) | 0.09 (0.05-0.16) | 0.01 (0.01-0.03) | 0.02 (0.01-0.04) | 1.43 (1.19,1.66) |  | 0.04 (0.02-0.08) | 0.06 (0.03-0.11) | 0.01 (0.01-0.02) | 0.01 (0.01-0.02) | 1.30 (1.07,1.54) |  | 3.46 (1.85-6.85) | 5.57 (2.99-9.46) | 0.91 (0.49-1.81) | 1.21 (0.65-2.05) | 1.33 (1.09,1.57) |
| Finland | 0.33 (0.29-0.38) | 0.30 (0.23-0.38) | 0.01 (0.01-0.02) | 0.01 (0.01-0.01) | -0.31 (-0.94,0.32) |  | 0.15 (0.13-0.17) | 0.07 (0.05-0.08) | 0.01 (0.01-0.01) | 0.00 (0.00-0.00) | -2.59 (-2.95,-2.24) |  | 13.11 (11.54-15.12) | 5.94 (4.62-7.52) | 0.52 (0.46-0.60) | 0.21 (0.17-0.27) | -2.54 (-2.89,-2.18) |
| France | 9.98 (8.79-11.34) | 15.31 (12.25-18.96) | 0.03 (0.03-0.04) | 0.05 (0.04-0.06) | 1.32 (1.08,1.56) |  | 4.14 (3.65-4.70) | 3.30 (2.67-4.01) | 0.01 (0.01-0.02) | 0.01 (0.01-0.01) | -0.82 (-1.13,-0.52) |  | 368.29 (323.86-416.36) | 296.04 (239.06-359.85) | 1.27 (1.12-1.44) | 0.89 (0.72-1.08) | -0.78 (-1.08,-0.48) |
| Gabon | 0.80 (0.42-1.47) | 0.59 (0.27-1.03) | 0.16 (0.09-0.30) | 0.06 (0.03-0.11) | -2.42 (-2.78,-2.05) |  | 0.57 (0.30-1.00) | 0.41 (0.19-0.72) | 0.12 (0.06-0.20) | 0.04 (0.02-0.08) | -2.50 (-2.86,-2.13) |  | 50.02 (26.22-88.95) | 35.56 (16.39-63.17) | 10.17 (5.33-18.09) | 3.92 (1.81-6.96) | -2.51 (-2.88,-2.14) |
| Gambia | 4.83 (3.25-7.02) | 5.05 (3.29-7.54) | 0.98 (0.66-1.43) | 0.42 (0.27-0.63) | -2.91 (-3.20,-2.63) |  | 3.39 (2.27-4.98) | 3.49 (2.27-5.20) | 0.69 (0.46-1.01) | 0.29 (0.19-0.43) | -2.96 (-3.25,-2.68) |  | 300.16 (201.08-441.04) | 308.83 (200.22-461.24) | 61.16 (40.97-89.86) | 25.80 (16.73-38.53) | -2.97 (-3.25,-2.68) |
| Georgia | 0.85 (0.70-1.02) | 0.11 (0.08-0.14) | 0.03 (0.03-0.04) | 0.01 (0.00-0.01) | -5.47 (-6.27,-4.67) |  | 0.56 (0.46-0.66) | 0.07 (0.05-0.09) | 0.02 (0.02-0.02) | 0.00 (0.00-0.00) | -5.60 (-6.39,-4.80) |  | 49.03 (40.36-57.97) | 5.90 (4.43-7.70) | 1.78 (1.46-2.10) | 0.33 (0.25-0.43) | -5.59 (-6.38,-4.79) |
| Germany | 7.75 (6.57-9.28) | 9.45 (7.59-11.62) | 0.02 (0.02-0.02) | 0.02 (0.02-0.03) | 0.20 (-0.16,0.57) |  | 3.37 (2.84-4.06) | 2.08 (1.66-2.61) | 0.01 (0.01-0.01) | 0.00 (0.00-0.01) | -1.57 (-1.94,-1.20) |  | 298.55 (252.05-359.26) | 186.98 (148.80-232.83) | 0.75 (0.63-0.90) | 0.44 (0.35-0.55) | -1.53 (-1.90,-1.16) |
| Ghana | 15.82 (9.93-24.84) | 9.58 (5.56-16.98) | 0.21 (0.13-0.33) | 0.06 (0.03-0.10) | -4.35 (-4.60,-4.09) |  | 11.05 (6.88-17.20) | 6.54 (3.74-11.68) | 0.15 (0.09-0.23) | 0.04 (0.02-0.07) | -4.41 (-4.67,-4.15) |  | 982.98 (612.87-1518.86) | 582.29 (332.87-1036.96) | 13.13 (8.19-20.29) | 3.40 (1.94-6.06) | -4.41 (-4.66,-4.15) |
| Greece | 1.78 (1.63-1.95) | 2.10 (1.73-2.53) | 0.03 (0.03-0.04) | 0.04 (0.03-0.05) | 0.98 (0.71,1.25) |  | 0.56 (0.51-0.60) | 0.47 (0.39-0.56) | 0.01 (0.01-0.01) | 0.01 (0.01-0.01) | 0.14 (-0.21,0.49) |  | 49.28 (45.30-53.73) | 42.49 (35.08-50.50) | 0.95 (0.87-1.03) | 0.84 (0.69-0.99) | 0.17 (-0.17,0.51) |
| Greenland | 0.02 (0.02-0.03) | 0.01 (0.01-0.02) | 0.08 (0.06-0.11) | 0.04 (0.02-0.06) | -2.21 (-2.62,-1.80) |  | 0.01 (0.01-0.02) | 0.01 (0.00-0.01) | 0.05 (0.04-0.07) | 0.02 (0.01-0.03) | -2.70 (-3.11,-2.29) |  | 1.27 (0.91-1.78) | 0.51 (0.31-0.82) | 4.57 (3.29-6.41) | 1.83 (1.09-2.91) | -2.73 (-3.13,-2.33) |
| Grenada | 0.01 (0.01-0.02) | 0.01 (0.00-0.01) | 0.03 (0.02-0.04) | 0.01 (0.01-0.01) | -2.95 (-3.22,-2.67) |  | 0.01 (0.01-0.01) | 0.00 (0.00-0.00) | 0.02 (0.01-0.03) | 0.01 (0.01-0.01) | -3.07 (-3.34,-2.80) |  | 0.70 (0.49-1.00) | 0.30 (0.23-0.38) | 1.62 (1.12-2.30) | 0.58 (0.44-0.74) | -3.08 (-3.35,-2.81) |
| Guam | 0.01 (0.01-0.01) | 0.02 (0.01-0.03) | 0.01 (0.01-0.01) | 0.02 (0.02-0.03) | 3.64 (3.09,4.20) |  | 0.00 (0.00-0.01) | 0.01 (0.01-0.02) | 0.01 (0.00-0.01) | 0.01 (0.01-0.02) | 3.32 (2.84,3.80) |  | 0.39 (0.29-0.55) | 0.93 (0.64-1.34) | 0.57 (0.43-0.80) | 1.16 (0.80-1.68) | 3.34 (2.86,3.82) |
| Guatemala | 15.93 (14.27-17.78) | 4.48 (3.44-5.76) | 0.38 (0.34-0.42) | 0.06 (0.04-0.07) | -5.54 (-6.02,-5.06) |  | 11.36 (10.19-12.63) | 3.09 (2.41-3.94) | 0.27 (0.24-0.30) | 0.04 (0.03-0.05) | -5.64 (-6.13,-5.15) |  | 996.81 (894.34-1110.25) | 269.91 (209.58-344.55) | 23.78 (21.33-26.48) | 3.42 (2.66-4.37) | -5.66 (-6.14,-5.17) |
| Guinea | 40.48 (17.30-62.81) | 29.72 (16.81-45.98) | 1.35 (0.58-2.10) | 0.44 (0.25-0.68) | -2.94 (-3.22,-2.66) |  | 28.58 (12.33-44.99) | 20.76 (11.70-31.88) | 0.95 (0.41-1.50) | 0.31 (0.17-0.47) | -2.98 (-3.26,-2.69) |  | 2547.54 (1103.34-4016.16) | 1851.11 (1046.77-2847.37) | 85.00 (36.81-134.01) | 27.57 (15.59-42.40) | -2.97 (-3.26,-2.69) |
| Guinea-Bissau | 2.55 (1.28-4.21) | 1.39 (0.78-2.14) | 0.51 (0.26-0.84) | 0.13 (0.08-0.21) | -4.04 (-4.49,-3.58) |  | 1.81 (0.91-2.99) | 0.97 (0.55-1.46) | 0.36 (0.18-0.59) | 0.09 (0.05-0.14) | -4.08 (-4.55,-3.62) |  | 160.72 (80.70-263.93) | 86.44 (48.57-130.07) | 31.92 (16.03-52.41) | 8.38 (4.71-12.60) | -4.07 (-4.53,-3.60) |
| Guyana | 0.13 (0.10-0.16) | 0.05 (0.03-0.06) | 0.03 (0.03-0.04) | 0.01 (0.01-0.02) | -2.51 (-2.83,-2.18) |  | 0.09 (0.07-0.11) | 0.03 (0.02-0.04) | 0.02 (0.02-0.03) | 0.01 (0.01-0.01) | -2.54 (-2.88,-2.19) |  | 7.87 (6.21-9.80) | 2.83 (2.07-3.67) | 2.02 (1.59-2.51) | 0.74 (0.54-0.96) | -2.56 (-2.89,-2.23) |
| Haiti | 2.18 (0.81-4.38) | 1.88 (0.93-3.52) | 0.07 (0.03-0.14) | 0.03 (0.01-0.05) | -2.43 (-2.66,-2.19) |  | 1.57 (0.58-3.12) | 1.34 (0.67-2.52) | 0.05 (0.02-0.10) | 0.02 (0.01-0.04) | -2.46 (-2.70,-2.23) |  | 138.29 (51.25-274.53) | 117.58 (58.56-222.77) | 4.33 (1.61-8.60) | 1.83 (0.91-3.46) | -2.47 (-2.70,-2.23) |
| Honduras | 2.27 (1.56-3.29) | 1.10 (0.62-1.84) | 0.10 (0.07-0.14) | 0.02 (0.01-0.04) | -5.01 (-5.23,-4.79) |  | 1.62 (1.11-2.34) | 0.76 (0.44-1.28) | 0.07 (0.05-0.10) | 0.02 (0.01-0.03) | -5.10 (-5.32,-4.87) |  | 142.14 (97.33-203.96) | 66.81 (38.28-112.27) | 6.04 (4.13-8.66) | 1.32 (0.76-2.22) | -5.10 (-5.32,-4.87) |
| Hungary | 0.70 (0.57-0.87) | 0.24 (0.18-0.32) | 0.01 (0.01-0.02) | 0.01 (0.00-0.01) | -3.15 (-3.79,-2.51) |  | 0.44 (0.36-0.54) | 0.08 (0.06-0.10) | 0.01 (0.01-0.01) | 0.00 (0.00-0.00) | -5.30 (-5.96,-4.63) |  | 38.95 (31.77-47.74) | 6.90 (5.08-8.99) | 0.75 (0.61-0.92) | 0.14 (0.11-0.19) | -5.28 (-5.94,-4.61) |
| Iceland | 0.04 (0.03-0.04) | 0.04 (0.03-0.05) | 0.03 (0.02-0.03) | 0.02 (0.02-0.03) | -1.40 (-1.60,-1.19) |  | 0.01 (0.01-0.01) | 0.01 (0.01-0.01) | 0.01 (0.01-0.01) | 0.00 (0.00-0.01) | -1.90 (-2.10,-1.71) |  | 0.90 (0.75-1.07) | 0.75 (0.59-0.98) | 0.71 (0.59-0.84) | 0.43 (0.34-0.56) | -1.90 (-2.10,-1.70) |
| India | 376.11 (237.70-508.75) | 201.58 (145.19-276.91) | 0.09 (0.06-0.12) | 0.03 (0.02-0.04) | -3.64 (-3.78,-3.50) |  | 266.51 (167.49-359.59) | 137.06 (99.17-189.64) | 0.06 (0.04-0.08) | 0.02 (0.01-0.03) | -3.78 (-3.94,-3.63) |  | 23467.36 (14771.13-31680.70) | 12120.56 (8761.17-16813.58) | 5.50 (3.46-7.43) | 1.71 (1.24-2.38) | -3.76 (-3.92,-3.61) |
| Indonesia | 58.76 (19.40-99.09) | 33.98 (15.43-59.54) | 0.06 (0.02-0.11) | 0.02 (0.01-0.04) | -2.82 (-2.94,-2.71) |  | 41.62 (13.82-70.37) | 23.23 (10.70-40.45) | 0.04 (0.01-0.08) | 0.02 (0.01-0.03) | -2.93 (-3.04,-2.82) |  | 3656.03 (1210.67-6187.39) | 2038.57 (934.04-3552.38) | 3.95 (1.31-6.69) | 1.46 (0.67-2.55) | -2.93 (-3.05,-2.82) |
| Iran (Islamic Republic of) | 39.43 (29.40-50.67) | 13.87 (9.42-22.39) | 0.14 (0.10-0.18) | 0.03 (0.02-0.05) | -3.76 (-4.47,-3.04) |  | 27.16 (20.31-34.70) | 7.54 (5.25-12.06) | 0.10 (0.07-0.12) | 0.02 (0.01-0.03) | -4.57 (-5.23,-3.91) |  | 2385.35 (1780.20-3049.94) | 655.77 (455.66-1053.06) | 8.35 (6.24-10.68) | 1.54 (1.07-2.47) | -4.57 (-5.23,-3.91) |
| Iraq | 12.36 (8.04-17.06) | 8.23 (5.15-13.14) | 0.13 (0.09-0.19) | 0.04 (0.02-0.06) | -3.22 (-3.48,-2.95) |  | 8.61 (5.68-11.93) | 4.98 (3.07-7.98) | 0.09 (0.06-0.13) | 0.02 (0.01-0.04) | -3.64 (-3.93,-3.34) |  | 757.05 (497.56-1044.90) | 436.05 (270.27-702.12) | 8.22 (5.40-11.35) | 2.12 (1.31-3.41) | -3.66 (-3.95,-3.37) |
| Ireland | 0.31 (0.26-0.36) | 0.36 (0.27-0.44) | 0.02 (0.01-0.02) | 0.01 (0.01-0.02) | 0.20 (-0.38,0.78) |  | 0.13 (0.11-0.15) | 0.08 (0.07-0.10) | 0.01 (0.01-0.01) | 0.00 (0.00-0.00) | -1.76 (-2.06,-1.46) |  | 11.25 (9.68-13.06) | 7.38 (5.82-8.97) | 0.62 (0.54-0.73) | 0.30 (0.24-0.36) | -1.73 (-2.03,-1.42) |
| Israel | 0.68 (0.58-0.78) | 1.20 (0.94-1.49) | 0.03 (0.02-0.03) | 0.03 (0.02-0.03) | 0.59 (0.13,1.04) |  | 0.34 (0.29-0.39) | 0.27 (0.22-0.34) | 0.01 (0.01-0.02) | 0.01 (0.00-0.01) | -2.11 (-2.37,-1.85) |  | 29.75 (25.43-34.42) | 24.62 (19.39-30.41) | 1.20 (1.03-1.39) | 0.51 (0.40-0.63) | -2.06 (-2.32,-1.80) |
| Italy | 10.67 (10.07-11.36) | 4.36 (3.50-5.25) | 0.04 (0.04-0.04) | 0.01 (0.01-0.02) | -3.30 (-3.85,-2.74) |  | 3.69 (3.50-3.90) | 0.96 (0.78-1.13) | 0.01 (0.01-0.01) | 0.00 (0.00-0.00) | -4.47 (-4.94,-3.99) |  | 328.01 (311.22-346.93) | 85.74 (70.27-101.73) | 1.15 (1.10-1.22) | 0.29 (0.23-0.34) | -4.44 (-4.92,-3.97) |
| Jamaica | 0.24 (0.17-0.31) | 0.09 (0.06-0.12) | 0.02 (0.01-0.03) | 0.01 (0.00-0.01) | -3.85 (-4.11,-3.58) |  | 0.16 (0.12-0.21) | 0.06 (0.04-0.07) | 0.01 (0.01-0.02) | 0.00 (0.00-0.01) | -4.06 (-4.34,-3.79) |  | 13.96 (10.16-18.07) | 4.81 (3.45-6.53) | 1.18 (0.86-1.53) | 0.34 (0.25-0.47) | -4.07 (-4.34,-3.79) |
| Japan | 29.25 (28.12-30.66) | 13.28 (11.69-14.73) | 0.05 (0.04-0.05) | 0.02 (0.02-0.02) | -3.11 (-3.83,-2.39) |  | 9.77 (9.41-10.23) | 2.93 (2.62-3.21) | 0.02 (0.01-0.02) | 0.00 (0.00-0.01) | -4.30 (-4.79,-3.80) |  | 866.36 (833.51-905.71) | 262.10 (234.31-288.07) | 1.38 (1.32-1.44) | 0.41 (0.37-0.45) | -4.26 (-4.76,-3.76) |
| Jordan | 0.78 (0.49-1.33) | 0.99 (0.66-1.54) | 0.04 (0.03-0.07) | 0.02 (0.01-0.02) | -3.05 (-3.25,-2.85) |  | 0.53 (0.33-0.90) | 0.52 (0.35-0.81) | 0.03 (0.02-0.05) | 0.01 (0.01-0.01) | -4.09 (-4.34,-3.84) |  | 47.00 (29.15-78.62) | 45.75 (30.56-71.84) | 2.52 (1.56-4.21) | 0.74 (0.50-1.17) | -4.10 (-4.34,-3.85) |
| Kazakhstan | 8.02 (7.01-9.16) | 2.40 (1.85-3.05) | 0.10 (0.09-0.11) | 0.03 (0.02-0.03) | -4.32 (-4.79,-3.85) |  | 5.46 (4.78-6.23) | 1.44 (1.11-1.82) | 0.07 (0.06-0.08) | 0.02 (0.01-0.02) | -4.82 (-5.22,-4.42) |  | 479.96 (419.97-549.02) | 125.91 (97.06-159.67) | 5.86 (5.12-6.70) | 1.33 (1.02-1.68) | -4.81 (-5.21,-4.41) |
| Kenya | 17.45 (10.55-24.12) | 14.88 (10.61-21.09) | 0.15 (0.09-0.21) | 0.06 (0.04-0.08) | -2.31 (-2.63,-1.98) |  | 12.20 (7.37-16.87) | 10.31 (7.36-14.71) | 0.11 (0.06-0.15) | 0.04 (0.03-0.06) | -2.32 (-2.65,-1.98) |  | 1081.28 (652.55-1497.55) | 908.80 (648.67-1295.84) | 9.34 (5.64-12.94) | 3.63 (2.59-5.18) | -2.34 (-2.67,-2.01) |
| Kiribati | 0.03 (0.02-0.05) | 0.02 (0.01-0.04) | 0.08 (0.04-0.14) | 0.03 (0.01-0.07) | -3.86 (-4.24,-3.48) |  | 0.02 (0.01-0.04) | 0.01 (0.01-0.03) | 0.06 (0.03-0.10) | 0.02 (0.01-0.05) | -3.87 (-4.25,-3.49) |  | 1.84 (1.02-3.18) | 1.09 (0.49-2.46) | 4.94 (2.74-8.56) | 1.80 (0.81-4.06) | -3.89 (-4.27,-3.51) |
| Kuwait | 0.65 (0.53-0.80) | 0.15 (0.12-0.20) | 0.08 (0.06-0.09) | 0.01 (0.01-0.01) | -4.70 (-5.91,-3.48) |  | 0.37 (0.30-0.45) | 0.05 (0.04-0.07) | 0.04 (0.04-0.05) | 0.00 (0.00-0.00) | -7.00 (-8.09,-5.90) |  | 32.57 (26.59-39.98) | 4.40 (3.40-5.68) | 3.79 (3.09-4.65) | 0.19 (0.15-0.24) | -6.98 (-8.07,-5.88) |
| Kyrgyzstan | 4.33 (3.14-5.97) | 0.47 (0.35-0.63) | 0.19 (0.14-0.27) | 0.01 (0.01-0.02) | -6.41 (-7.83,-4.97) |  | 2.94 (2.14-4.00) | 0.30 (0.23-0.40) | 0.13 (0.10-0.18) | 0.01 (0.01-0.01) | -6.67 (-8.04,-5.28) |  | 260.92 (189.68-356.39) | 26.48 (19.82-34.89) | 11.69 (8.50-15.97) | 0.77 (0.58-1.02) | -6.68 (-8.05,-5.28) |
| Lao People's Democratic Republic | 5.66 (1.70-9.78) | 2.20 (1.32-3.39) | 0.27 (0.08-0.47) | 0.06 (0.04-0.09) | -5.27 (-5.42,-5.12) |  | 4.06 (1.22-7.03) | 1.54 (0.94-2.34) | 0.19 (0.06-0.34) | 0.04 (0.03-0.06) | -5.35 (-5.50,-5.20) |  | 357.65 (107.19-619.48) | 135.27 (82.01-205.05) | 17.16 (5.14-29.71) | 3.67 (2.22-5.56) | -5.35 (-5.51,-5.20) |
| Latvia | 0.28 (0.23-0.32) | 0.23 (0.17-0.31) | 0.02 (0.02-0.02) | 0.02 (0.02-0.03) | 0.86 (0.23,1.50) |  | 0.17 (0.15-0.20) | 0.10 (0.08-0.13) | 0.01 (0.01-0.02) | 0.01 (0.01-0.01) | -0.27 (-0.85,0.32) |  | 15.06 (12.74-17.63) | 8.81 (6.67-11.62) | 1.13 (0.96-1.33) | 0.94 (0.71-1.24) | -0.26 (-0.85,0.32) |
| Lebanon | 0.81 (0.53-1.20) | 0.46 (0.24-0.91) | 0.05 (0.04-0.08) | 0.02 (0.01-0.03) | -3.98 (-4.19,-3.77) |  | 0.54 (0.35-0.80) | 0.19 (0.10-0.37) | 0.04 (0.02-0.05) | 0.01 (0.00-0.01) | -5.64 (-5.94,-5.35) |  | 47.57 (30.72-70.86) | 16.59 (8.64-32.79) | 3.18 (2.05-4.74) | 0.60 (0.31-1.18) | -5.65 (-5.94,-5.35) |
| Lesotho | 0.29 (0.12-0.58) | 0.43 (0.17-1.18) | 0.04 (0.02-0.08) | 0.05 (0.02-0.13) | 0.61 (0.21,1.01) |  | 0.21 (0.08-0.42) | 0.31 (0.12-0.89) | 0.03 (0.01-0.06) | 0.03 (0.01-0.09) | 0.66 (0.25,1.08) |  | 18.22 (7.43-37.41) | 27.21 (10.47-77.66) | 2.38 (0.97-4.88) | 2.90 (1.12-8.29) | 0.63 (0.22,1.04) |
| Liberia | 8.89 (3.37-14.88) | 4.40 (2.25-7.41) | 0.72 (0.27-1.21) | 0.16 (0.08-0.27) | -5.12 (-5.52,-4.72) |  | 6.27 (2.38-10.51) | 3.01 (1.55-5.06) | 0.51 (0.19-0.85) | 0.11 (0.06-0.19) | -5.21 (-5.61,-4.81) |  | 559.37 (211.47-937.61) | 269.23 (138.48-450.94) | 45.47 (17.19-76.21) | 9.86 (5.07-16.52) | -5.21 (-5.61,-4.81) |
| Libya | 3.07 (2.05-4.34) | 3.22 (1.78-4.85) | 0.15 (0.10-0.21) | 0.09 (0.05-0.14) | -0.51 (-0.90,-0.11) |  | 2.07 (1.38-2.94) | 1.84 (1.04-2.76) | 0.10 (0.07-0.14) | 0.05 (0.03-0.08) | -1.11 (-1.49,-0.74) |  | 182.62 (122.32-259.79) | 162.73 (91.71-243.19) | 8.67 (5.80-12.33) | 4.74 (2.67-7.08) | -1.11 (-1.49,-0.73) |
| Lithuania | 0.33 (0.28-0.37) | 0.29 (0.23-0.37) | 0.02 (0.02-0.02) | 0.02 (0.02-0.03) | 0.00 (-0.87,0.89) |  | 0.19 (0.17-0.22) | 0.13 (0.10-0.17) | 0.01 (0.01-0.01) | 0.01 (0.01-0.01) | -0.80 (-1.63,0.04) |  | 17.18 (14.93-19.70) | 11.76 (9.07-15.25) | 0.94 (0.81-1.07) | 0.86 (0.66-1.12) | -0.80 (-1.63,0.04) |
| Luxembourg | 0.04 (0.04-0.05) | 0.07 (0.05-0.08) | 0.02 (0.02-0.02) | 0.02 (0.02-0.03) | -0.30 (-0.76,0.16) |  | 0.02 (0.02-0.02) | 0.01 (0.01-0.02) | 0.01 (0.01-0.01) | 0.00 (0.00-0.01) | -2.42 (-2.85,-1.99) |  | 1.65 (1.46-1.85) | 1.27 (1.04-1.56) | 0.87 (0.77-0.97) | 0.40 (0.32-0.48) | -2.38 (-2.81,-1.95) |
| Madagascar | 10.64 (6.41-16.88) | 7.89 (3.93-15.25) | 0.18 (0.11-0.28) | 0.06 (0.03-0.11) | -3.32 (-3.51,-3.13) |  | 7.53 (4.58-12.05) | 5.55 (2.78-10.86) | 0.13 (0.08-0.20) | 0.04 (0.02-0.08) | -3.35 (-3.54,-3.15) |  | 664.43 (403.95-1055.62) | 488.57 (243.70-955.28) | 11.17 (6.79-17.74) | 3.42 (1.71-6.69) | -3.35 (-3.54,-3.16) |
| Malawi | 33.83 (21.06-50.49) | 19.41 (8.46-44.71) | 0.69 (0.43-1.03) | 0.20 (0.09-0.46) | -4.18 (-4.71,-3.63) |  | 23.84 (14.97-35.10) | 13.57 (5.90-31.21) | 0.49 (0.31-0.72) | 0.14 (0.06-0.32) | -4.20 (-4.74,-3.66) |  | 2112.32 (1328.45-3105.05) | 1197.31 (520.72-2762.62) | 43.08 (27.10-63.33) | 12.31 (5.36-28.41) | -4.21 (-4.75,-3.67) |
| Malaysia | 3.69 (2.18-5.86) | 2.72 (1.77-3.94) | 0.04 (0.02-0.07) | 0.02 (0.01-0.02) | -2.79 (-3.33,-2.24) |  | 2.56 (1.50-4.11) | 1.69 (1.09-2.46) | 0.03 (0.02-0.05) | 0.01 (0.01-0.02) | -3.16 (-3.67,-2.64) |  | 225.52 (132.32-360.24) | 148.59 (95.82-214.85) | 2.55 (1.50-4.08) | 0.93 (0.60-1.35) | -3.16 (-3.68,-2.64) |
| Maldives | 0.11 (0.04-0.19) | 0.04 (0.02-0.06) | 0.10 (0.03-0.17) | 0.01 (0.01-0.02) | -5.50 (-5.96,-5.05) |  | 0.08 (0.03-0.13) | 0.02 (0.01-0.03) | 0.07 (0.02-0.12) | 0.01 (0.00-0.01) | -6.34 (-6.74,-5.94) |  | 6.62 (2.27-11.46) | 1.75 (1.06-2.74) | 5.96 (2.04-10.32) | 0.68 (0.41-1.06) | -6.32 (-6.72,-5.92) |
| Mali | 50.40 (23.12-76.60) | 84.75 (49.34-126.26) | 1.16 (0.53-1.77) | 0.70 (0.41-1.05) | -1.50 (-1.69,-1.31) |  | 35.48 (16.14-53.55) | 58.80 (34.60-88.33) | 0.82 (0.37-1.24) | 0.49 (0.29-0.73) | -1.55 (-1.74,-1.35) |  | 3158.25 (1439.97-4766.01) | 5242.15 (3082.27-7852.28) | 72.91 (33.24-110.03) | 43.50 (25.57-65.15) | -1.54 (-1.73,-1.35) |
| Malta | 0.03 (0.02-0.03) | 0.04 (0.03-0.05) | 0.01 (0.01-0.02) | 0.02 (0.01-0.02) | 1.12 (0.90,1.34) |  | 0.01 (0.01-0.01) | 0.01 (0.01-0.01) | 0.01 (0.00-0.01) | 0.00 (0.00-0.01) | -1.17 (-1.55,-0.79) |  | 1.00 (0.79-1.20) | 0.77 (0.57-1.00) | 0.54 (0.42-0.65) | 0.35 (0.26-0.45) | -1.11 (-1.49,-0.73) |
| Marshall Islands | 0.00 (0.00-0.01) | 0.00 (0.00-0.01) | 0.01 (0.01-0.03) | 0.01 (0.00-0.02) | -1.41 (-2.01,-0.80) |  | 0.00 (0.00-0.00) | 0.00 (0.00-0.00) | 0.01 (0.00-0.02) | 0.01 (0.00-0.01) | -1.44 (-2.04,-0.84) |  | 0.17 (0.07-0.39) | 0.15 (0.06-0.33) | 0.75 (0.32-1.70) | 0.52 (0.21-1.19) | -1.45 (-2.05,-0.85) |
| Mauritania | 4.71 (1.26-11.00) | 3.69 (1.79-6.01) | 0.46 (0.12-1.07) | 0.17 (0.08-0.27) | -3.33 (-3.85,-2.81) |  | 3.31 (0.88-7.66) | 2.47 (1.21-4.01) | 0.32 (0.09-0.75) | 0.11 (0.06-0.18) | -3.47 (-4.00,-2.94) |  | 294.16 (78.62-684.47) | 220.16 (108.05-357.38) | 28.63 (7.65-66.63) | 10.02 (4.92-16.26) | -3.46 (-3.99,-2.93) |
| Mauritius | 0.12 (0.11-0.13) | 0.01 (0.01-0.01) | 0.02 (0.02-0.02) | 0.00 (0.00-0.00) | -4.25 (-6.09,-2.37) |  | 0.08 (0.07-0.09) | 0.00 (0.00-0.00) | 0.01 (0.01-0.02) | 0.00 (0.00-0.00) | -4.54 (-6.38,-2.66) |  | 7.17 (6.51-7.96) | 0.38 (0.32-0.44) | 1.31 (1.19-1.45) | 0.06 (0.05-0.07) | -4.54 (-6.37,-2.67) |
| Mexico | 43.61 (39.32-49.24) | 23.08 (17.29-30.95) | 0.10 (0.09-0.12) | 0.04 (0.03-0.05) | -2.86 (-3.13,-2.59) |  | 30.32 (27.35-34.19) | 14.83 (11.21-19.71) | 0.07 (0.06-0.08) | 0.02 (0.02-0.03) | -3.10 (-3.36,-2.83) |  | 2671.17 (2404.61-3014.96) | 1298.51 (978.50-1728.02) | 6.26 (5.63-7.06) | 2.01 (1.51-2.67) | -3.12 (-3.39,-2.85) |
| Micronesia (Federated States of) | 0.01 (0.01-0.02) | 0.00 (0.00-0.01) | 0.03 (0.01-0.05) | 0.01 (0.00-0.02) | -3.78 (-4.10,-3.45) |  | 0.01 (0.01-0.02) | 0.00 (0.00-0.01) | 0.02 (0.01-0.03) | 0.01 (0.00-0.01) | -3.85 (-4.18,-3.52) |  | 0.87 (0.46-1.53) | 0.26 (0.12-0.58) | 1.68 (0.89-2.96) | 0.51 (0.23-1.12) | -3.86 (-4.19,-3.53) |
| Monaco | 0.01 (0.00-0.01) | 0.00 (0.00-0.01) | 0.04 (0.02-0.06) | 0.02 (0.02-0.04) | -1.55 (-1.90,-1.21) |  | 0.00 (0.00-0.00) | 0.00 (0.00-0.00) | 0.01 (0.01-0.02) | 0.01 (0.00-0.01) | -2.17 (-2.46,-1.89) |  | 0.13 (0.08-0.22) | 0.09 (0.06-0.14) | 0.88 (0.51-1.47) | 0.48 (0.31-0.73) | -2.16 (-2.44,-1.88) |
| Mongolia | 20.92 (12.62-32.82) | 5.22 (3.28-7.87) | 1.94 (1.17-3.04) | 0.31 (0.20-0.47) | -5.49 (-6.07,-4.90) |  | 14.73 (8.90-22.93) | 3.54 (2.24-5.33) | 1.37 (0.83-2.13) | 0.21 (0.13-0.32) | -5.66 (-6.23,-5.08) |  | 1294.56 (779.48-2019.16) | 309.08 (195.39-466.19) | 119.99 (72.25-187.15) | 18.53 (11.71-27.95) | -5.66 (-6.23,-5.09) |
| Montenegro | 0.19 (0.13-0.27) | 0.06 (0.03-0.10) | 0.06 (0.04-0.09) | 0.02 (0.01-0.03) | -2.81 (-3.18,-2.44) |  | 0.10 (0.06-0.13) | 0.02 (0.01-0.03) | 0.03 (0.02-0.04) | 0.01 (0.00-0.01) | -4.71 (-4.99,-4.43) |  | 8.34 (5.59-11.65) | 1.71 (0.93-2.75) | 2.66 (1.78-3.72) | 0.55 (0.30-0.89) | -4.68 (-4.95,-4.40) |
| Morocco | 2.46 (1.54-3.76) | 1.06 (0.58-1.89) | 0.02 (0.01-0.03) | 0.01 (0.00-0.01) | -3.46 (-3.65,-3.27) |  | 1.72 (1.08-2.65) | 0.70 (0.38-1.25) | 0.01 (0.01-0.02) | 0.00 (0.00-0.01) | -3.63 (-3.83,-3.43) |  | 152.46 (95.53-235.05) | 61.83 (33.17-110.22) | 1.20 (0.75-1.85) | 0.33 (0.18-0.59) | -3.65 (-3.84,-3.45) |
| Mozambique | 16.96 (9.34-26.09) | 13.39 (5.45-34.84) | 0.25 (0.14-0.39) | 0.09 (0.04-0.22) | -3.47 (-3.72,-3.22) |  | 12.03 (6.74-18.32) | 9.53 (3.87-25.00) | 0.18 (0.10-0.27) | 0.06 (0.02-0.16) | -3.45 (-3.70,-3.20) |  | 1062.31 (597.50-1619.68) | 839.00 (340.63-2202.73) | 15.90 (8.94-24.25) | 5.40 (2.19-14.18) | -3.46 (-3.71,-3.21) |
| Myanmar | 19.06 (3.41-40.86) | 9.38 (3.56-18.60) | 0.09 (0.02-0.20) | 0.03 (0.01-0.07) | -3.42 (-3.53,-3.32) |  | 13.56 (2.44-28.63) | 6.46 (2.49-12.70) | 0.07 (0.01-0.14) | 0.02 (0.01-0.05) | -3.54 (-3.65,-3.42) |  | 1193.66 (213.47-2520.02) | 568.66 (218.82-1118.87) | 5.90 (1.06-12.46) | 2.02 (0.78-3.97) | -3.53 (-3.65,-3.42) |
| Namibia | 0.42 (0.24-0.66) | 0.68 (0.42-1.06) | 0.06 (0.03-0.09) | 0.06 (0.03-0.09) | 0.33 (0.03,0.64) |  | 0.30 (0.16-0.46) | 0.46 (0.29-0.72) | 0.04 (0.02-0.07) | 0.04 (0.02-0.06) | 0.23 (-0.08,0.55) |  | 26.10 (14.61-40.63) | 40.79 (25.50-63.32) | 3.72 (2.08-5.79) | 3.36 (2.10-5.21) | 0.22 (-0.09,0.54) |
| Nauru | 0.00 (0.00-0.00) | 0.00 (0.00-0.00) | 0.05 (0.03-0.09) | 0.03 (0.02-0.06) | -1.77 (-2.19,-1.34) |  | 0.00 (0.00-0.00) | 0.00 (0.00-0.00) | 0.04 (0.02-0.06) | 0.02 (0.01-0.04) | -1.81 (-2.25,-1.37) |  | 0.17 (0.10-0.28) | 0.12 (0.06-0.20) | 3.25 (1.94-5.49) | 2.09 (1.12-3.70) | -1.82 (-2.26,-1.37) |
| Nepal | 11.44 (4.05-19.05) | 5.93 (3.62-8.73) | 0.12 (0.04-0.20) | 0.04 (0.02-0.06) | -3.42 (-3.54,-3.30) |  | 8.10 (2.85-13.42) | 4.06 (2.46-6.01) | 0.08 (0.03-0.14) | 0.03 (0.02-0.04) | -3.52 (-3.64,-3.40) |  | 715.50 (252.45-1185.43) | 358.38 (217.95-532.63) | 7.35 (2.59-12.18) | 2.30 (1.40-3.42) | -3.53 (-3.65,-3.41) |
| Netherlands | 1.27 (1.11-1.43) | 1.08 (0.90-1.29) | 0.02 (0.01-0.02) | 0.01 (0.01-0.02) | -1.21 (-1.50,-0.92) |  | 0.36 (0.32-0.40) | 0.23 (0.20-0.27) | 0.00 (0.00-0.01) | 0.00 (0.00-0.00) | -2.14 (-2.39,-1.90) |  | 32.21 (28.63-36.19) | 20.88 (17.59-24.74) | 0.43 (0.38-0.49) | 0.24 (0.20-0.29) | -2.12 (-2.36,-1.87) |
| New Zealand | 0.54 (0.45-0.65) | 0.85 (0.66-1.09) | 0.03 (0.03-0.04) | 0.03 (0.03-0.04) | 0.84 (0.49,1.19) |  | 0.25 (0.20-0.30) | 0.26 (0.20-0.35) | 0.01 (0.01-0.02) | 0.01 (0.01-0.01) | -0.42 (-0.72,-0.12) |  | 21.55 (17.73-26.05) | 23.01 (17.63-31.09) | 1.26 (1.04-1.52) | 0.89 (0.68-1.20) | -0.39 (-0.70,-0.09) |
| Nicaragua | 3.95 (3.04-5.12) | 1.05 (0.68-1.59) | 0.20 (0.16-0.26) | 0.03 (0.02-0.05) | -5.73 (-6.16,-5.30) |  | 2.74 (2.10-3.58) | 0.68 (0.45-1.02) | 0.14 (0.11-0.18) | 0.02 (0.01-0.03) | -5.93 (-6.34,-5.51) |  | 241.76 (185.18-314.95) | 59.76 (39.33-89.50) | 12.44 (9.53-16.20) | 1.79 (1.18-2.68) | -5.95 (-6.36,-5.53) |
| Niger | 26.61 (16.34-41.96) | 21.27 (12.72-35.09) | 0.66 (0.41-1.04) | 0.17 (0.10-0.28) | -4.53 (-4.78,-4.28) |  | 18.85 (11.60-29.61) | 14.85 (8.88-24.25) | 0.47 (0.29-0.74) | 0.12 (0.07-0.19) | -4.58 (-4.84,-4.33) |  | 1674.85 (1030.48-2629.74) | 1322.41 (788.85-2162.54) | 41.70 (25.66-65.48) | 10.56 (6.30-17.27) | -4.58 (-4.83,-4.32) |
| Nigeria | 81.27 (50.90-110.66) | 110.11 (70.98-149.06) | 0.18 (0.11-0.25) | 0.10 (0.06-0.13) | -1.99 (-2.21,-1.77) |  | 57.22 (35.77-77.70) | 76.09 (49.25-103.44) | 0.13 (0.08-0.17) | 0.07 (0.04-0.09) | -2.06 (-2.28,-1.84) |  | 5090.68 (3174.98-6909.21) | 6775.31 (4393.87-9187.24) | 11.31 (7.05-15.35) | 5.86 (3.80-7.95) | -2.05 (-2.28,-1.83) |
| Niue | 0.00 (0.00-0.00) | 0.00 (0.00-0.00) | 0.02 (0.01-0.05) | 0.02 (0.01-0.05) | -0.19 (-0.51,0.13) |  | 0.00 (0.00-0.00) | 0.00 (0.00-0.00) | 0.02 (0.01-0.03) | 0.02 (0.01-0.03) | -0.40 (-0.73,-0.07) |  | 0.02 (0.01-0.03) | 0.01 (0.01-0.02) | 1.48 (0.70-2.90) | 1.36 (0.68-2.89) | -0.39 (-0.72,-0.07) |
| North Macedonia | 1.27 (0.87-1.63) | 0.12 (0.08-0.19) | 0.13 (0.09-0.16) | 0.01 (0.01-0.02) | -6.87 (-7.75,-5.98) |  | 0.83 (0.58-1.06) | 0.06 (0.04-0.09) | 0.08 (0.06-0.11) | 0.01 (0.00-0.01) | -8.04 (-8.89,-7.19) |  | 73.49 (51.01-94.00) | 5.35 (3.49-8.16) | 7.38 (5.12-9.44) | 0.49 (0.32-0.75) | -8.07 (-8.92,-7.21) |
| Northern Mariana Islands | 0.00 (0.00-0.00) | 0.00 (0.00-0.00) | 0.01 (0.00-0.01) | 0.00 (0.00-0.01) | -0.92 (-1.73,-0.10) |  | 0.00 (0.00-0.00) | 0.00 (0.00-0.00) | 0.00 (0.00-0.01) | 0.00 (0.00-0.00) | -1.04 (-1.81,-0.27) |  | 0.07 (0.04-0.13) | 0.05 (0.03-0.10) | 0.33 (0.19-0.58) | 0.22 (0.12-0.41) | -1.05 (-1.82,-0.28) |
| Norway | 0.53 (0.49-0.58) | 1.34 (1.11-1.61) | 0.03 (0.02-0.03) | 0.05 (0.04-0.06) | 1.91 (1.20,2.63) |  | 0.19 (0.17-0.20) | 0.29 (0.24-0.34) | 0.01 (0.01-0.01) | 0.01 (0.01-0.01) | 0.46 (-0.39,1.33) |  | 16.53 (15.20-17.83) | 25.86 (21.68-31.02) | 0.78 (0.72-0.84) | 0.95 (0.80-1.14) | 0.50 (-0.35,1.36) |
| Oman | 0.53 (0.32-0.83) | 0.64 (0.41-1.00) | 0.05 (0.03-0.08) | 0.03 (0.02-0.04) | -1.31 (-2.25,-0.35) |  | 0.36 (0.21-0.56) | 0.29 (0.18-0.46) | 0.04 (0.02-0.06) | 0.01 (0.01-0.02) | -2.53 (-3.33,-1.72) |  | 31.70 (18.85-49.59) | 25.67 (16.18-40.23) | 3.20 (1.90-5.00) | 1.09 (0.69-1.71) | -2.51 (-3.32,-1.69) |
| Pakistan | 111.80 (64.86-163.19) | 172.25 (118.07-241.33) | 0.20 (0.12-0.29) | 0.15 (0.10-0.20) | -0.22 (-0.53,0.09) |  | 78.88 (45.64-116.54) | 119.70 (82.00-167.93) | 0.14 (0.08-0.21) | 0.10 (0.07-0.14) | -0.28 (-0.58,0.03) |  | 6974.88 (4042.11-10283.13) | 10557.66 (7232.58-14775.52) | 12.55 (7.27-18.51) | 8.96 (6.14-12.55) | -0.28 (-0.58,0.02) |
| Palau | 0.00 (0.00-0.00) | 0.00 (0.00-0.00) | 0.03 (0.01-0.05) | 0.01 (0.01-0.02) | -2.10 (-2.45,-1.74) |  | 0.00 (0.00-0.00) | 0.00 (0.00-0.00) | 0.02 (0.01-0.03) | 0.01 (0.00-0.02) | -2.23 (-2.58,-1.87) |  | 0.12 (0.05-0.23) | 0.06 (0.03-0.12) | 1.52 (0.68-3.07) | 0.66 (0.31-1.36) | -2.23 (-2.59,-1.87) |
| Palestine | 1.38 (0.87-2.03) | 1.08 (0.71-1.66) | 0.13 (0.09-0.20) | 0.04 (0.03-0.06) | -3.33 (-3.53,-3.14) |  | 0.94 (0.59-1.40) | 0.65 (0.43-1.00) | 0.09 (0.06-0.14) | 0.03 (0.02-0.04) | -3.68 (-3.90,-3.45) |  | 83.16 (52.19-123.46) | 57.37 (37.59-88.06) | 8.13 (5.10-12.06) | 2.23 (1.46-3.43) | -3.70 (-3.92,-3.48) |
| Panama | 0.91 (0.75-1.07) | 0.85 (0.66-1.08) | 0.08 (0.06-0.09) | 0.04 (0.03-0.05) | -1.74 (-2.10,-1.39) |  | 0.61 (0.51-0.72) | 0.50 (0.39-0.62) | 0.05 (0.04-0.06) | 0.02 (0.02-0.03) | -2.21 (-2.55,-1.86) |  | 53.99 (44.62-63.20) | 43.56 (33.97-54.92) | 4.52 (3.74-5.29) | 2.03 (1.58-2.56) | -2.20 (-2.55,-1.85) |
| Papua New Guinea | 1.05 (0.61-1.92) | 1.62 (0.85-2.99) | 0.05 (0.03-0.09) | 0.03 (0.02-0.06) | -1.87 (-2.16,-1.57) |  | 0.74 (0.42-1.41) | 1.12 (0.59-2.08) | 0.04 (0.02-0.07) | 0.02 (0.01-0.04) | -1.90 (-2.20,-1.60) |  | 65.48 (37.57-125.08) | 99.35 (52.54-184.76) | 3.19 (1.83-6.10) | 1.90 (1.00-3.53) | -1.90 (-2.20,-1.61) |
| Paraguay | 1.63 (1.10-2.37) | 1.42 (0.86-2.31) | 0.08 (0.05-0.12) | 0.04 (0.02-0.06) | -1.44 (-1.82,-1.06) |  | 1.13 (0.76-1.65) | 0.91 (0.55-1.48) | 0.06 (0.04-0.08) | 0.03 (0.02-0.04) | -1.67 (-2.06,-1.29) |  | 98.87 (66.78-145.35) | 80.22 (48.20-130.73) | 4.89 (3.30-7.19) | 2.24 (1.34-3.65) | -1.66 (-2.04,-1.27) |
| Peru | 9.09 (5.52-13.16) | 3.43 (1.81-6.00) | 0.08 (0.05-0.12) | 0.02 (0.01-0.03) | -4.56 (-4.84,-4.27) |  | 6.34 (3.86-9.26) | 2.03 (1.08-3.54) | 0.06 (0.04-0.09) | 0.01 (0.01-0.02) | -5.09 (-5.34,-4.85) |  | 557.65 (340.24-813.35) | 177.53 (94.64-308.29) | 5.16 (3.15-7.52) | 0.98 (0.52-1.70) | -5.12 (-5.36,-4.87) |
| Philippines | 61.31 (40.94-80.22) | 27.20 (21.19-35.19) | 0.19 (0.13-0.25) | 0.05 (0.04-0.06) | -4.14 (-4.32,-3.96) |  | 42.84 (28.84-55.97) | 18.83 (14.73-24.45) | 0.14 (0.09-0.18) | 0.03 (0.03-0.04) | -4.16 (-4.34,-3.99) |  | 3753.79 (2525.79-4903.91) | 1642.38 (1283.27-2137.34) | 11.92 (8.02-15.57) | 2.90 (2.27-3.77) | -4.17 (-4.35,-4.00) |
| Poland | 0.99 (0.90-1.11) | 0.89 (0.73-1.06) | 0.01 (0.00-0.01) | 0.00 (0.00-0.01) | 0.99 (-0.14,2.13) |  | 0.66 (0.59-0.73) | 0.33 (0.28-0.39) | 0.00 (0.00-0.00) | 0.00 (0.00-0.00) | -1.19 (-2.21,-0.16) |  | 57.27 (51.71-64.17) | 29.06 (24.24-34.08) | 0.30 (0.27-0.34) | 0.15 (0.13-0.18) | -1.15 (-2.17,-0.11) |
| Portugal | 1.22 (1.00-1.44) | 0.91 (0.69-1.20) | 0.02 (0.02-0.03) | 0.02 (0.01-0.02) | -0.66 (-0.95,-0.36) |  | 0.70 (0.57-0.81) | 0.21 (0.16-0.27) | 0.01 (0.01-0.02) | 0.00 (0.00-0.01) | -3.86 (-4.23,-3.49) |  | 61.28 (50.37-71.83) | 18.66 (14.47-23.88) | 1.21 (0.99-1.42) | 0.35 (0.27-0.45) | -3.80 (-4.16,-3.43) |
| Puerto Rico | 0.23 (0.19-0.28) | 0.07 (0.05-0.09) | 0.01 (0.01-0.02) | 0.00 (0.00-0.01) | -3.87 (-4.20,-3.54) |  | 0.15 (0.12-0.18) | 0.03 (0.02-0.03) | 0.01 (0.01-0.01) | 0.00 (0.00-0.00) | -5.67 (-6.03,-5.32) |  | 12.98 (10.61-15.86) | 2.31 (1.81-2.95) | 0.72 (0.59-0.88) | 0.14 (0.11-0.18) | -5.66 (-6.01,-5.31) |
| Qatar | 0.11 (0.08-0.16) | 0.47 (0.30-0.73) | 0.05 (0.03-0.07) | 0.03 (0.02-0.05) | -1.84 (-2.42,-1.27) |  | 0.07 (0.05-0.11) | 0.14 (0.09-0.22) | 0.03 (0.02-0.05) | 0.01 (0.01-0.01) | -4.49 (-4.94,-4.05) |  | 6.46 (4.42-9.23) | 12.63 (7.98-19.83) | 2.90 (1.99-4.15) | 0.85 (0.54-1.33) | -4.46 (-4.90,-4.02) |
| Republic of Korea | 20.13 (12.13-29.36) | 5.13 (3.15-8.40) | 0.09 (0.05-0.13) | 0.02 (0.01-0.03) | -4.18 (-5.09,-3.26) |  | 13.39 (8.00-19.31) | 1.24 (0.80-1.98) | 0.06 (0.04-0.09) | 0.00 (0.00-0.01) | -8.06 (-8.53,-7.58) |  | 1167.00 (697.74-1692.83) | 109.61 (69.91-175.19) | 5.28 (3.15-7.65) | 0.43 (0.27-0.68) | -7.99 (-8.47,-7.50) |
| Republic of Moldova | 4.70 (4.05-5.47) | 1.01 (0.76-1.35) | 0.21 (0.18-0.25) | 0.06 (0.04-0.07) | -3.53 (-4.52,-2.54) |  | 3.10 (2.67-3.61) | 0.55 (0.43-0.73) | 0.14 (0.12-0.16) | 0.03 (0.02-0.04) | -4.15 (-5.02,-3.27) |  | 272.87 (235.21-317.55) | 48.22 (37.25-63.89) | 12.27 (10.58-14.28) | 2.68 (2.07-3.56) | -4.14 (-5.02,-3.25) |
| Romania | 4.84 (3.83-6.22) | 0.69 (0.50-0.92) | 0.04 (0.03-0.05) | 0.01 (0.01-0.01) | -4.73 (-5.55,-3.90) |  | 3.21 (2.55-4.08) | 0.30 (0.21-0.40) | 0.03 (0.02-0.03) | 0.00 (0.00-0.00) | -6.21 (-6.91,-5.51) |  | 282.78 (224.13-359.31) | 26.09 (18.83-34.95) | 2.42 (1.92-3.07) | 0.28 (0.20-0.37) | -6.21 (-6.91,-5.50) |
| Russian Federation | 57.66 (55.01-60.65) | 22.60 (20.28-24.90) | 0.08 (0.07-0.08) | 0.03 (0.03-0.03) | -2.63 (-3.62,-1.62) |  | 37.18 (35.51-39.10) | 10.02 (9.04-10.97) | 0.05 (0.05-0.05) | 0.01 (0.01-0.02) | -4.12 (-4.91,-3.32) |  | 3257.08 (3107.44-3421.01) | 878.00 (790.99-964.76) | 4.31 (4.12-4.53) | 1.21 (1.09-1.33) | -4.08 (-4.86,-3.29) |
| Rwanda | 9.65 (4.96-15.67) | 5.04 (2.72-9.43) | 0.27 (0.14-0.44) | 0.08 (0.04-0.14) | -4.55 (-5.04,-4.05) |  | 6.90 (3.55-11.07) | 3.52 (1.91-6.65) | 0.19 (0.10-0.31) | 0.05 (0.03-0.10) | -4.62 (-5.11,-4.13) |  | 607.36 (313.40-978.58) | 309.42 (167.02-585.05) | 16.90 (8.72-27.22) | 4.66 (2.52-8.82) | -4.63 (-5.12,-4.13) |
| Saint Kitts and Nevis | 0.01 (0.00-0.01) | 0.00 (0.00-0.00) | 0.03 (0.02-0.03) | 0.01 (0.01-0.01) | -3.52 (-3.96,-3.08) |  | 0.00 (0.00-0.00) | 0.00 (0.00-0.00) | 0.02 (0.02-0.02) | 0.00 (0.00-0.01) | -3.61 (-4.08,-3.13) |  | 0.35 (0.29-0.42) | 0.13 (0.09-0.17) | 1.70 (1.41-2.04) | 0.43 (0.32-0.57) | -3.67 (-4.13,-3.20) |
| Saint Lucia | 0.01 (0.01-0.02) | 0.00 (0.00-0.01) | 0.02 (0.02-0.03) | 0.01 (0.00-0.01) | -4.46 (-4.86,-4.06) |  | 0.01 (0.01-0.01) | 0.00 (0.00-0.00) | 0.01 (0.01-0.02) | 0.00 (0.00-0.00) | -4.60 (-5.01,-4.19) |  | 0.83 (0.64-1.05) | 0.26 (0.20-0.35) | 1.22 (0.94-1.54) | 0.30 (0.22-0.40) | -4.63 (-5.04,-4.22) |
| Saint Vincent and the Grenadines | 0.02 (0.01-0.02) | 0.00 (0.00-0.01) | 0.03 (0.03-0.04) | 0.01 (0.01-0.01) | -4.54 (-4.92,-4.15) |  | 0.01 (0.01-0.02) | 0.00 (0.00-0.00) | 0.02 (0.02-0.03) | 0.01 (0.00-0.01) | -4.60 (-5.02,-4.19) |  | 1.15 (0.91-1.44) | 0.29 (0.22-0.36) | 2.10 (1.66-2.64) | 0.50 (0.39-0.64) | -4.62 (-5.02,-4.21) |
| Samoa | 0.07 (0.04-0.11) | 0.04 (0.02-0.10) | 0.08 (0.05-0.14) | 0.04 (0.02-0.09) | -2.38 (-2.61,-2.16) |  | 0.04 (0.03-0.08) | 0.03 (0.01-0.07) | 0.05 (0.03-0.09) | 0.03 (0.01-0.06) | -2.49 (-2.70,-2.28) |  | 4.00 (2.32-6.87) | 2.42 (1.06-6.02) | 4.74 (2.75-8.13) | 2.27 (0.99-5.64) | -2.49 (-2.70,-2.28) |
| San Marino | 0.00 (0.00-0.01) | 0.00 (0.00-0.00) | 0.04 (0.02-0.06) | 0.01 (0.01-0.02) | -2.94 (-3.34,-2.54) |  | 0.00 (0.00-0.00) | 0.00 (0.00-0.00) | 0.01 (0.01-0.01) | 0.00 (0.00-0.00) | -3.24 (-3.55,-2.93) |  | 0.09 (0.06-0.14) | 0.04 (0.02-0.07) | 0.79 (0.50-1.19) | 0.24 (0.14-0.43) | -3.25 (-3.56,-2.94) |
| Sao Tome and Principe | 0.08 (0.05-0.12) | 0.02 (0.01-0.04) | 0.14 (0.09-0.21) | 0.02 (0.01-0.04) | -6.65 (-7.14,-6.16) |  | 0.06 (0.04-0.09) | 0.01 (0.00-0.03) | 0.10 (0.06-0.14) | 0.01 (0.00-0.03) | -6.78 (-7.28,-6.28) |  | 5.21 (3.30-7.81) | 1.02 (0.42-2.46) | 8.59 (5.45-12.88) | 0.94 (0.39-2.27) | -6.77 (-7.27,-6.28) |
| Saudi Arabia | 9.16 (5.69-15.75) | 2.45 (1.48-4.18) | 0.12 (0.07-0.20) | 0.01 (0.01-0.02) | -7.19 (-7.43,-6.95) |  | 6.31 (3.88-11.03) | 1.26 (0.76-2.13) | 0.08 (0.05-0.14) | 0.01 (0.00-0.01) | -8.11 (-8.31,-7.91) |  | 559.31 (344.97-974.58) | 110.82 (67.10-187.95) | 7.05 (4.35-12.29) | 0.59 (0.36-1.00) | -8.12 (-8.31,-7.92) |
| Senegal | 11.32 (6.66-16.75) | 5.94 (3.90-8.59) | 0.30 (0.17-0.44) | 0.07 (0.05-0.11) | -4.15 (-4.53,-3.78) |  | 7.94 (4.75-11.93) | 4.09 (2.72-5.97) | 0.21 (0.12-0.31) | 0.05 (0.03-0.08) | -4.21 (-4.58,-3.84) |  | 705.04 (422.50-1054.59) | 364.89 (242.53-529.65) | 18.48 (11.07-27.63) | 4.60 (3.06-6.68) | -4.19 (-4.57,-3.82) |
| Serbia | 2.03 (1.03-3.48) | 0.30 (0.15-0.55) | 0.04 (0.02-0.07) | 0.01 (0.00-0.01) | -6.00 (-6.46,-5.54) |  | 1.31 (0.67-2.21) | 0.12 (0.06-0.22) | 0.03 (0.01-0.05) | 0.00 (0.00-0.00) | -7.70 (-8.14,-7.26) |  | 114.73 (58.83-194.32) | 10.53 (5.32-18.83) | 2.38 (1.22-4.04) | 0.24 (0.12-0.42) | -7.72 (-8.16,-7.28) |
| Seychelles | 0.01 (0.01-0.01) | 0.00 (0.00-0.01) | 0.02 (0.02-0.04) | 0.01 (0.01-0.01) | -2.44 (-3.21,-1.67) |  | 0.01 (0.00-0.01) | 0.00 (0.00-0.00) | 0.02 (0.01-0.03) | 0.01 (0.00-0.01) | -2.76 (-3.52,-1.99) |  | 0.55 (0.37-0.84) | 0.23 (0.16-0.32) | 1.50 (1.02-2.31) | 0.44 (0.31-0.62) | -2.74 (-3.50,-1.97) |
| Sierra Leone | 14.47 (7.60-21.67) | 7.93 (4.42-12.20) | 0.70 (0.37-1.04) | 0.18 (0.10-0.28) | -4.70 (-5.12,-4.29) |  | 10.15 (5.36-15.28) | 5.48 (3.05-8.36) | 0.49 (0.26-0.74) | 0.12 (0.07-0.19) | -4.75 (-5.17,-4.33) |  | 905.49 (477.35-1363.50) | 489.94 (272.56-748.51) | 43.62 (23.00-65.68) | 11.05 (6.15-16.88) | -4.74 (-5.16,-4.32) |
| Singapore | 0.59 (0.49-0.71) | 0.35 (0.28-0.45) | 0.04 (0.03-0.05) | 0.01 (0.01-0.02) | -3.16 (-4.01,-2.31) |  | 0.33 (0.28-0.40) | 0.08 (0.07-0.10) | 0.02 (0.02-0.03) | 0.00 (0.00-0.00) | -6.27 (-6.83,-5.70) |  | 29.22 (24.36-34.90) | 7.46 (5.99-9.23) | 1.92 (1.60-2.29) | 0.26 (0.21-0.32) | -6.21 (-6.78,-5.65) |
| Slovakia | 0.74 (0.48-1.11) | 0.34 (0.19-0.60) | 0.03 (0.02-0.04) | 0.01 (0.01-0.02) | -2.60 (-3.16,-2.03) |  | 0.46 (0.30-0.70) | 0.12 (0.07-0.21) | 0.02 (0.01-0.03) | 0.00 (0.00-0.01) | -4.73 (-5.17,-4.29) |  | 40.41 (26.01-61.06) | 10.18 (5.77-18.23) | 1.53 (0.98-2.31) | 0.37 (0.21-0.67) | -4.68 (-5.13,-4.24) |
| Slovenia | 0.24 (0.20-0.28) | 0.07 (0.05-0.09) | 0.02 (0.02-0.03) | 0.01 (0.00-0.01) | -3.01 (-3.57,-2.45) |  | 0.12 (0.10-0.14) | 0.02 (0.01-0.02) | 0.01 (0.01-0.01) | 0.00 (0.00-0.00) | -5.70 (-6.21,-5.17) |  | 10.60 (8.99-12.49) | 1.49 (1.10-1.99) | 1.07 (0.91-1.27) | 0.14 (0.11-0.19) | -5.67 (-6.18,-5.15) |
| Solomon Islands | 0.06 (0.02-0.15) | 0.06 (0.03-0.15) | 0.04 (0.01-0.09) | 0.02 (0.01-0.04) | -2.47 (-2.90,-2.03) |  | 0.05 (0.02-0.11) | 0.04 (0.02-0.10) | 0.03 (0.01-0.06) | 0.01 (0.01-0.03) | -2.51 (-2.94,-2.07) |  | 4.06 (1.51-9.59) | 3.77 (1.82-8.94) | 2.39 (0.89-5.66) | 1.10 (0.53-2.62) | -2.52 (-2.95,-2.09) |
| Somalia | 12.86 (6.66-21.73) | 18.39 (7.08-37.31) | 0.32 (0.17-0.55) | 0.17 (0.07-0.35) | -1.70 (-2.11,-1.28) |  | 9.14 (4.73-15.51) | 13.01 (5.11-26.27) | 0.23 (0.12-0.39) | 0.12 (0.05-0.24) | -1.71 (-2.12,-1.30) |  | 807.03 (416.23-1365.96) | 1148.57 (450.79-2314.47) | 20.33 (10.49-34.41) | 10.63 (4.17-21.42) | -1.71 (-2.12,-1.30) |
| South Africa | 8.17 (5.37-11.00) | 7.55 (5.43-9.87) | 0.04 (0.03-0.06) | 0.03 (0.02-0.03) | -1.71 (-1.88,-1.54) |  | 5.73 (3.76-7.71) | 5.21 (3.79-6.83) | 0.03 (0.02-0.04) | 0.02 (0.01-0.02) | -1.81 (-1.99,-1.64) |  | 502.08 (330.09-675.36) | 455.20 (330.72-595.56) | 2.71 (1.78-3.65) | 1.60 (1.16-2.10) | -1.81 (-1.97,-1.64) |
| South Sudan | 8.82 (3.67-15.00) | 14.37 (5.89-23.78) | 0.30 (0.13-0.51) | 0.30 (0.12-0.49) | 0.37 (-0.19,0.93) |  | 6.20 (2.56-10.41) | 9.99 (4.12-16.42) | 0.21 (0.09-0.35) | 0.21 (0.09-0.34) | 0.33 (-0.22,0.89) |  | 548.30 (227.62-915.51) | 881.77 (364.96-1450.38) | 18.66 (7.75-31.15) | 18.23 (7.55-29.99) | 0.33 (-0.23,0.88) |
| Spain | 6.66 (5.83-7.63) | 6.26 (5.15-7.64) | 0.03 (0.03-0.04) | 0.03 (0.02-0.03) | -0.36 (-0.82,0.10) |  | 2.63 (2.29-3.02) | 1.40 (1.14-1.69) | 0.01 (0.01-0.02) | 0.01 (0.01-0.01) | -1.91 (-2.18,-1.63) |  | 231.88 (202.51-266.37) | 124.97 (103.00-151.31) | 1.20 (1.04-1.37) | 0.55 (0.45-0.66) | -1.87 (-2.15,-1.59) |
| Sri Lanka | 0.98 (0.72-1.30) | 0.45 (0.28-0.73) | 0.01 (0.01-0.02) | 0.00 (0.00-0.01) | -3.34 (-3.73,-2.95) |  | 0.69 (0.50-0.92) | 0.26 (0.16-0.42) | 0.01 (0.01-0.01) | 0.00 (0.00-0.00) | -4.05 (-4.49,-3.62) |  | 60.17 (43.61-80.02) | 22.44 (14.29-36.41) | 0.70 (0.51-0.93) | 0.20 (0.13-0.33) | -4.03 (-4.46,-3.59) |
| Sudan | 27.98 (14.44-46.37) | 22.95 (14.06-36.88) | 0.28 (0.14-0.46) | 0.11 (0.06-0.17) | -2.57 (-2.80,-2.34) |  | 19.72 (10.19-32.53) | 15.54 (9.70-24.78) | 0.20 (0.10-0.32) | 0.07 (0.04-0.11) | -2.70 (-2.93,-2.46) |  | 1741.52 (898.54-2875.74) | 1365.71 (854.49-2177.61) | 17.40 (8.98-28.73) | 6.29 (3.94-10.03) | -2.71 (-2.95,-2.47) |
| Suriname | 0.06 (0.04-0.08) | 0.04 (0.03-0.07) | 0.03 (0.02-0.04) | 0.01 (0.01-0.02) | -2.32 (-2.54,-2.10) |  | 0.04 (0.03-0.06) | 0.03 (0.02-0.05) | 0.02 (0.01-0.03) | 0.01 (0.01-0.02) | -2.40 (-2.63,-2.17) |  | 3.44 (2.28-4.90) | 2.60 (1.64-3.97) | 1.78 (1.18-2.54) | 0.90 (0.57-1.37) | -2.41 (-2.64,-2.19) |
| Sweden | 1.61 (1.38-1.91) | 1.17 (0.90-1.52) | 0.04 (0.03-0.04) | 0.02 (0.02-0.03) | -0.56 (-1.66,0.56) |  | 0.46 (0.40-0.53) | 0.26 (0.20-0.33) | 0.01 (0.01-0.01) | 0.00 (0.00-0.01) | -1.37 (-2.53,-0.19) |  | 41.05 (35.43-47.47) | 22.90 (18.01-29.05) | 0.96 (0.83-1.11) | 0.44 (0.35-0.56) | -1.35 (-2.51,-0.16) |
| Switzerland | 1.67 (1.41-1.96) | 0.72 (0.57-0.91) | 0.05 (0.04-0.06) | 0.02 (0.01-0.02) | -3.56 (-3.73,-3.38) |  | 0.44 (0.38-0.51) | 0.16 (0.13-0.20) | 0.01 (0.01-0.01) | 0.00 (0.00-0.00) | -4.01 (-4.30,-3.73) |  | 39.41 (33.68-45.79) | 14.39 (11.47-17.63) | 1.15 (0.98-1.33) | 0.32 (0.26-0.40) | -4.02 (-4.30,-3.73) |
| Syrian Arab Republic | 9.34 (6.01-12.58) | 2.46 (1.18-3.77) | 0.15 (0.09-0.20) | 0.04 (0.02-0.05) | -4.22 (-4.97,-3.46) |  | 6.55 (4.23-8.82) | 1.42 (0.71-2.16) | 0.10 (0.07-0.14) | 0.02 (0.01-0.03) | -4.87 (-5.59,-4.14) |  | 573.49 (368.49-774.66) | 124.68 (62.02-190.28) | 9.02 (5.80-12.18) | 1.78 (0.88-2.71) | -4.86 (-5.58,-4.12) |
| Taiwan (Province of China) | 5.49 (4.86-6.24) | 3.19 (2.61-3.86) | 0.05 (0.05-0.06) | 0.03 (0.02-0.03) | -0.31 (-1.40,0.80) |  | 3.34 (2.96-3.80) | 0.91 (0.76-1.09) | 0.03 (0.03-0.04) | 0.01 (0.01-0.01) | -3.17 (-4.06,-2.27) |  | 291.41 (258.32-330.85) | 80.58 (67.33-95.99) | 2.86 (2.53-3.25) | 0.68 (0.57-0.81) | -3.09 (-3.99,-2.19) |
| Tajikistan | 4.13 (1.75-6.81) | 2.97 (1.63-5.15) | 0.15 (0.07-0.25) | 0.06 (0.03-0.10) | -3.39 (-3.86,-2.92) |  | 2.86 (1.20-4.69) | 2.02 (1.10-3.51) | 0.11 (0.04-0.17) | 0.04 (0.02-0.07) | -3.48 (-3.94,-3.02) |  | 251.93 (106.06-414.04) | 177.85 (96.64-308.98) | 9.38 (3.95-15.42) | 3.50 (1.90-6.08) | -3.46 (-3.92,-3.00) |
| Thailand | 48.55 (35.10-65.64) | 8.64 (6.28-11.53) | 0.17 (0.12-0.23) | 0.03 (0.02-0.03) | -6.60 (-6.98,-6.22) |  | 33.63 (24.63-45.26) | 4.74 (3.42-6.42) | 0.12 (0.09-0.16) | 0.01 (0.01-0.02) | -7.38 (-7.80,-6.96) |  | 2934.58 (2145.87-3939.13) | 415.43 (300.49-560.85) | 10.34 (7.56-13.88) | 1.25 (0.90-1.68) | -7.36 (-7.78,-6.95) |
| Timor-Leste | 0.55 (0.12-0.99) | 0.27 (0.13-0.47) | 0.14 (0.03-0.25) | 0.04 (0.02-0.07) | -4.67 (-5.00,-4.33) |  | 0.39 (0.08-0.70) | 0.19 (0.09-0.32) | 0.10 (0.02-0.18) | 0.03 (0.01-0.05) | -4.75 (-5.08,-4.42) |  | 34.28 (7.41-61.54) | 16.52 (7.77-28.30) | 8.78 (1.90-15.76) | 2.36 (1.11-4.05) | -4.75 (-5.09,-4.42) |
| Togo | 3.07 (1.91-4.60) | 2.38 (1.37-4.01) | 0.17 (0.10-0.25) | 0.06 (0.03-0.10) | -3.36 (-3.58,-3.13) |  | 2.15 (1.34-3.22) | 1.64 (0.94-2.75) | 0.12 (0.07-0.18) | 0.04 (0.02-0.07) | -3.41 (-3.64,-3.17) |  | 191.22 (119.21-284.49) | 146.34 (84.36-245.22) | 10.49 (6.54-15.60) | 3.50 (2.02-5.86) | -3.40 (-3.63,-3.16) |
| Tokelau | 0.00 (0.00-0.00) | 0.00 (0.00-0.00) | 0.02 (0.01-0.04) | 0.01 (0.00-0.03) | -2.36 (-2.87,-1.85) |  | 0.00 (0.00-0.00) | 0.00 (0.00-0.00) | 0.01 (0.01-0.03) | 0.01 (0.00-0.02) | -2.53 (-3.03,-2.02) |  | 0.01 (0.00-0.02) | 0.00 (0.00-0.01) | 1.02 (0.46-2.32) | 0.56 (0.22-1.64) | -2.53 (-3.03,-2.02) |
| Tonga | 0.06 (0.03-0.11) | 0.04 (0.02-0.10) | 0.12 (0.06-0.22) | 0.08 (0.04-0.18) | -1.28 (-1.75,-0.81) |  | 0.04 (0.02-0.08) | 0.03 (0.01-0.06) | 0.08 (0.04-0.15) | 0.06 (0.03-0.12) | -1.40 (-1.88,-0.91) |  | 3.45 (1.78-6.82) | 2.60 (1.20-5.73) | 6.97 (3.60-13.81) | 4.90 (2.26-10.78) | -1.40 (-1.88,-0.92) |
| Trinidad and Tobago | 0.13 (0.11-0.15) | 0.04 (0.03-0.06) | 0.02 (0.02-0.02) | 0.01 (0.00-0.01) | -3.57 (-3.88,-3.26) |  | 0.09 (0.08-0.11) | 0.03 (0.02-0.04) | 0.01 (0.01-0.02) | 0.00 (0.00-0.01) | -3.85 (-4.18,-3.52) |  | 7.84 (6.63-9.23) | 2.55 (1.87-3.41) | 1.30 (1.10-1.53) | 0.37 (0.27-0.49) | -3.83 (-4.14,-3.51) |
| Tunisia | 3.03 (1.91-4.32) | 1.02 (0.64-1.59) | 0.07 (0.05-0.10) | 0.02 (0.01-0.03) | -4.03 (-4.44,-3.63) |  | 2.02 (1.29-2.88) | 0.53 (0.34-0.82) | 0.05 (0.03-0.07) | 0.01 (0.01-0.01) | -4.85 (-5.20,-4.49) |  | 178.89 (113.25-254.37) | 46.61 (29.86-73.07) | 4.29 (2.71-6.09) | 0.79 (0.50-1.23) | -4.85 (-5.21,-4.50) |
| Turkey | 17.10 (11.85-25.25) | 5.94 (4.05-9.13) | 0.06 (0.04-0.09) | 0.01 (0.01-0.02) | -4.23 (-4.38,-4.08) |  | 11.82 (8.23-17.54) | 2.89 (1.93-4.41) | 0.04 (0.03-0.06) | 0.01 (0.00-0.01) | -5.36 (-5.58,-5.14) |  | 1047.14 (727.04-1552.82) | 255.96 (171.65-391.73) | 3.64 (2.53-5.40) | 0.61 (0.41-0.94) | -5.35 (-5.57,-5.13) |
| Turkmenistan | 3.76 (3.18-4.35) | 0.93 (0.72-1.18) | 0.20 (0.17-0.24) | 0.04 (0.03-0.05) | -5.62 (-6.14,-5.10) |  | 2.58 (2.17-2.97) | 0.62 (0.48-0.78) | 0.14 (0.12-0.16) | 0.02 (0.02-0.03) | -5.77 (-6.28,-5.25) |  | 228.68 (192.39-262.81) | 54.24 (42.13-69.04) | 12.36 (10.40-14.21) | 2.10 (1.63-2.68) | -5.77 (-6.28,-5.26) |
| Tuvalu | 0.00 (0.00-0.00) | 0.00 (0.00-0.00) | 0.05 (0.02-0.08) | 0.01 (0.01-0.02) | -4.07 (-4.20,-3.94) |  | 0.00 (0.00-0.00) | 0.00 (0.00-0.00) | 0.03 (0.02-0.06) | 0.01 (0.00-0.02) | -4.14 (-4.27,-4.01) |  | 0.13 (0.07-0.24) | 0.05 (0.02-0.09) | 2.83 (1.42-4.95) | 0.73 (0.40-1.41) | -4.15 (-4.28,-4.02) |
| Uganda | 46.95 (29.71-68.58) | 57.97 (30.88-105.47) | 0.54 (0.34-0.79) | 0.27 (0.14-0.49) | -1.88 (-2.17,-1.59) |  | 32.95 (21.25-47.94) | 40.31 (21.66-73.63) | 0.38 (0.25-0.55) | 0.19 (0.10-0.34) | -1.92 (-2.22,-1.62) |  | 2919.43 (1880.64-4261.36) | 3556.24 (1913.67-6511.26) | 33.77 (21.75-49.29) | 16.42 (8.84-30.06) | -1.93 (-2.23,-1.63) |
| Ukraine | 13.56 (10.69-17.44) | 2.50 (1.89-3.10) | 0.05 (0.04-0.07) | 0.01 (0.01-0.01) | -3.42 (-4.33,-2.51) |  | 8.24 (6.51-10.58) | 1.35 (1.03-1.68) | 0.03 (0.02-0.04) | 0.01 (0.00-0.01) | -4.03 (-4.82,-3.23) |  | 726.90 (573.99-935.25) | 119.42 (90.99-147.64) | 2.76 (2.18-3.55) | 0.55 (0.42-0.69) | -4.00 (-4.80,-3.18) |
| United Arab Emirates | 0.36 (0.20-0.60) | 0.43 (0.29-0.63) | 0.04 (0.02-0.06) | 0.01 (0.01-0.01) | -4.42 (-5.14,-3.70) |  | 0.25 (0.14-0.42) | 0.27 (0.18-0.40) | 0.03 (0.02-0.04) | 0.01 (0.00-0.01) | -4.77 (-5.45,-4.09) |  | 22.17 (12.45-36.42) | 23.27 (15.65-34.81) | 2.37 (1.33-3.89) | 0.48 (0.33-0.72) | -4.78 (-5.46,-4.09) |
| United Kingdom | 5.69 (5.45-5.93) | 12.66 (11.30-13.86) | 0.02 (0.02-0.02) | 0.04 (0.03-0.04) | 3.85 (3.26,4.45) |  | 2.29 (2.20-2.38) | 3.07 (2.78-3.33) | 0.01 (0.01-0.01) | 0.01 (0.01-0.01) | 1.91 (1.44,2.39) |  | 203.19 (195.01-211.71) | 273.62 (247.88-297.57) | 0.71 (0.68-0.74) | 0.81 (0.73-0.88) | 1.95 (1.48,2.43) |
| United Republic of Tanzania | 42.64 (26.59-62.10) | 42.77 (24.28-72.51) | 0.33 (0.21-0.48) | 0.15 (0.08-0.25) | -2.18 (-2.38,-1.98) |  | 29.97 (18.88-43.29) | 29.73 (16.91-50.53) | 0.23 (0.15-0.34) | 0.10 (0.06-0.17) | -2.22 (-2.42,-2.01) |  | 2651.47 (1672.22-3816.08) | 2621.80 (1490.40-4486.09) | 20.52 (12.94-29.54) | 8.97 (5.10-15.35) | -2.23 (-2.43,-2.02) |
| United States of America | 50.51 (49.25-51.82) | 81.41 (72.10-90.92) | 0.04 (0.04-0.04) | 0.05 (0.04-0.05) | 1.00 (0.71,1.29) |  | 17.60 (17.20-18.01) | 20.56 (18.32-22.82) | 0.01 (0.01-0.01) | 0.01 (0.01-0.01) | -0.02 (-0.18,0.13) |  | 1562.20 (1523.61-1600.68) | 1834.26 (1638.72-2040.11) | 1.23 (1.20-1.26) | 1.10 (0.99-1.23) | -0.00 (-0.16,0.16) |
| United States Virgin Islands | 0.00 (0.00-0.01) | 0.00 (0.00-0.00) | 0.01 (0.01-0.01) | 0.00 (0.00-0.01) | -4.25 (-4.54,-3.96) |  | 0.00 (0.00-0.00) | 0.00 (0.00-0.00) | 0.01 (0.00-0.01) | 0.00 (0.00-0.00) | -4.58 (-4.88,-4.28) |  | 0.29 (0.19-0.43) | 0.06 (0.03-0.11) | 0.55 (0.35-0.80) | 0.13 (0.06-0.27) | -4.58 (-4.88,-4.29) |
| Uruguay | 0.07 (0.06-0.09) | 0.06 (0.04-0.08) | 0.00 (0.00-0.01) | 0.00 (0.00-0.00) | -0.50 (-1.28,0.27) |  | 0.05 (0.04-0.06) | 0.03 (0.02-0.04) | 0.00 (0.00-0.00) | 0.00 (0.00-0.00) | -1.13 (-1.89,-0.36) |  | 4.20 (3.39-5.22) | 2.75 (1.97-3.66) | 0.27 (0.22-0.33) | 0.16 (0.12-0.22) | -1.15 (-1.92,-0.38) |
| Uzbekistan | 9.89 (6.87-13.61) | 6.34 (4.59-8.52) | 0.09 (0.07-0.13) | 0.04 (0.03-0.05) | -2.72 (-3.03,-2.40) |  | 6.72 (4.75-9.22) | 4.25 (3.06-5.74) | 0.06 (0.05-0.09) | 0.02 (0.02-0.03) | -2.81 (-3.10,-2.51) |  | 592.39 (418.87-813.35) | 371.33 (267.37-500.96) | 5.65 (4.00-7.76) | 2.17 (1.56-2.93) | -2.83 (-3.12,-2.53) |
| Vanuatu | 0.02 (0.01-0.03) | 0.02 (0.01-0.03) | 0.02 (0.01-0.04) | 0.01 (0.01-0.02) | -1.62 (-2.16,-1.06) |  | 0.01 (0.01-0.02) | 0.01 (0.01-0.02) | 0.01 (0.01-0.03) | 0.01 (0.00-0.02) | -1.61 (-2.16,-1.06) |  | 0.99 (0.51-2.04) | 1.09 (0.49-2.11) | 1.31 (0.68-2.68) | 0.70 (0.31-1.35) | -1.63 (-2.17,-1.08) |
| Venezuela (Bolivarian Republic of) | 6.69 (6.21-7.17) | 6.33 (4.58-8.64) | 0.07 (0.07-0.08) | 0.05 (0.03-0.06) | -1.27 (-2.29,-0.24) |  | 4.63 (4.30-4.96) | 4.02 (2.92-5.44) | 0.05 (0.05-0.05) | 0.03 (0.02-0.04) | -1.60 (-2.60,-0.58) |  | 407.79 (378.71-436.13) | 354.05 (256.42-480.76) | 4.34 (4.03-4.64) | 2.66 (1.93-3.61) | -1.59 (-2.60,-0.56) |
| Viet Nam | 112.69 (75.56-158.88) | 37.54 (23.30-57.88) | 0.33 (0.22-0.47) | 0.07 (0.05-0.12) | -4.12 (-4.59,-3.65) |  | 77.37 (51.12-110.61) | 22.33 (13.84-34.95) | 0.23 (0.15-0.32) | 0.04 (0.03-0.07) | -4.59 (-5.03,-4.15) |  | 6849.05 (4538.73-9741.18) | 1969.58 (1218.46-3071.65) | 20.08 (13.30-28.56) | 3.93 (2.43-6.13) | -4.60 (-5.04,-4.15) |
| Yemen | 12.62 (7.24-20.67) | 8.57 (4.67-14.32) | 0.19 (0.11-0.30) | 0.05 (0.03-0.09) | -3.71 (-3.93,-3.50) |  | 8.90 (5.10-14.53) | 5.93 (3.22-10.01) | 0.13 (0.07-0.21) | 0.04 (0.02-0.06) | -3.81 (-4.01,-3.61) |  | 788.00 (450.47-1285.82) | 522.62 (285.35-879.56) | 11.56 (6.61-18.86) | 3.11 (1.70-5.23) | -3.82 (-4.02,-3.61) |
| Zambia | 21.51 (14.37-30.44) | 16.49 (7.34-35.99) | 0.54 (0.36-0.77) | 0.17 (0.08-0.37) | -3.88 (-4.15,-3.61) |  | 15.19 (10.15-21.70) | 11.53 (5.11-25.68) | 0.38 (0.26-0.55) | 0.12 (0.05-0.26) | -3.92 (-4.19,-3.64) |  | 1344.84 (898.44-1915.91) | 1015.48 (451.30-2252.48) | 33.89 (22.64-48.28) | 10.41 (4.63-23.08) | -3.93 (-4.21,-3.65) |
| Zimbabwe | 7.08 (3.78-11.79) | 10.28 (5.64-16.59) | 0.14 (0.07-0.23) | 0.13 (0.07-0.21) | -0.08 (-0.56,0.41) |  | 4.94 (2.65-8.20) | 7.28 (4.02-11.54) | 0.10 (0.05-0.16) | 0.09 (0.05-0.15) | 0.00 (-0.48,0.49) |  | 437.81 (233.78-726.44) | 638.84 (354.65-1016.71) | 8.47 (4.52-14.05) | 8.19 (4.55-13.04) | -0.05 (-0.53,0.45) |

**Abbreviations:** UI, uncertainty interval; ASR, age-standardised rate per 100,000; EAPC, estimated annual percentage change; CI, confidence interval; DALYs, disability-adjusted life-year; SDI, socio-demographic index.

**Table 14. Burden of liver cancer due to other causes in 204 countries and territories in 1990, 2021 and the estimated annual percentage changes (EAPC) from 1990 to 2021**

| **Country** | **Incidence** | | | | |  | **Deaths** | | | | |  | **DALYs** | | | | |
| --- | --- | --- | --- | --- | --- | --- | --- | --- | --- | --- | --- | --- | --- | --- | --- | --- | --- |
|  | **1990 No. (95% UI)** | **2021 No. (95% UI)** | **1990 ASR per 100 000 (95% UI)** | **2021 ASR per 100 000 (95% UI)** | **EAPC (95% CI)** |  | **1990 No. (95% UI)** | **2021 No. (95% UI)** | **1990 ASR per 100 000 (95% UI)** | **2021 ASR per 100 000 (95% UI)** | **EAPC (95% CI)** |  | **1990 No. (95% UI)** | **2021 No. (95% UI)** | **1990 ASR per 100 000 (95% UI)** | **2021 ASR per 100 000 (95% UI)** | **EAPC (95% CI)** |
| Afghanistan | 14.80 (8.35-24.52) | 29.29 (18.09-45.57) | 0.30 (0.17-0.49) | 0.19 (0.12-0.29) | -1.62 (-1.94,-1.31) |  | 15.25 (8.53-26.10) | 29.47 (18.37-45.90) | 0.31 (0.17-0.53) | 0.19 (0.12-0.29) | -1.72 (-2.04,-1.40) |  | 527.20 (319.84-854.60) | 1191.52 (753.92-1832.52) | 10.60 (6.43-17.19) | 7.63 (4.83-11.74) | -1.21 (-1.53,-0.90) |
| Albania | 4.95 (3.15-7.28) | 5.30 (3.07-8.76) | 0.30 (0.19-0.44) | 0.40 (0.23-0.66) | 0.77 (0.31,1.23) |  | 5.27 (3.37-7.68) | 5.70 (3.29-9.53) | 0.32 (0.20-0.46) | 0.43 (0.25-0.71) | 0.75 (0.23,1.27) |  | 162.44 (106.78-232.68) | 138.14 (82.60-225.83) | 9.83 (6.46-14.08) | 10.36 (6.19-16.93) | -0.09 (-0.56,0.38) |
| Algeria | 5.62 (3.72-8.21) | 19.32 (12.04-28.75) | 0.04 (0.03-0.06) | 0.09 (0.05-0.13) | 2.18 (2.08,2.28) |  | 5.80 (3.85-8.48) | 19.45 (12.17-28.89) | 0.05 (0.03-0.07) | 0.09 (0.06-0.13) | 2.08 (1.98,2.17) |  | 209.05 (139.72-301.16) | 606.80 (381.59-890.10) | 1.65 (1.10-2.38) | 2.75 (1.73-4.03) | 1.64 (1.55,1.73) |
| American Samoa | 0.02 (0.01-0.03) | 0.06 (0.04-0.09) | 0.09 (0.06-0.14) | 0.24 (0.15-0.37) | 3.42 (3.21,3.63) |  | 0.02 (0.01-0.03) | 0.06 (0.04-0.09) | 0.09 (0.06-0.14) | 0.24 (0.15-0.37) | 3.49 (3.24,3.74) |  | 0.79 (0.50-1.21) | 1.97 (1.22-3.00) | 3.24 (2.07-5.01) | 7.92 (4.90-12.05) | 3.12 (2.87,3.38) |
| Andorra | 0.15 (0.09-0.23) | 0.40 (0.23-0.60) | 0.55 (0.32-0.85) | 0.93 (0.54-1.41) | 1.79 (1.66,1.93) |  | 0.14 (0.08-0.22) | 0.36 (0.21-0.56) | 0.52 (0.31-0.82) | 0.85 (0.50-1.31) | 1.72 (1.56,1.88) |  | 4.12 (2.49-6.37) | 9.01 (5.16-13.83) | 15.16 (9.15-23.44) | 21.06 (12.06-32.31) | 1.16 (1.00,1.32) |
| Angola | 9.81 (1.65-29.05) | 19.62 (4.99-50.35) | 0.19 (0.03-0.57) | 0.12 (0.03-0.31) | -1.85 (-2.06,-1.64) |  | 9.90 (1.67-28.80) | 19.83 (4.97-51.43) | 0.19 (0.03-0.56) | 0.12 (0.03-0.31) | -1.80 (-2.01,-1.59) |  | 390.48 (65.16-1137.35) | 778.33 (198.17-2087.34) | 7.60 (1.27-22.14) | 4.76 (1.21-12.76) | -1.75 (-1.93,-1.56) |
| Antigua and Barbuda | 0.04 (0.03-0.06) | 0.06 (0.04-0.09) | 0.14 (0.09-0.19) | 0.14 (0.10-0.19) | -0.14 (-0.69,0.41) |  | 0.04 (0.03-0.06) | 0.06 (0.04-0.09) | 0.14 (0.10-0.20) | 0.14 (0.10-0.20) | -0.27 (-0.82,0.28) |  | 1.19 (0.86-1.61) | 1.76 (1.24-2.43) | 3.94 (2.86-5.35) | 3.93 (2.78-5.43) | -0.28 (-0.78,0.23) |
| Argentina | 4.65 (3.18-6.64) | 16.67 (12.00-22.59) | 0.03 (0.02-0.04) | 0.07 (0.05-0.10) | 3.73 (3.46,4.00) |  | 4.84 (3.30-6.88) | 17.20 (12.35-23.16) | 0.03 (0.02-0.04) | 0.08 (0.05-0.10) | 3.75 (3.38,4.11) |  | 140.76 (98.67-197.49) | 476.14 (344.99-640.72) | 0.85 (0.60-1.19) | 2.09 (1.52-2.82) | 3.56 (3.21,3.91) |
| Armenia | 3.64 (2.45-5.46) | 4.01 (2.60-5.86) | 0.21 (0.14-0.32) | 0.27 (0.17-0.39) | 0.97 (0.46,1.48) |  | 3.83 (2.57-5.73) | 4.28 (2.78-6.28) | 0.22 (0.15-0.34) | 0.29 (0.19-0.42) | 0.75 (0.14,1.37) |  | 112.10 (76.97-167.29) | 109.88 (72.89-157.80) | 6.55 (4.50-9.78) | 7.34 (4.87-10.54) | 0.34 (-0.19,0.88) |
| Australia | 7.55 (5.25-10.17) | 52.00 (36.60-71.23) | 0.09 (0.06-0.12) | 0.40 (0.28-0.55) | 5.11 (4.94,5.29) |  | 7.45 (5.21-10.04) | 46.90 (33.30-64.06) | 0.09 (0.06-0.12) | 0.36 (0.26-0.50) | 4.64 (4.51,4.77) |  | 220.55 (158.86-294.13) | 1175.98 (825.01-1609.81) | 2.62 (1.88-3.49) | 9.12 (6.40-12.48) | 4.17 (4.07,4.28) |
| Austria | 5.13 (3.64-6.96) | 14.73 (10.15-20.44) | 0.13 (0.09-0.18) | 0.33 (0.23-0.46) | 3.14 (2.82,3.47) |  | 4.96 (3.51-6.79) | 12.56 (8.46-17.75) | 0.13 (0.09-0.17) | 0.28 (0.19-0.40) | 2.66 (2.40,2.91) |  | 129.30 (94.00-172.07) | 288.35 (203.94-390.72) | 3.33 (2.42-4.43) | 6.42 (4.54-8.70) | 2.18 (1.94,2.42) |
| Azerbaijan | 5.80 (3.03-10.34) | 12.37 (5.96-22.13) | 0.16 (0.08-0.28) | 0.24 (0.11-0.42) | 0.87 (0.74,1.00) |  | 6.01 (3.16-10.78) | 12.75 (6.23-22.74) | 0.16 (0.09-0.29) | 0.24 (0.12-0.43) | 0.88 (0.73,1.03) |  | 198.50 (110.33-347.25) | 393.67 (198.70-714.44) | 5.42 (3.01-9.48) | 7.50 (3.78-13.61) | 0.52 (0.33,0.72) |
| Bahamas | 0.15 (0.10-0.20) | 0.29 (0.20-0.44) | 0.12 (0.08-0.16) | 0.15 (0.10-0.23) | 0.62 (0.35,0.90) |  | 0.15 (0.11-0.21) | 0.30 (0.20-0.44) | 0.12 (0.08-0.16) | 0.15 (0.10-0.23) | 0.66 (0.35,0.98) |  | 5.15 (3.69-6.90) | 9.36 (6.40-14.18) | 4.01 (2.88-5.37) | 4.82 (3.30-7.31) | 0.37 (0.07,0.67) |
| Bahrain | 0.37 (0.24-0.55) | 1.02 (0.63-1.56) | 0.15 (0.10-0.22) | 0.13 (0.08-0.20) | -1.47 (-2.03,-0.90) |  | 0.38 (0.25-0.55) | 0.99 (0.61-1.50) | 0.15 (0.10-0.22) | 0.13 (0.08-0.20) | -1.73 (-2.35,-1.11) |  | 12.40 (8.45-17.65) | 31.00 (19.48-46.63) | 4.90 (3.34-6.97) | 4.05 (2.55-6.10) | -1.67 (-2.24,-1.10) |
| Bangladesh | 15.35 (10.14-22.46) | 41.27 (25.95-62.04) | 0.03 (0.02-0.04) | 0.05 (0.03-0.08) | 2.10 (1.92,2.27) |  | 15.81 (10.50-23.06) | 42.64 (26.63-63.92) | 0.03 (0.02-0.04) | 0.05 (0.03-0.08) | 2.17 (2.00,2.35) |  | 616.15 (411.84-882.81) | 1426.35 (893.00-2165.27) | 1.13 (0.75-1.62) | 1.73 (1.08-2.63) | 1.65 (1.51,1.80) |
| Barbados | 0.16 (0.11-0.22) | 0.28 (0.18-0.42) | 0.13 (0.08-0.18) | 0.19 (0.12-0.28) | 1.20 (1.06,1.35) |  | 0.17 (0.11-0.24) | 0.30 (0.19-0.44) | 0.14 (0.09-0.19) | 0.20 (0.13-0.30) | 1.14 (0.94,1.33) |  | 4.46 (3.14-5.94) | 7.30 (4.69-10.87) | 3.52 (2.48-4.69) | 4.89 (3.14-7.27) | 0.97 (0.80,1.15) |
| Belarus | 4.97 (3.32-7.28) | 5.93 (3.76-8.95) | 0.10 (0.06-0.14) | 0.13 (0.08-0.19) | 0.61 (0.41,0.82) |  | 5.20 (3.48-7.56) | 6.13 (3.92-9.15) | 0.10 (0.07-0.14) | 0.13 (0.08-0.20) | 0.57 (0.35,0.80) |  | 146.01 (98.85-213.31) | 169.84 (107.59-258.76) | 2.80 (1.89-4.08) | 3.64 (2.31-5.55) | 0.51 (0.29,0.73) |
| Belgium | 8.43 (5.57-11.97) | 17.38 (11.58-24.88) | 0.17 (0.11-0.24) | 0.30 (0.20-0.43) | 1.91 (1.56,2.27) |  | 8.73 (5.70-12.36) | 16.88 (10.97-24.22) | 0.18 (0.11-0.25) | 0.29 (0.19-0.42) | 1.77 (1.30,2.25) |  | 206.01 (143.42-285.13) | 378.16 (261.40-527.68) | 4.13 (2.87-5.71) | 6.59 (4.56-9.20) | 1.61 (1.24,1.97) |
| Belize | 0.06 (0.04-0.08) | 0.21 (0.15-0.28) | 0.06 (0.04-0.08) | 0.10 (0.07-0.13) | 0.85 (0.56,1.15) |  | 0.06 (0.04-0.08) | 0.21 (0.15-0.28) | 0.06 (0.04-0.09) | 0.10 (0.07-0.13) | 0.78 (0.37,1.19) |  | 1.90 (1.39-2.61) | 7.09 (5.15-9.46) | 2.03 (1.48-2.79) | 3.30 (2.40-4.41) | 0.93 (0.52,1.34) |
| Benin | 9.37 (4.79-17.33) | 17.24 (10.70-26.61) | 0.39 (0.20-0.71) | 0.26 (0.16-0.39) | -1.68 (-1.85,-1.52) |  | 9.67 (5.02-17.92) | 17.59 (10.96-27.40) | 0.40 (0.21-0.74) | 0.26 (0.16-0.41) | -1.66 (-1.82,-1.49) |  | 329.94 (167.50-603.07) | 622.62 (394.63-992.58) | 13.60 (6.91-24.87) | 9.22 (5.85-14.71) | -1.57 (-1.76,-1.38) |
| Bermuda | 0.04 (0.03-0.06) | 0.04 (0.03-0.06) | 0.14 (0.09-0.19) | 0.12 (0.08-0.18) | -0.35 (-0.82,0.11) |  | 0.04 (0.03-0.06) | 0.04 (0.03-0.06) | 0.14 (0.10-0.20) | 0.13 (0.08-0.18) | -0.54 (-1.05,-0.03) |  | 1.15 (0.82-1.55) | 0.91 (0.61-1.30) | 3.87 (2.76-5.23) | 2.88 (1.93-4.09) | -1.09 (-1.59,-0.59) |
| Bhutan | 0.13 (0.08-0.21) | 0.34 (0.19-0.55) | 0.04 (0.02-0.07) | 0.09 (0.05-0.15) | 2.37 (2.26,2.48) |  | 0.13 (0.08-0.21) | 0.35 (0.20-0.57) | 0.04 (0.03-0.07) | 0.09 (0.05-0.15) | 2.48 (2.34,2.61) |  | 5.22 (3.10-8.28) | 11.21 (6.35-18.06) | 1.66 (0.98-2.63) | 2.96 (1.68-4.77) | 1.82 (1.67,1.97) |
| Bolivia (Plurinational State of) | 3.71 (2.27-5.87) | 9.67 (5.22-15.43) | 0.12 (0.07-0.18) | 0.16 (0.09-0.26) | 1.21 (1.11,1.31) |  | 3.90 (2.39-6.13) | 10.28 (5.61-16.40) | 0.12 (0.07-0.19) | 0.17 (0.10-0.28) | 1.28 (1.18,1.39) |  | 126.27 (76.87-197.02) | 286.34 (163.16-457.35) | 3.96 (2.41-6.18) | 4.85 (2.77-7.75) | 0.67 (0.55,0.79) |
| Bosnia and Herzegovina | 4.89 (3.26-7.15) | 5.54 (3.39-8.47) | 0.22 (0.14-0.32) | 0.34 (0.21-0.51) | 1.09 (0.93,1.25) |  | 5.08 (3.38-7.48) | 5.94 (3.62-9.06) | 0.23 (0.15-0.33) | 0.36 (0.22-0.55) | 1.15 (1.01,1.29) |  | 153.36 (102.90-224.09) | 138.98 (87.04-212.50) | 6.82 (4.57-9.96) | 8.42 (5.27-12.87) | 0.20 (0.01,0.39) |
| Botswana | 0.82 (0.37-1.75) | 2.12 (1.05-4.26) | 0.12 (0.06-0.27) | 0.18 (0.09-0.36) | 0.67 (0.42,0.92) |  | 0.83 (0.38-1.78) | 2.14 (1.07-4.25) | 0.13 (0.06-0.27) | 0.18 (0.09-0.36) | 0.65 (0.38,0.93) |  | 30.29 (13.50-65.67) | 76.78 (36.27-156.72) | 4.59 (2.05-9.96) | 6.42 (3.03-13.10) | 0.62 (0.33,0.92) |
| Brazil | 41.03 (35.97-47.18) | 103.25 (88.42-120.26) | 0.06 (0.05-0.06) | 0.09 (0.08-0.11) | 2.33 (2.07,2.59) |  | 42.19 (37.01-48.24) | 107.06 (91.73-125.19) | 0.06 (0.05-0.06) | 0.10 (0.08-0.11) | 2.45 (2.19,2.70) |  | 1477.97 (1303.31-1668.84) | 3131.83 (2716.99-3603.81) | 1.99 (1.76-2.25) | 2.84 (2.47-3.27) | 1.87 (1.62,2.12) |
| Brunei Darussalam | 0.27 (0.16-0.42) | 0.66 (0.41-1.05) | 0.21 (0.13-0.32) | 0.29 (0.18-0.46) | 0.92 (0.74,1.11) |  | 0.27 (0.16-0.42) | 0.63 (0.39-0.98) | 0.21 (0.13-0.32) | 0.28 (0.17-0.43) | 0.74 (0.51,0.96) |  | 8.33 (5.12-12.98) | 18.65 (11.81-30.22) | 6.43 (3.95-10.01) | 8.27 (5.23-13.40) | 0.56 (0.36,0.77) |
| Bulgaria | 13.92 (9.10-20.08) | 6.09 (3.81-9.47) | 0.32 (0.21-0.46) | 0.18 (0.11-0.28) | -1.65 (-2.09,-1.21) |  | 14.67 (9.55-21.16) | 6.52 (4.03-10.05) | 0.34 (0.22-0.49) | 0.19 (0.12-0.30) | -1.55 (-2.09,-1.01) |  | 403.14 (274.13-570.58) | 161.65 (103.63-244.03) | 9.29 (6.32-13.15) | 4.76 (3.05-7.19) | -1.87 (-2.37,-1.36) |
| Burkina Faso | 23.57 (10.20-50.69) | 39.36 (17.72-70.85) | 0.49 (0.21-1.06) | 0.35 (0.16-0.62) | -1.36 (-1.56,-1.16) |  | 24.15 (10.54-51.72) | 40.08 (17.99-72.75) | 0.51 (0.22-1.09) | 0.35 (0.16-0.64) | -1.39 (-1.60,-1.19) |  | 818.91 (358.40-1784.39) | 1432.32 (636.71-2612.05) | 17.19 (7.52-37.46) | 12.59 (5.59-22.95) | -1.19 (-1.42,-0.97) |
| Burundi | 3.77 (2.22-6.19) | 5.48 (3.18-9.05) | 0.14 (0.08-0.22) | 0.08 (0.05-0.14) | -2.07 (-2.30,-1.85) |  | 3.88 (2.30-6.35) | 5.60 (3.25-9.34) | 0.14 (0.08-0.23) | 0.08 (0.05-0.14) | -2.08 (-2.30,-1.85) |  | 149.62 (91.12-243.06) | 226.82 (133.50-380.37) | 5.39 (3.28-8.75) | 3.43 (2.02-5.75) | -1.81 (-2.00,-1.62) |
| Cabo Verde | 0.58 (0.36-0.99) | 1.26 (0.74-1.90) | 0.33 (0.20-0.56) | 0.45 (0.26-0.68) | 0.92 (0.75,1.10) |  | 0.62 (0.37-1.06) | 1.31 (0.76-1.99) | 0.35 (0.21-0.60) | 0.47 (0.27-0.71) | 0.84 (0.64,1.03) |  | 17.86 (11.47-29.24) | 38.41 (23.29-58.50) | 10.09 (6.48-16.53) | 13.74 (8.33-20.92) | 0.87 (0.74,1.01) |
| Cambodia | 6.74 (3.25-13.05) | 12.41 (5.97-23.92) | 0.13 (0.06-0.25) | 0.15 (0.07-0.28) | 0.34 (0.24,0.44) |  | 6.85 (3.31-13.28) | 12.54 (6.01-24.12) | 0.13 (0.06-0.26) | 0.15 (0.07-0.28) | 0.31 (0.22,0.40) |  | 247.59 (125.17-486.86) | 406.97 (198.46-804.74) | 4.82 (2.44-9.48) | 4.78 (2.33-9.44) | -0.10 (-0.17,-0.02) |
| Cameroon | 20.30 (12.47-31.99) | 39.45 (19.61-67.93) | 0.39 (0.24-0.61) | 0.25 (0.12-0.43) | -1.78 (-1.95,-1.61) |  | 20.64 (12.60-32.34) | 39.75 (20.07-68.58) | 0.40 (0.24-0.62) | 0.25 (0.13-0.43) | -1.79 (-1.97,-1.62) |  | 735.60 (461.00-1144.25) | 1460.58 (722.86-2543.31) | 14.10 (8.83-21.93) | 9.19 (4.55-16.01) | -1.69 (-1.87,-1.51) |
| Canada | 18.19 (13.20-24.74) | 81.83 (56.54-114.43) | 0.13 (0.10-0.18) | 0.44 (0.30-0.61) | 4.11 (4.03,4.19) |  | 16.88 (12.16-23.18) | 73.22 (49.86-104.42) | 0.12 (0.09-0.17) | 0.39 (0.27-0.56) | 4.15 (4.05,4.25) |  | 480.23 (358.91-647.80) | 1739.46 (1231.83-2421.15) | 3.52 (2.63-4.75) | 9.28 (6.58-12.92) | 3.54 (3.43,3.64) |
| Central African Republic | 2.68 (1.04-5.70) | 3.44 (1.44-7.92) | 0.20 (0.08-0.42) | 0.13 (0.05-0.29) | -1.85 (-2.00,-1.70) |  | 2.69 (1.05-5.75) | 3.45 (1.45-7.97) | 0.20 (0.08-0.42) | 0.13 (0.05-0.29) | -1.91 (-2.08,-1.74) |  | 102.62 (41.76-221.02) | 136.33 (56.75-302.24) | 7.52 (3.06-16.19) | 4.97 (2.07-11.02) | -1.79 (-1.96,-1.63) |
| Chad | 9.73 (4.13-20.52) | 19.33 (10.39-36.56) | 0.32 (0.14-0.68) | 0.22 (0.12-0.41) | -1.62 (-1.79,-1.46) |  | 10.07 (4.31-21.46) | 19.69 (10.56-37.38) | 0.33 (0.14-0.71) | 0.22 (0.12-0.42) | -1.66 (-1.84,-1.49) |  | 333.25 (140.83-690.74) | 709.91 (383.99-1337.31) | 11.06 (4.67-22.92) | 8.00 (4.33-15.07) | -1.37 (-1.55,-1.18) |
| Chile | 4.29 (2.88-6.23) | 20.20 (13.55-28.06) | 0.06 (0.04-0.09) | 0.21 (0.14-0.30) | 4.45 (4.10,4.80) |  | 4.48 (3.00-6.53) | 20.56 (13.60-29.14) | 0.07 (0.05-0.10) | 0.22 (0.14-0.31) | 4.44 (4.14,4.74) |  | 123.97 (85.95-178.25) | 498.63 (344.59-688.91) | 1.87 (1.29-2.68) | 5.31 (3.67-7.33) | 4.00 (3.72,4.27) |
| China | 2533.88 (2008.42-3152.38) | 4617.60 (3517.24-5937.32) | 0.43 (0.34-0.54) | 0.65 (0.49-0.83) | 1.48 (1.32,1.64) |  | 2490.67 (1978.66-3092.58) | 4016.74 (3051.07-5115.41) | 0.42 (0.34-0.53) | 0.56 (0.43-0.72) | 1.06 (0.91,1.22) |  | 90464.03 (72743.17-112527.94) | 118708.10 (90374.17-155229.66) | 15.38 (12.37-19.13) | 16.69 (12.70-21.82) | 0.25 (0.06,0.43) |
| Colombia | 15.09 (10.80-20.76) | 31.34 (21.12-43.02) | 0.09 (0.07-0.13) | 0.13 (0.09-0.18) | 1.23 (0.64,1.82) |  | 15.71 (11.28-21.57) | 33.15 (22.12-45.93) | 0.10 (0.07-0.13) | 0.14 (0.09-0.19) | 1.26 (0.67,1.85) |  | 523.70 (393.16-690.42) | 864.77 (602.23-1170.81) | 3.22 (2.42-4.25) | 3.53 (2.46-4.77) | 0.39 (-0.18,0.97) |
| Comoros | 0.40 (0.22-0.62) | 0.77 (0.45-1.16) | 0.17 (0.10-0.27) | 0.21 (0.12-0.31) | 0.19 (-0.05,0.44) |  | 0.41 (0.23-0.63) | 0.80 (0.46-1.22) | 0.18 (0.10-0.27) | 0.21 (0.12-0.33) | 0.20 (-0.04,0.43) |  | 14.86 (8.28-22.83) | 27.21 (16.14-41.12) | 6.43 (3.58-9.87) | 7.31 (4.34-11.05) | -0.10 (-0.43,0.25) |
| Congo | 2.72 (1.12-6.11) | 4.28 (1.83-9.50) | 0.23 (0.09-0.51) | 0.16 (0.07-0.35) | -1.66 (-1.89,-1.43) |  | 2.75 (1.13-6.23) | 4.28 (1.83-9.44) | 0.23 (0.09-0.52) | 0.16 (0.07-0.35) | -1.66 (-1.90,-1.42) |  | 101.60 (42.17-226.68) | 163.46 (68.42-367.93) | 8.46 (3.51-18.88) | 6.06 (2.54-13.65) | -1.54 (-1.78,-1.31) |
| Cook Islands | 0.03 (0.02-0.04) | 0.04 (0.02-0.06) | 0.27 (0.18-0.41) | 0.46 (0.28-0.70) | 1.87 (1.70,2.04) |  | 0.03 (0.02-0.04) | 0.04 (0.02-0.06) | 0.27 (0.18-0.41) | 0.44 (0.27-0.67) | 1.71 (1.55,1.87) |  | 0.87 (0.58-1.28) | 1.11 (0.68-1.76) | 9.20 (6.10-13.58) | 12.53 (7.72-19.87) | 1.25 (1.09,1.42) |
| Costa Rica | 2.17 (1.61-2.92) | 7.01 (4.72-9.60) | 0.14 (0.11-0.19) | 0.30 (0.20-0.40) | 2.18 (1.72,2.64) |  | 2.25 (1.65-3.06) | 7.24 (4.81-9.99) | 0.15 (0.11-0.20) | 0.30 (0.20-0.42) | 2.29 (1.80,2.79) |  | 71.69 (54.28-93.73) | 194.47 (136.94-265.06) | 4.71 (3.57-6.16) | 8.19 (5.77-11.17) | 1.68 (1.21,2.15) |
| Côte d'Ivoire | 6.33 (3.87-10.35) | 11.07 (5.92-17.46) | 0.10 (0.06-0.17) | 0.08 (0.04-0.13) | -1.09 (-1.21,-0.97) |  | 6.40 (3.97-10.37) | 11.21 (6.00-17.82) | 0.10 (0.07-0.17) | 0.08 (0.04-0.13) | -1.03 (-1.16,-0.91) |  | 243.64 (147.19-394.90) | 417.54 (228.33-660.27) | 3.99 (2.41-6.47) | 3.00 (1.64-4.74) | -1.06 (-1.20,-0.92) |
| Croatia | 2.98 (2.01-4.23) | 4.26 (2.62-6.17) | 0.12 (0.08-0.17) | 0.20 (0.12-0.29) | 2.17 (1.63,2.71) |  | 3.07 (2.08-4.36) | 4.18 (2.56-6.05) | 0.13 (0.09-0.18) | 0.20 (0.12-0.29) | 2.09 (1.56,2.63) |  | 81.72 (56.14-115.07) | 89.31 (56.96-129.59) | 3.36 (2.31-4.73) | 4.24 (2.71-6.16) | 1.28 (0.76,1.81) |
| Cuba | 6.13 (4.38-8.17) | 7.12 (4.82-10.05) | 0.11 (0.08-0.15) | 0.13 (0.09-0.18) | -0.22 (-0.60,0.15) |  | 6.40 (4.52-8.56) | 7.33 (4.94-10.24) | 0.12 (0.08-0.16) | 0.13 (0.09-0.18) | -0.29 (-0.78,0.21) |  | 184.03 (136.55-243.70) | 190.69 (130.55-268.30) | 3.39 (2.52-4.49) | 3.38 (2.32-4.76) | -0.59 (-1.06,-0.13) |
| Cyprus | 0.56 (0.35-0.83) | 1.40 (0.88-2.17) | 0.14 (0.09-0.21) | 0.21 (0.13-0.32) | 1.28 (1.22,1.35) |  | 0.58 (0.36-0.86) | 1.34 (0.85-2.05) | 0.15 (0.09-0.22) | 0.20 (0.13-0.30) | 0.95 (0.87,1.04) |  | 15.02 (9.65-22.24) | 30.97 (19.57-46.70) | 3.86 (2.48-5.72) | 4.56 (2.88-6.88) | 0.51 (0.46,0.57) |
| Czechia | 8.80 (6.07-12.30) | 6.39 (4.00-9.24) | 0.17 (0.12-0.24) | 0.12 (0.08-0.17) | -1.26 (-1.47,-1.05) |  | 9.39 (6.47-13.29) | 6.72 (4.21-9.64) | 0.18 (0.13-0.26) | 0.13 (0.08-0.18) | -1.33 (-1.59,-1.07) |  | 244.82 (172.92-336.92) | 149.12 (94.87-216.41) | 4.76 (3.36-6.55) | 2.81 (1.78-4.07) | -1.86 (-2.11,-1.60) |
| Democratic People's Republic of Korea | 43.08 (21.90-81.37) | 60.01 (34.96-95.61) | 0.42 (0.21-0.79) | 0.45 (0.26-0.72) | 0.13 (0.07,0.18) |  | 42.82 (21.89-80.47) | 58.34 (34.07-92.34) | 0.42 (0.21-0.78) | 0.44 (0.26-0.70) | 0.07 (0.02,0.13) |  | 1523.68 (762.31-2843.07) | 1880.31 (1094.34-3021.64) | 14.80 (7.40-27.61) | 14.25 (8.29-22.90) | -0.27 (-0.34,-0.21) |
| Democratic Republic of the Congo | 15.82 (7.71-33.07) | 30.24 (13.24-69.76) | 0.08 (0.04-0.17) | 0.07 (0.03-0.15) | -0.92 (-1.09,-0.75) |  | 16.08 (7.84-33.42) | 30.74 (13.57-71.03) | 0.08 (0.04-0.18) | 0.07 (0.03-0.16) | -0.94 (-1.11,-0.78) |  | 615.28 (316.85-1253.83) | 1170.11 (519.94-2676.79) | 3.23 (1.66-6.57) | 2.60 (1.16-5.95) | -0.91 (-1.09,-0.74) |
| Denmark | 3.02 (2.10-4.11) | 7.27 (4.68-10.36) | 0.12 (0.08-0.16) | 0.25 (0.16-0.35) | 2.16 (1.91,2.41) |  | 2.47 (1.68-3.47) | 6.45 (4.09-9.18) | 0.10 (0.07-0.13) | 0.22 (0.14-0.31) | 2.55 (2.24,2.85) |  | 63.56 (45.25-85.62) | 139.76 (91.88-195.93) | 2.47 (1.76-3.33) | 4.78 (3.14-6.70) | 2.02 (1.69,2.35) |
| Djibouti | 0.20 (0.12-0.34) | 0.94 (0.55-1.56) | 0.10 (0.06-0.16) | 0.15 (0.09-0.25) | 1.16 (0.98,1.34) |  | 0.20 (0.12-0.34) | 0.95 (0.55-1.56) | 0.10 (0.06-0.17) | 0.15 (0.09-0.25) | 1.26 (1.03,1.50) |  | 8.09 (4.61-13.70) | 34.26 (19.44-56.18) | 3.90 (2.23-6.62) | 5.44 (3.09-8.93) | 0.99 (0.71,1.27) |
| Dominica | 0.04 (0.02-0.06) | 0.06 (0.04-0.10) | 0.11 (0.07-0.17) | 0.19 (0.12-0.29) | 1.47 (1.33,1.60) |  | 0.04 (0.03-0.07) | 0.07 (0.04-0.10) | 0.12 (0.07-0.19) | 0.21 (0.13-0.30) | 1.51 (1.32,1.69) |  | 1.16 (0.74-1.76) | 1.79 (1.16-2.67) | 3.19 (2.06-4.87) | 5.35 (3.46-7.95) | 1.46 (1.27,1.64) |
| Dominican Republic | 1.64 (1.12-2.34) | 5.06 (3.27-7.56) | 0.05 (0.03-0.07) | 0.09 (0.06-0.14) | 2.57 (2.44,2.70) |  | 1.70 (1.16-2.38) | 5.24 (3.36-7.85) | 0.05 (0.03-0.07) | 0.10 (0.06-0.14) | 2.57 (2.44,2.71) |  | 60.46 (41.44-85.19) | 163.18 (106.18-243.82) | 1.69 (1.16-2.38) | 2.96 (1.93-4.43) | 2.10 (1.93,2.28) |
| Ecuador | 6.99 (5.04-9.34) | 13.84 (9.20-20.23) | 0.14 (0.10-0.19) | 0.15 (0.10-0.22) | 0.47 (-0.22,1.17) |  | 7.37 (5.25-9.84) | 14.86 (9.78-21.61) | 0.15 (0.11-0.20) | 0.16 (0.11-0.24) | 0.34 (-0.38,1.07) |  | 242.89 (175.36-318.21) | 393.36 (270.48-563.82) | 4.87 (3.51-6.38) | 4.35 (2.99-6.24) | -0.51 (-1.24,0.23) |
| Egypt | 112.36 (66.24-197.36) | 340.10 (215.40-513.00) | 0.41 (0.24-0.71) | 0.64 (0.41-0.97) | 1.65 (1.57,1.73) |  | 114.70 (67.95-201.80) | 340.51 (214.63-511.18) | 0.41 (0.25-0.73) | 0.64 (0.41-0.97) | 1.52 (1.41,1.63) |  | 4069.42 (2536.81-6756.52) | 11152.30 (7418.63-16556.54) | 14.71 (9.17-24.42) | 21.12 (14.05-31.35) | 1.32 (1.23,1.40) |
| El Salvador | 1.72 (1.18-2.49) | 2.69 (1.78-3.89) | 0.06 (0.04-0.09) | 0.08 (0.06-0.12) | 1.22 (0.76,1.69) |  | 1.80 (1.24-2.59) | 2.80 (1.86-4.00) | 0.07 (0.05-0.10) | 0.09 (0.06-0.12) | 1.04 (0.55,1.53) |  | 61.18 (44.50-85.09) | 83.94 (56.14-119.06) | 2.31 (1.68-3.21) | 2.60 (1.74-3.69) | 0.69 (0.20,1.18) |
| Equatorial Guinea | 0.17 (0.09-0.34) | 0.60 (0.35-1.02) | 0.08 (0.04-0.16) | 0.08 (0.05-0.14) | 0.05 (-0.32,0.42) |  | 0.17 (0.09-0.35) | 0.60 (0.35-1.00) | 0.08 (0.04-0.16) | 0.08 (0.05-0.13) | 0.04 (-0.33,0.42) |  | 6.43 (3.37-12.87) | 23.87 (13.27-40.25) | 3.04 (1.59-6.09) | 3.16 (1.76-5.32) | 0.14 (-0.20,0.49) |
| Eritrea | 1.81 (1.10-2.88) | 4.21 (2.49-7.19) | 0.11 (0.06-0.17) | 0.13 (0.08-0.22) | 0.14 (0.03,0.26) |  | 1.82 (1.11-2.90) | 4.27 (2.55-7.48) | 0.11 (0.07-0.17) | 0.13 (0.08-0.23) | 0.20 (0.06,0.33) |  | 71.67 (44.22-109.84) | 163.07 (97.28-277.57) | 4.21 (2.60-6.45) | 4.94 (2.95-8.41) | 0.12 (-0.02,0.26) |
| Estonia | 1.00 (0.68-1.40) | 1.26 (0.82-1.82) | 0.13 (0.09-0.18) | 0.19 (0.13-0.28) | 0.64 (0.33,0.96) |  | 1.05 (0.71-1.47) | 1.34 (0.88-1.92) | 0.13 (0.09-0.19) | 0.20 (0.13-0.29) | 0.86 (0.55,1.18) |  | 29.33 (20.22-40.96) | 31.08 (20.90-44.07) | 3.74 (2.58-5.22) | 4.74 (3.19-6.72) | 0.10 (-0.23,0.44) |
| Eswatini | 0.72 (0.32-1.26) | 2.59 (1.01-5.46) | 0.18 (0.08-0.31) | 0.45 (0.17-0.95) | 3.18 (2.10,4.26) |  | 0.74 (0.33-1.26) | 2.58 (1.02-5.36) | 0.18 (0.08-0.31) | 0.45 (0.18-0.93) | 3.16 (2.06,4.27) |  | 27.39 (12.05-49.82) | 98.53 (37.37-218.58) | 6.79 (2.99-12.36) | 17.06 (6.47-37.84) | 3.25 (2.09,4.42) |
| Ethiopia | 23.91 (17.26-33.11) | 34.57 (22.74-55.37) | 0.09 (0.07-0.13) | 0.06 (0.04-0.10) | -1.92 (-2.18,-1.66) |  | 24.46 (17.78-33.86) | 35.68 (23.44-57.70) | 0.10 (0.07-0.13) | 0.07 (0.04-0.11) | -1.86 (-2.12,-1.60) |  | 948.16 (666.82-1323.93) | 1344.48 (877.27-2096.80) | 3.75 (2.64-5.24) | 2.47 (1.61-3.85) | -1.98 (-2.23,-1.73) |
| Fiji | 0.36 (0.21-0.61) | 0.76 (0.43-1.18) | 0.10 (0.06-0.16) | 0.16 (0.09-0.26) | 2.17 (1.96,2.39) |  | 0.36 (0.21-0.60) | 0.75 (0.43-1.15) | 0.10 (0.06-0.16) | 0.16 (0.09-0.25) | 2.23 (1.97,2.50) |  | 13.77 (8.18-22.81) | 25.58 (15.25-40.03) | 3.63 (2.16-6.01) | 5.53 (3.30-8.66) | 1.84 (1.57,2.11) |
| Finland | 5.68 (3.90-7.94) | 13.60 (9.02-19.10) | 0.23 (0.16-0.32) | 0.49 (0.33-0.69) | 2.75 (2.66,2.83) |  | 4.97 (3.34-7.06) | 10.58 (7.03-14.95) | 0.20 (0.13-0.28) | 0.38 (0.25-0.54) | 2.34 (2.14,2.55) |  | 126.65 (89.90-171.46) | 217.68 (150.85-301.93) | 5.06 (3.59-6.84) | 7.86 (5.45-10.91) | 1.66 (1.49,1.83) |
| France | 71.77 (49.16-102.48) | 177.25 (115.37-243.88) | 0.25 (0.17-0.35) | 0.53 (0.35-0.73) | 2.42 (2.21,2.64) |  | 72.88 (49.42-103.32) | 158.62 (100.57-220.07) | 0.25 (0.17-0.36) | 0.48 (0.30-0.66) | 1.91 (1.67,2.15) |  | 1871.88 (1336.26-2578.03) | 3516.48 (2373.26-4877.40) | 6.48 (4.63-8.92) | 10.59 (7.15-14.69) | 1.46 (1.21,1.71) |
| Gabon | 1.00 (0.41-2.15) | 1.80 (0.96-3.13) | 0.20 (0.08-0.44) | 0.20 (0.11-0.34) | -0.41 (-0.55,-0.28) |  | 1.03 (0.42-2.23) | 1.81 (0.97-3.15) | 0.21 (0.09-0.45) | 0.20 (0.11-0.35) | -0.41 (-0.57,-0.26) |  | 35.34 (14.41-75.82) | 64.15 (33.48-114.69) | 7.19 (2.93-15.42) | 7.07 (3.69-12.63) | -0.31 (-0.47,-0.14) |
| Gambia | 2.43 (1.61-3.66) | 8.26 (4.58-13.07) | 0.49 (0.33-0.75) | 0.69 (0.38-1.09) | 0.66 (0.44,0.88) |  | 2.46 (1.62-3.68) | 8.34 (4.61-13.18) | 0.50 (0.33-0.75) | 0.70 (0.39-1.10) | 0.74 (0.50,0.98) |  | 90.99 (61.16-136.56) | 306.95 (175.91-481.94) | 18.54 (12.46-27.82) | 25.64 (14.70-40.26) | 0.66 (0.39,0.93) |
| Georgia | 4.46 (3.02-6.58) | 2.95 (1.96-4.13) | 0.16 (0.11-0.24) | 0.16 (0.11-0.23) | -0.37 (-1.40,0.67) |  | 4.62 (3.11-6.74) | 3.14 (2.08-4.38) | 0.17 (0.11-0.24) | 0.17 (0.12-0.24) | -0.37 (-1.46,0.73) |  | 137.90 (95.29-202.04) | 82.80 (55.72-115.37) | 4.99 (3.45-7.32) | 4.59 (3.09-6.40) | -0.75 (-1.82,0.32) |
| Germany | 92.15 (64.95-126.62) | 220.93 (153.46-309.20) | 0.23 (0.16-0.32) | 0.52 (0.36-0.72) | 3.00 (2.71,3.30) |  | 93.60 (65.09-130.78) | 194.77 (133.93-279.30) | 0.23 (0.16-0.33) | 0.46 (0.31-0.65) | 2.62 (2.39,2.85) |  | 2231.75 (1624.93-2985.18) | 4265.33 (2999.26-5797.93) | 5.58 (4.07-7.47) | 9.99 (7.03-13.58) | 2.29 (2.05,2.53) |
| Ghana | 18.13 (10.13-31.32) | 31.43 (17.79-49.98) | 0.24 (0.14-0.42) | 0.18 (0.10-0.29) | -1.50 (-1.86,-1.15) |  | 18.30 (10.27-31.73) | 31.82 (17.78-49.99) | 0.24 (0.14-0.42) | 0.19 (0.10-0.29) | -1.50 (-1.84,-1.16) |  | 689.32 (385.62-1200.98) | 1146.53 (645.68-1837.85) | 9.21 (5.15-16.04) | 6.70 (3.77-10.73) | -1.63 (-1.97,-1.28) |
| Greece | 6.54 (4.52-8.82) | 19.05 (12.97-25.83) | 0.13 (0.09-0.17) | 0.37 (0.25-0.51) | 3.62 (3.37,3.87) |  | 6.64 (4.57-9.09) | 19.47 (13.19-26.74) | 0.13 (0.09-0.17) | 0.38 (0.26-0.53) | 3.77 (3.45,4.08) |  | 159.38 (115.75-211.22) | 404.00 (284.64-545.79) | 3.07 (2.23-4.07) | 7.94 (5.59-10.73) | 3.29 (2.97,3.61) |
| Greenland | 0.08 (0.06-0.12) | 0.14 (0.09-0.22) | 0.31 (0.21-0.43) | 0.50 (0.32-0.77) | 1.66 (1.52,1.80) |  | 0.08 (0.06-0.12) | 0.14 (0.09-0.21) | 0.30 (0.21-0.43) | 0.50 (0.32-0.77) | 1.71 (1.52,1.90) |  | 3.11 (2.18-4.40) | 4.06 (2.63-6.23) | 11.21 (7.85-15.84) | 14.49 (9.38-22.22) | 0.98 (0.83,1.12) |
| Grenada | 0.04 (0.03-0.06) | 0.10 (0.07-0.13) | 0.10 (0.07-0.15) | 0.19 (0.13-0.26) | 1.86 (1.34,2.39) |  | 0.05 (0.03-0.07) | 0.10 (0.07-0.14) | 0.11 (0.07-0.16) | 0.20 (0.14-0.27) | 1.79 (1.25,2.33) |  | 1.35 (0.91-1.88) | 2.77 (1.96-3.82) | 3.09 (2.09-4.33) | 5.41 (3.83-7.45) | 1.81 (1.40,2.23) |
| Guam | 0.05 (0.03-0.07) | 0.18 (0.11-0.26) | 0.07 (0.05-0.10) | 0.22 (0.14-0.32) | 4.32 (4.12,4.51) |  | 0.05 (0.03-0.07) | 0.17 (0.11-0.25) | 0.07 (0.05-0.10) | 0.22 (0.14-0.31) | 4.28 (4.07,4.50) |  | 1.65 (1.15-2.36) | 5.39 (3.50-7.87) | 2.41 (1.67-3.45) | 6.77 (4.39-9.88) | 3.97 (3.76,4.19) |
| Guatemala | 7.98 (5.91-10.78) | 16.34 (11.69-22.22) | 0.19 (0.14-0.26) | 0.21 (0.15-0.28) | 0.35 (-1.04,1.75) |  | 8.28 (6.12-11.24) | 17.04 (12.13-23.43) | 0.20 (0.15-0.27) | 0.22 (0.15-0.30) | -0.05 (-1.69,1.61) |  | 306.62 (238.87-397.91) | 554.09 (409.04-739.59) | 7.31 (5.70-9.49) | 7.03 (5.19-9.38) | -0.44 (-1.83,0.98) |
| Guinea | 19.48 (12.94-29.67) | 30.64 (17.80-48.55) | 0.65 (0.43-0.99) | 0.46 (0.27-0.72) | -1.09 (-1.26,-0.92) |  | 20.06 (13.33-30.36) | 31.18 (18.42-48.81) | 0.67 (0.44-1.01) | 0.46 (0.27-0.73) | -1.11 (-1.30,-0.93) |  | 680.00 (452.59-1016.29) | 1144.95 (665.89-1796.66) | 22.69 (15.10-33.91) | 17.05 (9.92-26.76) | -0.84 (-0.97,-0.71) |
| Guinea-Bissau | 3.01 (1.35-5.19) | 3.77 (2.37-5.57) | 0.60 (0.27-1.03) | 0.36 (0.23-0.54) | -1.88 (-1.98,-1.78) |  | 3.04 (1.36-5.25) | 3.77 (2.37-5.57) | 0.60 (0.27-1.04) | 0.36 (0.23-0.54) | -1.85 (-1.97,-1.74) |  | 111.58 (50.25-189.66) | 144.27 (89.90-217.85) | 22.16 (9.98-37.67) | 13.98 (8.71-21.11) | -1.74 (-1.86,-1.62) |
| Guyana | 0.30 (0.21-0.42) | 0.42 (0.27-0.62) | 0.08 (0.06-0.11) | 0.11 (0.07-0.16) | 1.11 (0.87,1.34) |  | 0.32 (0.22-0.43) | 0.43 (0.28-0.64) | 0.08 (0.06-0.11) | 0.11 (0.07-0.17) | 1.22 (0.93,1.50) |  | 10.63 (7.74-14.58) | 14.34 (9.20-20.92) | 2.73 (1.99-3.74) | 3.75 (2.41-5.47) | 1.15 (0.89,1.42) |
| Haiti | 2.03 (1.12-3.60) | 3.67 (1.88-7.19) | 0.06 (0.04-0.11) | 0.06 (0.03-0.11) | -0.35 (-0.43,-0.27) |  | 2.09 (1.16-3.74) | 3.79 (1.91-7.51) | 0.07 (0.04-0.12) | 0.06 (0.03-0.12) | -0.33 (-0.40,-0.26) |  | 74.61 (44.14-128.14) | 131.50 (69.04-260.99) | 2.34 (1.38-4.02) | 2.04 (1.07-4.06) | -0.40 (-0.48,-0.32) |
| Honduras | 1.49 (0.87-2.52) | 6.60 (3.89-9.98) | 0.06 (0.04-0.11) | 0.13 (0.08-0.20) | 2.44 (2.31,2.57) |  | 1.55 (0.90-2.67) | 6.91 (4.01-10.31) | 0.07 (0.04-0.11) | 0.14 (0.08-0.20) | 2.55 (2.38,2.73) |  | 57.93 (36.61-90.62) | 206.54 (123.31-315.66) | 2.46 (1.55-3.85) | 4.09 (2.44-6.24) | 1.74 (1.59,1.89) |
| Hungary | 6.11 (4.18-8.65) | 4.20 (2.63-6.06) | 0.12 (0.08-0.17) | 0.09 (0.05-0.13) | -1.43 (-2.07,-0.79) |  | 6.50 (4.36-9.18) | 4.45 (2.76-6.36) | 0.13 (0.08-0.18) | 0.09 (0.06-0.13) | -1.35 (-1.97,-0.72) |  | 176.68 (125.20-249.44) | 105.27 (68.55-150.81) | 3.40 (2.41-4.80) | 2.19 (1.43-3.14) | -1.78 (-2.33,-1.22) |
| Iceland | 0.17 (0.12-0.23) | 0.52 (0.34-0.72) | 0.13 (0.09-0.18) | 0.30 (0.19-0.41) | 2.39 (2.10,2.68) |  | 0.16 (0.11-0.23) | 0.47 (0.30-0.67) | 0.13 (0.09-0.18) | 0.27 (0.17-0.38) | 2.12 (1.78,2.46) |  | 4.29 (3.09-5.82) | 10.69 (7.21-14.92) | 3.38 (2.43-4.59) | 6.10 (4.12-8.52) | 1.64 (1.33,1.94) |
| India | 161.44 (134.37-194.54) | 513.86 (427.35-614.70) | 0.04 (0.03-0.05) | 0.07 (0.06-0.09) | 2.18 (2.12,2.24) |  | 165.27 (137.06-199.24) | 529.49 (440.64-634.48) | 0.04 (0.03-0.05) | 0.07 (0.06-0.09) | 2.15 (2.07,2.23) |  | 5865.59 (4933.51-6954.29) | 16568.53 (13718.45-19868.01) | 1.38 (1.16-1.63) | 2.34 (1.94-2.81) | 1.69 (1.61,1.76) |
| Indonesia | 67.03 (41.18-98.04) | 160.27 (88.07-253.51) | 0.07 (0.04-0.11) | 0.11 (0.06-0.18) | 1.56 (1.53,1.60) |  | 67.17 (41.18-98.60) | 159.22 (86.72-252.21) | 0.07 (0.04-0.11) | 0.11 (0.06-0.18) | 1.53 (1.49,1.57) |  | 2559.89 (1565.44-3744.57) | 5430.35 (3035.97-8634.09) | 2.77 (1.69-4.05) | 3.89 (2.18-6.19) | 1.16 (1.11,1.21) |
| Iran (Islamic Republic of) | 20.95 (16.29-27.44) | 70.14 (56.93-85.39) | 0.07 (0.06-0.10) | 0.16 (0.13-0.20) | 2.29 (1.59,2.99) |  | 21.75 (16.95-28.46) | 72.35 (58.75-87.93) | 0.08 (0.06-0.10) | 0.17 (0.14-0.21) | 2.26 (1.53,2.99) |  | 760.96 (599.01-980.67) | 2005.15 (1639.63-2436.95) | 2.67 (2.10-3.43) | 4.70 (3.84-5.71) | 1.40 (0.70,2.11) |
| Iraq | 10.84 (7.00-15.99) | 35.17 (21.23-55.09) | 0.12 (0.08-0.17) | 0.17 (0.10-0.27) | 1.46 (1.14,1.78) |  | 11.17 (7.19-16.89) | 35.24 (21.22-55.39) | 0.12 (0.08-0.18) | 0.17 (0.10-0.27) | 1.36 (1.01,1.71) |  | 389.55 (265.62-558.13) | 1125.74 (723.51-1736.96) | 4.23 (2.88-6.06) | 5.46 (3.51-8.43) | 1.07 (0.79,1.35) |
| Ireland | 1.77 (1.23-2.49) | 5.77 (3.80-8.19) | 0.10 (0.07-0.14) | 0.23 (0.15-0.33) | 3.16 (3.02,3.30) |  | 1.81 (1.24-2.56) | 5.37 (3.45-7.61) | 0.10 (0.07-0.14) | 0.22 (0.14-0.31) | 2.72 (2.51,2.93) |  | 46.17 (33.74-63.07) | 123.21 (84.78-171.59) | 2.56 (1.87-3.50) | 4.99 (3.43-6.94) | 2.30 (2.16,2.43) |
| Israel | 2.82 (1.91-3.92) | 7.39 (5.01-10.43) | 0.11 (0.08-0.16) | 0.15 (0.10-0.22) | 1.06 (0.92,1.20) |  | 2.96 (2.01-4.15) | 7.25 (4.85-10.27) | 0.12 (0.08-0.17) | 0.15 (0.10-0.21) | 0.78 (0.63,0.93) |  | 72.90 (51.66-99.19) | 171.76 (119.89-241.56) | 2.94 (2.08-4.00) | 3.58 (2.50-5.04) | 0.69 (0.52,0.86) |
| Italy | 69.53 (58.08-82.69) | 80.37 (66.15-96.09) | 0.24 (0.20-0.29) | 0.27 (0.22-0.32) | 0.10 (-0.13,0.33) |  | 67.23 (56.09-79.90) | 69.63 (56.29-84.20) | 0.24 (0.20-0.28) | 0.23 (0.19-0.28) | -0.42 (-0.75,-0.08) |  | 1694.53 (1433.12-2015.48) | 1508.71 (1250.35-1801.33) | 5.97 (5.05-7.10) | 5.04 (4.18-6.02) | -0.91 (-1.26,-0.56) |
| Jamaica | 0.57 (0.38-0.81) | 1.31 (0.85-1.94) | 0.05 (0.03-0.07) | 0.09 (0.06-0.14) | 2.15 (2.01,2.29) |  | 0.61 (0.40-0.87) | 1.37 (0.88-2.01) | 0.05 (0.03-0.07) | 0.10 (0.06-0.14) | 2.11 (1.75,2.47) |  | 16.93 (11.65-23.77) | 38.09 (23.98-56.61) | 1.43 (0.98-2.01) | 2.72 (1.71-4.04) | 2.09 (1.70,2.49) |
| Japan | 348.41 (296.04-408.48) | 472.41 (366.81-577.77) | 0.55 (0.47-0.65) | 0.74 (0.57-0.90) | -0.08 (-0.61,0.46) |  | 279.76 (238.10-326.98) | 365.45 (278.67-453.22) | 0.44 (0.38-0.52) | 0.57 (0.44-0.71) | -0.03 (-0.61,0.56) |  | 7792.92 (6653.19-9175.98) | 6291.11 (5077.13-7574.80) | 12.39 (10.58-14.58) | 9.85 (7.95-11.86) | -1.62 (-2.20,-1.03) |
| Jordan | 0.90 (0.53-1.51) | 3.27 (1.99-4.82) | 0.05 (0.03-0.08) | 0.05 (0.03-0.08) | -0.22 (-0.44,-0.00) |  | 0.92 (0.54-1.55) | 3.23 (1.94-4.78) | 0.05 (0.03-0.08) | 0.05 (0.03-0.08) | -0.32 (-0.55,-0.09) |  | 31.72 (18.92-52.59) | 101.35 (62.07-152.65) | 1.70 (1.01-2.82) | 1.64 (1.01-2.48) | -0.64 (-0.88,-0.40) |
| Kazakhstan | 20.84 (14.56-29.75) | 13.78 (9.18-19.78) | 0.25 (0.18-0.36) | 0.15 (0.10-0.21) | -2.60 (-2.95,-2.24) |  | 21.29 (14.98-30.34) | 14.21 (9.56-20.55) | 0.26 (0.18-0.37) | 0.15 (0.10-0.22) | -2.63 (-2.98,-2.27) |  | 711.46 (506.04-1013.48) | 431.99 (294.36-616.36) | 8.68 (6.17-12.36) | 4.56 (3.11-6.50) | -2.95 (-3.33,-2.57) |
| Kenya | 8.76 (6.17-13.04) | 36.13 (26.42-48.62) | 0.08 (0.05-0.11) | 0.14 (0.11-0.19) | 2.18 (2.08,2.29) |  | 9.02 (6.37-13.39) | 36.67 (26.90-49.32) | 0.08 (0.06-0.12) | 0.15 (0.11-0.20) | 2.12 (2.02,2.23) |  | 346.31 (246.73-509.25) | 1348.43 (971.21-1847.00) | 2.99 (2.13-4.40) | 5.39 (3.88-7.38) | 2.03 (1.89,2.17) |
| Kiribati | 0.07 (0.04-0.10) | 0.13 (0.08-0.20) | 0.18 (0.12-0.28) | 0.21 (0.13-0.33) | 0.39 (0.23,0.56) |  | 0.07 (0.04-0.10) | 0.13 (0.08-0.20) | 0.18 (0.12-0.28) | 0.21 (0.13-0.33) | 0.38 (0.22,0.54) |  | 2.64 (1.74-3.95) | 4.82 (3.02-7.47) | 7.10 (4.69-10.61) | 7.96 (4.99-12.33) | 0.33 (0.14,0.51) |
| Kuwait | 0.75 (0.52-1.03) | 0.84 (0.57-1.20) | 0.09 (0.06-0.12) | 0.04 (0.02-0.05) | -2.57 (-2.97,-2.18) |  | 0.73 (0.51-1.01) | 0.79 (0.53-1.12) | 0.09 (0.06-0.12) | 0.03 (0.02-0.05) | -2.86 (-3.82,-1.89) |  | 26.51 (19.25-36.46) | 24.09 (16.27-34.21) | 3.09 (2.24-4.24) | 1.04 (0.70-1.47) | -3.41 (-4.36,-2.44) |
| Kyrgyzstan | 3.33 (2.21-4.95) | 2.62 (1.69-3.92) | 0.15 (0.10-0.22) | 0.08 (0.05-0.11) | -1.58 (-2.46,-0.70) |  | 3.43 (2.29-5.08) | 2.69 (1.75-4.01) | 0.15 (0.10-0.23) | 0.08 (0.05-0.12) | -1.97 (-2.96,-0.97) |  | 116.19 (79.03-169.49) | 82.87 (53.92-126.32) | 5.21 (3.54-7.59) | 2.41 (1.57-3.68) | -2.22 (-3.14,-1.29) |
| Lao People's Democratic Republic | 3.19 (1.95-4.81) | 4.45 (2.66-6.94) | 0.15 (0.09-0.23) | 0.12 (0.07-0.19) | -0.94 (-1.06,-0.81) |  | 3.24 (1.95-4.90) | 4.48 (2.69-7.06) | 0.16 (0.09-0.23) | 0.12 (0.07-0.19) | -0.98 (-1.10,-0.86) |  | 115.49 (72.36-172.74) | 155.00 (92.15-250.98) | 5.54 (3.47-8.29) | 4.20 (2.50-6.80) | -1.06 (-1.15,-0.96) |
| Latvia | 1.43 (0.98-2.01) | 1.51 (1.01-2.14) | 0.11 (0.07-0.15) | 0.16 (0.11-0.23) | 0.54 (0.29,0.79) |  | 1.50 (1.02-2.08) | 1.62 (1.08-2.31) | 0.11 (0.08-0.16) | 0.17 (0.12-0.25) | 0.94 (0.65,1.24) |  | 42.80 (29.90-60.01) | 39.20 (26.38-56.12) | 3.22 (2.25-4.51) | 4.19 (2.82-6.00) | 0.37 (0.08,0.66) |
| Lebanon | 1.61 (1.04-2.51) | 2.92 (1.87-4.29) | 0.11 (0.07-0.17) | 0.11 (0.07-0.15) | -0.11 (-0.28,0.06) |  | 1.66 (1.07-2.56) | 2.94 (1.88-4.33) | 0.11 (0.07-0.17) | 0.11 (0.07-0.16) | -0.22 (-0.40,-0.03) |  | 50.57 (33.14-77.34) | 79.04 (52.63-115.98) | 3.38 (2.21-5.17) | 2.85 (1.90-4.19) | -0.65 (-0.81,-0.49) |
| Lesotho | 1.25 (0.48-2.67) | 4.11 (1.75-8.72) | 0.16 (0.06-0.35) | 0.44 (0.19-0.93) | 3.28 (2.59,3.98) |  | 1.30 (0.50-2.79) | 4.15 (1.76-8.63) | 0.17 (0.07-0.36) | 0.44 (0.19-0.92) | 3.23 (2.57,3.91) |  | 42.75 (15.97-90.22) | 149.44 (62.44-334.06) | 5.58 (2.08-11.77) | 15.95 (6.66-35.64) | 3.63 (2.95,4.32) |
| Liberia | 5.31 (2.81-8.99) | 9.12 (5.69-14.53) | 0.43 (0.23-0.73) | 0.33 (0.21-0.53) | -1.58 (-1.90,-1.27) |  | 5.49 (2.94-9.22) | 9.16 (5.73-14.21) | 0.45 (0.24-0.75) | 0.34 (0.21-0.52) | -1.75 (-2.11,-1.40) |  | 179.78 (97.08-302.10) | 339.33 (206.35-539.01) | 14.61 (7.89-24.56) | 12.43 (7.56-19.75) | -1.24 (-1.60,-0.88) |
| Libya | 3.35 (2.03-5.34) | 11.76 (7.31-18.57) | 0.16 (0.10-0.25) | 0.34 (0.21-0.54) | 2.74 (2.52,2.95) |  | 3.44 (2.08-5.51) | 11.67 (7.22-18.07) | 0.16 (0.10-0.26) | 0.34 (0.21-0.53) | 2.59 (2.35,2.83) |  | 116.92 (72.25-182.31) | 385.65 (243.21-603.22) | 5.55 (3.43-8.65) | 11.23 (7.08-17.56) | 2.54 (2.30,2.79) |
| Lithuania | 1.58 (1.10-2.25) | 2.48 (1.71-3.55) | 0.09 (0.06-0.12) | 0.18 (0.13-0.26) | 2.01 (1.66,2.37) |  | 1.63 (1.13-2.31) | 2.52 (1.74-3.65) | 0.09 (0.06-0.13) | 0.18 (0.13-0.27) | 2.26 (1.92,2.60) |  | 45.41 (32.75-63.99) | 61.90 (43.21-87.01) | 2.47 (1.78-3.48) | 4.54 (3.17-6.38) | 1.74 (1.41,2.07) |
| Luxembourg | 0.36 (0.25-0.50) | 0.90 (0.60-1.25) | 0.19 (0.13-0.26) | 0.28 (0.19-0.39) | 1.50 (1.28,1.72) |  | 0.37 (0.26-0.52) | 0.86 (0.58-1.20) | 0.20 (0.13-0.27) | 0.27 (0.18-0.37) | 1.20 (0.96,1.45) |  | 9.35 (6.76-12.67) | 19.61 (13.45-26.73) | 4.91 (3.54-6.65) | 6.09 (4.17-8.30) | 0.83 (0.60,1.06) |
| Madagascar | 6.47 (4.22-9.83) | 13.10 (7.70-21.20) | 0.11 (0.07-0.17) | 0.09 (0.05-0.15) | -0.69 (-0.89,-0.49) |  | 6.61 (4.33-9.92) | 13.21 (7.72-21.07) | 0.11 (0.07-0.17) | 0.09 (0.05-0.15) | -0.76 (-0.98,-0.55) |  | 266.34 (178.50-392.84) | 532.10 (326.35-830.83) | 4.48 (3.00-6.60) | 3.73 (2.29-5.82) | -0.70 (-0.91,-0.49) |
| Malawi | 4.86 (3.19-7.21) | 12.16 (8.03-18.20) | 0.10 (0.07-0.15) | 0.13 (0.08-0.19) | 0.01 (-0.43,0.44) |  | 4.99 (3.27-7.36) | 12.40 (8.22-18.71) | 0.10 (0.07-0.15) | 0.13 (0.08-0.19) | 0.00 (-0.45,0.46) |  | 193.05 (126.33-287.21) | 480.31 (312.74-715.12) | 3.94 (2.58-5.86) | 4.94 (3.22-7.35) | 0.08 (-0.34,0.51) |
| Malaysia | 5.96 (4.05-8.78) | 22.04 (13.91-33.01) | 0.07 (0.05-0.10) | 0.14 (0.09-0.21) | 2.43 (2.23,2.63) |  | 6.11 (4.11-9.02) | 21.78 (13.80-32.06) | 0.07 (0.05-0.10) | 0.14 (0.09-0.20) | 2.34 (2.12,2.56) |  | 198.18 (135.98-283.19) | 648.95 (422.39-963.87) | 2.24 (1.54-3.21) | 4.08 (2.66-6.06) | 2.04 (1.84,2.25) |
| Maldives | 0.08 (0.05-0.13) | 0.18 (0.11-0.25) | 0.07 (0.04-0.12) | 0.07 (0.04-0.10) | -0.35 (-0.45,-0.24) |  | 0.08 (0.05-0.13) | 0.17 (0.11-0.25) | 0.07 (0.04-0.12) | 0.07 (0.04-0.10) | -0.41 (-0.51,-0.30) |  | 2.73 (1.59-4.23) | 5.08 (3.17-7.45) | 2.46 (1.43-3.81) | 1.96 (1.22-2.88) | -0.94 (-1.08,-0.80) |
| Mali | 23.03 (14.80-34.01) | 54.15 (34.50-80.96) | 0.53 (0.34-0.79) | 0.45 (0.29-0.67) | -0.54 (-0.68,-0.41) |  | 23.48 (15.12-34.74) | 55.02 (35.16-81.66) | 0.54 (0.35-0.80) | 0.46 (0.29-0.68) | -0.53 (-0.65,-0.40) |  | 814.66 (538.10-1202.93) | 1989.45 (1311.67-2927.74) | 18.81 (12.42-27.77) | 16.51 (10.88-24.29) | -0.39 (-0.53,-0.25) |
| Malta | 0.19 (0.13-0.26) | 0.56 (0.36-0.80) | 0.10 (0.07-0.14) | 0.25 (0.16-0.36) | 3.08 (2.93,3.23) |  | 0.20 (0.13-0.28) | 0.54 (0.35-0.77) | 0.11 (0.07-0.15) | 0.24 (0.16-0.35) | 3.04 (2.87,3.21) |  | 4.98 (3.49-6.71) | 12.16 (8.00-16.96) | 2.69 (1.88-3.62) | 5.50 (3.62-7.67) | 2.60 (2.42,2.79) |
| Marshall Islands | 0.01 (0.01-0.03) | 0.03 (0.02-0.06) | 0.06 (0.03-0.11) | 0.12 (0.07-0.21) | 2.26 (2.11,2.41) |  | 0.01 (0.01-0.03) | 0.03 (0.02-0.06) | 0.06 (0.03-0.11) | 0.12 (0.07-0.20) | 2.15 (2.02,2.29) |  | 0.55 (0.29-0.98) | 1.29 (0.73-2.17) | 2.43 (1.28-4.33) | 4.58 (2.60-7.73) | 2.04 (1.83,2.25) |
| Mauritania | 8.21 (2.39-17.86) | 9.53 (5.19-15.71) | 0.80 (0.23-1.74) | 0.43 (0.24-0.71) | -2.31 (-2.50,-2.11) |  | 8.46 (2.47-18.34) | 9.83 (5.32-16.03) | 0.82 (0.24-1.79) | 0.45 (0.24-0.73) | -2.32 (-2.51,-2.13) |  | 278.14 (79.21-612.25) | 312.84 (172.47-517.73) | 27.07 (7.71-59.60) | 14.23 (7.85-23.55) | -2.44 (-2.62,-2.26) |
| Mauritius | 0.46 (0.33-0.66) | 0.18 (0.12-0.26) | 0.08 (0.06-0.12) | 0.03 (0.02-0.04) | 0.90 (-0.87,2.70) |  | 0.47 (0.34-0.67) | 0.18 (0.12-0.26) | 0.09 (0.06-0.12) | 0.03 (0.02-0.04) | 2.43 (0.48,4.42) |  | 14.58 (10.18-20.47) | 4.98 (3.41-7.22) | 2.66 (1.86-3.74) | 0.78 (0.54-1.13) | 2.11 (0.17,4.08) |
| Mexico | 23.41 (20.55-26.57) | 80.94 (65.60-97.79) | 0.05 (0.05-0.06) | 0.13 (0.10-0.15) | 2.91 (2.68,3.14) |  | 24.50 (21.60-27.84) | 84.47 (68.53-101.72) | 0.06 (0.05-0.07) | 0.13 (0.11-0.16) | 2.94 (2.71,3.18) |  | 881.81 (784.41-983.23) | 2518.67 (2051.44-3033.04) | 2.07 (1.84-2.30) | 3.90 (3.17-4.69) | 2.39 (2.17,2.61) |
| Micronesia (Federated States of) | 0.06 (0.03-0.09) | 0.08 (0.05-0.14) | 0.11 (0.07-0.17) | 0.17 (0.09-0.28) | 1.42 (1.27,1.57) |  | 0.06 (0.03-0.09) | 0.08 (0.04-0.14) | 0.11 (0.07-0.17) | 0.16 (0.09-0.28) | 1.36 (1.22,1.50) |  | 2.09 (1.28-3.26) | 3.00 (1.59-5.09) | 4.04 (2.46-6.29) | 5.84 (3.11-9.92) | 1.20 (1.02,1.38) |
| Monaco | 0.05 (0.03-0.08) | 0.16 (0.09-0.24) | 0.35 (0.21-0.54) | 0.82 (0.46-1.25) | 3.08 (2.52,3.65) |  | 0.05 (0.03-0.08) | 0.15 (0.08-0.23) | 0.35 (0.21-0.54) | 0.79 (0.45-1.23) | 2.92 (2.34,3.49) |  | 1.22 (0.76-1.82) | 3.31 (1.96-4.91) | 8.03 (4.97-12.00) | 17.49 (10.35-25.92) | 2.84 (2.28,3.40) |
| Mongolia | 9.61 (5.83-15.56) | 24.18 (15.28-37.34) | 0.89 (0.54-1.44) | 1.45 (0.92-2.24) | 1.90 (1.57,2.23) |  | 9.87 (6.00-16.08) | 24.94 (15.81-38.41) | 0.91 (0.56-1.49) | 1.50 (0.95-2.30) | 1.89 (1.58,2.19) |  | 360.80 (222.02-581.05) | 792.46 (499.94-1216.25) | 33.44 (20.58-53.86) | 47.51 (29.97-72.91) | 1.28 (0.97,1.60) |
| Montenegro | 0.58 (0.37-0.87) | 0.95 (0.59-1.48) | 0.19 (0.12-0.28) | 0.31 (0.19-0.48) | 1.78 (1.66,1.90) |  | 0.61 (0.39-0.90) | 1.00 (0.61-1.55) | 0.19 (0.13-0.29) | 0.32 (0.20-0.50) | 1.86 (1.72,1.99) |  | 17.42 (11.54-24.79) | 24.73 (15.80-38.56) | 5.56 (3.69-7.92) | 8.00 (5.11-12.48) | 1.28 (1.14,1.43) |
| Morocco | 1.75 (1.11-2.79) | 5.11 (3.23-7.60) | 0.01 (0.01-0.02) | 0.03 (0.02-0.04) | 2.24 (2.03,2.44) |  | 1.81 (1.14-2.87) | 5.22 (3.32-7.73) | 0.01 (0.01-0.02) | 0.03 (0.02-0.04) | 2.19 (1.99,2.40) |  | 60.80 (39.88-93.14) | 159.16 (102.35-238.67) | 0.48 (0.31-0.73) | 0.86 (0.55-1.28) | 1.85 (1.64,2.06) |
| Mozambique | 26.50 (15.76-41.06) | 62.29 (32.06-120.56) | 0.40 (0.24-0.61) | 0.40 (0.21-0.78) | 0.17 (0.05,0.29) |  | 27.82 (16.63-43.14) | 64.85 (33.62-124.59) | 0.42 (0.25-0.65) | 0.42 (0.22-0.80) | 0.14 (0.01,0.26) |  | 830.35 (497.20-1298.02) | 2012.22 (1023.10-3864.78) | 12.43 (7.44-19.43) | 12.95 (6.59-24.88) | 0.31 (0.18,0.43) |
| Myanmar | 12.83 (4.45-25.05) | 21.65 (9.06-40.61) | 0.06 (0.02-0.12) | 0.08 (0.03-0.14) | 0.51 (0.42,0.60) |  | 13.01 (4.57-25.10) | 21.82 (9.23-41.15) | 0.06 (0.02-0.12) | 0.08 (0.03-0.15) | 0.47 (0.38,0.57) |  | 480.77 (168.14-929.76) | 716.26 (293.72-1342.17) | 2.38 (0.83-4.60) | 2.54 (1.04-4.76) | 0.08 (-0.01,0.17) |
| Namibia | 0.39 (0.23-0.64) | 1.04 (0.63-1.68) | 0.06 (0.03-0.09) | 0.09 (0.05-0.14) | 0.95 (0.57,1.33) |  | 0.41 (0.24-0.66) | 1.06 (0.65-1.69) | 0.06 (0.03-0.09) | 0.09 (0.05-0.14) | 0.95 (0.57,1.33) |  | 14.16 (8.31-22.46) | 36.39 (22.29-59.66) | 2.02 (1.18-3.20) | 2.99 (1.83-4.91) | 0.90 (0.50,1.31) |
| Nauru | 0.01 (0.00-0.01) | 0.01 (0.00-0.01) | 0.15 (0.09-0.22) | 0.15 (0.09-0.25) | -0.26 (-0.41,-0.10) |  | 0.01 (0.00-0.01) | 0.01 (0.00-0.01) | 0.14 (0.09-0.21) | 0.15 (0.08-0.23) | -0.32 (-0.49,-0.15) |  | 0.29 (0.19-0.44) | 0.33 (0.18-0.53) | 5.76 (3.66-8.64) | 6.06 (3.34-9.62) | -0.25 (-0.43,-0.07) |
| Nepal | 2.69 (1.65-4.08) | 10.58 (5.90-17.30) | 0.03 (0.02-0.04) | 0.07 (0.04-0.11) | 3.36 (2.99,3.74) |  | 2.76 (1.68-4.17) | 10.90 (6.07-17.74) | 0.03 (0.02-0.04) | 0.07 (0.04-0.11) | 3.40 (3.02,3.78) |  | 105.18 (64.18-159.86) | 360.39 (209.46-568.05) | 1.08 (0.66-1.64) | 2.32 (1.35-3.65) | 2.88 (2.54,3.23) |
| Netherlands | 5.89 (4.13-7.81) | 19.82 (13.40-27.25) | 0.08 (0.06-0.10) | 0.23 (0.16-0.32) | 3.68 (3.49,3.87) |  | 6.31 (4.39-8.46) | 21.28 (14.08-29.51) | 0.08 (0.06-0.11) | 0.25 (0.16-0.34) | 3.65 (3.46,3.83) |  | 163.26 (119.38-209.55) | 483.13 (338.31-656.20) | 2.19 (1.60-2.81) | 5.61 (3.93-7.63) | 3.22 (3.06,3.37) |
| New Zealand | 2.26 (1.91-2.64) | 10.16 (8.58-12.02) | 0.13 (0.11-0.15) | 0.39 (0.33-0.46) | 3.22 (3.01,3.43) |  | 1.84 (1.55-2.14) | 7.21 (6.03-8.43) | 0.11 (0.09-0.12) | 0.28 (0.23-0.33) | 2.98 (2.71,3.24) |  | 57.48 (48.96-67.29) | 198.35 (170.11-230.14) | 3.36 (2.87-3.94) | 7.67 (6.58-8.90) | 2.57 (2.33,2.82) |
| Nicaragua | 1.53 (1.05-2.21) | 3.88 (2.55-5.79) | 0.08 (0.05-0.11) | 0.12 (0.08-0.17) | 1.65 (1.24,2.06) |  | 1.59 (1.10-2.31) | 3.99 (2.60-6.01) | 0.08 (0.06-0.12) | 0.12 (0.08-0.18) | 1.63 (1.18,2.08) |  | 59.31 (42.23-84.02) | 126.91 (85.58-187.47) | 3.05 (2.17-4.32) | 3.81 (2.57-5.62) | 1.03 (0.61,1.45) |
| Niger | 13.49 (5.99-28.44) | 22.72 (12.69-40.83) | 0.34 (0.15-0.71) | 0.18 (0.10-0.33) | -2.24 (-2.35,-2.13) |  | 13.64 (6.10-28.71) | 23.23 (12.92-41.70) | 0.34 (0.15-0.71) | 0.19 (0.10-0.33) | -2.14 (-2.25,-2.03) |  | 505.51 (227.77-1071.23) | 819.30 (470.17-1483.02) | 12.59 (5.67-26.67) | 6.54 (3.76-11.85) | -2.35 (-2.48,-2.21) |
| Nigeria | 62.78 (28.84-127.17) | 114.77 (73.10-174.54) | 0.14 (0.06-0.28) | 0.10 (0.06-0.15) | -1.49 (-1.65,-1.33) |  | 65.37 (30.00-131.85) | 118.10 (76.37-178.86) | 0.15 (0.07-0.29) | 0.10 (0.07-0.15) | -1.50 (-1.67,-1.34) |  | 2090.40 (936.21-4279.22) | 3911.52 (2428.40-6147.87) | 4.64 (2.08-9.50) | 3.38 (2.10-5.32) | -1.41 (-1.57,-1.24) |
| Niue | 0.00 (0.00-0.00) | 0.00 (0.00-0.00) | 0.15 (0.09-0.24) | 0.22 (0.12-0.35) | 1.12 (0.79,1.44) |  | 0.00 (0.00-0.00) | 0.00 (0.00-0.00) | 0.15 (0.09-0.25) | 0.22 (0.12-0.34) | 1.01 (0.69,1.33) |  | 0.05 (0.03-0.09) | 0.05 (0.03-0.09) | 4.62 (2.68-8.02) | 6.48 (3.62-10.66) | 0.90 (0.59,1.20) |
| North Macedonia | 3.02 (2.01-4.28) | 3.85 (2.39-6.04) | 0.30 (0.20-0.43) | 0.35 (0.22-0.55) | 0.25 (0.08,0.43) |  | 3.18 (2.08-4.54) | 4.08 (2.51-6.29) | 0.32 (0.21-0.46) | 0.37 (0.23-0.58) | 0.24 (0.06,0.41) |  | 92.64 (64.13-130.30) | 102.44 (63.66-161.76) | 9.30 (6.44-13.08) | 9.41 (5.85-14.86) | -0.30 (-0.49,-0.10) |
| Northern Mariana Islands | 0.02 (0.01-0.03) | 0.06 (0.04-0.09) | 0.10 (0.06-0.15) | 0.24 (0.15-0.38) | 3.33 (2.88,3.78) |  | 0.02 (0.01-0.03) | 0.06 (0.03-0.09) | 0.09 (0.06-0.15) | 0.23 (0.14-0.36) | 3.45 (2.96,3.94) |  | 0.85 (0.53-1.34) | 1.77 (1.08-2.77) | 3.76 (2.34-5.92) | 7.28 (4.46-11.41) | 2.53 (2.14,2.93) |
| Norway | 2.58 (2.18-3.00) | 8.76 (7.46-10.17) | 0.12 (0.10-0.14) | 0.32 (0.28-0.38) | 3.05 (2.69,3.40) |  | 2.56 (2.16-2.99) | 7.77 (6.56-9.05) | 0.12 (0.10-0.14) | 0.29 (0.24-0.33) | 2.69 (2.23,3.14) |  | 64.66 (55.50-74.54) | 190.31 (164.22-221.03) | 3.05 (2.61-3.51) | 7.02 (6.06-8.16) | 2.63 (2.19,3.07) |
| Oman | 0.63 (0.35-1.13) | 2.11 (1.34-3.15) | 0.06 (0.04-0.11) | 0.09 (0.06-0.13) | 1.29 (1.08,1.49) |  | 0.64 (0.35-1.15) | 2.01 (1.28-2.91) | 0.06 (0.04-0.12) | 0.09 (0.05-0.12) | 1.16 (0.91,1.42) |  | 22.04 (12.62-38.47) | 69.22 (44.56-103.45) | 2.22 (1.27-3.88) | 2.94 (1.89-4.40) | 1.19 (0.91,1.47) |
| Pakistan | 28.53 (21.21-37.62) | 82.93 (61.10-111.34) | 0.05 (0.04-0.07) | 0.07 (0.05-0.09) | 0.90 (0.70,1.11) |  | 29.62 (22.23-39.21) | 84.82 (62.75-113.68) | 0.05 (0.04-0.07) | 0.07 (0.05-0.10) | 0.87 (0.66,1.08) |  | 1211.68 (905.70-1602.44) | 3648.44 (2717.46-4869.20) | 2.18 (1.63-2.88) | 3.10 (2.31-4.13) | 1.05 (0.79,1.30) |
| Palau | 0.01 (0.01-0.02) | 0.02 (0.01-0.04) | 0.13 (0.08-0.24) | 0.26 (0.15-0.42) | 2.12 (1.85,2.39) |  | 0.01 (0.01-0.02) | 0.02 (0.01-0.04) | 0.13 (0.08-0.24) | 0.26 (0.15-0.42) | 2.06 (1.83,2.29) |  | 0.35 (0.20-0.63) | 0.80 (0.46-1.29) | 4.63 (2.60-8.30) | 8.80 (5.04-14.24) | 2.05 (1.73,2.37) |
| Palestine | 1.97 (1.15-3.06) | 4.39 (3.00-6.56) | 0.19 (0.11-0.30) | 0.17 (0.12-0.26) | -0.54 (-0.69,-0.40) |  | 2.07 (1.20-3.22) | 4.45 (3.03-6.62) | 0.20 (0.12-0.31) | 0.17 (0.12-0.26) | -0.68 (-0.83,-0.54) |  | 62.37 (38.63-97.83) | 137.54 (95.61-200.85) | 6.09 (3.77-9.56) | 5.36 (3.72-7.82) | -0.60 (-0.76,-0.44) |
| Panama | 1.46 (1.07-1.99) | 3.31 (2.19-4.74) | 0.12 (0.09-0.17) | 0.15 (0.10-0.22) | 1.33 (0.68,1.98) |  | 1.55 (1.12-2.11) | 3.47 (2.34-5.02) | 0.13 (0.09-0.18) | 0.16 (0.11-0.23) | 1.23 (0.59,1.88) |  | 47.86 (36.83-62.27) | 97.35 (65.79-135.01) | 4.01 (3.08-5.21) | 4.54 (3.07-6.29) | 0.93 (0.32,1.54) |
| Papua New Guinea | 1.58 (0.63-4.21) | 3.11 (1.37-7.05) | 0.08 (0.03-0.21) | 0.06 (0.03-0.13) | -0.95 (-1.01,-0.89) |  | 1.59 (0.64-4.20) | 3.08 (1.35-7.01) | 0.08 (0.03-0.20) | 0.06 (0.03-0.13) | -1.05 (-1.13,-0.96) |  | 60.74 (23.98-159.39) | 119.00 (52.35-271.14) | 2.96 (1.17-7.77) | 2.27 (1.00-5.18) | -0.99 (-1.07,-0.90) |
| Paraguay | 1.13 (0.77-1.56) | 4.17 (2.57-6.30) | 0.06 (0.04-0.08) | 0.12 (0.07-0.18) | 3.25 (2.92,3.58) |  | 1.18 (0.80-1.63) | 4.31 (2.65-6.41) | 0.06 (0.04-0.08) | 0.12 (0.07-0.18) | 3.28 (2.92,3.64) |  | 40.01 (28.47-54.98) | 134.47 (83.46-200.40) | 1.98 (1.41-2.72) | 3.75 (2.33-5.59) | 2.87 (2.56,3.18) |
| Peru | 7.56 (5.06-10.84) | 22.39 (13.87-33.65) | 0.07 (0.05-0.10) | 0.12 (0.08-0.19) | 1.71 (1.53,1.88) |  | 7.96 (5.31-11.44) | 23.51 (14.57-35.34) | 0.07 (0.05-0.11) | 0.13 (0.08-0.19) | 1.78 (1.55,2.01) |  | 263.14 (181.95-372.02) | 642.09 (407.23-971.65) | 2.43 (1.68-3.44) | 3.54 (2.25-5.36) | 1.12 (0.89,1.35) |
| Philippines | 33.83 (23.76-46.43) | 74.41 (58.10-95.83) | 0.11 (0.08-0.15) | 0.13 (0.10-0.17) | 0.66 (0.59,0.74) |  | 33.79 (23.80-46.56) | 74.50 (58.35-95.17) | 0.11 (0.08-0.15) | 0.13 (0.10-0.17) | 0.64 (0.56,0.72) |  | 1323.69 (933.44-1767.58) | 2525.16 (1955.39-3231.88) | 4.20 (2.96-5.61) | 4.46 (3.45-5.71) | 0.17 (0.09,0.24) |
| Poland | 4.69 (3.93-5.46) | 16.90 (13.70-19.96) | 0.02 (0.02-0.03) | 0.09 (0.07-0.10) | 5.13 (4.45,5.81) |  | 5.12 (4.29-5.94) | 18.32 (14.94-21.59) | 0.03 (0.02-0.03) | 0.10 (0.08-0.11) | 5.17 (4.46,5.89) |  | 131.62 (112.51-153.72) | 438.92 (358.86-519.26) | 0.69 (0.59-0.81) | 2.30 (1.88-2.72) | 5.00 (4.29,5.72) |
| Portugal | 5.76 (4.07-7.91) | 20.19 (13.86-28.53) | 0.11 (0.08-0.16) | 0.38 (0.26-0.54) | 3.78 (3.53,4.02) |  | 6.07 (4.29-8.31) | 20.65 (14.12-28.92) | 0.12 (0.08-0.16) | 0.39 (0.27-0.55) | 3.77 (3.50,4.05) |  | 168.00 (124.00-222.26) | 483.88 (325.97-688.52) | 3.31 (2.45-4.39) | 9.12 (6.15-12.98) | 3.27 (3.02,3.52) |
| Puerto Rico | 2.57 (1.78-3.56) | 3.98 (2.57-5.67) | 0.14 (0.10-0.20) | 0.24 (0.16-0.34) | 0.96 (0.46,1.45) |  | 2.67 (1.83-3.71) | 4.12 (2.67-5.92) | 0.15 (0.10-0.21) | 0.25 (0.16-0.36) | 1.22 (0.79,1.66) |  | 75.50 (53.39-101.73) | 98.37 (66.31-139.06) | 4.18 (2.96-5.63) | 5.97 (4.03-8.44) | 0.56 (0.11,1.01) |
| Qatar | 0.34 (0.22-0.49) | 2.94 (1.80-4.47) | 0.15 (0.10-0.22) | 0.20 (0.12-0.30) | -0.35 (-0.94,0.25) |  | 0.34 (0.22-0.49) | 2.68 (1.61-4.13) | 0.15 (0.10-0.22) | 0.18 (0.11-0.28) | -0.68 (-1.29,-0.06) |  | 12.22 (7.98-17.98) | 91.00 (57.31-136.67) | 5.49 (3.59-8.09) | 6.11 (3.85-9.18) | -0.73 (-1.28,-0.17) |
| Republic of Korea | 171.17 (109.44-259.02) | 275.79 (175.07-397.78) | 0.77 (0.49-1.17) | 1.07 (0.68-1.54) | 0.97 (0.87,1.06) |  | 166.73 (106.29-251.41) | 200.12 (124.66-289.29) | 0.75 (0.48-1.14) | 0.78 (0.48-1.12) | -0.15 (-0.22,-0.08) |  | 5161.62 (3286.20-7797.84) | 4740.01 (3005.64-6955.76) | 23.33 (14.85-35.25) | 18.38 (11.66-26.98) | -1.08 (-1.19,-0.97) |
| Republic of Moldova | 1.94 (1.40-2.70) | 2.25 (1.54-3.12) | 0.09 (0.06-0.12) | 0.13 (0.09-0.17) | 0.74 (0.04,1.43) |  | 2.02 (1.45-2.80) | 2.36 (1.62-3.26) | 0.09 (0.07-0.13) | 0.13 (0.09-0.18) | 0.68 (-0.06,1.42) |  | 67.73 (50.45-91.65) | 67.05 (46.98-92.55) | 3.05 (2.27-4.12) | 3.73 (2.61-5.15) | 0.18 (-0.48,0.85) |
| Romania | 5.76 (3.79-8.29) | 13.95 (9.10-20.18) | 0.05 (0.03-0.07) | 0.15 (0.10-0.21) | 4.01 (3.77,4.25) |  | 6.06 (4.03-8.70) | 14.85 (9.69-21.05) | 0.05 (0.03-0.07) | 0.16 (0.10-0.22) | 4.17 (3.84,4.49) |  | 183.95 (124.73-263.50) | 361.54 (238.91-529.16) | 1.57 (1.07-2.25) | 3.82 (2.52-5.59) | 3.39 (3.07,3.71) |
| Russian Federation | 48.81 (41.95-57.18) | 89.64 (74.79-105.44) | 0.06 (0.06-0.08) | 0.12 (0.10-0.15) | 2.69 (2.26,3.12) |  | 50.79 (43.45-59.18) | 93.99 (78.44-110.71) | 0.07 (0.06-0.08) | 0.13 (0.11-0.15) | 2.71 (2.23,3.20) |  | 1630.13 (1427.77-1891.63) | 2549.26 (2146.51-3004.85) | 2.16 (1.89-2.51) | 3.52 (2.96-4.15) | 2.05 (1.61,2.50) |
| Rwanda | 6.45 (3.73-9.66) | 9.25 (5.18-14.19) | 0.18 (0.10-0.27) | 0.14 (0.08-0.21) | -1.68 (-2.08,-1.27) |  | 6.58 (3.87-9.79) | 9.47 (5.35-14.61) | 0.18 (0.11-0.27) | 0.14 (0.08-0.22) | -1.48 (-1.95,-1.02) |  | 261.10 (154.92-389.13) | 346.23 (208.75-532.90) | 7.26 (4.31-10.82) | 5.22 (3.15-8.03) | -1.74 (-2.20,-1.27) |
| Saint Kitts and Nevis | 0.04 (0.03-0.06) | 0.05 (0.03-0.07) | 0.19 (0.13-0.27) | 0.18 (0.11-0.25) | -0.74 (-1.40,-0.08) |  | 0.04 (0.03-0.06) | 0.05 (0.03-0.08) | 0.21 (0.13-0.29) | 0.18 (0.12-0.26) | -0.73 (-1.34,-0.12) |  | 1.15 (0.82-1.58) | 1.51 (1.00-2.21) | 5.56 (3.95-7.64) | 5.15 (3.42-7.52) | -0.51 (-1.15,0.14) |
| Saint Lucia | 0.05 (0.04-0.07) | 0.08 (0.05-0.11) | 0.08 (0.06-0.11) | 0.09 (0.06-0.13) | -0.04 (-0.52,0.43) |  | 0.06 (0.04-0.08) | 0.08 (0.05-0.12) | 0.08 (0.06-0.11) | 0.09 (0.06-0.13) | -0.11 (-0.58,0.37) |  | 1.69 (1.22-2.31) | 2.40 (1.62-3.37) | 2.48 (1.79-3.38) | 2.71 (1.82-3.80) | -0.17 (-0.62,0.28) |
| Saint Vincent and the Grenadines | 0.07 (0.05-0.09) | 0.08 (0.06-0.12) | 0.12 (0.09-0.17) | 0.15 (0.10-0.20) | 0.30 (-0.08,0.68) |  | 0.07 (0.05-0.10) | 0.09 (0.06-0.12) | 0.13 (0.09-0.18) | 0.15 (0.10-0.21) | 0.22 (-0.21,0.66) |  | 2.15 (1.60-2.88) | 2.55 (1.76-3.57) | 3.92 (2.92-5.26) | 4.46 (3.09-6.26) | 0.12 (-0.28,0.52) |
| Samoa | 0.08 (0.05-0.12) | 0.13 (0.07-0.20) | 0.10 (0.06-0.15) | 0.12 (0.07-0.18) | 0.63 (0.54,0.72) |  | 0.08 (0.05-0.13) | 0.13 (0.08-0.20) | 0.10 (0.06-0.15) | 0.12 (0.07-0.18) | 0.56 (0.45,0.67) |  | 2.70 (1.69-4.18) | 4.16 (2.43-6.36) | 3.19 (2.00-4.94) | 3.90 (2.28-5.96) | 0.53 (0.44,0.63) |
| San Marino | 0.01 (0.01-0.02) | 0.03 (0.02-0.06) | 0.11 (0.07-0.17) | 0.20 (0.11-0.34) | 2.07 (1.98,2.16) |  | 0.01 (0.01-0.02) | 0.03 (0.02-0.05) | 0.11 (0.07-0.17) | 0.19 (0.11-0.32) | 1.97 (1.86,2.07) |  | 0.33 (0.21-0.46) | 0.71 (0.38-1.16) | 2.75 (1.74-3.89) | 4.31 (2.33-7.10) | 1.74 (1.61,1.86) |
| Sao Tome and Principe | 0.04 (0.02-0.05) | 0.06 (0.03-0.11) | 0.06 (0.04-0.09) | 0.06 (0.03-0.10) | -0.36 (-0.50,-0.22) |  | 0.04 (0.03-0.06) | 0.06 (0.03-0.11) | 0.06 (0.04-0.09) | 0.06 (0.03-0.10) | -0.53 (-0.70,-0.36) |  | 1.22 (0.80-1.72) | 2.20 (1.10-4.05) | 2.00 (1.32-2.83) | 2.03 (1.02-3.73) | -0.18 (-0.39,0.04) |
| Saudi Arabia | 11.09 (6.28-18.58) | 28.45 (17.11-43.16) | 0.14 (0.08-0.23) | 0.15 (0.09-0.23) | -0.62 (-0.94,-0.31) |  | 11.36 (6.38-19.06) | 27.46 (16.83-41.44) | 0.14 (0.08-0.24) | 0.15 (0.09-0.22) | -0.78 (-1.11,-0.46) |  | 381.34 (220.28-632.61) | 907.85 (559.57-1385.88) | 4.81 (2.78-7.98) | 4.82 (2.97-7.35) | -0.79 (-1.11,-0.47) |
| Senegal | 11.91 (7.52-17.83) | 21.90 (13.54-32.69) | 0.31 (0.20-0.47) | 0.28 (0.17-0.41) | -0.56 (-0.68,-0.44) |  | 12.22 (7.77-18.17) | 22.59 (13.92-33.71) | 0.32 (0.20-0.48) | 0.28 (0.18-0.43) | -0.60 (-0.75,-0.46) |  | 422.56 (266.08-617.72) | 736.69 (457.92-1089.63) | 11.07 (6.97-16.19) | 9.29 (5.77-13.74) | -0.80 (-0.95,-0.64) |
| Serbia | 7.81 (4.94-11.61) | 9.58 (5.98-14.08) | 0.16 (0.10-0.24) | 0.21 (0.13-0.32) | 0.49 (0.28,0.70) |  | 8.13 (5.16-12.04) | 10.11 (6.32-14.95) | 0.17 (0.11-0.25) | 0.23 (0.14-0.34) | 0.56 (0.33,0.79) |  | 237.93 (154.19-357.84) | 242.93 (151.93-361.23) | 4.94 (3.20-7.43) | 5.45 (3.41-8.10) | -0.12 (-0.34,0.09) |
| Seychelles | 0.06 (0.04-0.09) | 0.06 (0.04-0.09) | 0.17 (0.12-0.26) | 0.12 (0.07-0.18) | -0.80 (-1.46,-0.14) |  | 0.07 (0.05-0.10) | 0.06 (0.04-0.09) | 0.18 (0.12-0.26) | 0.11 (0.07-0.17) | -0.99 (-1.66,-0.32) |  | 1.95 (1.37-2.87) | 1.81 (1.16-2.86) | 5.35 (3.75-7.87) | 3.43 (2.20-5.42) | -0.88 (-1.53,-0.23) |
| Sierra Leone | 7.71 (2.76-15.63) | 9.65 (5.68-15.50) | 0.37 (0.13-0.75) | 0.22 (0.13-0.35) | -2.09 (-2.28,-1.89) |  | 7.98 (2.88-16.25) | 9.84 (5.87-15.76) | 0.38 (0.14-0.78) | 0.22 (0.13-0.36) | -2.15 (-2.36,-1.94) |  | 261.55 (94.36-531.22) | 348.96 (201.72-569.46) | 12.60 (4.55-25.59) | 7.87 (4.55-12.84) | -1.81 (-2.00,-1.62) |
| Singapore | 2.50 (1.74-3.48) | 7.94 (5.06-11.38) | 0.16 (0.11-0.23) | 0.28 (0.18-0.40) | 1.51 (1.25,1.77) |  | 2.37 (1.62-3.34) | 5.96 (3.85-8.45) | 0.16 (0.11-0.22) | 0.21 (0.13-0.30) | 0.87 (0.57,1.17) |  | 68.55 (48.60-97.62) | 138.46 (90.99-199.70) | 4.50 (3.19-6.41) | 4.84 (3.18-6.97) | 0.11 (-0.21,0.43) |
| Slovakia | 4.81 (3.15-6.95) | 4.83 (2.93-7.55) | 0.18 (0.12-0.26) | 0.18 (0.11-0.28) | -0.36 (-0.49,-0.23) |  | 5.06 (3.29-7.33) | 5.06 (3.09-7.94) | 0.19 (0.12-0.28) | 0.19 (0.11-0.29) | -0.44 (-0.60,-0.29) |  | 139.49 (93.93-201.55) | 125.34 (76.79-195.61) | 5.28 (3.56-7.63) | 4.62 (2.83-7.21) | -0.76 (-0.91,-0.60) |
| Slovenia | 1.61 (1.09-2.23) | 2.71 (1.75-3.92) | 0.16 (0.11-0.23) | 0.26 (0.17-0.38) | 1.80 (1.58,2.02) |  | 1.68 (1.12-2.34) | 2.83 (1.81-4.13) | 0.17 (0.11-0.24) | 0.27 (0.18-0.40) | 1.61 (1.34,1.88) |  | 46.21 (31.59-64.36) | 59.83 (39.80-87.57) | 4.68 (3.20-6.52) | 5.78 (3.85-8.46) | 0.66 (0.40,0.91) |
| Solomon Islands | 0.18 (0.06-0.43) | 0.40 (0.23-0.69) | 0.11 (0.04-0.25) | 0.12 (0.07-0.20) | 0.19 (0.03,0.36) |  | 0.18 (0.06-0.44) | 0.39 (0.23-0.68) | 0.11 (0.04-0.26) | 0.11 (0.07-0.20) | 0.11 (-0.04,0.26) |  | 6.88 (2.30-16.24) | 15.17 (9.07-25.62) | 4.06 (1.36-9.58) | 4.44 (2.65-7.49) | 0.25 (0.10,0.40) |
| Somalia | 7.86 (3.71-14.54) | 18.55 (9.22-37.02) | 0.20 (0.09-0.37) | 0.17 (0.09-0.34) | -0.92 (-1.12,-0.72) |  | 7.90 (3.77-14.60) | 18.82 (9.44-37.82) | 0.20 (0.09-0.37) | 0.17 (0.09-0.35) | -0.84 (-1.05,-0.63) |  | 305.44 (146.85-553.19) | 725.00 (356.69-1399.90) | 7.69 (3.70-13.94) | 6.71 (3.30-12.96) | -0.89 (-1.12,-0.67) |
| South Africa | 25.57 (16.81-39.00) | 65.46 (52.93-81.21) | 0.14 (0.09-0.21) | 0.23 (0.19-0.29) | 1.28 (0.60,1.96) |  | 25.51 (16.78-39.08) | 66.32 (53.55-82.28) | 0.14 (0.09-0.21) | 0.23 (0.19-0.29) | 1.23 (0.55,1.90) |  | 1028.78 (683.82-1521.41) | 2247.28 (1793.66-2771.28) | 5.56 (3.70-8.22) | 7.91 (6.31-9.75) | 0.62 (-0.15,1.40) |
| South Sudan | 4.17 (2.43-6.63) | 8.47 (4.94-14.13) | 0.14 (0.08-0.23) | 0.18 (0.10-0.29) | 0.31 (-0.13,0.74) |  | 4.29 (2.53-6.79) | 8.63 (5.08-14.41) | 0.15 (0.09-0.23) | 0.18 (0.11-0.30) | 0.37 (-0.06,0.81) |  | 155.52 (90.36-244.17) | 328.80 (192.71-536.24) | 5.29 (3.07-8.31) | 6.80 (3.98-11.09) | 0.60 (0.19,1.00) |
| Spain | 39.76 (28.42-55.32) | 96.71 (65.97-138.63) | 0.21 (0.15-0.29) | 0.42 (0.29-0.61) | 2.10 (1.78,2.42) |  | 40.26 (28.25-56.30) | 87.31 (58.23-121.76) | 0.21 (0.15-0.29) | 0.38 (0.26-0.53) | 1.71 (1.45,1.97) |  | 1095.80 (808.54-1482.93) | 2032.32 (1395.38-2847.98) | 5.65 (4.17-7.65) | 8.92 (6.13-12.51) | 1.31 (1.11,1.50) |
| Sri Lanka | 3.13 (2.05-4.61) | 5.09 (2.69-8.22) | 0.04 (0.02-0.05) | 0.05 (0.02-0.07) | 0.15 (-0.23,0.53) |  | 3.22 (2.06-4.73) | 4.98 (2.61-7.96) | 0.04 (0.02-0.06) | 0.04 (0.02-0.07) | 0.08 (-0.31,0.47) |  | 104.02 (70.00-148.96) | 139.72 (75.61-222.75) | 1.21 (0.82-1.74) | 1.25 (0.68-2.00) | -0.41 (-0.81,-0.02) |
| Sudan | 12.59 (6.47-23.98) | 29.24 (17.09-47.07) | 0.13 (0.06-0.24) | 0.13 (0.08-0.22) | 0.26 (-0.02,0.55) |  | 13.02 (6.73-25.35) | 29.85 (17.60-48.33) | 0.13 (0.07-0.25) | 0.14 (0.08-0.22) | 0.21 (-0.08,0.49) |  | 470.05 (243.41-873.11) | 1069.60 (635.43-1712.30) | 4.70 (2.43-8.72) | 4.93 (2.93-7.89) | 0.22 (-0.05,0.49) |
| Suriname | 0.16 (0.11-0.23) | 0.42 (0.26-0.66) | 0.08 (0.05-0.12) | 0.15 (0.09-0.23) | 1.78 (1.53,2.03) |  | 0.17 (0.11-0.24) | 0.44 (0.27-0.67) | 0.09 (0.06-0.12) | 0.15 (0.09-0.23) | 1.59 (1.27,1.90) |  | 5.41 (3.64-7.77) | 13.25 (8.49-20.13) | 2.80 (1.88-4.02) | 4.57 (2.93-6.95) | 1.30 (0.99,1.61) |
| Sweden | 9.72 (8.19-11.49) | 14.27 (11.44-17.25) | 0.23 (0.19-0.27) | 0.28 (0.22-0.33) | 1.14 (0.28,2.00) |  | 10.41 (8.73-12.42) | 14.78 (11.79-17.84) | 0.24 (0.20-0.29) | 0.28 (0.23-0.34) | 1.29 (0.45,2.14) |  | 261.01 (222.58-307.13) | 339.78 (276.57-406.22) | 6.08 (5.18-7.15) | 6.55 (5.33-7.83) | 1.13 (0.28,2.00) |
| Switzerland | 7.80 (5.53-10.86) | 14.30 (9.41-20.06) | 0.23 (0.16-0.32) | 0.32 (0.21-0.45) | 1.19 (0.99,1.40) |  | 7.42 (5.26-10.42) | 12.93 (8.25-18.09) | 0.22 (0.15-0.30) | 0.29 (0.18-0.41) | 1.12 (1.00,1.24) |  | 192.79 (139.86-260.24) | 280.15 (192.57-389.15) | 5.62 (4.07-7.58) | 6.28 (4.32-8.72) | 0.52 (0.37,0.67) |
| Syrian Arab Republic | 11.27 (7.09-18.31) | 18.97 (11.51-29.85) | 0.18 (0.11-0.29) | 0.27 (0.16-0.43) | 0.97 (0.19,1.75) |  | 11.61 (7.32-19.06) | 19.10 (11.50-29.94) | 0.18 (0.12-0.30) | 0.27 (0.16-0.43) | 0.87 (0.05,1.69) |  | 413.49 (273.83-647.11) | 575.04 (350.96-911.39) | 6.50 (4.31-10.18) | 8.20 (5.00-12.99) | 0.29 (-0.49,1.07) |
| Taiwan (Province of China) | 27.16 (19.24-39.22) | 92.32 (63.25-127.69) | 0.27 (0.19-0.38) | 0.78 (0.54-1.08) | 4.63 (3.74,5.52) |  | 25.72 (18.15-37.04) | 78.11 (52.47-109.48) | 0.25 (0.18-0.36) | 0.66 (0.44-0.93) | 4.34 (3.41,5.27) |  | 937.53 (691.80-1347.48) | 2087.48 (1456.24-2919.29) | 9.20 (6.79-13.22) | 17.66 (12.32-24.70) | 3.25 (2.35,4.16) |
| Tajikistan | 2.92 (1.84-4.56) | 4.82 (2.72-7.74) | 0.11 (0.07-0.17) | 0.09 (0.05-0.15) | -0.77 (-0.96,-0.59) |  | 3.02 (1.90-4.77) | 4.90 (2.79-7.86) | 0.11 (0.07-0.18) | 0.10 (0.05-0.15) | -0.79 (-0.97,-0.60) |  | 112.86 (72.29-176.45) | 177.06 (101.89-283.35) | 4.20 (2.69-6.57) | 3.49 (2.01-5.58) | -1.06 (-1.28,-0.84) |
| Thailand | 70.09 (45.63-111.00) | 130.70 (76.61-198.66) | 0.25 (0.16-0.39) | 0.39 (0.23-0.60) | 1.06 (0.76,1.37) |  | 70.83 (46.69-111.94) | 126.98 (74.58-192.37) | 0.25 (0.16-0.39) | 0.38 (0.22-0.58) | 0.96 (0.68,1.24) |  | 2395.68 (1602.26-3596.07) | 3607.30 (2127.72-5570.65) | 8.44 (5.65-12.67) | 10.82 (6.38-16.71) | 0.34 (0.01,0.67) |
| Timor-Leste | 0.18 (0.10-0.29) | 0.42 (0.23-0.71) | 0.05 (0.03-0.08) | 0.06 (0.03-0.10) | 0.98 (0.81,1.14) |  | 0.19 (0.11-0.30) | 0.43 (0.24-0.73) | 0.05 (0.03-0.08) | 0.06 (0.03-0.10) | 1.08 (0.90,1.25) |  | 6.96 (3.93-11.24) | 13.78 (7.82-22.83) | 1.78 (1.01-2.88) | 1.97 (1.12-3.27) | 0.42 (0.17,0.67) |
| Togo | 3.31 (2.23-4.92) | 8.90 (5.16-14.70) | 0.18 (0.12-0.27) | 0.21 (0.12-0.35) | 0.17 (0.06,0.28) |  | 3.36 (2.24-5.01) | 9.01 (5.19-14.61) | 0.18 (0.12-0.27) | 0.22 (0.12-0.35) | 0.20 (0.08,0.32) |  | 125.36 (84.85-189.42) | 315.77 (182.59-524.79) | 6.88 (4.65-10.39) | 7.54 (4.36-12.54) | -0.02 (-0.14,0.10) |
| Tokelau | 0.00 (0.00-0.00) | 0.00 (0.00-0.00) | 0.14 (0.07-0.27) | 0.18 (0.10-0.32) | 0.83 (0.67,1.00) |  | 0.00 (0.00-0.00) | 0.00 (0.00-0.00) | 0.14 (0.07-0.28) | 0.18 (0.10-0.32) | 0.69 (0.51,0.87) |  | 0.04 (0.02-0.07) | 0.04 (0.02-0.07) | 4.48 (2.37-8.74) | 5.66 (3.26-9.85) | 0.74 (0.58,0.90) |
| Tonga | 0.23 (0.14-0.37) | 0.30 (0.18-0.47) | 0.46 (0.28-0.76) | 0.57 (0.33-0.89) | 0.41 (0.08,0.74) |  | 0.23 (0.14-0.37) | 0.30 (0.18-0.47) | 0.46 (0.28-0.76) | 0.57 (0.34-0.88) | 0.41 (0.08,0.75) |  | 7.66 (4.66-12.45) | 9.66 (5.77-15.34) | 15.50 (9.44-25.19) | 18.18 (10.86-28.85) | 0.31 (-0.02,0.63) |
| Trinidad and Tobago | 0.57 (0.41-0.76) | 1.05 (0.68-1.54) | 0.09 (0.07-0.13) | 0.15 (0.10-0.22) | 1.26 (0.95,1.56) |  | 0.60 (0.43-0.81) | 1.09 (0.70-1.57) | 0.10 (0.07-0.13) | 0.16 (0.10-0.23) | 1.15 (0.79,1.51) |  | 18.43 (13.71-24.11) | 31.34 (20.30-46.41) | 3.06 (2.28-4.00) | 4.50 (2.91-6.66) | 0.94 (0.61,1.27) |
| Tunisia | 1.83 (1.17-2.84) | 4.95 (2.72-8.03) | 0.04 (0.03-0.07) | 0.08 (0.05-0.14) | 2.10 (1.99,2.20) |  | 1.89 (1.22-2.91) | 4.97 (2.70-7.97) | 0.05 (0.03-0.07) | 0.08 (0.05-0.13) | 2.01 (1.90,2.11) |  | 62.92 (41.72-95.70) | 144.06 (82.75-232.34) | 1.51 (1.00-2.29) | 2.43 (1.40-3.92) | 1.58 (1.48,1.67) |
| Turkey | 28.38 (18.65-41.60) | 65.83 (42.78-96.68) | 0.10 (0.06-0.14) | 0.16 (0.10-0.23) | 1.79 (1.40,2.18) |  | 29.57 (19.39-43.80) | 67.81 (44.34-100.44) | 0.10 (0.07-0.15) | 0.16 (0.11-0.24) | 1.75 (1.34,2.16) |  | 915.12 (608.15-1290.97) | 1767.51 (1162.73-2628.92) | 3.18 (2.12-4.49) | 4.23 (2.78-6.29) | 1.09 (0.69,1.49) |
| Turkmenistan | 1.84 (1.31-2.64) | 3.79 (2.44-5.82) | 0.10 (0.07-0.14) | 0.15 (0.09-0.23) | 1.60 (1.46,1.73) |  | 1.88 (1.36-2.69) | 3.84 (2.47-5.90) | 0.10 (0.07-0.15) | 0.15 (0.10-0.23) | 1.46 (1.21,1.72) |  | 70.47 (51.51-96.73) | 130.95 (86.30-197.64) | 3.81 (2.79-5.23) | 5.08 (3.35-7.66) | 1.18 (0.98,1.38) |
| Tuvalu | 0.01 (0.00-0.01) | 0.01 (0.01-0.02) | 0.14 (0.08-0.25) | 0.16 (0.10-0.27) | 0.25 (0.10,0.39) |  | 0.01 (0.00-0.01) | 0.01 (0.01-0.02) | 0.14 (0.08-0.26) | 0.16 (0.10-0.27) | 0.23 (0.08,0.39) |  | 0.24 (0.14-0.42) | 0.33 (0.20-0.54) | 4.96 (2.96-8.83) | 5.29 (3.24-8.78) | 0.15 (0.01,0.30) |
| Uganda | 14.54 (9.22-21.60) | 35.83 (22.13-56.11) | 0.17 (0.11-0.25) | 0.17 (0.10-0.26) | -0.73 (-1.00,-0.45) |  | 15.00 (9.41-22.26) | 36.63 (22.61-56.98) | 0.17 (0.11-0.26) | 0.17 (0.10-0.26) | -0.72 (-1.01,-0.44) |  | 544.65 (348.25-819.34) | 1487.40 (913.65-2303.45) | 6.30 (4.03-9.48) | 6.87 (4.22-10.64) | -0.38 (-0.72,-0.05) |
| Ukraine | 21.05 (16.53-25.84) | 15.34 (10.86-20.62) | 0.08 (0.06-0.10) | 0.07 (0.05-0.10) | -1.39 (-2.01,-0.76) |  | 21.48 (16.86-26.29) | 15.51 (10.99-20.85) | 0.08 (0.06-0.10) | 0.07 (0.05-0.10) | -1.15 (-1.81,-0.48) |  | 661.08 (527.75-812.31) | 445.83 (309.96-600.04) | 2.51 (2.00-3.08) | 2.07 (1.44-2.79) | -1.41 (-2.09,-0.73) |
| United Arab Emirates | 1.27 (0.78-1.97) | 11.41 (7.18-17.99) | 0.14 (0.08-0.21) | 0.24 (0.15-0.37) | 0.79 (0.27,1.31) |  | 1.27 (0.77-1.94) | 10.89 (6.89-17.30) | 0.14 (0.08-0.21) | 0.23 (0.14-0.36) | 0.60 (0.08,1.13) |  | 47.17 (29.64-71.83) | 394.36 (252.78-620.73) | 5.04 (3.17-7.68) | 8.19 (5.25-12.89) | 0.70 (0.28,1.13) |
| United Kingdom | 39.51 (32.96-46.47) | 165.27 (138.97-194.60) | 0.14 (0.12-0.16) | 0.49 (0.41-0.57) | 4.97 (4.74,5.20) |  | 37.48 (31.31-44.22) | 142.77 (117.22-168.95) | 0.13 (0.11-0.15) | 0.42 (0.35-0.50) | 4.50 (4.25,4.76) |  | 942.05 (800.29-1109.50) | 3234.80 (2735.35-3790.10) | 3.29 (2.79-3.87) | 9.54 (8.06-11.17) | 4.20 (3.96,4.44) |
| United Republic of Tanzania | 20.62 (13.67-30.58) | 40.86 (24.70-64.73) | 0.16 (0.11-0.24) | 0.14 (0.08-0.22) | -0.78 (-0.90,-0.66) |  | 21.19 (13.87-31.14) | 41.90 (25.39-66.31) | 0.16 (0.11-0.24) | 0.14 (0.09-0.23) | -0.79 (-0.91,-0.66) |  | 785.56 (515.65-1157.01) | 1562.86 (945.53-2498.59) | 6.08 (3.99-8.96) | 5.35 (3.24-8.55) | -0.73 (-0.86,-0.61) |
| United States of America | 354.87 (308.81-403.55) | 1318.91 (1120.53-1546.06) | 0.28 (0.24-0.32) | 0.79 (0.67-0.93) | 3.31 (3.20,3.43) |  | 301.78 (261.33-344.38) | 1019.77 (867.47-1191.59) | 0.24 (0.21-0.27) | 0.61 (0.52-0.72) | 3.03 (2.97,3.08) |  | 8279.36 (7301.27-9362.96) | 25790.96 (22195.42-30163.81) | 6.52 (5.75-7.37) | 15.51 (13.34-18.14) | 2.88 (2.77,2.98) |
| United States Virgin Islands | 0.04 (0.03-0.07) | 0.08 (0.05-0.13) | 0.08 (0.05-0.13) | 0.19 (0.11-0.31) | 2.76 (2.57,2.95) |  | 0.04 (0.03-0.07) | 0.09 (0.05-0.14) | 0.08 (0.05-0.13) | 0.21 (0.12-0.33) | 2.86 (2.67,3.04) |  | 1.37 (0.86-2.12) | 2.17 (1.25-3.42) | 2.58 (1.63-3.99) | 5.05 (2.91-7.95) | 2.09 (1.92,2.26) |
| Uruguay | 0.90 (0.60-1.30) | 2.96 (2.01-4.09) | 0.06 (0.04-0.08) | 0.17 (0.12-0.24) | 4.33 (4.05,4.60) |  | 0.94 (0.63-1.36) | 3.07 (2.07-4.24) | 0.06 (0.04-0.09) | 0.18 (0.12-0.25) | 4.27 (3.96,4.59) |  | 25.23 (17.04-36.25) | 76.56 (53.20-106.39) | 1.61 (1.09-2.31) | 4.50 (3.12-6.25) | 4.06 (3.74,4.38) |
| Uzbekistan | 7.38 (4.68-11.16) | 20.63 (13.03-31.35) | 0.07 (0.04-0.11) | 0.12 (0.08-0.18) | 1.66 (1.53,1.79) |  | 7.54 (4.78-11.22) | 20.89 (13.18-31.69) | 0.07 (0.05-0.11) | 0.12 (0.08-0.19) | 1.68 (1.56,1.80) |  | 276.73 (177.08-405.74) | 723.12 (464.87-1089.37) | 2.64 (1.69-3.87) | 4.22 (2.72-6.36) | 1.53 (1.40,1.66) |
| Vanuatu | 0.06 (0.03-0.13) | 0.16 (0.09-0.28) | 0.08 (0.05-0.17) | 0.10 (0.06-0.18) | 0.77 (0.68,0.87) |  | 0.06 (0.04-0.13) | 0.16 (0.09-0.28) | 0.08 (0.05-0.17) | 0.10 (0.06-0.18) | 0.75 (0.64,0.87) |  | 2.37 (1.28-4.81) | 5.86 (3.34-10.13) | 3.12 (1.68-6.32) | 3.74 (2.13-6.47) | 0.57 (0.45,0.70) |
| Venezuela (Bolivarian Republic of) | 13.78 (10.01-18.66) | 18.59 (11.99-27.04) | 0.15 (0.11-0.20) | 0.14 (0.09-0.20) | 0.13 (-0.98,1.25) |  | 14.34 (10.30-19.38) | 19.22 (12.50-27.95) | 0.15 (0.11-0.21) | 0.14 (0.09-0.21) | -0.00 (-1.27,1.28) |  | 457.84 (342.97-611.75) | 605.27 (404.41-846.27) | 4.87 (3.65-6.51) | 4.55 (3.04-6.36) | -0.04 (-1.17,1.10) |
| Viet Nam | 78.96 (47.84-116.79) | 167.93 (96.98-259.06) | 0.23 (0.14-0.34) | 0.33 (0.19-0.52) | 1.44 (1.28,1.60) |  | 80.70 (48.95-119.02) | 162.24 (94.66-248.28) | 0.24 (0.14-0.35) | 0.32 (0.19-0.50) | 1.26 (1.13,1.40) |  | 2637.44 (1634.18-3871.74) | 5084.65 (2945.50-8082.68) | 7.73 (4.79-11.35) | 10.14 (5.88-16.12) | 1.17 (1.01,1.33) |
| Yemen | 4.66 (1.74-10.28) | 10.11 (5.03-20.12) | 0.07 (0.03-0.15) | 0.06 (0.03-0.12) | -0.55 (-0.72,-0.38) |  | 4.79 (1.80-10.61) | 10.34 (5.08-20.74) | 0.07 (0.03-0.16) | 0.06 (0.03-0.12) | -0.59 (-0.75,-0.44) |  | 173.45 (65.99-383.37) | 368.17 (180.32-746.42) | 2.54 (0.97-5.62) | 2.19 (1.07-4.44) | -0.68 (-0.82,-0.55) |
| Zambia | 9.27 (5.53-16.06) | 10.96 (4.17-27.52) | 0.23 (0.14-0.40) | 0.11 (0.04-0.28) | -3.72 (-4.24,-3.21) |  | 9.43 (5.60-16.51) | 11.25 (4.33-28.39) | 0.24 (0.14-0.42) | 0.12 (0.04-0.29) | -3.70 (-4.23,-3.18) |  | 377.05 (225.45-666.22) | 432.64 (159.57-1107.12) | 9.50 (5.68-16.79) | 4.43 (1.64-11.35) | -3.77 (-4.29,-3.24) |
| Zimbabwe | 11.62 (7.05-18.01) | 30.74 (17.41-49.00) | 0.22 (0.14-0.35) | 0.39 (0.22-0.63) | 1.52 (0.87,2.17) |  | 11.95 (7.21-18.55) | 31.28 (17.93-49.56) | 0.23 (0.14-0.36) | 0.40 (0.23-0.64) | 1.53 (0.86,2.20) |  | 415.63 (255.35-659.67) | 1167.81 (670.67-1815.94) | 8.04 (4.94-12.76) | 14.98 (8.60-23.29) | 1.88 (1.17,2.59) |

**Abbreviations:** UI, uncertainty interval; ASR, age-standardised rate per 100,000; EAPC, estimated annual percentage change; CI, confidence interval; DALYs, disability-adjusted life-year; SDI, socio-demographic index.

**Figure 1. Etiology-specific percentages and ranking heatmap of liver cancer incidence, mortality, and disability-adjusted life years (DALYs) in 2021**

**A**

Incidence number


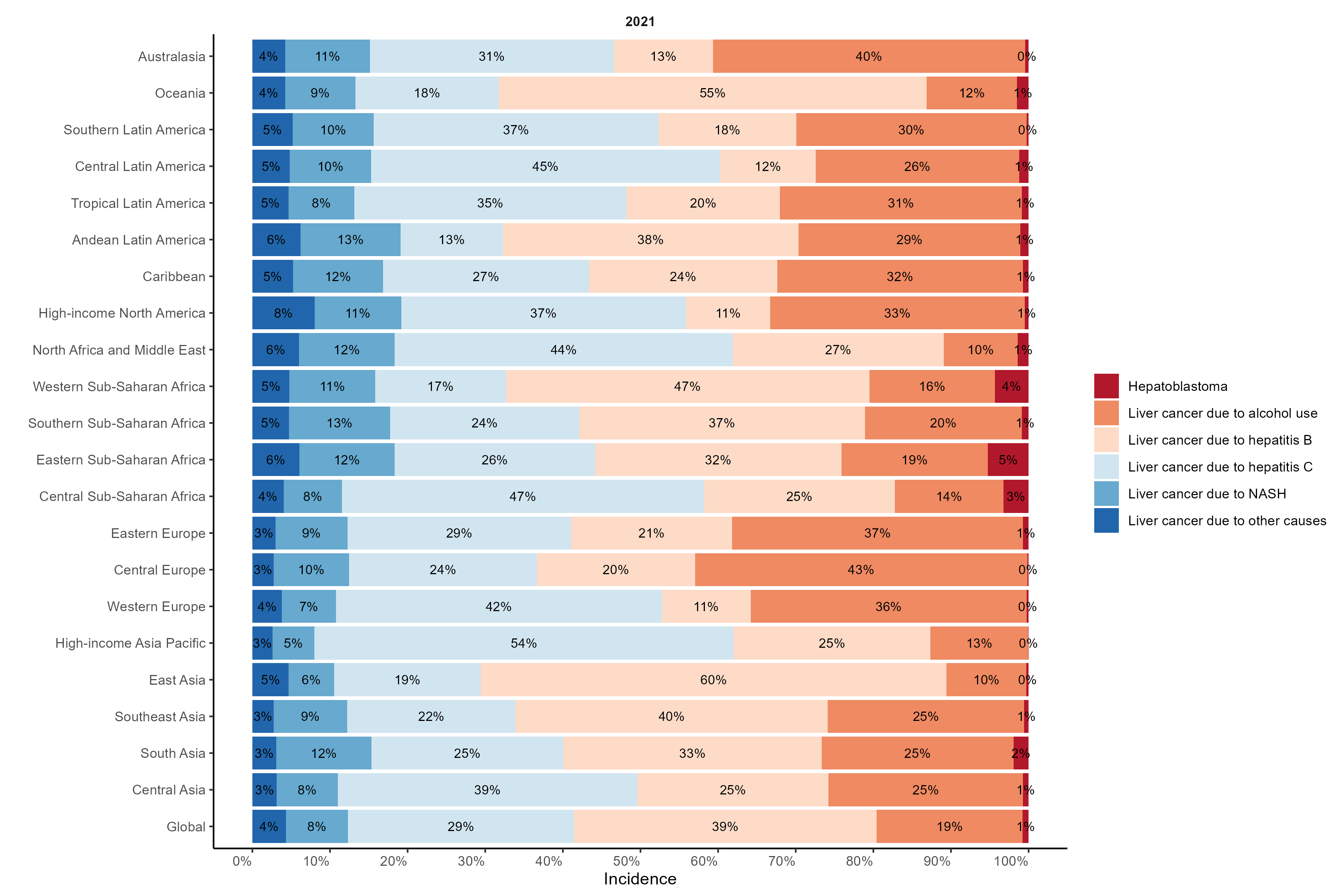


**B**

Incidence rate


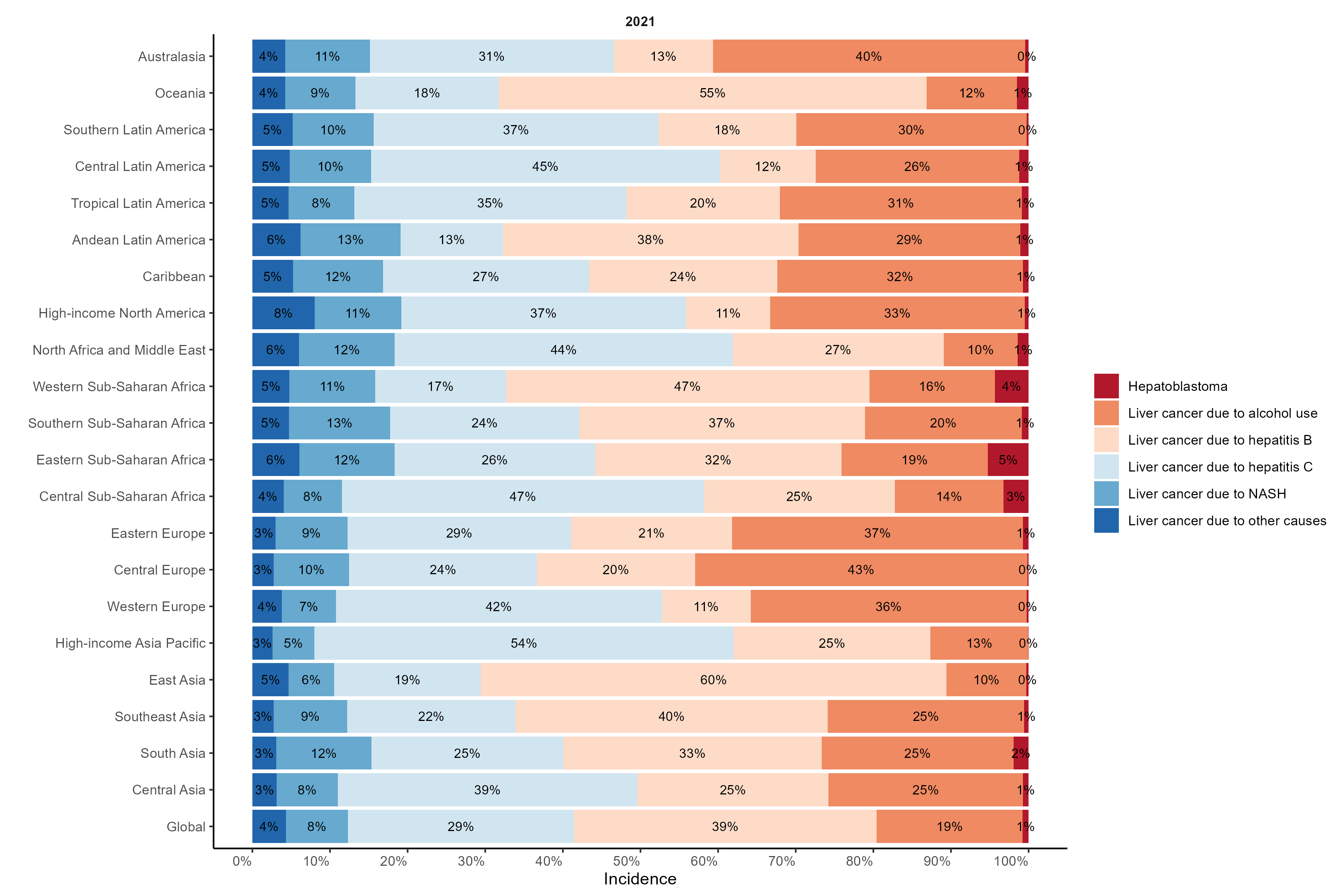


**C** Incidence rate


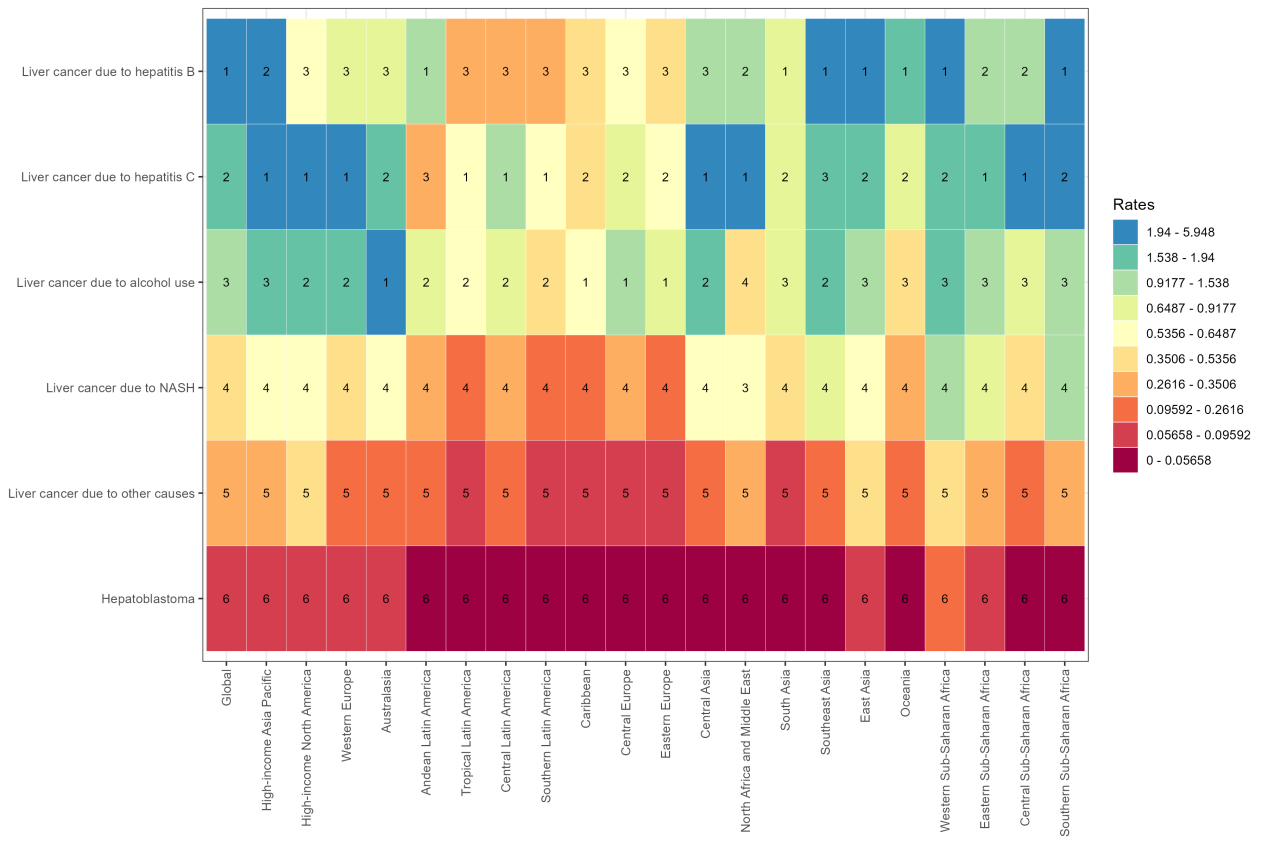


**D**

Deaths number


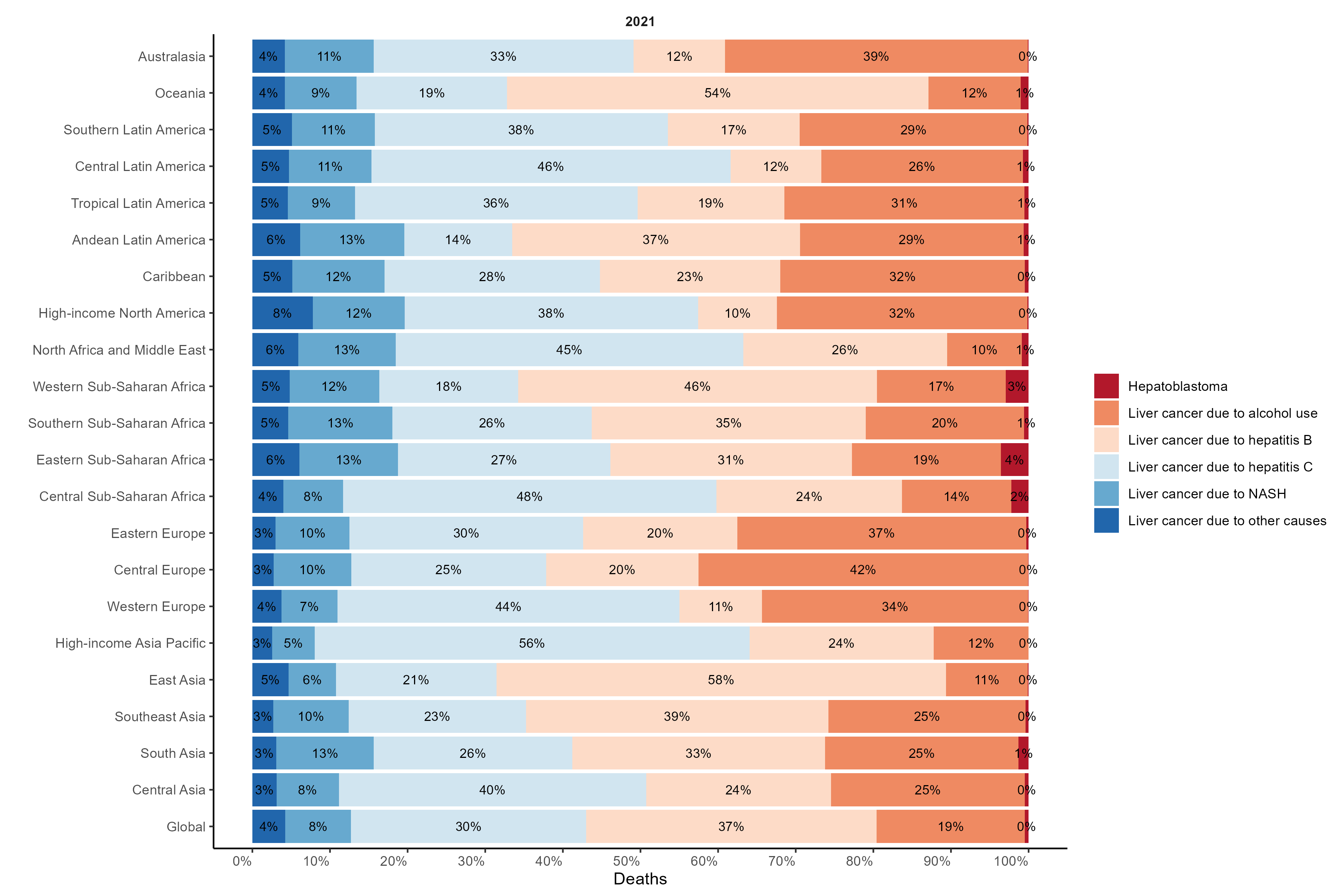


**E**

Deaths rate


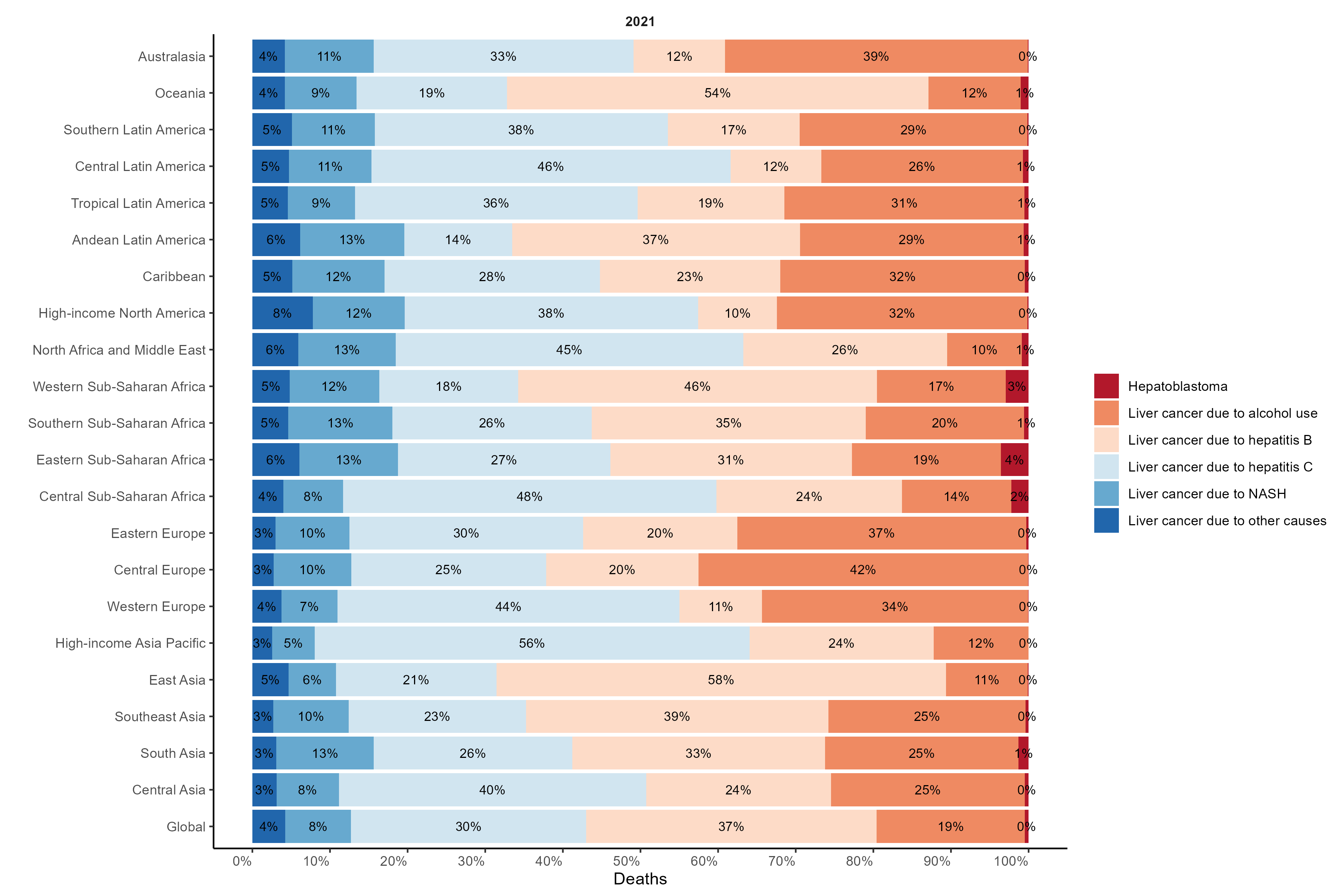


**F** Deaths rate


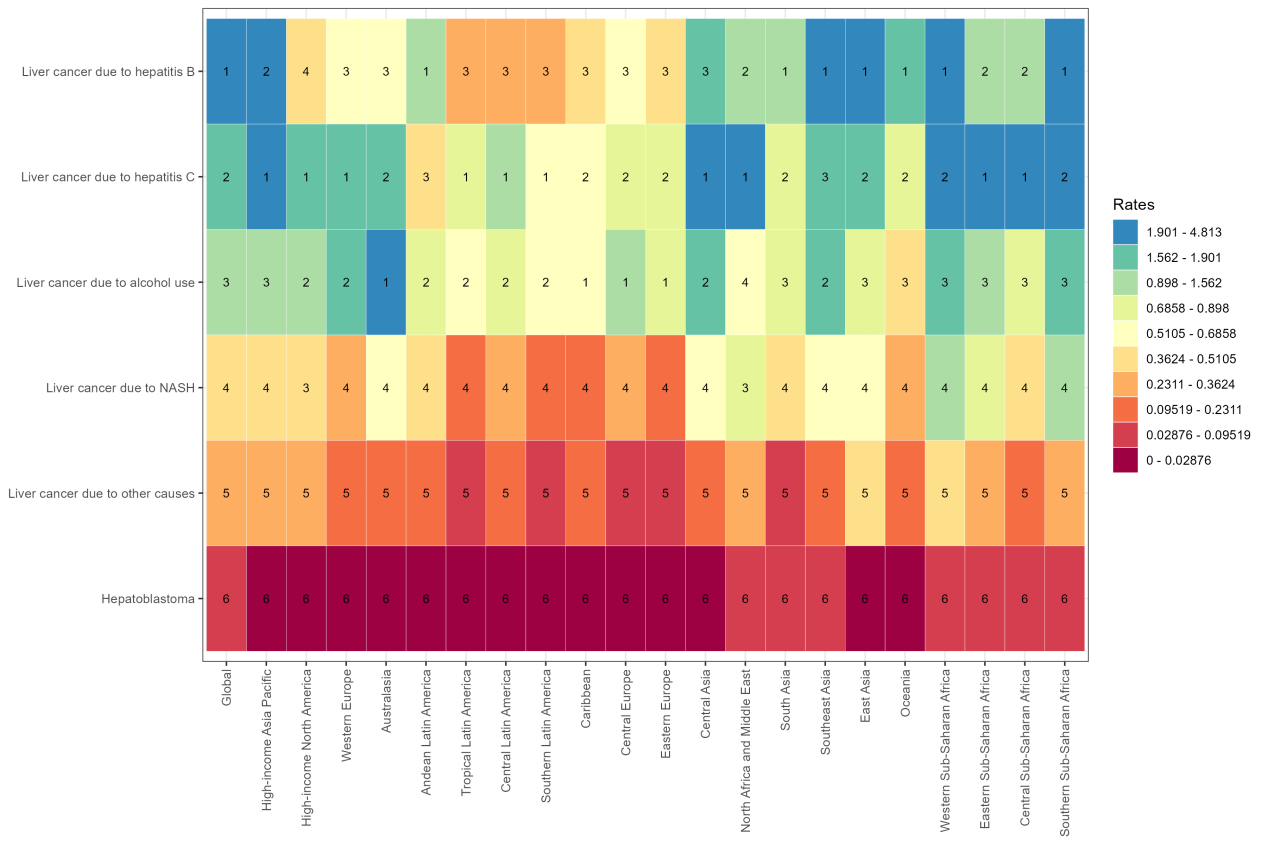


**G**

DALYs number


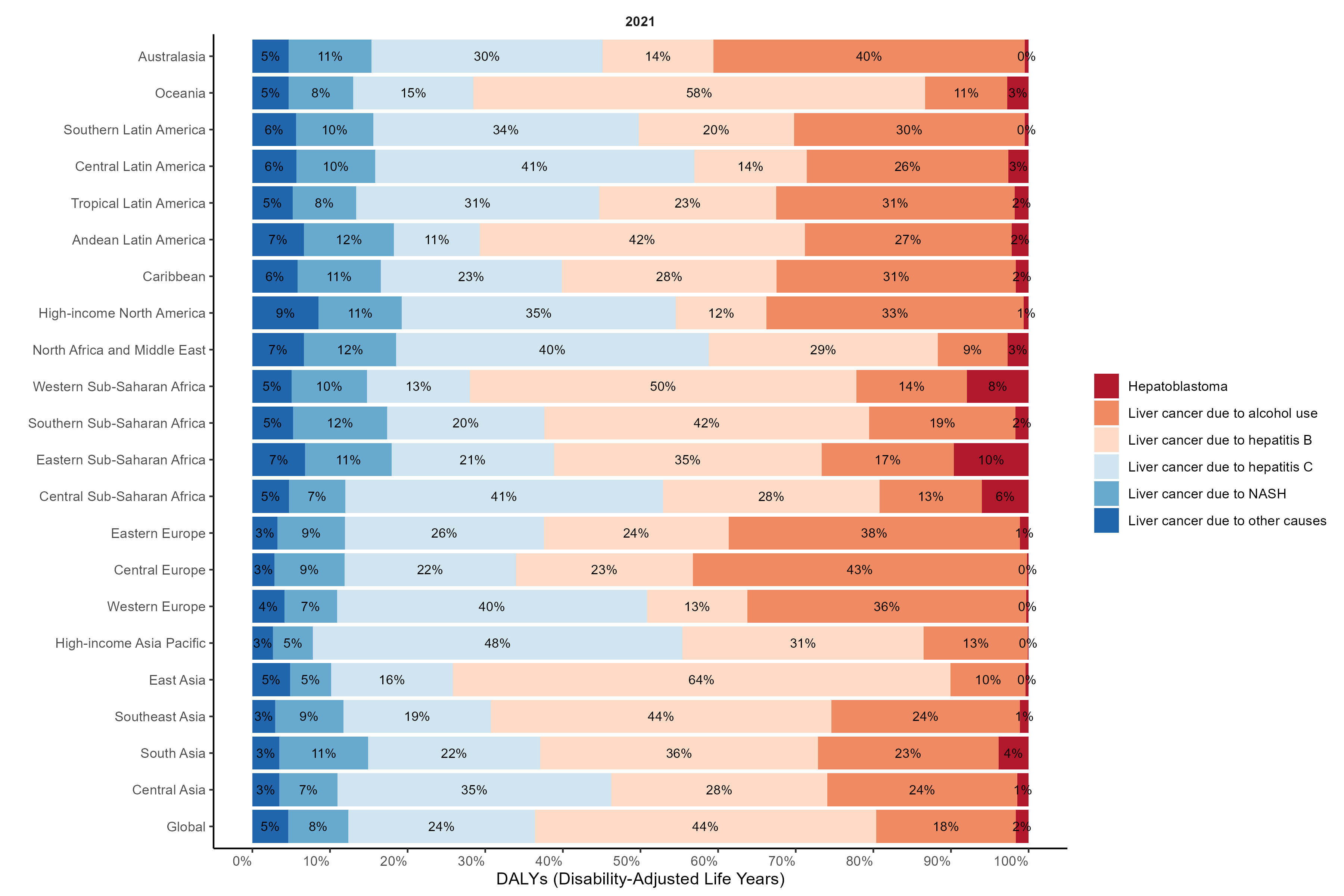


**H**

DALYs rate


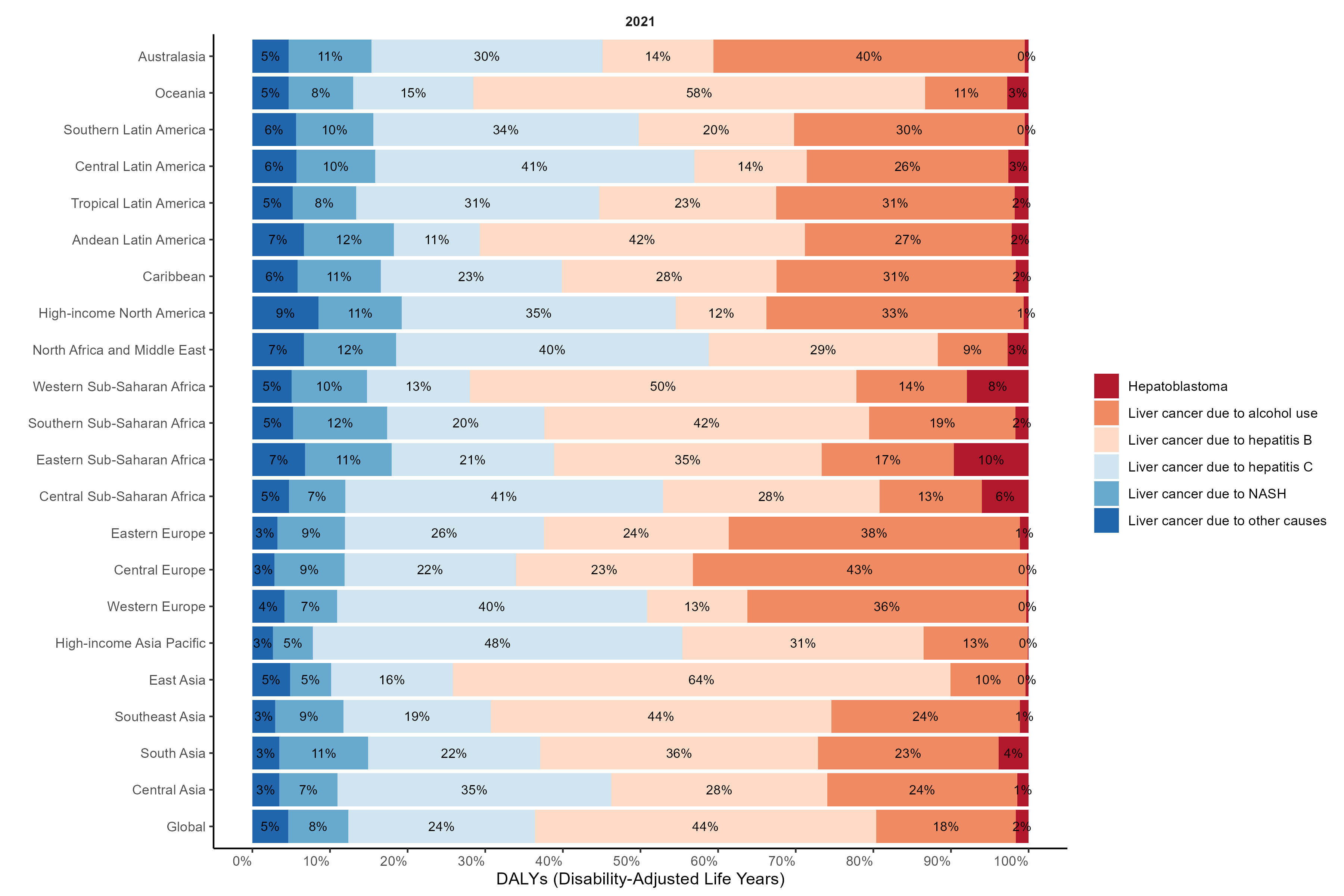


**I** DALYs rate


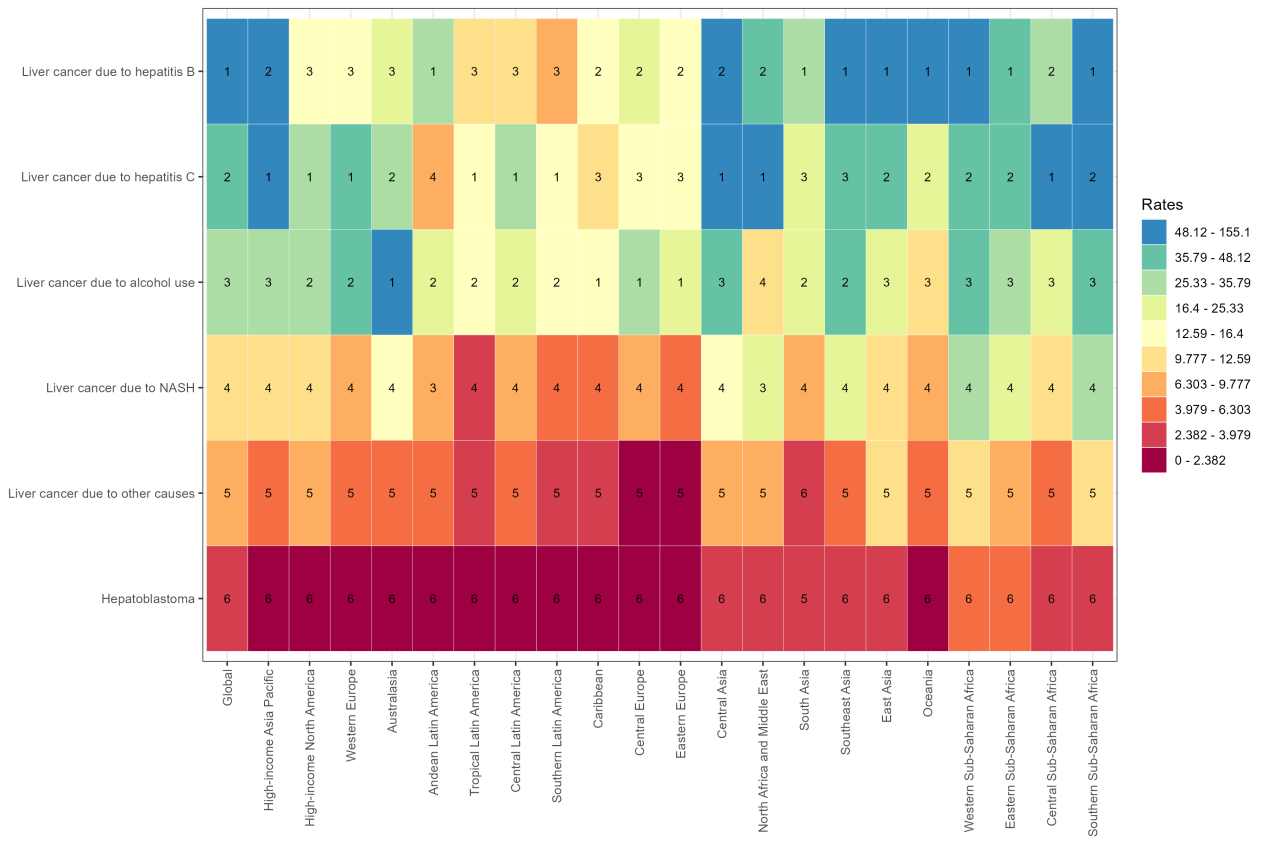


This figure demonstrates etiology-specific percentages (A, B, D, E, G, H) of liver cancer incidence, mortality, and disability-adjusted life years (DALYs) and ranking heatmap (C, F, I) in 2021.

**Figure 2. Age-specific number and percentages of liver cancer incidence, mortality, and disability-adjusted life years (DALYs) across six etiology in 2021**

**A**

Incidence number


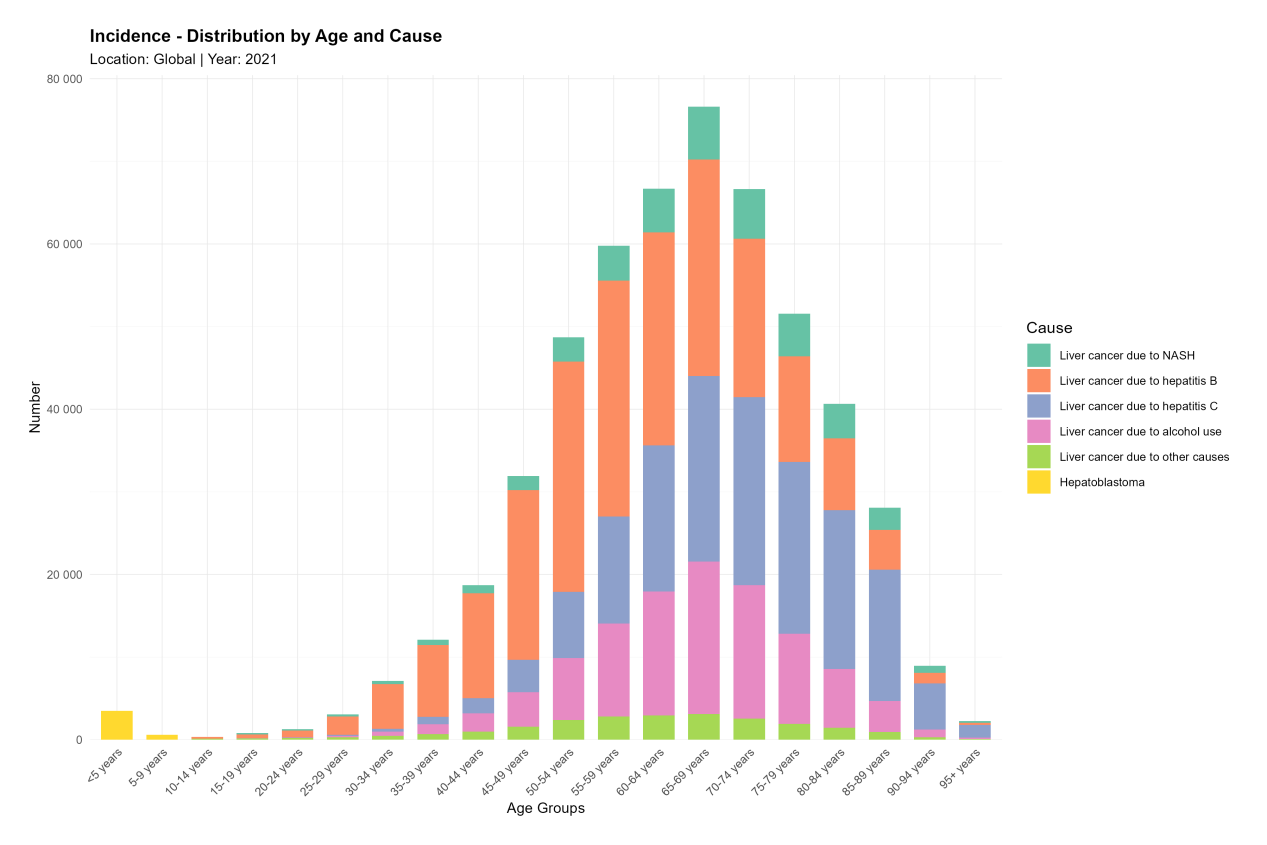


**B**


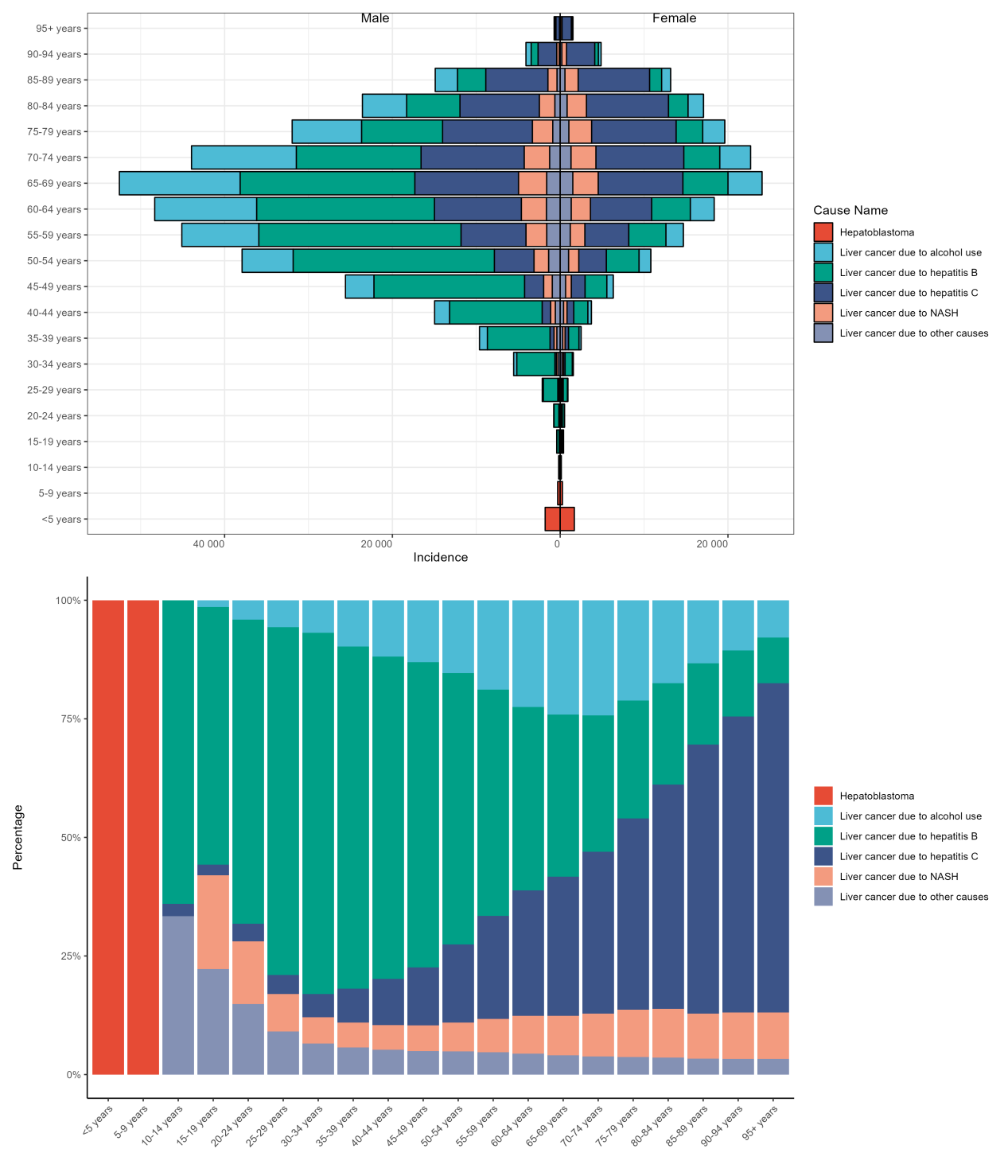


**C**

Death number


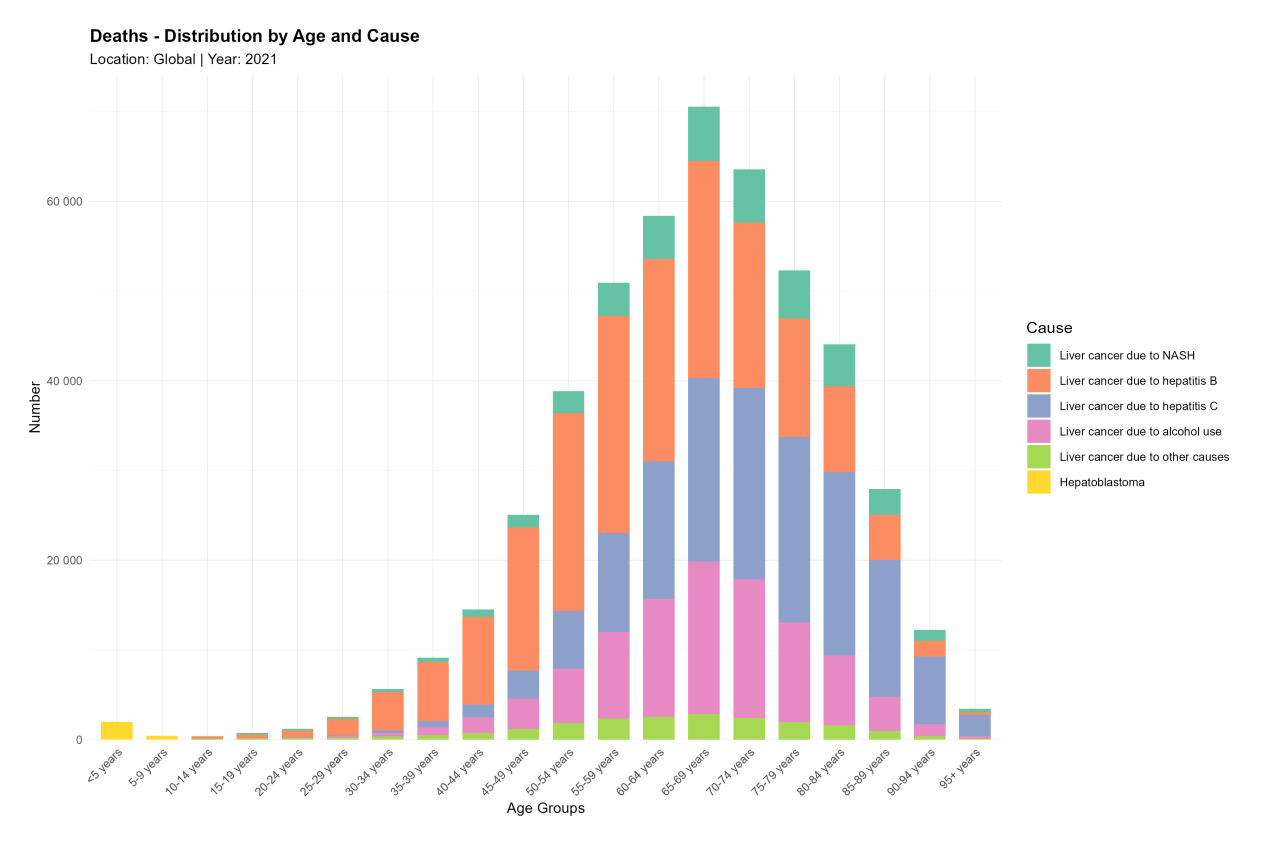


**D**


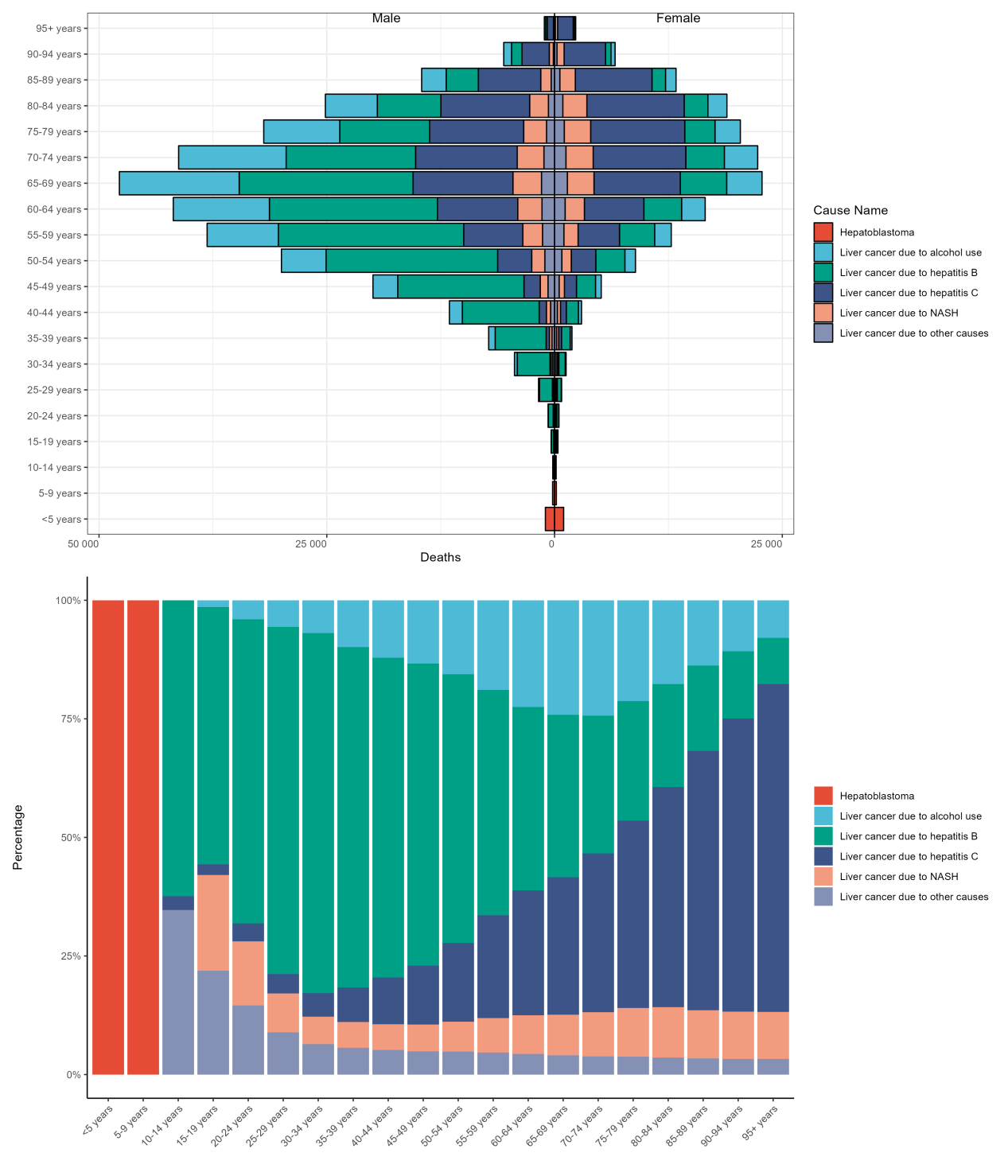


**E**

DALYs number


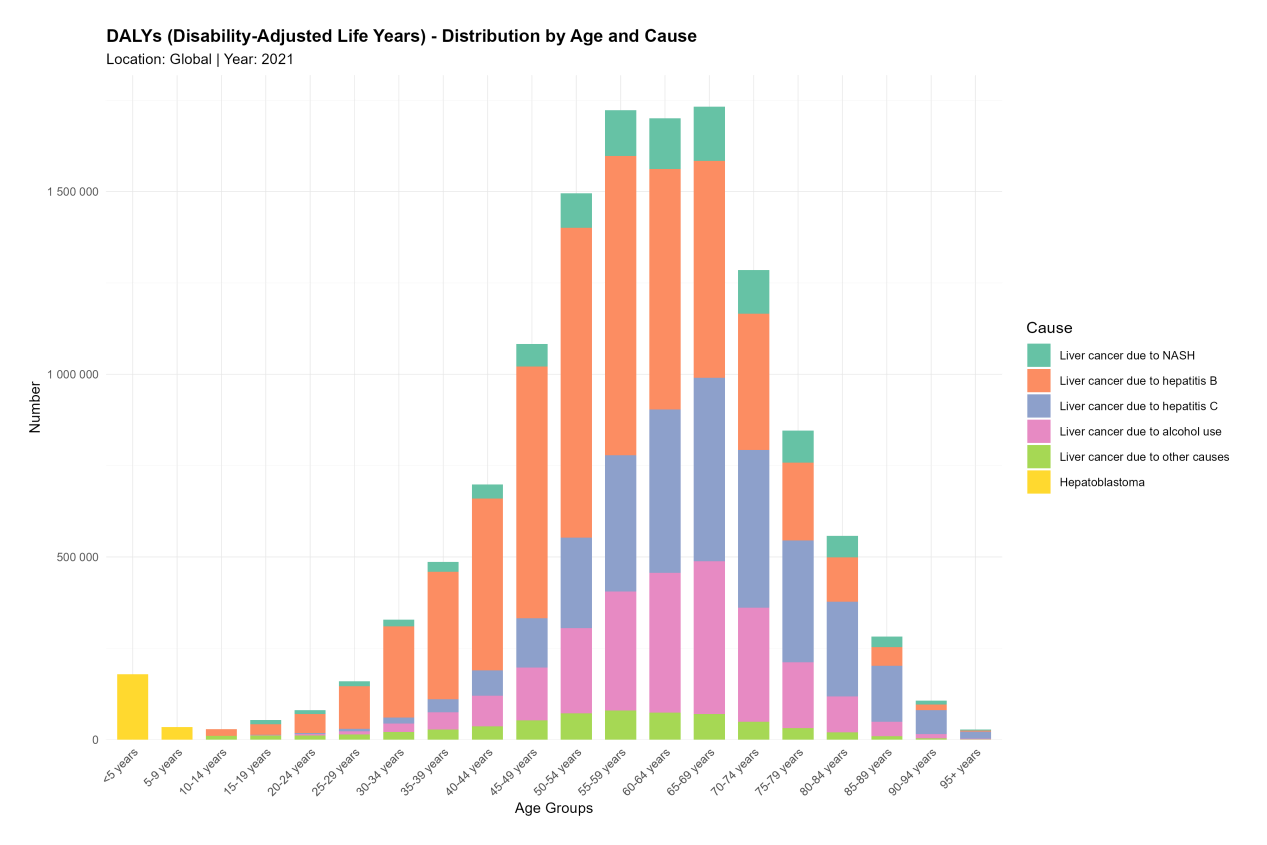


F


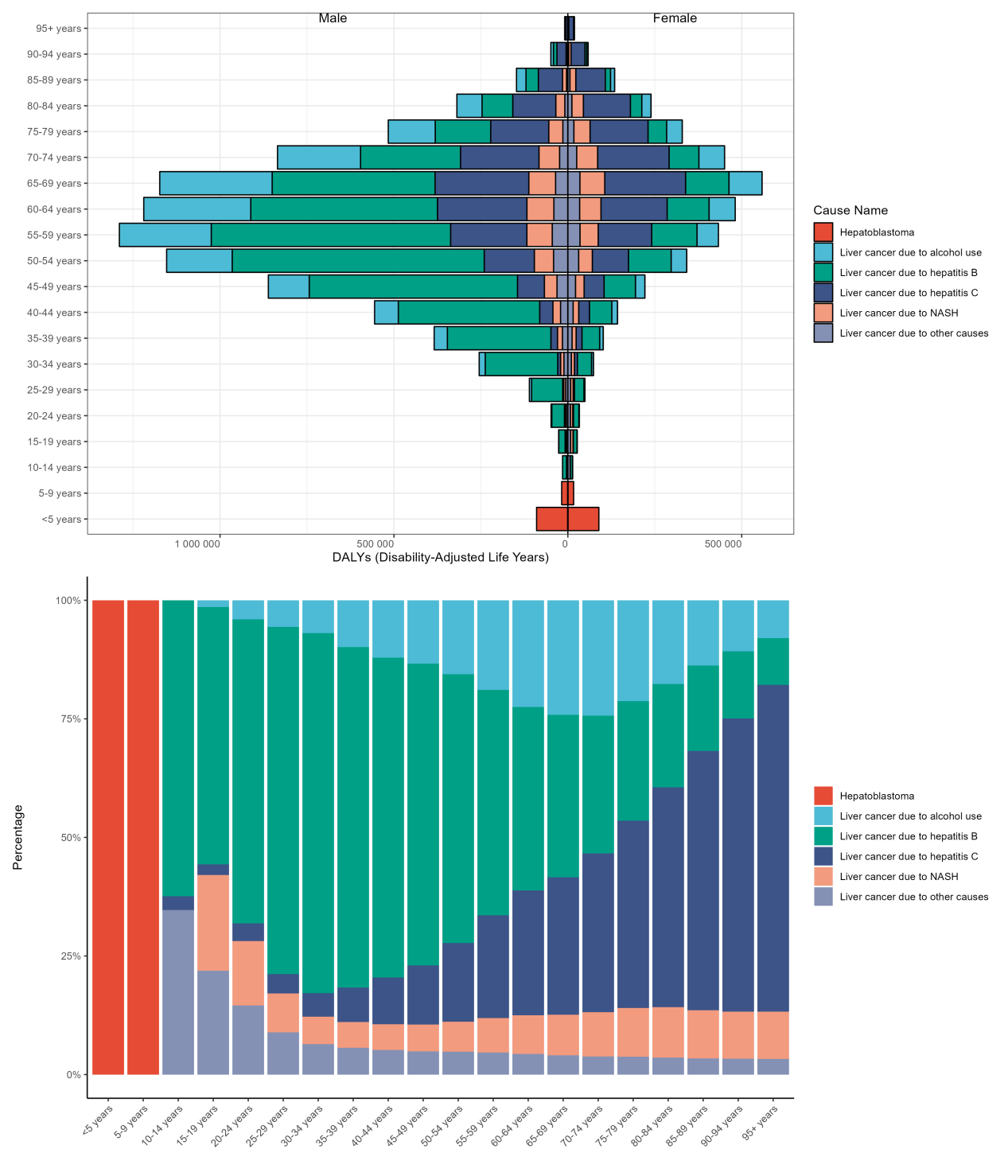


This figure demonstrates age-specific number (A, C, E) and percentages (B, D, F) of liver cancer incidence, mortality, and disability-adjusted life years (DALYs) across six etiology in 2021.

**Figure 3. Epidemiologic trends in liver cancer incidence, mortality, and disability-adjusted life years (DALYs) rates across six etiology from 1990 to 2021**

**A**

Incidence rate


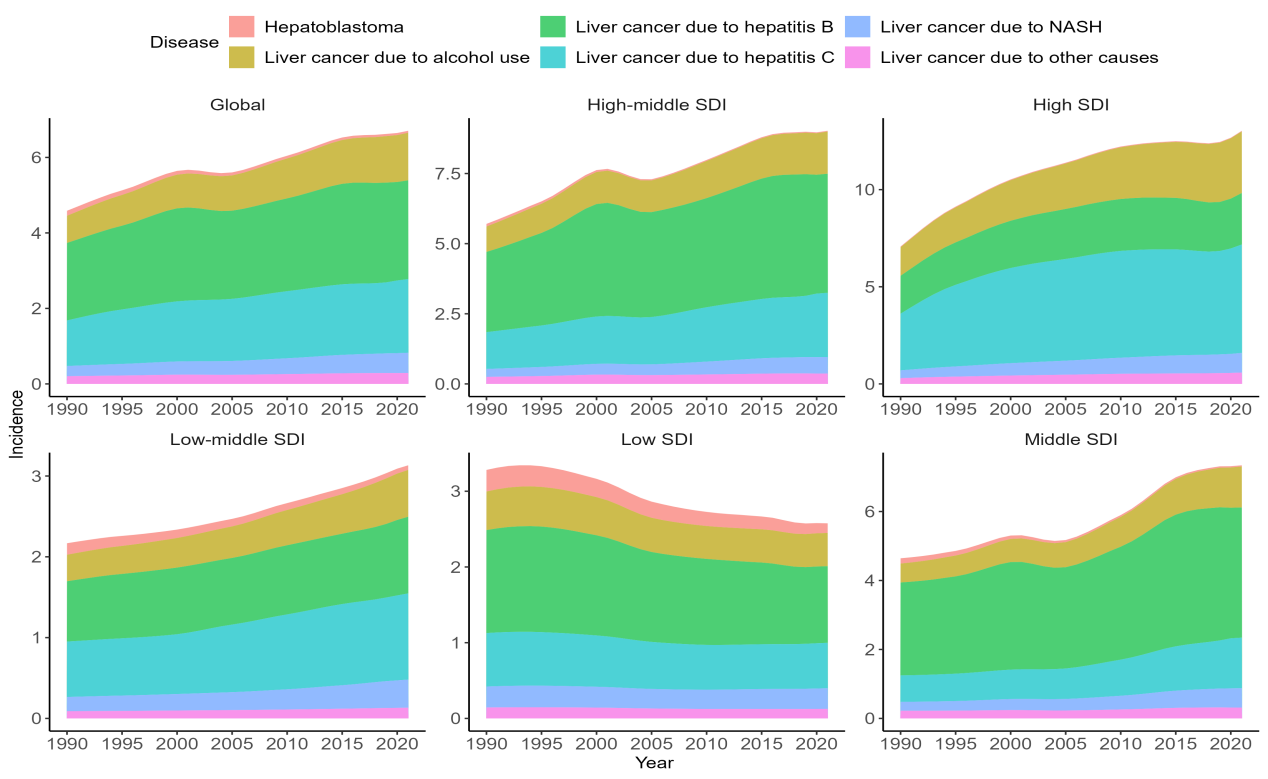


**B**

Deaths rate


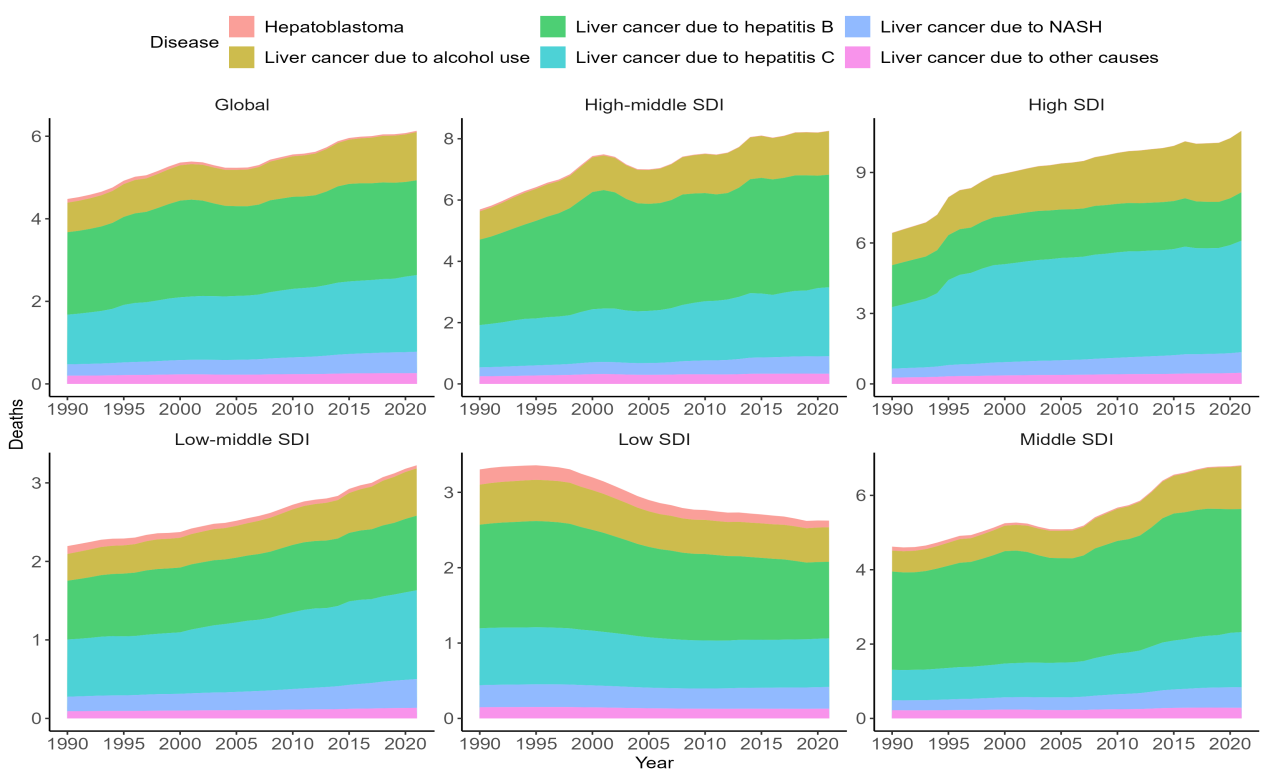


**C**

DALYs rate


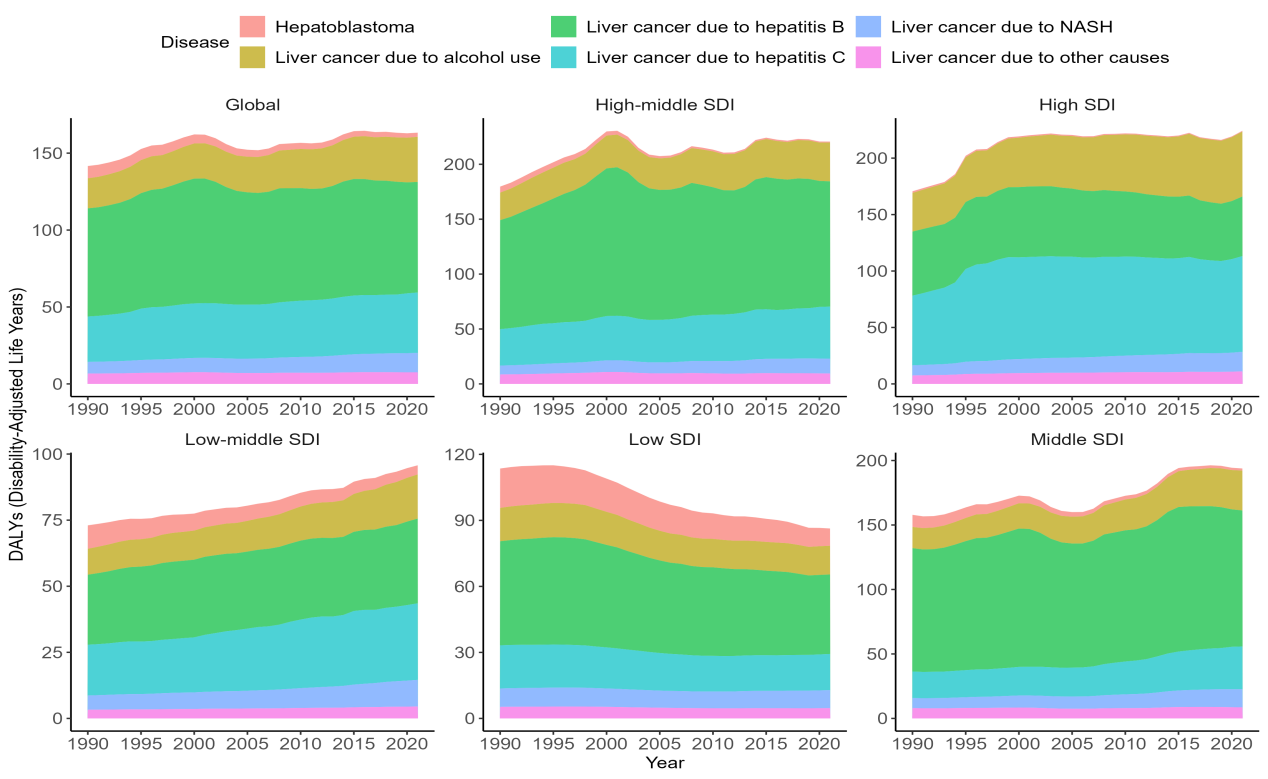


This figure demonstrates epidemiologic trends in liver cancer incidence (A), mortality (B), and disability-adjusted life years (DALYs) (C) rates across six etiology from 1990 to 2021.

**Figure 4. Global maps of rates and estimated annual percentage change (EAPC) of incidence, deaths, and disability-adjusted life-years (DALYs) attributable to liver cancer due to non-alcoholic steatohepatitis (NASH) in 2021**

**A**

**Liver cancer due to non-alcoholic steatohepatitis (NASH)**

Incidence rate


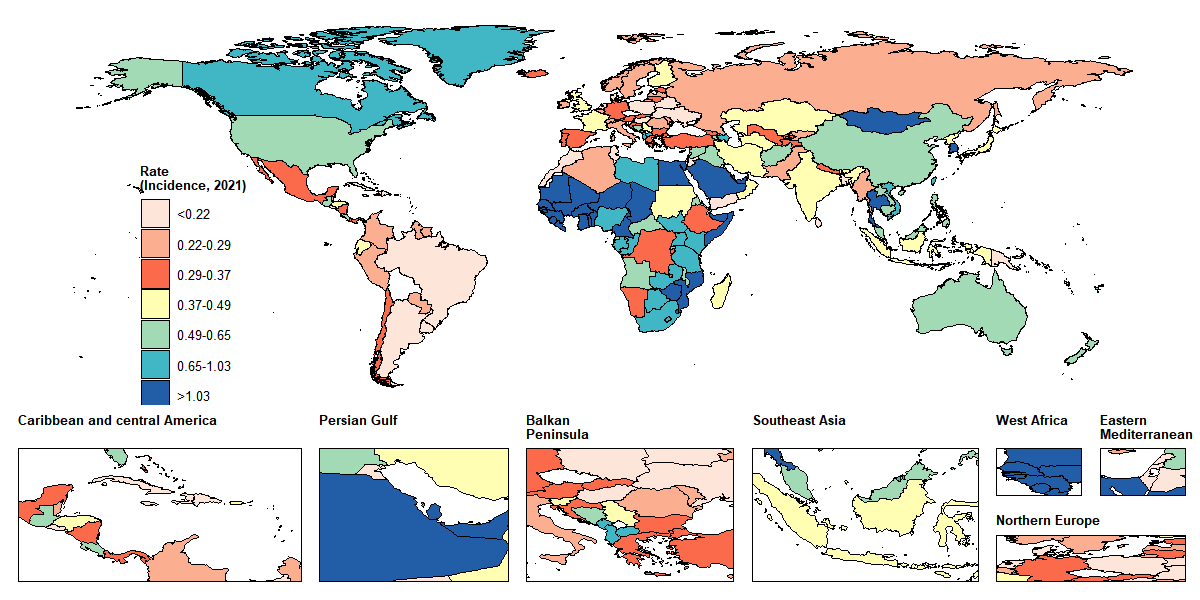


EAPC


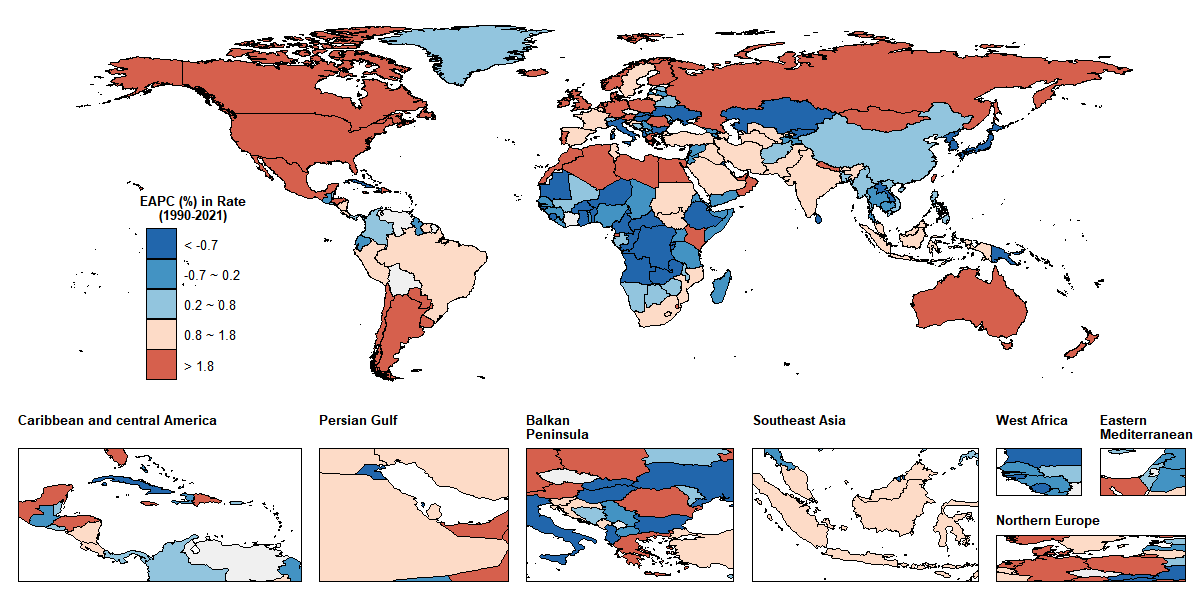


**B**

Deaths rate


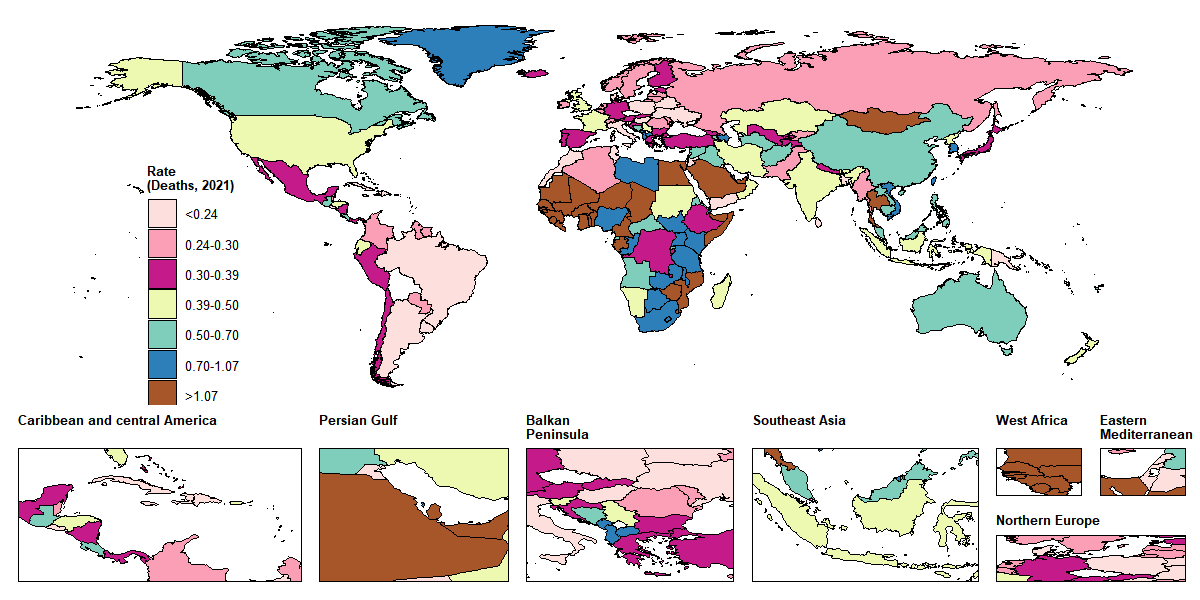


EAPC


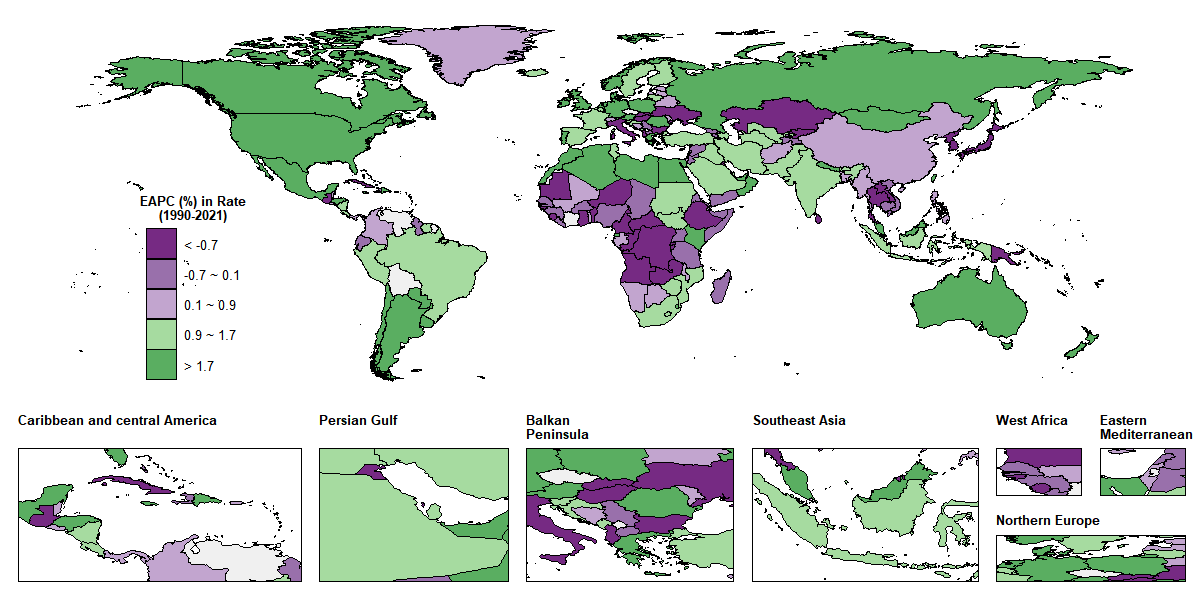


**C**

DALYs rate


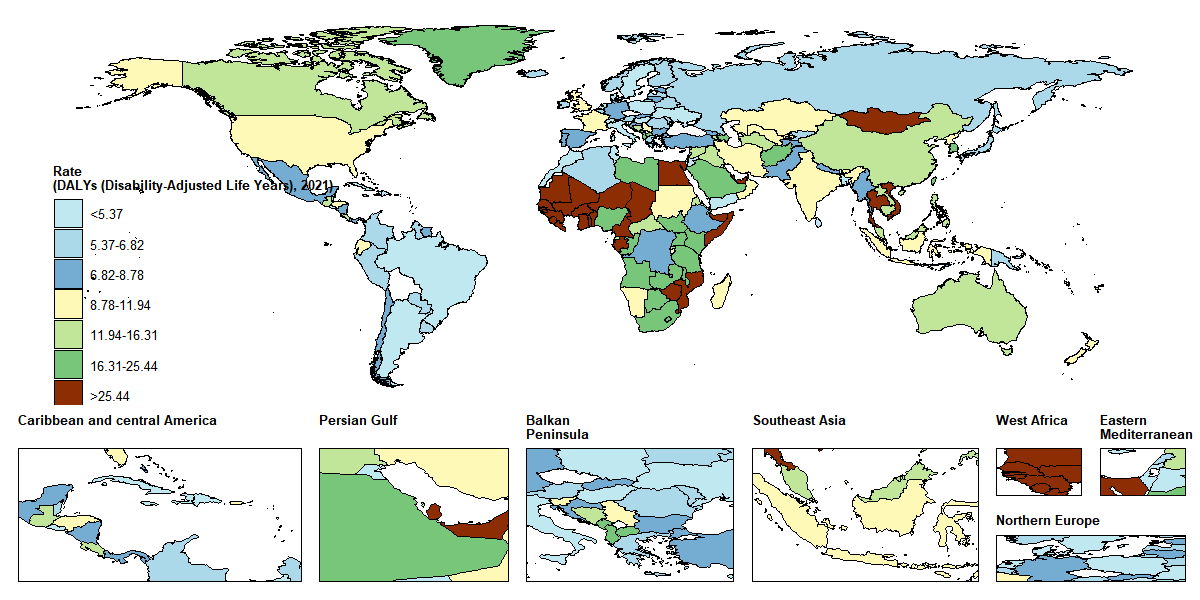


EAPC


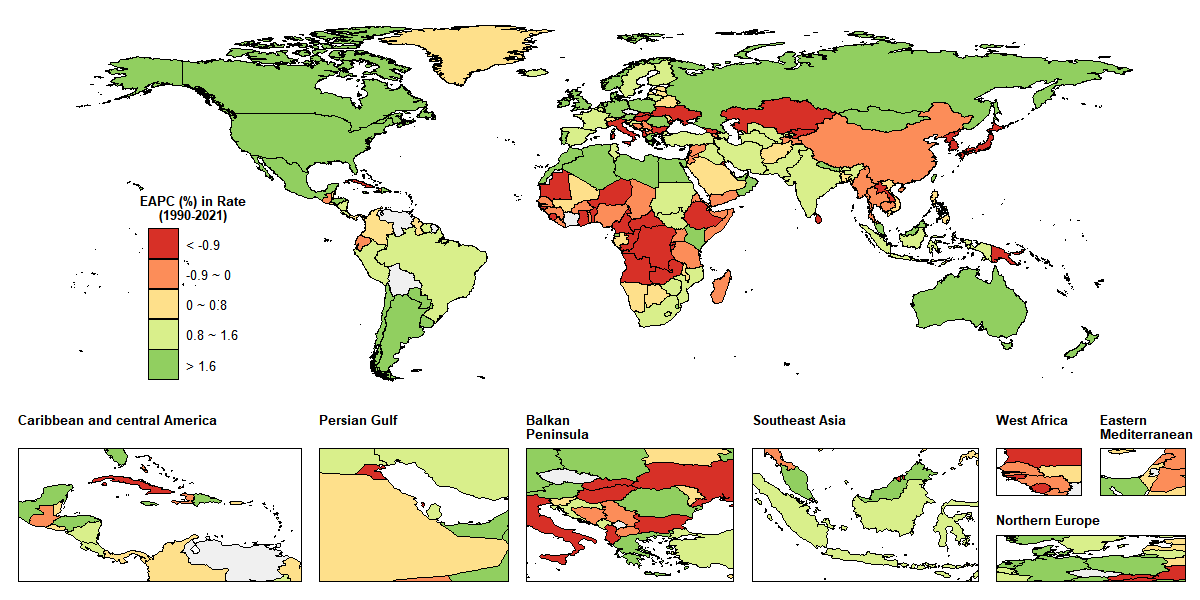


This figure demonstrates rates and estimated annual percentage change (EAPC) of incidence (A), deaths (B), and disability-adjusted life-years (DALYs) (C) attributable to liver cancer due to non-alcoholic steatohepatitis (NASH) across 204 countries and territories in 2021.

**Figure 5. Global maps of rates and estimated annual percentage change (EAPC) of incidence, deaths, and disability-adjusted life-years (DALYs) attributable to liver cancer due to hepatitis B in 2021**

**A**

**Liver cancer due to hepatitis B**

Incidence rate


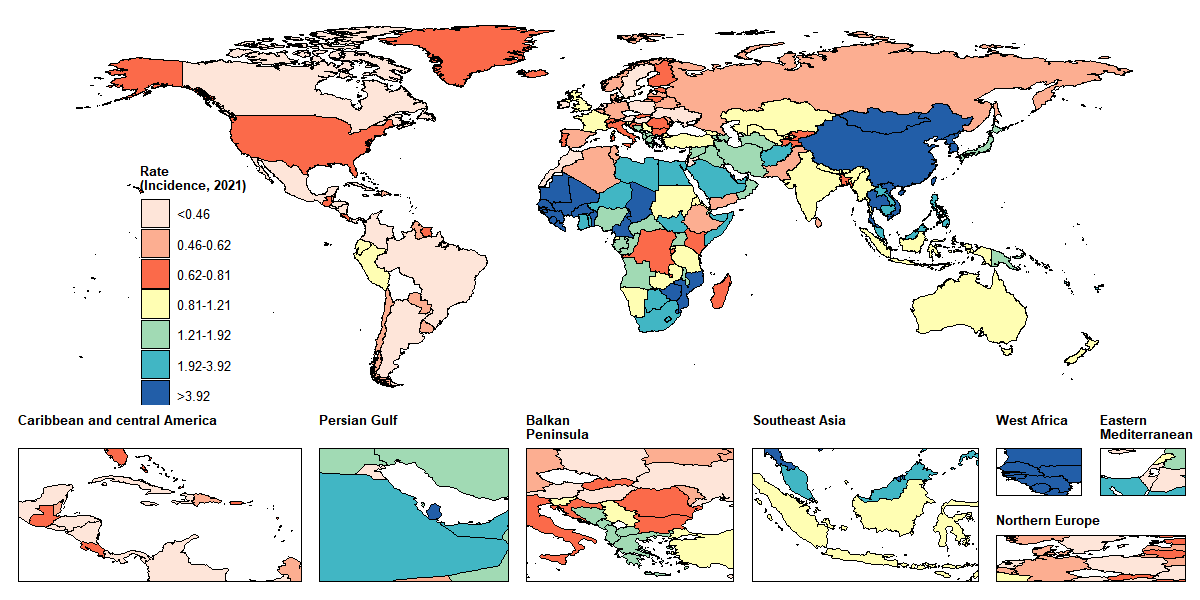


EAPC


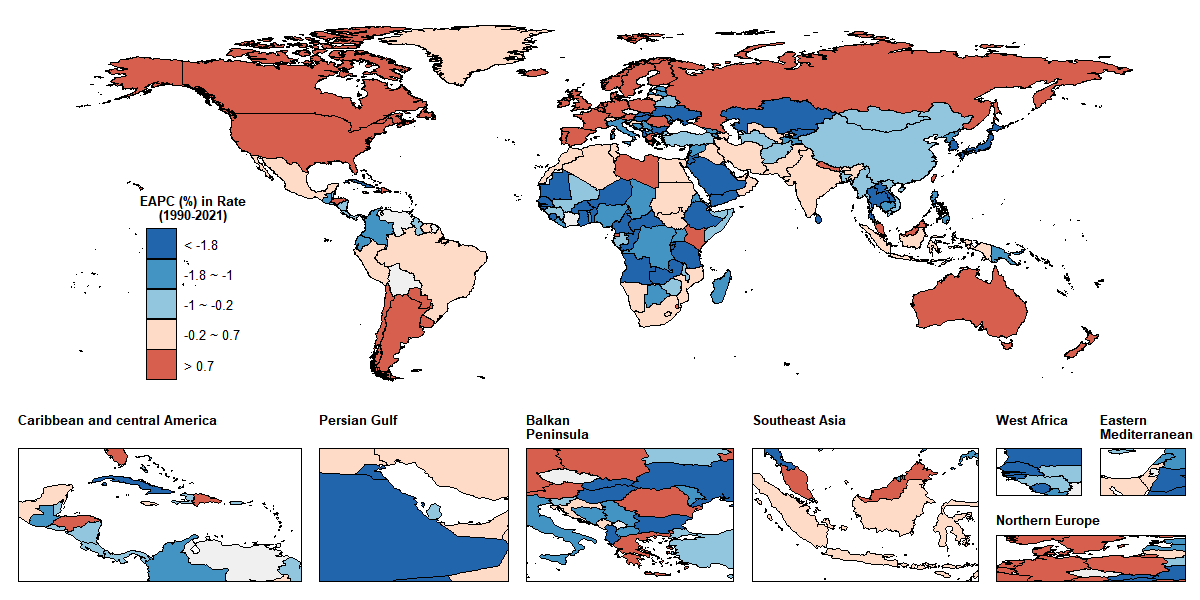


**B**

Deaths rate


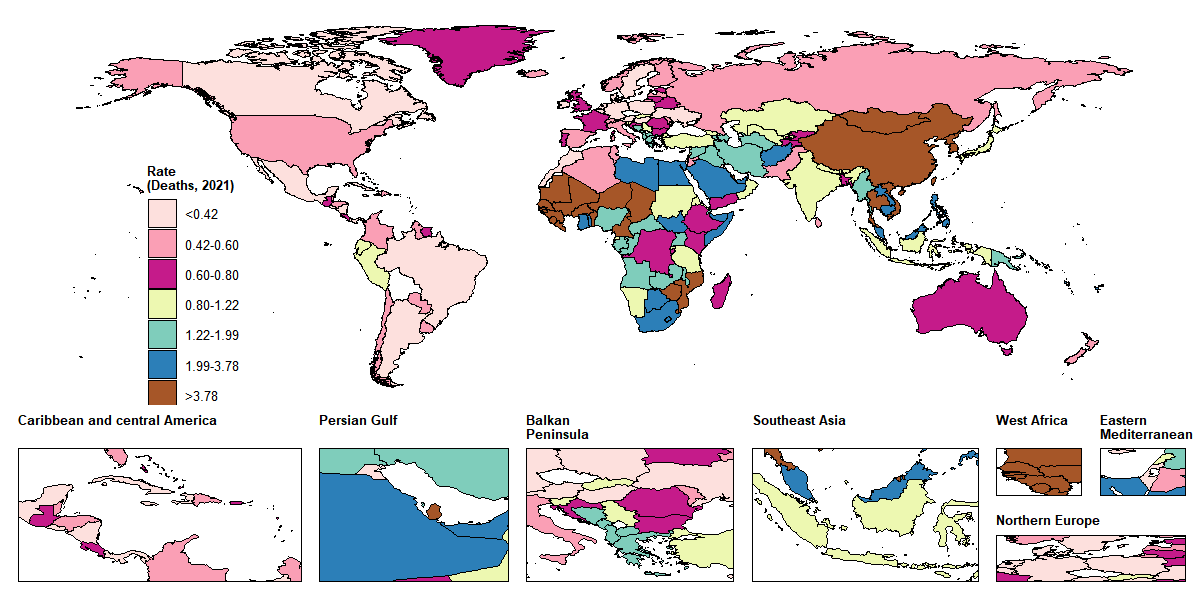


EAPC


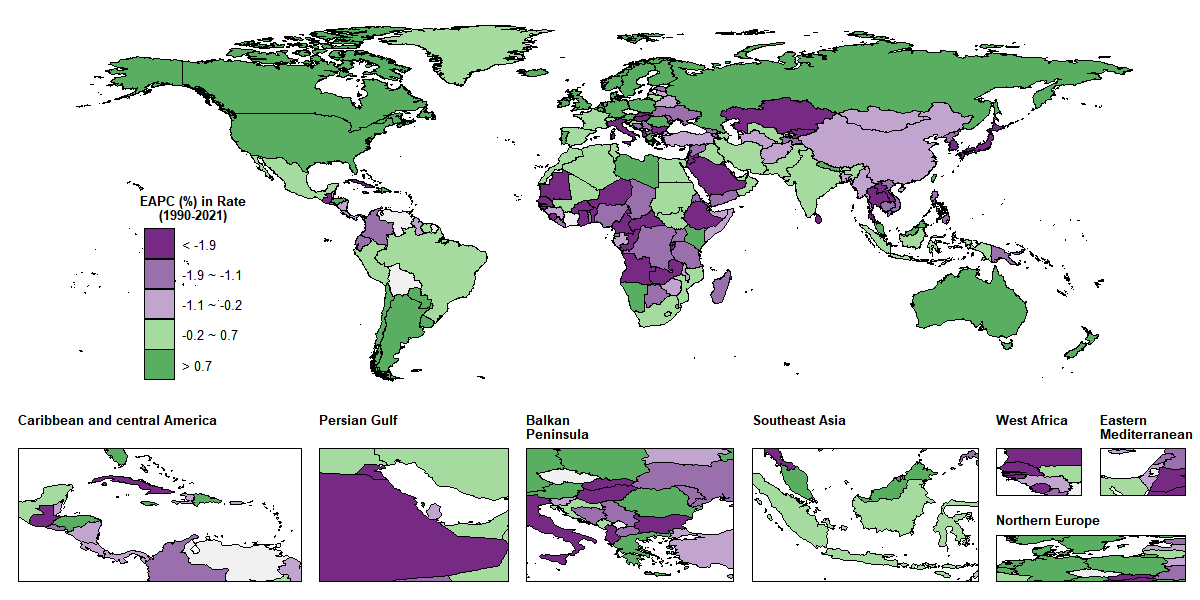


**C**

DALYs rate


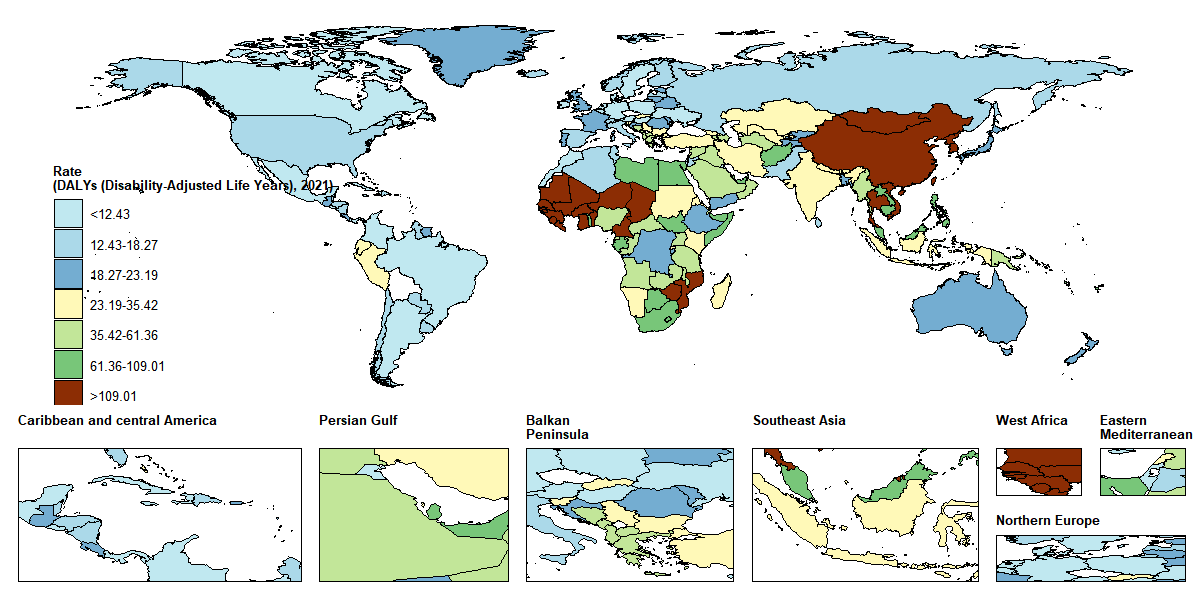


EAPC


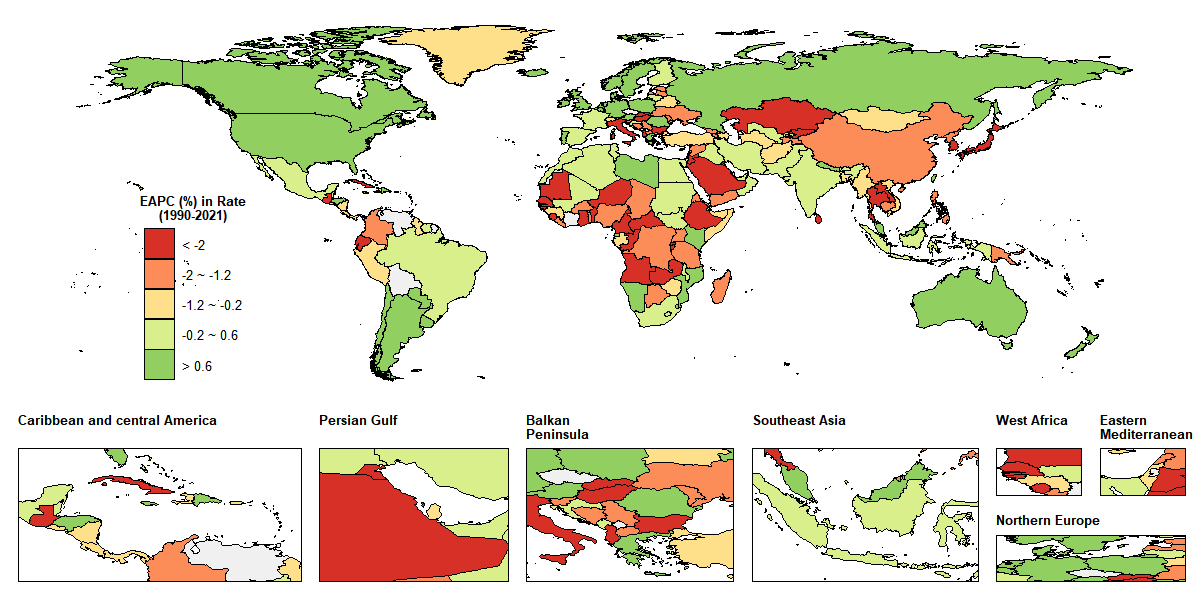


This figure demonstrates rates and estimated annual percentage change (EAPC) of incidence (A), deaths (B), and disability-adjusted life-years (DALYs) (C) attributable to liver cancer due to hepatitis B across 204 countries and territories in 2021.

**Figure 6. Global maps of rates and estimated annual percentage change (EAPC) of incidence, deaths, and disability-adjusted life-years (DALYs) attributable to liver cancer due to hepatitis C in 2021**

**A**

**Liver cancer due to hepatitis C**

Incidence rate


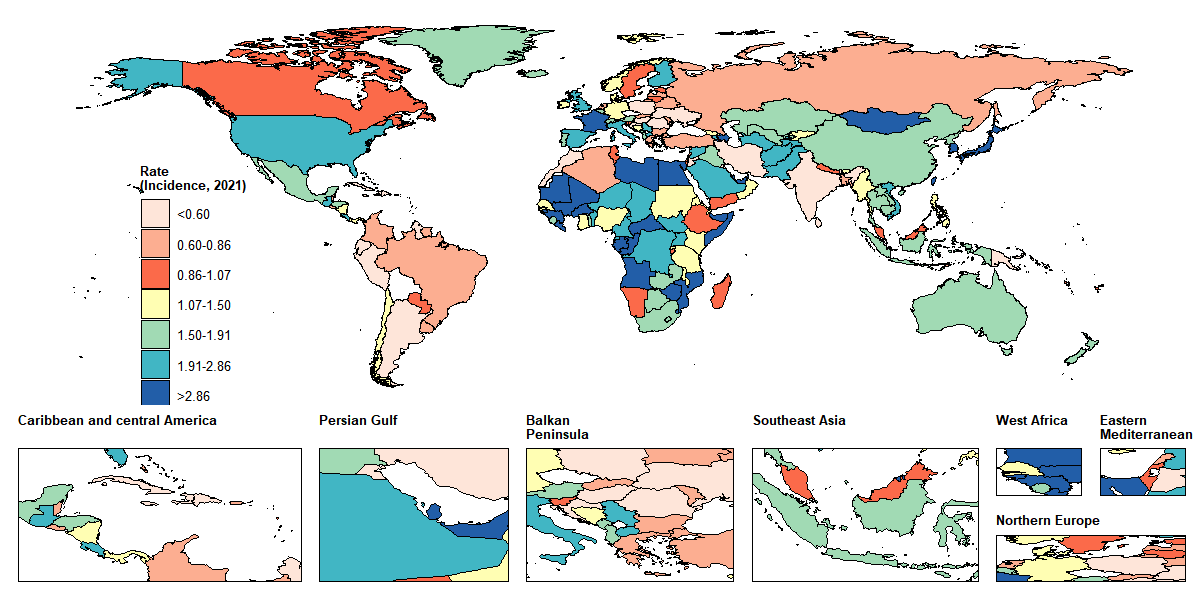


EAPC


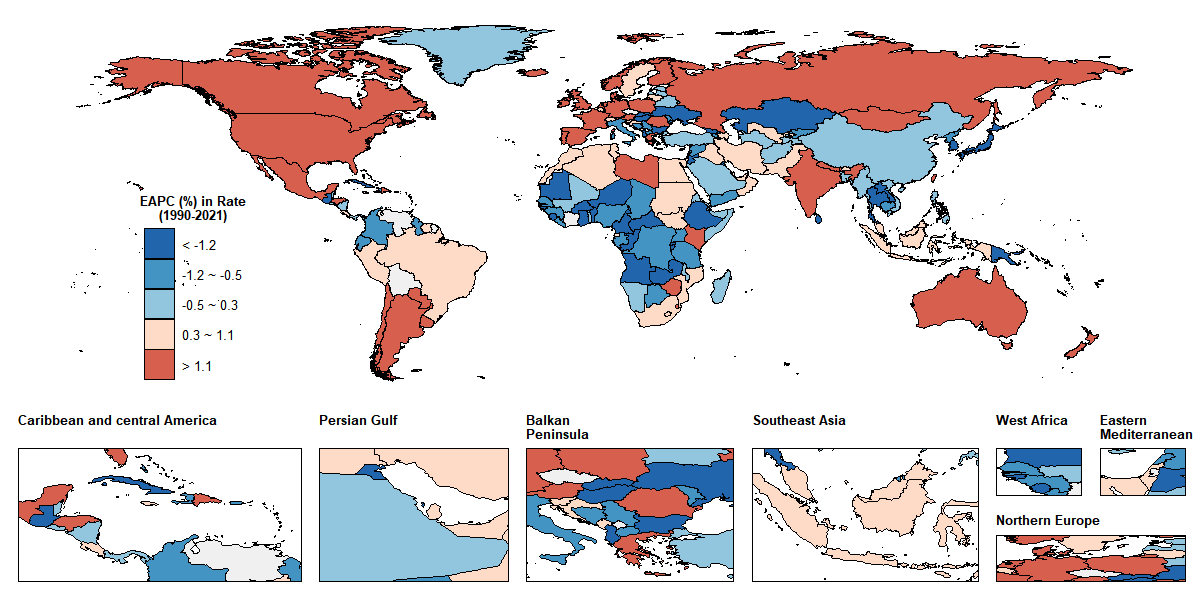


**B**

Deaths rate


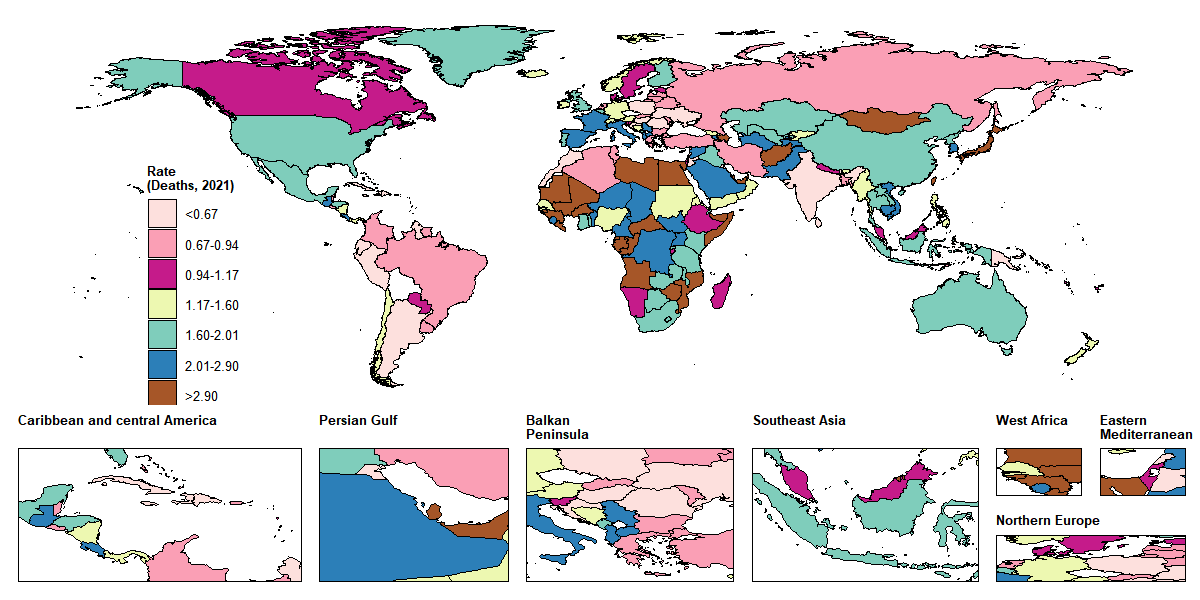


EAPC


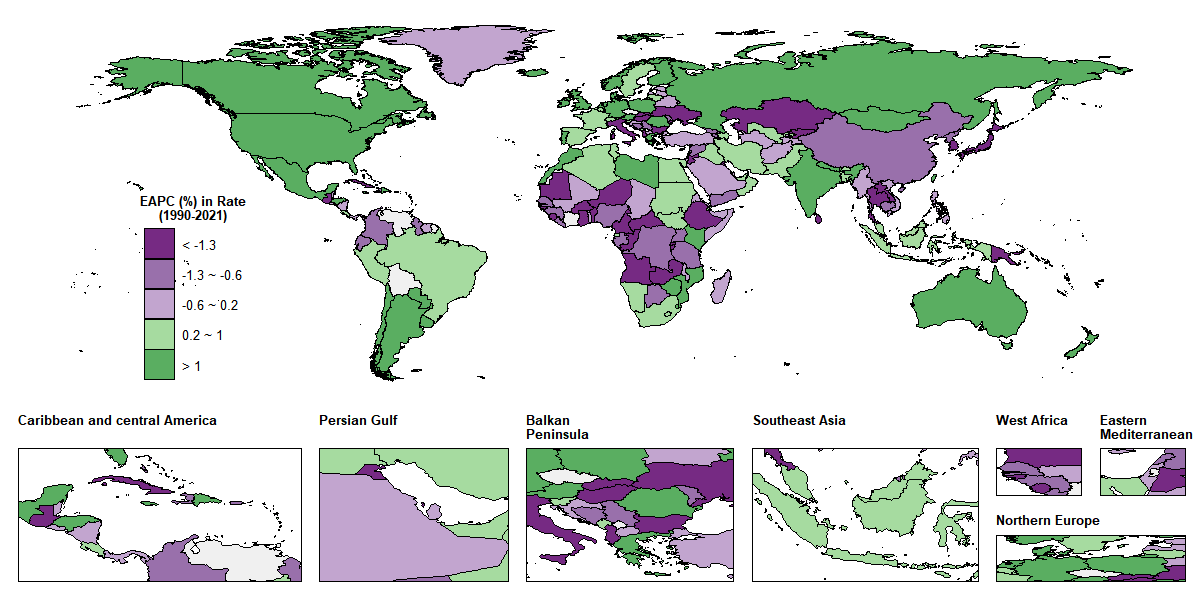


**C**

DALYs rate


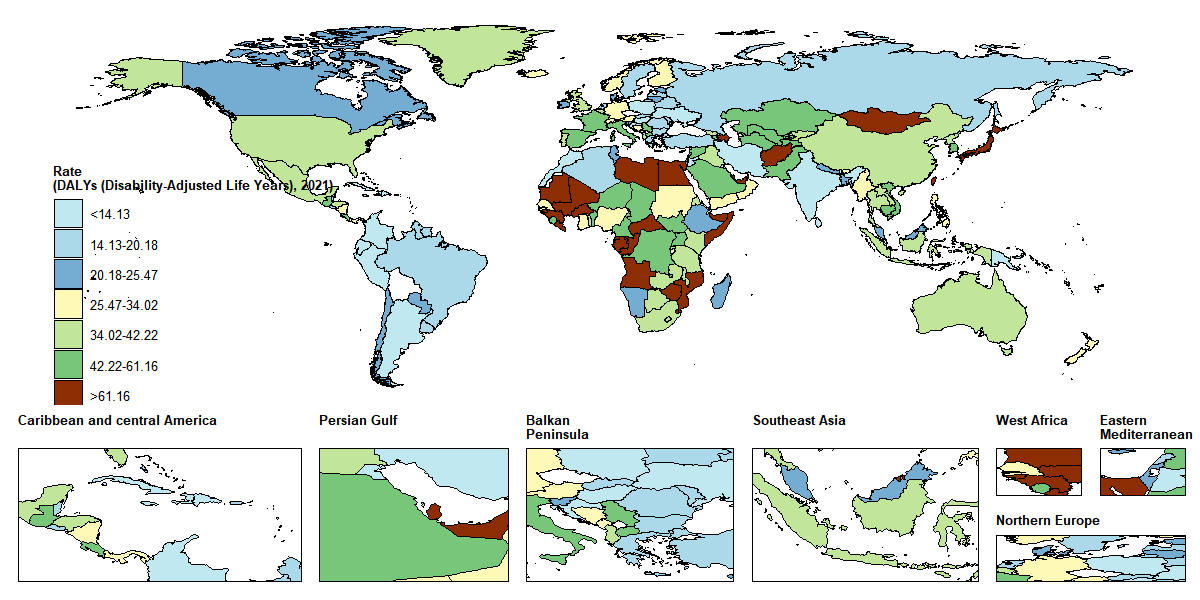


EAPC


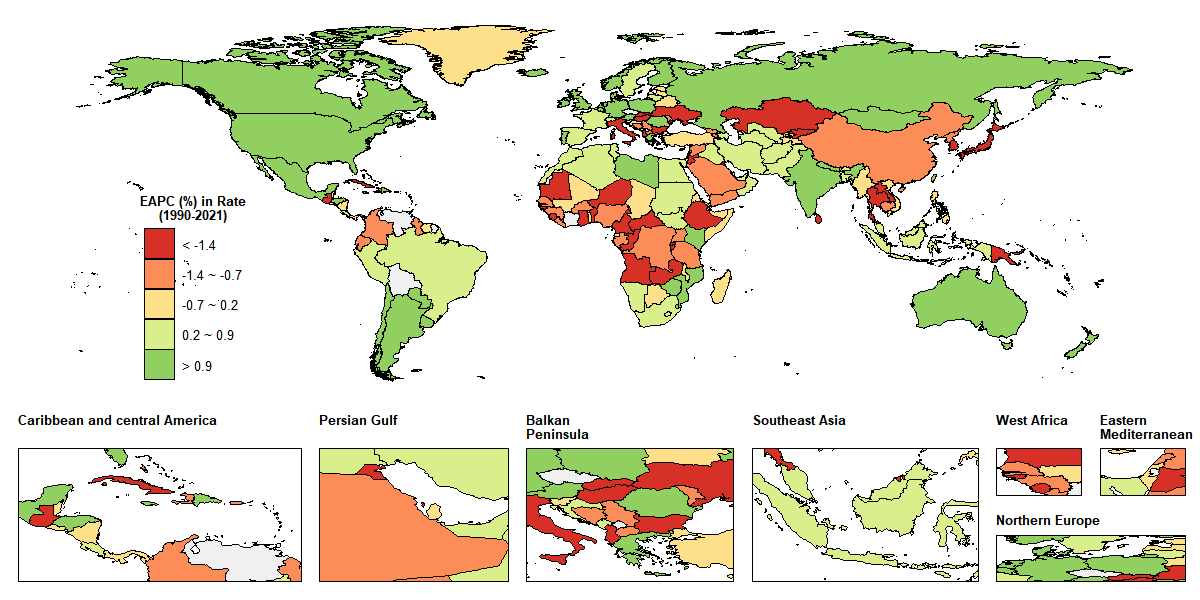


This figure demonstrates rates and estimated annual percentage change (EAPC) of incidence (A), deaths (B), and disability-adjusted life-years (DALYs) (C) attributable to liver cancer due to hepatitis C across 204 countries and territories in 2021.

**Figure 7. Global maps of rates and estimated annual percentage change (EAPC) of incidence, deaths, and disability-adjusted life-years (DALYs) attributable to liver cancer due to alcohol use in 2021**

**A**

**Liver cancer due to alcohol use**

Incidence rate


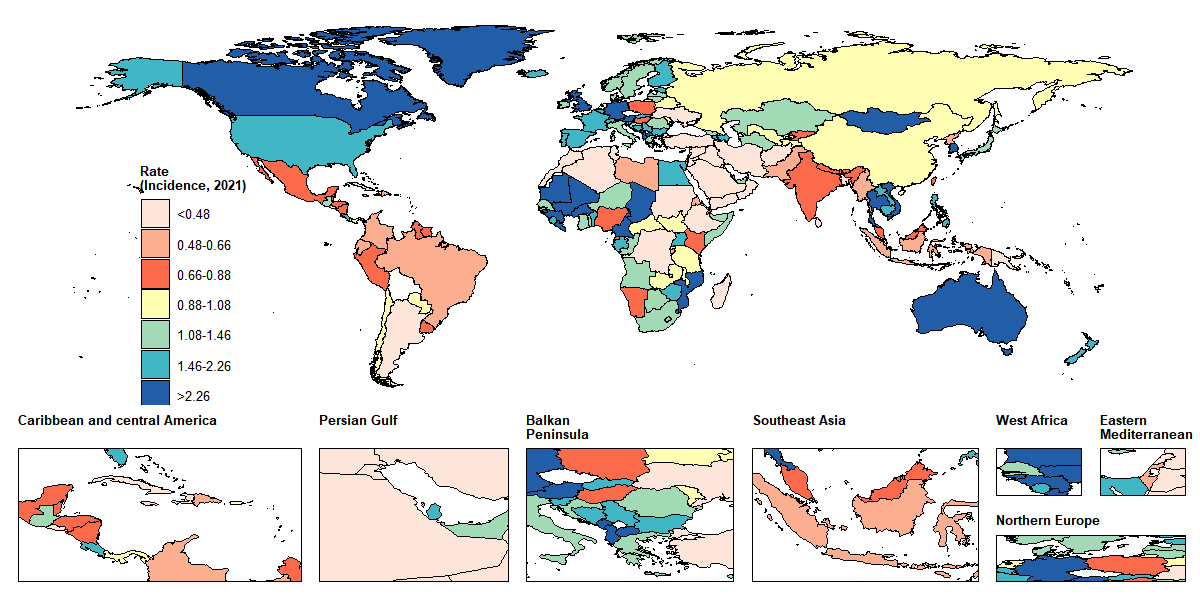


EAPC


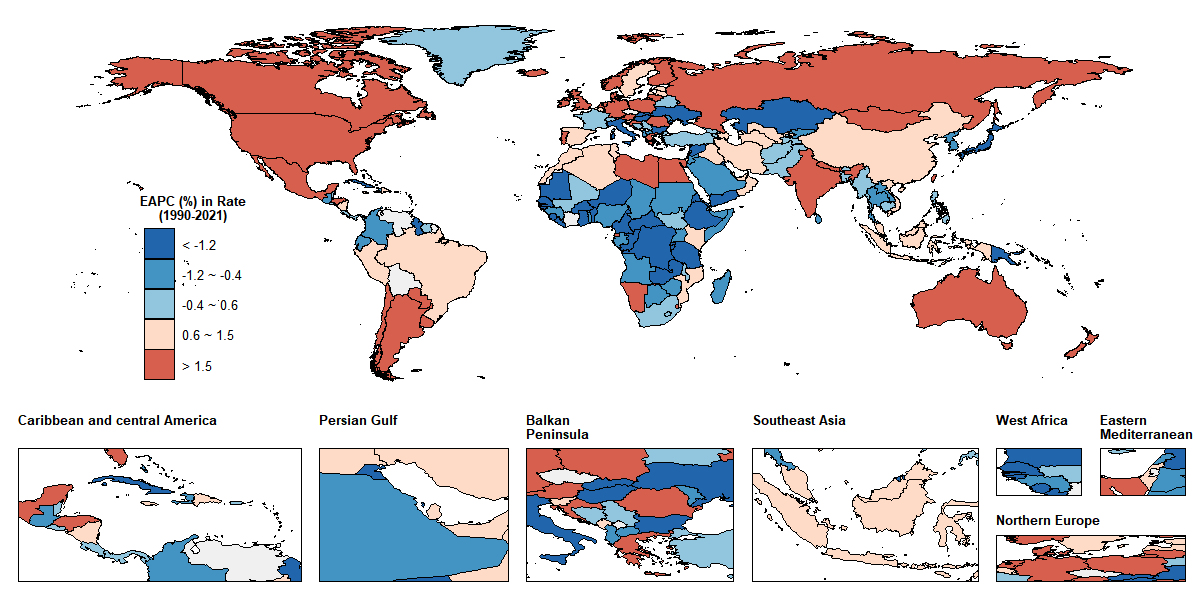


**B**

Deaths rate


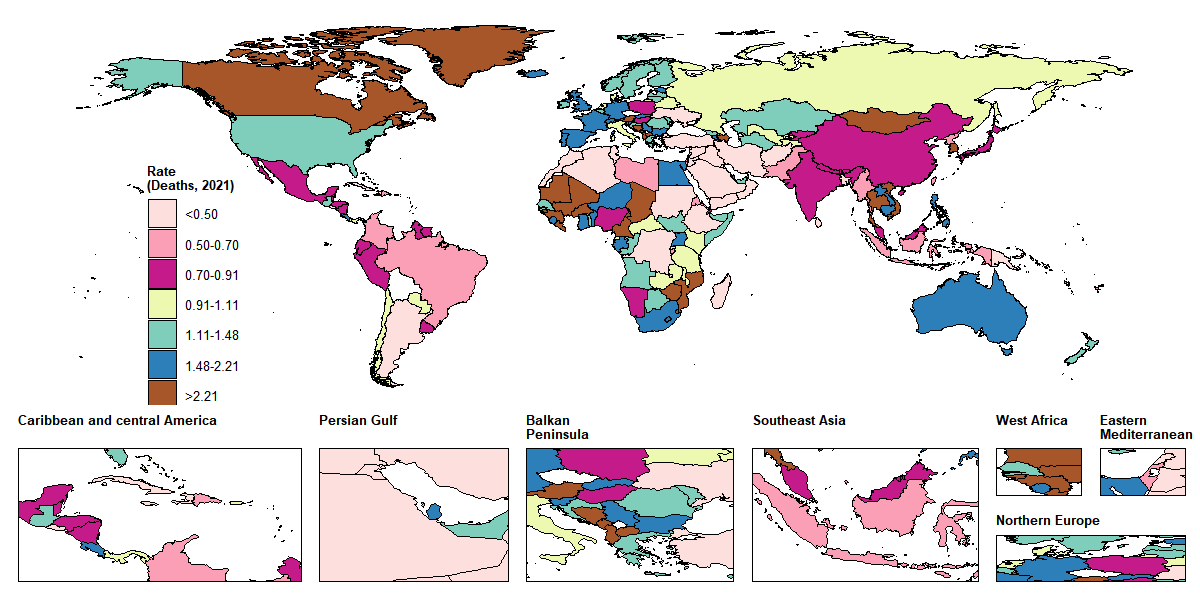


EAPC


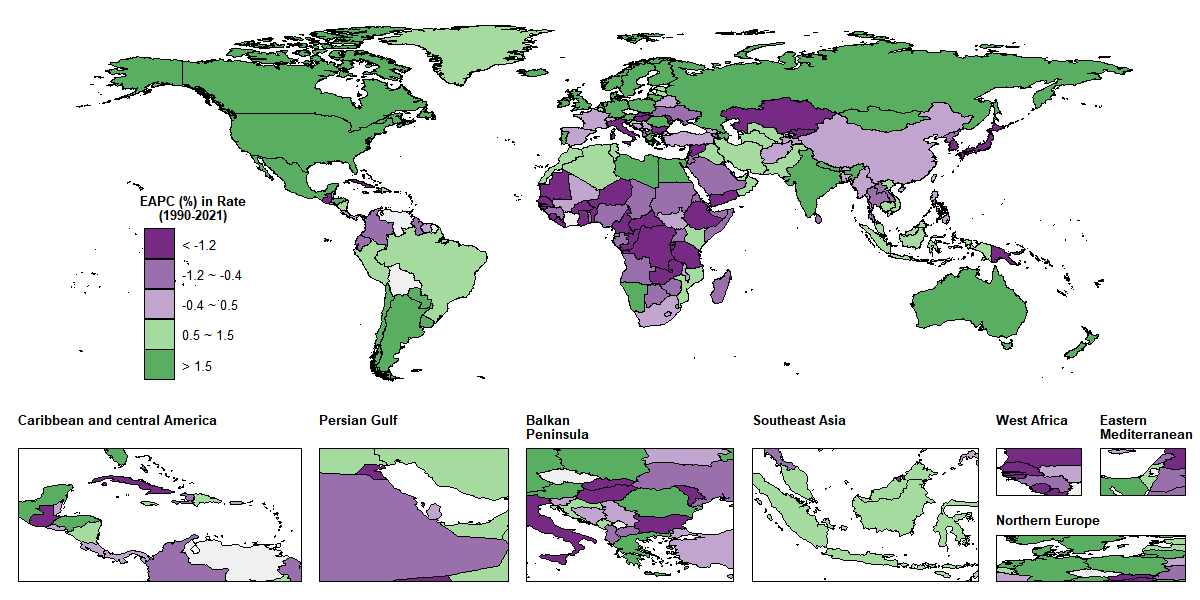


**C**

DALYs rate


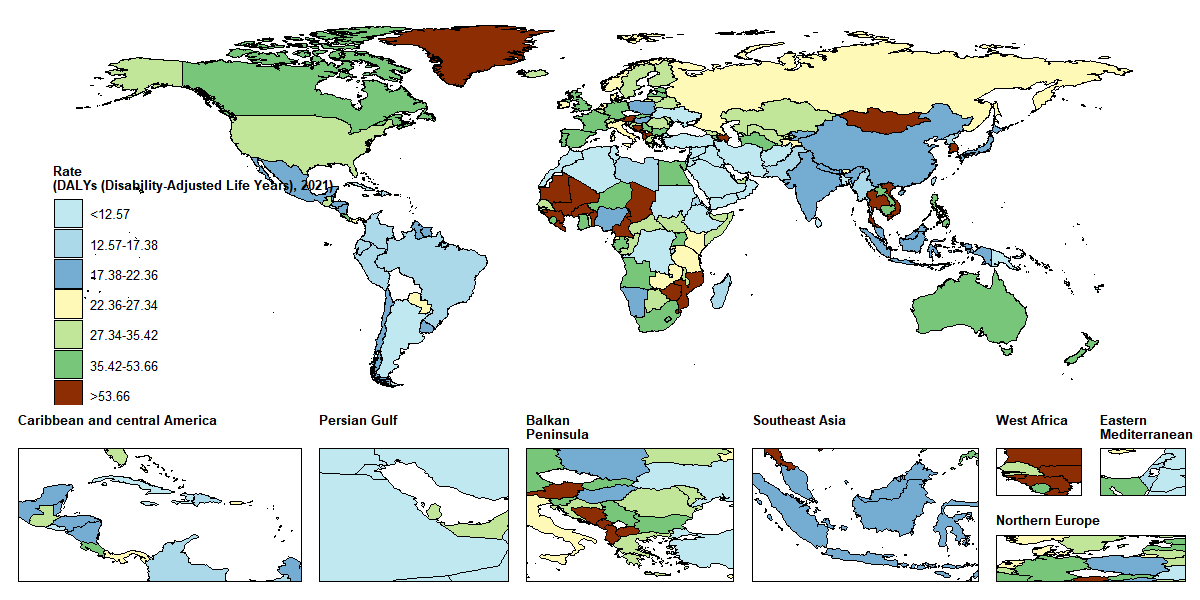


EAPC


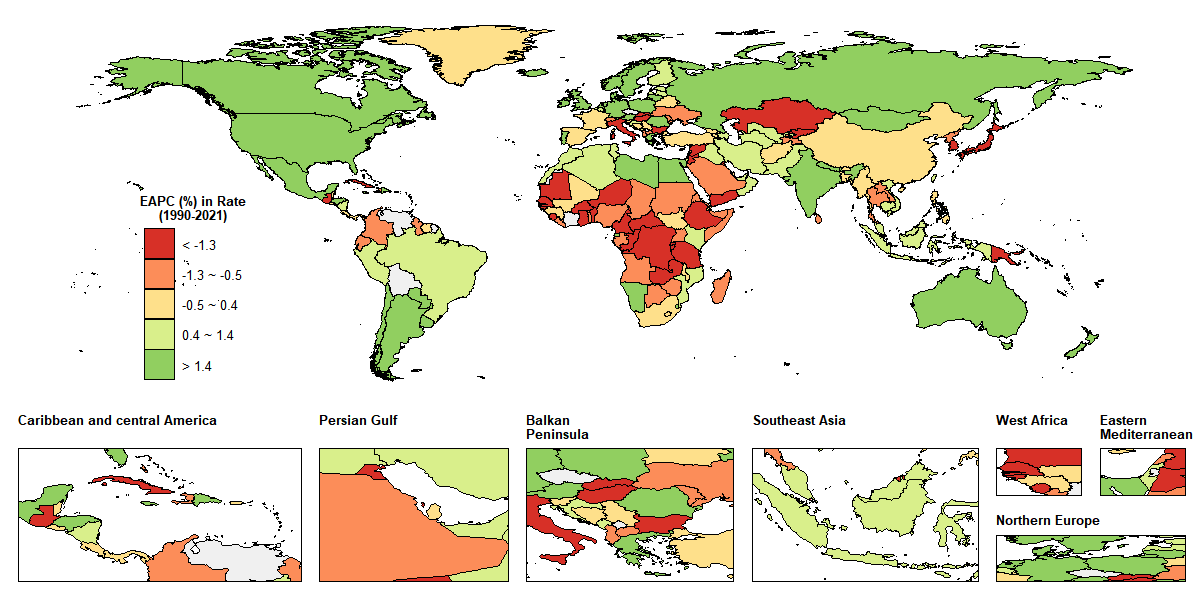


This figure demonstrates rates and estimated annual percentage change (EAPC) of incidence (A), deaths (B), and disability-adjusted life-years (DALYs) (C) attributable to liver cancer due to alcohol use across 204 countries and territories in 2021.

**Figure 8. Global maps of rates and estimated annual percentage change (EAPC) of incidence, deaths, and disability-adjusted life-years (DALYs) attributable to hepatoblastoma in 2021**

**A**

**Hepatoblastoma**

Incidence rate


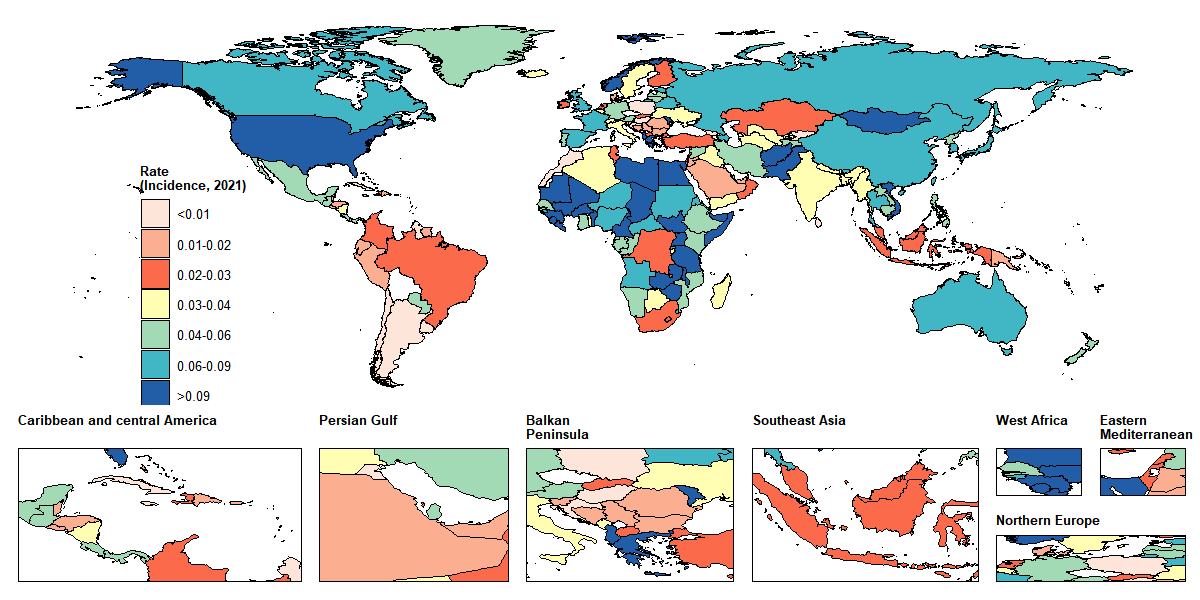


EAPC


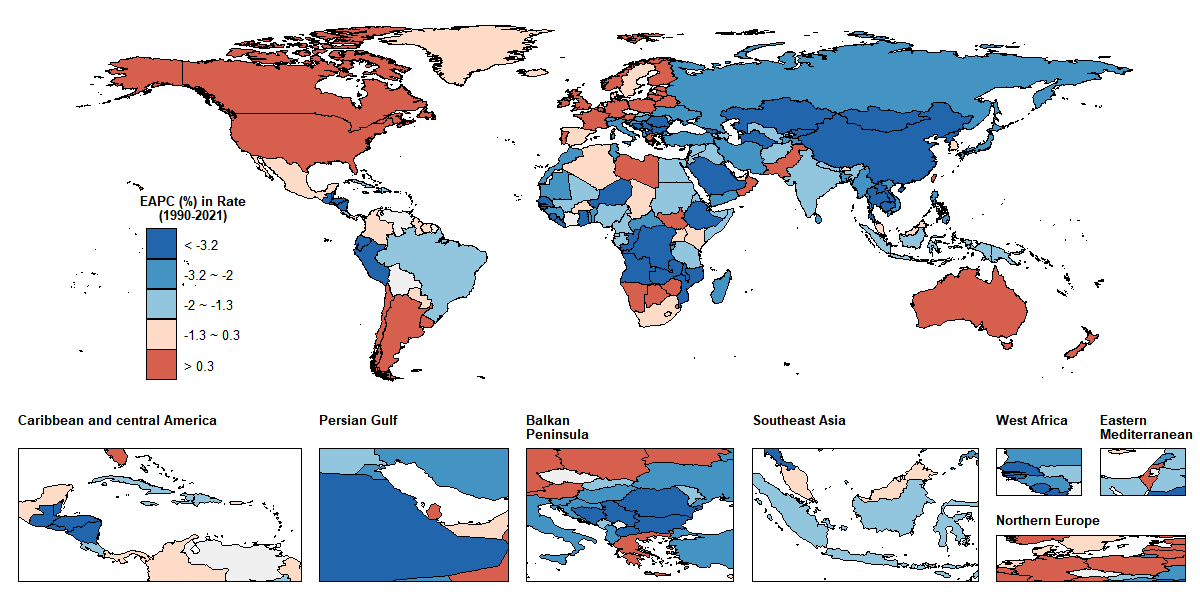


**B**

Deaths rate


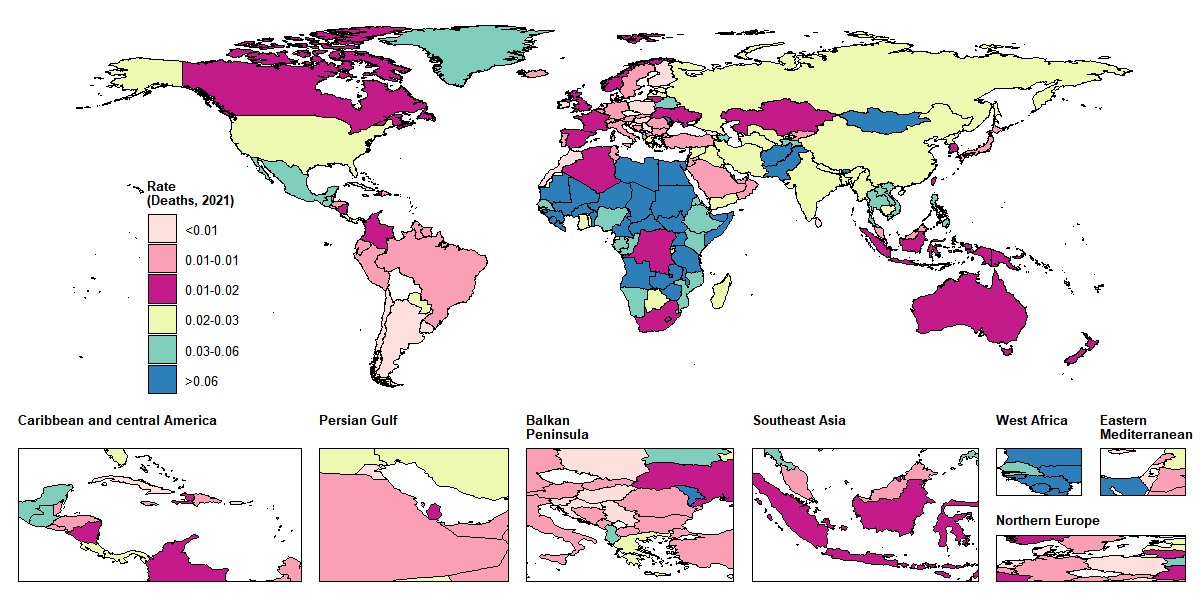


EAPC


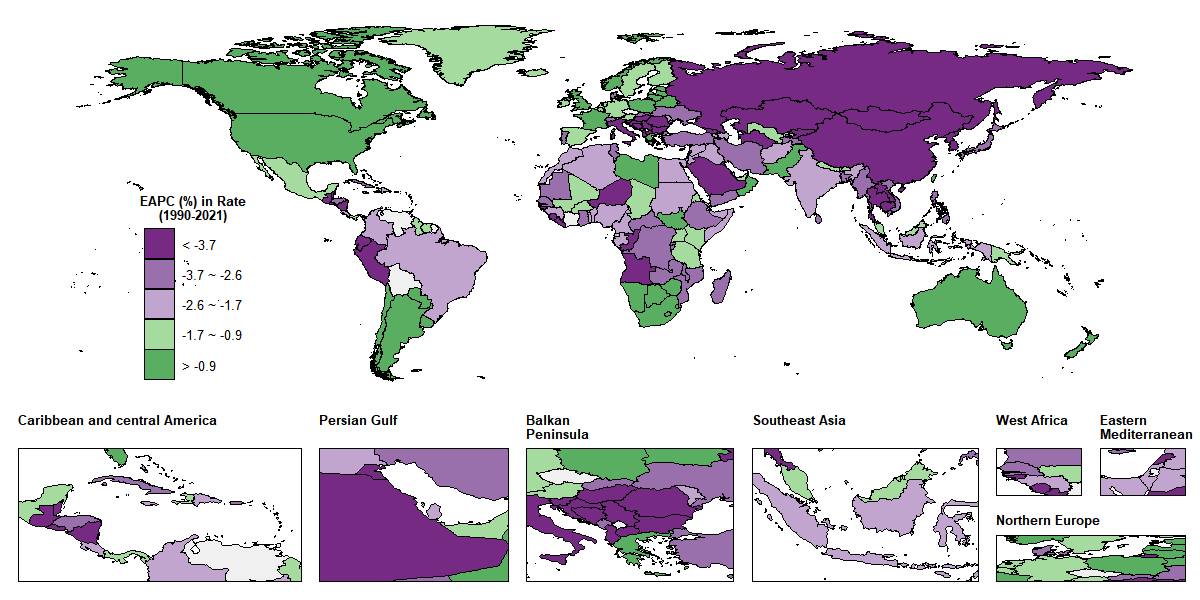


**C**

DALYs rate


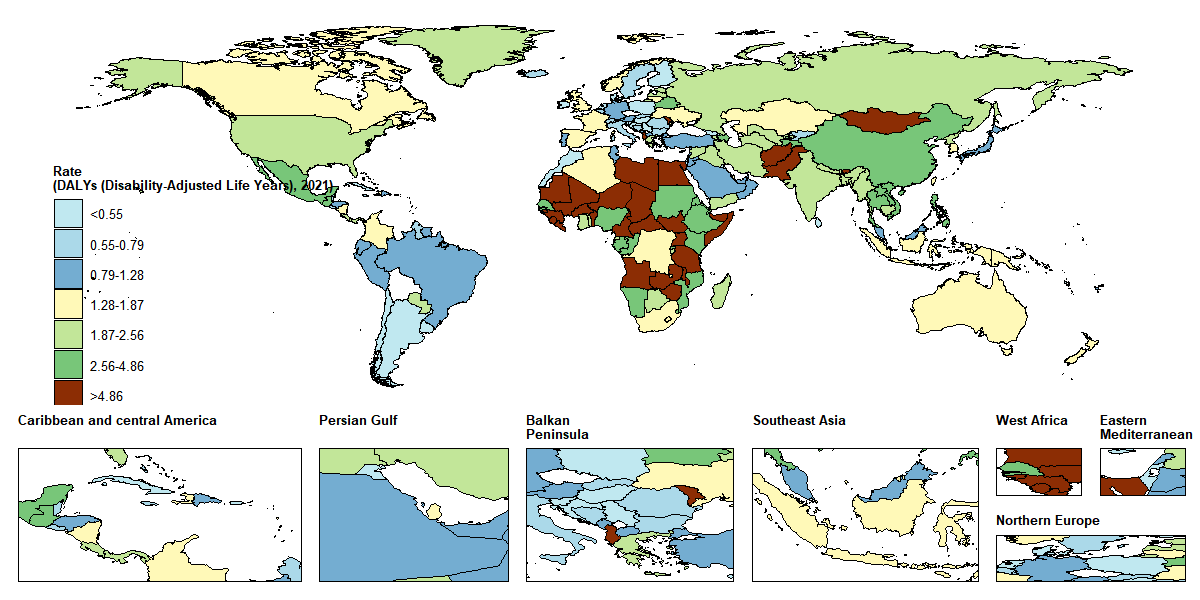


EAPC


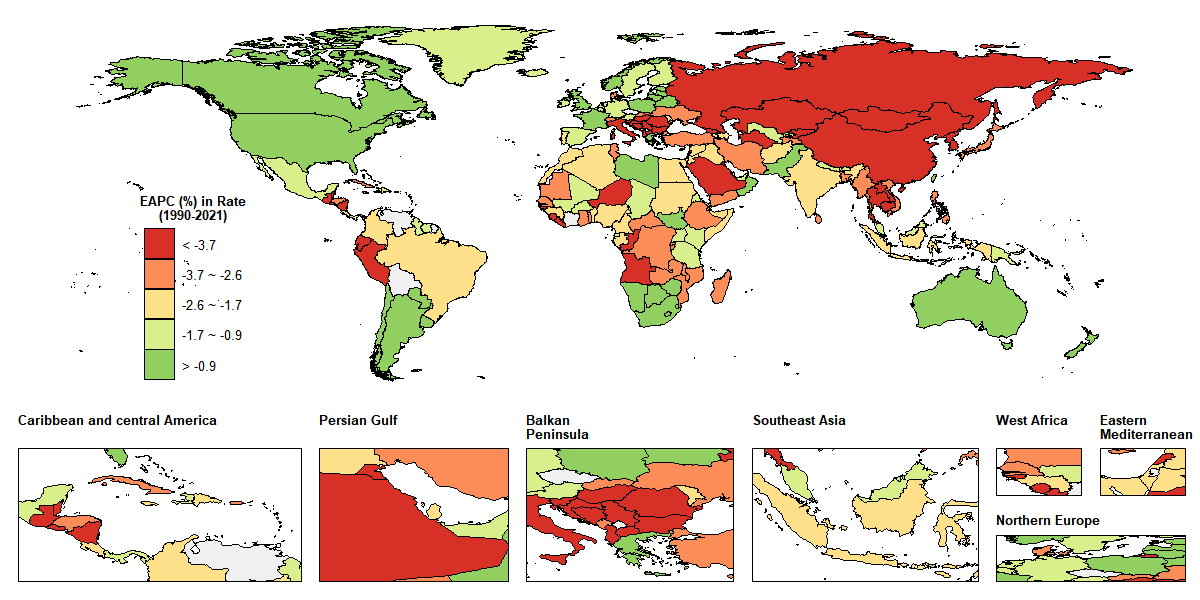


This figure demonstrates rates and estimated annual percentage change (EAPC) of incidence (A), deaths (B), and disability-adjusted life-years (DALYs) (C) attributable to hepatoblastoma across 204 countries and territories in 2021.

**Figure 9. Global maps of rates and estimated annual percentage change (EAPC) of incidence, deaths, and disability-adjusted life-years (DALYs) attributable to liver cancer due to other causes in 2021**

**A**

**Liver cancer due to other causes**

Incidence rate


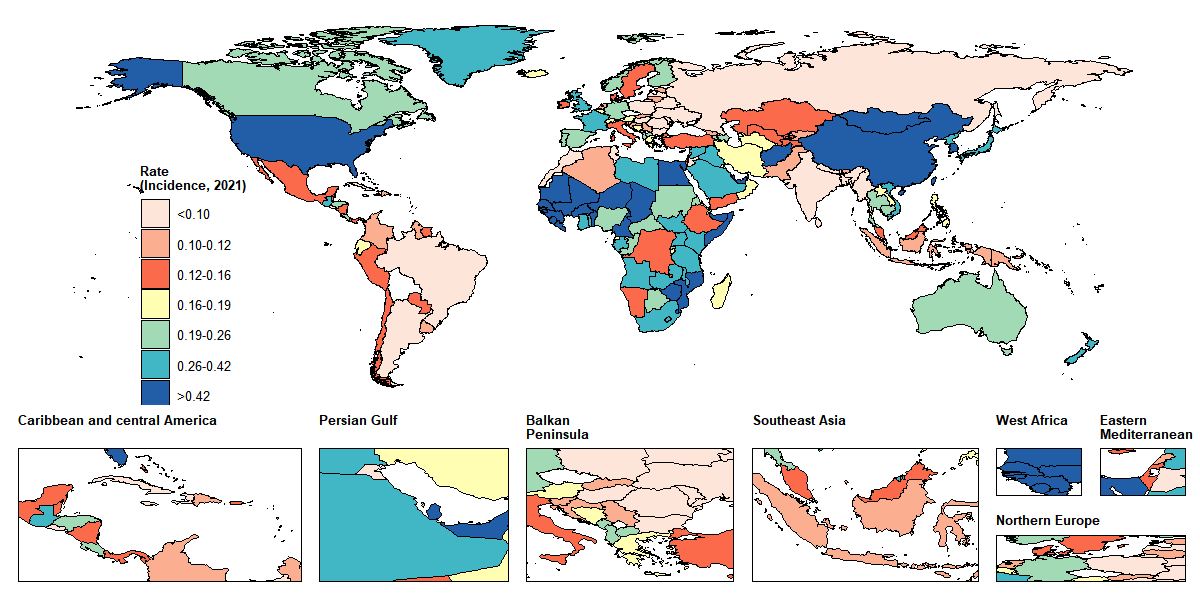


EAPC


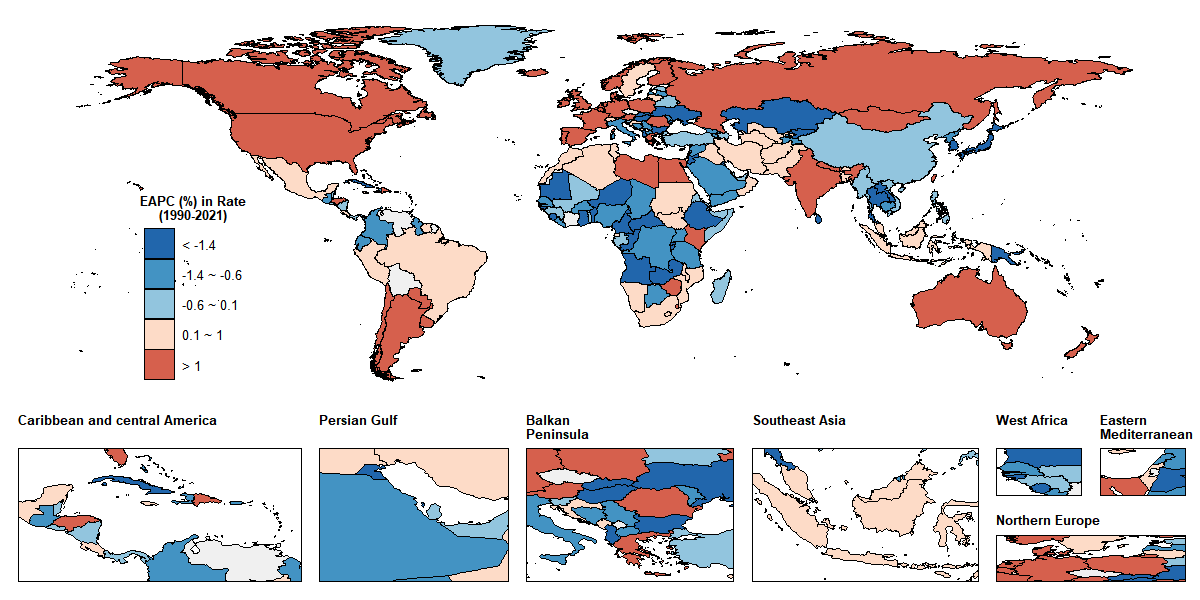


**B**

Deaths rate


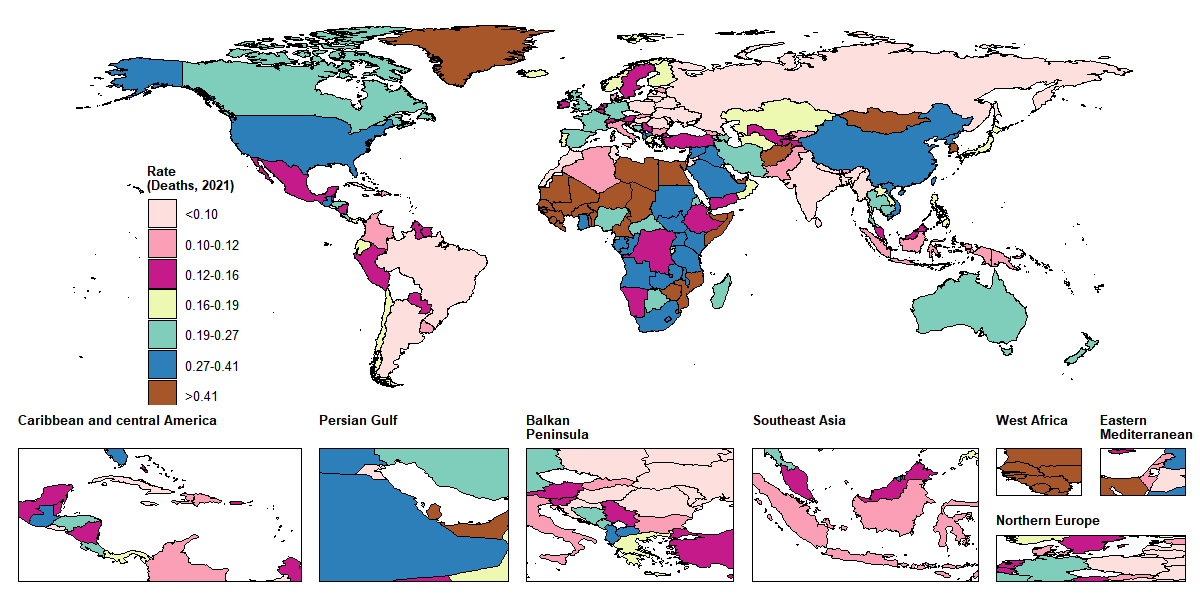


EAPC


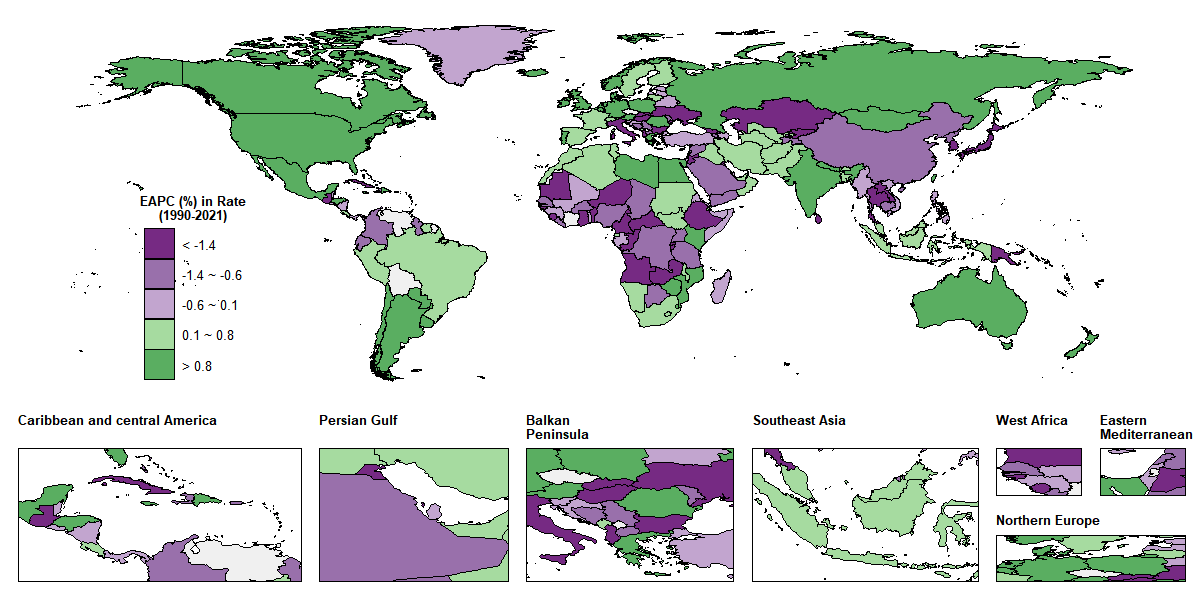


**C**

DALYs rate


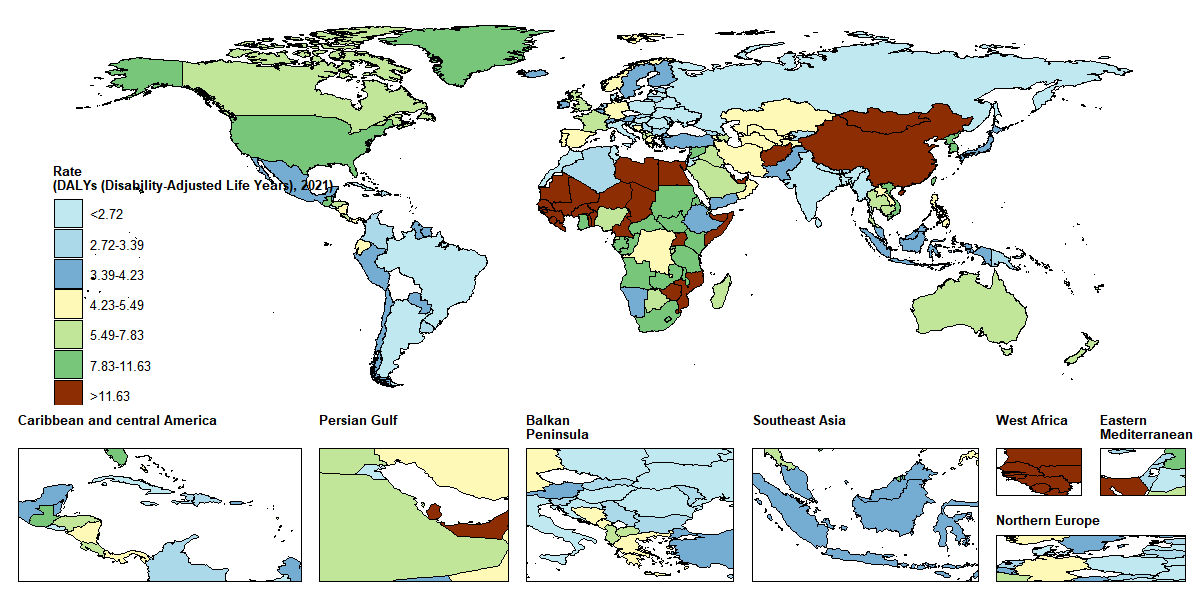


EAPC


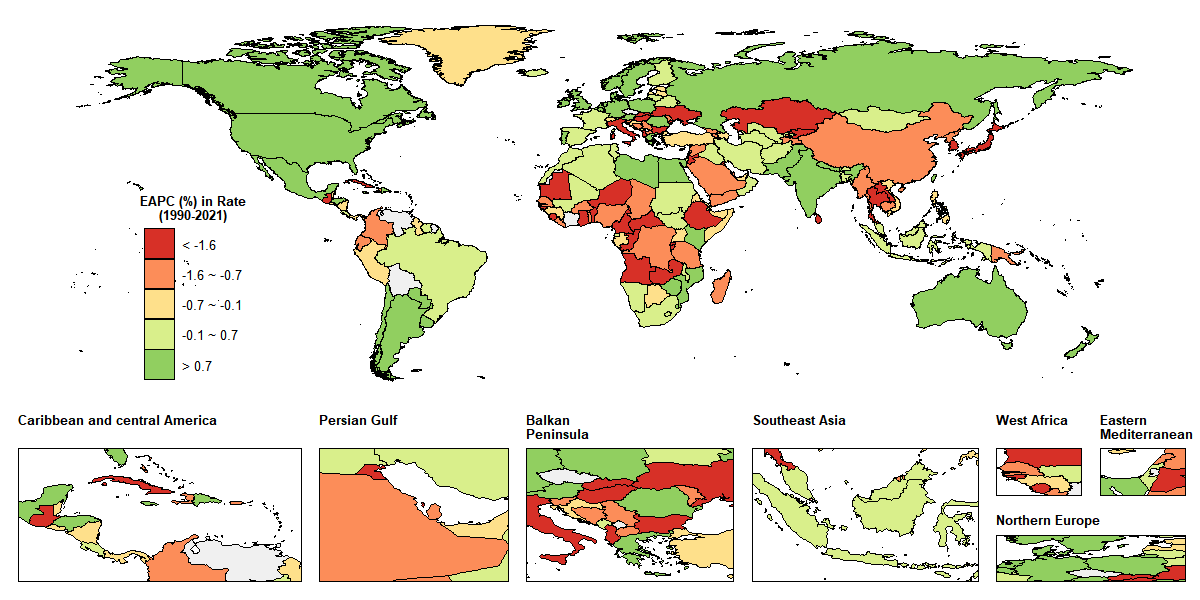


This figure demonstrates rates and estimated annual percentage change (EAPC) of incidence (A), deaths (B), and disability-adjusted life-years (DALYs) (C) attributable to liver cancer due to other causes across 204 countries and territories in 2021.

**Figure 10. Inequality analysis of DALYs attributable to liver cancer, 1990-2021**

**A**

**Liver cancer due to non-alcoholic steatohepatitis (NASH)**


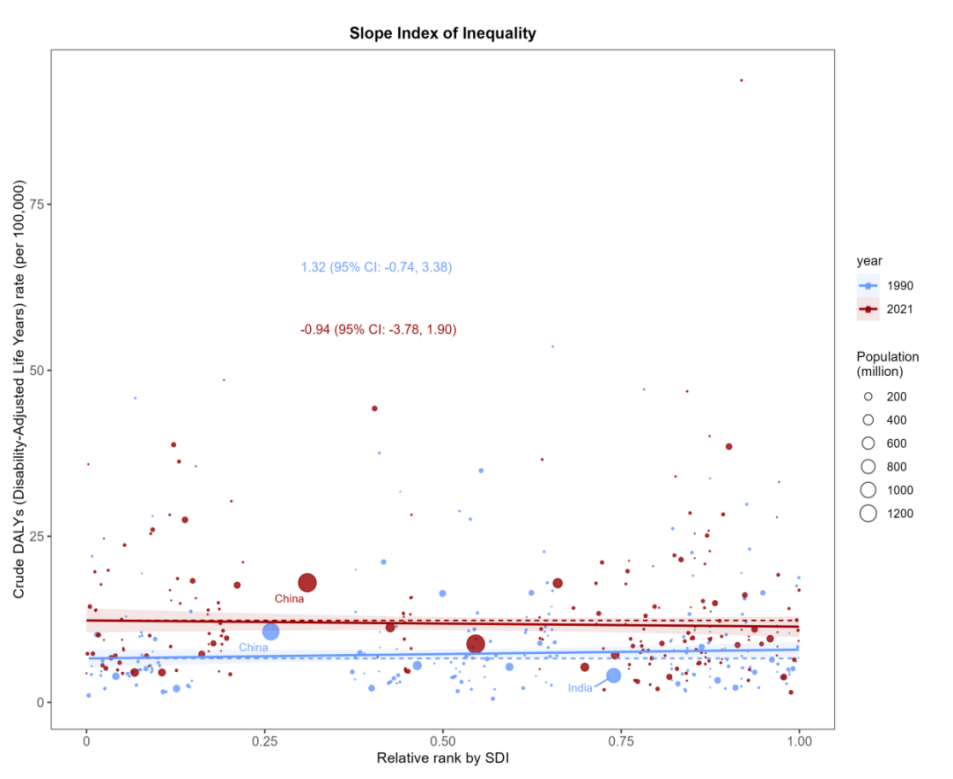


**B**


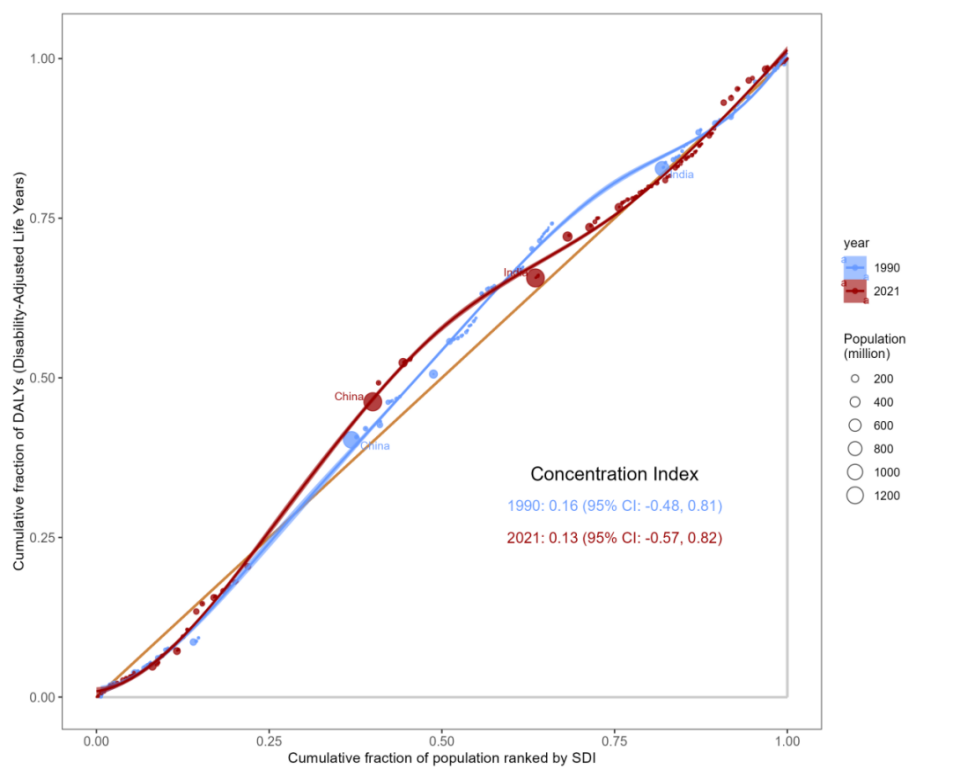


**C**

**Liver cancer due to hepatitis B**


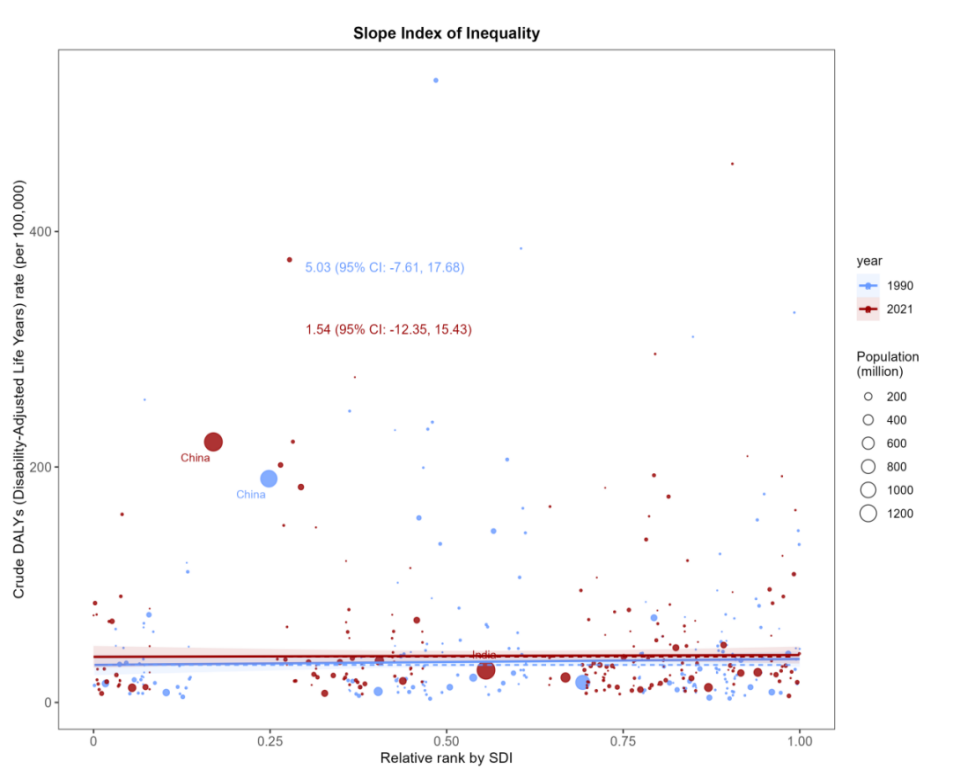


**D**


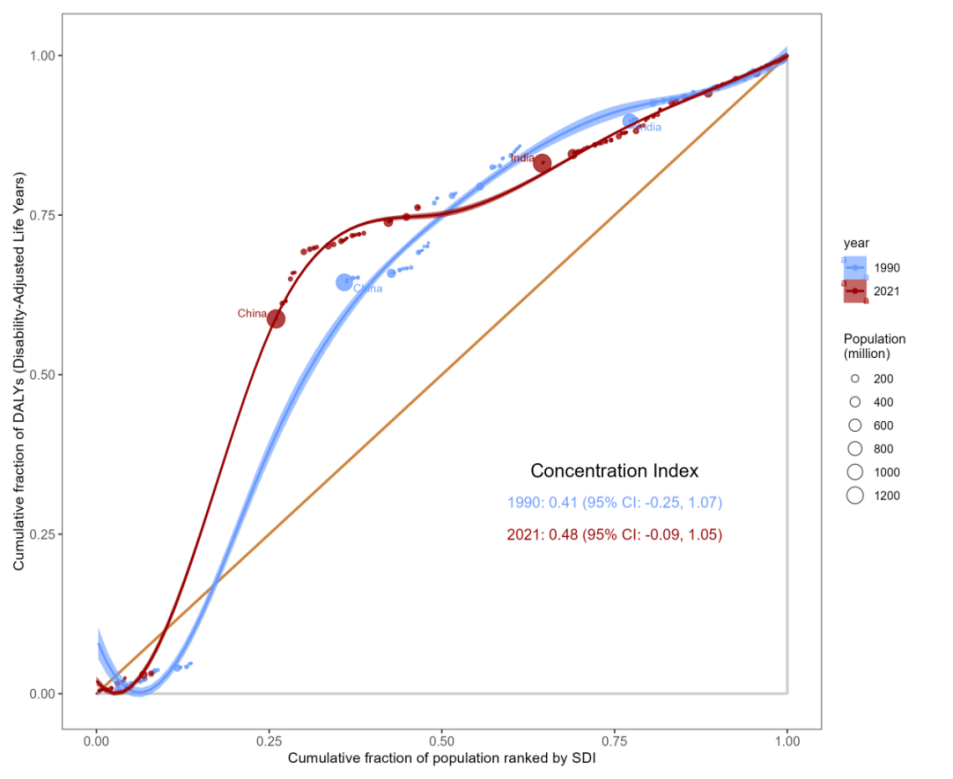


**E**

**Liver cancer due to hepatitis C**


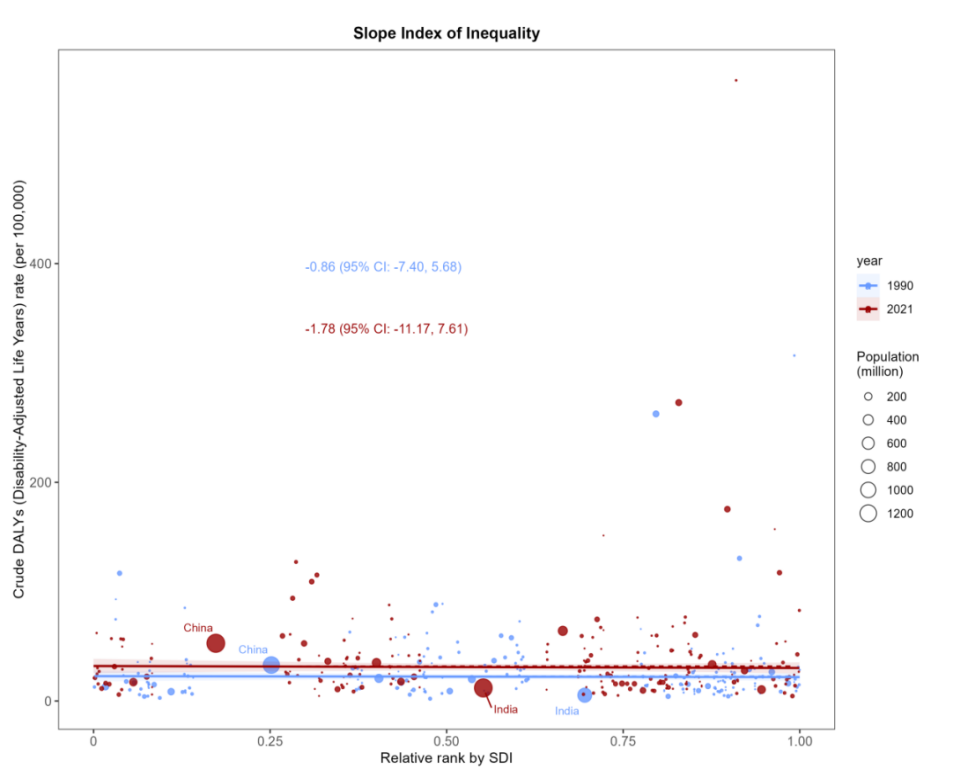


**F**


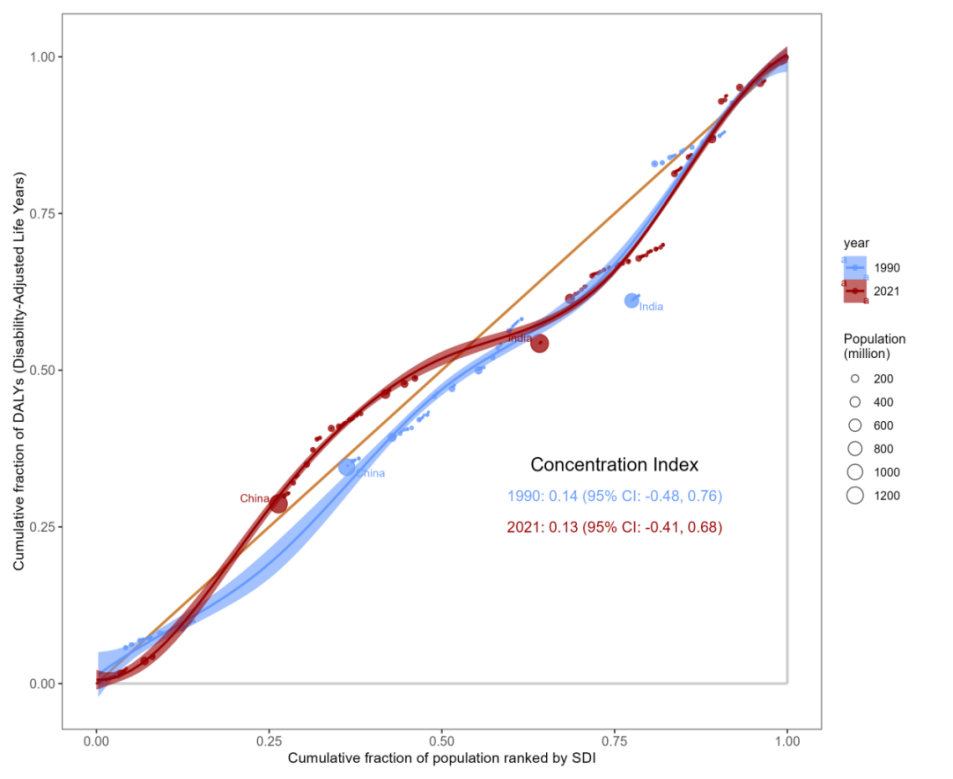


**G**

**Liver cancer due to alcohol use**


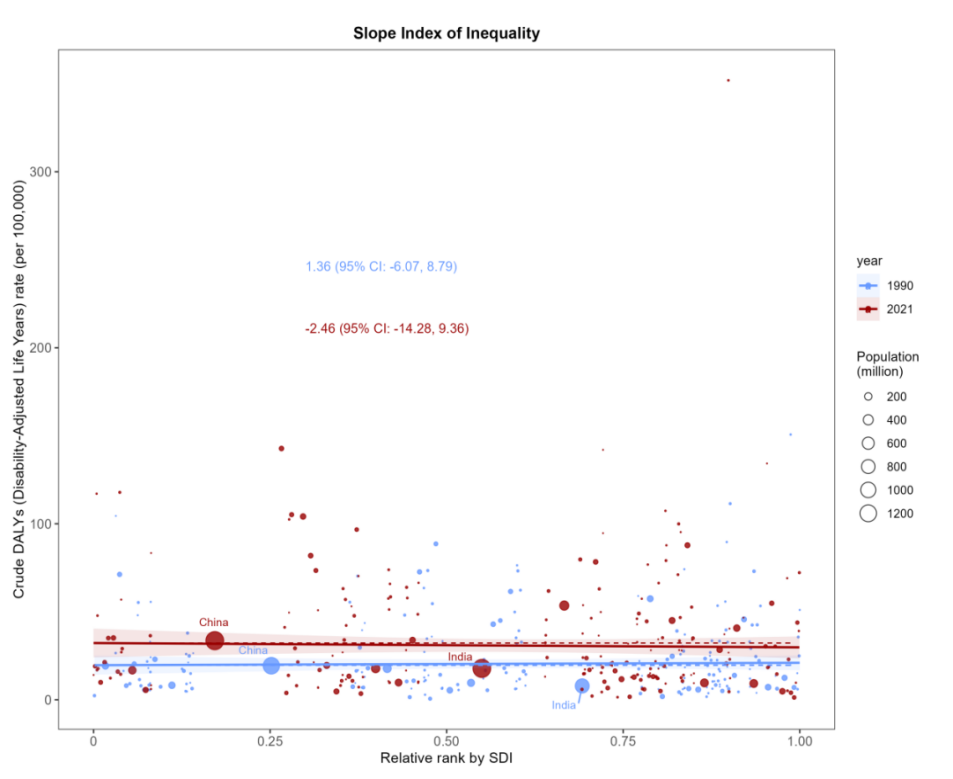


**H**


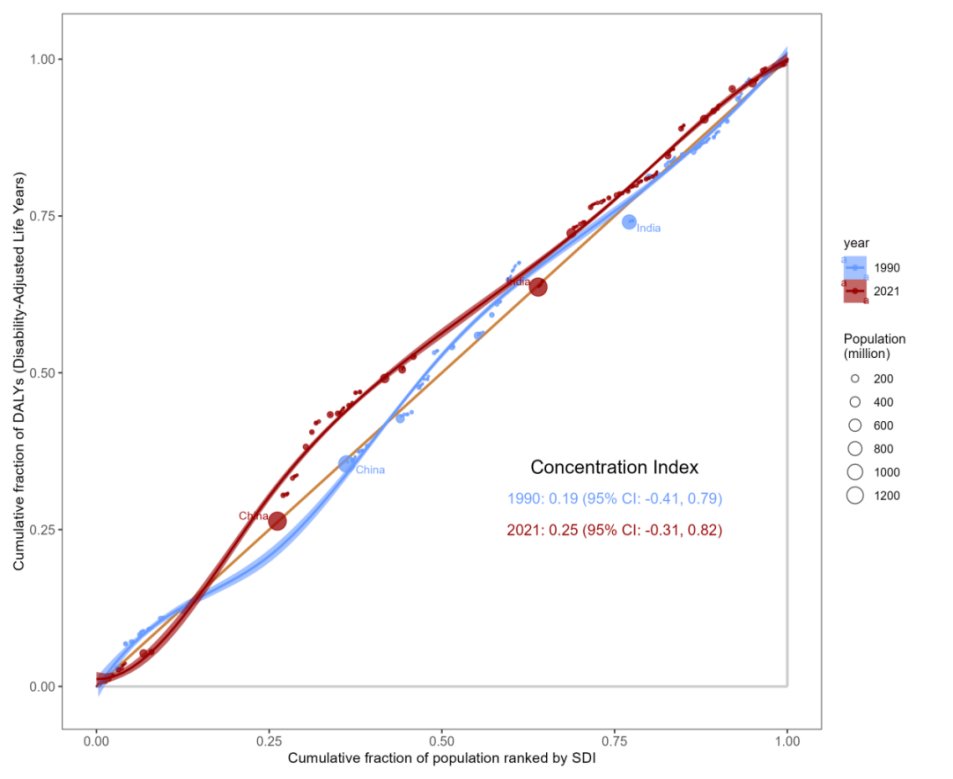


**I**

**Hepatoblastoma**


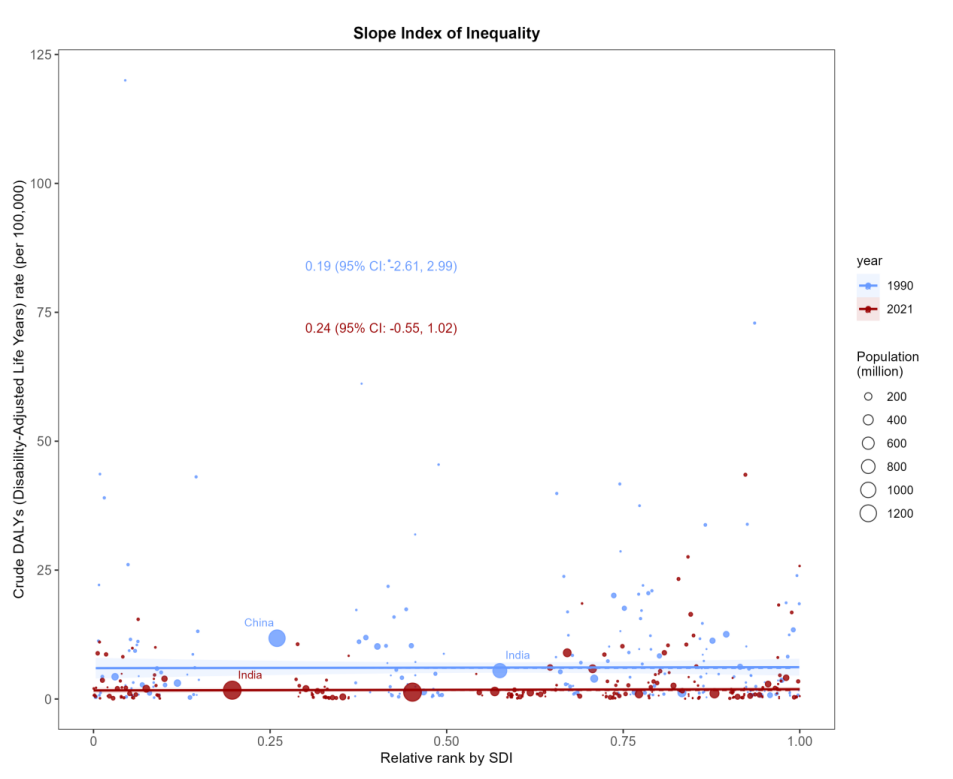


**J**


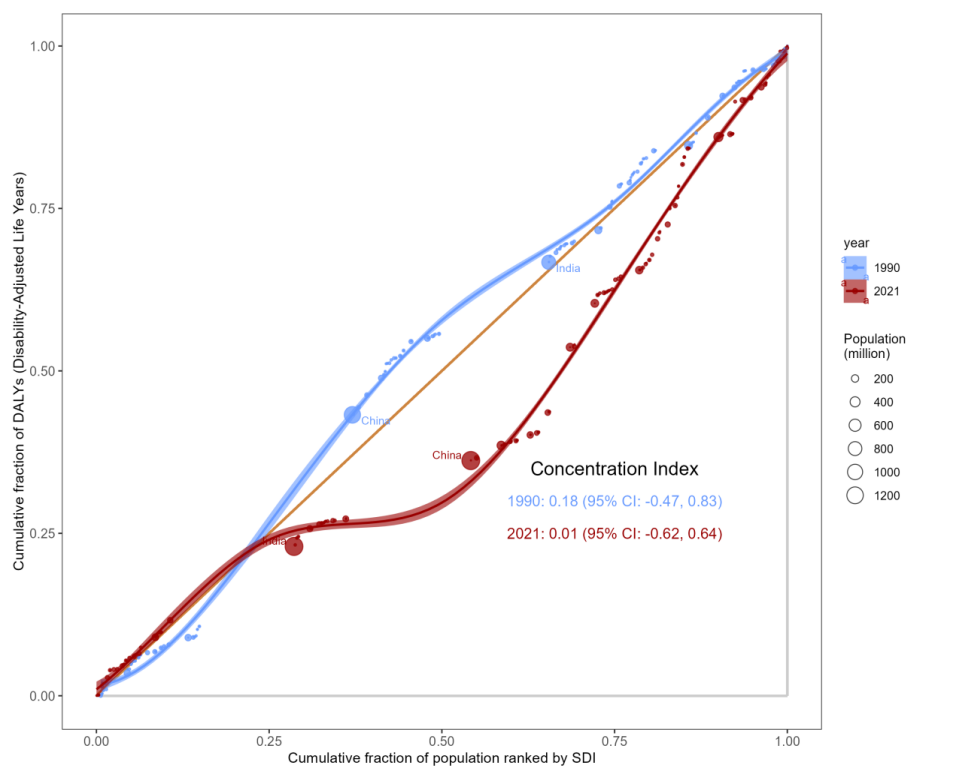


**K**

**Liver cancer due to other causes**


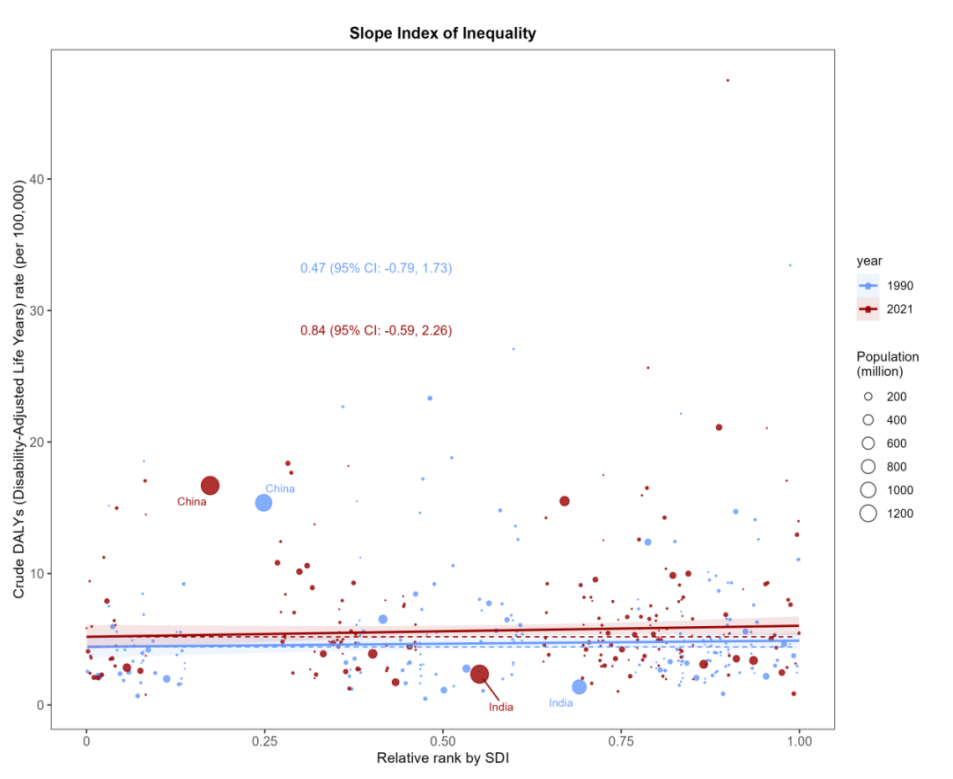


**L**


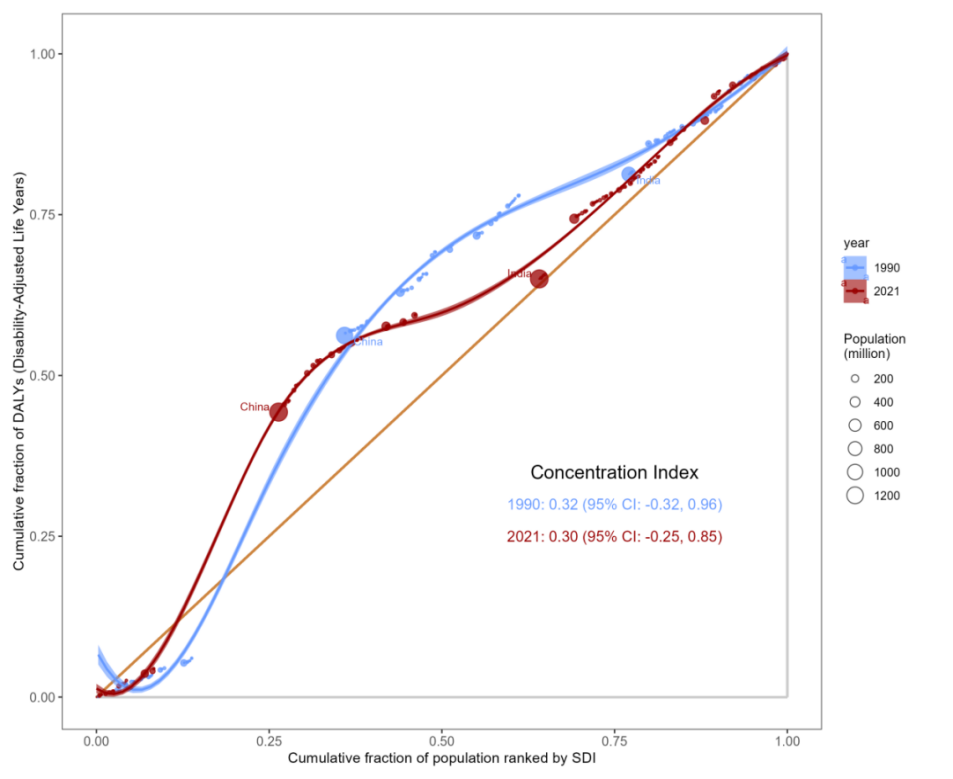


The inequality slope index and concentration index for DALYs of liver cancer due to non-alcoholic steatohepatitis (NASH) (A-B), liver cancer due to hepatitis B (C-D), liver cancer due to hepatitis B (E-F), liver cancer due to alcohol use (G-H), hepatoblastoma (I-J), and liver cancer due to other causes (K-L) worldwide in 1990 and 2021. A, C, E, G, and I illustrate the inequality slope index, depicting the relationship between SDI and age-standardised DALYs rates for each condition, with points representing individual countries sized by population. B, D, F, H, and J present the concentration index, which quantifies relative inequalities by integrating the area under the Lorenz curve, aligning DALYs distribution with population distribution by SDI. Blue represents data from 1990, and red represents data from 2021.

**Figure 11. Projected burden of liver cancer due to non-alcoholic steatohepatitis (NASH) by 2040**

**A**


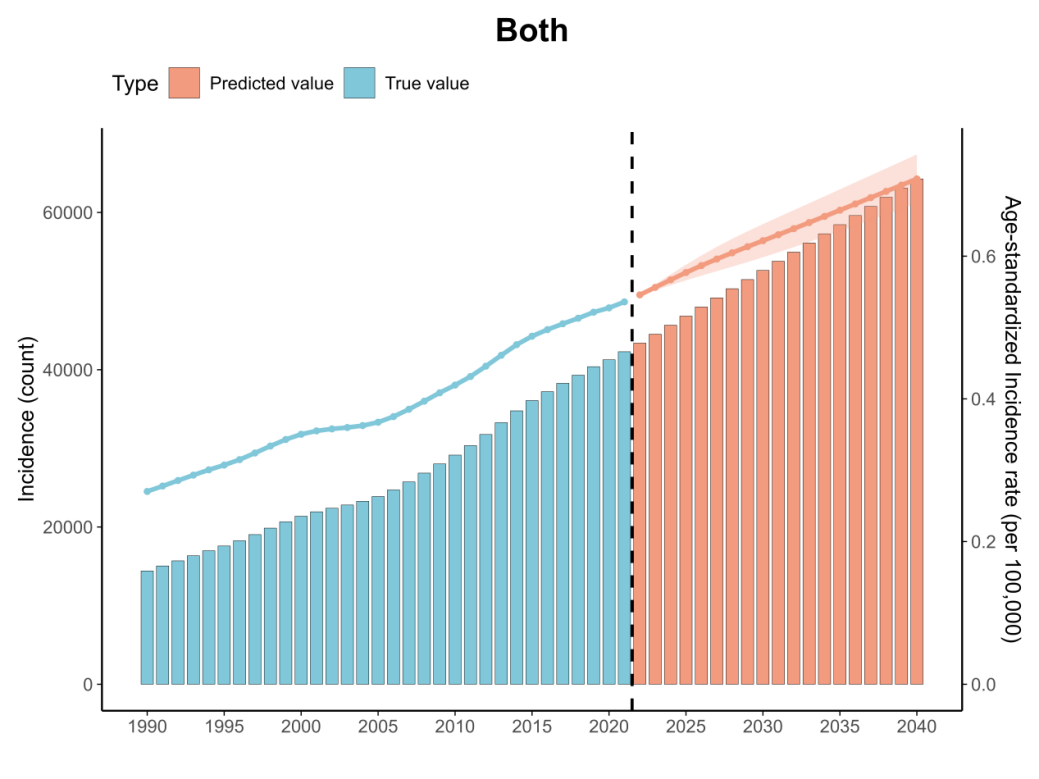


**B**


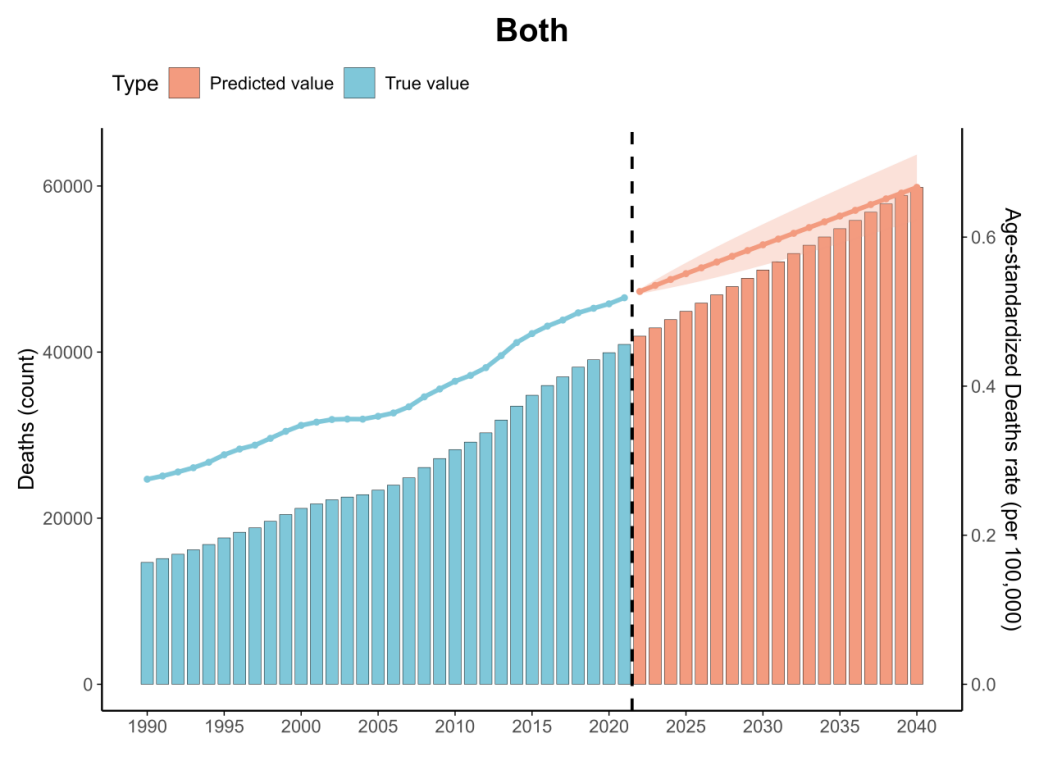


**C**


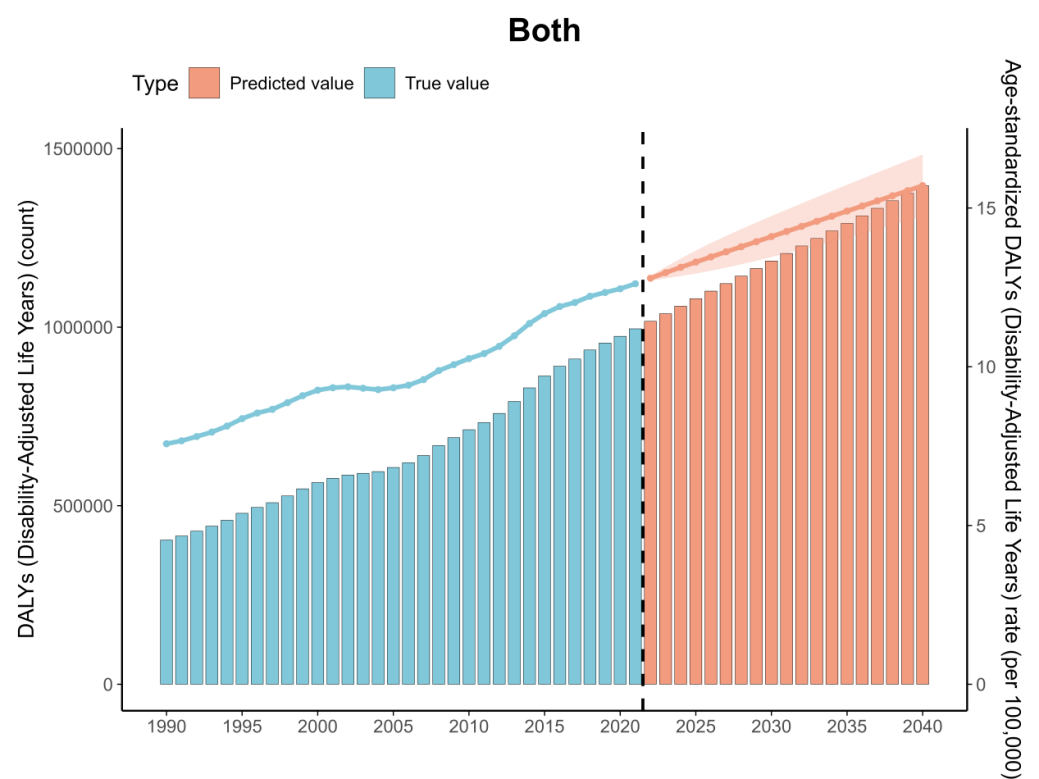


This figure demonstrates projected number and rates of incidence (A), deaths (B), and disability-adjusted life-years (DALYs) (C) attributable to liver cancer due to non-alcoholic steatohepatitis (NASH) by 2040. Blue represents actual data, and orange represents forecast data.

**Figure 12. Projected burden of liver cancer due to hepatitis B by 2040**

**A**


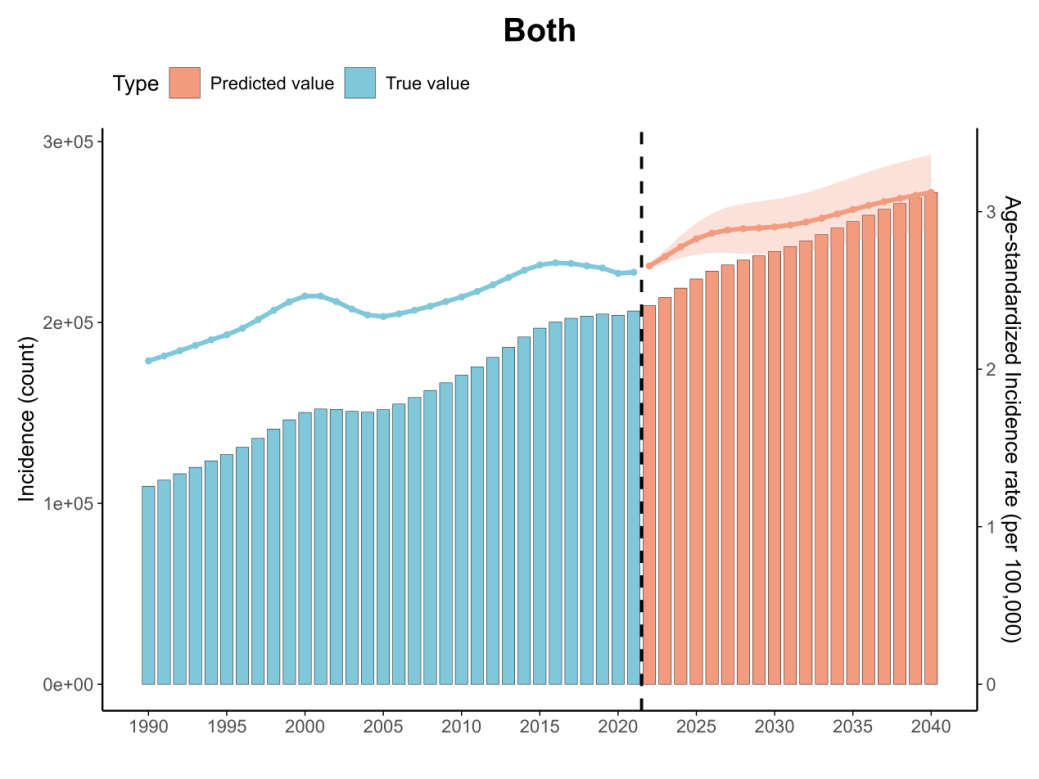


**B**


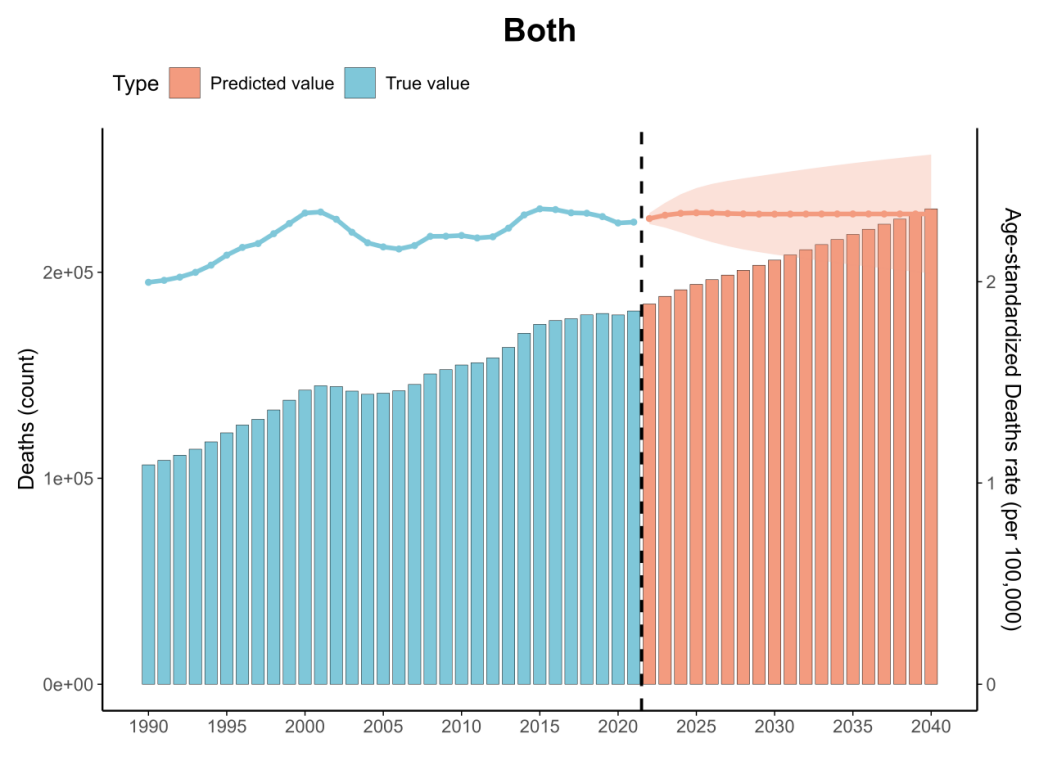


**C**


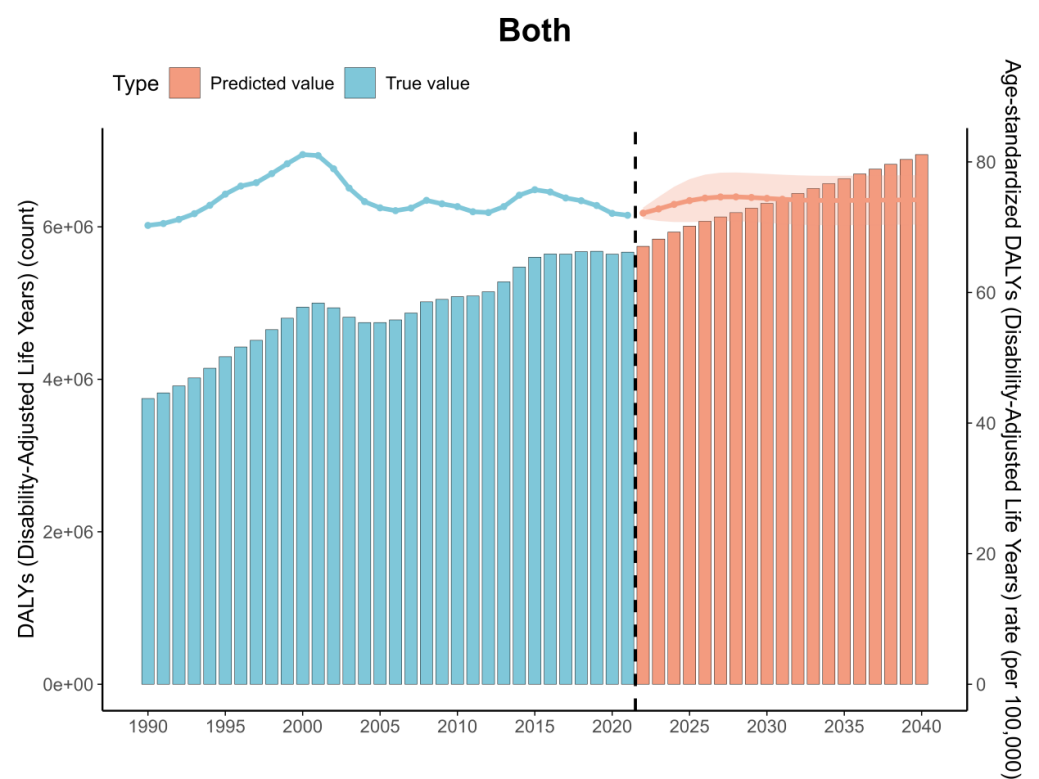


This figure demonstrates projected number and rates of incidence (A), deaths (B), and disability-adjusted life-years (DALYs) (C) attributable to liver cancer due to hepatitis B by 2040. Blue represents actual data, and orange represents forecast data.

**Figure 13. Projected burden of liver cancer due to hepatitis C by 2040**

**A**


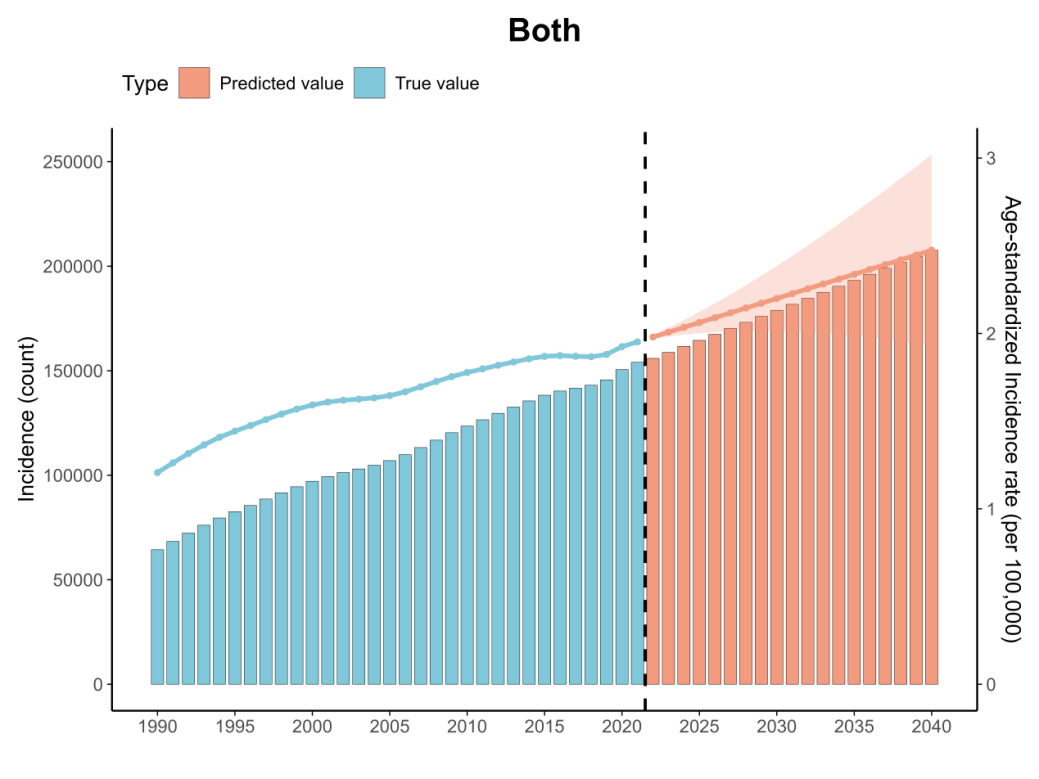


**B**


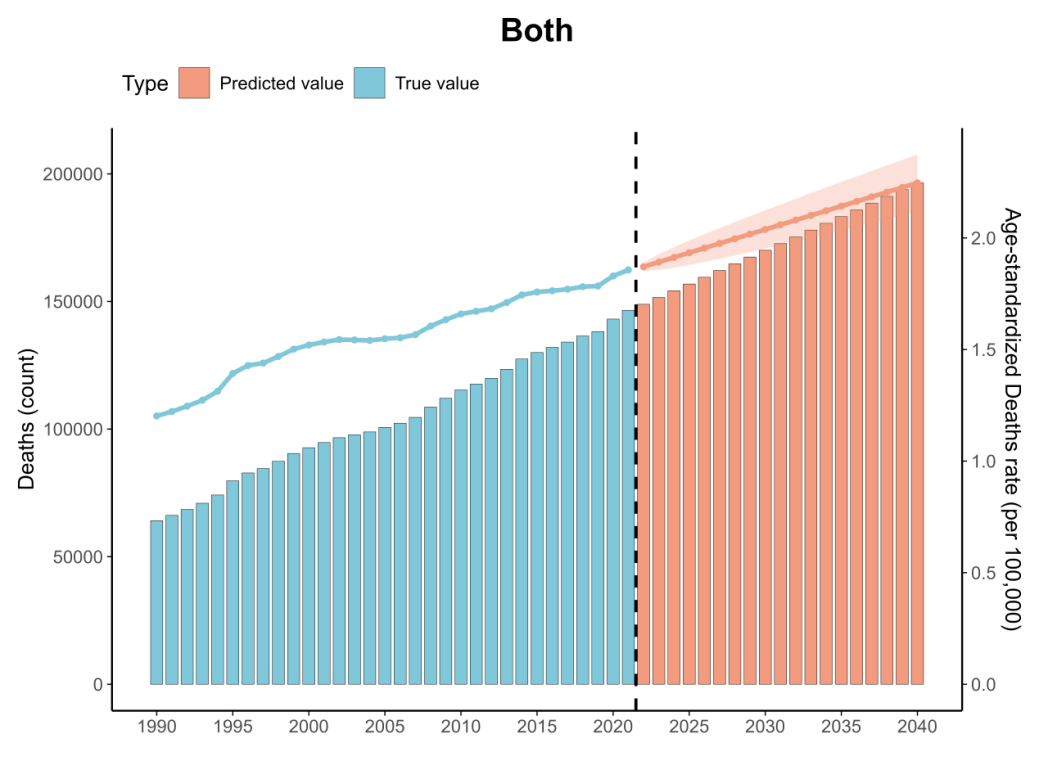


**C**


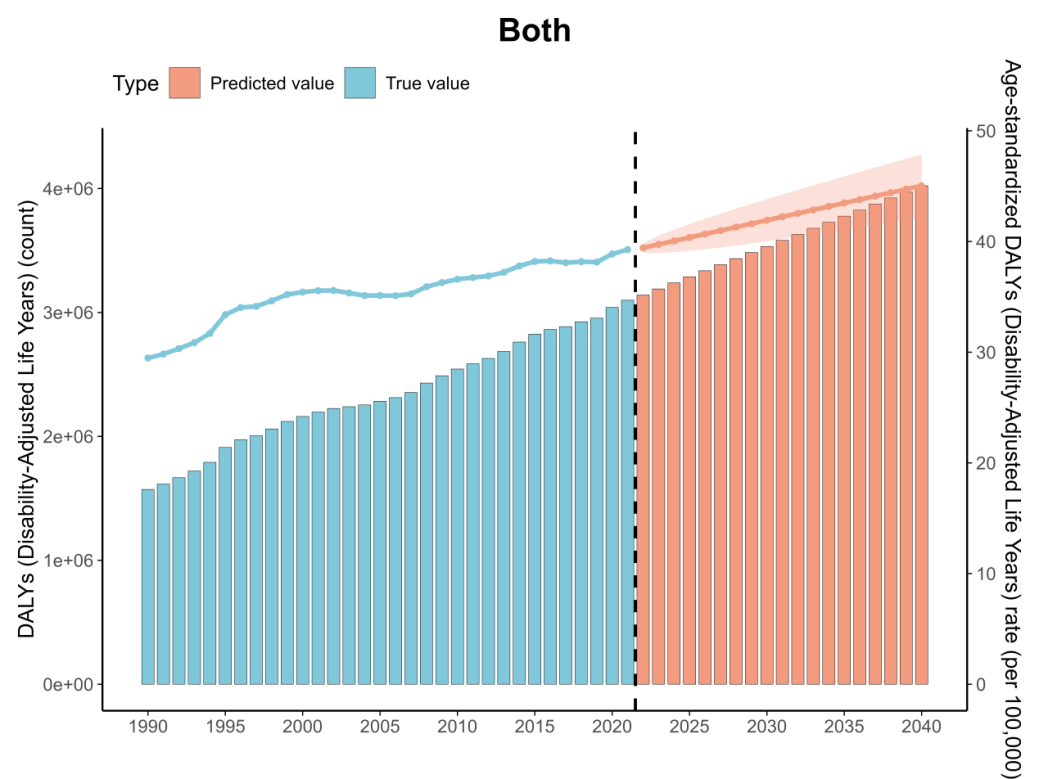


This figure demonstrates projected number and rates of incidence (A), deaths (B), and disability-adjusted life-years (DALYs) (C) attributable to liver cancer due to hepatitis C by 2040. Blue represents actual data, and orange represents forecast data.

**Figure 14. Projected burden of liver cancer due to alcohol use by 2040**

**A**


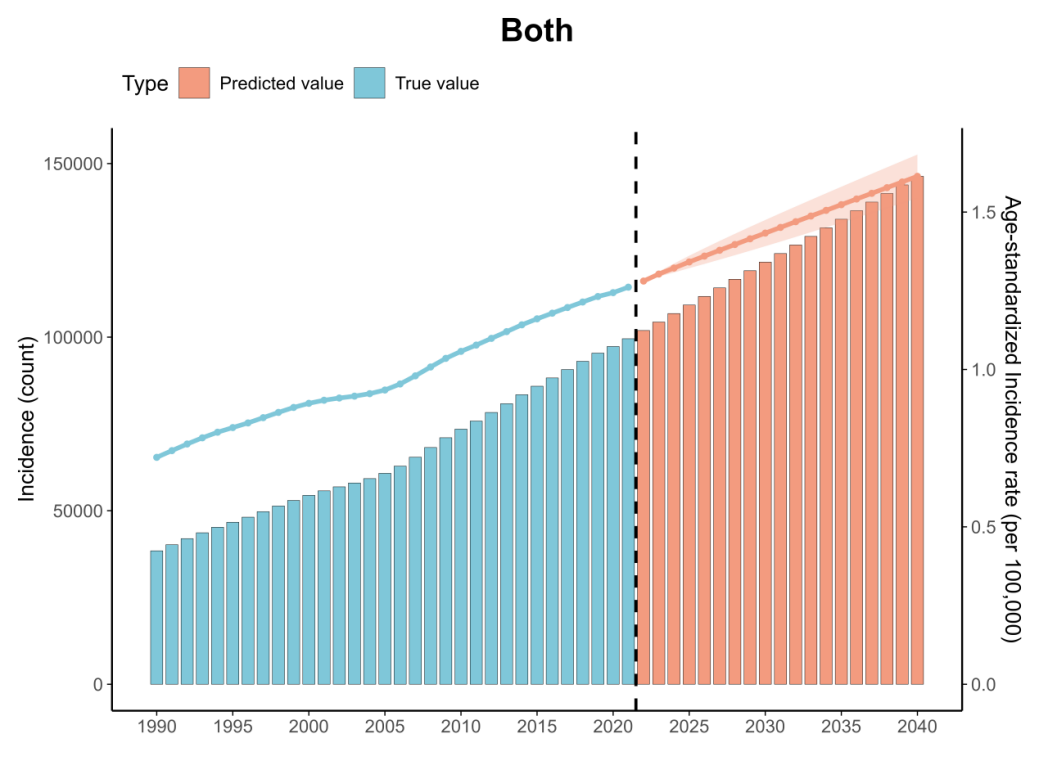


**B**


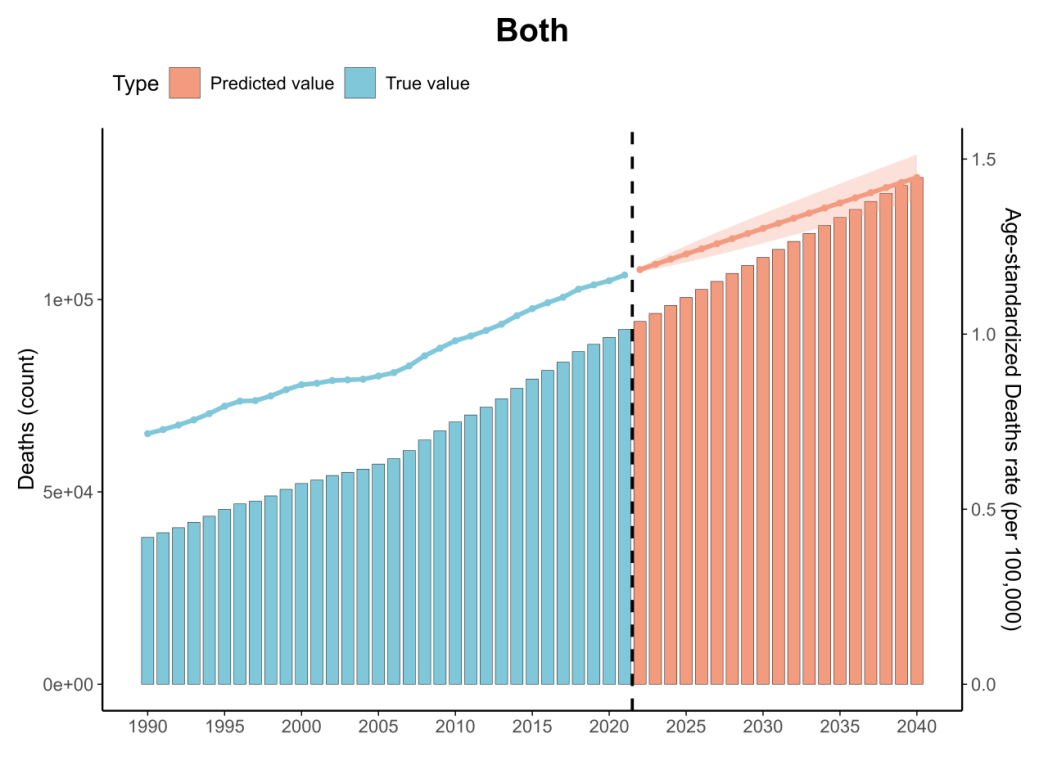


**C**


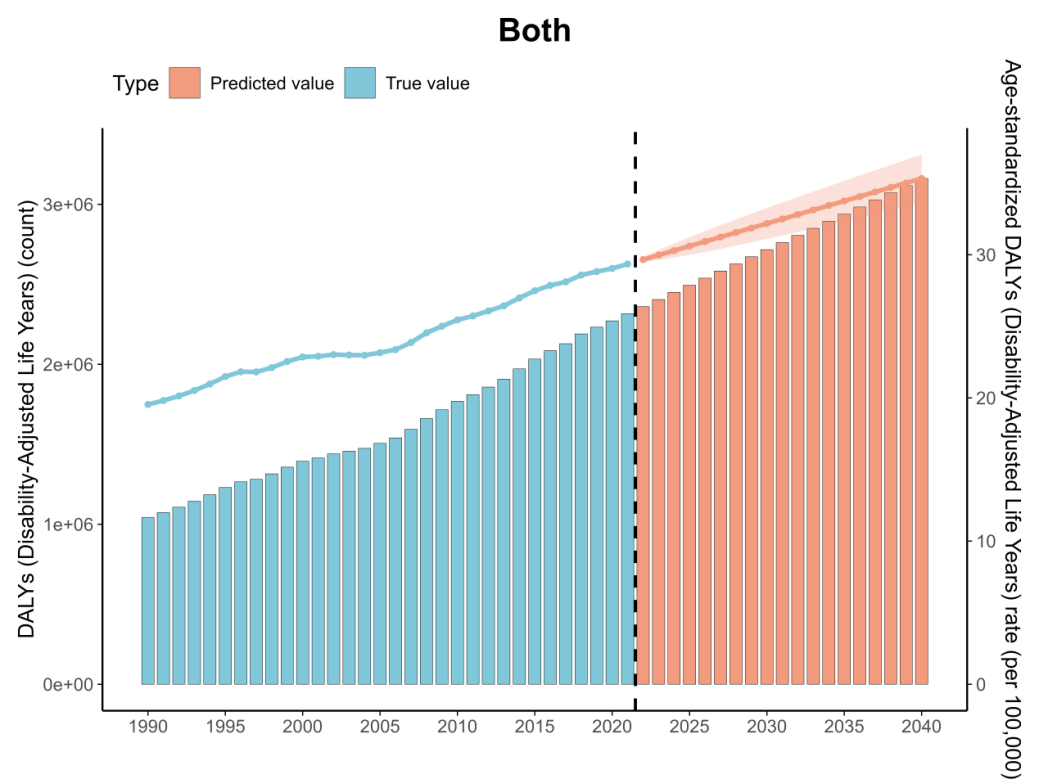


This figure demonstrates projected number and rates of incidence (A), deaths (B), and disability-adjusted life-years (DALYs) (C) attributable to liver cancer due to alcohol use by 2040. Blue represents actual data, and orange represents forecast data.

**Figure 15. Projected burden of hepatoblastoma by 2040**

**A**

**
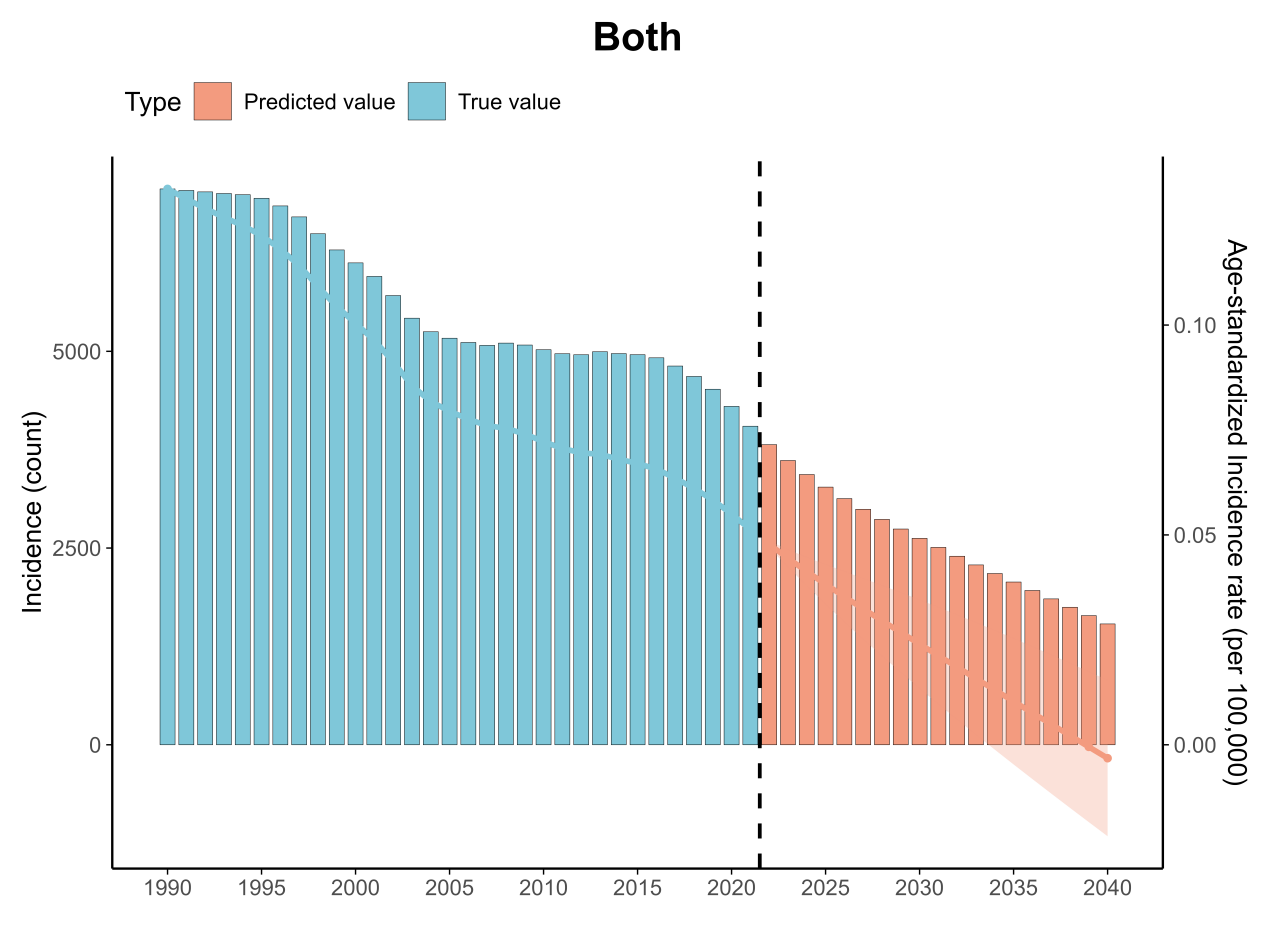
**

**B**


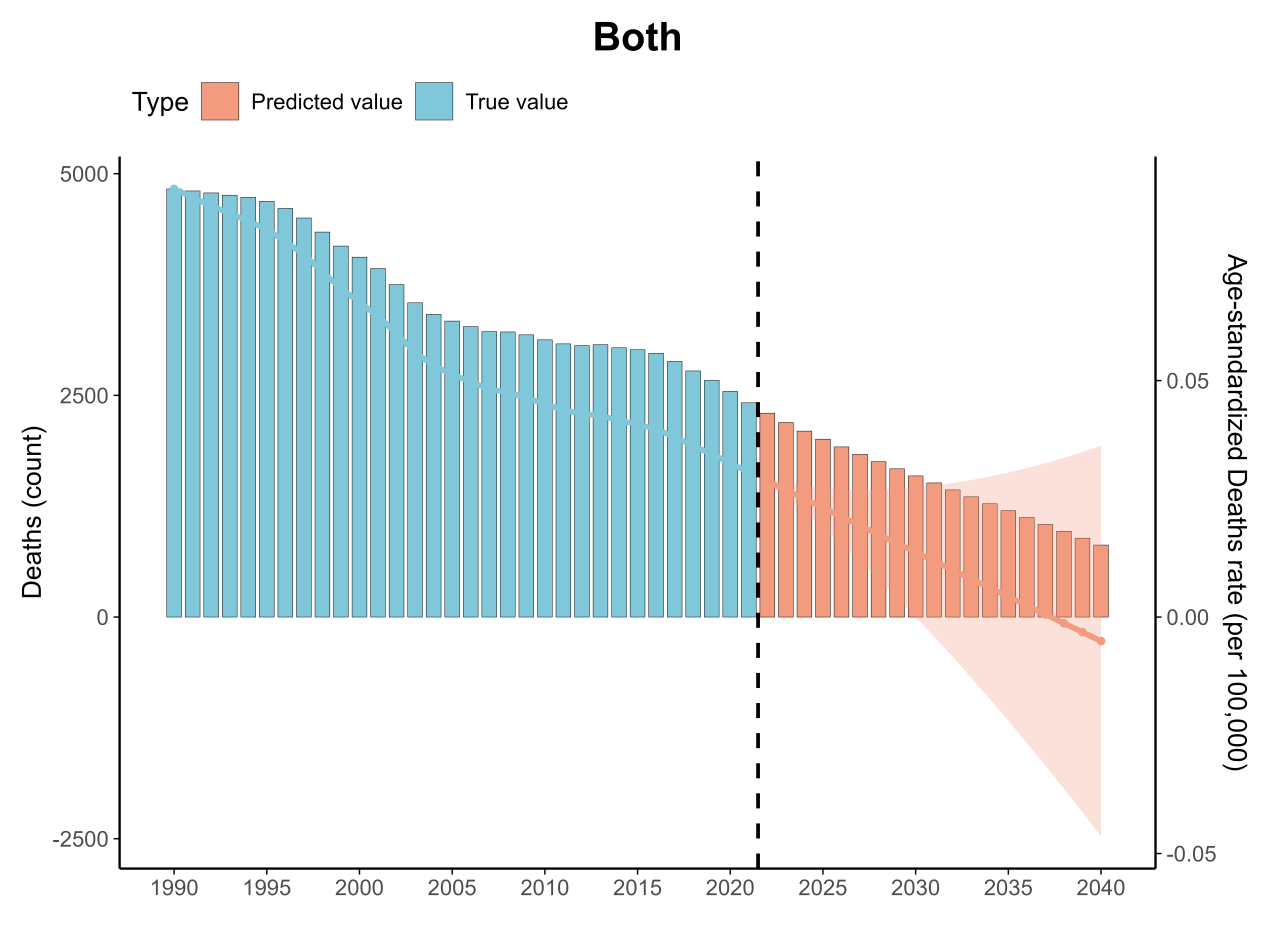


**C**


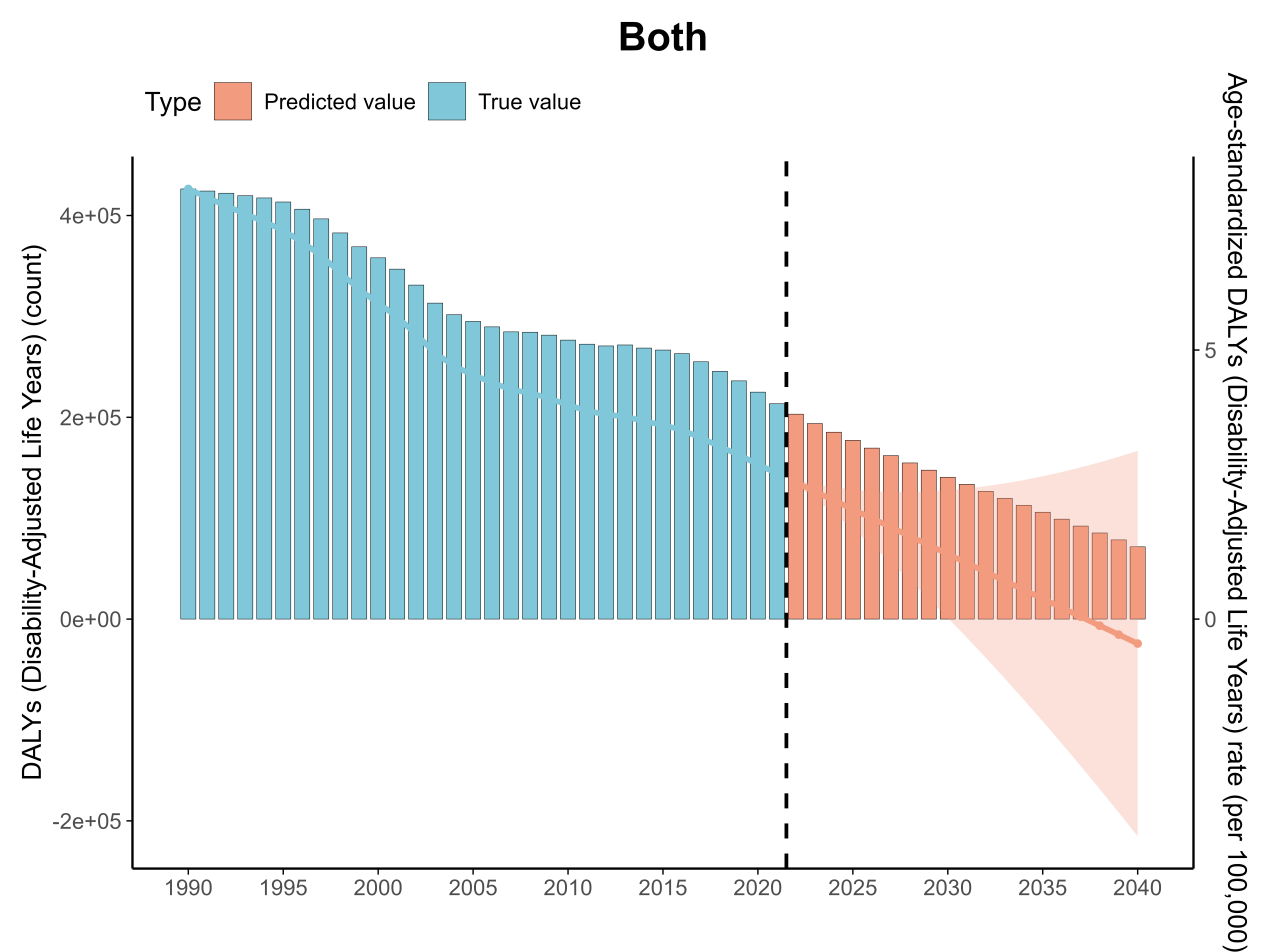


This figure demonstrates projected number and rates of incidence (A), deaths (B), and disability-adjusted life-years (DALYs) (C) attributable to hepatoblastoma by 2040. Blue represents actual data, and orange represents forecast data.

**Figure 16. Projected burden of liver cancer due to other causes by 2040**

**A**


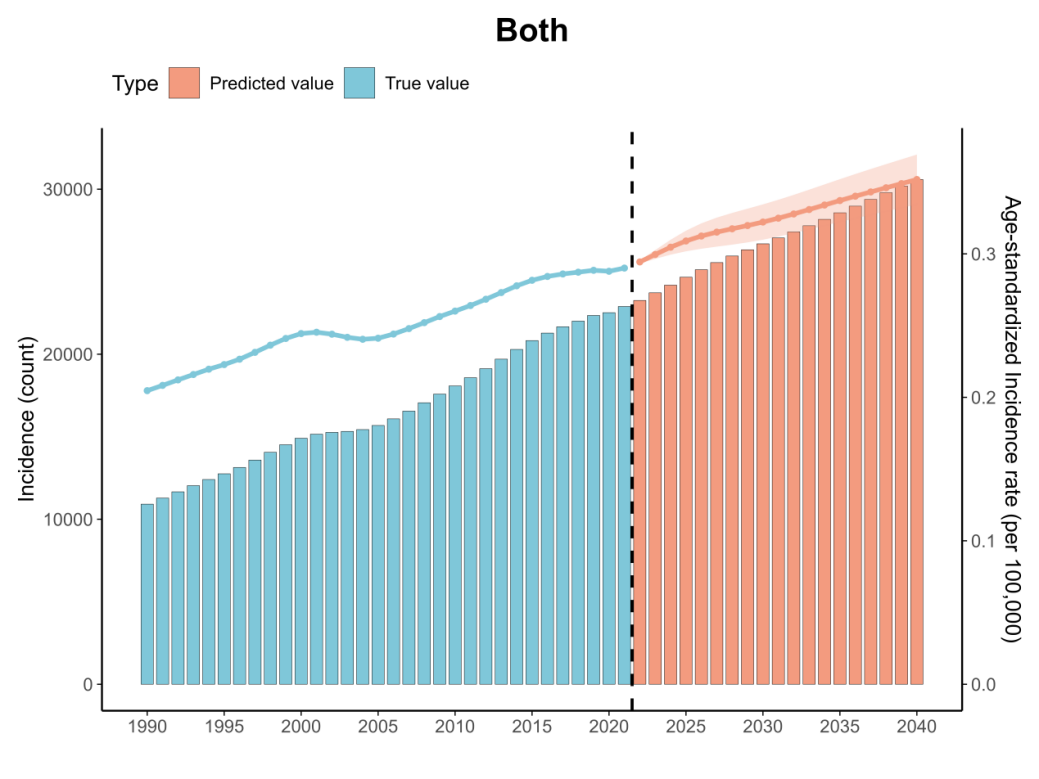


**B**


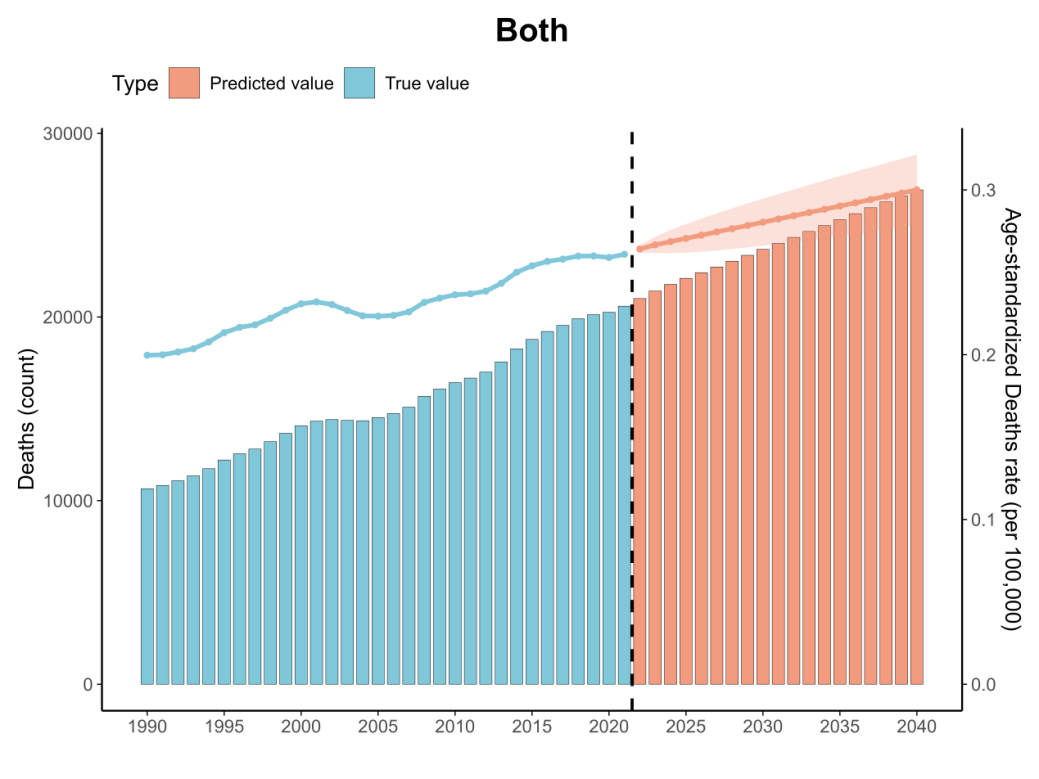


**C**


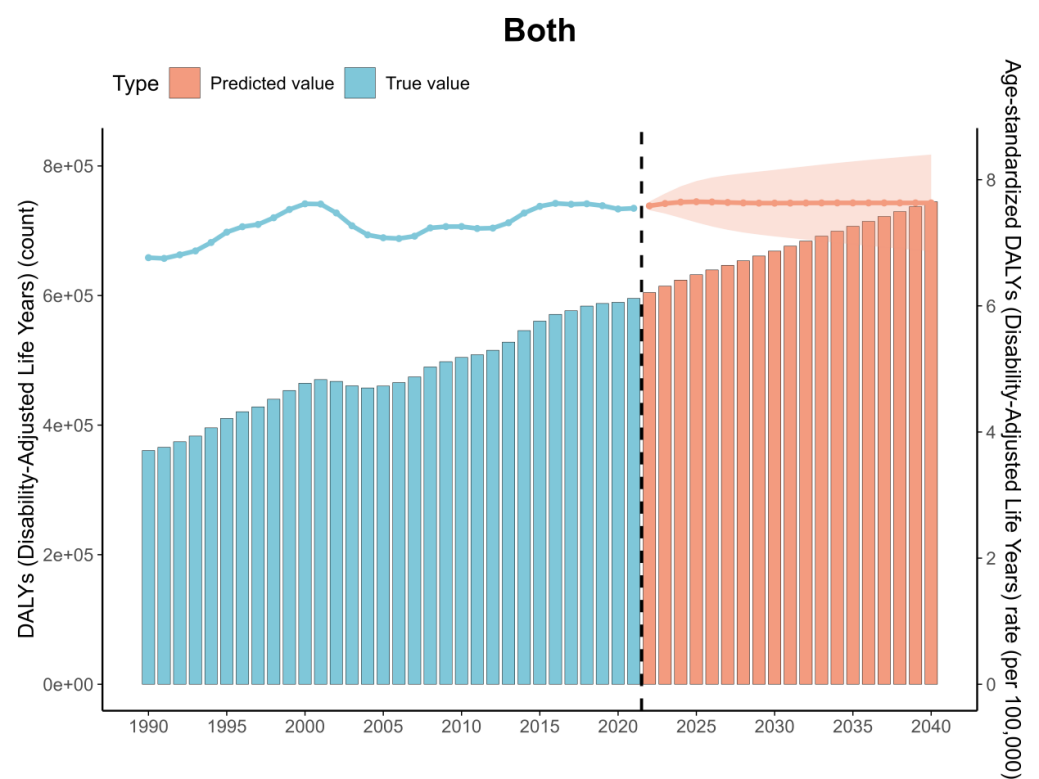


This figure demonstrates projected number and rates of incidence (A), deaths (B), and disability-adjusted life-years (DALYs) (C) attributable to liver cancer due to other causes by 2040. Blue represents actual data, and orange represents forecast data.
